# Palladium-catalyzed redox diversified entry to axially chiral styrenes *via* asymmetric olefination with alkynes†

**DOI:** 10.1039/d5sc04080a

**Published:** 2025-07-04

**Authors:** Ziwei Lin, Yuqing Jiang, Fen Wang, Genping Huang, Jessica Li, Xingwei Li

**Affiliations:** a School of Chemistry and Chemical Engineering, Shaanxi Normal University (SNNU) Xi'an 710062 P. R. China lixw@snnu.edu.cn fenwang@snnu.edu.cn; b Department of Chemistry, School of Science, Tianjin University Tianjin 300072 P. R. China gphuang@tju.edu.cn; c School of Arts and Sciences, Brandeis University Waltham MA 02453 USA; d Institute of Molecular Science and Engineering, Institute of Frontier and Interdisciplinary Sciences, Shandong University Qingdao 266237 P. R. China

## Abstract

Axially chiral olefins represent an underexplored class of atropoisomers given their conformational flexibility and relatively low configurational stability. Atroposelective access to axially chiral olefins *via de novo* formation of a chiral axis is challenging. Reported herein is palladium-catalyzed redox-diversified olefination of aryl halides with two classes of bifunctional alkynes based on rational design of catalytic systems. The reductive Heck reaction (hydroarylation) of 1,6-diynes afforded axially chiral dienes using potassium formate as the reductant. In the case of 1-alkynylcyclobutanol substrates, ring strain-driven C–C coupling-olefination gave axially chiral, tetrasubstituted enones as the product. In both coupling systems, the reactions proceeded effectively with high regioselectivity, *Z*/*E* selectivity, and excellent enantioselectivity.

## Abstract 2

Axially chiral olefins represent an underexplored class of atropoisomers given their conformational flexibility and relatively low configurational stability.

## Graphical abstract

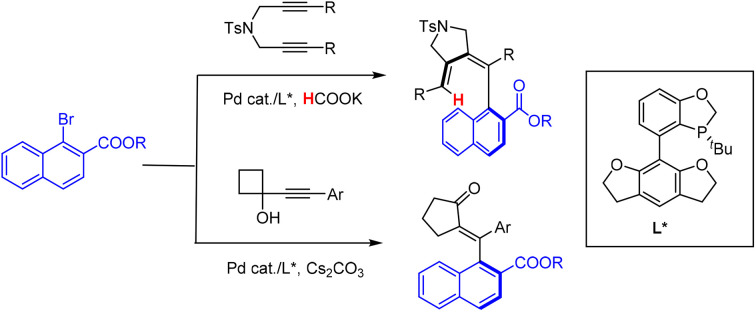

## Introduction

**Scheme 1:**
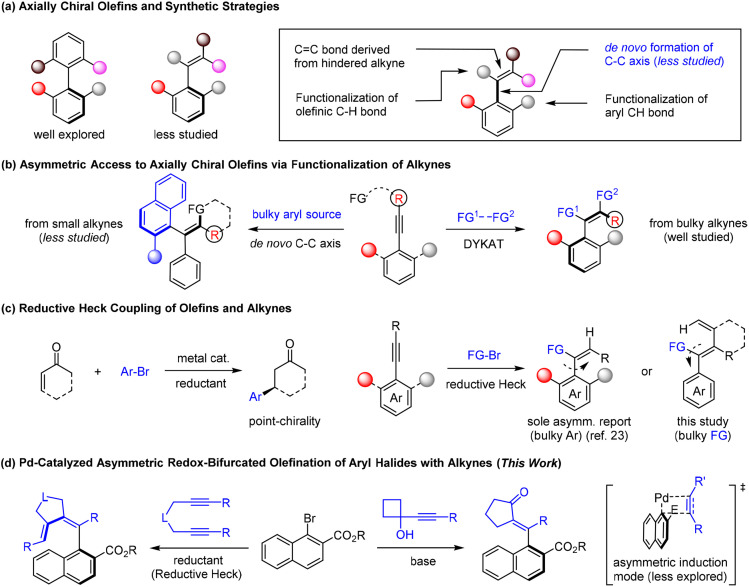
Atroposelective synthesis of axially chiral olefins.

Axially chiral platforms are ubiquitous in bioactive molecules, chiral ligands, natural products, and functional materials.^1^ Ever since the discovery of axial chirality, significant efforts have been dedicated to the exploration of axially chiral biaryls.^2^ On the other hand, axially chiral olefins represent a more challenging synthetic target, given their conformational flexibility and relatively low configurational stability, especially in acyclic setting.^3^ Three distinct synthetic strategies have been adopted (Scheme 1a), namely, functionalization of the C≡C bond of a sterically hindered alkyne that forges a C=C bond,^4,5^ functionalization of an olefinic or aryl C–H bond,^6^ and *de novo* construction of a chiral axis through metal-catalyzed cross-coupling^7–11^ (Scheme 1a). The former two strategies take advantage of the size-increasing effect of substrates bearing a preinstalled chiral axis, while the *de novo* coupling strategy is drastically more challenging by connecting two bulky fragments. With the increasing importance of axially chiral styrenes, it is of great necessity to develop novel atroposelective synthetic methods by developing new *de novo* coupling strategies.

As a pivotal functional group in organic compounds, the triple bond in alkynes undergoes numerous functionalization to deliver olefins. Consequently, atroposelective functionalization of alkynes bearing a sterically hindered aryl group becomes a well-explored under organocatalysis^4^ or metal catalysis.^5^ However, this reaction mode of dynamic kinetic asymmetric transformation (DYKAT) is limited to employment of sterically hindered but reactive alkynes (Scheme 1b), and the introduction of a sterically hindered aryl group to the starting alkynes may pose significant synthetic challenges. Compared to bulky alkynes, very limited studies utilized simple alkynes through *de novo* construction of a chiral axis (Scheme 1b).^12–16^

In the past decades, reductive coupling of electrophiles has been established as an attractive synthetic strategy owing to the availability of electrophiles.^17–20^ This strategy not only avoids the employment of sensitive organometallic species but also offers orthogonality to the classical cross-coupling. To this end, asymmetric functionalization of π-bonds has been realized by reductive Heck reactions that deliver point-chiral products (Scheme 1c).^21^ However, the reductive Heck reaction of alkynes predominantly delivered achiral olefins.^22^ In 2023, the Zhu group reported the only example of Ni-catalyzed reductive hydroarylation of sterically hindered alkynes (Scheme 1b).^23^ Compared with the powerful roles of Ni and Co catalysts in reductive couplings, Pd-catalyzed versions are less common. This is likely ascribed to the low rate of reductive elimination at the palladium center.^24^ To address the challenge of reductive Heck reactions, we devised palladium-catalyzed atroposelective hydroarylation of 1,6-diynes (Scheme 1c) through *de novo* C–C formation enabled by a bulky chiral ligand, while such 1,6-diynes were previously extensively investigated in catalytic atroposelective [2 + 2 + 2] annulation.^25^ Meanwhile, the palladium(ii) aryl intermediate can be also trapped by an alkyl species derived from strain-driven ring expansion of alkynylcyclobutanol to give axially chiral,^26^ exo-cyclic enones (Scheme 1d). By employing rationally designed alkynes, we now report Pd-catalyzed redox-diversified asymmetric olefination of aryl halides, streamlining the assembly of axially chiral styrenes with excellent regio- and enantioselectivity.

## Results and discussion

With this reaction design in mind, we initiated our studies with the optimization of the reductive olefination reaction between 1-naphthyl bromide 1a and diyne 2a using palladium acetate as a catalyst (Table 1). It was found that essentially no reaction occurred when a chiral P–P or P–N bidentate ligand was used (L1–L5), likely due to coordinative saturation (see Scheme 7). Switching to a phosphoramidite ligand led to observation of the desired axially chiral diene 3 in low yield (L6 and L7) when HCOOK was used as a reductant in MeOH. Inspired by the performance of this monodentate ligand, we moved to P-chiral dihydrobenzooxaphosphole monodentate ligand^27^L8, and both efficiency and enantioselectivity were significantly improved. Variation of the aryl group in this class of ligand returned L10 as the optimal one, where product 3 was isolated in good yield and excellent enantioselectivity, together with a small of amount of its regioisomer (rr = 9 : 1). Further optimization revealed that 50 °C is the optimal temperature (entry 2). The 15-crown-5 additive improved both the regioselectivity and the conversion (entry 7). In contrast to the effective HCOOK reductant, lower efficiency and regioselectivity was observed when HCOONa or Mn was used as a reductant (entries 8 and 9). Lowering the catalyst loading to 5 mol% resulted in a slightly lower yield of 3 (entry 10).

**Table 1:** Optimization studies on atroposelective synthesis of axially chiral dienes^a^^,^^b^

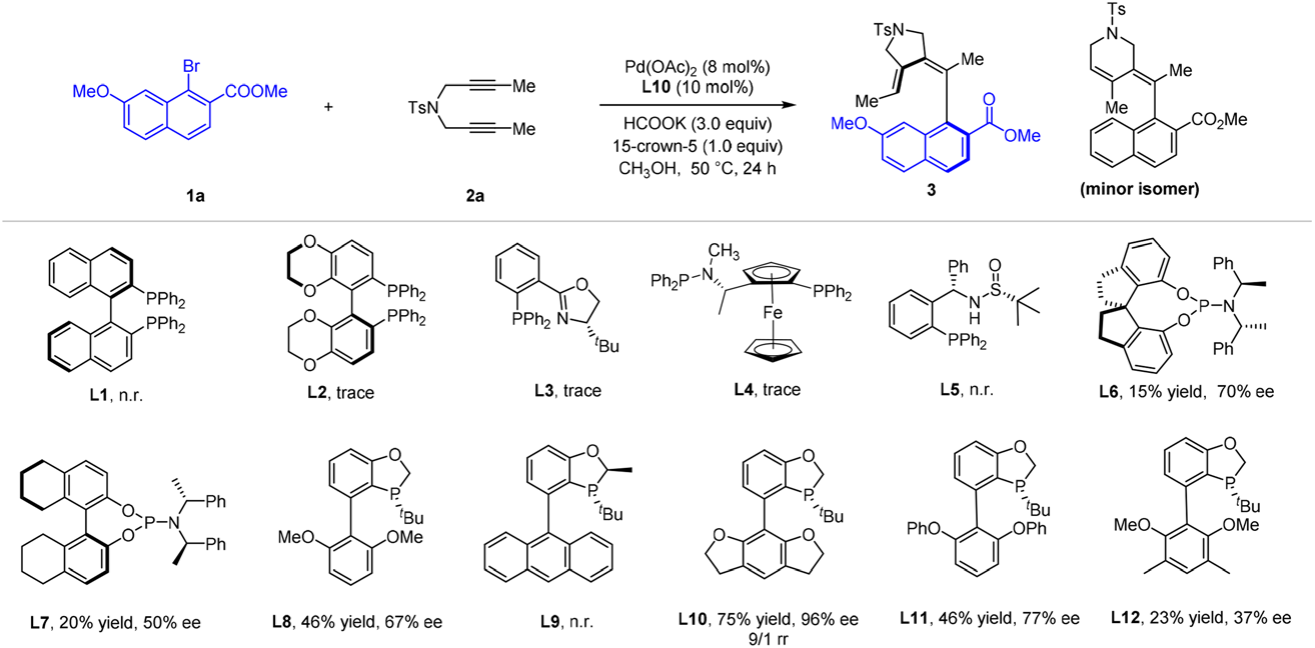				
Entry	Variations from standard conditions	Yield^b^ (%)	ee (%)	rr (%)
1	None	75	95	9/1
2	60 °C instead of 50 °C	65	96	6/1
3	Pd_2_dba_3_ as a catalyst	45	93	2/1
4	EtOH as a solvent	50	90	4/1
5	HFIP or TFE as a solvent	n.r	—	—
6	^i^PrOH as a solvent	32	85	5/1
7	No 15-crown-5 additive	63	95	4/1
8	Mn as reductant	52	91	2/1
9	HCOONa as reductant	60	92	6/1
10	5 mol% Pd(OAc)_2_	68	96	9/1

a Standard reaction conditions: 1a (0.12 mmol), 2a (0.10 mmol), Pd(OAc)_2_ (8 mol%), L10 (10 mol%) in MeOH (2 mL) at 50 °C for 24 h, the ee was determined by HPLC using a chiral stationary phase. The rr was determined by ^1^H NMR analysis of the crude reaction mixture.

b Isolated yield of the desired isomer.

**Scheme 2:**
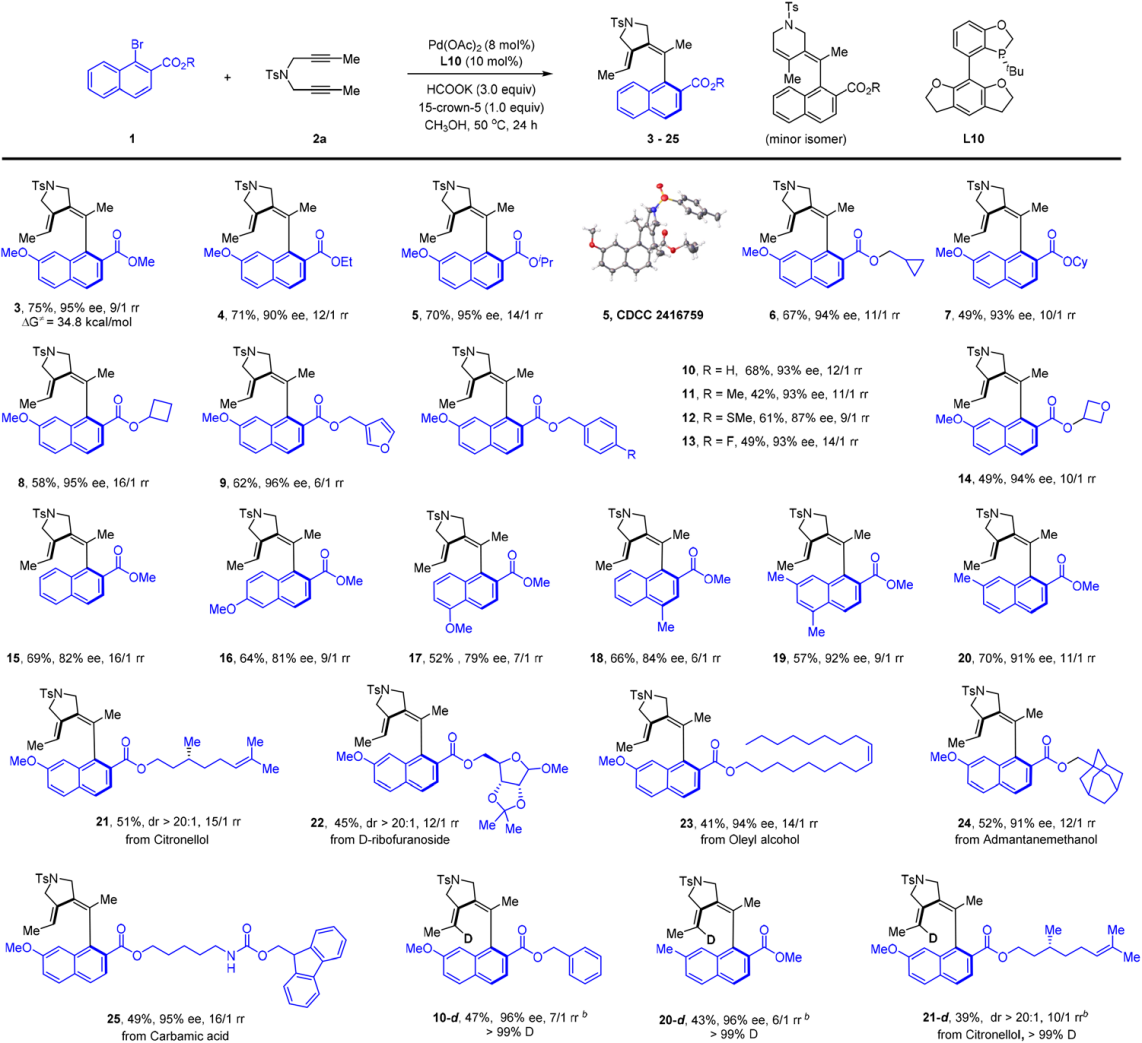
Scope of aryl bromides in reductive olefination.^*a*,*b a*^Conditions: aryl bromide 1 (0.12 mmol), 1,6-diyne 2a (0.10 mmol), Pd(OAc)_2_ (8 mol%), L10 (10 mol%), HCOOK (3.0 equiv.), 15-crown-5 (1.0 equiv.) in MeOH (2 mL), 50 °C, 24 h, isolated yield of the desired isomer. The ee was determined by HPLC using a chiral stationary phase. The ratio of rr was determined by ^1^H NMR analysis of the crude reaction mixture. ^*b*^DCOONa was used.

We next went on to explore the scope of this coupling system (Scheme 2). The scope of the 1-naphthyl bromide was examined using 1,6-dyne 2a as the coupling partner. Our studies revealed the compatibility of a series of primary and secondary alkyl esters (4–14, 87–96% ee). The absolute configuration of 5 has been established by X-ray crystallography (CCDC 2416759). As expected, a series of benzyl ester was also compatible (10–13). Extension of the ester group to those derived from a natural product (21–25) further verified the generality of this protocol, and the product was isolated in excellent enantio- or diastereoselectivity. Removal of the 7-substituent in the naphthalene ring led to lower enantioselectivity (15–18, 79–84% ee), suggesting that the steric effect of a 7-substituent facilitated the chiral induction because introduction of a 7-methyl group restored the excellent enantioselectivity (19 and 20). To our delight, the employment of DCOONa as a reductant afforded the deuterated product with >99% deuterium incorporation at the olefinic position (10-d, 20-d, 21-d). Among all the reactions, the regioselectivity varied within 6–20 : 1 rr. We also applied simple 1-naphthyl bromide as a substrate, but observed no reaction. The inactivity is caused by lack of directing and/or EWG nature of the ester group.

**Scheme 3:**
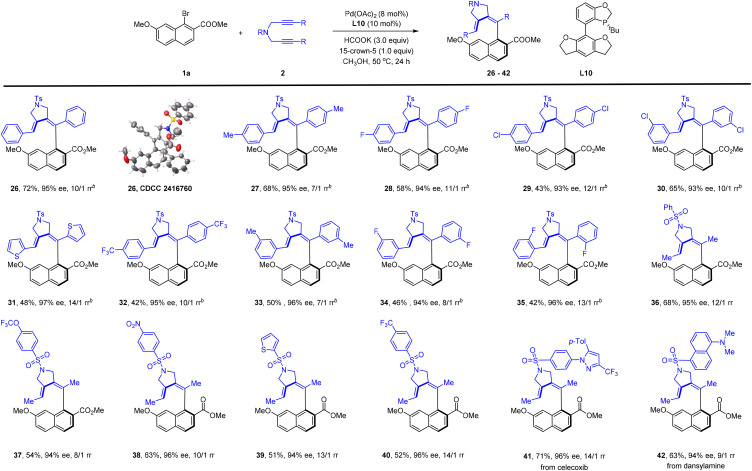
Scope of diynes in atroposelective C–C Coupling.^*a*,*b a*^Conditions: 1-naphthyl bromide 1a (0.12 mmol), 1,6-diyne 2 (0.10 mmol), Pd(OAc)_2_ (8 mol%), L10 (10 mol%), HCOOK (3.0 equiv.), and 15-crown-5 (1.0 equiv.) in MeOH (2 mL) at 50 °C for 24 h, isolated yield. The ee was determined by HPLC using a chiral stationary phase. The rr was determined by ^1^H NMR analysis of the crude reaction mixture. ^*b*^1a (0.12 mmol), 2 (0.10 mmol), (η^3^-allyl)(η^5^-Cp)Pd (8 mol%), L10 (10 mol%), HCOOK (2.0 equiv.), and 15-crown-5 (1.0 equiv.) in MeOH (2 mL) at 50 °C for 24 h.

**Scheme 4:**
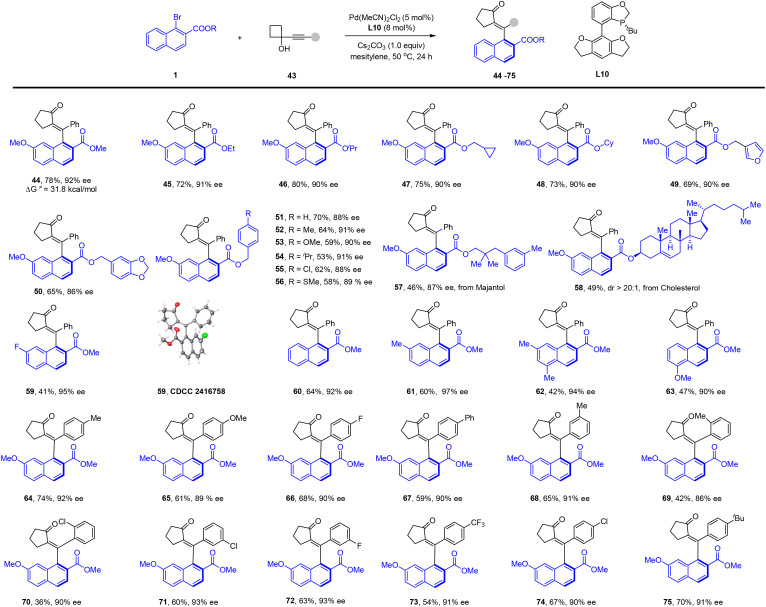
Scope of atroposelective redox-neutral olefination using 1-alkynylcyclobutanols.^*a a*^Reaction conditions: 1 (0.15 mmol), alkynylcyclobutanol 43 (0.10 mmol), Pd(MeCN)_2_Cl_2_ (5 mol%), L10 (8 mol%) and Cs_2_CO_3_ (1.0 equiv.) in mesitylene (2 mL) at 50 °C for 24 h under N_2_; isolated yield. The ee was determined by HPLC using a chiral stationary phase.

After the exploration of the scope of naphthyl bromide, we then proceeded with investigation of the scope of the 1,6-diyne (Scheme 3). The reaction went equally well for a series of diaryl-substituted diynes bearing alkyl, halogen, and EWG at different positions of the benzene ring (26–35, 93–97% ee). A thienyl-functionalized symmetric diyne was also applicable (31). The sulfonamide linker in the alkyne was then investigated, and the presence of different aryl groups in this moiety was well tolerated (36–42), including a few functionalized sulfonamide linkers (41 and 42). Extension of the linker to an oxygen atom, however, met with failure and essentially no reaction occurred. To better explore the scope of the alkyne, we extended the alkyne to 1-alkynylcyclobutanol, another class of bifunctional alkyne (Scheme 4). To our delight, the desired redox-neural difunctionalization of the alkyne proceeded efficiently using the same ligand with mesitylene as a solvent and with Cs_2_CO_3_ as a base, affording an axially chiral enone 44 (92% ee) as the only regioisomer. The scope of this coupling system was then briefly explored. As given in Scheme 4, a similar scope of the ester group has been established, and the coupled product was isolated in consistently high enantioselectivity (44–56, 86–92% ee). In line with the scope of the aryl halide in our reductive olefination, the inclusion of a natural product was also tolerated (57 and 58). The coupling of 1-naphthyl bromide with or without a 7-substituent proceeded essentially equally well (59–63, 90–97% ee), which stays contrast to the reliance of a 7-substituent in the above reductive olefination system (Scheme 2). The absolute configuration of product 59 was confirmed by X-ray crystallography (CCDC 2416758). Variations of the substituent in the benzene ring of the 1-alkynylcyclobutanol also verified the compatibility of diverse electron-donating, -withdrawing, and halogen groups (64–75, 86–93% ee).

**Scheme 5:**
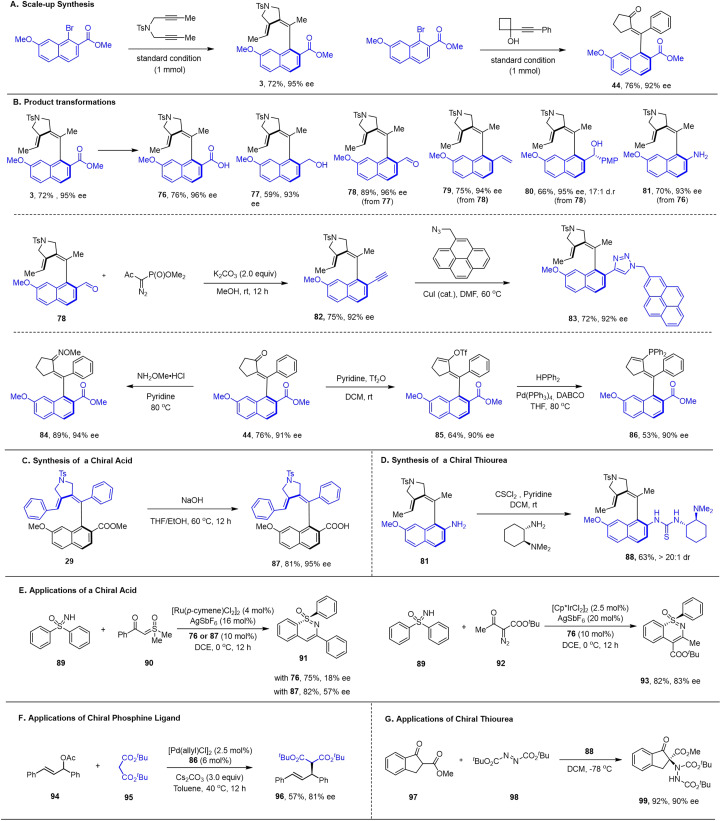
Downstream synthetic and catalytic applications (see ESI† for details).

The synthetic utility of representative products is showcased in Scheme 5. Scale-up synthesis (1 mmol scale) of both products 3 and 44 was accomplished with no compromise of the reaction efficiency or enantioselectivity (Scheme 5A). The ester group in product 3 provides a useful synthetic handle (Scheme 5B), which allows efficient transformations to other functional groups in 76–78 (acid, alcohol, and aldehyde), and the aldehyde 78 was further converted to an olefin and a secondary alcohol with excellent enantio- or diastereoselectivity (79 and 80). Meanwhile, acid was also converted to amine 81. Aldehyde 78 was also converted to an alkyne and then to a triazole (82 and 83). The ketone carbonyl group in product 44 was converted to an oxime (84) and a vinyl triflate (85), and the latter was readily engaged in Pd-catalyzed phosphination reaction to give 86 as a potentially useful axial chirality-based ligand. Saponification of 29 provided a chiral carboxylic acid 87, which functioned as a chiral additive in Ru-catalyzed asymmetric C–H activation, affording product 91 in moderate enantioselectivity. Analogously, acid 76 was demonstrated as a chiral additive in Ir(iii) catalyzed C–H activation, and the annulation product 93 was obtained in high enantioselectivity. Amine 81 can be easily converted to bifunctional thiourea 88, which has been proved to be an excellent organic catalyst for enantioselective α-amination of β-ketoester 97 with azodicarboxylate 98. The employment of ligand 86 in Pd-catalyzed allylic substitution afforded 96 in 81% ee.

**Scheme 6:**
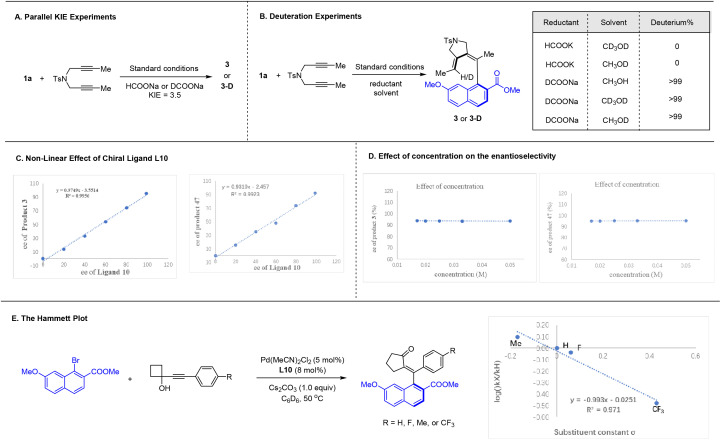
Mechanistic studies.

A series of experiments have been conducted to explore the mechanism of these two coupling systems (Scheme 6). Kinetic isotope effect of the reductive olefination reaction using a diyne has been measured from parallel reactions using HCOONa and DCOONa (Scheme 6A). The rather large value of KIE = 3.5 suggests that the C–H bond formation is likely involved in the rate-limiting step. Further control studies using deuterated solvent and/or deuterated formate salt verified that the olefinic hydrogen in the product originates exclusively from the formate (Scheme 6B). Non-linear effect studies revealed that the catalyst functions as a monomeric form with 1 : 1 ratio to the chiral ligand in both coupling systems (Scheme 6C). Furthermore, the ee of the product in each system stayed constant in all concentrations examined, suggesting that the palladium species devoid of the chiral ligand is essentially inactive (Scheme 6D). To further explore the coupling of 1-alkynylcyclobutanol, we conducted the linear correlation studies using a series of electronically different 1-alkynylcyclobutanol (Scheme 6E). The large slope of *ρ* = −0.9 suggests buildup of positive change in the transition state, and this observation may offer mechanistic details of the alkyne insertion event (*vide infra*).

**Scheme 7:**
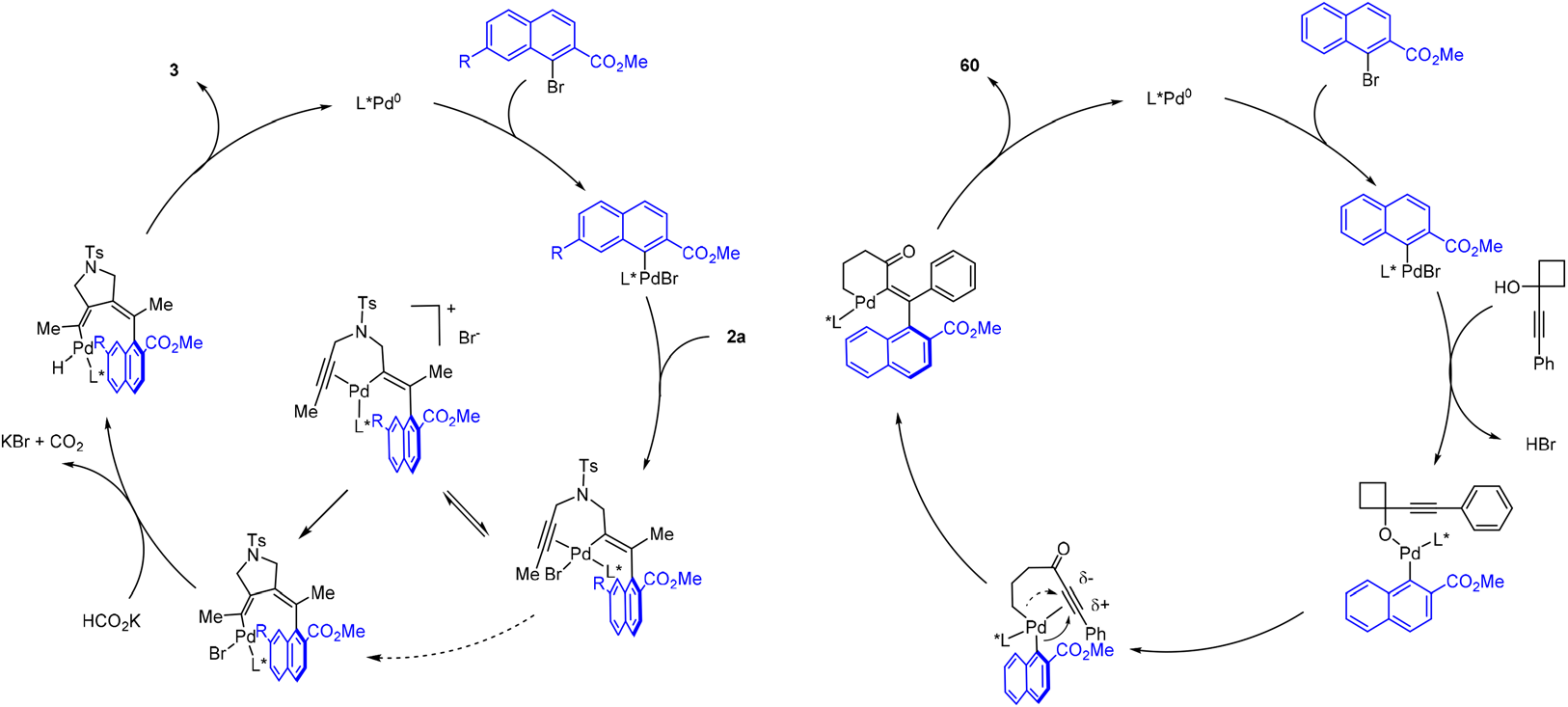
Proposed mechanism.

The mechanism of the reductive coupling of diyne 2a is proposed in Scheme 7 (left). Oxidative addition of aryl bromide gives a Pd(ii) aryl species. The resulting Pd–aryl bond is proposed to undergo selective migratory insertion into the alkyne to give an alkenyl intermediate. Subsequently, the bromide ligand likely undergoes reversible dissociation in MeOH to give coordinatively unsaturated, less hindered cationic intermediate to allow for Pd–alkenyl migratory insertion. In fact, significant inhibition was observed when TBAB was introduced to the reaction conditions, suggesting the participation of a cationic palladium intermediate (see ESI†). After the 2^nd^ migratory insertion, ligand exchange with a formate is followed by β-H elimination to give a palladium hydride. The coupled product 3 is released upon rate-limiting C–H reductive elimination. In the case of the redox-neutral coupling of an 1-alkynylcyclobutanol, the mechanism involves a common Pd(ii) aryl intermediate (Scheme 7, right). Substitution by an incoming alkoxide ligand likely triggers β-carbon elimination driven by the ring strain. The resulting Pd(ii) alkyl–aryl species preferentially undergoes migratory insertion of the aryl group based on the electronic effect of the triple bond. The attack of aryl group at the more electrophilic β-position of the alkyne is electronically favorable because of buildup of positive charge at this position. In contrast, the migratory insertion of the alkyl group to the alpha position of the alkyne is electronically disfavored. Indeed, this propensity also aligns well with our Hammett studies. After this enantio-determining migratory insertion, the palladium alkyl species then undergoes C–C reductive elimination to furnish the final product 60.

**Fig. 1:**
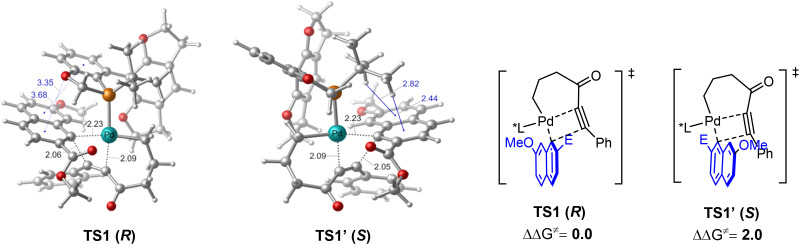
DFT studies of the key migratory insertion step. Bond distances and energies are given in Å and kcal mol^−1^, respectively.

Density functional theory (DFT) calculations were further performed to explore the coupling of 1-alkynylcyclobutanol to gain insight into the enantioselective control of this reaction (Fig. 1). Starting from a key palladium(ii) alkyl–aryl intermediate, two possible modes of the migratory insertion, namely insertion of the C–C triple bond into the Pd–C(alkyl) *versus* the Pd–C(aryl) bond, were both evaluated. In line with our experimental rationalization, the insertion into the Pd–C(aryl) bond occurs with a lower energy barrier regardless of the orientation of the naphthyl group (see the ESI† for details). The transition state TS1 was calculated to be lower in energy than TS1′ by 2.0 kcal mol^−1^, which is in accordance with the experimentally observed enantioselectivity. Scrutiny of the optimized geometries reveals that the enantioselectivity is predominantly attributed to non-covalent interactions. In TS1, the π–π and lone pair–π interactions were observed between the ligand and the aryl group, whereas only C–H–π interactions were present in TS1′, thereby making TS1 lower in energy than TS1′.

## Conclusions

In summary, we have realized palladium-catalyzed redox-diversified olefination of aryl halides with two classes of bifunctional alkynes. The coupling of 1,6-diynes occurred under reductive conditions using formate salt as the reductant, affording axially chiral dienes as the product. In the case of 1-alkynylcyclobutanol substrates, the alkyne undergoes ring expansion-driven C–C coupling to give axially chiral enones as the product. In both coupling systems, the reactions proceeded effectively with high regioselectivity, *Z*/*E* selectivity, and excellent enantioselectivity. Mechanistic studies of the reaction of 1-alkynylcyclobutanol revealed mechanistic details, and the migratory insertion of the aryl group has been established as the enantiodetermining step. Given limited exploration of axially chiral olefins and the rarity of reports on small alkynes toward construction of axially chiral olefins, this protocol may find applications in development of new atroposelective catalytic systems.

## Author contributions

X. L. proposed the research direction and guided the project. Z. L. performed the experiments. X. L., F. W., J. L. and Z. L. analyzed and discussed the experimental results and drafted the manuscript. Y. J. conducted the computational studies under the supervision of G. H., X. L. conceived and directed the project and acquired the research funding. All authors contributed to the writing of the manuscript.

## Conflicts of interest

The authors declare no competing financial interests.

## Supplementary Material

SC-OLF-D5SC04080A-s001

SC-OLF-D5SC04080A-s002

## Data Availability

Further details of the experimental procedure, ^1^H and ^13^C NMR, HPLC spectra, and X-ray crystallographic data are available in the ESI.†
