# Supplementary material for: Palladium-catalyzed redox diversified entry to axially chiral styrenes via asymmetric olefination with alkynes
Source: Chem Sci. 2025 Jul 4;16(31):14252–61. doi: 10.1039/d5sc04080a (PMC12242276; doi:10.1039/d5sc04080a)

Supplementary Information (SI) for Chemical Science.
This journal is © The Royal Society of Chemistry 2025

Supporting Information

Palladium-Catalyzed Redox-Diversified Entry to Axially Chiral Styrenes via Asymmetric Olefination of Alkynes

Ziwei Lin,[a] Yuqing Jiang,[b] Fen Wang,*[a] Genping Huang,*[b] Jessica Li,[c] and Xingwei Li*[a,d]

[a] School of Chemistry and Chemical Engineering, Shaanxi Normal University, Xi'an 710062, P. R. China.

[b] Department of Chemistry, School of Science, Tianjin University, Tianjin 300072, P. R. China.

[c] School of Arts and Sciences, Brandeis University, Waltham, MA 02453, U.S.A.

[d] Institute of Frontier Chemistry, School of Chemistry and Chemical Engineering, Shandong University, Qingdao 266237, P. R. China.

E-mail: lixw@snnu.edu.cn, gphuang@tju.edu.cn, fenwang@snnu.edu.cn

Table of Contents

1. General Information:..........S3

2. Optimization Studies..........S4

3. General Procedure and Characterization of Products..........S11

4. Synthetic Applications..........S71

5. Mechanistic Studies..........S88

6. X-Ray Crystallographic Data..........S97

7. Rotational Barriers..........S101

8. DFT Calculation..........S103

9. References..........S115

10. NMR Spectra..........S116

1. General Information:

All chemicals were obtained from commercial sources and were used as received unless otherwise noted. All the reactions were carried out in an argon-filled glove box. The ^{1}H NMR spectra were recorded on 600 MHz NMR spectrometer. The ^{13}C NMR spectra were recorded at 150 MHz. The ^{19}F NMR spectra were recorded at 376 MHz. The ^{31}P NMR spectra were recorded at 243 MHz. Chemical shifts were expressed in parts per million (*δ*) downfield from the internal standard tetramethylsilane (TMS), and were reported as s (singlet), d (doublet), t (triplet), dd (doublets of doublet) and m (multiplet). The residual solvent signals were used as references and the chemical shifts were converted to the TMS scale ($CDCl_3$: *δ* H = 7.26 ppm, *δ* C = 77.16 ppm; DMSO-d_6: *δ* H = 2.50 ppm). The coupling constants *J* were given in Hz. High resolution mass spectra (HRMS) were obtained via ESI mode by using a MicroTOF mass spectrometer. Column chromatography was performed on silica gel 200-300 mesh. The enantiomeric excess (ee) of the products were determined by high-performance liquid chromatography (HPLC) with a chiral stationary phase in comparison with the authentic racemate sample with ^{n}hexane and ^{i}PrOH as solvents. All the chiral stationary phases including Chiralcel IA, IB, IC, IG, ID, OZ, IE, OD-H, AD-H were purchased from Daicel Chirsal Technologies. Optical rotations were reported as follows: $[\alpha]_D^T$ = (c: mg/ mL, in $CDCl_3$). Aryl bromides,[1-3] 1,6-diynes,[4-7] and 1-alkynylcyclobutanols[8-9] were prepared according to published procedures.

2. Optimization Studies

Table S1. Initial Screening for Synthesis of Racemic Product 3[a]

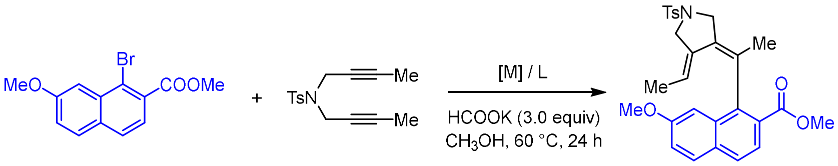

1a 2a 3

Entry	Catalyst	Ligand	yield (%)
1	$NiBr_2$.DME (10.0 mol%)	dtbppy	n.r.
2	$Ni(cod)_2$ (10.0 mol%)	dtbppy	n.r.
3	$CoBr_2$ (10.0 mol%)	Bipy	n.r.
4	Pd_2dba_3 (5.0 mol%)	PPh_3	38
5	$Pd(acac)_2$ (5.0 mol%)	PPh_3	35
6	$Pd(OAc)_2$ (5.0 mol%)	PPh_3	46
7	$Pd(OAc)_2$ (5.0 mol%)	BINAP	n.r.
8	$Pd(OAc)_2$ (6.0 mol%)	xphos	21
9	$Pd(OAc)_2$ (6.0 mol%)	dppp	30

[a]Reaction conditions: **1a** (0.12 mmol), **2a** (0.10 mmol), metal catalyst, ligand (10.0 mol%) and HCOOK (0.30 mmol) in CH_3OH (0.05 M) at 60 °C for 24 h under N_2; isolated yield.

Table S2. Screening of Chiral Ligands[a]

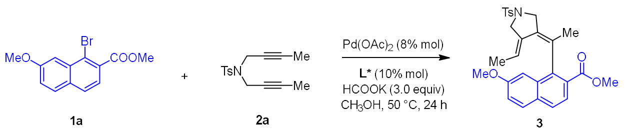

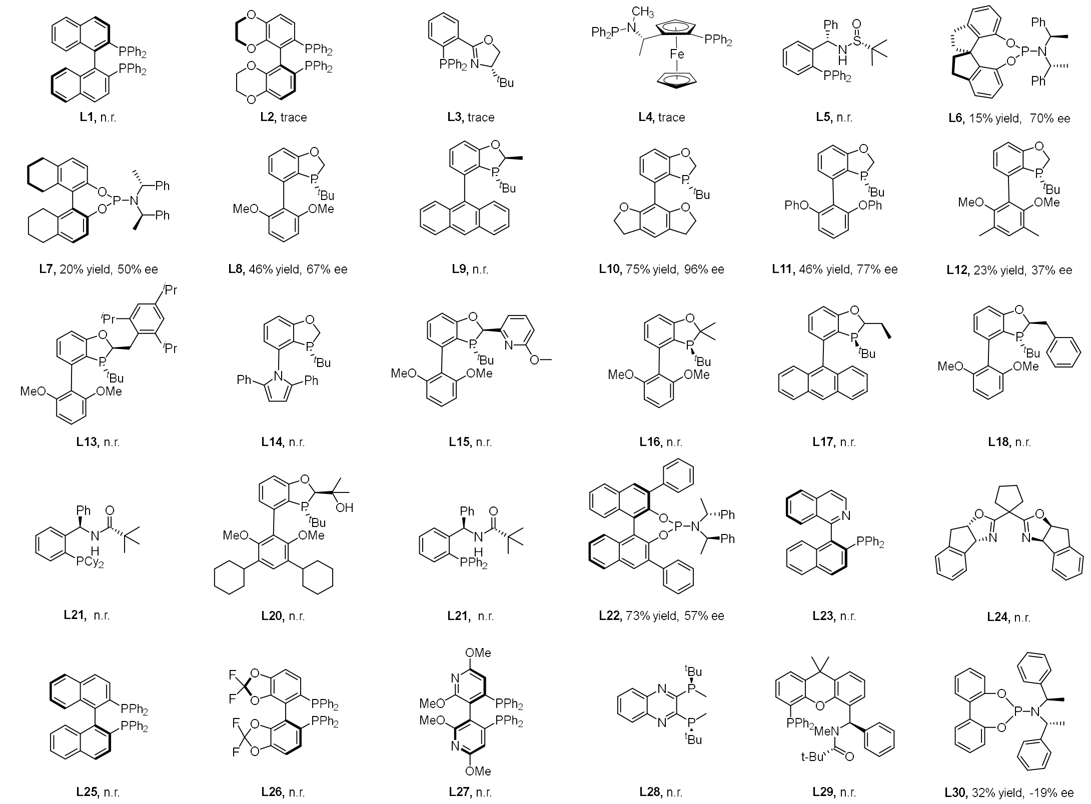

[a]Reaction conditions: **1a** (0.12 mmol), **2a** (0.10 mmol), Pd(OAc)$_2$ (8.0 mol%), chiral ligand **L*** (10.0 mol%), HCOOK (0.30 mmol) in CH_3OH (0.05 M) at 50 °C for 24 h under N_2; isolated yield; The ee was determined by HPLC using a chiral stationary phase.

Table S3. Screening of the Solvent[a]

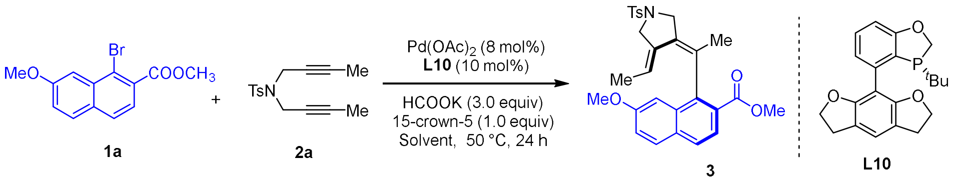

Entry	Solvent	Yield (%)	Ee (%)
1	^{t}BuOH	n.r.	-
2	Toluene + 5 equiv H_2O	n.r.	-
3	Toluene + 5 equiv CH_3OH	45	85
4	EA + 5 equiv CH_3OH	29	85
5	DME + 5 equiv CH_3OH	27	83
6	DCE + 5 equiv CH_3OH	37	90

7	DMA + 5 equiv CH_3OH	n.r.	-
8	CH_3CN + 5 equiv CH_3OH	n.r.	-
9	CH_3CN + 5 equiv CH_3OH	n.r.	-
10	PhCl + 5 equiv CH_3OH	n.r.	-
11	^{i}PrOH + 5 equiv CH_3OH	52	93
12	Toluene + 5 equiv H_2O	n.r.	-
13	MeOH	75	95

[a]Reaction conditions: $Pd(OAc)_2$ (8.0 mol%), **L10** (10.0 mol%), 15-crown-5 (1.0 mmol), **1a** (0.12 mmol), HCOOK (0.30 mmol) and **2a** (0.10 mmol) in a solvent (0.05 M) at 50 °C for 24 h under N_2; isolated yield; The ee was determined by HPLC using a chiral stationary phase.

Table S4. Screening of the Reductant[a]

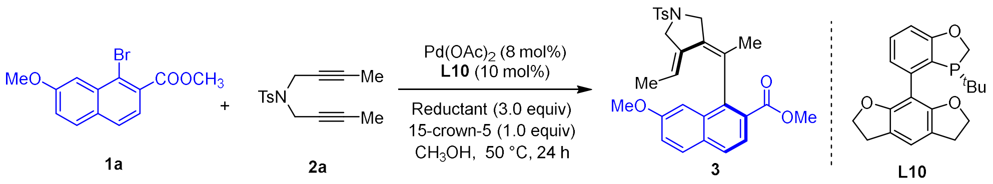

Entry	Reductant	Yield (%)	Ee (%)
1	*t*-BuONa	62	77
2	$NaBH_4$	45	89
3	HCOOH	50	85
4	$BH_3{\cdot}NH_3$	n.r.	-
5	$(MeO)_3SiH$	n.r.	-
6	Zn	28	81
7	Mg	n.r.	-
8	$HCOONH_4$	n.r.	-
9	HCOOCs	n.r.	-
10	TDAE	45	75
11	HCOOK	75	95

[a]Reaction conditions: $Pd(OAc)_2$ (8.0 mol%), **L10** (10.0 mol%), 15-crown-5 (1.0 mmol), **1a** (0.12 mmol), Reductant (0.30 mmol) and **2a** (0.10 mmol), and reductant in MeOH (0.05 M) at 50 °C for 24 h under N_2; isolated yield; the ee was determined by HPLC using a chiral stationary phase.

Table S5. Screening of the Additive[a]

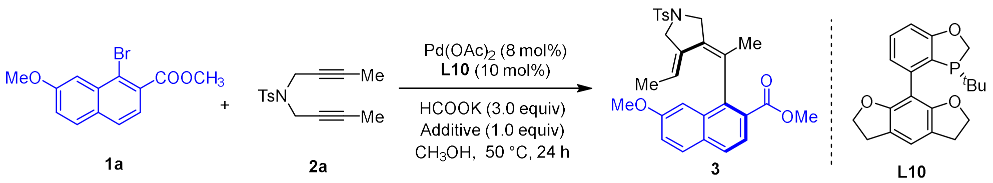

Entry	Additive	Yield (%)	Ee (%)
1	18-crown-6	66	93
2	TBAF	59	93
3	TBAB	41	91
4	TBAI	60	84
5	LiBr	63	93
6	15-crown-5	75	95

[a]Reaction conditions: $Pd(OAc)_2$ (8.0 mol%), **L10** (10.0 mol%), additive (1.0 mmol), **1a** (0.12 mmol), HCOOK (0.30 mmol) and **2a** (0.10 mmol) in MeOH (0.05 M) at 50 °C for 24 h under N_2; isolated yield; The ee was determined by HPLC using a chiral stationary phase.

Table S6. Screening of the catalyst[a]

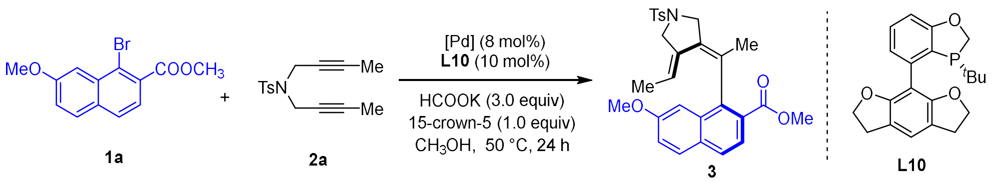

Entry	Catalyst	Yield (%)	Ee (%)
1	$Pd(MeCN)_2Cl_2$	n.r.	-
2	$PdBr_2$	n.r.	-
3	$Pd(NO_3)_2{\cdot}2H_2O$	48	91
4	$Pd(TFA)_2$	n.r.	-
5	$Pd(OH)_2$	n.r.	-
6	$Pd(hfac)_2$	28	83
7	$PdCl_2$	n.r.	-
8	(η^3-allyl)(η^5-Cp)Pd	51	91
9	$Pd(cod)Br_2$	35	81
10	$Pd(OAc)_2$	75	95

[a]Reaction conditions: catalyst (8.0 mol%), **L10** (10.0 mol%), 15-crown-5 (1.0 mmol), **1a** (0.12 mmol), HCOOK (0.30 mmol) and **2a** (0.10 mmol) in MeOH (0.05 M) at 50 °C for 24 h under N_2; isolated yield; The ee was determined by HPLC using a chiral stationary phase.

Table S7. Screening of the catalyst using a diphenyl-diyne[a]

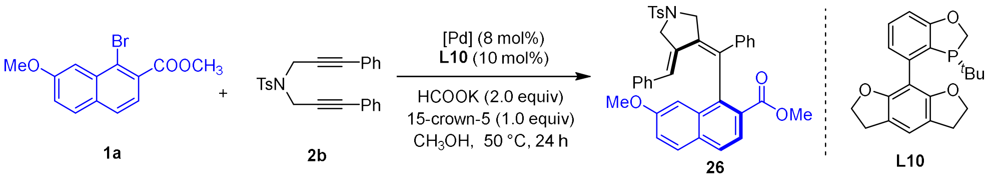

Entry	Catalyst	Yield (%)	Ee (%)
1	$Pd(OAc)_2$	70	91

2	(η^3-allyl)(η^5-Cp)Pd	72	95
3	$PdBr_2$	n.r.	-
4	$PdCl_2$	n.r.	-
5	$Pd(acac)_2$	63	86
6	$Pd(TFA)_2$	46	89
7	$Pd(OH)_2$	n.r.	-

[a]Reaction conditions: Pd catalyst (8.0 mol%), **L10** (10.0 mol%), 15-crown-5 (1.0 mmol), **1a** (0.12 mmol), HCOOK (0.30 mmol) and **2b** (0.10 mmol) in MeOH (0.05 M) at 50 °C for 24 h under N_2; isolated yield; The ee was determined by HPLC using a chiral stationary phase.

Table S8. Screening of chiral ligand for the coupling of 1-Alkynylcyclobutanol[a]

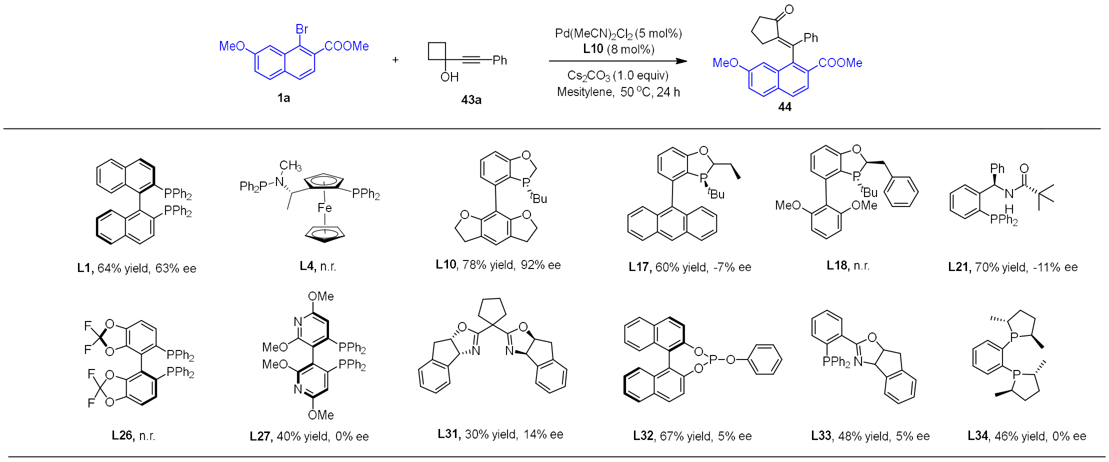

[a]Reaction conditions: $Pd(MeCN)_2Cl_2$ (5.0 mol%), ligand (8.0 mol%), **1a** (0.15 mmol), Cs_2CO_3 (0.10 mmol) and **43a** (0.10 mmol) in mesitylene (0.05 M) at 50 °C for 24 h under N_2; isolated yield; The ee was determined by HPLC using a chiral stationary phase.

Table S9. Screening of catalyst for 1-alkynylcyclobutanols[a]

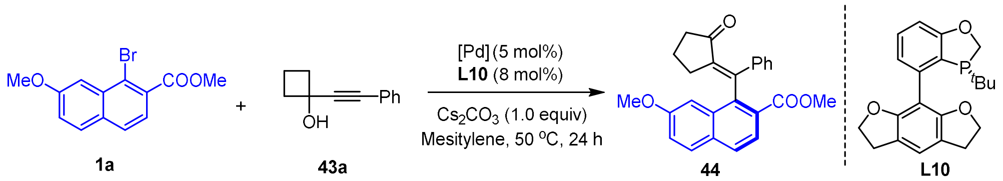

Entry	Catalyst	Yield (%)	Ee (%)
1	$Pd(acac)_2$	trace	-
2	$Pd(TFA)_2$	n.r.	-
3	Pd_2dba_3	48	85
4	$Pd(OAc)_2$	70	82
5	$Pd(OH)_2$	n.r.	-
6	$Pd(PhMeCNCl)_2$	48	76
7	(η^3-allyl)(η^5-Cp)Pd	n.r.	-

8	Pd(cod)Br_2	n.r.	-
9	Pd$(MeCN)_2Cl_2$	78	92

[a]Reaction conditions: catalyst (5.0 mol%), **L10** (8.0 mol%), **1a** (0.15 mmol), Cs_2CO_3 (0.10 mmol) and **43a** (0.10 mmol) in mesitylene (0.05 M) at 50 °C for 24 h under N_2; isolated yield; The ee was determined by HPLC using a chiral stationary phase.

Table S10. Screening of Solvent for 1-Alkynylcyclobutanol[a]

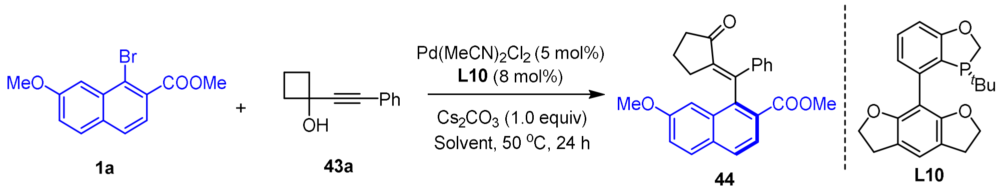

Entry	Solvent	Yield (%)	Ee (%)
1	THF	56	17
2	PhOMe	43	77
3	PhF	48	85
4	1,4-dioxane	53	83
5	CH_3OH	n.r.	-
6	CH_3CN	n.r.	-
7	$PhCF_3$	61	77
8	DCM	n.r.	-
9	MTBE	n.r.	-
10	Mesitylene	78	92

[a]Reaction conditions: Pd$(MeCN)_2Cl_2$ (5.0 mol%), **L10** (8.0 mol%), **1a** (0.15 mmol), Cs_2CO_3 (0.10 mmol) and **43a** (0.10 mmol) in a solvent (0.05 M) at 50 °C for 24 h under N_2; isolated yield; The ee was determined by HPLC using a chiral stationary phase.

Table S11. Screening of the Base for 1-Alkynylcyclobutanol[a]

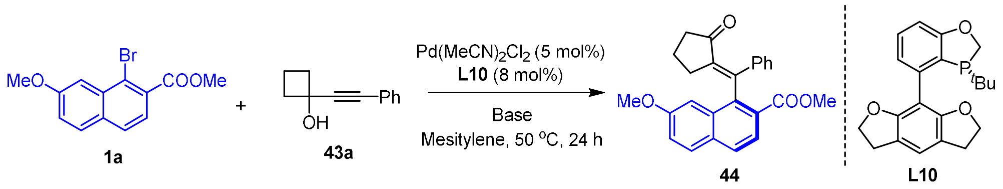

Entry	Base	Yield (%)	Ee (%)
1	K_2CO_3	n.r.	-
2	*t*-BuONa	65	83
3	$KHCO_3$	n.r.	-
4	LiOH	48	73
5	NaOMe	42	75

6	K_3PO_4	28	83
7	Et_3N	n.r.	-
8	NaOAc	33	91
9	Cs_2CO_3	78	92

[a]Reaction conditions: $Pd(MeCN)_2Cl_2$ (5.0 mol%), **L10** (8.0 mol%), **1a** (0.15 mmol), base (0.10 mmol), and **43a** (0.10 mmol) in mesitylene (0.05 M) at 50 °C for 24 h under N_2; isolated yield; The ee was determined by HPLC using a chiral stationary phase.

3. General Procedure and Characterization of Products

3.1. General procedure for the synthesis of 3-25 and 36-42

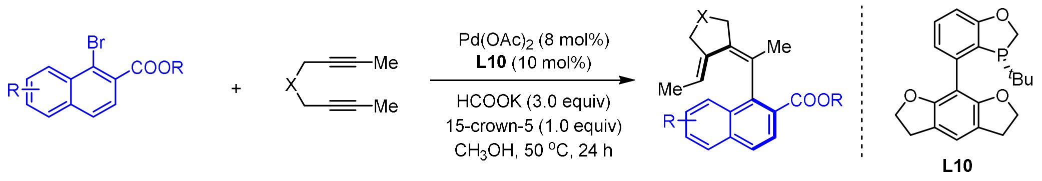

To a sealable tube (8 mL) were charged with aryl bromides **1** (0.12 mmol), 1,6-diyne **2** (0.10 mmol), $Pd(OAc)_2$ (8 mol%), **L10** (10 mol%), HCOOK (0.30 mmol), 15-Crown-5 (0.10 mmol), and anhydrous MeOH (2 mL) under N_2. The resulting mixture was stirred for 24 h at 50 °C. After that, the reaction mixture was concentrated under vacuum. The residue was purified by flash chromatography on silica gel (petroleum ether/EtOAc = 8/1) to afford the desired product.

3.2 General procedure for the synthesis of 26-35

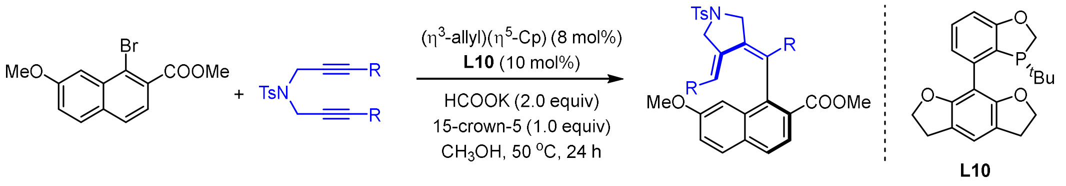

To a sealable tube (8 mL) were charged with aryl bromide **1a** (0.12 mmol), 1,6-diyne **2** (0.10 mmol), (η^3-allyl)(η^5-Cp)Pd (8 mol%), **L10** (10 mol%), HCOOK (0.20 mmol), 15-Crown-5 (0.10 mmol), and anhydrous MeOH (2.0 mL) under N_2. The resulting mixture was stirred for 24 h at 50 °C. After that, the reaction mixture was concentrated under vacuum. The residue was purified by flash chromatography on silica gel (petroleum ether/EtOAc = 10/1 to 5/1) to afford the desired product.

3.3 General procedure for the synthesis of 10-*d*, 20-*d*, 21-*d*.

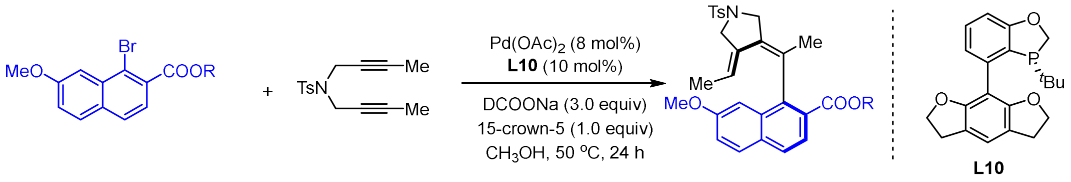

To a sealable tube (8 mL) were charged with aryl bromide **1** (0.12 mmol), 1,6-diyne **2a** (0.10 mmol), $Pd(OAc)_2$ (8 mol%), **L10** (10 mol%), DCOONa (0.30 mmol), 15-crown-5 (0.10 mmol), and anhydrous MeOH (2.0 mL) under N_2. The resulting mixture was

stirred for 24 h at 50 °C. After that, the reaction mixture was concentrated under vacuum. The residue was purified by flash chromatography on silica gel (petroleum ether/EtOAc = 10/1 to 5/1) to afford the desired product.

3.4 General procedure for the synthesis of 44-75

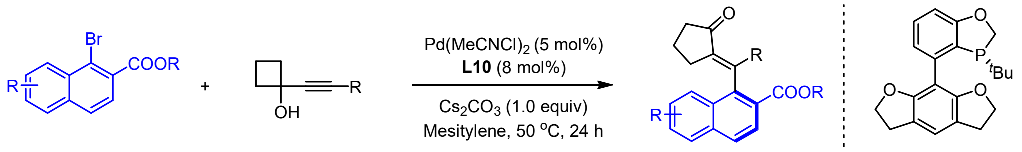

L10

To a sealable tube (8 mL) were charged with aryl bromide **1** (0.15 mmol), 1-alkynylcyclobutanol **43** (0.10 mmol), $Pd(MeCN)_2Cl_2$ (5 mol%), **L10** (8 mol%), Cs_2CO_3 (0.10 mmol), and anhydrous mesitylene (2.0 mL) under N_2. The resulting mixture was stirred for 24 h at 50 °C. After that, the residue was purified by flash chromatography on silica gel (petroleum ether/EtOAc = 8/1) to afford the desired product.

3.5 NMR and HPLC Data

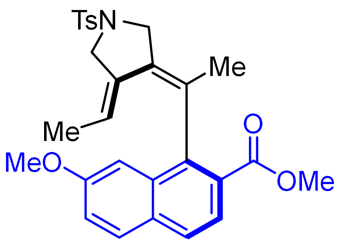

(*R*)-methyl 1-((*E*)-1-((*Z*)-4-ethylidene-1 tosylpyrrolidin-3-ylidene)ethyl)-7-methoxy-2-naphthoate (3).

The title compound was isolated as a yellow solid (eluent: petroleum ether/ethyl acetate = 4/1, 36.8 mg, 75%). **^{1}H NMR (600 MHz, Chloroform-d) *δ*** 7.82 – 7.78 (m, 3H), 7.77 – 7.72 (m, 2H), 7.38 (d, *J* = 8.3 Hz, 2H), 7.23 (dd, *J* = 8.9, 2.6 Hz, 1H), 7.10 – 7.07 (m, 1H), 4.39 (d, *J* = 13.3 Hz, 1H), 4.20 (q, *J* = 7.3 Hz, 1H), 4.11 (d, *J* = 13.0 Hz, 1H), 3.96 (d, *J* = 12.8 Hz, 1H), 3.80 (s, 3H), 3.77 – 3.74 (m, 4H), 2.47 (s, 3H), 2.05 (s, 3H), 1.17 (d, *J* = 7.2 Hz, 3H).**^{13}C NMR (150 MHz, Chloroform-d) *δ*** 167.6, 158.6, 143.8, 142.0, 133.0, 132.9, 131.3, 131.1, 129.9, 129.8, 129.5, 128.3, 128.1, 127.2, 126.0, 124.2, 120.5, 120.4, 104.7, 55.4, 52.9, 52.1, 51.5, 23.2, 21.7, 15.5. **HRMS (ESI):** calcd. for $C_{28}H_{29}NNaO_5S^+$ $[M+Na]^+$: 514.1659; found: 514.1660; **$[\alpha]_D^{20}$** = +110 (c = 0.1, $CHCl_3$).

HPLC analysis: IC column (hexane:2-propanol = 80:20, v = 1.0 mL/min, 40 °C, 254 nm); tr (major) = 22.899 min, tr (minor) = 25.061 min, 95% ee.

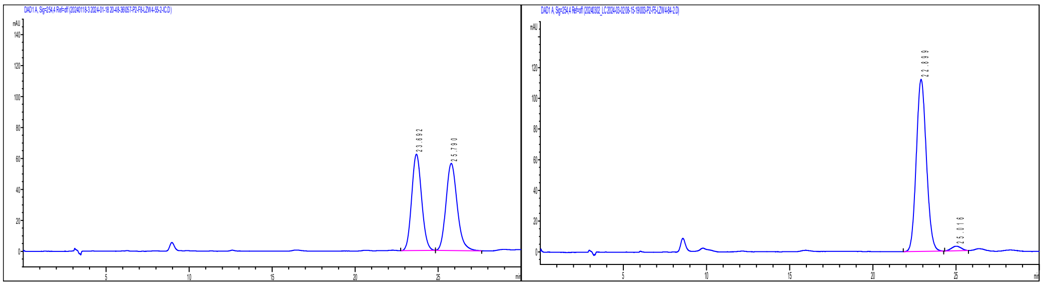

No.	Time	Area	Area (%)	No.	Time	Area	Area (%)
1	23.692	2596.5	49.565	1	22.899	4728.3	97.495
2	25.79	2642.1	50.435	2	25.016	121.5	2.505

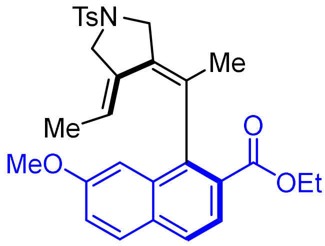

(*R*)-ethyl 1-((*E*)-1-((*Z*)-4-ethylidene-1-tosylpyrrolidin-3-ylidene)ethyl)-7-methoxy-2-naphthoate (4).

The title compound was isolated as a yellow solid (eluent: petroleum ether/ethyl acetate = 4/1, 35.9 mg, 71%). **^{1}H NMR (600 MHz, Chloroform-d) *δ*** 7.81 – 7.78 (m, 3H), 7.77 – 7.75 (m, 1H), 7.74 – 7.72 (m, 1H), 7.38 (d, *J* = 8.2, 2H), 7.23 (dd, *J* = 8.9, 2.5, 1H), 7.07-7.06 (m, 1H), 4.37 (d, *J* = 13.2, 1H), 4.25 – 4.17 (m, 3H), 4.09 (d, *J* = 13.4, 1H), 3.96 (d, *J* = 13.3, 1H), 3.80 (s, 3H), 3.76 (d, *J* = 13.8, 1H), 2.47 (s, 3H), 2.05 (s, 3H), 1.21 (t, *J* = 7.2, 3H), 1.18 (d, *J* = 7.0, 3H). **^{13}C NMR (150 MHz, Chloroform-d) *δ*** 167.5, 158.6, 143.8, 141.4, 132.89, 132.87, 131.3, 131.0, 129.9, 129.8, 129.5, 128.4, 127.7, 127.2, 126.5, 124.3, 120.5, 120.4, 104.7, 61.0, 55.4, 52.9, 51.6, 23.3, 21.7, 15.5, 14.3. **HRMS (ESI):** calcd. for $C_{29}H_{31}NNaO_5S^+$[M+Na]$^+$: 528.1815; found: 528.1817; **$[\alpha]_D^{20}$** = +204 (c = 0.1, $CHCl_3$).

HPLC analysis: IG column (hexane:2-propanol = 90:10, v = 1.0 mL/min, 40 ^{o}C, 254 nm); tr (minor) = 23.747 min, tr (major) = 26.137 min, 90% ee.

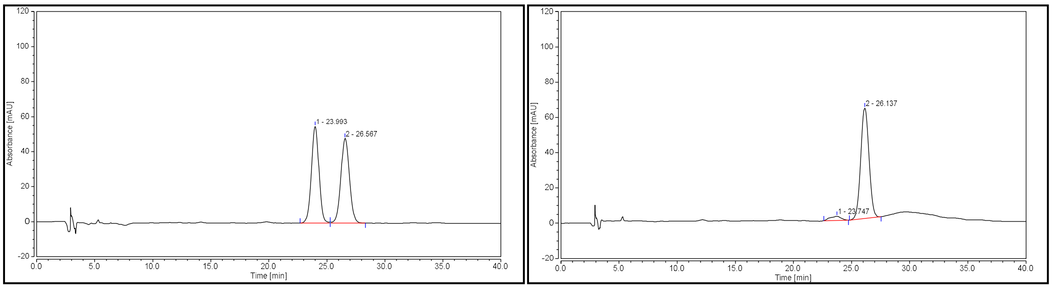

No.	Time	Area	Area (%)	No.	Time	Area	Area (%)
1	23.993	42.095	50.16	1	23.747	2.576	4.76
2	26.567	41.825	49.84	2	26.137	51.565	95.24

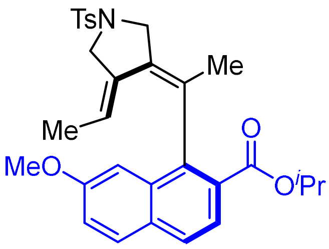

(*R*)-isopropyl 1-((*E*)-1-((*Z*)-4-ethylidene-1-tosylpyrrolidin-3-ylidene)ethyl)-7-methoxy-2-naphthoate (5)

The title compound was isolated as a white solid (eluent: petroleum ether/ethyl acetate = 4/1, 36.4 mg, 70%). **^{1}H NMR (600 MHz, Chloroform-d) *δ*** 7.82 – 7.78 (m, 2H), 7.78 – 7.75 (m, 2H), 7.74 – 7.70 (m, 2H), 7.38 (d, *J* = 8.2, 2H), 7.22 (dd, *J* = 8.9, 2.5, 1H), 7.06-7.05 (m, 1H), 5.15 – 5.05 (m, 1H), 4.35 (d, *J* = 13.2, 1H), 4.24 (q, *J* = 7.2, 1H), 4.09 (d, *J* = 13.1, 1H), 3.96 (d, *J* = 12.8, 1H), 3.81 (s, 3H), 3.76 (d, *J* = 13.0, 1H), 2.46 (s, 3H), 2.05 (s, 3H), 1.23 – 1.17 (m, 6H), 1.12 (d, *J* = 6.2, 3H).**^{13}C NMR (150 MHz, Chloroform-d) *δ*** 167.2, 158.6, 143.8, 140.8, 132.8, 131.3, 131.0, 129.89, 129.87, 129.4, 128.4, 128.1, 127.2, 127.1, 126.6, 124.4, 120.6, 120.2, 104.7, 68.3, 55.4, 52.9, 51.6, 23.4, 21.91, 21.86, 21.7, 15.5. **HRMS (ESI):** calcd. for $C_{30}H_{33}NNaO_5S^+$ $[M+Na]^+$: 542.1972; found: 542.1979; **$[\alpha]_D^{20}$** = +102 (c = 0.1, $CHCl_3$).

HPLC analysis: IG column (hexane:2-propanol = 95:5, v = 1.0 mL/min, 40 °C, 254 nm); tr (minor) = 46.000 min, tr (major) = 50.097 min, 96% ee.

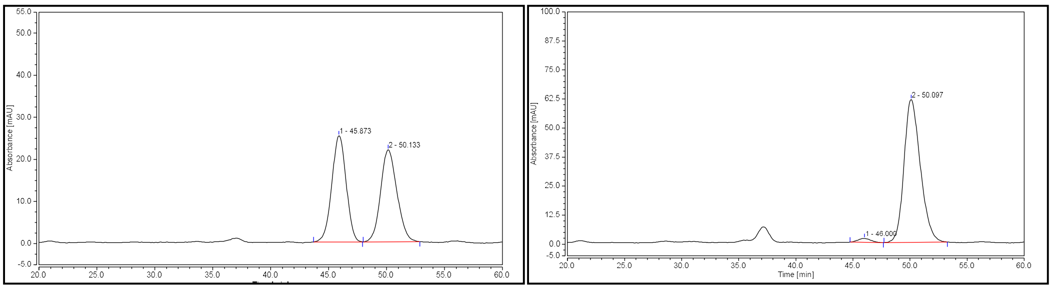

No.	Time	Area	Area (%)	No.	Time	Area	Area (%)
1	45.873	36.351	50.36	1	46.000	2.124	2.03
2	50.133	35.838	49.64	2	50.097	102.463	97.97

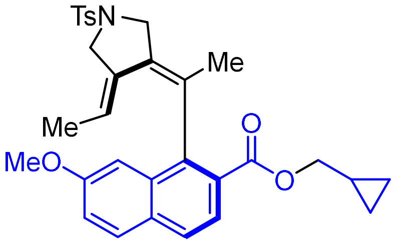

(*R*)-cyclopropylmethyl 1-((*E*)-1-((*Z*)-4-ethylidene-1-tosylpyrrolidin-3-ylidene)ethyl)-7-methoxy-2-naphthoate (6)

The title compound was isolated as a yellow solid (eluent: petroleum ether/ethyl acetate = 4/1, 35.6 mg, 67%). **^{1}H NMR (600 MHz, Chloroform-d) *δ*** 7.86 – 7.72 (m, 5H), 7.42 – 7.37 (m, 2H), 7.28 – 7.23 (m, 1H), 7.10 (s, 1H), 4.39 (d, *J* = 13.1, 1H), 4.27 – 4.22 (m, 1H), 4.12 (d, *J* =

13.2, 1H), 4.06 – 3.96 (m, 3H), 3.85 – 3.73 (m, 4H), 2.49 (s, 3H), 2.10 (s, 3H), 1.21 (d, *J* = 7.3, 3H), 1.10 – 1.03 (m, 1H), 0.55 – 0.45 (m, 2H), 0.30 – 0.24 (m, 2H). **^{13}C NMR (150 MHz, Chloroform-d) *δ*** 167.6, 158.5, 143.7, 141.2, 132.8, 131.2, 130.9, 129.75, 129.72, 129.34, 129.32, 128.3, 127.9, 127.1, 126.5, 124.3, 120.4, 120.2, 104.6, 69.8, 55.3, 52.8, 51.5, 23.2, 21.6, 15.4, 9.8, 3.34, 3.28. **HRMS (ESI):** calcd. for $C_{31}H_{33}NNaO_5S^+$[M+Na]$^+$: 554.1972; found: 554.1991; **$[\alpha]_D^{20}$** = +220 (c = 0.1, $CHCl_3$).

HPLC analysis: IC column (hexane:2-propanol = 90:10, v = 1.0 mL/min, 40 °C, 227 nm); tr (minor) = 37.653 min, tr (major) = 40.343 min, 94% ee.

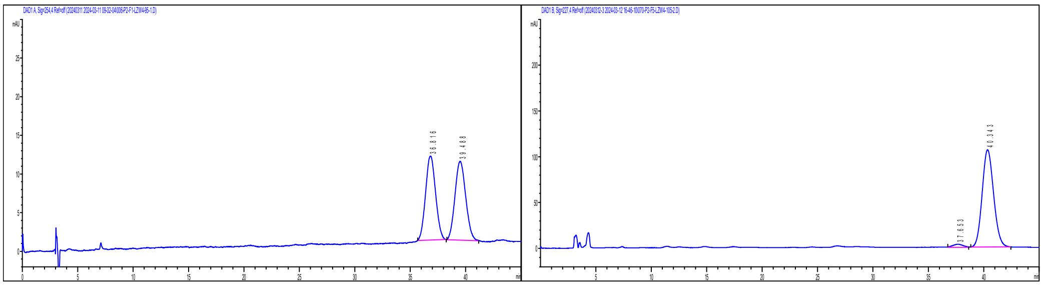

No.	Time	Area	Area (%)	No.	Time	Area	Area (%)
1	36.816	683.1	49.892	1	37.653	217.3	2.78
2	39.488	686.1	50.108	2	40.343	7599.8	97.22

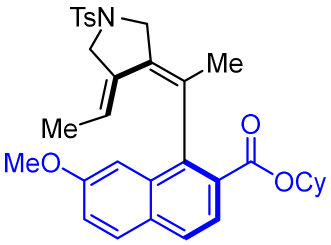

(*R*)-cyclohexyl 1-((*E*)-1-((*Z*)-4-ethylidene-1-tosylpyrrolidin-3-ylidene)ethyl)-7-methoxy-2-naphthoate (7)

The title compound was isolated as a white solid (eluent: petroleum ether/ethyl acetate = 3/1, 27.4 mg, 49%). **^{1}H NMR (600 MHz, Chloroform-d) *δ*** 7.82 – 7.70 (m, 5H), 7.38 (d, *J* = 8.2, 2H), 7.22 (dd, *J* = 8.9, 2.6, 1H), 7.07-7.06 (m, 1H), 4.90 – 4.82 (m, 1H), 4.38 (d, *J* = 13.2, 1H) 4.26 (q, *J* = 7.2, 1H), 4.07 (d, *J* = 11.8, 1H), 3.97 (d, *J* = 13.2, 1H), 3.80 (s, 3H), 3.74 (d, *J* = 13.2, 1H), 2.46 (s, 3H), 2.05 (s, 3H), 1.89 – 1.78 (m, 2H), 1.74 – 1.63 (m, 2H), 1.59 – 1.54 (m, 1H), 1.42 – 1.24 (m, 4H), 1.22 – 1.14 (m, 4H). **^{13}C NMR (150 MHz, Chloroform-d) *δ*** 167.2, 158.6, 143.8, 140.8, 132.9, 132.8, 131.3, 131.0, 129.9, 129.8, 129.4, 128.4, 128.0, 127.2, 127.1, 124.4, 120.5, 120.3, 104.6, 73.5, 55.4, 52.8, 51.5, 31.9, 25.4, 24.0, 23.4, 21.7, 15.5. **HRMS (ESI):** calcd. for $C_{33}H_{37}NNaO_5S^+$ [M+Na]$^+$: 582.2285; found: 582.2290; **$[\alpha]_D^{20}$** = +50 (c = 0.1, $CHCl_3$).

HPLC analysis: IC column (hexane:2-propanol = 95:5, v = 1.0 mL/min, 40 °C, 254 nm);

tr (minor) = 59.73 min, tr (major) = 63.489 min, 93% ee.

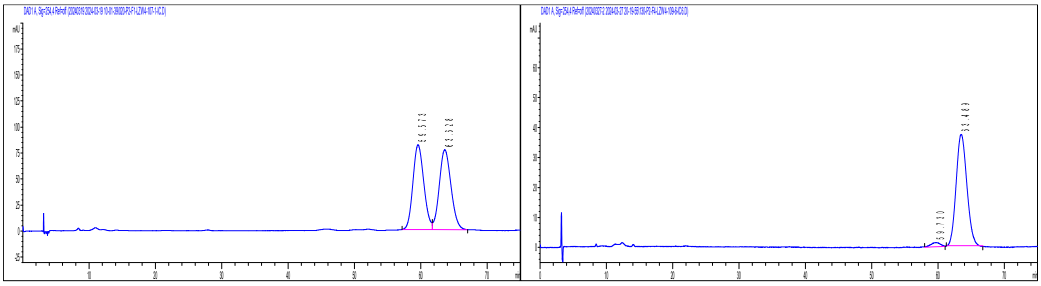

No.	Time	Area	Area (%)	No.	Time	Area	Area (%)
1	59.573	9319.8	49.735	1	59.73	142.5	3.307
2	63.628	9419.3	50.265	2	63.489	4166.4	96.693

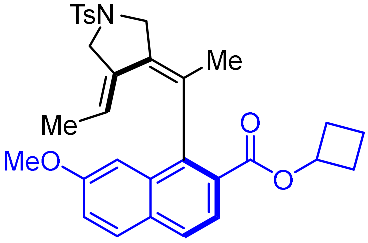

(*R*)-cyclobutyl 1-((*E*)-1-((*Z*)-4-ethylidene-1-tosylpyrrolidin-3-ylidene)ethyl)-7-methoxy-2-naphthoate (8)

The title compound was isolated as a white solid (eluent: petroleum ether/ethyl acetate = 4/1, 30.8 mg, 58%). **^{1}H NMR (600 MHz, Chloroform-d) *δ*** 7.82 – 7.70 (m, 5H), 7.38 (d, *J* = 8.3, 2H), 7.23 (dd, *J* = 8.9, 2.5, 1H), 7.07 – 7.06 (m, 1H), 5.09 – 5.01 (m, 1H), 4.37 (d, *J* = 14.6, 1H), 4.22 (q, *J* = 7.2, 1H), 4.07 (d, *J* = 13.4, 1H), 3.98 (d, *J* = 13.9, 1H), 3.81 (s, 3H), 3.75 (d, *J* = 13.8, 1H), 2.46 (s, 3H), 2.36 – 2.19 (m, 2H), 2.06 (s, 3H), 2.03 – 1.97 (m, 1H), 1.97 – 1.89 (m, 1H), 1.77-1.72 (m, 1H), 1.67 – 1.58 (m, 1H) 1.19 (d, *J* = 7.2, 3H). **^{13}C NMR (150 MHz, Chloroform-d) *δ*** 167.0, 158.6, 143.8, 141.2, 132.9, 132.8, 131.3, 131.0, 129.88, 129.87, 129.6, 128.3, 128.1, 127.2, 126.6, 124.4, 120.6, 120.4, 104.7, 69.3, 55.4, 52.9, 51.6, 30.5, 30.4, 23.4, 21.7, 15.5, 13.8. **HRMS (ESI):** calcd. $C_{31}H_{33}NNaO_5S^+$ for $[M+Na]^+$: 554.1972; found: 554.1992; **$[\alpha]_D^{20}$** = +26 (c = 0.1, $CHCl_3$).

HPLC analysis: IG column (hexane:2-propanol = 90:10, v = 1.0 mL/min, 40 °C, 254 nm); tr (minor) = 26.520 min, tr (major) = 29.937 min, 95% ee.

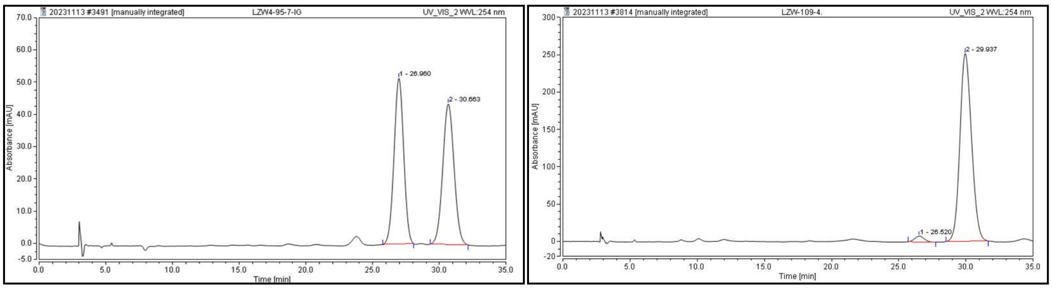

No.	Time	Area	Area (%)	No.	Time	Area	Area (%)

1	26.960	43.066	50.11	1	26.520	6.280	2.43
2	30.663	42.881	49.89	2	29.937	252.457	97.57

(*R*)-furan-3-ylmethyl 1-((*E*)-1-((*Z*)-4-ethylidene-1-tosylpyrrolidin-3-ylidene)ethyl)-7-methoxy-2-naphthoate (9)

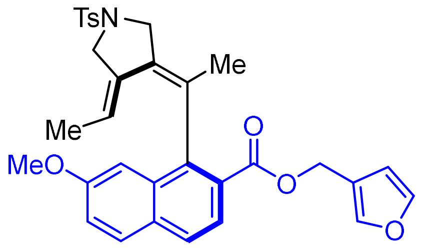

The title compound was isolated as a white solid (eluent: petroleum ether/ethyl acetate = 3/1, 34.5 mg, 62%). **^{1}H NMR (600 MHz, Chloroform-d) *δ*** 7.82 – 7.70 (m, 5H), 7.43 – 7.37 (m, 3H), 7.33 (t, *J* = 1.7, 1H), 7.22 (dd, *J* = 8.9, 2.6, 1H), 7.06 – 7.03 (m, 1H), 6.39 – 6.36 (m, 1H), 5.08 (q, *J* = 12.3, 2H), 4.28 (d, *J* = 14.6, 1H), 4.19 (q, *J* = 7.0, 1H), 3.96 (d, *J* = 14.9, 1H), 3.91 (d, *J* = 14.1, 1H), 3.79 (s, 3H), 3.72 (d, *J* = 13.0, 1H), 2.46 (s, 3H), 1.98 (s, 3H), 1.15 (d, *J* = 7.2, 3H). **^{13}C NMR (150 MHz, Chloroform-d) *δ*** 167.3, 158.6, 143.8, 143.6, 141.8, 141.6, 133.1, 133.0, 131.3, 131.1, 129.92, 129.85, 129.6, 128.2, 128.1, 127.2, 126.3, 124.2, 120.5, 120.4, 110.8, 104.8, 58.2, 55.4, 52.7, 51.5, 23.3, 21.7, 15.5. **HRMS (ESI):** calcd. for $C_{32}H_{31}NNaO_6S^+$[M+Na]$^+$: 580.1764; found: 580.1763; **$[\alpha]_D^{20}$** = +74 (c = 0.1, $CHCl_3$). **HPLC analysis:** IG column (hexane:2-propanol = 85:15, v = 1.0 mL/min, 40 ^{o}C, 254 nm); tr (major) =34.353 min, tr (minor)= 38.873 min, 96% ee.

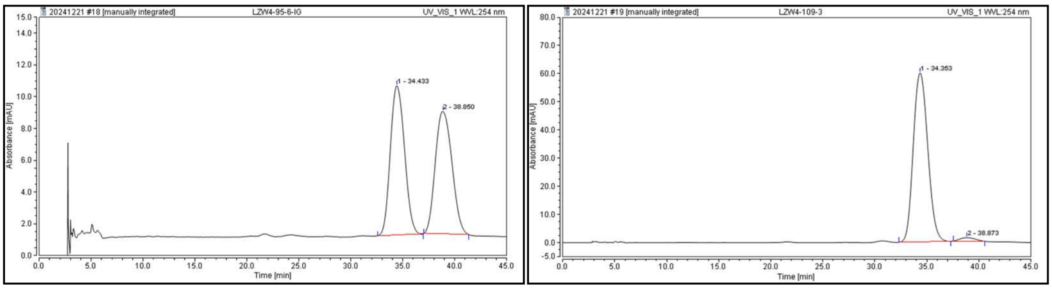

No.	Time	Area	Area (%)	No.	Time	Area	Area (%)
1	34.433	14.851	50.92	1	34.353	96.664	97.90
2	38.850	14.317	49.08	2	38.873	2.071	2.10

(*R*)-benzyl 1-((*E*)-1-((*Z*)-4-ethylidene-1-tosylpyrrolidin-3-ylidene)ethyl)-7-methoxy-2-naphthoate (10)

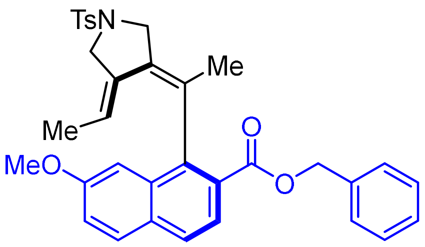

The title compound was isolated as a white solid (eluent: petroleum ether/ethyl acetate = 4/1, 38.6 mg, 68%). **^{1}H NMR (600 MHz, Chloroform-d) *δ*** 7.84 – 7.71 (m, 5H), 7.38 (d, *J* = 8.0, 2H),

7.34 – 7.31 (m, 5H), 7.22 (dd, *J* = 8.9, 2.6, 1H), 7.05 – 7.02 (m, 1H), 5.30 -5.11 (m, 2H), 4.26 (d, *J* = 13.1, 1H), 4.21 (q, *J* = 7.0, 1H), 3.99 – 3.87 (m, 2H), 3.77 (s, 3H), 3.76 – 3.70 (m, 1H), 2.45 (s, 3H), 1.95 (s, 3H), 1.15 (d, *J* = 7.2, 3H).**^{13}C NMR (150 MHz, Chloroform-d) *δ*** 167.2, 158.6, 143.8, 141.7, 135.9, 133.2, 133.0, 131.3, 131.1, 129.9, 129.8, 129.6, 128.7, 128.5, 128.2, 128.0, 127.2, 126.3, 124.3, 120.5, 120.4, 104.8, 67.0, 55.4, 52.7, 51.5, 23.3, 21.7, 15.5. **HRMS (ESI):** calcd. for $C_{34}H_{33}NNaO_5S^+$[M+Na]$^+$: 590.1972; found: 590.1969; **$[\alpha]_D^{20}$** = +46 (c = 0.1, $CHCl_3$).

HPLC analysis: IG column (hexane:2-propanol = 85:15, v = 1.0 mL/min, 40 °C, 254 nm); tr (major) =29.853 min, tr (minor) = 33.573 min, 93% ee.

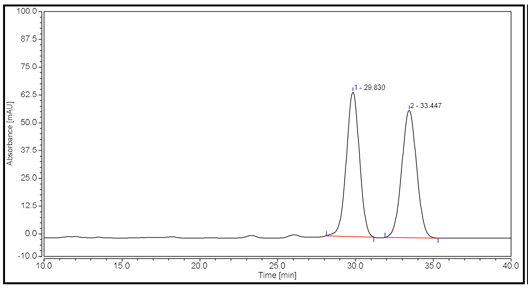

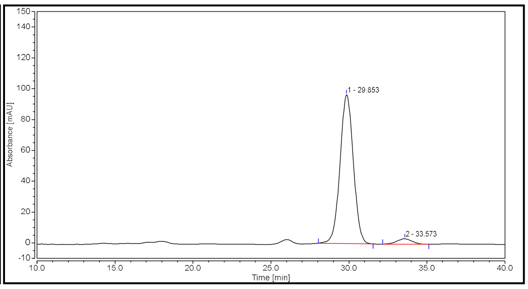

No.	Time	Area	Area (%)	No.	Time	Area	Area (%)
1	29.830	63.690	50.44	1	29.853	95.070	96.45
2	33.447	62.568	49.56	2	33.573	3.497	3.55

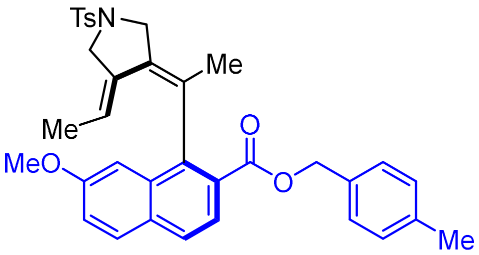

(*R*)-4-methylbenzyl 1-((*E*)-1-((*Z*)-4-ethylidene-1-tosylpyrrolidin-3-ylidene)ethyl)-7-methoxy-2-naphthoate (11)

This compound was isolated as a white solid (eluent: petroleum ether/ethyl acetate = 4/1, 27.9 mg, 42%).**^{1}H NMR (600 MHz, Chloroform-d) *δ*** 7.83 – 7.77 (m, 3H), 7.76 – 7.70 (m, 2H), 7.39 – 7.35 (m, 2H), 7.25 – 7.19 (m, 3H), 7.18 – 7.15 (m, 2H), 7.04 – 7.01 (m, 1H), 5.21 – 5.12 (m, 2H), 4.26 (d, *J* = 12.0, 1H), 4.20 (q, *J* = 7.2, 1H), 3.97 (d, *J* = 13.3, 1H), 3.89 (d, *J* = 13.4, 1H), 3.78 – 3.73 (m, 4H), 2.44 (s, 3H), 2.38 (s, 3H), 1.94 (s, 3H), 1.15 (d, *J* = 7.1, 3H).**^{13}C NMR (150 MHz, Chloroform-d) *δ*** 167.2, 158.6, 143.7, 141.7, 138.4, 133.2, 133.0, 132.9, 131.2, 131.1, 129.9, 129.8, 129.6, 129.4, 128.6, 128.3, 128.0, 127.2, 126.4, 124.3, 120.43, 120.35, 104.8, 66.9, 55.3, 52.7, 51.5, 23.2, 21.7, 21.4, 15.5. **HRMS (ESI):** calcd. for $C_{35}H_{35}NNaO_5S^+$[M+Na]$^+$: 604.2128; found: 604.2126; **$[\alpha]_D^{20}$** = +68 (c = 0.1, $CHCl_3$).

HPLC analysis: IG column (hexane:2-propanol = 85:15, v = 1.0 mL/min, 40 °C, 228nm); tr (major) =30.143 min, tr (minor) = 45.960 min, 93% ee.

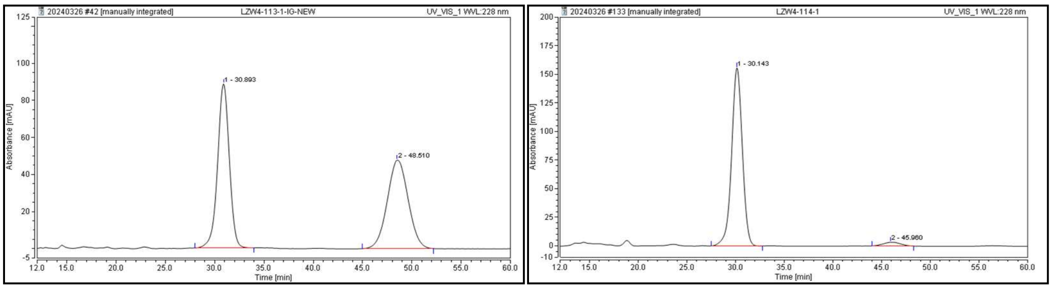

No.	Time	Area	Area (%)
1	30.893	121.319	50.81
2	48.510	117.428	49.19

No.	Time	Area	Area (%)
1	30.143	198.964	96.74
2	45.960	6.705	3.26

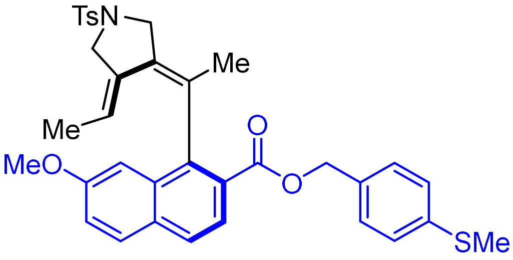

(*R*)-4-(methylthio)benzyl 1-((*E*)-1-((*Z*)-4-ethylidene-1-tosylpyrrolidin-3-ylidene)ethyl)-7-methoxy-2-naphthoate (12)

The title compound was isolated as a white solid (eluent: petroleum ether/ethyl acetate = 4/1, 37.4 mg, 61%). **^{1}H NMR (600 MHz, Chloroform-d)** ***δ*** 7.84 – 7.77 (m, 3H), 7.76 – 7.70 (m, 2H), 7.40 – 7.35 (m, 2H), 7.27 (d, *J* = 2.0, 1H), 7.25 – 7.20 (m, 3H), 7.04 – 7.01 (m, 1H), 5.22 – 5.11 (m, 2H), 4.24 (d, *J* = 11.8, 1H), 4.20 (q, *J* = 7.2, 1H), 4.03 (d, *J* = 11.7, 1H), 3.87 (d, *J* = 14.2, 1H), 3.81 – 3.74 (m, 4H), 2.51 (s, 3H), 2.45 (s, 3H), 1.95 (s, 3H), 1.14 (d, *J* = 7.2, 3H). **^{13}C NMR (150 MHz, Chloroform-d)** ***δ*** 167.0, 158.5, 143.7, 141.6, 139.0, 133.0, 132.9, 132.4, 131.1, 131.0, 129.8, 129.7, 129.4, 129.1, 128.2, 127.9, 127.1, 126.5, 126.1, 124.2, 120.4, 120.3, 104.7, 66.5, 55.2, 52.7, 51.4, 23.1, 21.6, 15.7, 15.4. **HRMS (ESI):** calcd. for $C_{35}H_{35}NNaO_5S_2^+$[M+Na]$^+$: 636.1849; found: 636.1848; **$[\alpha]_D^{20}$** = +80 (c = 0.1, $CHCl_3$).

HPLC analysis: ID column (hexane:2-propanol = 85:15, v = 1.0 mL/min, 40 °C, 227 nm); tr (minor) = 25.077 min, tr (major) = 29.070 min, 87% ee.

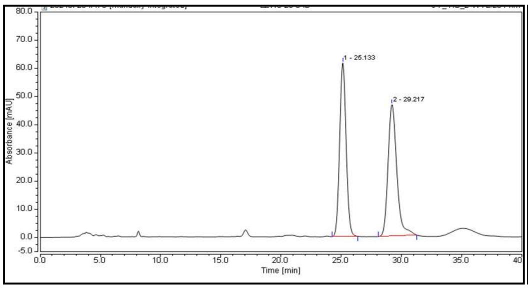

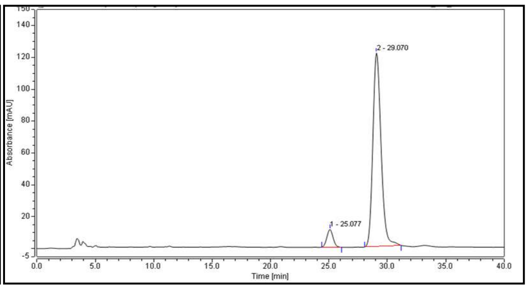

No.	Time	Area	Area (%)	No.	Time	Area	Area (%)
1	25.133	38.470	49.35	1	25.077	6.782	6.43
2	29.217	39.488	50.65	2	29.070	98.766	93.57

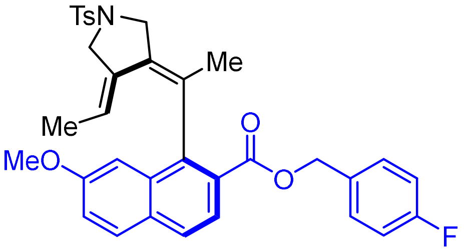

(*R*)-4-fluorobenzyl 1-((*E*)-1-((*Z*)-4-ethylidene-1-tosylpyrrolidin-3-ylidene)ethyl)-7-methoxy-2-naphthoate (13)

The title compound was isolated as a white solid (eluent: petroleum ether/ethyl acetate = 5/1, 28.8 mg, 49%). **^{1}H NMR (600 MHz, Chloroform-d) *δ*** 7.83 – 7.77 (m, 3H), 7.77 – 7.71 (m, 2H), 7.40 – 7.37 (m, 2H), 7.35 – 7.29 (m, 2H), 7.24 – 7.21 (m, 1H), 7.07 – 6.97 (m, 3H), 5.25 – 5.11 (m, 2H), 4.25 (d, *J* = 14.7, 1H), 4.20 (q, *J* = 7.2, 1H), 3.99 (d, *J* = 11.6, 1H), 3.89 (d, *J* = 13.3, 1H), 3.80 – 3.73 (m, 3H), 2.45 (s, 3H), 1.95 (s, 3H), 1.14 (d, *J* = 7.2, 3H).**^{13}C NMR (150 MHz, Chloroform-d) *δ*** 167.0, 162.7 (d, *J* = 247.6), 158.5, 143.7, 141.6, 132.9, 132.9, 131.7 (d, *J* = 3.3), 131.1, 131.0, 130.4, 130.3, 129.8, 129.7, 129.5, 128.2, 127.9, 127.2, 126.0, 124.1, 120.4, 120.3, 115.6, 115.5, 104.7, 66.1, 55.3, 52.6, 51.4, 23.1, 21.5, 15.4.**^{19}F NMR (376 MHz, Chloroform-d) *δ*** -113.1(m). **HRMS (ESI):** calcd. for $C_{34}H_{32}FNNaO_5S^+$[M+Na]$^+$: 608.1877; found: 608.1882; **$[\alpha]_D^{20}$** = +124 (c = 0.1, $CHCl_3$).

HPLC analysis: ID column (hexane:2-propanol = 85:15, v = 1.0 mL/min, 40 ^{o}C, 227 nm); tr (minor) =23.043 min, tr (major) = 25.850 min, 93% ee.

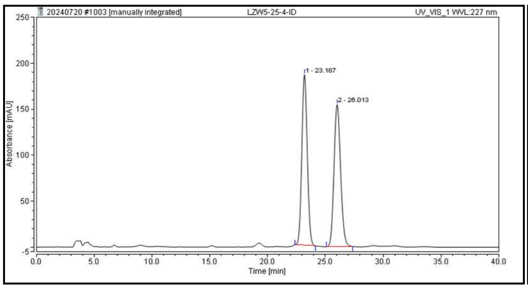

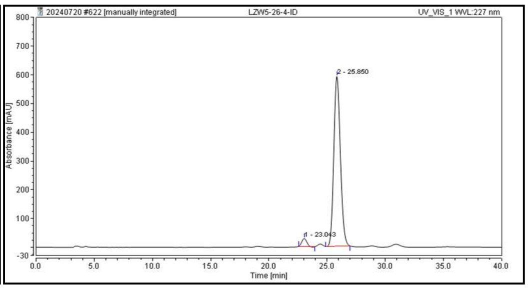

No.	Time	Area	Area (%)	No.	Time	Area	Area (%)

1	23.187	101.194	49.73	1	23.043	13.409	3.35
2	26.013	102.292	50.27	2	25.850	386.942	96.65

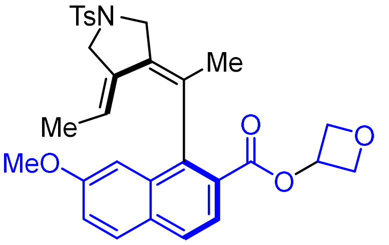

(*R*)-oxetan-3-yl 1-((*E*)-1-((*Z*)-4-ethylidene-1-tosylpyrrolidin-3-ylidene)ethyl)-7-methoxy-2-naphthoate (14)

The title compound was isolated as a white solid (eluent: petroleum ether/ethyl acetate = 2/1, 26.1 mg, 49%). **^{1}H NMR (600 MHz, Chloroform-d) *δ*** 7.84 – 7.74 (m, 5H), 7.38 (d, *J* = 8.3, 2H), 7.28 – 7.23 (m, 1H), 7.10 – 7.07 (m, 1H), 5.54 – 5.48 (m, 1H), 4.90 – 4.83 (m, 2H), 4.60 – 4.53 (m, 2H), 4.42 – 4.37 (m, 1H), 4.22 (q, *J* = 7.4, 1H), 4.08 (d, *J* = 13.5, 1H), 3.98 (d, *J* = 13.2, 1H), 3.81 (s, 3H), 3.74 (d, *J* = 13.2, 1H), 2.47 (s, 3H), 2.07 (s, 3H), 1.19 (d, *J* = 6.6, 3H). **^{13}C NMR (150 MHz, Chloroform-d) *δ*** 166.5, 158.8, 143.9, 142.1, 133.0, 132.9, 131.31, 131.28, 129.91, 129.88, 128.1, 128.0, 127.4, 125.2, 124.1, 120.9, 120.7, 104.7, 77.6, 68.5, 55.4, 52.9, 51.5, 23.3, 21.7, 15.6. **HRMS (ESI):** calcd. for $C_{30}H_{31}NNaO_6S^+$[M+Na]$^+$: 556.1764; found: 556.1771; **$[\alpha]_D^{20}$** = +168 (c = 0.1, $CHCl_3$). **HPLC analysis:** IG column (hexane:2-propanol = 85:15, v = 1.0 mL/min, 40 ^{o}C, 227 nm); tr (minor) =25.587 min, tr (major) = 28.157 min, 94% ee.

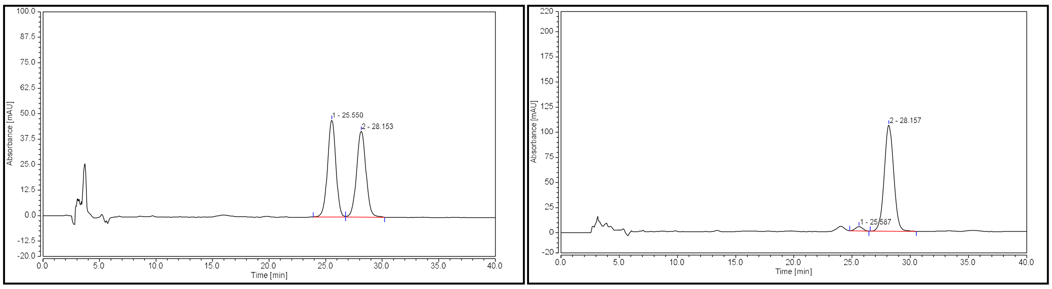

No.	Time	Area	Area (%)	No.	Time	Area	Area (%)
1	25.550	40.580	49.94	1	25.587	3.156	3.06
2	28.153	40.683	50.06	2	28.157	100.102	96.94

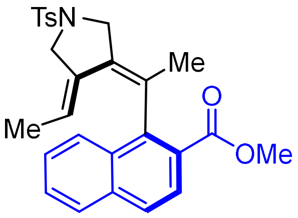

(*R*)-methyl 1-((*E*)-1-((*Z*)-4-ethylidene-1-tosylpyrrolidin-3-ylidene)ethyl)-2-naphthoate (15)

The title compound was isolated as a white solid (eluent: petroleum ether/ethyl acetate = 4/1, 31.8 mg, 69%). **^{1}H NMR (600 MHz, Chloroform-d) *δ*** 7.95 (d, *J* = 8.6, 1H), 7.86 (d, *J* = 7.5, 1H), 7.83 – 7.74 (m, 4H), 7.59 – 7.54 (m, 1H), 7.49 – 7.43 (m, 1H), 7.39 (d, *J*

= 7.8, 2H), 4.40 (d, *J* = 11.9, 1H), 4.16 – 4.06 (m, 2H), 3.97 (d, *J* = 13.4, 1H), 3.84 – 3.74 (m, 4H), 2.47 (s, 3H), 2.05 (s, 3H), 1.14 (d, *J* = 6.6, 3H). **^{13}C NMR (150 MHz, Chloroform-d) *δ*** 167.2, 143.7, 143.6, 135.5, 133.0, 129.9, 129.8, 129.3, 128.2, 128.1, 128.0, 127.9, 127.4, 127.1, 126.33, 126.28, 125.3, 120.2, 52.8, 52.1, 51.5, 23.5, 21.6, 15.4. **HRMS (ESI):** calcd. for $C_{27}H_{27}NNaO_4S^+$[M+Na]$^+$: 484.1553; found: 484.1566; **[α]$_D^{20}$** = +96 (c = 0.1, $CHCl_3$).

HPLC analysis: IG column (hexane:2-propanol = 85:15, v = 1.0 mL/min, 40 °C, 227 nm); tr (major) =15.957 min, tr (minor) = 18.353 min, 82% ee.

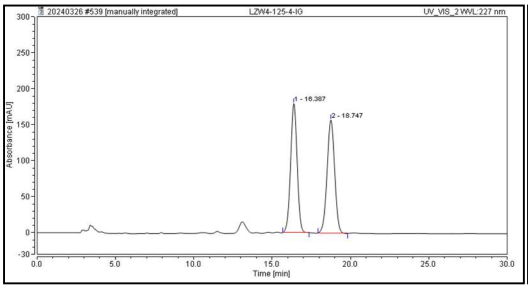

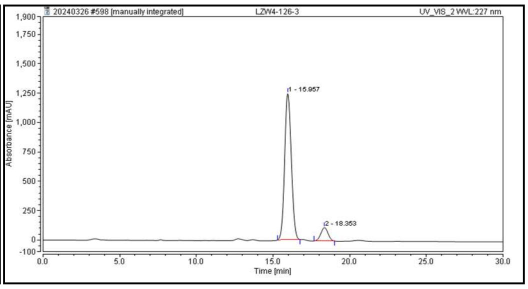

No.	Time	Area	Area (%)	No.	Time	Area	Area (%)
1	16.387	86.607	49.59	1	15.957	625.815	90.96
2	18.747	88.048	50.41	2	18.353	62.177	9.04

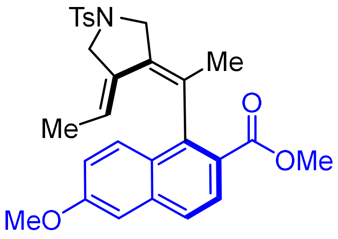

(*R*)-methyl 1-((*E*)-1-((*Z*)-4-ethylidene-1-tosylpyrrolidin-3-ylidene)ethyl)-6-methoxy-2-naphthoate (16)

The title compound was isolated as a white solid (eluent: petroleum ether/ethyl acetate = 4/1, 30.7 mg, 64%). **^{1}H NMR (600 MHz, Chloroform-d) *δ*** 7.95 (d, *J* = 8.6, 1H), 7.79 (d, *J* = 8.3, 2H), 7.70 – 7.63 (m, 2H), 7.38 (d, *J* = 8.5, 2H), 7.14 – 7.07 (m, 2H), 4.39 (d, *J* = 12.7, 1H), 4.12 (d, *J* = 12.8, 2H), 3.98 (d, *J* = 13.5, 1H), 3.93 (s, 3H), 3.80 – 3.75 (m, 4H), 2.47 (s, 3H), 2.03 (s, 3H), 1.15 (d, *J* = 7.1, 3H). **^{13}C NMR (150 MHz, Chloroform-d) *δ*** 167.2, 159.5, 143.9, 143.8, 137.4, 133.1, 129.9, 129.1, 128.3, 128.2, 128.0, 127.2, 126.2, 125.1, 122.8, 120.1, 119.9, 106.2, 55.5, 52.9, 52.0, 51.6, 23.6, 21.7, 15.5. **HRMS (ESI):** calcd. for $C_{28}H_{29}NNaO_5S^+$[M+Na]$^+$: 514.1659; found: 514.1665; **[α]$_D^{20}$** = +90 (c = 0.1, $CHCl_3$).

HPLC analysis: IC column (hexane:2-propanol = 80:20, v = 1.0 mL/min, 40 °C, 254 nm); tr (major) =34.879 min, tr (minor) = 37.919 min, 81% ee.

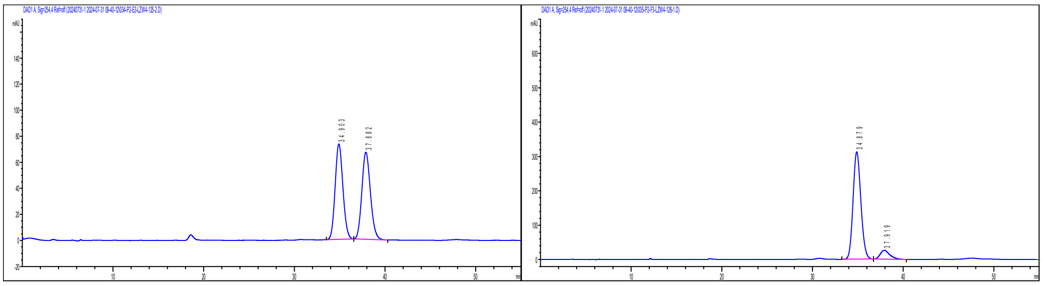

No.	Time	Area	Area (%)	No.	Time	Area	Area (%)
1	34.903	4368.4	49.764	1	34.879	18692.4	90.81
2	37.882	4409.8	50.236	2	37.919	1891.7	9.19

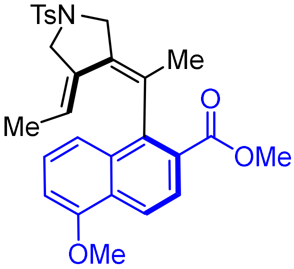

(*R*)-methyl 1-((*E*)-1-((*Z*)-4-ethylidene-1-tosylpyrrolidin-3-ylidene)ethyl)-5-methoxy-2-naphthoate (17)

The title compound was isolated as a white solid (eluent: petroleum ether/ethyl acetate = 4/1, 25.5 mg, 52%).^{1}**H NMR (600 MHz, Chloroform-d) *δ*** 8.25 (d, *J* = 8.9, 1H), 7.92 (d, *J* = 8.9, 1H), 7.79 (d, *J* = 8.2, 2H), 7.40 – 7.31 (m, 4H), 6.90 (d, *J* = 7.1, 1H), 4.37 (d, *J* = 13.6, 1H), 4.15 – 4.07 (m, 2H), 4.01 (s, 3H), 3.97 (d, *J* = 12.8, 1H), 3.80 – 3.75 (m, 4H), 2.47 (s, 3H), 2.04 (s, 3H), 1.15 (d, *J* = 7.2, 3H).^{13}**C NMR (150 MHz, Chloroform-d) *δ*** 167.3, 155.5, 143.6, 142.8, 133.1, 133.0, 131.1, 129.8, 129.2, 128.3, 127.9, 127.7, 127.1, 125.9, 125.6, 121.5, 120.1, 118.3, 105.9, 55.7, 52.8, 52.0, 51.5, 23.4, 21.6, 15.4. **HRMS (ESI):** calcd. for $C_{28}H_{29}NNaO_5S^+$[M+Na]$^+$: 514.1659; found: 514.1653; **$[\alpha]_D^{20}$** = +98 (c = 0.1, $CHCl_3$).

HPLC analysis: IC column (hexane:2-propanol = 85:15, v = 1.0 mL/min, 40 ^{o}C, 254 nm); tr (major) = 23.158 min, tr (minor) = 25.022 min, 79% ee.

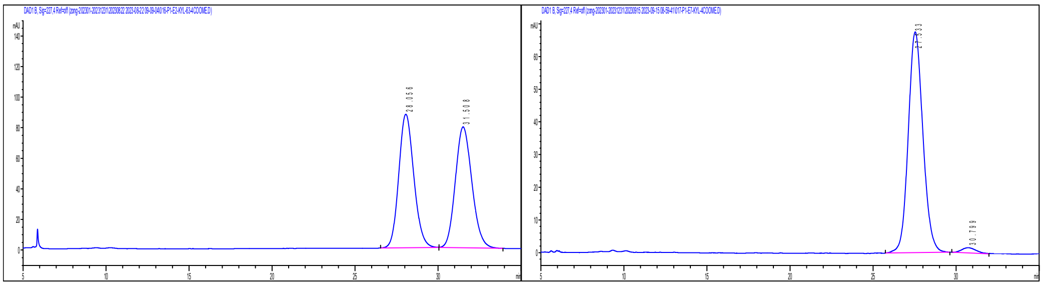

No.	Time	Area	Area (%)	No.	Time	Area	Area (%)
1	23.471	1191.3	48.536	1	23.158	2383.9	89.178
2	25.386	1263.2	51.464	2	25.022	289.3	10.822

(*R*)-methyl 1-((*E*)-1-((*Z*)-4-ethylidene-1-tosylpyrrolidin-3-ylidene)ethyl)-4-methyl-2-naphthoate (18)

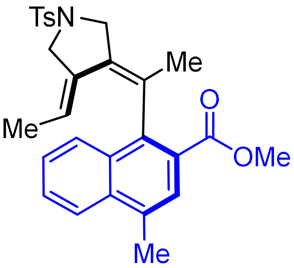

The title compound was isolated as a white solid (eluent: petroleum ether/ethyl acetate = 4/1, 31.4 mg, 66%). **^{1}H NMR (600 MHz, Chloroform-d) *δ*** 8.01 (d, *J* = 8.3, 1H), 7.83 – 7.76 (m, 4H), 7.63 – 7.58 (m, 1H), 7.50 – 7.44 (m, 1H), 7.38 (d, *J* = 8.3, 2H), 4.38 (d, *J* = 14.7, 1H), 4.19 – 4.09 (m, 2H), 3.97 (d, *J* = 14.1, 1H), 3.83 – 3.75 (m, 4H), 2.71 (s, 3H), 2.47 (s, 3H), 2.03 (s, 3H), 1.15 (d, *J* = 7.0, 3H).**^{13}C NMR (150 MHz, Chloroform-d) *δ*** 167.5, 143.8, 141.8, 134.9, 134.0, 133.2, 133.1, 130.0, 129.9, 129.5, 128.3, 128.1, 128.0, 127.0, 126.84, 126.80, 124.9, 124.6, 120.2, 52.9, 52.1, 51.7, 23.8, 21.7, 19.6, 15.5. **HRMS (ESI):** calcd. for $C_{28}H_{29}NNaO_4S^+$[M+Na]$^+$: 498.1710; found: 498.1709; **[α]$_D^{20}$** = +102 (c = 0.1, $CHCl_3$).

HPLC analysis: IE column (hexane:2-propanol = 90:10, v = 1.0 mL/min, 40 °C, 254 nm); tr (major) =30.234 min, tr (minor) =33.575 min, 84% ee.

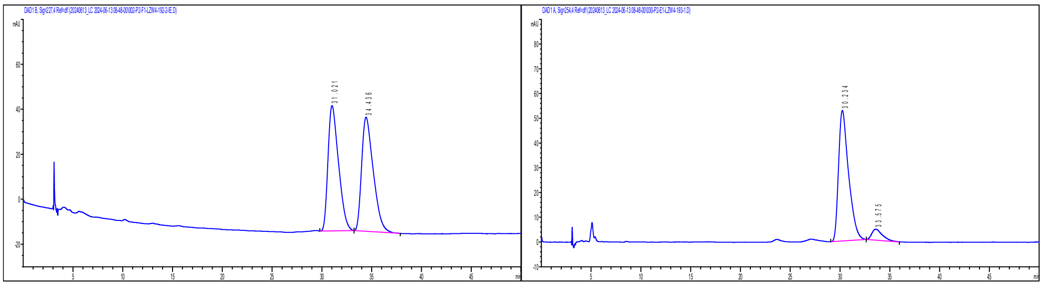

No.	Time	Area	Area (%)	No.	Time	Area	Area (%)
1	31.021	4098	49.744	1	30.234	3700.9	91.841
2	34.436	4140.2	50.256	2	33.575	328.8	8.159

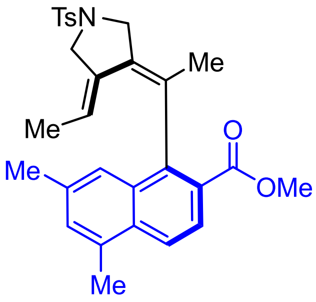

(*R*)-methyl 1-((*E*)-1-((*Z*)-4-ethylidene-1-tosylpyrrolidin-3-ylidene)ethyl)-5,7-dimethyl-2-naphthoate (19)

The title compound was isolated as a white solid (eluent: petroleum ether/ethyl acetate = 4/1, 27.9 mg, 57%). **^{1}H NMR (600 MHz, Chloroform-d) *δ*** 7.93 – 7.85 (m, 2H), 7.80 (d, *J* = 8.3, 2H), 7.43 – 7.36 (m, 3H), 7.25 (s, 1H), 4.39 (d, *J* = 13.3, 1H), 4.15 – 4.09 (m, 1H), 3.98 (d, *J* = 12.6, 1H), 3.78 – 3.71 (m, 4H), 2.67 (s, 3H), 2.46 (s, 3H), 2.44 (s, 3H), 2.04 (s, 3H), 1.16 (d, *J* = 7.2, 3H).**^{13}C NMR (150 MHz, Chloroform-d) *δ*** 167.7, 143.8, 143.1, 136.6, 134.5, 133.2, 133.12, 133.06, 131.4, 130.6,

129.9, 129.3, 128.7, 128.0, 125.30, 125.27, 123.47, 123.46, 120.2, 52.9, 52.0, 51.6, 23.7, 22.1, 21.7, 19.6, 15.5. **HRMS (ESI):** calcd. for $C_{29}H_{31}NNaO_4S^+$[M+Na]$^+$: 512.1866; found: 512.1875; **[α]$_D^{20}$** = +96 (c = 0.1, $CHCl_3$).

HPLC analysis: IG column (hexane:2-propanol = 97:3, v = 1.0 mL/min, 40 °C, 254 nm); tr (minor) =58.237 min, tr (major) =63.647 min, 92% ee.

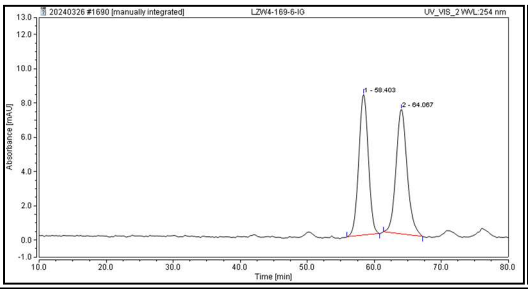

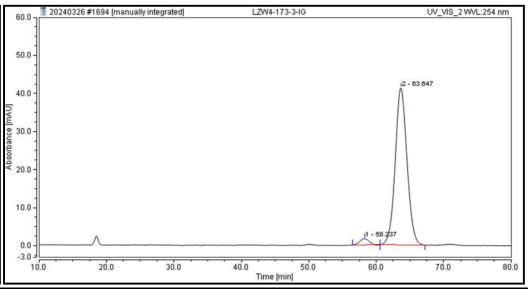

No.	Time	Area	Area (%)	No.	Time	Area	Area (%)
1	58.403	13.559	49.32	1	58.237	2.636	3.87
2	64.067	13.935	50.68	2	63.647	83.767	96.13

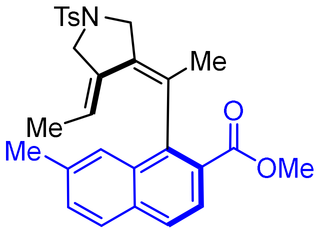

(*R*)-methyl 1-((*E*)-1-((*Z*)-4-ethylidene-1-tosylpyrrolidin-3-ylidene)ethyl)-7-methyl-2-naphthoate (20)

The title compound was isolated as a white solid (eluent: petroleum ether/ethyl acetate = 4/1, 33.3 mg, 70%). **^{1}H NMR (600 MHz, Chloroform-d) *δ*** 7.89 – 7.83 (m, 1H), 7.82 – 7.79 (m, 2H), 7.78 – 7.73 (m, 2H), 7.54 (s, 1H), 7.42 – 7.37 (m, 3H), 4.41 (d, *J* = 13.2, 1H), 4.16 – 4.08 (m, 2H), 3.98 (d, *J* = 12.2, 1H), 3.78 – 3.73 (m, 4H), 2.49 (s, 3H), 2.46 (s, 3H), 2.05 (s, 3H), 1.16 (d, *J* = 7.2, 3H). **^{13}C NMR (150 MHz, Chloroform-d) *δ*** 167.6, 143.8, 142.8, 137.1, 133.9, 133.2, 133.1, 130.5, 130.3, 129.9, 129.4, 128.3, 128.2, 128.1, 127.2, 125.53, 125.50, 125.2, 120.2, 52.9, 52.1, 51.6, 23.6, 22.2, 21.7, 15.5. **HRMS (ESI):** calcd. for $C_{28}H_{29}NNaO_4S^+$[M+Na]$^+$: 498.1710; found: 498.1714; **[α]$_D^{20}$** = +60 (c = 0.1, $CHCl_3$).

HPLC analysis: IG column (hexane:2-propanol = 90:10, v = 1.0 mL/min, 40 °C, 227 nm); tr (minor) =17.753 min, tr (major) =21.597 min, 91% ee.

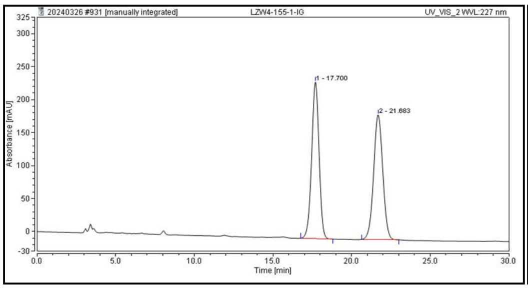

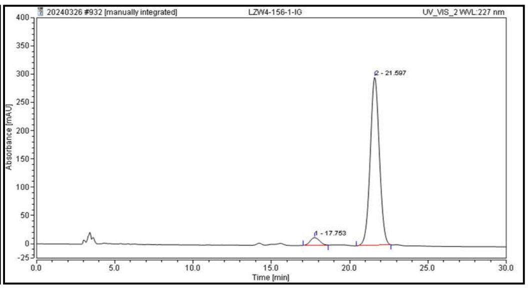

No.	Time	Area	Area (%)	No.	Time	Area	Area (%)
1	17.700	129.302	50.26	1	17.753	9.187	4.38
2	21.683	127.946	49.74	2	21.597	200.717	95.62

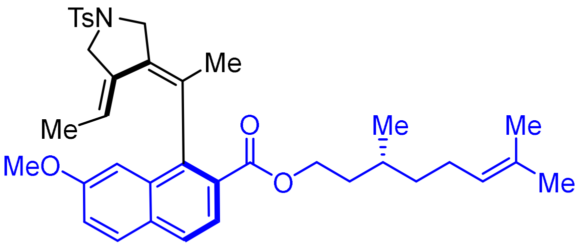

(*R*)-3,7-dimethyloct-6-en-1-yl 1-((*E*)-1-((*Z*)-4-ethylidene-1-tosylpyrrolidin-3-ylidene)ethyl)-7-methoxy-2-naphthoate (21)

The title compound was isolated as a yellow solid (eluent: petroleum ether/ethyl acetate = 5/1, 31.4 mg, 51%), dr > 20:1. **^{1}H NMR (600 MHz, Chloroform-d) *δ*** 7.81 – 7.78 (m, 3H), 7.77 – 7.74 (m, 1H), 7.74 – 7.69 (m, 1H), 7.37 (d, *J* = 8.2, 2H), 7.22 (dd, *J* = 8.9, 2.7, 1H), 7.10 – 7.06 (m, 1H), 5.09 (t, *J* = 7.2, 1H), 4.39 (d, *J* = 13.0, 1H), 4.28 – 4.15 (m, 3H), 4.09 (d, *J* = 13.5, 1H), 3.96 (d, *J* = 13.0, 1H), 3.79 (s, 3H), 3.75 (d, *J* = 13.5, 1H), 2.46 (s, 3H), 2.06 (s, 3H), 2.04 – 1.98 (m, 1H), 1.97 – 1.91 (m, 1H), 1.71 – 1.66 (m, 5H), 1.60 (s, 3H), 1.59 – 1.55 (m, 1H), 1.52 – 1.44 (m, 1H), 1.40 – 1.31 (m, 1H), 1.17 (d, *J* = 7.4, 3H), 0.92 (d, *J* = 6.7, 3H). **^{13}C NMR (150 MHz, Chloroform-d) *δ*** 167.2, 158.5, 143.6, 141.5, 132.91, 132.88, 131.4, 131.1, 130.9, 129.8, 129.7, 129.3, 128.3, 127.9, 127.0, 126.4, 124.5, 124.1, 120.3, 104.5, 63.5, 55.3, 52.7, 51.4, 36.9, 35.6, 29.6, 25.7, 25.4, 23.2, 21.6, 19.4, 17.7, 15.4. **HRMS (ESI):** calcd. for $C_{37}H_{45}NNaO_5S^+$[M+Na]$^+$: 638.2911; found: 638.2903; **$[\alpha]_D^{20}$** = +150 (c = 0.1, $CHCl_3$).

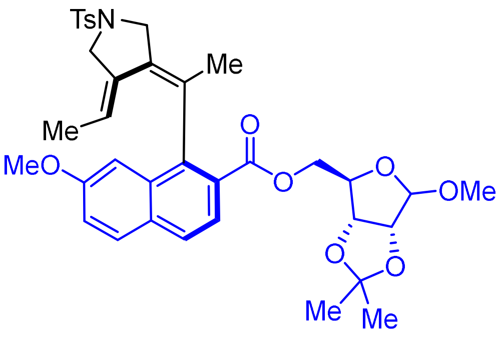

(*R*)- ((3a*R*,4*R*,6a*R*)-6-methoxy-2,2-dimethyltetrahydrofuro[3,4-d][1,3]dioxol-4-yl)methyl 1-((*E*)-1-((*Z*)-4-ethylidene-1-tosylpyrrolidin-3-ylidene)ethyl)-7-methoxy-2-naphthoate (22)

The title compound was isolated as a yellow

solid (eluent: petroleum ether/ethyl acetate = 3/1, 30.0 mg, 45%). dr > 20:1. **^{1}H NMR (600 MHz, Chloroform-d) *δ*** 7.88 – 7.85 (m, 1H), 7.81 – 7.78 (m, 2H), 7.78 – 7.75 (m, 1H), 7.75 – 7.73 (m, 1H), 7.41 – 7.35 (m, 2H), 7.24 (dd, *J* = 8.9, 2.6, 1H), 7.10 – 7.06 (m, 1H), 4.94 (s, 1H), 4.65 (d, *J* = 6.0, 1H), 4.59 (d, *J* = 6.0, 1H), 4.43 – 4.36 (m, 2H), 4.26 – 4.20 (m, 3H), 4.17 (d, *J* = 11.7, 1H), 3.96 (d, *J* = 12.1, 1H), 3.78 (s, 3H), 3.77 – 3.73 (m, 1H), 3.27 (s, 3H), 2.47 (s, 3H), 2.07 (s, 3H), 1.52 (s, 3H), 1.32 (s, 3H), 1.17 (d, *J* = 6.3, 3H). **^{13}C NMR (150 MHz, Chloroform-d) *δ*** 166.5, 158.6, 143.7, 142.1, 133.0, 132.9, 131.2, 131.0, 129.8, 129.7, 129.5, 128.0, 127.9, 127.2, 125.5, 124.2, 120.6, 120.3, 112.6, 109.3, 104.6, 85.2, 84.2, 81.9, 65.3, 55.3, 54.8, 52.7, 51.4, 26.5, 25.0, 23.2, 21.6, 15.4. **HRMS (ESI):** calcd. for $C_{36}H_{41}NNaO_9S^+$[M+Na]$^+$: 686.2394; found: 686.2392; **$[\alpha]_D^{20}$** = +80 (c = 0.1, $CHCl_3$).

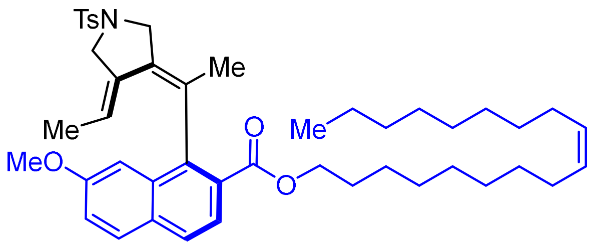

(*R*)- (Z)-octadec-9-en-1-yl 1-((*E*)-1-((*Z*)-4-ethylidene-1-tosylpyrrolidin-3-ylidene)ethyl)-7-methoxy-2-naphthoate (23)

The title compound was isolated as a yellow solid (eluent: petroleum ether/ethyl acetate = 4/1, 30.0 mg, 41%).**^{1}H NMR (600 MHz, Chloroform-d) *δ*** 7.82 – 7.78 (m, 3H), 7.77 – 7.70 (m, 2H), 7.40 – 7.35 (m, 2H), 7.25 – 7.20 (m, 1H), 7.09 – 7.06 (m, 1H), 5.40 – 5.33 (m, 2H), 4.38 (d, *J* = 13.2, 1H), 4.23 (q, *J* = 7.2, 1H), 4.19 – 4.13 (m, 2H), 4.09 (d, *J* = 13.4, 1H), 3.96 (d, *J* = 13.7,1H), 3.79 (s, 3H), 3.75 (d, *J* = 13.2, 1H), 2.46 (s, 3H), 2.05 (s, 1H), 2.03 – 1.99 (m, 3H), 1.67 – 1.58 (m, 2H), 1.37 – 1.21 (m, 22H), 1.17 (d, *J* = 7.2, 3H), 0.90 – 0.85 (m, 3H). **^{13}C NMR (150 MHz, Chloroform-d) *δ*** 167.3, 158.5, 143.6, 141.4, 132.92, 132.86, 131.1, 130.9, 130.2, 130.0, 129.8, 129.7, 129.3, 128.3, 127.9, 127.0, 126.4, 124.1, 120.32, 120.29, 104.5, 65.1, 55.3, 52.7, 51.4, 31.9, 29.8, 29.7, 29.53, 29.49, 29.33, 29.31, 29.24, 29.23, 28.7, 27.24, 27.21, 26.0, 23.2, 22.7, 21.6, 15.4, 14.1. **HRMS (ESI):** calcd. for $C_{45}H_{61}NNaO_5S^+$[M+Na]$^+$: 750.4163; found: 750.4167; **$[\alpha]_D^{20}$** = +168 (c = 0.1, $CHCl_3$).

HPLC analysis: IC column (hexane:2-propanol = 90:10, v = 1.0 mL/min, 40 °C, 227 nm); tr (minor) =17.408 min, tr (major) =19.836 min, 94% ee.

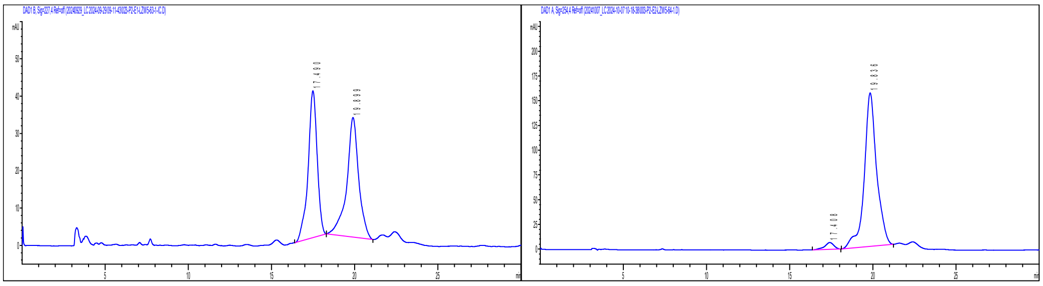

No.	Time	Area	Area (%)	No.	Time	Area	Area (%)
1	17.49	1497.9	48.165	1	17.408	262.4	3.157
2	19.899	1612	51.835	2	19.836	8050.6	96.843

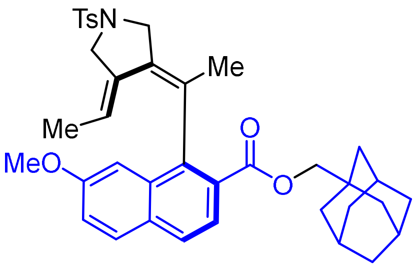

((3*R*,5*R*)-adamantan-1-yl)methyl 1-((*E*)-1-((*Z*)-4-ethylidene-1-tosylpyrrolidin-3-ylidene)ethyl)-7-methoxy-2-naphthoate (24)

The title compound was isolated as a yellow solid (eluent: petroleum ether/ethyl acetate = 4/1, 32.5 mg, 52%). **^{1}H NMR (600 MHz, Chloroform-d) *δ*** 7.80 – 7.76 (m, 3H), 7.76 – 7.71 (m, 2H), 7.37 (d, *J* = 8.1, 2H), 7.22 (dd, *J* = 8.9, 2.5, 1H), 7.11 – 7.07 (m, 1H), 4.41 (d, *J* = 13.2, 1H), 4.28 (q, *J* = 7.2, 1H), 4.05 (d, *J* = 13.1, 1H), 3.99 (d, *J* = 14.0, 1H), 3.86 – 3.83 (m, 1H), 3.83 – 3.75 (m, 4H), 3.74 – 3.66 (m, 1H), 2.46 (s, 3H), 2.06 (s, 3H), 1.92 – 1.85 (m, 3H), 1.72 – 1.67 (m, 3H), 1.62 – 1.55 (m, 4H), 1.52 – 1.46 (m, 5H), 1.18 (d, *J* = 7.1, 3H). **^{13}C NMR (150 MHz, Chloroform-d) *δ*** 167.6, 158.7, 143.8, 141.4, 133.1, 132.9, 131.2, 131.0, 129.9, 129.8, 129.6, 128.4, 128.1, 127.2, 127.0, 124.2, 120.6, 120.4, 104.6, 74.7, 55.4, 52.9, 51.5, 39.4, 37.1, 33.6, 28.2, 23.5, 21.7, 15.5. **HRMS (ESI):** calcd. for $C_{38}H_{43}NNaO_5S^+[M+Na]^+$: 648.2754; found:648.2747; **$[\alpha]_D^{20}$** = +150 (c = 0.1, $CHCl_3$). **HPLC analysis:** IC column (hexane:2-propanol = 85:15, v = 1.0 mL/min, 40 ^oC, 227 nm); tr (minor) = 21.977min, tr (major) = 24.326min, 91% ee.

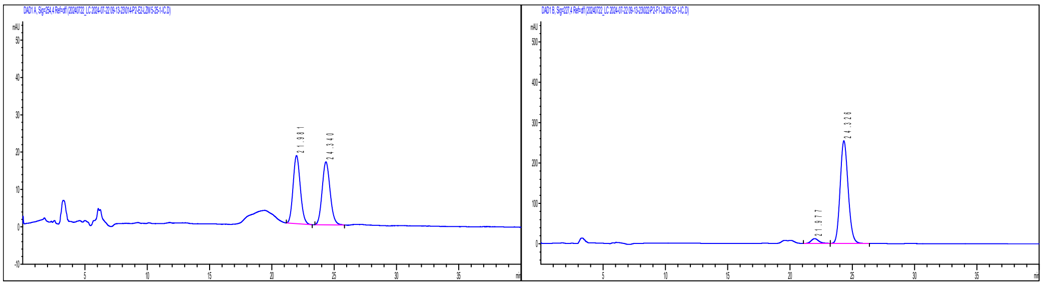

No.	Time	Area	Area (%)	No.	Time	Area	Area (%)
1	21.981	732.3	49.362	1	21.977	502.4	4.241
2	24.34	751.2	50.638	2	24.326	11342	95.759

(*R*)-5-((((9H-fluoren-9-yl)methoxy)carbonyl)amino)pentyl 1-((*E*)-1-((*Z*)-4-ethylidene-1-tosylpyrrolidin-3-ylidene)ethyl)-7-methoxy-2-naphthoate(25)

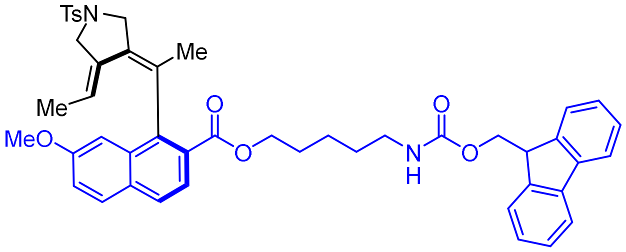

The title compound was isolated as a yellow solid (eluent: petroleum ether/ethyl acetate = 2/1, 38.5 mg, 49%). **^{1}H NMR (600 MHz, Chloroform-d) *δ*** 7.82 – 7.78 (m, 3H), 7.77 – 7.74 (m, 3H), 7.74 – 7.72 (m, 1H)., 7.63 – 7.59 (m, 2H), 7.40 – 7.34 (m, 4H), 7.31 – 7.26 (m, 2H), 7.23 – 7.20 (m, 1H), 7.06 – 7.03 (m, 1H), 5.06 (t, *J* = 6.1, 1H), 4.40 – 4.36 (m, 2H), 4.29 – 4.26 (m, 1H), 4.25 – 4.16 (m, 5H), 3.86 (s, 2H), 3.76 (s, 3H), 3.21 (q, *J* = 6.7, 2H), 2.45 (s, 3H), 2.06 (s, 3H), 1.72 – 1.64 (m, 2H), 1.57 – 1.50 (m, 2H), 1.41 – 1.34 (m, 4H), 1.17 (d, *J* = 7.2, 3H).**^{13}C NMR (150 MHz, Chloroform-d) *δ*** 167.4, 158.5, 156.5, 144.1, 143.7, 141.3, 141.2, 132.8, 132.7, 131.1, 130.9, 129.79, 129.76, 129.3, 128.4, 128.0, 127.6, 127.1, 127.0, 126.5, 125.1, 124.2, 120.5, 120.1, 119.9, 104.7, 66.6, 65.0, 55.2, 52.7, 51.4, 47.3, 41.0, 29.7, 28.7, 26.4, 25.7, 23.3, 21.6, 15.4. **HRMS (ESI):** calcd. for $C_{47}H_{48}N_2NaO_7S^+$[M+Na]$^+$: 807.3074; found: 807.3075; **[α]$_D^{20}$** = +72 (c = 0.1, $CHCl_3$).

HPLC analysis: IG column (hexane:2-propanol = 60:40, v = 1.0 mL/min, 40 ^{o}C, 227 nm); tr (minor) =23.023 min, tr (major) =34.100 min, 95% ee.

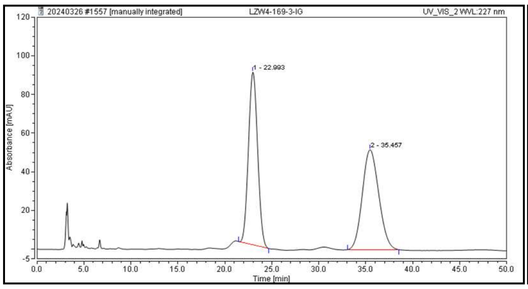

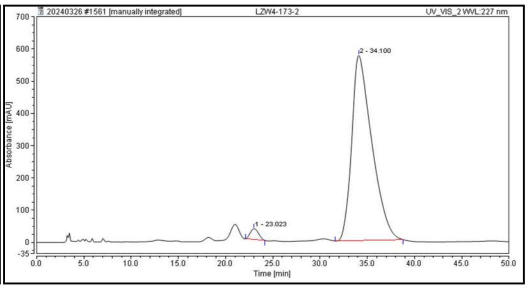

No.	Time	Area	Area (%)	No.	Time	Area	Area (%)
1	22.993	101.243	49.14	1	23.023	34.112	2.34
2	35.457	104.793	50.86	2	34.100	1420.940	97.66

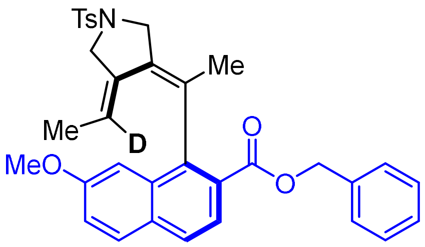

(*R*)-benzyl 1-((*E*)-1-((*Z*)-4-(ethylidene-1-*d*)-1-tosylpyrrolidin-3-ylidene)ethyl)-7-methoxy-2-naphthoate (10-*d*)

The title compound was isolated as a yellow solid (eluent: petroleum ether/ethyl acetate = 4/1, 26.7 mg, 47%). **^{1}H NMR (600 MHz, Chloroform-d) *δ*** 7.86 – 7.71 (m, 5H), 7.40 – 7.36 (m, 2H), 7.36 – 7.29 (m, 5H), 7.25 – 7.20 (m, 1H), 7.05 – 7.02 (m, 1H), 5.26 – 5.22 (m, 1H), 5.20 – 5.17 (m, 1H), 4.26 (d, *J* = 11.9, 1H), 3.96 – 3.87 (m, 2H), 3.78 (s, 3H), 3.75 – 3.70 (m, 1H), 2.46 (s, 3H), 1.95 (s, 3H), 1.14 (s, 3H). **^{13}C NMR (150 MHz, Chloroform-d) *δ*** 167.2, 158.6, 143.8, 141.7, 135.9, 133.2, 133.0, 131.3, 131.1, 129.9, 129.8, 129.6, 128.7, 128.5, 128.2, 128.0, 127.2, 126.3, 124.3, 120.5, 120.4, 104.8, 67.0, 55.4, 52.7, 51.5, 23.3, 21.7, 15.5. **HRMS (ESI):** calcd. for $C_{34}H_{32}DNNaO_5S^+[M+Na]^+$: 591.2034; found:591.2035; **$[α]_D^{20}$** = +60 (c = 0.1, $CHCl_3$).

HPLC analysis: IG column (hexane:2-propanol = 85:15, v = 1.0 mL/min, 40 ^{o}C, 254 nm); tr (major) =37.500 min, tr (minor) =43.637 min, 96% ee.

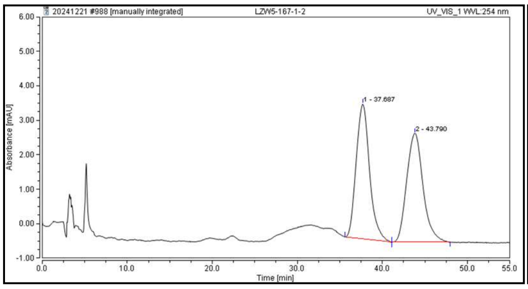

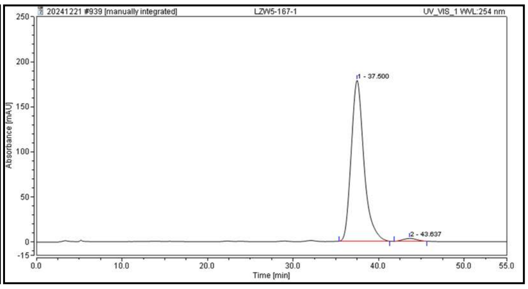

No.	Time	Area	Area (%)	No.	Time	Area	Area (%)
1	37.677	8.022	49.66	1	37.500	308.897	98.23
2	43.837	8.133	50.34	2	43.637	5.888	1.77

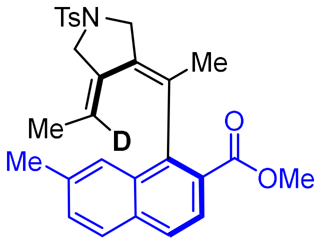

(*R*)-methyl 1-((*E*)-1-((*Z*)-4-(ethylidene-1-*d*)-1-tosylpyrrolidin-3-ylidene)ethyl)-7-methyl-2-naphthoate (20-*d*)

The title compound was isolated as a yellow solid (eluent: petroleum ether/ethyl acetate = 4/1, 20.5 mg, 43%). **^{1}H NMR (600 MHz, Chloroform-d) *δ*** 7.88 – 7.84 (m, 1H), 7.83 – 7.78 (m, 2H), 7.78 – 7.74 (m, 2H), 7.54 (s, 1H), 7.43 – 7.37 (m, 3H), 4.41 (d, *J* = 12.1, 1H), 4.10 (d, *J* = 13.2, 1H), 3.99 (d, *J* = 14.7, 1H), 3.78 – 3.71 (m, 4H), 2.50 – 2.45 (m, 6H), 2.05 (s, 3H), 1.15 (s,

3H). **^{13}C NMR (150 MHz, Chloroform-d) *δ*** 167.6, 143.8, 142.8, 137.1, 133.9, 133.2, 133.1, 130.5, 130.3, 129.9, 129.4, 128.3, 128.2, 128.1, 127.2, 125.53, 125.50, 125.2, 120.2, 52.9, 52.1, 51.6, 23.6, 22.2, 21.7, 15.5. **HRMS (ESI):** calcd. for $C_{28}H_{28}DNNaO_4S^+$[M+Na]$^+$: 499.1772; found: 499.1772; **$[\alpha]_D^{20}$** = +42 (c = 0.1, $CHCl_3$).

HPLC analysis: IG column (hexane:2-propanol = 90:10, v = 1.0 mL/min, 40 ^{o}C, 254 nm); tr (minor) =20.500 min, tr (major) =25.320 min, 96% ee.

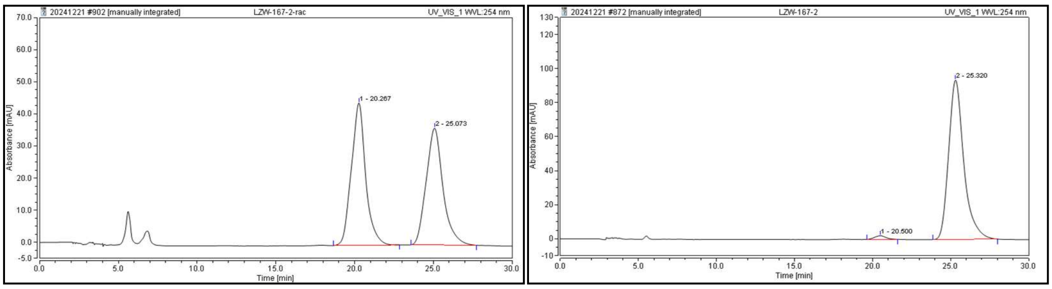

No.	Time	Area	Area (%)	No.	Time	Area	Area (%)
1	20.267	46.527	50.85	1	20.500	1.816	1.70
2	25.073	44.979	49.15	2	25.320	105.245	98.30

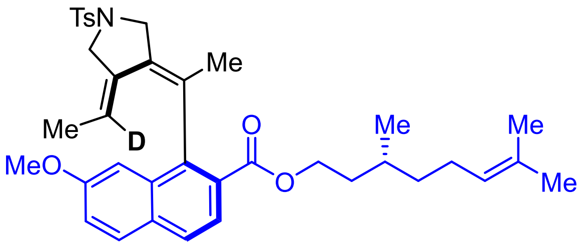

(*R*)-3,7-dimethyloct-6-en-1-yl 1-((*E*)-1-((*Z*)-4-(ethylidene-1-*d*)-1-tosylpyrrolidin-3-ylidene)ethyl)-7-methoxy-2-naphthoate (21-*d*)

The title compound was isolated as a yellow solid (eluent: petroleum ether/ethyl acetate = 4/1, 24.0 mg, 39%). **^{1}H NMR (600 MHz, Chloroform-d) *δ*** 7.81 – 7.71 (m, 5H), 7.40 – 7.35 (m, 2H), 7.25 – 7.21 (m, 1H), 7.10 – 7.06 (m, 1H), 5.12 – 5.06 (m, 1H), 4.39 (d, *J* = 12.1, 1H), 4.27 – 4.15 (m, 2H), 4.08 (d, *J* = 13.0, 1H), 3.96 (d, *J* = 14.7, 1H), 3.80 (s, 3H), 3.74 (d, *J* = 13.2, 1H), 2.46 (s, 3H), 2.05 (s, 3H), 2.03 – 1.90 (m, 1H), 1.73 – 1.65 (m, 4H), 1.62 – 1.59 (m, 3H), 1.59 – 1.54 (m, 1H), 1.52 – 1.40 (m, 1H), 1.39 – 1.30 (m, 2H), 1.17 (s, 3H), 0.91 (d, *J* = 6.6, 3H). **^{13}C NMR (150 MHz, Chloroform-d) *δ*** 167.2, 158.5, 143.6, 141.5, 132.91, 132.88, 131.4, 131.1, 130.9, 129.8, 129.7, 129.3, 128.3, 127.9, 127.0, 126.4, 124.5, 124.1, 120.3, 104.5, 63.5, 55.3, 52.7, 51.4, 36.9, 35.6, 29.6, 25.7, 25.4, 23.2, 21.6, 19.4, 17.7, 15.4. **HRMS (ESI):** calcd. for $C_{37}H_{44}DNNaO_5S^+$[M+Na]$^+$: 639.2973; found: 639.2973; **$[\alpha]_D^{20}$** = +160 (c = 0.1, $CHCl_3$).

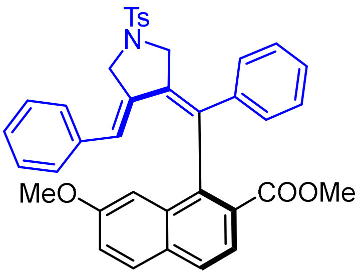

(*R*)-methyl 1-((*E*)-(4-((*Z*)-benzylidene)-1-tosylpyrrolidin-3-ylidene)(phenyl)methyl)-7-methoxy-2-naphthoate (26)

The title compound was isolated as a yellow solid (eluent: petroleum ether/ethyl acetate = 4/1, 44.3 mg, 72%). **^{1}H NMR (600 MHz, Chloroform-d) *δ*** 7.79 – 7.75 (m, 4H), 7.74 – 7.72 (m, 1H), 7.38 – 7.33 (m, 3H), 7.29 – 7.27 (m, 2H), 7.25 – 7.23 (m, 1H), 7.18 (dd, *J* = 8.9, 2.5, 1H), 7.16 – 7.11 (m, 4H), 7.11 – 7.07 (m, 1H), 6.62 (d, *J* = 7.3, 2H), 5.66 (t, *J* = 2.7, 1H), 4.42 (d, *J* = 12.4, 1H), 4.25 (dd, *J* = 13.8, 2.5, 1H), 4.18 (d, *J* = 12.4, 1H), 4.02 (dd, *J* = 13.8, 2.7, 1H), 3.73 (s, 3H), 3.67 (s, 3H), 2.45 (s, 3H). **^{13}C NMR (150 MHz, Chloroform-d) *δ*** 168.0, 159.0, 144.0, 141.2, 139.6, 136.5, 134.6, 134.34, 134.28, 132.8, 132.7, 131.0, 130.0, 129.8, 129.0, 128.8, 128.5, 128.4, 128.3, 128.1, 127.94, 127.91, 127.6, 127.4, 124.1, 121.0, 104.6, 55.4, 52.5, 52.1, 51.1, 21.7. **HRMS (ESI):** calcd. for $C_{38}H_{33}NNaO_5S^+$[M+Na]$^+$: 638.1972; found: 638.1972; **$[\alpha]_D^{20}$** = +72 (c = 0.1, $CHCl_3$).

HPLC analysis: IE column (hexane:2-propanol = 75:25, v = 1.0 mL/min, 40 ^{o}C, 254 nm); tr (minor) =14.086 min, tr (major) =40.502 min, 95% ee.

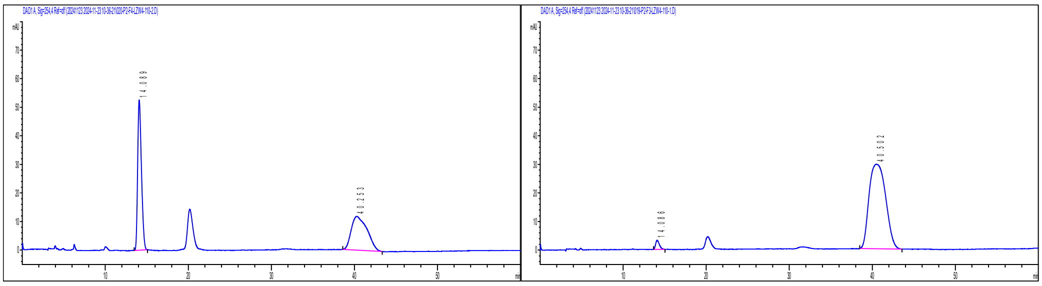

No.	Time	Area	Area (%)	No.	Time	Area	Area (%)
1	14.089	1669.9	51.509	1	14.086	100.8	2.389
2	40.253	1572	48.491	2	40.502	4117.2	97.611

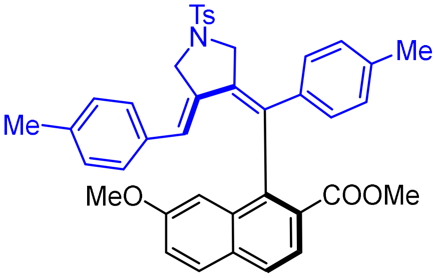

(*R*)-methyl 7-methoxy-1-((*E*)-(4-((*Z*)-4-methylbenzylidene)-1-tosylpyrrolidin-3-ylidene)(p-tolyl)methyl)-2-naphthoate (27)

The title compound was isolated as a white solid (eluent: petroleum ether/ethyl acetate = 4/1, 43.4 mg, 68%).**^{1}H NMR (600 MHz, Chloroform-d) *δ*** 7.77 – 7.74 (m, 4H), 7.73 – 7.70 (m, 1H),

7.38 – 7.32 (m, 3H), 7.17 (dd, *J* = 8.9, 2.5, 1H), 7.08 – 7.04 (m, 2H), 7.03 – 6.97 (m, 2H), 6.96 – 6.93 (m, 2H), 6.52 (d, *J* = 8.3, 2H), 5.61 (t, *J* = 2.7, 1H), 4.41 (d, *J* = 12.3, 1H), 4.24 (dd, *J* = 13.8, 2.4, 1H), 4.17 (d, *J* = 12.3, 1H), 4.01 (dd, *J* = 13.8, 2.6, 1H), 3.73 (s, 3H), 3.66 (s, 3H), 2.44 (s, 3H), 2.32 (s, 3H), 2.23 (s, 3H). **^{13}C NMR (150 MHz, Chloroform-d)** ***δ*** 168.0, 158.8, 143.8, 139.6, 138.4, 137.6, 137.3, 134.1, 133.8, 133.7, 133.3, 132.7, 132.6, 130.9, 129.8, 129.6, 129.0, 128.8, 128.72, 128.65, 128.3, 127.9, 127.7, 127.2, 123.9, 120.8, 104.6, 55.3, 52.4, 52.0, 51.0, 21.5, 21.2, 21.1. **HRMS (ESI):** calcd. for $C_{40}H_{37}NNaO_5S^+$[M+Na]$^+$: 666.2285; found: 666.2288; **[α]$_D$^{20}** = +72 (c = 0.1, $CHCl_3$). **HPLC analysis:** IE column (hexane:2-propanol = 70:30, v = 1.0 mL/min, 40 °C, 254 nm); tr (minor) =14.277 min, tr (major) =45.763 min, 95% ee.

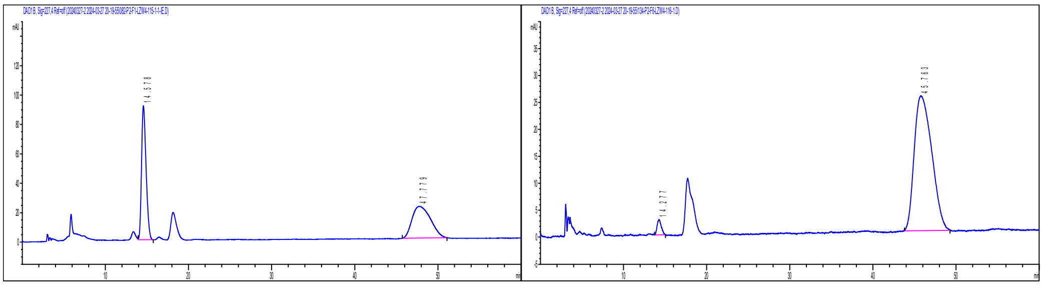

No.	Time	Area	Area (%)	No.	Time	Area	Area (%)
1	14.578	3462.2	50.826	1	14.277	100	2.648
2	47.779	3349.7	49.174	2	45.763	3675.3	97.352

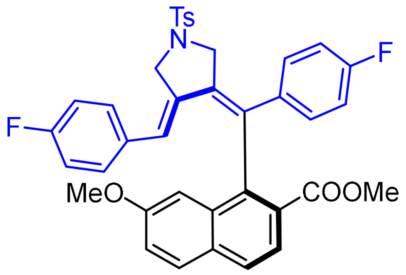

(*R*)-methyl 1-((*E*)-(4-((*Z*)-4-fluorobenzylidene)-1-tosylpyrrolidin-3-ylidene)(4-fluorophenyl)methyl)-7-methoxy-2-naphthoate (28)

The title compound was isolated as a white solid (eluent: petroleum ether/ethyl acetate = 4/1, 37.8 mg, 58%). **^{1}H NMR (600 MHz, Chloroform-d)** ***δ*** 7.81 – 7.77 (m, 1H), 7.77 – 7.73 (m, 4H), 7.37 (d, *J* = 8.0, 2H), 7.30 (d, *J* = 2.7, 1H), 7.20 (dd, *J* = 8.9, 2.5, 1H), 7.12 – 7.07 (m, 2H), 7.00 – 6.94 (m, 2H), 6.88 – 6.79 (m, 2H), 6.62 – 6.54 (m, 2H), 5.59 (t, *J* = 2.7, 1H), 4.38 (d, *J* = 12.3, 1H), 4.21 (dd, *J* = 13.8, 2.5, 1H), 4.13 (d, *J* = 12.3, 1H), 3.96 (dd, *J* = 13.8, 2.8, 1H), 3.74 (s, 3H), 3.67 (s, 3H), 2.45 (s, 3H). **^{13}C NMR (150 MHz, Chloroform-d)** ***δ*** 167.8, 162.1 (d, *J* = 248.2 Hz), 161.8 (d, *J* = 248.9 Hz), 159.0, 144.0, 139.1, 137.1 (d, *J* = 3.2 Hz), 134.1, 133.7, 133.5, 132.46, 132.44 (d, *J* = 3.4 Hz), 132.4, 130.9, 130.6 (d, *J* = 8.1 Hz), 130.0, 129.94, 129.93,

129.7, 129.5, 128.7, 128.0, 127.9, 126.4, 123.9, 121.0, 115.5, 115.31, 115.29, 115.1, 104.3, 55.3, 52.3, 52.1, 50.9, 21.6.[19]**F NMR (376 MHz, Chloroform-d) *δ*** -113.3 (m), -113.1 (m). **HRMS (ESI):** calcd. for $C_{38}H_{31}F_2NNaO_5S^+$[M+Na]$^+$: 674.1783; found: 674.1794; **$[\alpha]_D^{20}$** = +78 (c = 0.1, $CHCl_3$).

HPLC analysis: IE column (hexane:2-propanol = 70:30, v = 1.0 mL/min, 40 °C, 254 nm); tr (minor) =9.656 min, tr (major) =20.662 min, 94% ee.

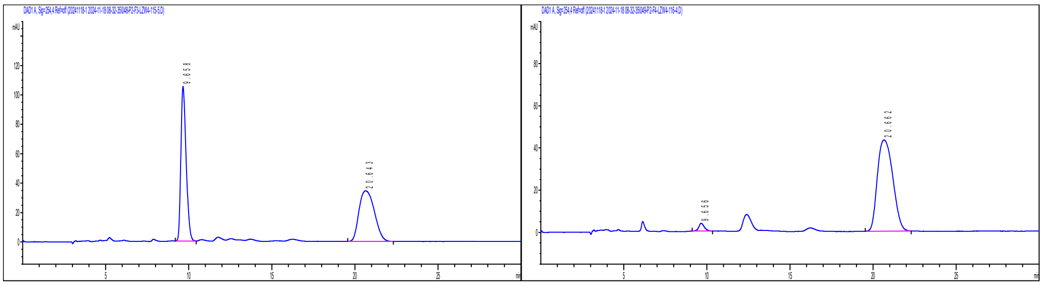

No.	Time	Area	Area (%)	No.	Time	Area	Area (%)
1	9.658	2305.6	50.992	1	9.656	85.6	2.983
2	20.643	2215.9	49.008	2	20.662	2783.2	97.017

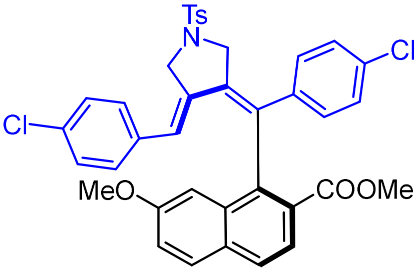

(*R*)-methyl 1-((*E*)-(4-((*Z*)-4-chlorobenzylidene)-1-tosylpyrrolidin-3-ylidene)(4-chlorophenyl)methyl)-7-methoxy-2-naphthoate (29)

The title compound was isolated as a white solid (eluent: petroleum ether/ethyl acetate = 3/1, 29.4 mg, 43%). **^{1}H NMR (600 MHz, Chloroform-d) *δ*** 7.81 – 7.73 (m, 5H), 7.39 – 7.35 (m, 2H), 7.28 – 7.23 (m, 1H), 7.21 (dd, *J* = 9.0, 2.6, 2H), 7.13 – 7.08 (m, 1H), 7.07 – 7.03 (m, 4H), 6.55 – 6.50 (m, 2H), 5.57 (t, *J* = 2.6, 1H), 4.38 (d, *J* = 12.4, 1H), 4.21 (dd, *J* = 13.8, 2.5, 1H), 4.13 (d, *J* = 12.4, 1H), 3.95 (dd, *J* = 13.8, 2.7, 1H), 3.74 (s, 3H), 3.67 (3, 1H), 2.45 (s, 3H).**^{13}C NMR (150 MHz, Chloroform-d) *δ*** 167.8, 159.2, 144.2, 139.5, 139.0, 134.8, 134.7, 134.6, 133.91, 133.86, 133.4, 132.6, 132.5, 131.1, 130.2, 130.1, 129.9, 129.6, 128.73, 128.69, 128.6, 128.2, 128.1, 126.6, 124.0, 121.2, 104.3, 55.4, 52.4, 52.2, 51.0, 21.7. **HRMS (ESI)**: calcd. for $C_{38}H_{31}Cl_2NNaO_5S^+$[M+Na]$^+$: 706.1192; found: 706.1192. **$[\alpha]_D^{20}$** = +80 (c = 0.1, $CHCl_3$).

HPLC analysis: IE column (hexane:2-propanol = 70:30, v = 1.0 mL/min, 40 °C, 254 nm); tr (minor) = 11.576 min, tr (major) = 30.212 min, 93% ee.

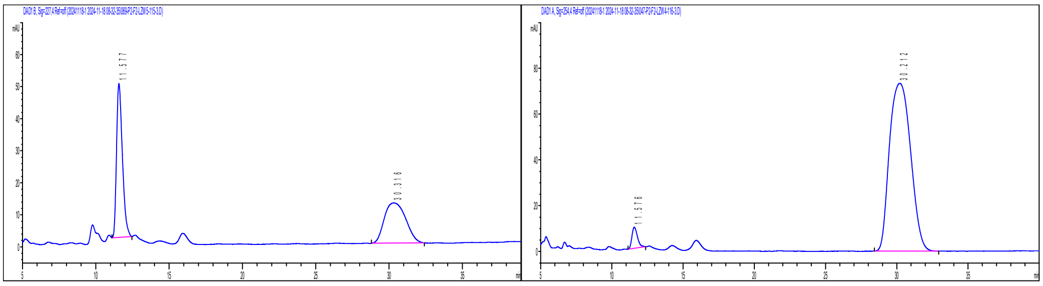

No.	Time	Area	Area (%)	No.	Time	Area	Area (%)
1	11.577	1335.8	51.115	1	11.576	250.6	3.197
2	30.316	1277.6	48.885	2	30.212	7586.6	96.803

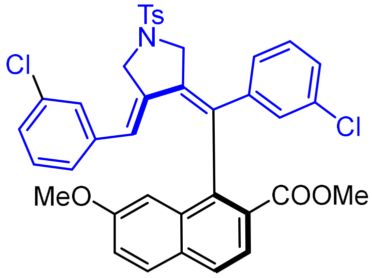

(*R*)-methyl 1-((*E*)-(4-((*Z*)-3-chlorobenzylidene)-1-tosylpyrrolidin-3-ylidene)(3-chlorophenyl)methyl)-7-methoxy-2-naphthoate (30)

The title compound was isolated as a white solid (eluent: petroleum ether/ethyl acetate = 3/1, 44.5 mg, 65%). **^{1}H NMR (600 MHz, Chloroform-d) *δ*** 7.83 – 7.73 (m, 5H), 7.40 – 7.37 (m, 2H), 7.30 – 7.27 (m, 1H), 7.26 – 7.19 (m, 3H), 7.12 – 7.03 (m, 4H), 6.57 – 6.47 (m, 2H), 5.57 (t, *J* = 2.7, 1H), 4.39 (d, *J* = 12.6, 1H), 4.22 (dd, *J* = 13.9, 2.5, 1H), 4.15 (d, *J* = 12.6, 1H), 3.97 (dd, *J* = 13.9, 2.7, 1H), 3.76 (s, 3H), 3.69 (s, 3H), 2.45 (s, 3H).**^{13}C NMR (150 MHz, Chloroform-d) *δ*** 167.8, 159.2, 144.2, 142.8, 138.5, 138.0, 135.4, 135.0, 134.32, 134.28, 134.0, 132.5, 132.4, 131.1, 130.1, 129.9, 129.7, 129.6, 128.8, 128.7, 128.4, 128.3, 128.2, 128.0, 127.6, 127.2, 126.6, 126.3, 124.0, 121.2, 104.2, 55.4, 52.3, 52.3, 50.9, 21.7. **HRMS (ESI)**: calcd. for $C_{38}H_{31}Cl_2NNaO_5S^+$[M+Na]$^+$: 706.1192; found: 706.1197 **[α]$_D$^{20}** = +66 (c = 0.1, $CHCl_3$).

HPLC analysis: IE column (hexane:2-propanol = 85:15, v = 1.0 mL/min, 40 ^{o}C, 254 nm); tr (minor) = 14.258 min, tr (major) = 25.593 min, 93% ee.

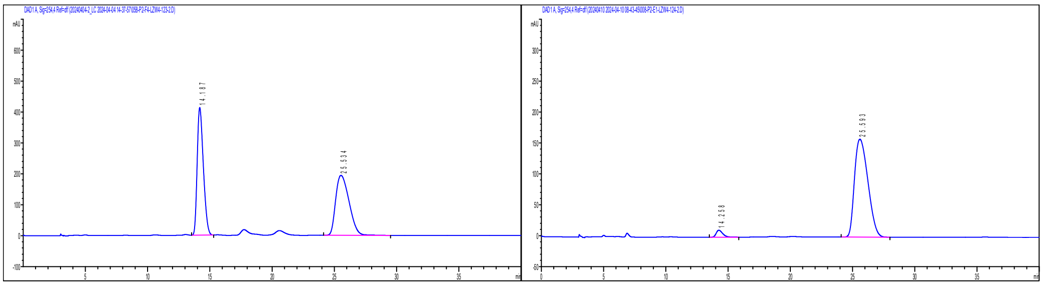

No.	Time	Area	Area (%)	No.	Time	Area	Area (%)
1	14.187	14229.6	49.536	1	14.258	418.6	3.407
2	25.534	14496.2	50.464	2	25.593	11870	96.593

(*R*)-methyl 7-methoxy-1-((*Z*)-thiophen-2-yl((*Z*)-4-(thiophen-2-ylmethylene)-1-tosylpyrrolidin-3-ylidene)methyl)-2-naphthoate (31)

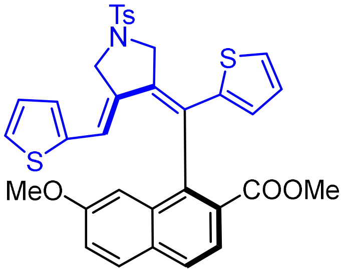

The title compound was isolated as a white solid (eluent: petroleum ether/ethyl acetate = 3/1, 30.1 mg, 48%). **^{1}H NMR (600 MHz, Chloroform-d) *δ*** 7.88 – 7.83 (m, 4H), 7.78 (d, *J* = 8.8, 1H), 7.41 – 7.36 (m, 2H), 7.30 – 7.27 (m, 1H), 7.23 – 7.18 (m, 3H), 6.98 – 6.94 (m, 1H), 6.84 – 6.80 (m, 1H), 6.76 – 6.72 (m, 1H), 6.28 – 6.25 (m, 1H), 5.52 (t, *J* = 2.6, 1H), 4.75 (d, *J* = 13.1, 1H), 4.45 (d, *J* = 13.1, 1H), 4.27 (dd, *J* = 14.0, 2.5, 1H), 4.07 (dd, *J* = 14.0, 2.6, 1H), 3.70 (s, 3H), 3.66 (s, 3H), 2.45 (s, 3H). **^{13}C NMR (150 MHz, Chloroform-d) *δ*** 167.4, 159.1, 144.6, 144.1, 140.5, 138.9, 132.8, 132.6, 132.1, 131.6, 131.1, 130.1, 129.7, 128.5, 128.4, 128.1, 128.0, 127.6, 127.5, 127.3, 127.1, 126.6, 124.3, 121.2, 119.9, 104.5, 55.4, 53.8, 52.24, 52.21, 21.7. **HRMS (ESI):** calcd. for $C_{34}H_{29}NNaO_5S_3^+$[M+Na]$^+$: 650.1100; found: 650.1102; **$[\alpha]_D^{20}$** = +56 (c = 0.1, $CHCl_3$).

HPLC analysis: IE column (hexane:2-propanol = 75:25, v = 1.0 mL/min, 40 °C, 254 nm); tr (minor) = 20.147 min, tr (major) = 41.97 min, 97% ee.

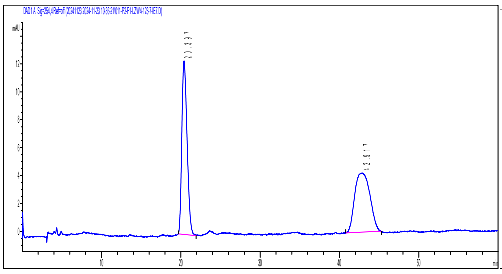

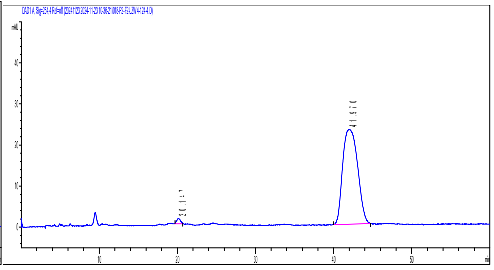

No.	Time	Area	Area (%)	No.	Time	Area	Area (%)
1	20.397	574.5	50.363	1	20.147	43.5	1.394
2	42.917	566.2	49.637	2	41.97	3078.2	98.606

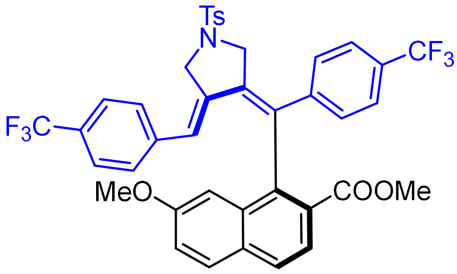

(*R*)-methyl 7-methoxy-1-((*E*)-(1-tosyl-4-((*Z*)-4-(trifluoromethyl)benzylidene)pyrrolidin-3-ylidene)(4-(trifluoromethyl)phenyl)methyl)-2-naphthoate (32)

The title compound was isolated as a white solid (eluent: petroleum ether/ethyl acetate = 3/1, 31.5 mg, 42%). **^{1}H NMR (600 MHz,**

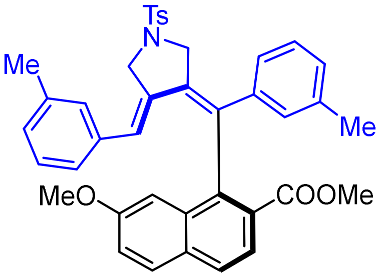

Chloroform-d) δ 7.86 – 7.74 (m, 5H), 7.57 – 7.53 (m, 2H), 7.42 – 7.37 (m, 4H), 7.30 – 7.28 (m, 1H), 7.27 – 7.22 (m, 3H), 6.70 (d, *J* = 8.4, 2H), 5.67 (t, *J* = 2.7, 1H), 4.41 (d, *J* = 12.4, 1H), 4.26 (dd, *J* = 14.0, 2.4, 1H), 4.16 (d, *J* = 12.4, 1H), 3.97 (dd, *J* = 14.0, 2.8, 1H), 3.75 (s, 3H), 3.69 (s, 3H), 2.46 (s, 3H). **^{13}C NMR (150 MHz, Chloroform-d) δ** 167.4, 159.2, 144.3, 144.2, 139.5, 138.6, 136.2, 135.2, 134.4, 132.3, 132.2, 131.0, 130.0, 129.9, 129.1, 128.5, 128.4, 128.0, 126.6, 125.3 (q, *J* = 3.7), 125.2 (q, *J* = 3.7), 124.0 (q, *J* = 270.6), 123.94, 123.85 (q, *J* = 270.2), 121.2 (d, *J* = 4.1), 104.0, 55.3, 52.3, 52.2, 50.9, 21.6. **^{19}F NMR (376 MHz, Chloroform-d)** *δ* -62.5 (m), -62.6 (m).

HRMS (ESI): calcd. for $C_{40}H_{31}F_6NNaO_5S^+$[M+Na]$^+$: 774.1719; found: 774.1726; **$[\alpha]_D^{20}$** = +70 (c = 0.1, $CHCl_3$).

HPLC analysis: IE column (hexane:2-propanol = 85:15, v = 1.0 mL/min, 40 °C, 254 nm); tr (minor) = 7.463 min, tr (major) = 13.895 min, 95% ee.

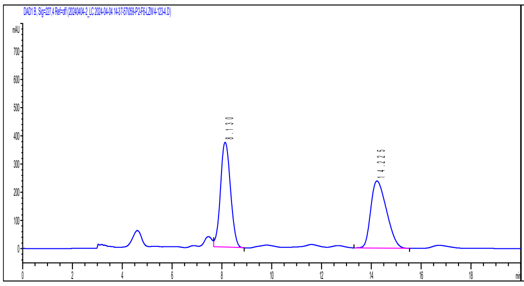

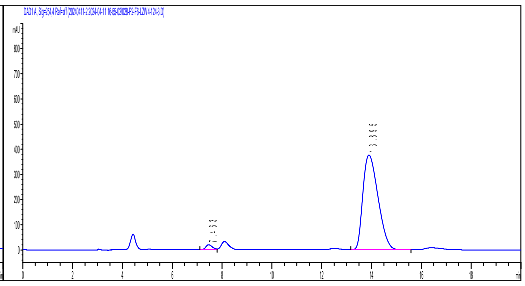

No.	Time	Area	Area (%)	No.	Time	Area	Area (%)
1	8.13	10405.7	49.848	1	7.463	365.5	2.249
2	14.225	10469.2	50.152	2	13.895	15884.9	97.751

(*R*)-methyl 7-methoxy-1-((*E*)-(4-((*Z*)-3-methylbenzylidene)-1-tosylpyrrolidin-3-ylidene)(m-tolyl)methyl)-2-naphthoate (33)

The title compound was isolated as a white solid (eluent: petroleum ether/ethyl acetate = 3/1, 32.1 mg, 50%).**^{1}H NMR (600 MHz, Chloroform-d) δ** 7.77 – 7.74 (m, 4H), 7.73 – 7.70 (m, 1H), 7.38 – 7.32 (m, 3H), 7.20 – 7.13 (m, 2H), 7.06 – 7.01 (m, 2H), 6.99 – 6.94 (m, 1H), 6.92 – 6.88 (m, 2H), 6.46 (d, *J* = 8.0, 1H), 6.40 (s, 1H), 5.64 (t, *J* = 2.6,

1H), 4.41 (d, *J* = 12.4, 1H), 4.24 (dd, *J* = 13.8, 2.4, 1H), 4.17 (d, *J* = 12.4, 1H), 4.02 (dd, *J* = 13.8, 2.6, 1H), 3.74 (s, 3H), 3.67 (s, 3H), 2.43 (s, 3H), 2.25 (s, 3H), 2.19 (s, 3H). **^{13}C NMR (150 MHz, Chloroform-d)** ***δ*** 168.2, 158.9, 143.9, 141.2, 139.5, 138.0, 137.7, 136.5, 134.6, 134.3, 134.1, 132.9, 132.7, 131.0, 130.0, 129.7, 129.4, 129.2, 128.8, 128.7, 128.3, 128.2, 128.1, 128.0, 127.8, 127.7, 126.2, 125.4, 124.0, 120.9, 104.7, 55.4, 52.4, 52.1, 51.1, 21.6, 21.5. **HRMS (ESI):** calcd. for $C_{40}H_{37}NNaO_5S^+$[M+Na]$^+$: 666.2285; found: 666.2295; **$[\alpha]_D^{20}$** = +48 (c = 0.1, $CHCl_3$).

HPLC analysis: IE column (hexane:2-propanol = 70:30, v = 1.0 mL/min, 40 °C, 254 nm); tr (minor) = 11.471 min, tr (major) = 23.258 min, 96% ee.

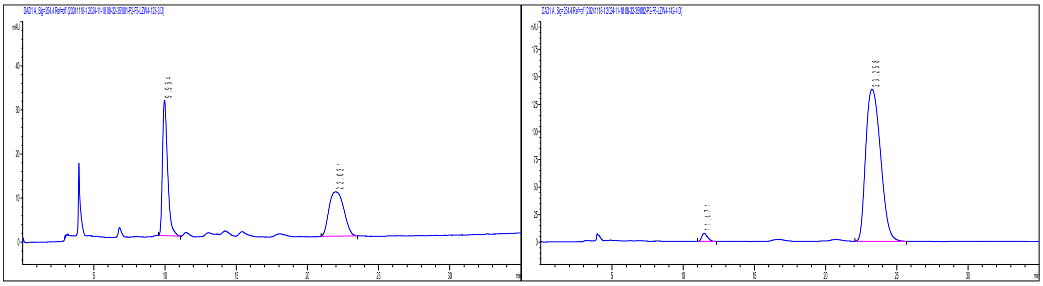

No.	Time	Area	Area (%)	No.	Time	Area	Area (%)
1	9.964	765.4	51.317	1	11.471	204	2.008
2	22.021	726.1	48.683	2	23.258	9952.9	97.992

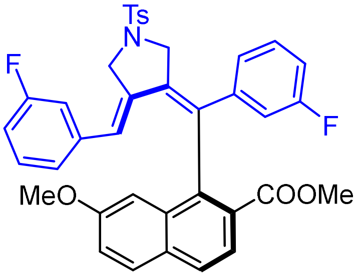

(*R*)-methyl 1-((*E*)-(4-((*Z*)-3-fluorobenzylidene)-1-tosylpyrrolidin-3-ylidene)(3-fluorophenyl)methyl)-7-methoxy-2-naphthoate (34)

The title compound was isolated as a white solid (eluent: petroleum ether/ethyl acetate = 4/1, 30.0 mg, 46%). **^{1}H NMR (600 MHz, Chloroform-d)** ***δ*** 7.83 – 7.74 (m, 5H), 7.38 (d, *J* = 8.4, 2H), 7.30 – 7.28 (m, 1H), 7.27 – 7.23 (m, 1H), 7.23 – 7.19 (m, 1H), 7.13 – 7.07 (m, 1H), 6.99 – 6.93 (m, 2H), 6.84 – 6.76 (m, 2H), 6.38 (d, *J* = 7.8, 1H), 6.29 (d, *J* = 10.0, 1H), 5.59 (t, *J* = 2.7, 1H), 4.42 (d, *J* = 12.5, 1H), 4.23 (dd, *J* = 13.9, 2.5, 1H), 4.16 (d, *J* = 12.4, 1H), 3.98 (dd, *J* = 13.9, 2.8, 1H), 3.75 (s, 3H), 3.69 (s, 3H), 2.45 (s, 3H). **^{13}C NMR (150 MHz, Chloroform-d)** ***δ*** 167.7, 162.5 (d, *J* = 246.1 Hz), 159.1, 144.1, 143.1 (d, *J* = 7.3 Hz), 138.6, 138.3 (d, *J* = 7.7 Hz), 135.2, 134.8, 134.0 (d, *J* = 2.0 Hz), 132.4, 132.3, 131.0, 130.0, 129.9, 129.8, 129.7 (d, *J* = 8.3 Hz), 128.6, 128.2, 128.0, 126.7 (d, *J* = 2.6 Hz), 124.6 (d, *J* = 2.8 Hz), 124.1 (d, *J* = 2.8 Hz), 123.9, 121.1, 115.7 (d, *J* = 22.0 Hz), 114.92 (d, *J* = 21.1 Hz), 114.90

(d, J = 21.5 Hz), 114.4 (d, J = 21.3 Hz), 104.2, 55.3, 52.3, 52.1, 50.9, 21.6. **^{19}F NMR (376 MHz, Chloroform-d) *δ*** -112.70 -112.6 (m, 2F). **HRMS (ESI):** calcd. for $C_{38}H_{31}F_2NNaO_5S^+$[M+Na]$^+$: 674.1783; found: 674.1792; **$[\alpha]_D^{20}$** = +94 (c = 0.1, $CHCl_3$).

HPLC analysis: IE column (hexane:2-propanol = 85:15, v = 1.0 mL/min, 40 ^{o}C, 254 nm); tr (minor) = 14.622 min, tr (major) = 25.939 min, 94% ee.

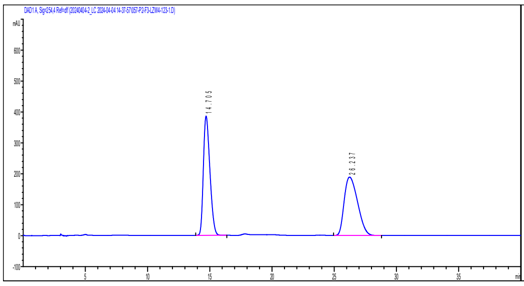
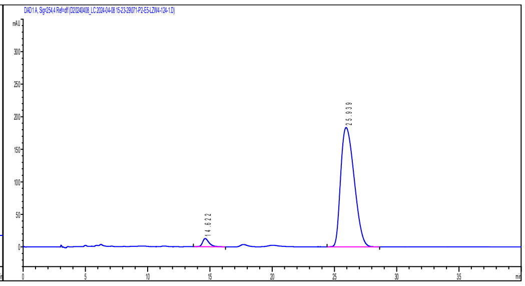

No.	Time	Area	Area (%)	No.	Time	Area	Area (%)
1	14.705	14060.9	49.941	1	14.622	454	3.183
2	26.237	14094.2	50.059	2	25.939	13812.2	96.817

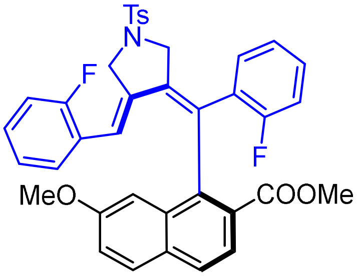

(*R*)-methyl 1-((*Z*)-(4-((*Z*)-2-fluorobenzylidene)-1-tosylpyrrolidin-3-ylidene)(2-fluorophenyl)methyl)-7-methoxy-2-naphthoate (35)

The title compound was isolated as a white solid (eluent: petroleum ether/ethyl acetate = 4/1, 27.3 mg, 42%).**^{1}H NMR (600 MHz, Chloroform-d) *δ*** 7.69 – 7.66 (m, 2H), 7.66 – 7.62 (m, 3H), 7.29 – 7.23 (m, 3H), 7.20 – 7.14 (m, 1H), 7.12 – 7.05 (m, 2H), 7.03 – 6.99 (m, 1H), 6.98 – 6.94 (m, 1H), 6.92 – 6.84 (m, 2H), 6.75 – 6.68 (m, 2H), 5.66 (t, J = 2.7, 1H), 4.19 – 4.13 (m, 1H), 4.07 – 4.01 (m, 1H), 3.98 – 3.90 (m, 2H), 3.71 (s, 3H), 3.66 (s, 3H), 2.35 (s, 3H).**^{13}C NMR (150 MHz, Chloroform-d) *δ*** 168.2, 159.60 (d, J = 250.2 Hz), 159.57 (d, J = 247.7 Hz), 158.9, 143.9, 138.0, 137.1, 135.6, 132.9, 132.4, 130.9, 130.5 (d, J = 2.3 Hz), 129.9, 129.8, 129.7, 129.2 (d, J = 8.3 Hz), 128.9 (d, J = 2.7 Hz), 128.5, 128.2, 128.11, 128.06, 127.6, 124.3 (d, J = 3.6 Hz), 124.2, 124.1, 123.9 (d, J = 3.7 Hz), 121.0, 119.9 (d, J = 5.1 Hz), 115.9 (d, J = 23.3 Hz), 115.4 (d, J = 22.0 Hz), 104.0, 55.3, 52.5 (d, J = 11.4 Hz), 52.2, 51.5 (d, J = 3.4 Hz), 21.5. **^{19}F NMR (376 MHz, Chloroform-d) *δ*** -112.2 (m), -114.7 (m). **HRMS (ESI):** calcd. for $C_{38}H_{31}F_2NNaO_5S^+$[M+Na]$^+$: 674.1783; found:674.1789; **$[\alpha]_D^{20}$** = +46 (c = 0.1, $CHCl_3$).

HPLC analysis: IE column (hexane:2-propanol = 80:20, v = 1.0 mL/min, 40 ^{o}C, 254 nm);

tr (minor) = 14.559 min, tr (major)= 30.199 min, 96% ee.

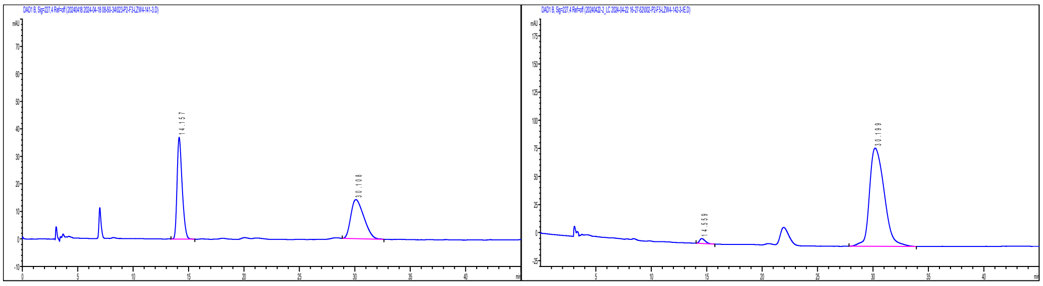

No.	Time	Area	Area (%)	No.	Time	Area	Area (%)
1	14.157	1182.1	50.48	1	14.559	149.7	1.824
2	30.108	1159.6	49.52	2	30.199	8057.4	98.176

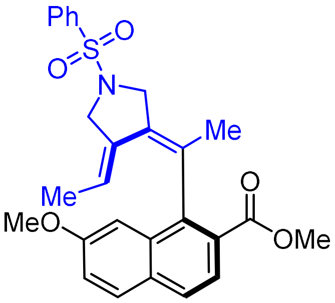

(*R*)-methyl 1-((*E*)-1-((*Z*)-4-ethylidene-1-(phenylsulfonyl)pyrrolidin-3-ylidene)ethyl)-7-methoxy-2-naphthoate (36)

The title compound was isolated as a white solid (eluent: petroleum ether/ethyl acetate = 4/1, 32.5 mg, 68%).**^{1}H NMR (600 MHz, Chloroform-d) *δ*** 7.94 – 7.90 (m, 2H), 7.82 – 7.72 (m, 3H), 7.67 – 7.63 (m, 1H), 7.62 – 7.57 (m, 2H), 7.25 – 7.21 (m, 1H), 7.09 – 7.06 (m, 1H), 4.41 (d, *J* = 14.7, 1H), 4.20 (q, *J* = 7.1, 1H), 4.13 (d, *J* = 13.3, 1H), 4.02 – 3.96 (m, 1H), 3.82 – 3.77 (m, 4H), 3.75 (s, 3H), 2.06 (s, 3H), 1.17 (d, *J* = 7.2, 3H). **^{13}C NMR (150 MHz, Chloroform-d) *δ*** 167.6, 158.7, 141.9, 136.1, 133.0, 132.9, 131.3, 131.1, 129.8, 129.4, 129.3, 128.5, 128.0, 127.2, 126.0, 124.2, 120.6, 120.5, 104.6, 55.4, 52.9, 52.1, 51.5, 23.2, 15.5. **HRMS (ESI):** calcd. for $C_{27}H_{27}NNaO_5S^+$[M+Na]$^+$: 500.1502; found: 500.1502; **[α]$_D$^{20}** = +136 (c = 0.1, $CHCl_3$).

HPLC analysis: IC column (hexane:2-propanol = 80:20, v = 1.0 mL/min, 40 ^{o}C, 254 nm); tr (major) =22.973 min, tr (minor) =25.502 min, 95% ee.

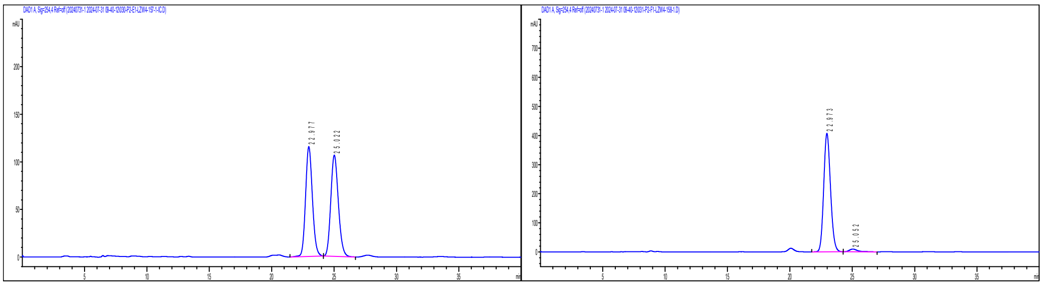

No.	Time	Area	Area (%)	No.	Time	Area	Area (%)
1	22.977	4407.8	49.534	1	22.973	15419.4	97.227
2	25.022	4490.7	50.466	2	25.052	439.8	2.773

(*R*)-methyl 1-((*E*)-1-((*Z*)-4-ethylidene-1-((4-(trifluoromethoxy)phenyl)sulfonyl)pyrrolidin-3-ylidene)ethyl)-7-methoxy-2-naphthoate (37)

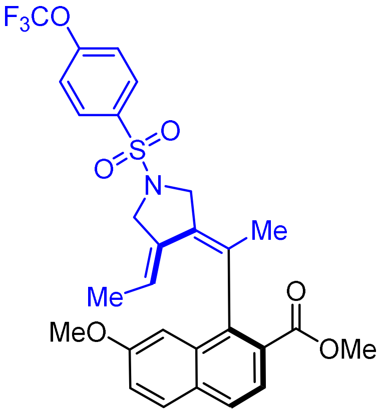

The title compound was isolated as a white solid (eluent: petroleum ether/ethyl acetate = 4/1, 30.3 mg, 54%).**^{1}H NMR (600 MHz, Chloroform-d) *δ*** 8.00 – 7.94 (m, 2H), 7.84 – 7.80 (m, 1H), 7.79 – 7.72 (m, 2H), 7.44 – 7.40 (m, 2H), 7.27 – 7.22 (m, 1H), 7.11 – 7.07 (m, 1H), 4.42 (d, *J* = 13.4, 1H), 4.21 (q, *J* = 7.1, 1H), 4.16 (d, *J* = 14.9, 1H), 4.01 (d, *J* = 14.1, 1H), 3.82 (s, 3H), 3.80 (d, *J* = 13.1, 1H), 3.77 (s, 3H), 2.07 (s, 3H), 1.18 (d, *J* = 7.2, 3H). **^{13}C NMR (150 MHz, Chloroform-d) *δ*** 167.3, 158.6, 152.4 (d, *J* = 1.8 Hz), 141.8, 134.6, 132.5, 131.1, 131.0, 129.9, 129.7, 129.0, 128.7, 127.2, 125.8, 124.1, 121.1, 120.6, 120.30, 120.27 (q, *J* = 259.6 Hz), 104.7, 55.3, 52.8, 52.0, 51.4, 23.0, 15.4. **^{19}F NMR (376 MHz, Chloroform-d) *δ*** -57.6(m). **HRMS (ESI):** calcd. for $C_{28}H_{26}F_3NNaO_6S^+$[M+Na]$^+$: 584.1325; found: 584.1334; **$[\alpha]_D^{20}$** = +162 (c = 0.1, $CHCl_3$).

HPLC analysis: IG column (hexane:2-propanol = 97:3, v = 1.0 mL/min, 40 ^{o}C, 254 nm); tr (minor) =24.497 min, tr (major) =27.060 min, 94% ee.

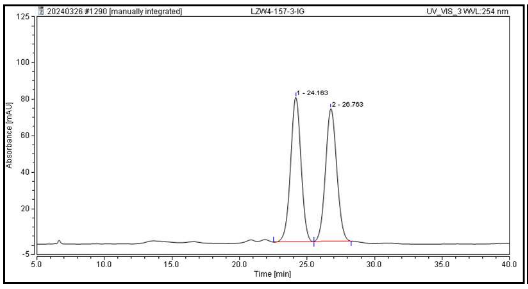

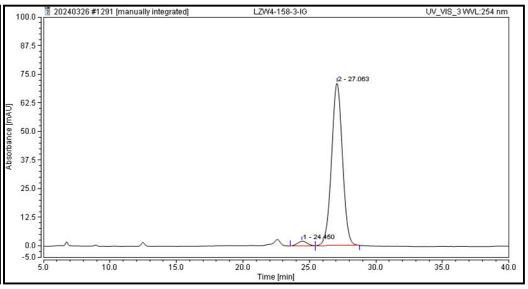

No.	Time	Area	Area (%)	No.	Time	Area	Area (%)
1	24.163	84.390	50.45	1	24.497	1.780	2.67
2	26.760	82.894	49.55	2	27.060	79.864	97.33

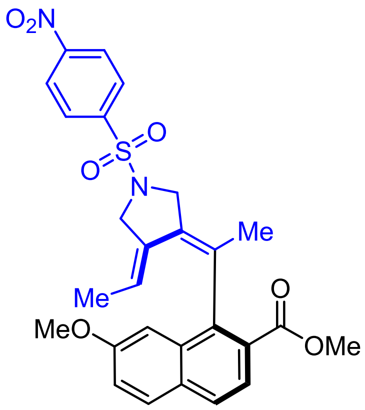

(*R*)-methyl 1-((*E*)-1-((*Z*)-4-ethylidene-1-((4-nitrophenyl)sulfonyl)pyrrolidin-3-ylidene)ethyl)-7-methoxy-2-naphthoate (38)

The title compound was isolated as a white solid (eluent: petroleum ether/ethyl acetate = 4/1, 32.9 mg, 63%). **^{1}H NMR (600 MHz, Chloroform-d) *δ*** 8.46 – 8.40 (m, 2H), 8.12 – 8.07 (m, 2H), 7.84 – 7.72 (m, 3H), 7.28 – 7.23 (m, 1H), 7.09 – 7.05 (m, 1H), 4.46 (d, *J* = 14.9, 1H), 4.25 (d, *J* = 13.7, 1H), 4.20 (q, *J* = 7.1, 1H), 4.04 (d, *J* = 13.7, 1H), 3.86 (d, *J* = 14.1, 1H), 3.83 (s, 3H), 3.79 (s, 3H), 2.07 (s, 3H), 1.17 (d, *J* = 7.2, 3H). **^{13}C NMR (150 MHz, Chloroform-d) *δ*** 167.2, 158.7, 150.4, 142.5, 141.8, 132.4, 131.2, 131.1, 129.9, 129.2, 128.9, 128.6, 127.3, 125.7, 124.6, 124.2, 120.8, 120.1, 105.1, 55.5, 53.0, 52.1, 51.6, 23.2, 15.6. **HRMS (ESI):** calcd. for $C_{27}H_{26}N_2NaO_7S^+$[M+Na]$^+$: 545.1353; found: 545.1363; **$[\alpha]_D^{20}$** = +120 (c = 0.1, $CHCl_3$).

HPLC analysis: IC column (hexane:2-propanol = 80:20, v = 1.0 mL/min, 40 ^{o}C, 254 nm); tr (major) = 32.225 min, tr (minor) = 35.554 min, 96% ee.

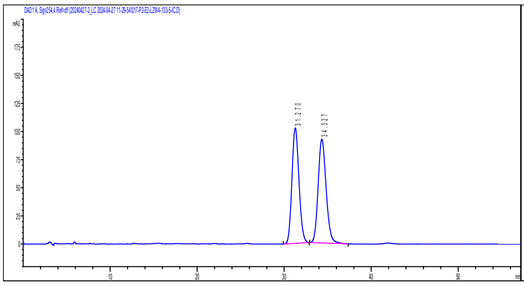

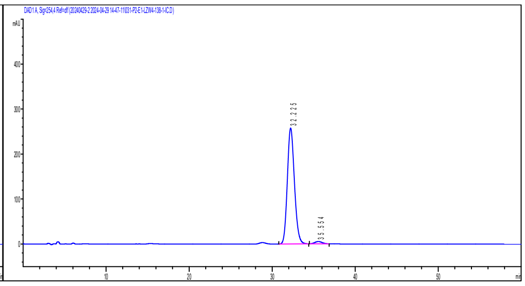

No.	Time	Area	Area (%)	No.	Time	Area	Area (%)
1	31.27	5773.6	49.601	1	32.225	15298.7	98.042
2	34.327	5866.5	50.399	2	35.554	305.5	1.958

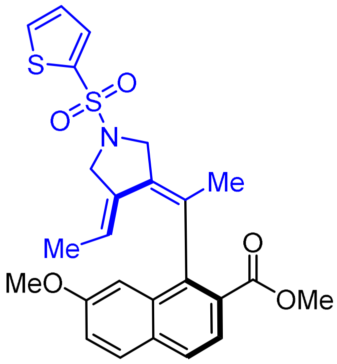

(*R*)-methyl 1-((*E*)-1-((*Z*)-4-ethylidene-1-(thiophen-2-ylsulfonyl)pyrrolidin-3-ylidene)ethyl)-7-methoxy-2-naphthoate (39)

The title compound was isolated as a white solid (eluent: petroleum ether/ethyl acetate = 5/1, 24.7 mg, 51%).**^{1}H NMR (600 MHz, Chloroform-d) *δ*** 7.82 – 7.80 (m, 1H), 7.78 – 7.75 (m, 1H), 7.75 – 7.73 (m, 1H), 7.69 – 7.67 (m, 1H), 7.66 – 7.64 (m, 1H), 7.25 – 7.22 (m, 1H), 7.21 – 7.19 (m, 1H), 7.09 – 7.08 (m, 1H), 4.43 (d, *J* = 12.1, 1H), 4.25 – 4.19 (m, 2H), 4.04 – 3.99 (m, 1H), 3.89 – 3.84 (m, 1H), 3.81 (s, 3H), 3.78 (s, 3H), 2.07 (s, 3H), 1.19 (d, *J* = 7.2, 3H).**^{13}C NMR (150 MHz, Chloroform-d) *δ*** 167.4, 158.5, 141.7, 135.8, 132.62, 132.60, 132.1, 131.1, 131.0, 129.7, 129.1, 128.5, 127.7, 127.1, 125.9, 124.1, 120.6, 120.4, 104.6, 55.3, 53.0, 52.0, 51.6, 23.1, 15.4. **HRMS (ESI):** calcd. for $C_{25}H_{25}NNaO_5S_2^+$[M+Na]$^+$: 506.1066; found: 506.1066; **$[\alpha]_D^{20}$** = +144 (c = 0.1, $CHCl_3$).

HPLC analysis: IC column (hexane:2-propanol = 80:20, v = 1.0 mL/min, 40 ^{o}C, 254 nm); tr (major) = 22.973 min, tr (minor) = 25.052 min, 94% ee.

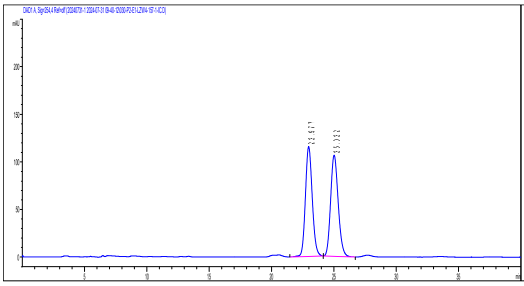

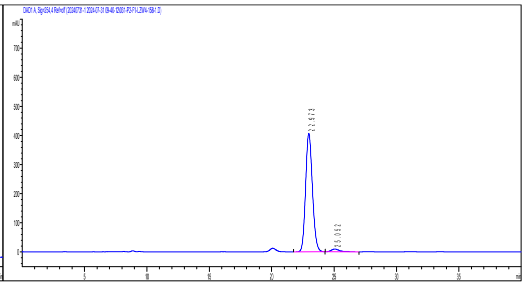

No.	Time	Area	Area (%)	No.	Time	Area	Area (%)
1	22.977	4407.8	49.534	1	22.973	15419.4	97.227
2	25.022	4490.7	50.466	2	25.052	439.8	2.773

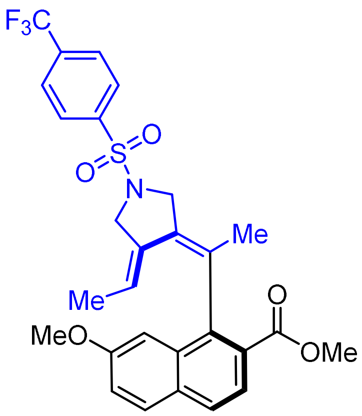

(*R*)-methyl 1-((*E*)-1-((*Z*)-4-ethylidene-1-((4-(trifluoromethyl)phenyl)sulfonyl)pyrrolidin-3-ylidene)ethyl)-7-methoxy-2-naphthoate (40)

The title compound was isolated as a white solid (eluent: petroleum ether/ethyl acetate = 5/1,28.4 mg, 52%).**^{1}H NMR (600 MHz, Chloroform-d) *δ*** = 8.07 – 8.02 (m, 2H), 7.89 – 7.86 (m, 2H), 7.83 – 7.80 (m, 1H), 7.79 – 7.72 (m, 2H), 7.27 – 7.22 (m, 1H), 7.09 – 7.07 (m, 1H), 4.43 (d, *J* = 14.9, 1H), 4.23 – 4.16 (m, 2H), 4.02 (d, *J* = 14.4, 1H), 3.81 (s, 4H), 3.76 (s, 3H), 2.07 (s, 3H), 1.17 (d, *J* = 6.3, 3H). **^{13}C NMR (150 MHz, Chloroform-d) *δ*** 167.2, 158.6, 141.7, 139.9, 134.4 (q, *J* = 33.5 Hz), 132.4, 131.1, 131.0, 129.8, 128.82, 128.79, 128.2, 127.2, 126.4 (q, *J* = 3.8 Hz), 125.7, 124.2 (q, *J* = 271.4 Hz), 124.1, 120.7, 120.3, 104.7, 55.3, 52.8, 52.0, 51.5, 23.1, 15.4. **^{19}F NMR (376 MHz, Chloroform-d) *δ*** -62.9 (m). **HRMS (ESI)**: calcd. for $C_{28}H_{26}F_3NNaO_5S^+$[M+Na]$^+$: 568.1376; found: 568.1369; **$[\alpha]_D^{20}$** = +94 (c = 0.1, $CHCl_3$).

HPLC analysis: IG column (hexane:2-propanol = 95:5, v = 1.0 mL/min, 40 °C, 254 nm); tr (minor) =14.920 min, tr (major) =16.400 min, 96% ee.

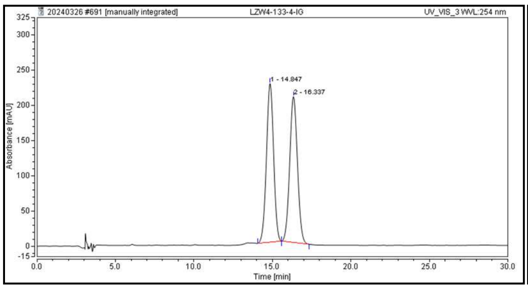

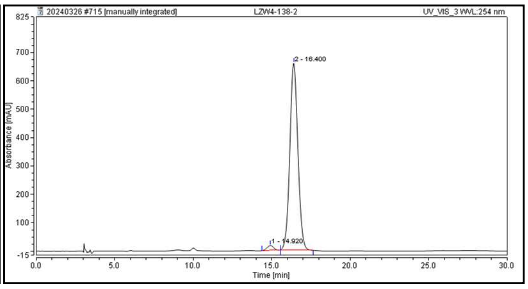

No.	Time	Area	Area (%)	No.	Time	Area	Area (%)
1	14.847	119.384	50.10	1	14.920	7.790	1.95
2	16.337	118.908	49.90	2	16.400	391.612	98.05

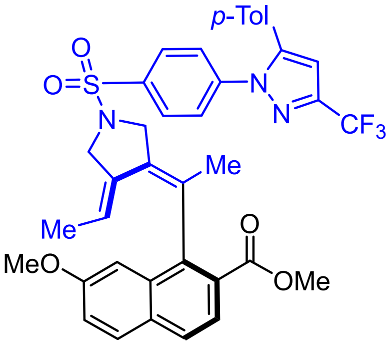

(*R*)-methyl 1-((*E*)-1-((*Z*)-4-ethylidene-1-((4-(5-(p-tolyl)-3-(trifluoromethyl)-1H-pyrazol-1-yl)phenyl)sulfonyl)pyrrolidin-3-ylidene)ethyl)-7-methoxy-2-naphthoate (41)

The title compound was isolated as a white solid (eluent: petroleum ether/ethyl acetate = 3/1, 48.8 mg, 71%). **^{1}H NMR (600 MHz, Chloroform-d) *δ*** 7.92 – 7.86 (m, 2H), 7.83 – 7.79 (m, 1H), 7.78 – 7.72 (m, 2H), 7.57 – 7.51 (m, 2H), 7.25 – 7.20 (m, 1H), 7.19 – 7.14 (m, 2H), 7.13 – 7.10 (m, 2H), 7.08 – 7.04 (m, 1H), 6.77 – 6.74 (m, 1H), 4.37 (d, *J* = 12.1, 1H), 4.22 – 4.14 (m, 2H), 3.95 (d, *J* = 14.1, 1H), 3.86 – 3.75 (m, 7H), 2.37 (s, 3H), 2.06 (s, 3H), 1.16 (d, *J* = 7.2, 3H). **^{13}C NMR (150 MHz, Chloroform-d) *δ*** 167.3, 158.6, 145.3, 144.3, 144.0, 142.8, 141.7, 139.8, 135.6, 132.5, 131.2, 130.9, 129.8, 129.7, 128.9, 128.73, 128.71, 127.1, 125.9, 125.7, 125.6, 124.1, 122.0 (q, *J* = 267.3 Hz), 120.5, 120.2, 106.3, 104.7, 55.3, 52.8, 52.1, 51.5, 23.0, 21.3, 15.4. **^{19}F NMR (376 MHz, Chloroform-d) *δ*** -62.3 (m). **HRMS (ESI)**: calcd. for $C_{38}H_{34}F_3N_3NaO_5S^+$[M+Na]$^+$: 724.2063; found: 724.2067; **$[\alpha]_D^{20}$** = +44 (c = 0.1, $CHCl_3$). **HPLC analysis:** IG column (hexane:2-propanol = 90:10, v = 1.0 mL/min, 40 °C, 254 nm); tr (minor) =12.623min, tr (major) =14.517 min, 96% ee.

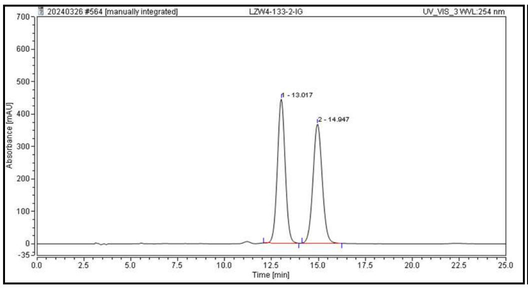
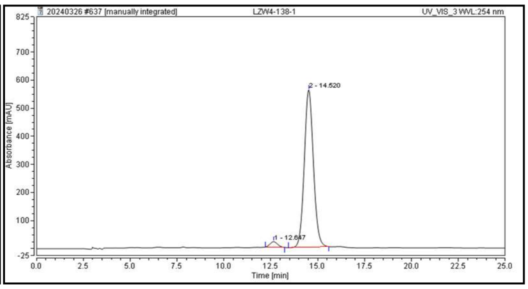

No.	Time	Area	Area (%)	No.	Time	Area	Area (%)
1	13.020	5.744	50.79	1	12.623	0.176	2.05
2	14.950	5.564	49.21	2	14.517	8.398	97.95

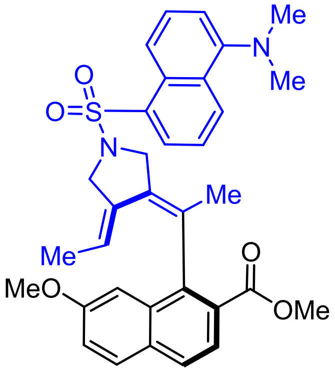

(*R*)-methyl 1-((*E*)-1-((*Z*)-1-((5-(dimethylamino)naphthalen-1-yl)sulfonyl)-4-ethylidenepyrrolidin-3-ylidene)ethyl)-7-methoxy-2-naphthoate (42)

The title compound was isolated as a white solid (eluent: petroleum ether/ethyl acetate = 5/1, 35.9 mg, 63%).**^{1}H NMR (600 MHz, Chloroform-d) *δ*** 8.61 – 8.54 (m, 2H), 8.28 – 8.23 (m, 1H), 7.82 – 7.78 (m, 1H), 7.77 – 7.70 (m, 2H), 7.58 (t, *J* = 8.1, 2H), 7.24 – 7.17 (m, 2H), 7.13 – 7.09 (m, 1H), 4.53 (d, *J* = 13.4, 1H), 4.31 (d, *J* = 13.5, 1H), 4.23 (q, *J* = 7.0, 1H), 4.09 (d, *J* = 13.4, 1H), 3.96 (d, *J* = 13.3, 1H), 3.78 (s, 3H), 3.75 (s, 3H), 2.90 (s, 6H), 2.04 (s, 3H), 1.15 (d, *J* = 7.2, 3H). **^{13}C NMR (150 MHz, Chloroform-d) *δ*** 167.6, 158.7, 151.9, 142.0, 133.6, 133.3, 131.3, 131.1, 130.8, 130.7, 130.3, 129.9, 129.8, 128.3, 128.2, 127.2, 126.1, 124.2, 123.4, 120.6, 120.4, 120.0, 115.4, 104.6, 55.4, 52.5, 52.1, 51.3, 45.6, 23.2, 15.5**. HRMS (ESI)**: calcd. for $C_{33}H_{34}N_2NaO_5S^+$[M+Na]$^+$: 593.2081; found: 593.2080; **[α]$_D$^{20}** = +70 (c = 0.1, $CHCl_3$).

HPLC analysis: ID column (hexane:2-propanol = 95:5, v = 1.0 mL/min, 40 ^{o}C, 227 nm); tr (minor) =40.683 min, tr (major) =44.237 min, 94% ee.

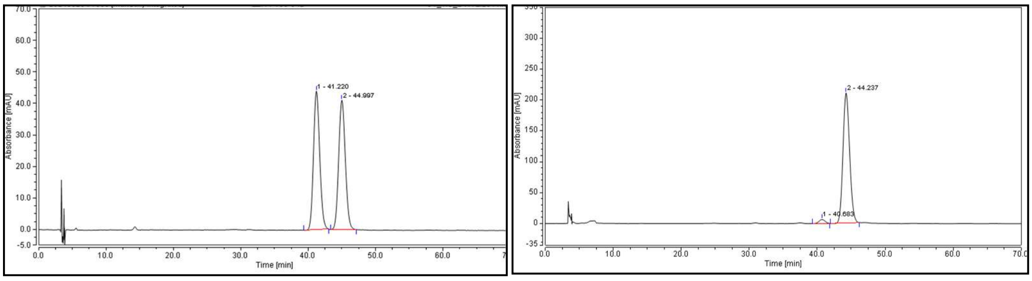

No.	Time	Area	Area (%)	No.	Time	Area	Area (%)
1	41.220	46.837	49.90	1	40.683	6.688	2.73
2	44.997	47.019	50.10	2	44.237	238.245	97.27

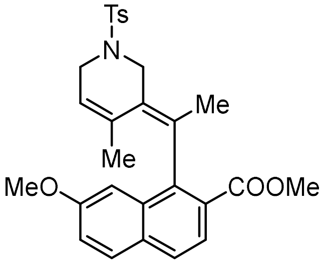

methyl (*E*)-7-methoxy-1-(1-(4-methyl-1-tosyl-1,6-dihydropyridin-3(2H)-ylidene)ethyl)-2-naphthoate (3'). **^{1}H NMR (600 MHz, DMSO-*d*$_6$) *δ*** 7.78 – 7.72 (m, 2H), 7.48 – 7.43 (m, 2H), 7.15 – 7.10 (m, 1H), 6.81 – 6.76 (m, 1H), 6.53 – 6.47 (m, 1H), 6.14 – 6.10 (m, 1H), 5.89 – 5.84 (m, 1H), 4.20 (d, *J* = 13.1, 1H), 3.94 – 3.86 (m, 2H), 3.79 (t, *J* = 2.7, 1H), 3.74 (d, *J* = 15.0, 1H),

3.64 (s, 3H), 3.10 (s, 3H), 2.39 (s, 3H), 1.76 (s, 3H), 1.49 (s, 3H). **^{13}C NMR (150 MHz, Chloroform-d) *δ*** 172.1, 160.0, 143.6, 140.7, 139.5, 137.2, 136.6, 134.8, 133.6, 129.8, 128.3, 127.6, 127.4, 126.4, 122.5, 111.7, 111.3, 55.2, 51.3, 46.4, 46.1, 21.5, 13.1, 12.0. **HRMS (ESI)**: calcd. for $C_{28}H_{29}NNaO_5S^+$ [M+Na]$^+$: 514.1659; found: 514.1662;

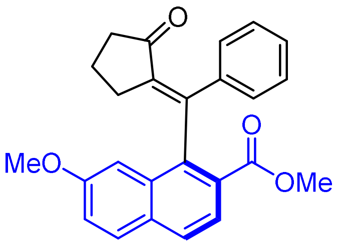

(*R*)-methyl (*E*)-7-methoxy-1-((2-oxocyclopentylidene)(phenyl)methyl)-2-naphthoate (44).

The title compound was isolated as a red oil liquid (eluent: petroleum ether/ethyl acetate = 4/1, 30.1 mg, 78%).**^{1}H NMR (600 MHz, Chloroform-d) *δ*** 7.83 – 7.77 (m, 3H), 7.37 – 7.32 (m, 3H), 7.27 – 7.16 (m, 4H), 3.80 – 3.77 (m, 6H), 2.54 – 2.35 (m, 3H), 2.23 – 2.15 (m, 1H), 1.94 – 1.84 (m, 1H), 1.84 – 1.76 (m, 1H). **^{13}C NMR (150 MHz, Chloroform-d) *δ*** 205.1, 167.6, 158.9, 146.3, 140.4, 137.2, 135.1, 131.4, 130.9, 130.1, 129.8, 128.5, 127.8, 127.2, 126.7, 123.9, 120.7, 104.6, 55.4, 52.2, 40.8, 32.2, 19.7. **HRMS (ESI):** calcd. for $C_{25}H_{22}NaO_4^+$ [M+Na]$^+$: 409.1410; found: 409.1413; **$[\alpha]_D^{20}$** = +34 (c = 0.1, $CHCl_3$).

HPLC analysis: OD-H column (hexane:2-propanol = 85:15, v = 1.0 mL/min, 40 °C, 254 nm); tr (minor) =6.793 min, tr (major) =7.703 min, 92% ee.

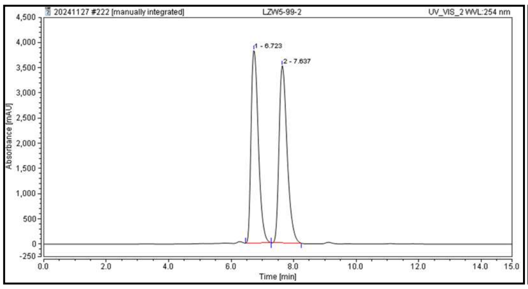

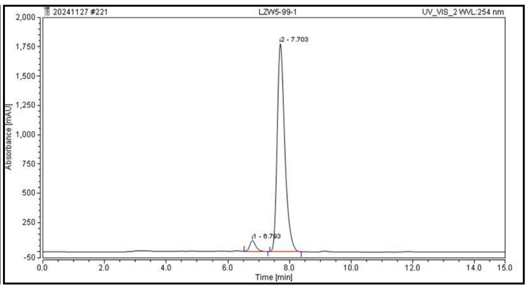

No.	Time	Area	Area (%)	No.	Time	Area	Area (%)
1	6.723	1032.766	49.46	1	6.793	20.878	3.97
2	7.637	1055.494	50.54	2	7.703	505.327	96.03

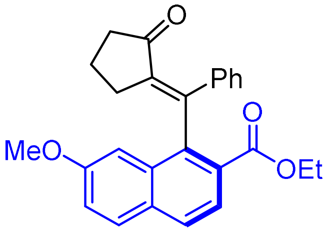

(*R*)-ethyl (*E*)-7-methoxy-1-((2-oxocyclopentylidene)(phenyl)methyl)-2-naphthoate (45).

The title compound was isolated as a red oil liquid

(eluent: petroleum ether/ethyl acetate = 4/1, 28.8 mg, 72%). **^{1}H NMR (600 MHz, Chloroform-d) *δ*** 7.83 – 7.77 (m, 3H), 7.40 – 7.35 (m, 2H), 7.33 – 7.30 (m, 1H), 7.25 – 7.17 (m, 4H), 4.32 – 4.18 (m, 2H), 3.78 (s, 3H), 2.52 – 2.37 (m, 3H), 2.24 – 2.16 (m, 1H), 1.93 – 1.86 (m, 1H), 1.85 – 1.75 (m, 1H), 1.23 (t, *J* = 7.2, 3H).**^{13}C NMR (150 MHz Chloroform-d) *δ*** 205.3, 167.6, 158.9, 146.6, 140.1, 137.3, 135.2, 131.5, 131.0, 130.3, 129.9, 128.6, 127.9, 127.4, 127.3, 124.0, 120.7, 104.7, 61.4, 55.5, 40.9, 32.4, 19.8, 14.3. **HRMS (ESI):** calcd. for $C_{26}H_{24}NaO_4^+$ $[M+Na]^+$: 423.1567; found: 423.1572; **$[\alpha]_D^{20}$** = +58 (c = 0.1, $CHCl_3$).

HPLC analysis: OD-H column (hexane:2-propanol = 95:5, v = 1.0 mL/min, 40 °C, 254 nm); tr (minor) =8.123 min, tr (major) =10.296 min, 91% ee.

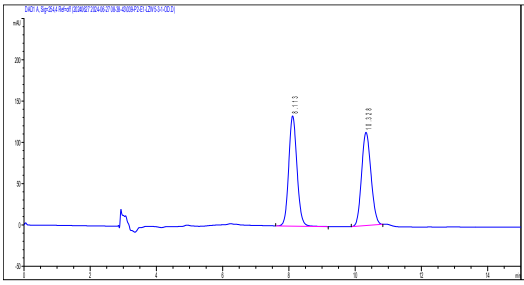

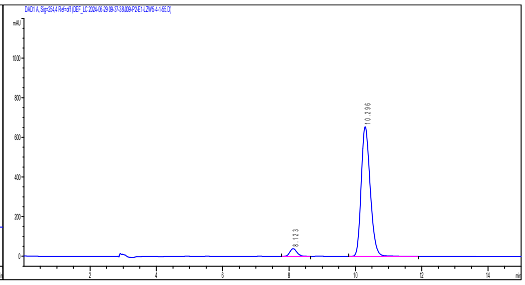

No.	Time	Area	Area (%)	No.	Time	Area	Area (%)
1	8.113	2307.1	50.941	1	8.123	585.8	4.509
2	10.328	2221.8	49.059	2	10.296	12405.4	95.491

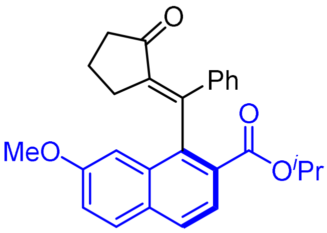

(*R*)-isopropyl (*E*)-7-methoxy-1-((2-oxocyclopentylidene)(phenyl)methyl)-2-naphthoate (46).

The title compound was isolated as a red oil liquid (eluent: petroleum ether/ethyl acetate = 4/1, 33.1 mg, 80%).**^{1}H NMR (600 MHz, Chloroform-d) *δ*** 7.81 – 7.75 (m, 3H), 7.44 – 7.40 (m, 2H), 7.29 – 7.26 (m, 1H), 7.23 – 7.17 (m, 4H), 5.18 – 5.09 (m, 1H), 3.76 (s, 3H), 2.52 – 2.39 (m, 3H), 2.23 – 2.15 (m, 1H), 1.95 – 1.85 (m, 1H), 1.83 – 1.76 (m, 1H), 1.26 (d, *J* = 6.3, 3H), 1.15 (d, *J* = 6.2, 3H).**^{13}C NMR (150 MHz, Chloroform-d) *δ*** 205.3, 167.3, 158.8, 146.7, 139.7, 137.3, 135.3, 131.5, 130.9, 130.5, 129.9, 128.6, 128.0, 127.8, 127.3, 123.9, 120.6, 104.7, 69.1, 55.4, 40.9, 32.4, 21.9, 19.7. **HRMS (ESI):** calcd. for $C_{27}H_{26}NaO_4^+$ $[M+Na]^+$: 437.1723; found: 437.1719; **$[\alpha]_D^{20}$** = +92 (c = 0.1, $CHCl_3$).

HPLC analysis: OD-H column (hexane:2-propanol = 95:5, v = 1.0 mL/min, 40 °C, 254

nm); tr (minor) =5.863 min, tr (major) =8.333min, 90% ee.

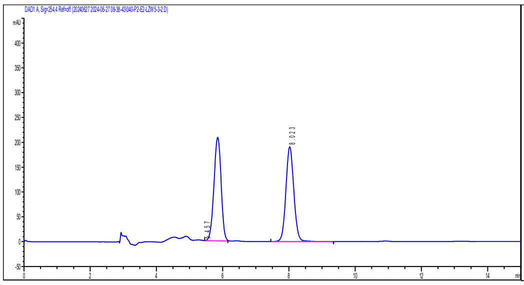

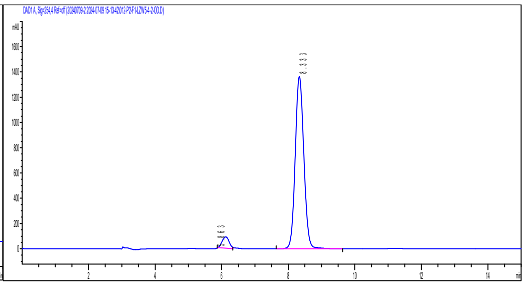

No.	Time	Area	Area (%)	No.	Time	Area	Area (%)
1	5.849	3147.8	49.681	1	5.863	1294.7	5.037
2	8.023	3188.2	50.319	2	8.333	24407.8	94.963

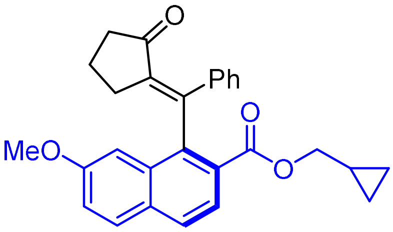

(*R*)-cyclopropylmethyl (*E*)-7-methoxy-1-((2-oxocyclopentylidene)(phenyl)methyl)-2-naphthoate (47).

The title compound was isolated as a red oil liquid (eluent: petroleum ether/ethyl acetate = 5/1, 32.0 mg, 75%).**^{1}H NMR (600 MHz, Chloroform-d) *δ*** 7.88 – 7.79 (m, 3H), 7.45 – 7.40 (m, 2H), 7.36 – 7.33 (m, 1H), 7.27 – 7.21 (m, 4H), 4.10 – 4.01 (m, 2H), 3.81 (s, 3H), 2.53 – 2.41 (m, 3H), 2.26 – 2.18 (m, 1H), 1.97 – 1.90 (m, 1H), 1.87 – 1.76 (m, 1H), 1.17 – 1.07 (m, 1H), 0.59 – 0.49 (m, 2H), 0.32 – 0.23 (m, 2H). **^{13}C NMR (150 MHz, Chloroform-d) *δ*** 205.3, 167.7, 158.9, 146.5, 140.1, 137.3, 135.2, 131.5, 131.0, 130.3, 129.9, 128.6, 127.9, 127.4, 127.3, 124.1, 120.7, 104.7, 70.3, 55.5, 53.6, 41.0, 32.4, 19.8, 9.9, 3.6, 3.5. **HRMS (ESI):** calcd. for $C_{28}H_{26}NaO_4^+$ [M+Na]$^+$: 449.1723; found: 449.1727; **$[\alpha]_D^{20}$** = +22 (c = 0.1, $CHCl_3$).

HPLC analysis: OD-H column (hexane:2-propanol = 95:5, v = 1.0 mL/min, 40 °C, 254 nm); tr (minor) =8.826 min, tr (major) =10.429 min, 90% ee.

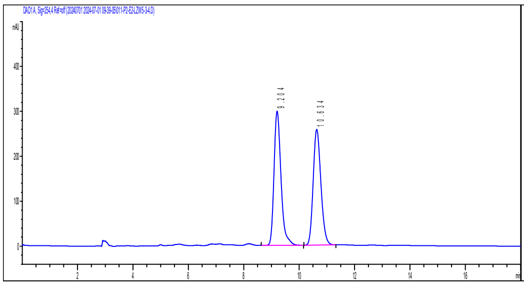

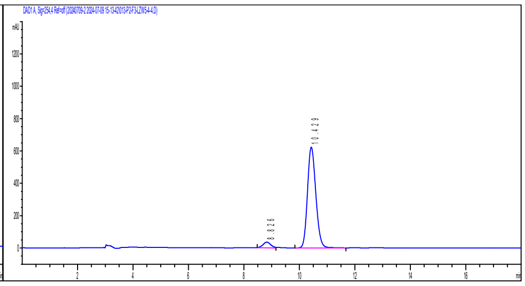

No.	Time	Area	Area (%)	No.	Time	Area	Area (%)
1	9.204	5238.3	51.23	1	8.826	697	5.078
2	10.634	4986.8	48.77	2	10.429	13029.9	94.922

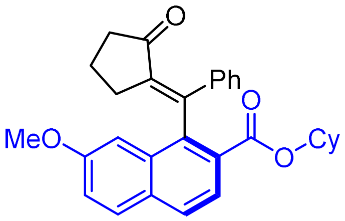

(*R*)-cyclohexyl (*E*)-7-methoxy-1-((2-oxocyclopentylidene)(phenyl)methyl)-2-naphthoate (48). The title compound was isolated as a red oil liquid (eluent: petroleum ether/ethyl acetate = 4/1, 33.2 mg, 73%).^{1}**H NMR (600 MHz, Chloroform-d) *δ*** 7.80 – 7.74 (m, 3H), 7.44 – 7.39 (m, 2H), 7.29 – 7.27 (m, 1H), 7.24 – 7.18 (m, 4H), 4.93 – 4.85 (m, 1H), 3.77 (s, 3H), 2.48 – 2.40 (m, 3H), 2.23 – 2.15 (m, 1H), 1.95 – 1.85 (m, 2H), 1.84 – 1.68 (m, 3H), 1.67 – 1.61 (m, 1H), 1.55 – 1.49 (m, 1H), 1.47 – 1.29 (m, 5H), 1.24 – 1.15 (m, 1H). ^{13}**C NMR (150 MHz, Chloroform-d) *δ*** 205.3, 167.2, 158.8, 146.7, 139.8, 137.3, 135.2, 131.5, 130.9, 130.5, 129.9, 128.6, 128.0, 127.8, 127.3, 124.0, 120.6, 104.7, 74.2, 55.5, 41.0, 32.4, 31.7, 31.6, 25.4, 24.0, 23.9, 19.8. **HRMS (ESI):** calcd. for $C_{30}H_{30}NaO_4^+$ [M+Na]$^+$: 477.2036; found: 477.2306; **[α]$_D^{20}$** = +26 (c = 0.1, $CHCl_3$).

HPLC analysis: OD-H column (hexane:2-propanol =95:5, v = 1.0 mL/min, 40 °C, 254 nm); tr (minor) = 5.576 min, tr (major) =8.33 min, 90% ee.

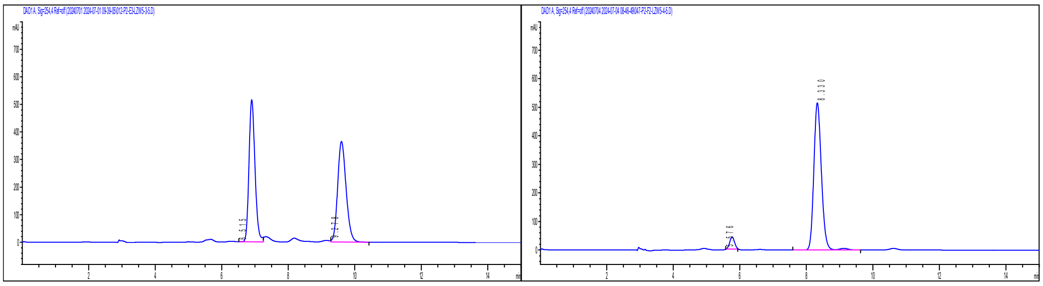

No.	Time	Area	Area (%)	No.	Time	Area	Area (%)
1	6.515	6439.4	49.966	1	5.576	424.3	4.921
2	9.292	6448.2	50.034	2	8.33	8196.6	95.079

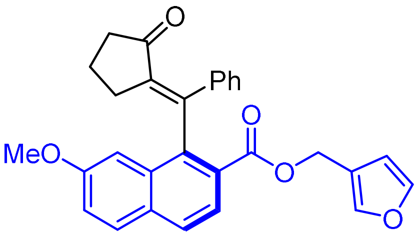

(*R*)-furan-3-ylmethyl (*E*)-7-methoxy-1-((2-oxocyclopentylidene)(phenyl)methyl)-2-naphthoate (49).

The title compound was isolated as a red oil liquid (eluent: petroleum ether/ethyl acetate = 5/1, 31.2 mg, 69%).^{1}**H NMR (600 MHz, Chloroform-d) *δ*** 7.83 – 7.72 (m, 3H), 7.46 – 7.42 (m, 1H), 7.38 – 7.33 (m, 3H), 7.32 – 7.27 (m, 1H), 7.25 – 7.17 (m, 4H), 6.38 – 6.35 (m, 1H), 5.13 (d, *J* = 12.4, 1H), 5.07 (d, *J* = 12.4, 1H), 3.77 (s, 3H), 2.45 – 2.35 (m, 2H), 2.34 – 2.26 (m, 1H), 2.18 – 2.08 (m, 1H), 1.81 – 1.70 (m, 2H). ^{13}**C NMR (150 MHz, Chloroform-d) *δ***

205.3, 167.4, 159.0, 146.3, 143.4, 142.1, 140.4, 137.2, 135.2, 131.5, 131.1, 130.3, 129.9, 128.6, 127.9, 127.4, 126.9, 124.0, 120.9, 120.2, 111.0, 104.8, 58.5, 55.5, 40.8, 32.3, 19.6. **HRMS (ESI):** calcd. for $C_{29}H_{24}NaO_5^+$ $[M+Na]^+$: 475.1516; found: 475.1510; $[\alpha]_D^{20}$ = +28 (c = 0.1, $CHCl_3$).

HPLC analysis: OD-H column (hexane:2-propanol = 95:5, v = 1.0 mL/min, 40 °C, 254 nm); tr (minor) = 12.440 min, tr (major) = 15.557min, 90% ee.

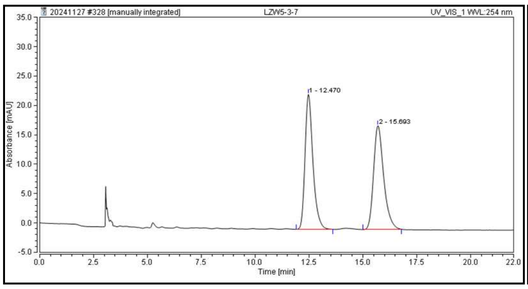

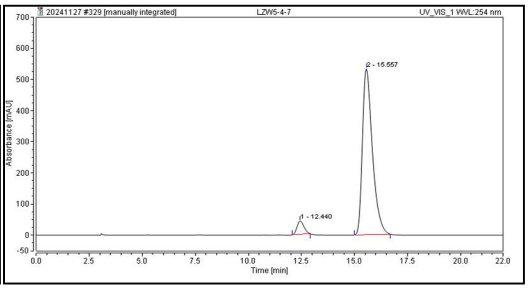

No.	Time	Area	Area (%)	No.	Time	Area	Area (%)
1	12.470	9.764	50.42	1	12.440	16.209	5.29
2	15.693	9.600	49.58	2	15.557	290.296	94.71

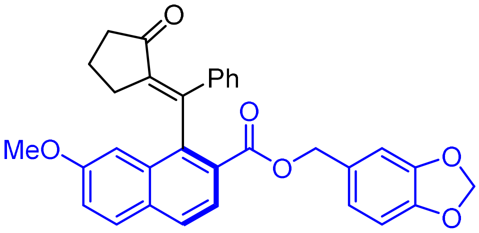

(*R*)-benzo[d][1,3]dioxol-5-ylmethyl (*E*)-7-methoxy-1-((2-oxocyclopentylidene)(phenyl)methyl)-2-naphthoate (50).

The title compound was isolated as a red oil liquid (eluent: petroleum ether/ethyl acetate = 5/1, 32.9 mg, 65%).**^{1}H NMR (600 MHz, Chloroform-d) *δ*** 7.83 – 7.74 (m, 3H), 7.33 – 7.29 (m, 3H), 7.25 – 7.16 (m, 4H), 6.82 – 6.74 (m, 3H), 5.97 – 5.93 (m, 2H), 5.20 – 5.04 (m, 2H), 3.77 (s, 3H), 2.42 – 2.36 (m, 2H), 2.35 – 2.29 (m, 1H), 2.19 – 2.11 (m, 1H), 1.81 – 1.71 (m, 2H). **^{13}C NMR (150 MHz, Chloroform-d) *δ*** 205.2, 167.3, 159.0, 147.9, 147.8, 146.1, 140.4, 137.2, 135.2, 131.5, 131.1, 130.3, 129.9, 129.5, 128.6, 127.9, 127.3, 126.9, 124.1, 122.8, 120.9, 109.5, 108.3, 104.8, 101.3, 67.2, 55.5, 40.8, 32.3, 19.6. **HRMS (ESI):** calcd. for $C_{32}H_{26}NaO_6^+$ $[M+Na]^+$: 529.1622; found: 529.1622; $[\alpha]_D^{20}$ = +64 (c = 0.1, $CHCl_3$).

HPLC analysis: OD-H column (hexane:2-propanol = 95:5, v = 1.0 mL/min, 40 °C, 254 nm); tr (minor) =20.505 min, tr (major) =32.364min, 86% ee.

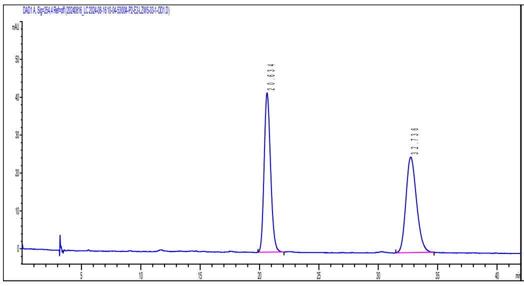

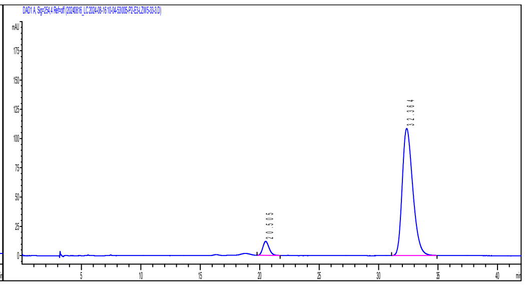

No.	Time	Area	Area (%)	No.	Time	Area	Area (%)
1	20.634	1583.6	50.198	1	20.505	444.2	7.004
2	32.736	1571.1	49.802	2	32.364	6708.8	92.996

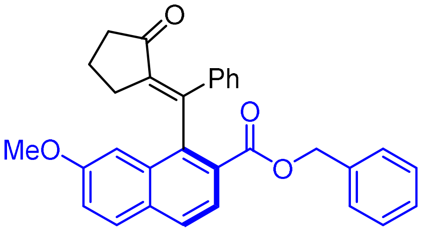

(*R*)-benzyl (*E*)-7-methoxy-1-((2-oxocyclopentylidene)(phenyl)methyl)-2-naphthoate (51).

The title compound was isolated as a red oil liquid (eluent: petroleum ether/ethyl acetate = 4/1, 32.3 mg, 70%).^{1}**H NMR (600 MHz, Chloroform-d) *δ*** 7.84 (d, *J* = 8.5, 1H), 7.81 – 7.76 (m, 2H), 7.32 (d, *J* = 6.1, 8H), 7.25 – 7.20 (m, 2H), 7.20 – 7.16 (m, 2H), 5.31 – 5.26 (m, 1H), 5.19 – 5.14 (m, 1H), 3.77 (s, 3H), 2.39 – 2.26 (m, 3H), 2.18 – 2.08 (m, 1H), 1.76 – 1.65 (m, 2H). ^{13}**C NMR (150 MHz, Chloroform-d) *δ*** 205.2, 167.3, 159.0, 146.1, 140.4, 137.2, 135.7, 135.3, 131.5, 131.1, 130.2, 129.9, 128.8, 128.7, 128.6, 128.5, 127.9, 127.3, 126.8, 124.2, 120.9, 104.7, 67.3, 55.5, 40.8, 32.3, 19.5. **HRMS (ESI):** calcd. for $C_{31}H_{26}NaO_4^+$ [M+Na]$^+$: 485.1723; found: 485.1727; **[α]$_D$^{20}** = +40 (c = 0.1, $CHCl_3$).

HPLC analysis: OD-H column (hexane:2-propanol = 95:5, v = 1.0 mL/min, 40 °C, 254 nm); tr (minor) =11.440 min, tr (major) =15.877min, 88% ee.

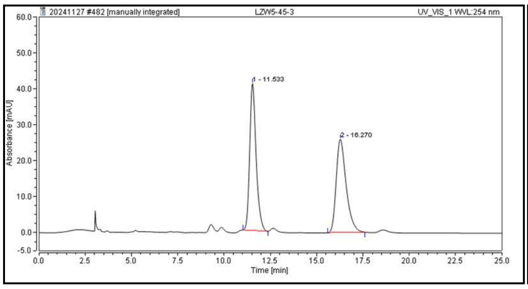

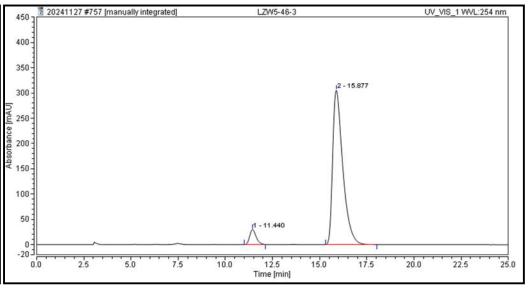

No.	Time	Area	Area (%)	No.	Time	Area	Area (%)
1	11.533	16.414	50.36	1	11.440	11.819	5.87
2	16.270	16.179	49.64	2	15.877	189.409	94.13

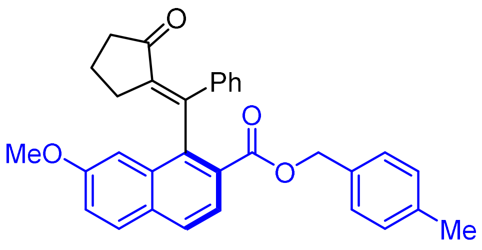

(*R*)-4-methylbenzyl (*E*)-7-methoxy-1-((2-oxocyclopentylidene)(phenyl)methyl)-2-naphthoate (52).

The title compound was isolated as a red oil liquid (eluent: petroleum ether/ethyl acetate = 4/1, 30.5 mg, 64%).**^{1}H NMR (600 MHz, Chloroform-d) *δ*** 7.85 – 7.80 (m, 1H), 7.80 – 7.75 (m, 2H), 7.34 – 7.29 (m, 3H), 7.24 – 7.20 (m, 4H), 7.20 – 7.16 (m, 2H), 7.15 – 7.11 (m, 2H), 5.23 (d, *J* = 12.0, 1H), 5.13 (d, *J* = 12.0, 1H), 3.77 (s, 3H), 2.39 – 2.32 (m, 4H), 2.32 – 2.25 (m, 2H), 2.17 – 2.09 (m, 1H), 1.76 – 1.66 (m, 2H). **^{13}C NMR (150 MHz, Chloroform-d) *δ*** 205.2, 167.3, 159.0, 146.1, 140.4, 138.3, 137.3, 135.2, 132.8, 131.5, 131.0, 130.2, 129.9, 129.3, 128.9, 128.6, 127.8, 127.3, 127.0, 124.2, 120.8, 104.7, 67.2, 55.5, 40.7, 32.3, 21.3, 19.5. **HRMS (ESI):** calcd. for $C_{32}H_{28}NaO_4^+$ $[M+Na]^+$: 499.1880; found: 499.1883; **$[\alpha]_D^{20}$** = +88 (c = 0.1, $CHCl_3$).

HPLC analysis: OD-H column (hexane:2-propanol = 95:5 v = 1.0 mL/min, 40 ^{o}C, 254 nm); tr (minor) = 10.809 min, tr (major) =14.509 min, 91% ee.

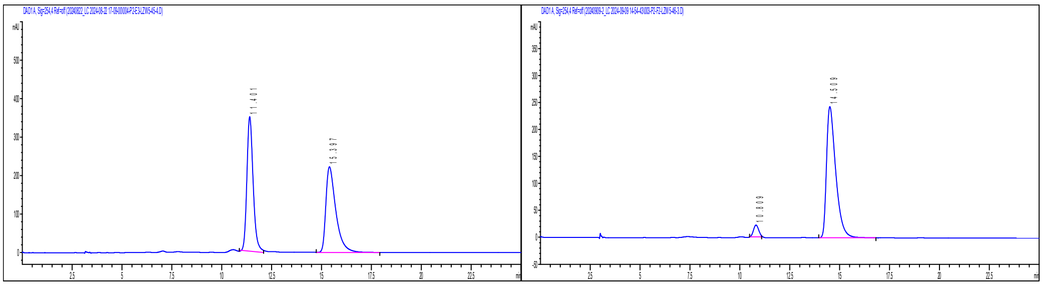

No.	Time	Area	Area (%)	No.	Time	Area	Area (%)
1	11.401	7611.2	49.561	1	10.809	386.3	4.7
2	15.397	7746	50.439	2	14.509	7833.8	95.3

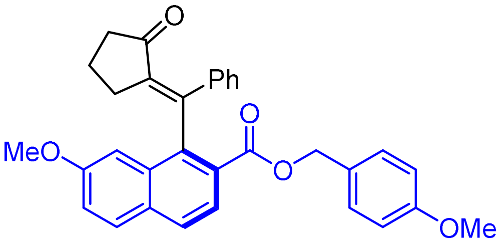

(*R*)-4-methoxybenzyl (*E*)-7-methoxy-1-((2-oxocyclopentylidene)(phenyl)methyl)-2-naphthoate (53).

The title compound was isolated as a red oil liquid (eluent: petroleum ether/ethyl acetate = 5/1, 29.1 mg, 59%).**^{1}H NMR (600 MHz, Chloroform-d) *δ*** 7.84 – 7.79 (m, 1H), 7.79 – 7.75 (m, 2H), 7.35 – 7.28 (m, 3H), 7.27 – 7.24 (m, 2H), 7.24 – 7.16 (m, 4H), 6.87 – 6.82 (m, 2H), 5.21 (d, *J* = 11.9, 1H), 5.12 (d, *J* = 11.8, 1H), 3.79 (s, 3H), 3.77 (s, 3H), 2.41 –

2.33 (m, 1H), 2.32 – 2.24 (m, 2H), 2.16 – 2.08 (m, 1H), 1.75 – 1.66 (m, 2H). **^{13}C NMR (150 MHz, Chloroform-d) *δ*** 205.2, 167.4, 159.8, 159.0, 146.1, 140.3, 137.3, 135.3, 131.5, 131.0, 130.6, 130.2, 129.9, 128.6, 127.9, 127.8, 127.3, 127.0, 124.1, 120.8, 114.1, 104.7, 67.1, 55.5, 55.4, 40.8, 32.3, 19.5. **HRMS (ESI):** calcd. for $C_{32}H_{28}NaO_5^+$ $[M+Na]^+$: 515.1829; found: 515.1835; **$[\alpha]_D^{20}$** = +102 (c = 0.1, $CHCl_3$).

HPLC analysis: OD-H column (hexane:2-propanol = 95:5, v = 1.0 mL/min, 40 °C, 254 nm); tr (minor) =16.064 min, tr (major) =21.438min, 90% ee.

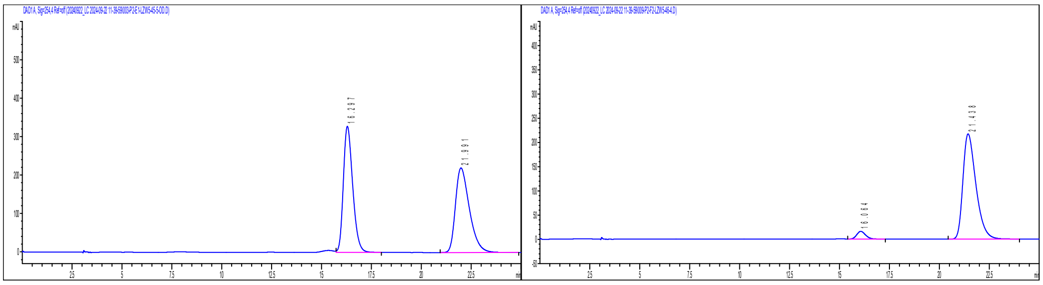

No.	Time	Area	Area (%)	No.	Time	Area	Area (%)
1	16.297	10690.6	49.952	1	16.064	493.1	4.778
2	21.991	10711.3	50.048	2	21.438	9826.5	95.222

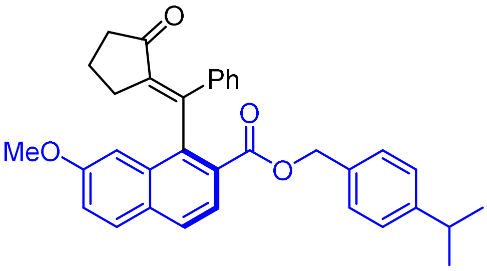

(*R*)-4-isopropylbenzyl (*E*)-7-methoxy-1-((2-oxocyclopentylidene)(phenyl)methyl)-2-naphthoate (54).

The title compound was isolated as a red oil liquid (eluent: petroleum ether/ethyl acetate = 5/1, 26.7 mg, 53%).**^{1}H NMR (600 MHz, Chloroform-d) *δ*** 7.86 – 7.81 (m, 1H), 7.79 – 7.74 (m, 2H), 7.35 – 7.30 (m, 3H), 7.28 – 7.24 (m, 2H), 7.24 – 7.15 (m, 6H), 5.26 (d, *J* = 11.9, 1H), 5.13 (d, *J* = 12.0, 1H), 3.77 (s, 3H), 2.95 – 2.85 (m, 1H), 2.38 – 2.20 (m, 3H), 2.16 – 2.08 (m, 1H), 1.74 – 1.60 (m, 2H), 1.24 (d, *J* = 7.0, 6H)**. ^{13}C NMR (150 MHz, Chloroform-d) *δ*** 205.1, 167.3, 158.9, 149.2, 145.9, 140.2, 137.2, 135.1, 133.0, 131.4, 130.9, 130.1, 129.8, 129.0, 128.5, 127.7, 127.2, 126.9, 126.6, 124.1, 120.7, 104.6, 67.1, 55.4, 40.7, 33.9, 32.1, 24.0, 23.9, 19.4.
HRMS (ESI): calcd. for $C_{34}H_{32}NaO_4^+$ $[M+Na]^+$: 527.2193; found: 527.2195; **$[\alpha]_D^{20}$** = +120 (c = 0.1, $CHCl_3$).

HPLC analysis: OD-H column (hexane:2-propanol = 95:5, v = 1.0 mL/min, 40 °C, 254 nm; tr (minor) = 8.867 min, tr (major) = 12.209 min, 91% ee.

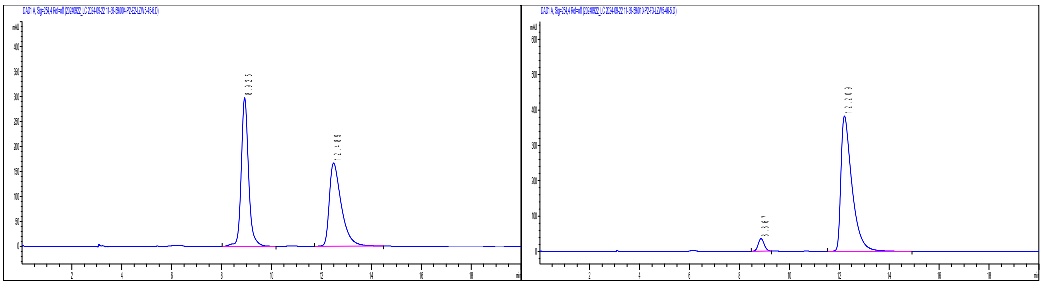

No.	Time	Area	Area (%)	No.	Time	Area	Area (%)
1	8.925	6020.4	51.984	1	8.867	592.6	4.793
2	12.489	5560.8	48.016	2	12.209	11770.3	95.207

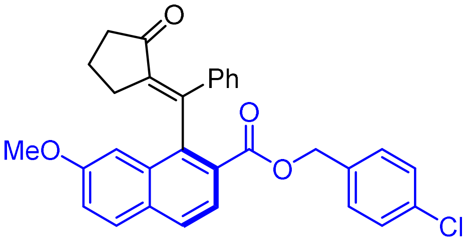

(*R*)-4-chlorobenzyl (*E*)-7-methoxy-1-((2-oxocyclopentylidene)(phenyl)methyl)-2-naphthoate (55).

The title compound was isolated as a red oil liquid (eluent: petroleum ether/ethyl acetate = 4/1, 30.8 mg, 62%).^{1}**H NMR (600 MHz, Chloroform-d)** ***δ*** 7.83 – 7.76 (m, 3H), 7.34 – 7.30 (m, 3H), 7.29 – 7.26 (m, 2H), 7.25 – 7.21 (m, 4H), 7.20 – 7.15 (m, 2H), 5.23 (d, *J* = 12.3, 1H), 5.13 (d, *J* = 12.3, 1H), 3.77 (s, 3H), 2.44 – 2.24 (m, 3H), 2.18 – 2.10 (m, 1H), 1.78 – 1.70 (m, 2H). ^{13}**C NMR (150 MHz, Chloroform-d)** ***δ*** 205.2, 167.1, 159.0, 146.1, 140.5, 137.1, 135.3, 134.4, 134.2, 131.5, 131.1, 130.2, 130.1, 129.9, 128.8, 128.6, 127.9, 127.3, 126.7, 124.0, 120.9, 104.7, 66.3, 55.5, 40.8, 32.3, 19.6. **HRMS (ESI):** calcd. for $C_{31}H_{25}ClNaO_4^+$ [M+Na]$^+$: 519.1334; found: 519.1331; **$[\alpha]_D^{20}$** = +140 (c = 0.1, $CHCl_3$). **HPLC analysis:** OD-H column (hexane:2-propanol = 95:5, v = 1.0 mL/min, 40 ^{o}C, 254 nm); tr (minor) =14.424 min, tr (major) =17.86 min, 88% ee.

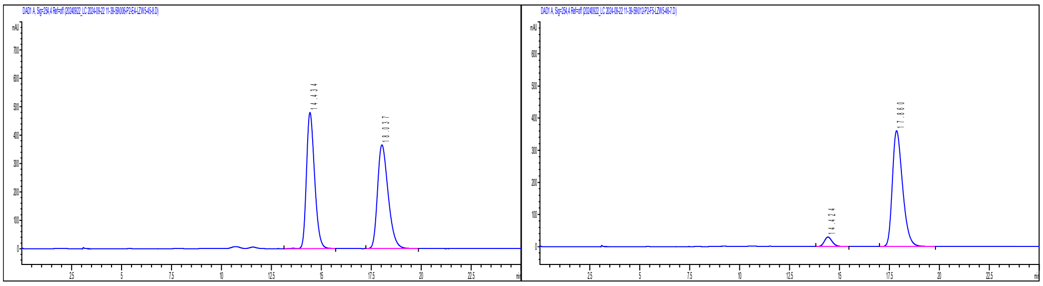

No.	Time	Area	Area (%)	No.	Time	Area	Area (%)
1	14.434	13546.5	50.005	1	14.424	799.7	5.885
2	18.037	13543.7	49.995	2	17.86	12789	94.115

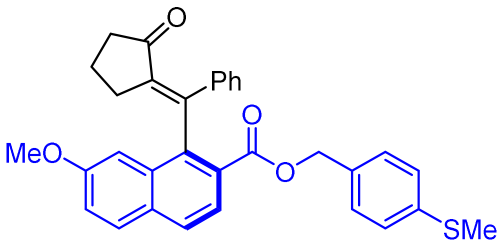

(*R*)-4-(methylthio)benzyl (*E*)-7-methoxy-1-((2-oxocyclopentylidene)(phenyl)methyl)-2-naphthoate (56).

The title compound was isolated as a red oil liquid (eluent: petroleum ether/ethyl acetate = 4/1, 29.5 mg, 58%).**^{1}H NMR (600 MHz, Chloroform-d) *δ*** 7.84 – 7.80 (m, 1H), 7.79 – 7.75 (m, 2H), 7.36 – 7.29 (m, 3H), 7.26 – 7.20 (m, 4H), 7.20 – 7.15 (m, 4H), 5.22 (d, *J* = 12.1, 1H), 5.13 (d, *J* = 12.1, 1H), 3.76 (s, 3H), 2.46 (s, 3H), 2.42 – 2.33 (m, 1H), 2.33 – 2.23 (m, 2H), 2.17 – 2.08 (m, 1H), 1.76 – 1.67 (m, 2H). **^{13}C NMR (150 MHz, Chloroform-d) *δ*** 205.2, 167.2, 159.0, 146.0, 140.3, 139.1, 137.2, 135.2, 132.4, 131.5, 131.0, 130.2, 129.9, 129.5, 128.5, 127.9, 127.3, 126.9, 126.6, 124.0, 120.8, 104.7, 66.8, 55.4, 40.7, 32.2, 19.5, 15.8. **HRMS (ESI):** calcd. for $C_{32}H_{28}NaO_4S^+$ [M+Na]$^+$: 531.1601; found: 531.1602; **$[\alpha]_D^{20}$** = +156 (c = 0.1, $CHCl_3$).

HPLC analysis: OD-H column (hexane:2-propanol = 95:5, v = 1.0 mL/min, 40 °C, 254 nm); tr (minor) = 15.970 min, tr (major) = 19.423 min, 89% ee.

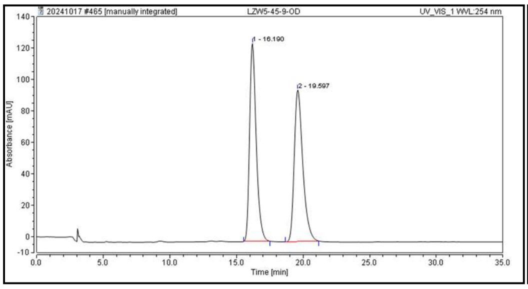

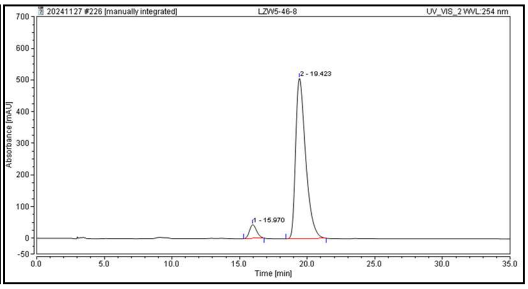

No.	Time	Area	Area (%)	No.	Time	Area	Area (%)
1	16.190	72.704	50.29	1	15.970	26.411	5.73
2	19.597	71.877	49.71	2	19.423	434.278	94.27

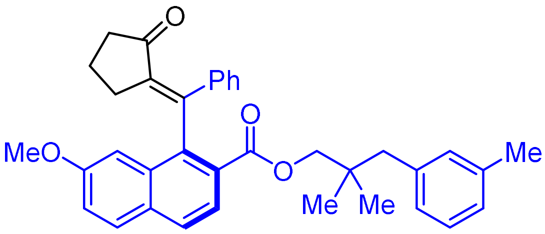

(*R*)-2,2-dimethyl-3-(m-tolyl)propyl (*E*)-7-methoxy-1-((2-oxocyclopentylidene)(phenyl)methyl)-2-naphthoate (57).

The title compound was isolated as a red oil liquid (eluent: petroleum ether/ethyl acetate = 4/1, 24.5 mg, 46%). **^{1}H NMR (600 MHz, Chloroform-d) *δ*** 7.84 – 7.80 (m, 3H), 7.40 – 7.35 (m, 3H), 7.28 – 7.24 (m, 2H), 7.22 – 7.18 (m, 3H), 7.15 – 7.09 (m, 1H), 7.03 – 6.99 (m, 1H), 6.90 – 6.83 (m, 2H), 3.92 (s, 2H), 3.80 (s, 3H), 2.53 (s, 2H), 2.51 – 2.39

(m, 3H), 2.29 (s, 3H), 2.26 – 2.18 (m, 1H), 1.95 – 1.85 (m, 1H), 1.85 – 1.75 (m, 1H), 0.90 (s, 3H), 0.87 (s, 3H). **^{13}C NMR (150 MHz, Chloroform-d) *δ*** 205.2, 167.1, 159.1, 146.3, 140.7, 138.0, 137.5, 137.2, 135.1, 131.6, 131.4, 131.0, 130.4, 129.9, 129.2, 128.6, 127.92, 127.91, 127.7, 127.3, 127.0, 123.9, 120.9, 104.7, 72.9, 55.5, 45.3, 40.9, 35.3, 32.4, 24.52, 24.49, 21.6, 19.7. **HRMS (ESI):** calcd. for $C_{36}H_{36}NaO_4^+$ [M+Na]$^+$: 555.2506; found: 555.2511; **$[\alpha]_D^{20}$** = +24 (c = 0.1, $CHCl_3$).

HPLC analysis: IC column (hexane:2-propanol = 90:10, v = 1.0 mL/min, 40 ^{o}C, 254 nm); tr (major) = 9.513 min, tr (minor) =11.567 min, 87% ee.

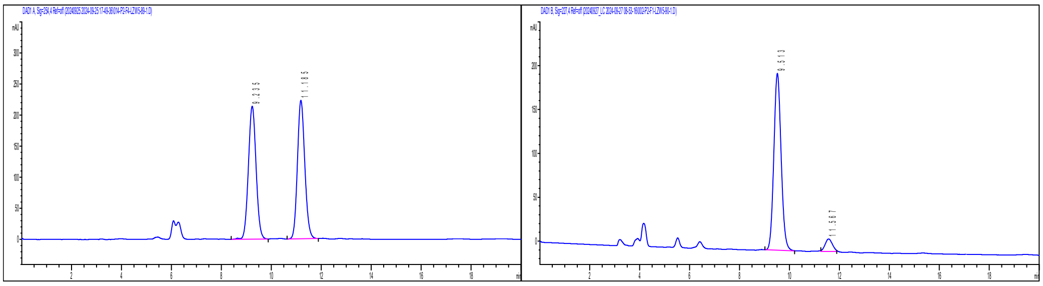

No.	Time	Area	Area (%)	No.	Time	Area	Area (%)
1	9.235	4559.8	49.939	1	9.513	4082.6	93.435
2	11.185	4570.9	50.061	2	11.567	286.9	6.565

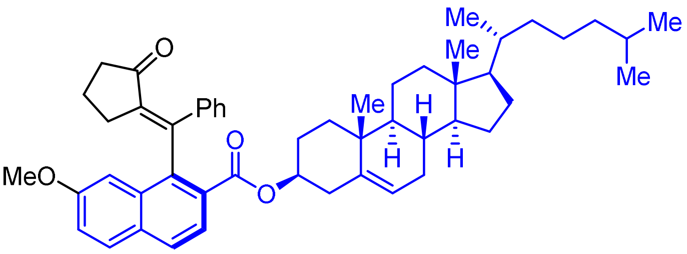

(3*S*,8*S*,9*S*,10*R*,13*R*,14*S*,17*R*)-10,13-dimethyl-17-((*R*)-6-methylheptan-2-yl)-2,3,4,7,8,9,10,11,12,13,14,15,16,17-tetradecahydro-1H-cyclopenta[a]phenanthren-3-yl 7-methoxy-1-((*E*)-(2-oxocyclopentylidene)(phenyl)methyl)-2-naphthoate(58).

The title compound was isolated as a red oil liquid (eluent: petroleum ether/ethyl acetate = 4/1, 36.2 mg, 49%).**^{1}H NMR (600 MHz, Chloroform-d) *δ*** 7.83 – 7.75 (m, 3H), 7.43 – 7.39 (m, 2H), 7.30 – 7.28 (m, 1H), 7.24 – 7.18 (m, 4H), 5.36 – 5.33 (m, 1H), 4.78 – 4.70 (m, 1H), 3.77 (s, 3H), 2.48 – 2.44 (m, 2H), 2.43 – 2.39 (m, 1H), 2.26 – 2.12 (m, 3H), 2.05 – 1.94 (m, 2H), 1.93 – 1.88 (m, 1H), 1.88 – 1.76 (m, 3H), 1.62 – 1.53 (m, 3H), 1.52 – 1.45 (m, 2H), 1.45 – 1.41 (m, 2H), 1.39 – 1.34 (m, 1H), 1.29 – 1.23 (m, 1H), 1.20 – 1.13 (m, 3H), 1.10 – 1.03 (m, 2H), 1.02 – 0.98 (m, 2H), 0.96 (s, 3H), 0.91 (d, *J* = 6.5, 3H), 0.89 – 0.85 (m, 6H), 0.67 (s, 3H). **^{13}C NMR (150 MHz, Chloroform-d) *δ*** 205.3, 167.1,

158.8, 146.7, 139.9, 139.7, 137.2, 135.2, 131.5, 130.9, 130.4, 129.9, 128.7, 127.8, 127.7, 127.3, 124.0, 122.9, 120.7, 104.7, 56.8, 56.3, 55.5, 50.2, 42.4, 41.0, 39.9, 39.7, 38.0, 37.1, 36.7, 36.3, 35.9, 32.4, 32.03, 31.98, 28.4, 28.2, 27.7, 24.4, 24.0, 23.0, 22.7, 21.1, 19.8, 19.4, 18.9, 12.0. **HRMS (ESI):** calcd. for $C_{51}H_{64}NaO_4^+$ $[M+Na]^+$: 763.4697; found: 763.4705; **$[\alpha]_D^{20}$** = +88 (c = 0.1, $CHCl_3$).

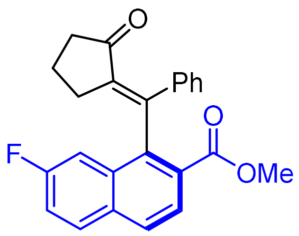

(*R*)-methyl (*E*)-7-fluoro-1-((2-oxocyclopentylidene)(phenyl)methyl)-2-naphthoate (59).

The title compound was isolated as a red oil liquid (eluent: petroleum ether/ethyl acetate = 4/1, 15.3 mg, 41%). **^{1}H NMR (600 MHz, Chloroform-d) *δ*** 7.93 – 7.86 (m, 3H), 7.74 – 7.68 (m, 1H), 7.41 – 7.35 (m, 1H), 7.32 – 7.27 (m, 2H), 7.25 – 7.18 (m, 3H), 3.78 (s, 3H), 2.54 – 2.43 (m, 2H), 2.39 – 2.31 (m, 1H), 2.20 – 2.12 (m, 1H), 1.93 – 1.80 (m, 2H). **^{13}C NMR (150 MHz, Chloroform-d) *δ*** 205.1, 167.2, 161.8 (d, *J* = 248.1), 145.2, 141.1 (d, *J* = 5.9), 136.9, 135.4, 132.3, 131.3 (d, *J* = 8.8), 130.8 (d, *J* = 9.2), 129.9, 128.6, 128.0, 127.3, 127.2, 125.5 (d, *J* = 2.6), 118.5 (d, *J* = 25.5), 110.2 (d, *J* = 22.2), 52.3, 40.6, 32.2, 19.5. **^{19}F NMR (376 MHz, Chloroform-d) *δ***: -111.1 (m). **HRMS (ESI):** calcd. for $C_{24}H_{19}FNaO_3^+$ $[M+Na]^+$: 397.1210; found: 397.1210; **$[\alpha]_D^{20}$** = -58 (c = 0.1, $CHCl_3$).

HPLC analysis: IC column (hexane:2-propanol = 90:10, v = 1.0 mL/min, 40 °C, 227 nm); tr (major) = 16.075 min, tr (minor) = 18.728 min, 95% ee.

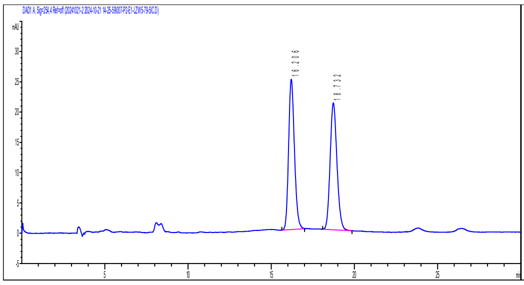

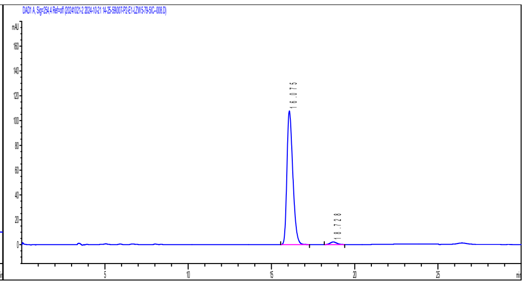

No.	Time	Area	Area (%)	No.	Time	Area	Area (%)
1	16.206	606.3	50.301	1	16.075	2648.6	97.841
2	18.732	599.1	49.699	2	18.728	58.5	2.159

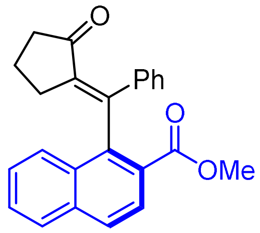

(*R*)-methyl (*E*)-1-((2-oxocyclopentylidene)(phenyl)methyl)-2-naphthoate (60).

The title compound was isolated as a red oil liquid (eluent: petroleum ether/ethyl acetate = 4/1, 22.8 mg, 64%).**^{1}H NMR (600 MHz, Chloroform-d) *δ*** 8.12 – 8.11 (m, 1H), 7.98 – 7.94 (m, 1H), 7.92 – 7.85 (m, 2H), 7.62 – 7.58 (m, 1H), 7.57 – 7.52 (m, 1H), 7.34 – 7.28 (m, 2H), 7.24 – 7.17 (m, 3H), 3.78 (s, 3H), 2.54 – 2.31 (m, 3H), 2.18 – 2.10 (m, 1H), 1.92 – 1.76 (m, 2H). **^{13}C NMR (150 MHz, Chloroform-d) *δ*** 205.4, 167.4, 145.9, 142.0, 137.3, 135.5, 135.3, 130.3, 130.1, 128.6, 128.5, 128.2, 128.1, 127.7, 127.3, 126.9, 126.3, 126.1, 52.3, 40.8, 32.4, 19.6. **HRMS (ESI):** calcd. for $C_{24}H_{20}NaO_3^+$ [M+Na]$^+$: 379.1305; found: 379.1312; **$[\alpha]_D^{20}$** = -20 (c = 0.1, $CHCl_3$).

HPLC analysis: IC column (hexane:2-propanol = 90:10, v = 1.0 mL/min, 40 °C, 254nm); tr (minor) = 19.283 min, tr (major) =20.683 min, 92% ee.

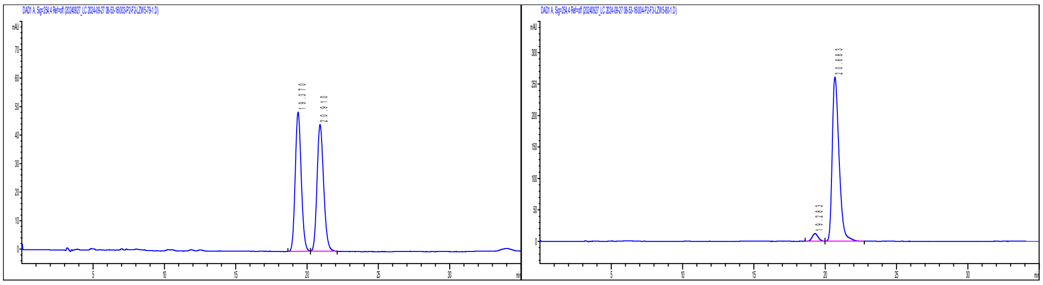

No.	Time	Area	Area (%)	No.	Time	Area	Area (%)
1	19.37	1431.2	49.991	1	19.283	349.3	3.866
2	20.91	1431.7	50.009	2	20.683	8686.4	96.134

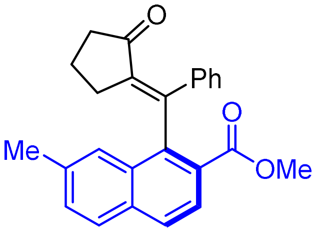

(*R*)-methyl (*E*)-7-methyl-1-((2-oxocyclopentylidene)(phenyl)methyl)-2-naphthoate (61).

The title compound was isolated as a red oil liquid (eluent: petroleum ether/ethyl acetate = 5/1, 22.2 mg, 60%). **^{1}H NMR (600 MHz, Chloroform-d) *δ*** 7.90 – 7.86 (m, 2H), 7.84 – 7.78 (m, 2H), 7.46 – 7.41 (m, 1H), 7.33 – 7.28 (m, 2H), 7.25 – 7.17 (m, 3H), 3.77 (s, 3H), 2.55 – 2.41 (m, 5H), 2.40 – 2.32 (m, 1H), 2.21 – 2.11 (m, 1H), 1.91 – 1.77 (m, 2H). **^{13}C NMR (150 MHz, Chloroform-d) *δ*** 205.5, 167.5, 146.1, 141.4, 137.7, 137.4, 135.2, 133.8, 130.53, 130.46, 130.1, 128.5, 128.3, 127.9, 127.3, 126.2, 125.6, 125.4, 52.3,

40.9, 32.4, 22.2, 19.6. **HRMS (ESI):** calcd. for $C_{25}H_{22}NaO_3^+$ $[M+Na]^+$: 393.1461; found: 393.1462; $[\alpha]_D^{20}$ = -42 (c = 0.1, $CHCl_3$).

HPLC analysis: IC column (hexane:2-propanol = 90:10, v = 1.0 mL/min, 40 °C, 254 nm); tr (major) =13.879 min, tr (minor) =17.492 min, 97% ee.

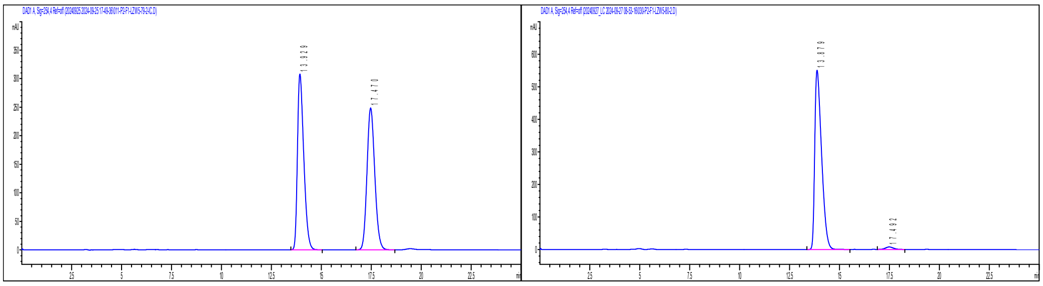

No.	Time	Area	Area (%)	No.	Time	Area	Area (%)
1	13.929	6786	50.177	1	13.879	13088.4	98.439
2	17.47	6738.1	49.823	2	17.492	207.5	1.561

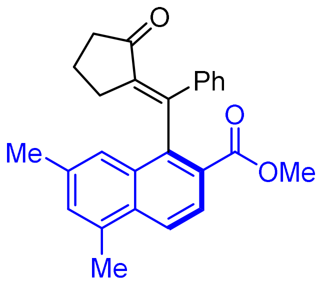

(*R*)-methyl (*E*)-5,7-dimethyl-1-((2-oxocyclopentylidene)(phenyl)methyl)-2-naphthoate (62).

The title compound was isolated as a red oil liquid (eluent: petroleum ether/ethyl acetate = 4/1, 16.1 mg, 42%). **^{1}H NMR (600 MHz, Chloroform-d) *δ*** 7.98 (d, *J* = 8.9, 1H), 7.91 (d, *J* = 8.8, 1H), 7.75 (s, 1H), 7.33 – 7.28 (m, 3H), 7.24 – 7.16 (m, 3H), 3.77 (s, 3H), 2.70 (s, 3H), 2.55 – 2.44 (m, 5H), 2.39 – 2.31 (m, 1H), 2.19 – 2.11 (m, 1H), 1.92 – 1.77 (m, 2H). **^{13}C NMR (150 MHz, Chloroform-d) *δ*** 205.5, 167.6, 146.4, 141.7, 137.5, 137.2, 135.2, 134.6, 133.0, 131.3, 130.8, 130.2, 128.5, 127.2, 125.9, 125.2, 124.1, 123.9, 52.3, 40.9, 32.4, 22.2, 19.7, 19.6. **HRMS (ESI):** calcd. for $C_{26}H_{24}NaO_3^+$ $[M+Na]^+$: 407.1618; found: 407.1623; $[\alpha]_D^{20}$ = -38(c = 0.1, $CHCl_3$).

HPLC analysis: IC column (hexane:2-propanol = 90:10, v = 1.0 mL/min, 40 °C, 254 nm); tr (major) =13.752 min, tr (minor) =18.349 min, 94% ee.

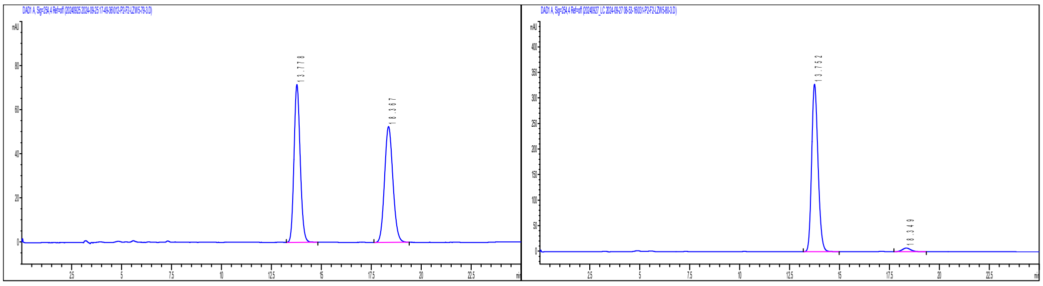

No.	Time	Area	Area (%)	No.	Time	Area	Area (%)
1	13.778	1537.2	50.071	1	13.752	7271.3	97.252
2	18.367	1532.9	49.929	2	18.349	205.4	2.748

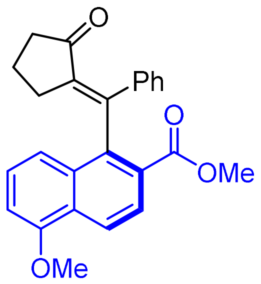

(*R*)-methyl (*E*)-5-methoxy-1-((2-oxocyclopentylidene)(phenyl)methyl)-2-naphthoate (63).

The title compound was isolated as a red oil liquid (eluent: petroleum ether/ethyl acetate = 5/1, 18.1 mg, 47%).**^{1}H NMR (600 MHz, Chloroform-d) *δ*** 8.33 (d, *J* = 8.9, 1H), 7.93 (d, *J* = 8.9, 1H), 7.68 (d, *J* = 8.6, 1H), 7.48 – 7.42 (m, 1H), 7.33 – 7.28 (m, 2H), 7.25 – 7.15 (m, 3H), 6.94 (d, *J* = 7.7, 1H), 4.03 (s, 3H), 3.78 (s, 3H), 2.54 – 2.31 (m, 3H), 2.19 – 2.11 (m, 1H), 1.91 – 1.76 (m, 1H). **^{13}C NMR (150 MHz, Chloroform-d) *δ*** 205.4, 167.6, 155.7, 146.2, 141.4, 137.4, 135.3, 131.4, 130.2, 128.5, 127.8, 127.7, 127.3, 126.7, 125.6, 122.3, 118.9, 105.9, 55.9, 52.3, 40.8, 32.4, 19.6. **HRMS (ESI):** calcd. for $C_{25}H_{22}NaO_4^+$ [M+Na]$^+$: 409.1410; found: 409.1410; **$[\alpha]_D^{20}$** = -16 (c = 0.1, $CHCl_3$).

HPLC analysis: IC column (hexane:2-propanol = 90:10, v = 1.0 mL/min, 40 °C, 254 nm); tr (minor) = 23.173 min, tr (major) =26.766 min, 90% ee.

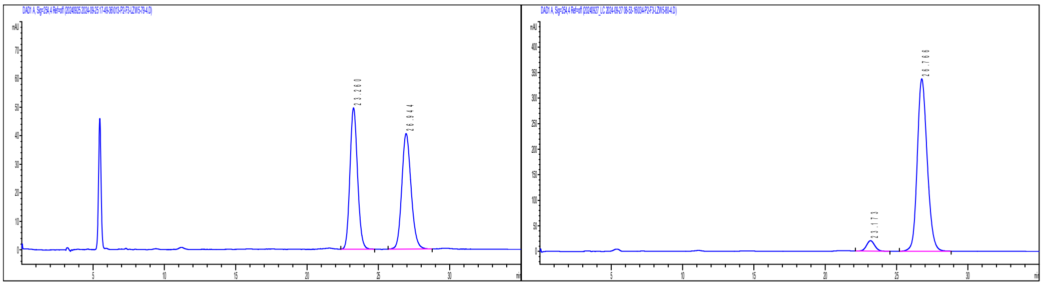

No.	Time	Area	Area (%)	No.	Time	Area	Area (%)
1	23.26	1635.1	50.097	1	23.173	826.2	4.81
2	26.944	1628.8	49.903	2	26.766	16351.7	95.19

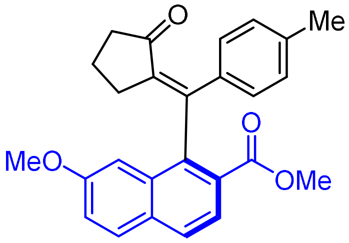

(*R*)-methyl (*E*)-7-methoxy-1-((2-oxocyclopentylidene)(p-tolyl)methyl)-2-naphthoate (64).

The title compound was isolated as a red oil liquid (eluent: petroleum ether/ethyl acetate = 4/1, 29.6 mg, 74%).**^{1}H NMR (600 MHz, Chloroform-d) *δ*** 7.84 – 7.77 (m, 3H), 7.34 – 7.32 (m, 1H), 7.26 – 7.22 (m, 3H), 7.04 – 6.99 (m, 2H), 3.81 – 3.76 (m, 6H), 2.54 – 2.33 (m, 3H), 2.29 (s, 3H), 2.22 – 2.12 (m, 1H), 1.93 – 1.74 (m, 2H). **^{13}C NMR (150 MHz, Chloroform-d) *δ*** 205.3, 167.8,

159.0, 146.7, 140.7, 138.7, 134.6, 134.4, 131.6, 131.0, 130.1, 129.9, 128.1, 127.8, 126.8, 124.0, 120.8, 104.8, 55.5, 52.3, 41.0, 32.4, 21.5, 19.8. **HRMS (ESI):** calcd. for $C_{26}H_{24}NaO_4^+$ [M+Na]$^+$: 423.1567; found: 423.1560. **[α]$_D^{20}$** = -30 (c = 0.1, $CHCl_3$).

HPLC analysis: OD-H column (hexane:2-propanol = 95:5, v = 1.0 mL/min, 40 °C, 254 nm); tr (major) = 8.405 min, tr (minor) =11.023 min, 92% ee.

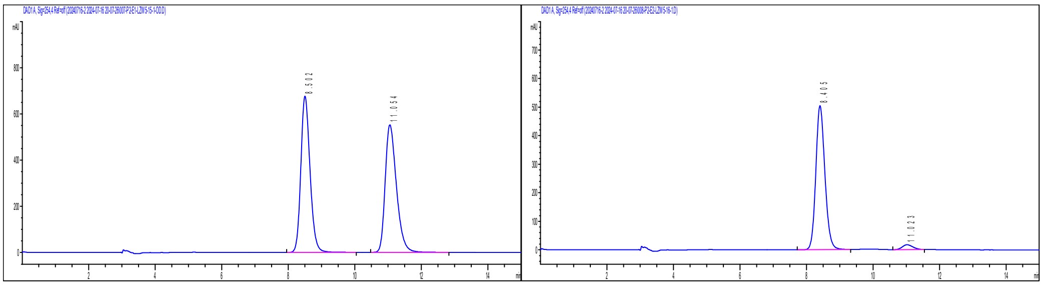

No.	Time	Area	Area (%)	No.	Time	Area	Area (%)
1	8.437	11312.9	50.019	1	8.405	9568.1	96.241
2	11.083	11304.4	49.981	2	11.023	373.7	3.759

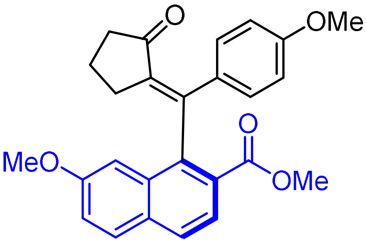

(*R*)-methyl (*E*)-7-methoxy-1-((4-methoxyphenyl)(2-oxocyclopentylidene)methyl)-2-naphthoate (65).

The title compound was isolated as a red oil liquid (eluent: petroleum ether/ethyl acetate = 3/1, 25.4 mg, 61%).**^{1}H NMR (600 MHz, Chloroform-d) *δ*** 7.83 – 7.75 (m, 3H), 7.33 – 7.27 (m, 3H), 7.27 – 7.21 (m, 1H), 6.76 – 6.71 (m, 2H), 3.79 – 3.74 (m, 9H), 2.53 – 2.40 (m, 2H), 2.40 – 2.32 (m, 1H), 2.19 – 2.11 (m, 1H), 1.91 – 1.83 (m, 1H), 1.82 – 1.74 (m, 1H). **^{13}C NMR (150 MHz, Chloroform-d) *δ*** 205.3, 167.8, 160.0, 159.0, 146.6, 140.8, 133.7, 131.9, 131.6, 131.0, 129.9, 129.7, 127.8, 126.8, 124.0, 120.8, 112.7, 104.7, 55.5, 55.3, 52.3, 41.1, 32.5, 19.8. **HRMS (ESI):** calcd. for $C_{26}H_{24}NaO_5^+$ [M+Na]$^+$: 439.1516; found: 439.1510; **[α]$_D^{20}$** = -18 (c = 0.1, $CHCl_3$).

HPLC analysis: IB column (hexane:2-propanol = 85:15, v = 1.0 mL/min, 40 °C, 254 nm); tr (major) = 8.804 min, tr (minor) =12.896 min, 89% ee.

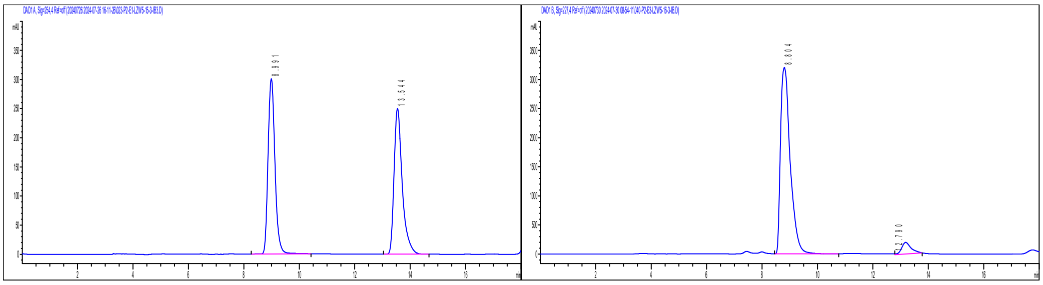

No.	Time	Area	Area (%)	No.	Time	Area	Area (%)
1	8.991	5221	50.45	1	8.804	77706.7	94.259
2	13.544	5127.9	49.55	2	12.896	4733.1	5.741

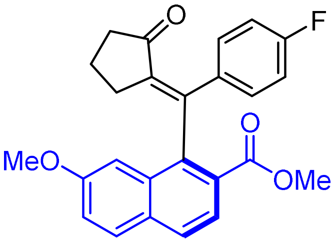

(*R*)-methyl (*E*)-1-((4-fluorophenyl)(2-oxocyclopentylidene)methyl)-7-methoxy-2-naphthoate (66).

The title compound was isolated as a red oil liquid (eluent: petroleum ether/ethyl acetate = 4/1, 27.5 mg, 68%). **^{1}H NMR (600 MHz, Chloroform-d) *δ*** 7.84 – 7.79 (m, 3H), 7.36 – 7.32 (m, 2H), 7.30 – 7.28 (m, 1H), 7.28 – 7.25 (m, 1H), 6.91 – 6.87 (m, 2H), 3.79 (s, 3H), 3.78 (s, 3H), 2.54 – 2.44 (m, 2H), 2.42 – 2.34 (m, 1H), 2.22 – 2.12 (m, 1H), 1.93 – 1.85 (m, 1H), 1.84 – 1.76 (m, 1H). **^{13}C NMR (150 MHz, Chloroform-d) *δ*** 205.2, 167.4, 162.7 (d, *J* = 248.7), 159.0, 145.2, 140.3, 134.9, 133.1 (d, *J* = 3.4), 132.1 (d, *J* = 8.2), 131.3, 131.0, 129.9, 127.9, 126.6, 123.9, 120.8, 114.2 (d, *J* = 21.7), 104.4, 55.4, 52.2, 40.8, 32.2, 19.6. **^{19}F NMR (376 MHz, Chloroform-d) *δ***: -112.5 (m). **HRMS (ESI):** calcd. for $C_{25}H_{21}FNaO_4^+$ [M+Na]$^+$: 427.1316; found: 427.1311; **[α]$_D^{20}$** = +30 (c = 0.1, $CHCl_3$).

HPLC analysis: IC column (hexane:2-propanol = 90:10, v = 1.0 mL/min, 40 °C, 254 nm); tr (major) = 10.179 min, tr (minor) =10.888 min, 90% ee.

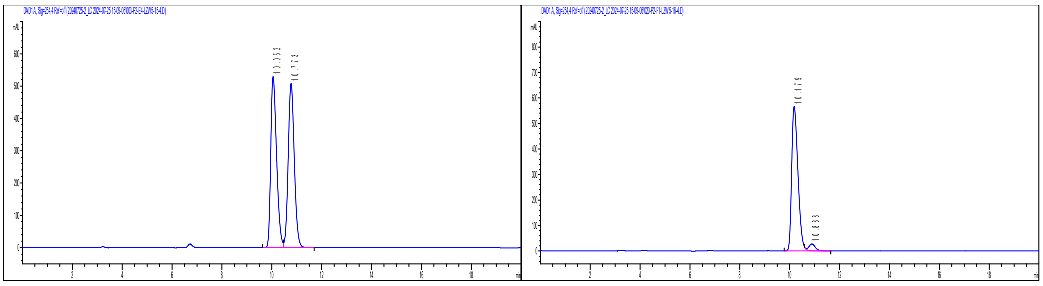

No.	Time	Area	Area (%)	No.	Time	Area	Area (%)
1	10.052	8603	49.828	1	10.179	9912.2	95.382
2	10.773	8662.4	50.172	2	10.888	479.9	4.618

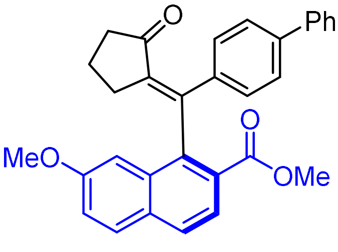

(*R*)-methyl (*E*)-1-((2-oxocyclopentylidene)(phenyl)methyl)-2-naphthoate (67).

The title compound was isolated as a red oil liquid (eluent: petroleum ether/ethyl acetate = 4/1, 27.2 mg, 59%).**^{1}H**

NMR (600 MHz, Chloroform-d) *δ* 7.86 – 7.79 (m, 3H), 7.56 – 7.53 (m, 2H), 7.46 – 7.41 (m, 4H), 7.40 – 7.36 (m, 3H), 7.32 – 7.28 (m, 1H), 7.27 – 7.24 (m, 1H), 3.83 – 3.76 (m, 6H), 2.57 – 2.37 (m, 3H), 2.24 – 2.16 (m, 1H), 1.95 – 1.86 (m, 1H), 1.85 – 1.77 (m, 1H). **^{13}C NMR (150 MHz, Chloroform-d) *δ*** 205.3, 167.7, 159.1, 146.2, 141.2, 140.9, 140.5, 136.3, 135.2, 131.6, 131.1, 130.7, 130.0, 128.8, 127.9, 127.4, 127.2, 126.8, 126.0, 124.1, 120.9, 104.8, 55.5, 52.3, 40.9, 32.4, 19.8. **HRMS (ESI):** calcd. for $C_{31}H_{26}NaO_4^+$ $[M+Na]^+$: 485.1723; found: 485.1730; **$[\alpha]_D^{20}$** = -68 (c = 0.1, $CHCl_3$).

HPLC analysis: IC column (hexane:2-propanol = 90:10, v = 1.0 mL/min, 40 °C, 254 nm); tr (major) = 17.11 min, tr (minor) =21.004 min, 90% ee.

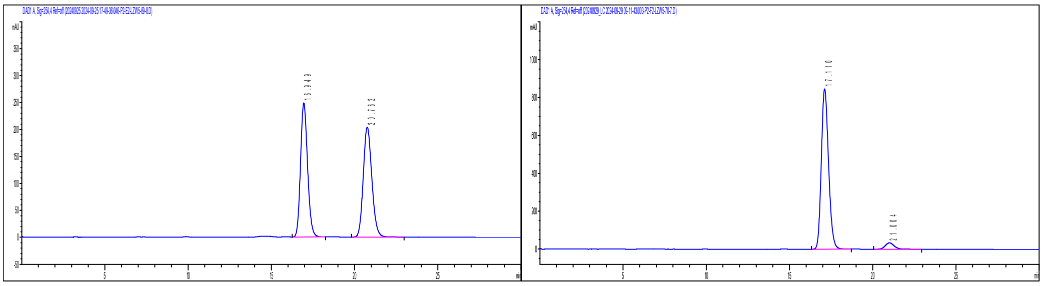

No.	Time	Area	Area (%)	No.	Time	Area	Area (%)
1	16.949	7291	49.854	1	17.11	25525.6	95.307
2	20.762	7333.8	50.146	2	21.004	1256.8	4.693

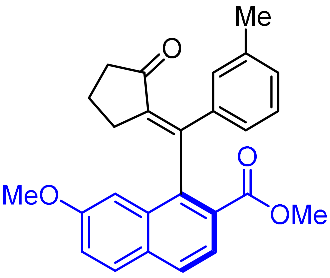

(*R*)-methyl (*E*)-7-methoxy-1-((2-oxocyclopentylidene)(m-tolyl)methyl)-2-naphthoate (68).

The title compound was isolated as a red oil liquid (eluent: petroleum ether/ethyl acetate = 4/1, 26.0 mg, 65%).**^{1}H NMR (600 MHz, Chloroform-d) *δ*** 7.81 – 7.77 (m, 3H), 7.36 – 7.34 (m, 1H), 7.26 – 7.22 (m, 1H), 7.18 – 7.13 (m, 2H), 7.12 – 7.08 (m, 1H), 7.07 – 7.02 (m, 1H), 3.80 – 3.77 (m, 6H), 2.54 – 2.34 (m, 3H), 2.23 (s, 3H), 2.22 – 2.14 (m, 1H), 1.93 – 1.75 (m, 2H). **^{13}C NMR (150 MHz, Chloroform-d) *δ*** 205.2, 167.9, 159.0, 146.5, 140.5, 137.3, 136.7, 135.2, 131.6, 131.0, 130.6, 129.9, 129.5, 127.8, 127.4, 127.2, 126.9, 124.0, 120.8, 104.8, 55.5, 52.3, 40.9, 32.3, 21.5, 19.8. **HRMS (ESI):** calcd. for $C_{26}H_{24}NaO_4^+$ $[M+Na]^+$: 423.1567; found: 423.1565; **$[\alpha]_D^{20}$** = +36 (c = 0.1, $CHCl_3$).

HPLC analysis: OD-H column (hexane:2-propanol = 95:5, v = 1.0 mL/min, 40 °C, 254 nm); tr (minor) = 11.155 min, tr (major) =12.628 min, 91% ee.

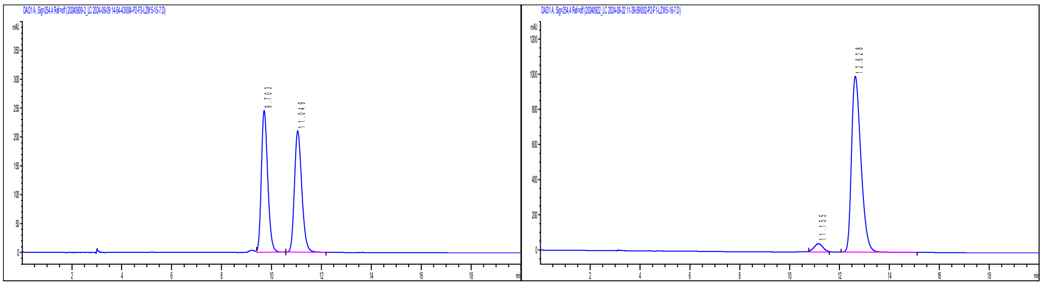

No.	Time	Area	Area (%)	No.	Time	Area	Area (%)
1	9.703	4350.9	50.265	1	11.155	1239.8	4.509
2	11.049	4305	49.735	2	12.628	26254.7	95.491

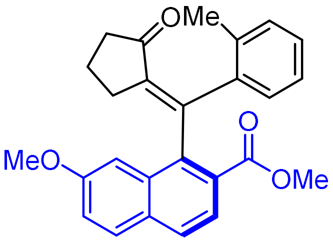

(*R*)-methyl (*E*)-7-methoxy-1-((2-oxocyclopentylidene)(o-tolyl)methyl)-2-naphthoate (69).

The title compound was isolated as a red oil liquid (eluent: petroleum ether/ethyl acetate = 4/1, 16.8 mg, 42%).^{1}**H NMR (600 MHz, Chloroform-d) *δ*** 7.81 – 7.75 (m, 2H), 7.66 – 7.62 (m, 1H), 7.29 – 7.20 (m, 2H), 7.19 – 7.17 (m, 1H), 7.15 – 7.10 (m, 1H), 6.97 – 6.92 (m, 1H), 3.78 (s, 3H), 3.73 (s, 3H), 2.53 – 2.38 (m, 3H), 2.37 – 2.30 (m, 4H), 1.98 – 1.80 (m, 2H).^{13}**C NMR (150 MHz, Chloroform-d) *δ*** 205.4, 168.7, 159.0, 138.3, 137.5, 132.1, 130.7, 130.4, 130.1, 128.11, 128.07, 125.1, 123.5, 120.5, 104.8, 55.5, 52.4, 40.3, 32.6, 20.8, 20.1. **HRMS (ESI):** calcd. for $C_{26}H_{24}NaO_4^+$ $[M+Na]^+$: 423.1567; found:423.1570; **$[\alpha]_D^{20}$** = +84 (c = 0.1, $CHCl_3$).

HPLC analysis: OD-H column (hexane:2-propanol = 95:5, v = 1.0 mL/min, 40 °C, 254 nm); tr (minor) =12.661 min, tr (major) =13.722 min, 86% ee.

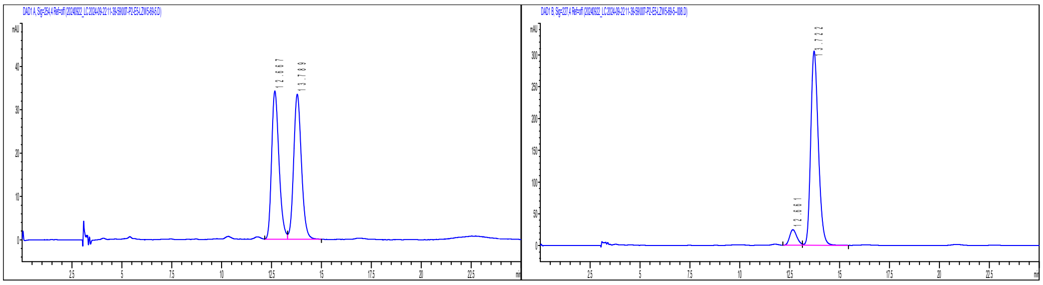

No.	Time	Area	Area (%)	No.	Time	Area	Area (%)
1	12.667	880.2	49.199	1	12.661	616.5	7.09
2	13.789	908.9	50.801	2	13.722	8079	92.91

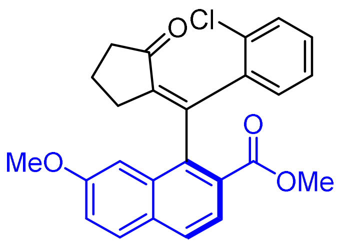

(*R*)-methyl (*Z*)-1-((2-chlorophenyl)(2-oxocyclopentylidene)methyl)-7-methoxy-2-naphthoate (70).

The title compound was isolated as a red oil liquid (eluent: petroleum ether/ethyl acetate = 4/1, 15.1 mg, 36%). **^{1}H NMR (600 MHz, Chloroform-d) *δ*** 7.83 – 7.78 (m, 1H), 7.76 – 7.68 (m, 2H), 7.42 – 7.38 (m, 1H), 7.31 – 7.27 (m, 2H), 7.20 – 7.13 (m, 2H), 7.12 – 7.06 (m, 1H), 3.83 (s, 6H), 2.56 – 2.43 (m, 2H), 2.42 – 2.29 (m, 2H), 1.97 – 1.81 (m, 2H). **^{13}C NMR (150 MHz, Chloroform-d) *δ*** 205.4, 169.2, 158.9, 140.2, 138.6, 138.1, 136.6, 134.0, 132.4, 131.6, 130.7, 129.9, 129.8, 129.6, 129.1, 128.5, 126.8, 123.5, 121.1, 104.5, 56.1, 52.7, 39.4, 32.1, 20.0. **HRMS (ESI):** calcd. for $C_{25}H_{21}ClNaO_4^+$ $[M+Na]^+$: 443.1021; found: 443.1025; **$[\alpha]_D^{20}$** = +66 (c = 0.1, $CHCl_3$).

HPLC analysis: IG column (hexane:2-propanol = 90:10, v = 1.0 mL/min, 40 °C, 227 nm); tr (minor) = 12.470 min, tr (major) =16.353 min, 90% ee.

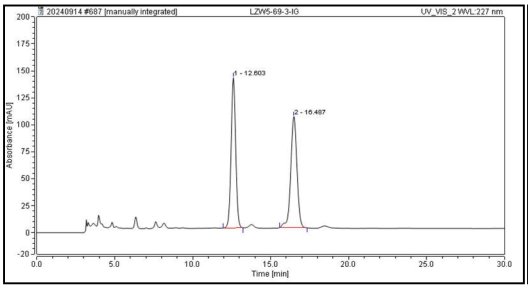

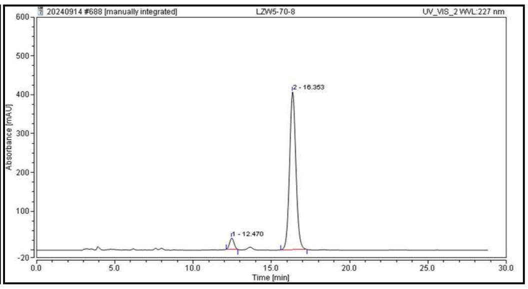

No.	Time	Area	Area (%)	No.	Time	Area	Area (%)
1	12.603	47.329	50.097	1	12.470	9.541	4.77
2	16.487	47.699	49.903	2	16.353	190.622	95.23

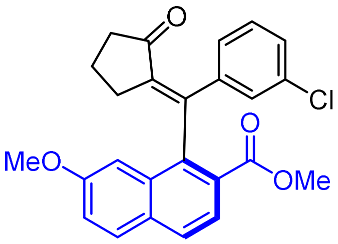

(*R*)-methyl (*E*)-1-((3-chlorophenyl)(2-oxocyclopentylidene)methyl)-7-methoxy-2-naphthoate (71).

The title compound was isolated as a red oil liquid (eluent: petroleum ether/ethyl acetate = 4/1, 25.3mg, 60%). **^{1}H NMR (600 MHz, Chloroform-d) *δ*** 7.86 – 7.77 (m, 3H), 7.33 – 7.24 (m, 4H), 7.23 – 7.18

(m, 1H), 7.17 – 7.11 (m, 1H), 3.82 – 3.77 (m, 6H), 2.55 – 2.34 (m, 3H), 2.23 – 2.15 (m, 1H), 1.94 – 1.76 (m, 2H). **^{13}C NMR (150 MHz, Chloroform-d) *δ*** 205.2, 167.6, 159.2, 144.5, 139.8, 139.1, 136.2, 133.2, 131.4, 131.1, 130.1, 130.0, 128.5, 128.52, 128.51, 128.2, 126.9, 124.0, 120.9, 104.5, 55.5, 52.4, 40.8, 32.2, 19.7. **HRMS (ESI):** calcd. for $C_{25}H_{21}ClNaO_4^+$ $[M+Na]^+$: 443.1021; found: 443.1022; **$[\alpha]_D^{20}$** = -24 (c = 0.1, $CHCl_3$).

HPLC analysis: IC column (hexane:2-propanol = 90:10, v = 1.0 mL/min, 40 °C, 254 nm); tr (major) = 12.204 min, tr (minor) =21.097 min, 93% ee.

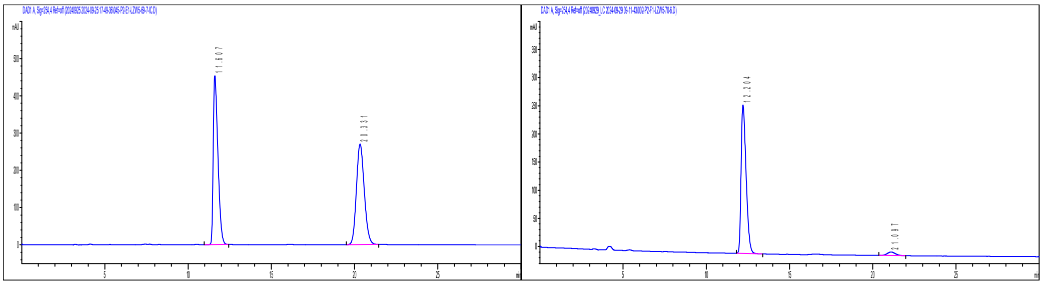

No.	Time	Area	Area (%)	No.	Time	Area	Area (%)
1	11.607	8857	50.034	1	12.204	5511.4	96.338
2	20.331	8845.1	49.966	2	21.097	209.5	3.662

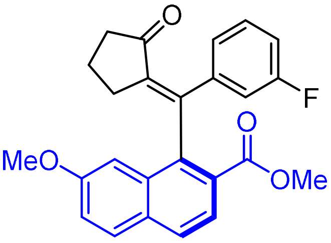

(*R*)-methyl (*E*)-1-((3-fluorophenyl)(2-oxocyclopentylidene)methyl)-7-methoxy-2-naphthoate (72).

The title compound was isolated as a red oil liquid (eluent: petroleum ether/ethyl acetate = 4/1, 25.5 mg, 63%).**^{1}H NMR (600 MHz, Chloroform-d) *δ*** 7.85 – 7.78 (m, 3H), 7.30 (d, *J* = 2.7, 1H), 7.28 – 7.24 (m, 1H), 7.21 – 7.13 (m, 2H), 7.07 – 7.01 (m, 1H), 6.97 – 6.89 (m, 1H), 3.83 – 3.78 (m, 6H), 2.55 – 2.35 (m, 3H), 2.23 – 2.15 (m, 1H), 1.95 – 1.76 (m, 2H). **^{13}C NMR (150 MHz, Chloroform-d) *δ*** 205.0, 167.4, 162.0 (d, *J* = 244.3), 159.0, 144.5 (d, *J* = 2.3), 139.8, 139.4 (d, *J* = 8.0), 136.0, 131.3, 131.0, 129.9, 128.5 (d, *J* = 8.2), 128.0, 126.7, 125.9 (d, *J* = 2.8), 123.9, 120.8, 116.9 (d, *J* = 22.4), 115.3 (d, *J* = 21.0), 104.5, 55.4, 52.2, 40.7, 32.2, 19.6. **^{19}F NMR (376 MHz, Chloroform-d) *δ***: -114.3 (m). **HRMS (ESI):** calcd. for $C_{25}H_{21}FNaO_4^+$ $[M+Na]^+$: 427.1316; found: 427.1313; **$[\alpha]_D^{20}$** = +34 (c = 0.1, $CHCl_3$).

HPLC analysis: IC column (hexane:2-propanol = 90:10, v = 1.0 mL/min, 40 °C, 254 nm); tr (major) =12.166 min, tr (minor) =14.634 min, 93% ee.

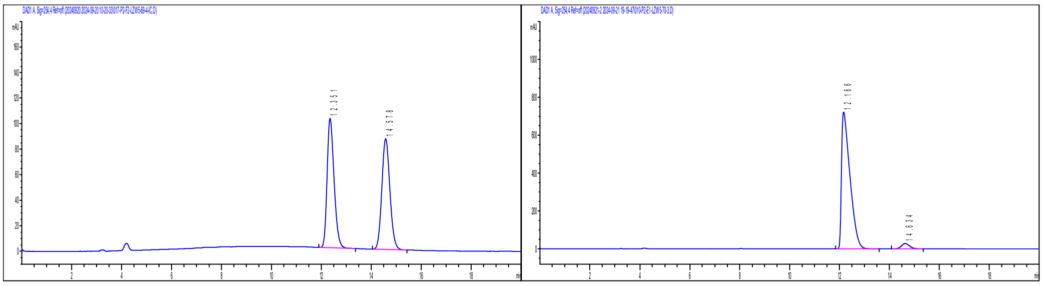

No.	Time	Area	Area (%)	No.	Time	Area	Area (%)
1	12.351	1962.6	50.121	1	12.166	16895.1	96.409
2	14.578	1953.1	49.879	2	14.634	629.3	3.591

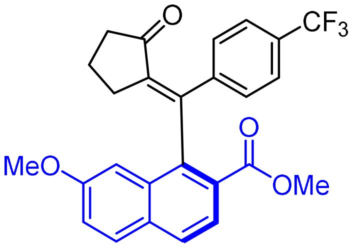

(*R*)-methyl (*E*)-7-methoxy-1-((2-oxocyclopentylidene)(4-(trifluoromethyl)phenyl)methyl)-2-naphthoate (73). The title compound was isolated as a red oil liquid (eluent: petroleum ether/ethyl acetate = 4/1, 24.5 mg, 54%).**^{1}H NMR (600 MHz, Chloroform-d) *δ*** 7.87 – 7.79 (m, 3H), 7.49 – 7.43 (m, 4H), 7.32 – 7.27 (m, 2H), 3.82 – 3.77 (m, 6H), 2.55 – 2.36 (m, 3H), 2.25 – 2.17 (m, 1H), 1.97 – 1.78 (m, 2H). **^{13}C NMR (150 MHz, Chloroform-d) *δ*** 205.2, 167.2, 159.1, 144.4, 140.7, 139.8, 136.4, 131.3, 131.0, 130.3, 130.0, 129.9, 128.1, 126.5, 125.0 (q, *J* = 270.8 Hz), 124.1 (q, *J* = 3.8 Hz), 124.0, 120.8, 104.4, 55.4, 52.3, 40.6, 32.1, 19.5. **^{19}F NMR (376 MHz, Chloroform-d) *δ***: -62.5 (m). **HRMS (ESI):** calcd. for $C_{26}H_{21}F_3NaO_4^+$ [M+Na]$^+$: 477.1284; found: 477.1287; **$[\alpha]_D^{20}$** = -28(c = 0.1, $CHCl_3$).

HPLC analysis: IC column (hexane:2-propanol = 95:5, v = 1.0 mL/min, 40 °C, 254 nm); tr (major) = 7.64 min, tr (minor) =8.397 min, 91% ee.

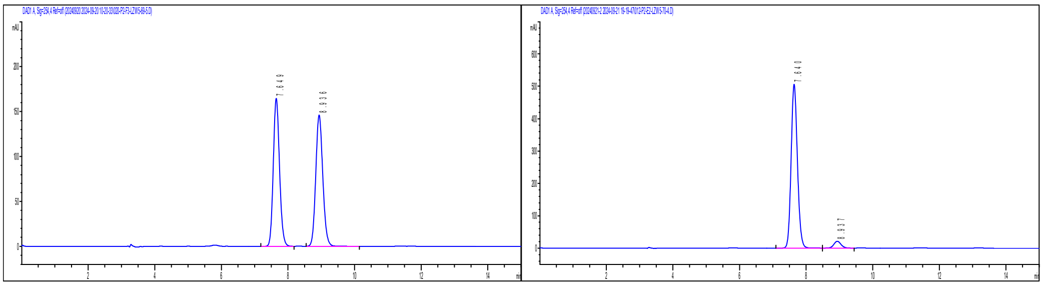

No.	Time	Area	Area (%)	No.	Time	Area	Area (%)
1	7.649	2144.8	49.896	1	7.64	6633.5	95.513
2	8.936	2153.7	50.104	2	8.937	311.6	4.487

(*R*)-methyl (*E*)-1-((4-chlorophenyl)(2-oxocyclopentylidene)methyl)-7-methoxy-2-naphthoate (74).

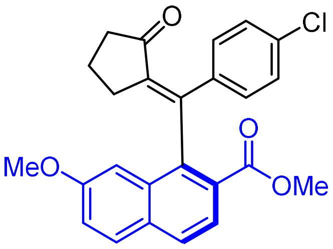

The title compound was isolated as a red oil liquid (eluent: petroleum ether/ethyl acetate = 4/1, 28.2 mg, 67%).**^{1}H NMR (600 MHz, Chloroform-d) *δ*** 7.85 – 7.79 (m, 3H), 7.32 – 7.24 (m, 4H), 7.20 – 7.14 (m, 2H), 3.81 – 3.77 (m, 6H), 2.55 – 2.33 (m, 3H), 2.23 – 2.13 (m, 1H), 1.94 – 1.75 (m, 2H). **^{13}C NMR (150 MHz, Chloroform-d) *δ*** 205.3, 167.5, 159.2, 145.1, 140.2, 135.7, 135.5, 134.5, 131.6, 131.5, 131.1, 130.0, 128.1, 127.6, 126.7, 124.1, 120.9, 104.6, 55.5, 52.4, 40.9, 32.3, 19.7. **HRMS (ESI):** calcd. for $C_{25}H_{21}ClNaO_4^+$ [M+Na]$^+$: 443.1021; found: 443.1015; **[α]$_D$^{20}** = -52 (c = 0.1, $CHCl_3$).

HPLC analysis: IC column (hexane:2-propanol = 85:5, v = 1.0 mL/min, 40 °C, 254 nm); tr (major) = 7.529 min, tr (minor) =9.72 min, 90% ee.

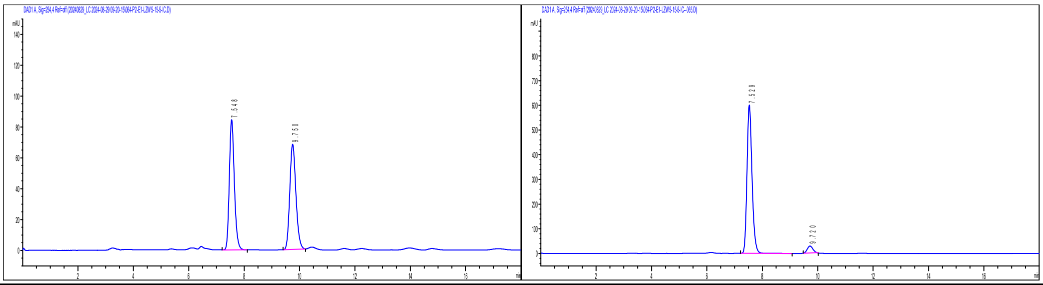

No.	Time	Area	Area (%)	No.	Time	Area	Area (%)
1	7.548	1066.6	50.353	1	7.529	7623.7	95.107
2	9.75	1051.7	49.647	2	9.72	392.2	4.893

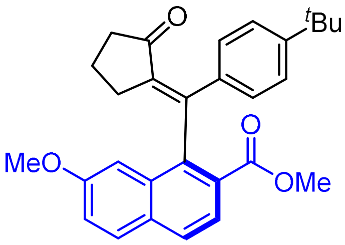

(*R*)-methyl (*E*)-1-((4-(tert-butyl)phenyl)(2-oxocyclopentylidene)methyl)-7-methoxy-2-naphthoate (75).

The title compound was isolated as a red oil liquid (eluent: petroleum ether/ethyl acetate = 4/1, 31.0 mg, 70%).**^{1}H NMR (600 MHz, Chloroform-d) *δ*** 7.83 – 7.75 (m, 3H), 7.34 (d, *J* = 2.7, 1H), 7.30 – 7.27 (m, 2H), 7.26 – 7.20 (m, 3H), 3.79 – 3.76 (m, 6H), 2.54 – 2.34 (m, 3H), 2.22 – 2.13 (m, 1H), 1.93 – 1.74 (m, 2H), 1.26 (s, 9H). **^{13}C NMR (150 MHz, Chloroform-d) *δ***

205.3, 167.8, 159.0, 151.6, 146.7, 140.7, 134.6, 134.3, 131.6, 131.0, 129.94, 129.86, 127.8, 126.8, 124.3, 124.0, 120.9, 104.9, 55.5, 52.3, 40.9, 34.7, 32.4, 31.3, 19.8. **HRMS (ESI):** calcd. for $C_{29}H_{30}NaO_4^+$ [M+Na]$^+$: 465.2036; found: 465.2032; **$[\alpha]_D^{20}$** = -34 (c = 0.1, $CHCl_3$).

HPLC analysis: IC column (hexane:2-propanol = 90:10, v = 1.0 mL/min, 40 °C, 254 nm); tr (major) = 7.034 min, tr (minor) =8.078 min, 91% ee.

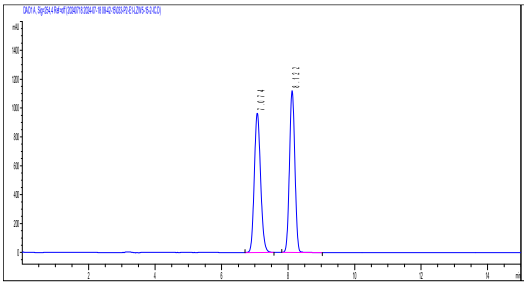

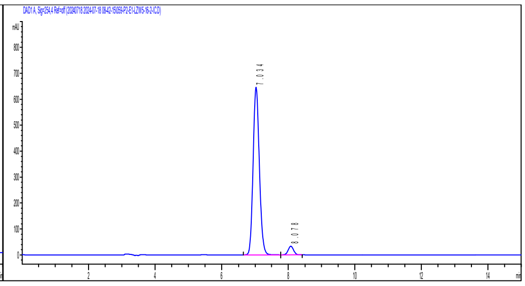

No.	Time	Area	Area (%)	No.	Time	Area	Area (%)
1	7.074	12662.5	50.027	1	7.034	8429.1	95.761
2	8.122	12648.8	49.973	2	8.078	373.1	4.239

4. Synthetic Applications

4.1 1 mmol scale synthesis of 3

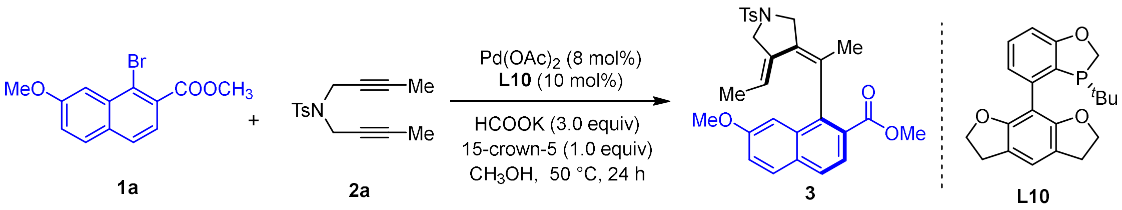

To a sealable tube (25 mL) were charged with aryl bromides **1a** (1.2 mmol), 1,6-diyne (1.0 mmol), $Pd(OAc)_2$ (8 mol%), **L10** (10 mol%), HCOOK (3.0 mmol), 15-Crown-5 (1.0 mmol), and anhydrous MeOH (15.0 mL) under N_2. The resulting mixture was stirred for 24 h at 50 °C. After that, the reaction mixture was concentrated under vacuum. The residue was purified by flash chromatography on silica gel (petroleum ether/EtOAc = 10/1 to 5/1) to afford the desired product as a yellow solid (354 mg, 72% yield). The enantiomeric excess was determined by chiral HPLC analysis.

HPLC analysis: IC column (hexane:2-propanol = 80:20, v = 1.0 mL/min, 40 °C, 254 nm); tr (major) = 22.311 min, tr (minor) = 24.399 min, 95% ee.

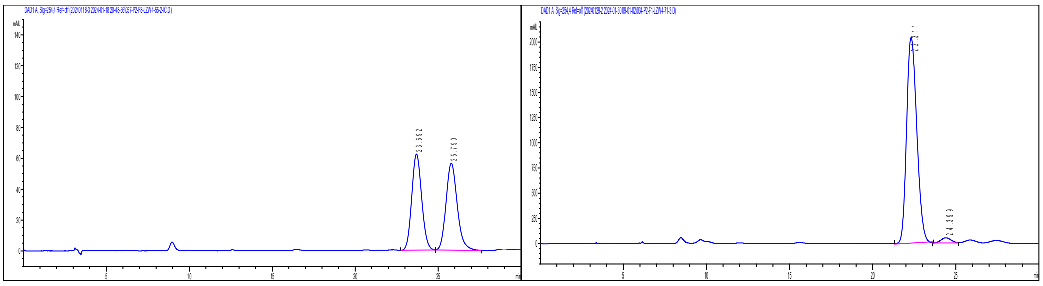

No.	Time	Area	Area (%)	No.	Time	Area	Area (%)
1	23.692	2596.5	49.565	1	22.311	86907.9	97.564
2	25.79	2642.1	50.435	2	24.399	2169.8	2.436

4.2 1 mmol scale synthesis of 44

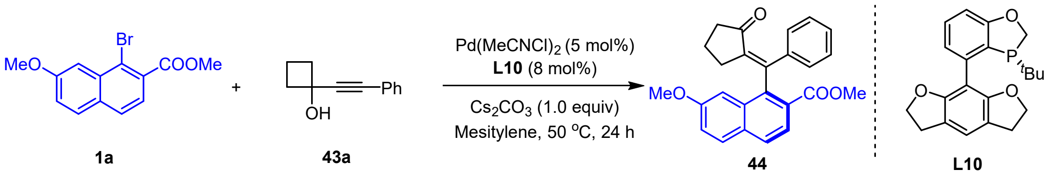

To a sealable tube (25 mL) were charged with aryl bromides **1a** (1.5 mmol), 1-alkynylcyclobutanols (1.0 mmol), $Pd(MeCN)_2Cl_2$, (5 mol%) **L10** (8 mol%), Cs_2CO_3 (1.0 mmol), and anhydrous mesitylene (15.0 mL) under N_2. The resulting mixture was

stirred for 24 h at 50 °C. After that, the residue was purified by flash chromatography on silica gel (petroleum ether/EtOAc = 8/1) to afford the desired product. (294 mg, 76% yield). The enantiomeric excess was determined by chiral HPLC analysis.

HPLC analysis: OD-H column (hexane:2-propanol = 85:15, v = 1.0 mL/min, 40 °C, 254 nm); tr (minor) = 6.793 min, tr (major) = 7.703 min, 92% ee.

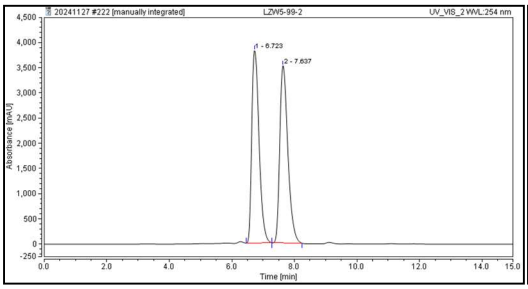

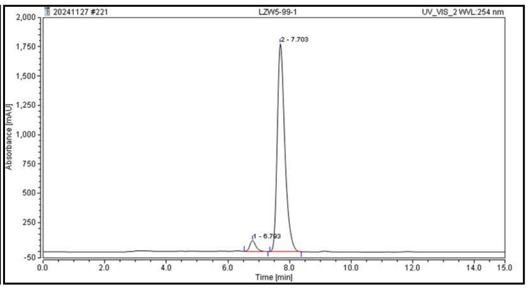

No.	Time	Area	Area (%)	No.	Time	Area	Area (%)
1	6.723	1032.766	49.46	1	6.793	20.878	3.97
2	7.637	1055.494	50.54	2	7.703	505.327	96.03

4.3 General procedure for the synthesis of 76

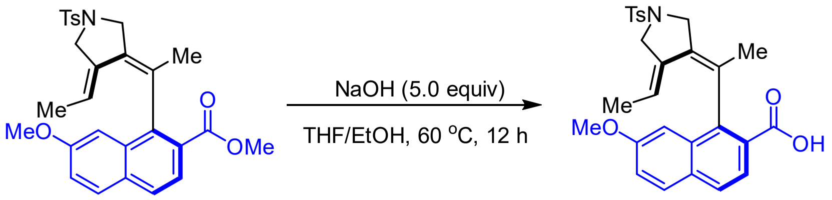

3 **76**

To a mixture of NaOH (0.50 mmol, 5.0 equiv.) in 2.0 mL THF and 2.0 mL EtOH was added **3** (0.10 mmol, 1.0 equiv.), The resulting mixture was stirred for 24 h at 60 °C. After that, the mixture was diluted with water and added hydrochloric acid to adjust PH, extracted with EtOAc (4 mL × 3) and concentrated in vacuo. The residue was purified by flash chromatography on silica gel (petroleum ether/EtOAc = 1/3 to 1/2) to afford the desired product **76** as a yellow solid (36.2 mg, 76% yield). The enantiomeric excess was determined by chiral HPLC analysis. **^{1}H NMR (600 MHz, Chloroform-d) *δ*** 7.96 – 7.92 (m, 1H), 7.82 – 7.75 (m, 4H), 7.39 – 7.33 (m, 2H), 7.28 – 7.24 (m, 1H), 7.12 – 7.08 (m, 1H), 4.39 (d, *J* = 13.0, 1H), 4.21 – 4.13 (m, 2H), 3.93 (d, *J* = 13.5, 1H), 3.85 – 3.78 (m, 4H), 2.42 (s, 3H), 2.09 (s, 3H), 1.17 (d, *J* = 7.2, 3H).**^{13}C NMR (150 MHz, Chloroform-d) *δ*** 171.3, 158.7, 143.8, 143.3, 133.2, 133.1, 131.6, 131.2, 129.90, 129.85, 129.6, 128.3, 128.0, 127.3, 124.73, 124.71, 121.0, 120.4, 104.8, 55.4, 53.0, 51.6, 23.2, 21.7, 15.6. **HRMS (ESI):** calcd. for: $C_{27}H_{27}NNaO_5S^+$ [M+Na]$^+$: 500.1502; found: 500.1506;

[α]$_D^{20}$ = +78 (c = 0.1, $CHCl_3$).

HPLC analysis: AD-H column (hexane:2-propanol = 85:15, v = 1.0 mL/min, 40 °C, 227 nm; tr (major) = 18.260 min, tr (minor) = 20.903 min, 96% ee

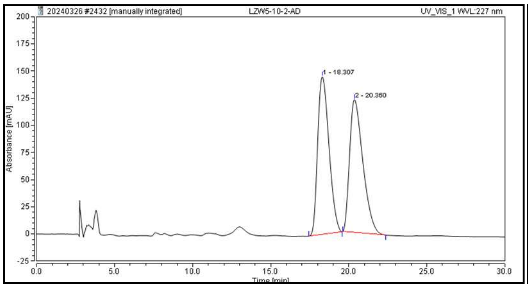

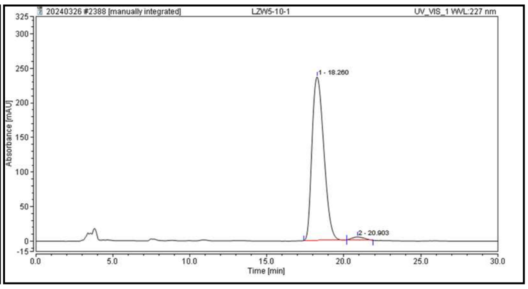

No.	Time	Area	Area (%)	No.	Time	Area	Area (%)
1	18.307	121.962	50.24	1	18.260	210.003	98.14
2	20.360	120.807	49.76	2	20.903	4.084	1.86

4.4 General procedure for the synthesis of 77

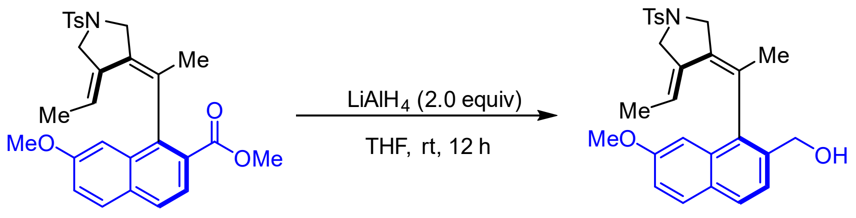

3 **77**

To a sealable tube (15 mL) were charged with **3** (0.1 mmol) and THF (2.0 mL) under argonat 0 °C. Then $LiAlH_4$ (0.2 mmol, 1N in THF) was addeded and the mixture was stirred at r.t for 12 h. After that, the reaction mixture was quenched with H_2O and the mixture was concentrated under vacuum. The residue was purified by flash chromatography on silica gel (petroleum ether/EtOAc = 5/1) to afford the desired product **77** (27.3 mg, 59% yield). The enantiomeric excess was determined by chiral HPLC analysis. **^{1}H NMR (600 MHz, Chloroform-d) *δ*** 7.82 – 7.77 (m, 2H), 7.77 – 7.70 (m, 2H), 7.47 – 7.42 (m, 1H), 7.42 – 7.37 (m, 2H), 7.17 – 7.12 (m, 1H), 6.92 – 6.89 (m, 1H), 4.51 (s, 2H), 4.38 (q, *J* = 7.2, 1H), 4.30 (d, *J* = 12.0, 1H), 4.22 (d, *J* = 15.1, 1H), 3.95 (d, *J* = 14.6, 1H), 3.88 (d, *J* = 13.5, 1H), 3.82 (s, 3H), 2.48 (s, 3H), 2.01 (s, 3H), 1.21 (d, *J* = 6.4, 3H). **^{13}C NMR (150 MHz, Chloroform-d) *δ*** 158.3, 144.0, 136.8, 134.2, 133.5, 133.0, 131.28, 131.25, 130.00, 129.98, 129.1, 128.0, 127.7, 127.4, 124.1, 121.0, 118.1, 104.0, 63.6, 55.4, 52.8, 51.6, 23.2, 21.7, 15.5. **HRMS (ESI):** calcd. for: $C_{27}H_{29}NNaO_4S^+$ [M+Na]$^+$: 486.1710; found: 486.1706; **[α]$_D^{20}$** = +26 (c = 0.1, $CHCl_3$).

HPLC analysis: OD-H column (hexane:2-propanol = 80:20, v = 1.0 mL/min, 40 °C, 254 nm; tr (major) = 8.516 min, tr (minor) = 11.026 min, 93% ee.

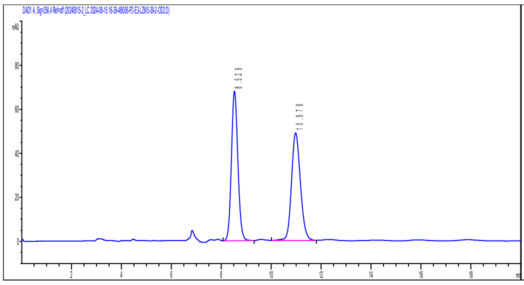

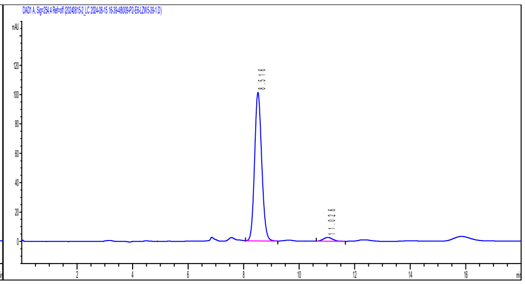

No.	Time	Area	Area (%)	No.	Time	Area	Area (%)
1	8.529	1220	50.167	1	8.516	1804.3	96.864
2	10.979	1211.9	49.833	2	11.026	58.4	3.136

4.5 General procedure for the synthesis of 78

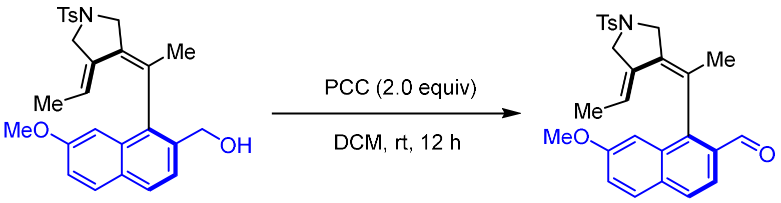

77 **78**

To a sealable tube (15 mL) were charged with **77** (0.1 mmol), PCC (0.2 mmol) and anhydrous dichloromethane (2.0 mL) under argon. And the mixture was stirred at r.t. for 12 h. The resulting mixture was diluted with dichloromethane and filtered through a celite pad and concentrated in vacuo. The resulting residue was purified by flash column chromatography on silica gel (petroleum ether/EtOAc = 5/1) to o afford the desired product **78** (41.0 mg, 89% yield). The enantiomeric excess was determined by chiral HPLC analysis. **^{1}H NMR (600 MHz, Chloroform-d) *δ*** 9.92 (s, 1H), 7.83 – 7.74 (m, 5H), 7.44 – 7.38 (m, 2H), 7.31 – 7.28 (m, 1H), 7.10 – 7.07 (m, 1H), 4.44 (d, *J* = 15.5, 1H), 4.29 (q, *J* = 7.2, 1H), 4.19 (d, *J* = 13.9, 1H), 4.06 – 4.00 (m, 1H), 3.86 (s, 3H), 3.84 – 3.80 (m, 1H), 2.49 (s, 3H), 2.11 (s, 3H), 1.21 (d, *J* = 7.2, 3H). **^{13}C NMR (150 MHz, Chloroform-d) *δ*** 192.2, 158.9, 146.2, 144.3, 133.6, 132.9, 132.8, 132.3, 131.3, 130.3, 130.1, 129.6, 127.98, 127.96, 124.0, 122.6, 121.4, 120.6, 104.4, 55.6, 52.9, 51.7, 24.6, 21.8, 15.7. **$[\alpha]_D^{20}$** = +82(c = 0.1, $CHCl_3$). **HRMS (ESI):** calcd. for: $C_{27}H_{27}NNaO_4S^+$ [M+Na]$^+$: 484.1553; found: 484.1545; **$[\alpha]_D^{20}$** = +36(c = 0.1, $CHCl_3$).

HPLC analysis: IE column (hexane:2-propanol = 85:15, v = 1.0 mL/min, 40 °C, 227 nm; tr (major) = 42.97 min, tr (minor) =53.653 min, 96% ee.

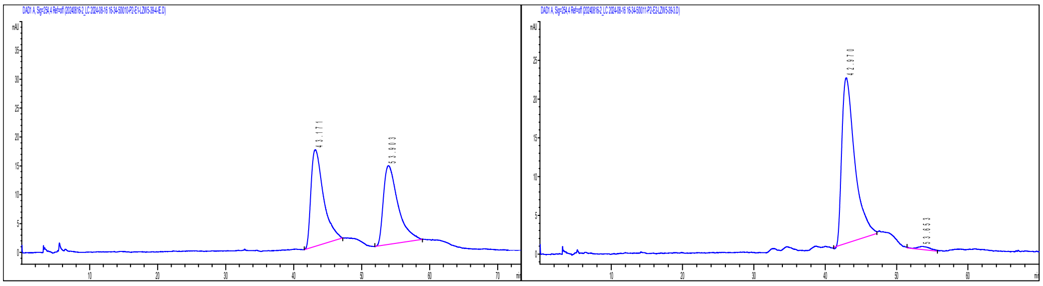

No.	Time	Area	Area (%)	No.	Time	Area	Area (%)
1	43.171	2144	51.344	1	42.97	2708.2	98.07
2	53.903	2031.7	48.656	2	53.653	53.3	1.93

4.6 General procedure for the synthesis of 79

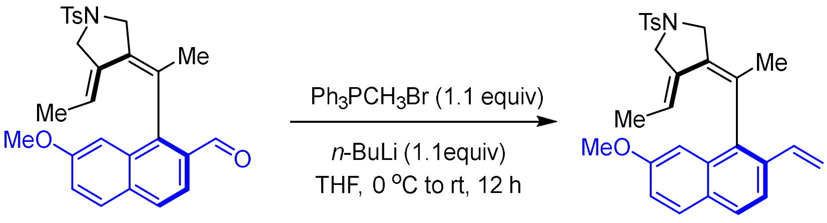

78 **79**

To a sealable tube (8 mL) were charged with Ph_3PCH_3Br (0.11 mmol) and THF (2.0 mL) under argon at 0 °C. *n*-BuLi (2.50 M, 0.11 mmol) was addeded and the mixture was stirred at 0 °C for 1 h. Then Aldehyde **78** (26.2 mg, 0.10 mmol) in THF (1 mL) was added. The mixture warmed to room temperature for another 12 h. The mixture was quenched with saturated aqueous ammonium chloride solution, extracted with DCM (3 x 5 mL), filtered and concentrated. The residue was purified by flash chromatography on silica gel (petroleum ether/EtOAc 5/1) to afford the desired product **79** as a white solid (34.4 mg, 75% yield). The enantiomeric excess was determined by chiral HPLC analysis. **^{1}H NMR (600 MHz, Chloroform-d) *δ*** 7.83 – 7.79 (m, 2H), 7.73 – 7.69 (m, 1H), 7.67 – 7.64 (m, 1H), 7.57 – 7.51 (m, 1H), 7.42 – 7.38 (m, 2H), 7.13 – 7.08 (m, 1H), 6.93 – 6.90 (m, 1H), 6.72 – 6.64 (m, 1H), 5.76 (d, *J* = 18.5, 1H), 5.23 (d, *J* = 10.9, 1H), 4.44 (q, *J* = 7.0, 1H), 4.34 – 4.29 (m, 1H), 4.27 – 4.21 (m, 1H), 3.94 – 3.90 (m, 2H), 3.79 (s, 3H), 2.48 (s, 3H), 1.96 (s, 3H), 1.21 (d, *J* = 7.2, 3H). **^{13}C NMR (151 MHz, Chloroform-d) *δ*** 158.3, 143.9, 137.2, 134.7, 133.1, 132.9, 131.5, 131.4, 131.2, 129.9, 129.8, 129.1, 128.1, 127.2, 127.1, 121.0, 120.7, 117.8, 115.4, 104.5, 55.4, 52.8, 51.6, 23.1, 21.7, 15.6. **HRMS (ESI):** calcd. for: $C_{28}H_{29}NNaO_3S^+$ [M+Na]$^+$: 482.1760; found: 482.1769; **$[\alpha]_D^{20}$** = +36 (c = 0.1, $CHCl_3$).

HPLC analysis: IA column (hexane:2-propanol = 98:2, v = 1.0 mL/min, 40 °C, 254 nm; tr (major) = 18.515 min, tr (minor) = 21.216 min, 94% ee.

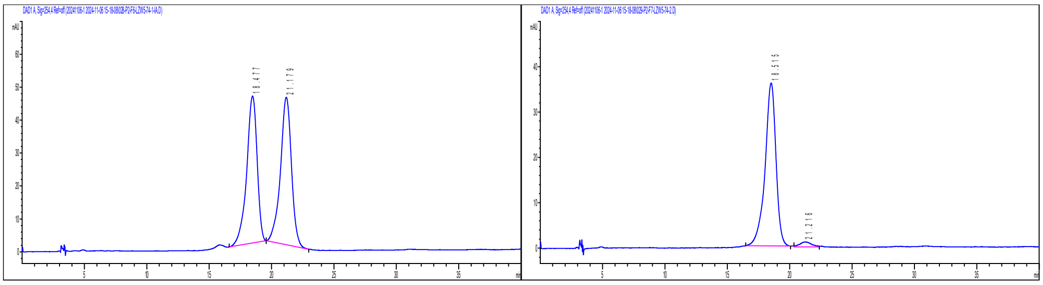

No.	Time	Area	Area (%)	No.	Time	Area	Area (%)
1	18.477	2603.2	48.584	1	18.515	2176.8	96.912
2	21.179	2754.9	51.416	2	21.216	69.4	3.088

4.7 General procedure for the synthesis of 80

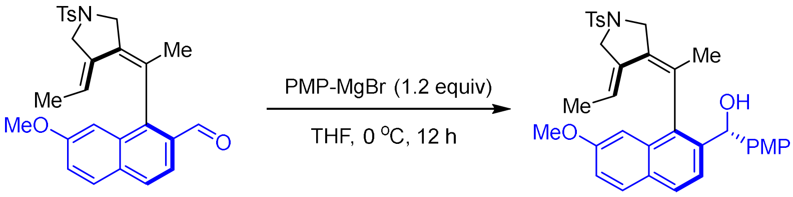

78 **80**

To a sealable tube (15 mL) were charged with **78** (0.1 mmol), THF (2.0 mL) under argon at 0 °C, Then PMP-MgBr (0.12 mmol, 1N in THF) was addeded and the mixture was stirred at 0 °C for 12 h. After that, the reaction mixture was quenched with H_2O and the mixture was concentrated under vacuum. The residue was purified by flash chromatography on silica gel (petroleum ether/EtOAc = 5/1) to afford the desired product **80** (37.5 mg, 66% yield). **^{1}H NMR (600 MHz, Chloroform-d) *δ*** 7.83 – 7.79 (m, 2H), 7.76 – 7.70 (m, 2H), 7.46 – 7.42 (m, 1H), 7.41 – 7.37 (m, 2H), 7.15 (dd, *J* = 8.9, 2.6, 1H), 7.13 – 7.10 (m, 2H), 6.90 – 6.87 (m, 1H), 6.85 – 6.79 (m, 2H), 5.74 (s, 1H), 4.54 (q, *J* = 7.1, 1H), 4.26 (d, *J* = 12.1, 1H), 4.16 (d, *J* = 12.3, 1H), 4.04 – 3.94 (m, 2H), 3.83 (s, 3H), 3.79 (s, 3H), 2.44 (s, 3H), 1.71 (s, 3H), 1.29 (d, *J* = 7.2, 3H). **^{13}C NMR (150 MHz, Chloroform-d) *δ*** 159.1, 158.3, 144.1, 137.3, 136.6, 135.6, 134.0, 133.0, 131.3, 131.2, 130.03, 129.99, 129.0, 128.2, 128.0, 127.9, 127.8, 122.8, 121.0, 118.1, 113.9, 104.3, 73.1, 55.5, 55.4, 52.8, 51.8, 23.1, 21.7, 15.6. **HRMS (ESI):** calcd. for: $C_{34}H_{35}NNaO_5S^+$ $[M+Na]^+$: 592.2128; found: 592.2121; **$[\alpha]_D^{20}$** = +90 (c = 0.1, $CHCl_3$).

HPLC analysis: IA column (hexane:2-propanol = 80:20, v = 1.0 mL/min, 40 °C, 227 nm; tr (major) = 16.226 min, tr (minor) = 32.635 min, 95% ee.

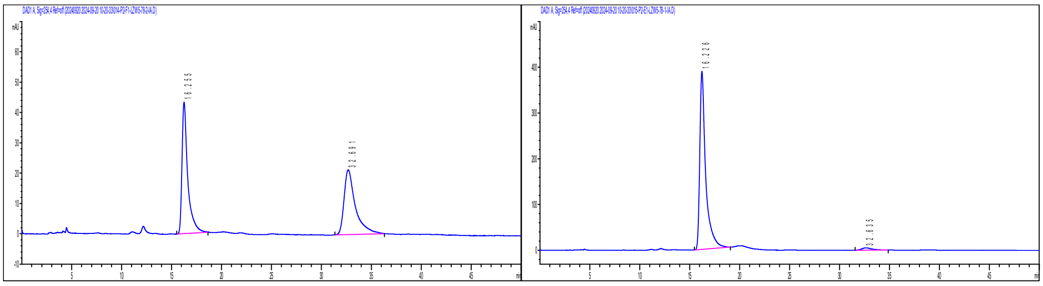

No.	Time	Area	Area (%)	No.	Time	Area	Area (%)
1	16.255	1743	50.818	1	16.226	16038.5	97.743
2	32.691	1686.9	49.182	2	32.635	370.4	2.257

4.8 General procedure for the synthesis of 81

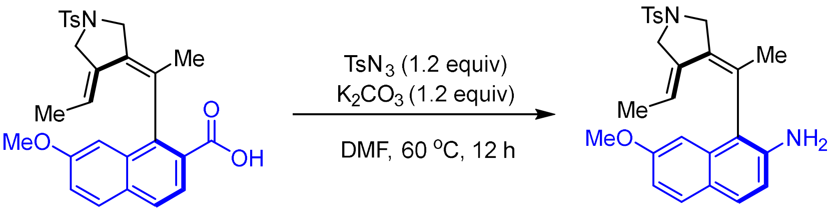

76 **81**

To a sealable tube (8 mL) were charged with **76** (0.10 mmol), TsN_3 (0.12 mmol), K_2CO_3 (0.12 mmol) and anhydrous DMF (2.0 mL) under air. And the mixture was stirred at 60°C for 12 h. The mixture was diluted with water, extracted with EtOAc (4 mL × 3) and filtered through a celite pad and concentrated in vacuo. The resulting residue was purified by flash column chromatography on silica gel (hexane/ EtOAc = 2:1) to o afford the desired product **81** (31.4 mg, 70% yield). **^{1}H NMR (600 MHz, Chloroform-d) *δ*** 7.83 – 7.78 (m, 2H), 7.60 (d, *J*=8.9, 1H), 7.51 (d, *J*=8.4, 1H), 7.39 (d, *J*=7.9, 2H), 6.90 (dd, *J*=8.8, 2.5, 1H), 6.78 (d, *J*=8.6, 1H), 6.68 (d, *J*=2.7, 1H), 4.87 (q, *J*=6.7, 1H), 4.28 (d, *J*=13.6, 1H), 4.20 (d, *J*=15.2, 1H), 4.04 – 3.97 (m, 1H), 3.92 – 3.86 (m, 1H), 3.80 (s, 3H), 2.47 (s, 3H), 1.96 (s, 3H), 1.29 (d, *J*=7.2, 3H).**^{13}C NMR (150 MHz, Chloroform-d) *δ*** 158.6, 143.9, 139.6, 133.1, 132.9, 132.6, 132.1, 130.0, 129.9, 128.3, 128.1, 125.6, 123.9, 120.4, 118.8, 115.8, 113.9, 102.9, 55.4, 52.9, 51.7, 21.7, 21.4, 15.7.. **HRMS (ESI):** calcd. for: $C_{26}H_{28}N_2NaO_3S^+$ [M+Na]$^+$: 471.1713; found: 471.1710; **$[\alpha]_D^{20}$** = +80 (c = 0.1, $CHCl_3$).

HPLC analysis: IE column (hexane:2-propanol = 80:20, v = 1.0 mL/min, 40 °C, 227 nm; tr (major) = 31.228 min, tr (minor) = 38.528 min, 93% ee.

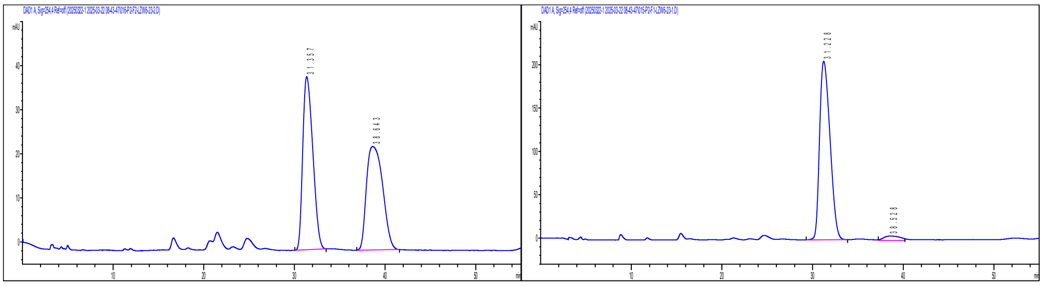

No.	Time	Area	Area (%)	No.	Time	Area	Area (%)
1	31.357	3022.9	50.899	1	31.228	16462.8	96.272
2	38.643	2916.1	49.101	2	38.528	637.5	3.728

4.9 General procedure for the synthesis of 82

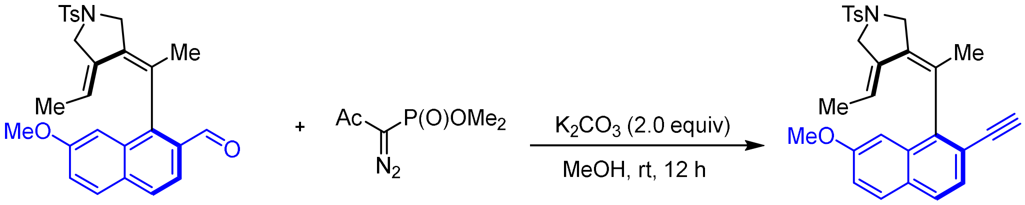

78 82

To a sealable tube (8 mL) were charged with aldehyde **78** (0.10 mmol) Bestmann reagent (0.12 mmol) in MeOH (1 mL) was was stirred at r.t. for 30min. K_2CO_3 (0.20 mmol) was addeded and the mixture was stirred at r.t. for 12 h. The reaction was then diluted with EA (3 x 5 mL) and quenched with sat. $NaHCO_3$ (10 mL). The aqueous layer was extracted with EA (3 x 10 mL). The combined organic phase was dried over Na_2SO_4, and concentrated in vacuo. The residue was purified by flash chromatography on silica gel (petroleum ether/EtOAc 5/1) to afford the desired product **82** as a white solid (34.3mg, 75% yield). **^{1}H NMR (600 MHz, Chloroform-d) *δ*** 7.83 – 7.78 (m, 2H), 7.74 – 7.68 (m, 1H), 7.65 – 7.60 (m, 1H), 7.40 – 7.35 (m, 3H), 7.18 – 7.13 (m, 1H), 6.95 – 6.92 (m, 1H), 4.41 – 4.32 (m, 2H), 4.16 (d, *J* = 11.8, 1H), 3.97 (d, *J* = 14.1, 1H), 3.91 – 3.84 (m, 1H), 3.80 (s, 3H), 3.00 (s, 1H), 2.47 (s, 3H), 2.03 (s, 3H), 1.21 (d, *J* = 7.2, 3H). **^{13}C NMR (150 MHz, Chloroform-d) *δ*** 158.6, 143.8, 143.4, 133.2, 133.0, 131.4, 131.1, 130.0, 129.9, 129.1, 128.1, 127.22, 127.20, 127.0, 120.8, 119.2, 117.8, 103.9, 83.0, 80.0, 55.5, 52.8, 51.4, 22.1, 21.7, 15.6. **HRMS (ESI):** calcd. for: $C_{28}H_{27}NNaO_3S^+$ [M+Na]$^+$: 480.1604; found: 480.1600; **$[\alpha]_D^{20}$** = +136 (c = 0.1, $CHCl_3$).

HPLC analysis: IG column (hexane:2-propanol = 85:15, v = 1.0 mL/min, 40 °C, 254 nm; tr (major) = 11.630 min, tr (minor) = 12.803 min, 92% ee.

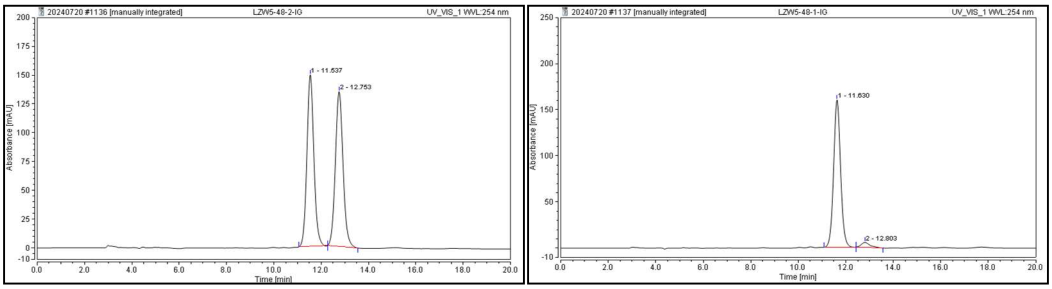

No.	Time	Area	Area (%)	No.	Time	Area	Area (%)
1	11.537	50.668	50.00	1	11.630	54.281	95.95
2	12.753	50.659	50.00	2	12.803	2.291	4.05

4.10 Synthesis of 83

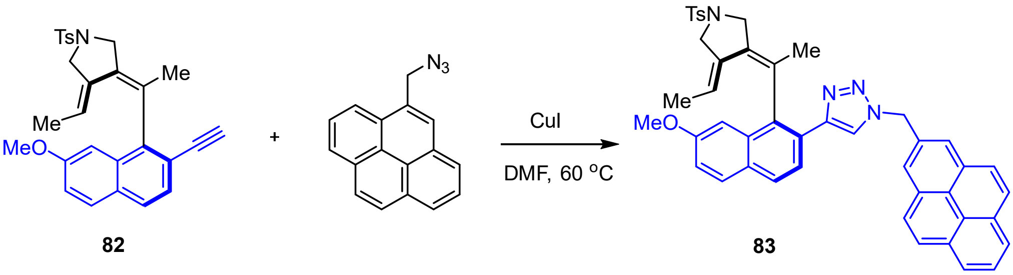

To a sealable tube (8 mL) were charged with **82** (0.10 mmol), 1- (azidomethyl)pyrene (0.20 mmol), CuI (0.02 mmol) and anhydrous DMF (2.0 mL) under air. And the mixture was stirred at 60°C for 12 h. The mixture was diluted with water, extracted with EtOAc (4 mL × 3) and filtered through a celite pad and concentrated in vacuo. The resulting residue was purified by flash column chromatography on silica gel (hexane/ EtOAc = 1:1) to o afford the desired product **83** (51.6 mg, 72% yield). **^{1}H NMR (600 MHz, Chloroform-d) *δ*** 8.34 – 8.23 (m, 5H), 8.21 – 8.13 (m, 3H), 8.07 – 7.99 (m, 3H), 7.78 – 7.73 (m, 2H), 7.72 – 7.67 (m, 2H), 7.40 (s, 1H), 7.33 (d, *J* = 8.3, 2H), 7.07 (dd, *J* = 8.8, 2.6, 1H), 6.73 (d, *J* = 2.8, 1H), 6.28 (d, *J* = 5.0, 2H), 4.39 (q, *J* = 7.1, 1H), 3.91 (d, *J* = 13.7, 1H), 3.83 (d, *J* = 12.3, 1H), 3.66 – 3.52 (m, 5H), 2.41 (s, 3H), 1.53 (s, 3H), 0.86 (d, *J* = 7.2, 3H). **^{13}C NMR (150 MHz, Chloroform-d) *δ*** 158.2, 146.2, 143.9, 135.6, 133.0, 132.4, 132.3, 131.8, 131.4, 131.0, 130.7, 130.0, 129.9, 129.3, 129.2, 129.1, 128.5, 128.1, 128.0, 127.6, 127.5, 127.3, 126.5, 126.1, 125.9, 125.5, 125.4, 125.2, 124.6, 124.1, 122.1, 122.06, 121.1, 117.8, 104.3, 55.2, 52.5, 52.4, 51.2, 22.2, 21.7, 15.3. **HRMS (ESI):** calcd. for: $C_{45}H_{38}N_4NaO_3S^+$ $[M+Na]^+$: 737.2557; found: 737.2563; **$[\alpha]_D^{20}$** = +44 (c = 0.1, $CHCl_3$).

HPLC analysis: ID column (hexane:2-propanol = 50:50, v = 1.0 mL/min, 40 °C, 254 nm;

tr (minor) = 40.027 min, tr (major) = 43.693 min, 92% ee.

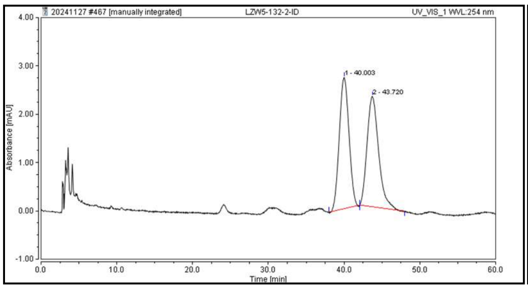

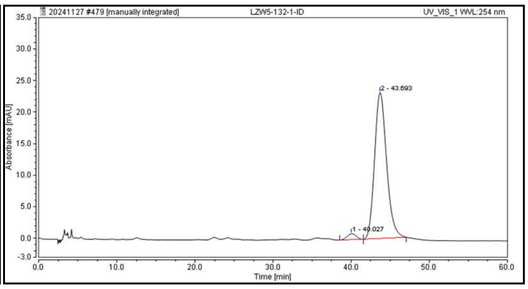

No.	Time	Area	Area (%)	No.	Time	Area	Area (%)
1	40.003	4.016	49.92	1	40.027	1.277	3.98
2	43.720	4.029	50.08	2	43.693	41.144	96.02

4.11 Synthesis of 84

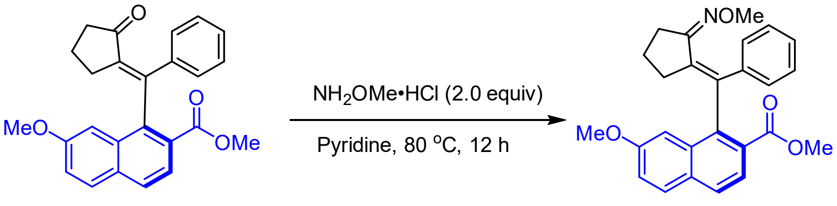

44 **84**

To a sealable tube (8 mL) were charged with **44** (0.10 mmol), Methoxyamine hydrochloride (0.20 mmol) and anhydrous pyridine (2.0 mL) under argon. And the mixture was stirred at 80 °C for 12 h. After that, the reaction mixture was concentrated under vacuum. The residue was purified by flash chromatography on silica gel (petroleum ether/EtOAc = 20/1) to afford the desired product **84** (37.0 mg, 89% yield). The enantiomeric excess was determined by chiral HPLC analysis. **^{1}H NMR (600 MHz, Chloroform-d) *δ*** 7.79 – 7.71 (m, 3H), 7.52 – 7.49 (m, 1H), 7.45 – 7.40 (m, 2H), 7.23 – 7.19 (m, 1H), 7.18 – 7.10 (m, 3H), 3.84 – 3.79 (m, 6H), 3.72 (s, 3H), 2.67 – 2.62 (m, 2H), 2.22 – 2.13 (m, 1H), 2.04 – 1.96 (m, 1H), 1.77 – 1.67 (m, 1H), 1.67 – 1.60 (m, 1H). **^{13}C NMR (150 MHz, Chloroform-d) *δ*** 168.6, 159.8, 158.8, 141.5, 140.0, 135.6, 135.4, 132.5, 131.0, 130.2, 129.8, 127.9, 127.4, 127.13, 127.05, 123.9, 120.4, 105.5, 61.9, 55.6, 52.3, 34.1, 29.0, 21.9. **HRMS (ESI):** calcd. for: $C_{26}H_{26}NO_4^+$ [M+H]$^+$: 416.1856; found: 416.1864; **$[\alpha]_D^{20}$** = +16 (c = 0.1, $CHCl_3$).

HPLC analysis: OZ column (hexane:2-propanol = 99:1, v = 0.65 mL/min, 40 °C, 254 nm; tr (major) = 10.257 min, tr (minor) = 11.467 min, 94% ee.

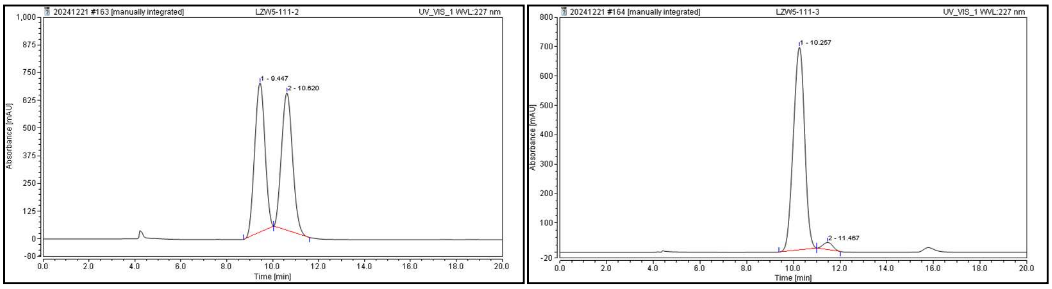

No.	Time	Area	Area (%)	No.	Time	Area	Area (%)
1	9.447	348.331	50.27	1	10.257	387.009	97.03
2	10.620	344.651	49.73	2	11.467	11.854	2.97

4.12 Synthesis of 85

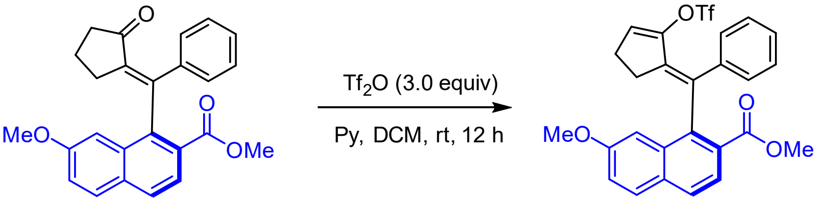

44 **85**

To a sealable tube (8 mL) were charged with **44** (0.1 mmol), DCM (2.0 mL) and pyridine (0.1 mmol) under argon at 0 °C. Tf_2O (0.30 mmol) was addeded and the mixture was stirred at r.t for 4 h. After that, the reaction mixture was concentrated under vacuum. The residue was purified by flash chromatography on silica gel (petroleum ether/EtOAc = 20/1) to afford the desired product **85** (33.1 mg, 64% yield). **^{1}H NMR (600 MHz, Chloroform-d) *δ*** 7.83 – 7.74 (m, 3H), 7.30 – 7.26 (m, 3H), 7.25 – 7.21 (m, 2H), 7.20 – 7.15 (m, 2H), 6.05 (t, *J* = 3.1, 2H), 3.76 (d, *J* = 8.2, 6H), 3.17 – 3.12 (m, 2H), 2.59 – 2.52 (m, 2H). **^{13}C NMR (150 MHz, Chloroform-d) *δ*** 168.1, 158.4, 149.4, 141.2, 138.6, 135.6, 133.7, 130.8, 130.6, 129.7, 129.0, 128.5, 128.0, 127.8, 124.7, 123.8, 120.2, 118.1 (q, *J* = 321.0), 105.8, 55.2, 52.1, 30.8, 26.5. **^{19}F NMR (376 MHz, Chloroform-d) *δ*** -73.8 (m). **HRMS (ESI):** calcd. for: $C_{26}H_{21}F_3NaO_6S^+$ [M+Na]$^+$: 541.0903; found: 541.0909; **$[\alpha]_D^{20}$** = +18 (c = 0.1, $CHCl_3$).

HPLC analysis: IC column (hexane:2-propanol = 98:2, v = 1.0 mL/min, 40 °C, 254 nm; tr (minor) = 5.253 min, tr (major) = 6.209 min, 90% ee.

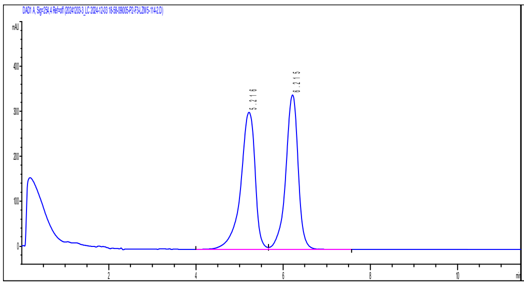

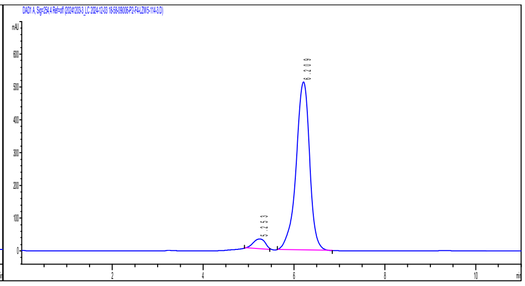

No.	Time	Area	Area (%)	No.	Time	Area	Area (%)
1	5.216	7028.3	50.833	1	5.253	542	4.876
2	6.215	6798	49.167	2	6.209	10573	95.124

4.13 Synthesis of 86

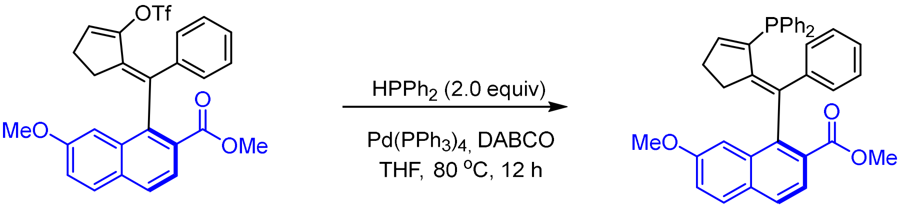

85 **86**

To a sealable tube (15 mL) were charged with **85** (0.1 mmol), $Pd(PPh_3)_4$ (0.05 mmol), DBACO (0.20 mmol) and $HPPh_2$ (0.20 mmol)in anhydrous THF (2.0 mL) under argon. And the mixture was stirred at 80 °C for 12 h. After that, the reaction mixture was concentrated under vacuum. The residue was purified by flash chromatography on silica gel (petroleum ether/EtOAc = 15/1) to afford the desired product **86** as a yellow solid (28.7 mg, 53% yield). The enantiomeric excess was determined by chiral HPLC analysis. **^{1}H NMR (600 MHz, Chloroform-d) *δ*** 7.85 – 7.80 (m, 1H), 7.78 – 7.73 (m, 1H), 7.69 – 7.63 (m, 1H), 7.32 – 7.27 (m, 2H), 7.22 – 7.15 (m, 5H), 7.15 – 7.05 (m, 2H), 7.05 – 6.96 (m, 5H), 6.80 – 6.76 (m, 1H), 6.67 – 6.61 (m, 2H), 5.66 (q, *J* = 2.8, 1H), 3.58 (s, 3H), 3.44 (s, 3H), 3.24 – 3.15 (m, 1H), 3.14 – 3.07 (m, 1H), 2.49 – 2.39 (m, 2H). **^{13}C NMR (150 MHz, Chloroform-d) *δ*** 168.5, 157.7, 148.1, 146.8 (d, *J* = 14.1), 145.3 (d, *J* = 24.0), 142.9, 139.6 (d, *J* = 3.7), 137.6 (d, *J* = 15.2), 136.6 (d, *J* = 13.6), 134.8 (d, *J* = 4.7), 133.7, 133.59, 133.57, 133.4, 130.8 (d, *J* = 6.6), 130.4 (d, *J* = 78.9), 129.5, 129.1, 128.3, 128.2, 128.1, 128.0, 127.92, 127.88, 127.8, 127.6, 126.3, 124.0, 120.0, 105.9, 54.7, 51.8, 35.8, 32.2 (d, *J* = 2.2). **^{31}P NMR (243 MHz, Chloroform-d) *δ*** -18.8. **HRMS (ESI):** calcd. for: $C_{37}H_{31}NaO_3P^+$ $[M+Na]^+$: 577.1903; found: 577.1906; **$[\alpha]_D^{20}$** = +150 (c = 0.1, $CHCl_3$).

HPLC analysis: IC column (hexane:2-propanol = 98:2, v = 1.0 mL/min, 40 °C, 227 nm; tr (major) = 7.469 min, tr (minor) = 8.76 min, 90% ee.

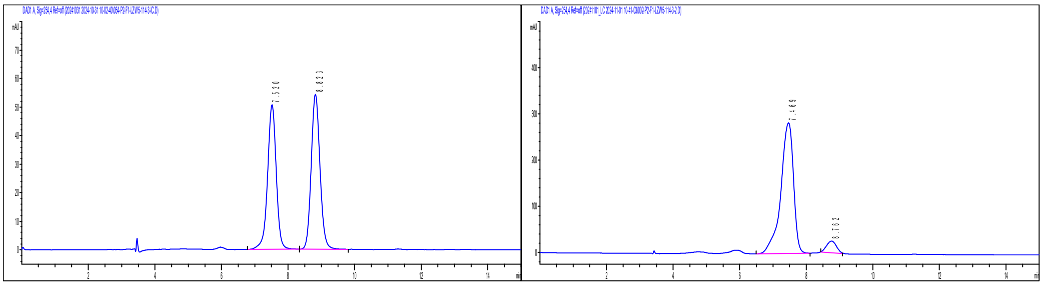

No.	Time	Area	Area (%)	No.	Time	Area	Area (%)
1	7.52	919.9	48.103	1	7.469	7465.9	95.056
2	8.823	992.5	51.897	2	8.76	388.3	4.944

4.14 Synthesis of 87

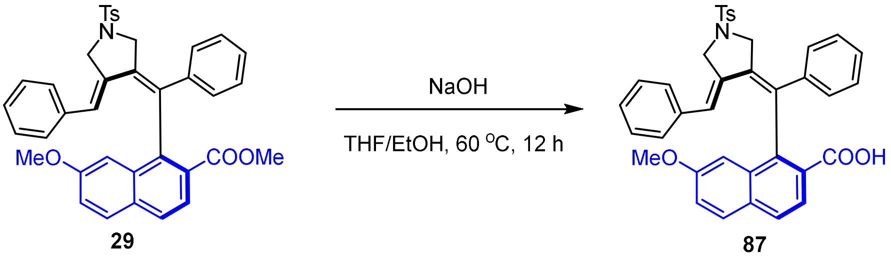

To a mixture of NaOH (0.50 mmol, 5.0 equiv.) in 2.0 mL THF and 2.0 mL EtOH was added **29** (0.10 mmol, 1.0 equiv.), The resulting mixture was stirred for 24 h at 60 °C. After that, the mixture was diluted with water and added hydrochloric acid to adjust PH, extracted with EtOAc (4 mL × 3) and concentrated in vacuo. The residue was purified by flash chromatography on silica gel (petroleum ether/EtOAc = 1/3 to 1/2) to afford the desired product **87** as a yellow solid (48.7 mg, 81% yield). The enantiomeric excess was determined by chiral HPLC analysis. **^{1}H NMR (600 MHz, Chloroform-d) *δ*** 7.92 – 7.87 (m, 1H), 7.83 – 7.77 (m, 1H), 7.77 – 7.73 (m, 3H), 7.43 – 7.39 (m, 1H), 7.36 – 7.31 (m, 2H), 7.24 – 7.18 (m, 4H), 7.15 – 7.11 (m, 2H), 7.10 – 7.05 (m, 3H), 6.63 – 6.58 (m, 2H), 5.67 (t, *J* = 2.6, 1H), 4.44 (d, *J* = 12.5, 1H), 4.29 (dd, *J* = 14.0, 2.5, 1H), 4.18 (d, *J* = 12.5, 1H), 4.00 (dd, *J* = 14.0, 2.6, 1H), 3.75 (s, 3H), 2.42 (s, 3H). **^{13}C NMR (150 MHz, Chloroform-d) *δ*** 171.1, 159.1, 144.0, 141.1, 140.8, 136.5, 134.44, 134.41, 134.2, 132.8, 132.7, 131.5, 130.0, 129.8, 129.1, 128.5, 128.4, 128.2, 128.1, 128.0, 127.9, 127.6, 127.4, 124.5, 121.4, 104.7, 55.5, 52.5, 51.1, 21.7. **HRMS (ESI):** calcd. for: $C_{37}H_{31}NNaO_5S^+$ [M+Na]$^+$: 624.1815; found: 624.1812; **$[\alpha]_D^{20}$** = +120 (c = 0.1, $CHCl_3$).

HPLC analysis: AD-H column (hexane:2-propanol = 70:30, v = 1.0 mL/min, 40 °C, 227 nm; tr (minor) = 29.115 min, tr (major) = 36.876 min, 95% ee.

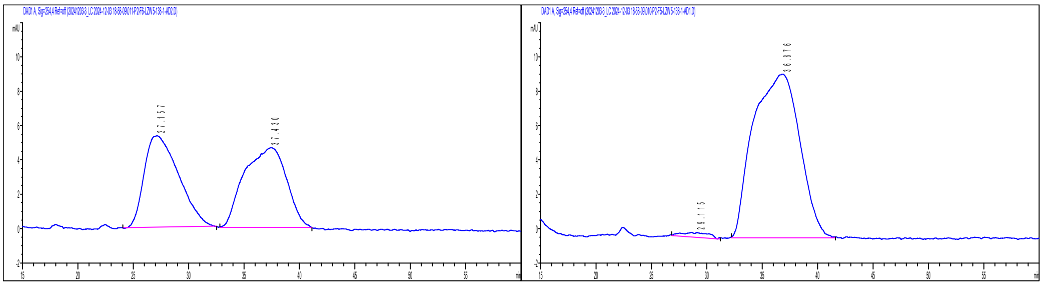

No.	Time	Area	Area (%)	No.	Time	Area	Area (%)
1	27.157	1148.5	48.382	1	29.115	52.1	1.837
2	37.43	1225.3	51.618	2	36.876	2784.4	98.163

4.15 Synthesis of 88

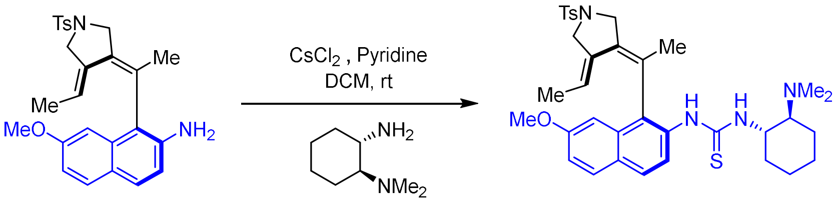

81 **88**

To a sealable tube (8 mL) were charged with **81** (0.1 mmol), $CSCl_2$ (2.0 equiv), Pyridine (3.0 equiv), and DCM (2.0 mL) at r.t. Then the mixture was stirred at r.t. for 0.5 h. the mixture was directly purified by quick column chromatography on silica gel (eluent: PE/EtOAc = 10:1) to afford the desired product. The above product was dissolved in DCM (2 mL), and (1*S*,2*S*)-*N*,*N*-dimethylcyclohexane-1,2-diamine (0.20 mmol) was added. The mixture was stirred at r.t. for 1 h. Upon completion, the mixture was directly purified by column chromatography on silica gel (DCM/MeOH = 15:1) to afford the desired product **88** (40.0 mg, 63% yield). **^{1}H NMR (600 MHz, Chloroform-d) *δ*** ^{1}H NMR (600 MHz, $CDCl_3$) δ 7.82 – 7.78 (m, 2H), 7.76 – 7.69 (m, 2H), 7.43 – 7.39 (m, 2H), 7.37 – 7.32 (m, 1H), 7.16 (dd, *J*=8.9, 2.6, 1H), 6.93 (d, *J*=2.7, 1H), 4.50 – 4.36 (m, 2H), 4.15 – 4.01 (m, 2H), 3.83 (s, 3H), 3.81 – 3.74 (m, 2H), 2.74 – 2.69 (m, 1H), 2.47 (s, 3H), 2.12 – 2.04 (m, 6H), 1.96 (s, 3H), 1.84 – 1.77 (m, 2H), 1.69 (d, *J*=15.7, 1H), 1.38 – 1.31 (m, 2H), 1.24 – 1.22 (m, 4H), 1.21 – 1.13 (m, 2H), 1.09 – 1.02 (m, 1H).

^{13}C NMR (150 MHz, Chloroform-d) *δ* 180.8, 158.8, 144.1, 133.2, 133.2, 132.63, 132.59, 132.0, 130.1, 130.0, 128.3, 128.0, 127.6, 124.7, 121.7, 120.9, 118.5, 103.9, 66.8, 56.4, 55.5, 52.8, 51.5, 39.8, 32.6, 25.3, 24.6, 22.1, 21.7, 21.5, 15.6.

HRMS (ESI): calcd. for: $C_{35}H_{44}N_4NaO_3S_2^+$ $[M+Na]^+$: 655.2747; found: 655.2751; **$[\alpha]_D^{20}$** = +100 (c = 0.1, $CHCl_3$).

4.16 Synthesis of 91

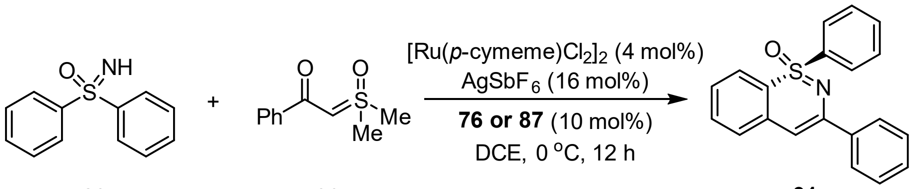

89 **90** **91**

To a sealable tube (8 mL) were charged with sulfoximines (0.10 mmol), sulfur ylide (0.15 mmol), **76** or **87** (0.01 mmol), $AgSbF_6$ (0.016 mmol), [Ru(*p*-cymeme)Cl_2]$_2$ (0.004 mmol) and DCE (1.0 mL). And the mixture was stirred at 0 ^oC for 12 h under argon. After that, the reaction mixture was concentrated under vacuum. The residue was purified by flash chromatography on silica gel (petroleum ether/EtOAc = 8/1) to afford the desired product **91**. used **76**: (28.9 mg, 75% yield), used **87**: (31.6 mg, 82% yield). The enantiomeric excess was determined by chiral HPLC analysis. **^{1}H NMR (600 MHz, Chloroform-d) *δ*** 8.03 – 7.98 (m, 4H), 7.66 – 7.62 (m, 1H), 7.60 – 7.55 (m, 2H), 7.50 – 7.47 (m, 1H), 7.46 – 7.39 (m, 3H), 7.38 – 7.31 (m, 2H), 7.27 – 7.20 (m, 1H), 6.82 (s, 1H). **^{13}C NMR (150 MHz, Chloroform-d) *δ*** 147.3, 140.6, 138.9, 136.6, 133.5, 132.2, 129.5, 129.1, 128.9, 128.5, 127.0, 126.8, 126.4, 125.1, 119.8, 98.3. **HRMS (ESI):** calcd. for: $C_{20}H_{16}NOS^+$ [M+H]$^+$: 318.0947; found: 318.0954;

HPLC analysis: ID column (hexane:2-propanol = 85:15, v = 1.0 mL/min, 40 ^oC, 254 nm; tr (minor) = 14.400 min, tr (major) = 16.393 min, 57% ee or tr (minor) = 14.583 min, tr (major) = 16.640 min, 18% ee.

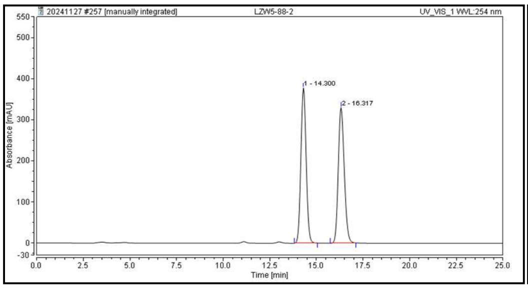

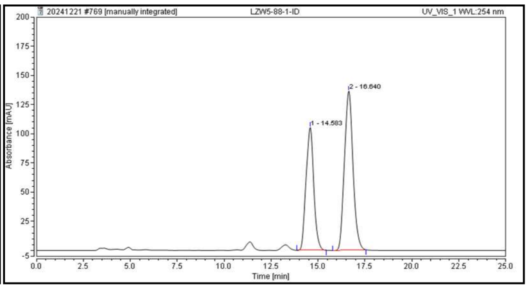

No.	Time	Area	Area (%)	No.	Time	Area	Area (%)
1	14.300	123.487	50.08	1	14.583	50.328	41.13
2	16.317	123.113	49.92	2	16.640	72.048	58.87

(Acid 76 was used)

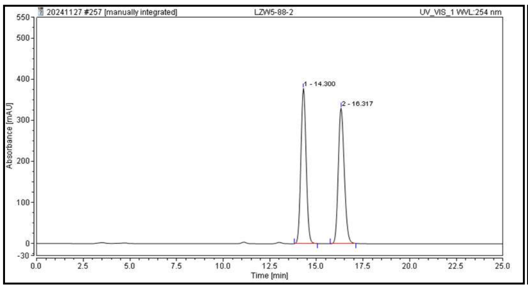

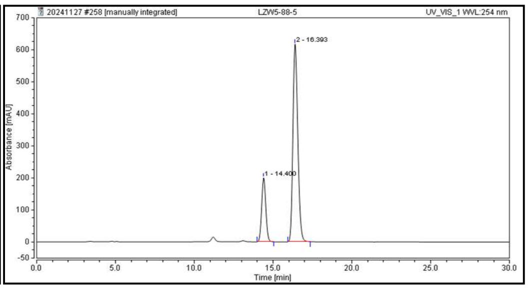

No.	Time	Area	Area (%)	No.	Time	Area	Area (%)
1	14.300	123.487	50.08	1	14.400	60.714	21.55
2	16.317	123.113	49.92	2	16.393	220.964	78.45

(Acid 87 was used)

4.17 Synthesis of 93

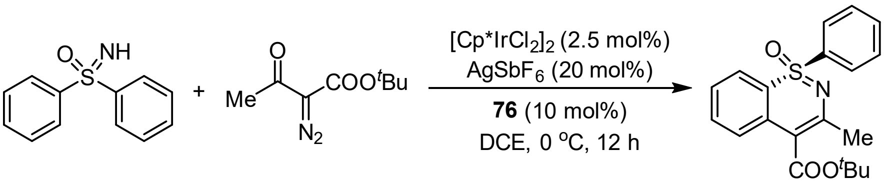

89 **92** **93**

To a sealable tube (8 mL) were charged with sulfoximines (0.10 mmol), diazoniums (0.15 mmol), **76** (0.01 mmol), $AgSbF_6$ (0.02 mmol), $[Cp^*IrCl_2]_2$ (0.0025 mmol) and DCE (1.0 mL). And the mixture was stirred at 0 °C for 12 h under argon. After that, the reaction mixture was concentrated under vacuum. The residue was purified by flash chromatography on silica gel (petroleum ether/EtOAc = 10/1) to afford the desired product **93** (29.1 mg, 82% yield). The enantiomeric excess was determined by chiral HPLC analysis. **^{1}H NMR (600 MHz, Chloroform-d) *δ*** 7.93 (d, *J* = 7.9, 2H), 7.71 – 7.63 (m, 2H), 7.62 – 7.55 (m, 2H), 7.54 – 7.48 (m, 1H), 7.30 – 7.24 (m, 1H), 7.24 – 7.20 (m, 1H), 2.46 (s, 3H), 1.64 (s, 9H). **^{13}C NMR (150 MHz, Chloroform-d) *δ*** 168.4, 150.1, 139.7, 134.2, 133.8, 132.5, 129.4, 129.2, 126.0, 125.2, 124.1, 118.0, 107.4, 81.7, 28.4, 24.8.

HRMS (ESI): calcd. for: $C_{20}H_{21}NNaO_3S^+$ [M+Na]$^+$: 378.1134; found: 378.1138;

HPLC analysis: ID column (hexane:2-propanol = 90:10, v = 1.0 mL/min, 40 °C, 254 nm; tr (major) = 14.287 min, tr (minor) = 16.223 min, 83% ee.

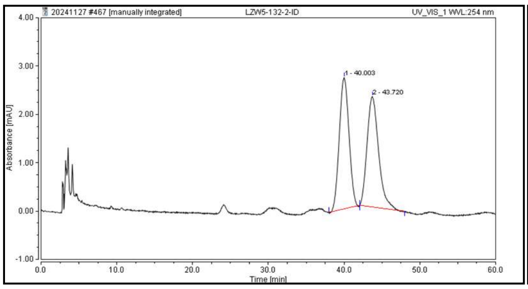

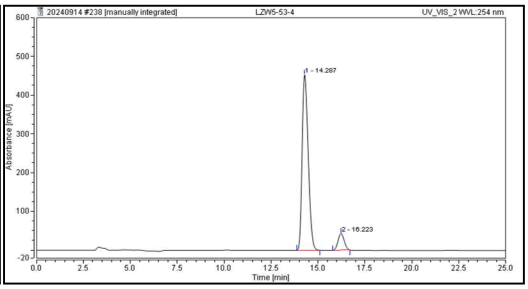

No.	Time	Area	Area (%)	No.	Time	Area	Area (%)
1	15.543	105.510	49.92	1	14.287	169.414	91.15
2	17.590	102.701	50.08	2	16.223	16.445	8.85

4.18 Synthesis of 96

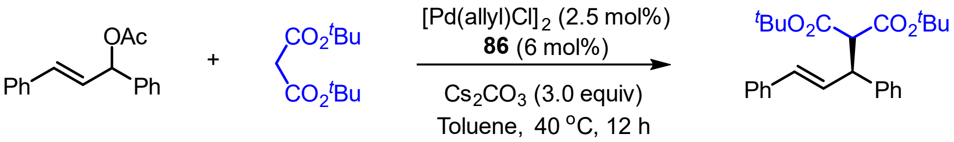

94 **95** **96**

To a suspension of the desired chiral ligand **(*R*)-86** (6 mol%), $[Pd(C_3H_5)Cl]_2$ (2.5 mol%) in Toluene (2.0 mL) were stirred at r.t. for 30 min under argon. Then the reaction solution was warmed to 40°C, 1,3-diphenyl-2-propenyl acetate (0.15 mmol), di-tert-butyl malonate (0.1 mmol) and Cs_2CO_3 (0.2 mmol) were subsequently added. The resulting mixture was kept at 40 °C for 12 h, the reaction mixture was diluted with EtOAc (5 mL). Saturated NH_4Cl (aq) (10 mL) was then added, the mixture was extracted with EtOAc (3 × 10 mL), and the extract was dried over $MgSO_4$. The residue was purified by flash column chromatography on silica gel (petroleum ether/EtOAc = 10/1) to afford the product **96** as a yellow oil (23.3 mg, 57% yield). **^{1}H NMR (600 MHz, Chloroform-d) *δ*** 7.32 – 7.28 (m, 6H), 7.27 – 7.24 (m, 2H), 7.22 – 7.15 (m, 2H), 6.47 – 6.42 (m, 1H), 6.37 – 6.30 (m, 1H), 4.19 – 4.12 (m, 1H), 3.76 – 3.71 (m, 1H), 1.42 (s, 9H), 1.22 (s, 9H). **^{13}C NMR (150 MHz, Chloroform-d) *δ*** 167.4, 166.9, 140.9, 137.2, 131.4, 130.3, 128.63, 128.58, 128.3, 127.5, 127.0, 126.4, 81.9, 81.7, 59.5, 49.2, 28.1, 27.7. **HRMS (ESI):** calcd. for: $C_{26}H_{32}NaNO_4^+$ $[M+Na]^+$: 431.2193; found: 431.2190;

HPLC analysis: IA column (hexane:2-propanol = 90:10, v = 1.0 mL/min, 40 °C,227 nm; tr (minor) = 4.899 min, tr (major) = 5.962 min, 81% ee

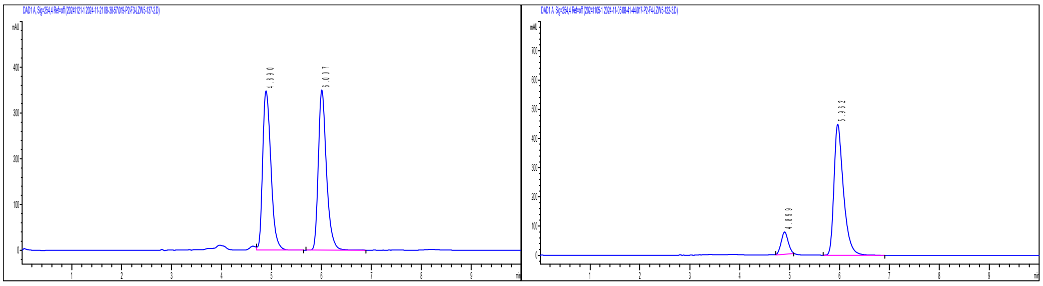

No.	Time	Area	Area (%)	No.	Time	Area	Area (%)
1	4.89	3989.2	50.126	1	4.899	621.4	9.538
2	6.007	3969.2	49.874	2	5.962	5893.4	90.462

4.19 Synthesis of 99

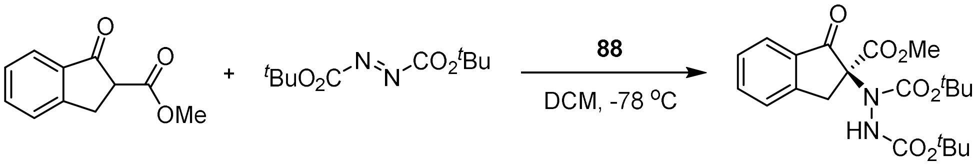

97 **98** **99**

Compound **99** was prepared in 92% yield (38.6 mg, 0.10 mmol scale) according to the known procedure with compound **88** (20:1 d.r.) as catalyst.[10] This compound is known and the spectroscopic data match those reported. **^{1}H NMR (600 MHz, Chloroform-d)** δ = 7.81 – 7.70 (m, 1H), 7.67 – 7.59 (m, 1H), 7.53 – 7.44 (m, 1H), 7.44 – 7.33 (m, 1H), 6.81 – 6.68 (m, 1H), 4.29 – 3.97 (m, 1H), 3.88 – 3.70 (m, 4H), 1.59 – 1.30 (m, 18H).

HPLC analysis: IE column (hexane:2-propanol = 80:20, v = 1.0 mL/min, 40 °C, 227 nm); tr (major) = 12.945 min, tr (minor) = 14.438 min, 90% ee.

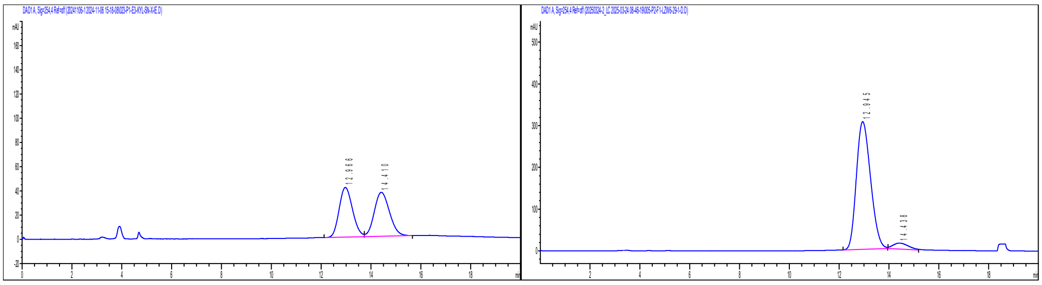

No.	Time	Area	Area (%)	No.	Time	Area	Area (%)
1	12.966	1535.3	49.887	1	12.945	12146	95.079
2	14.41	1542.3	50.113	2	14.438	628.6	4.921

5. Mechanistic Studies

5.1 Deuterium-labeling experiment of 3

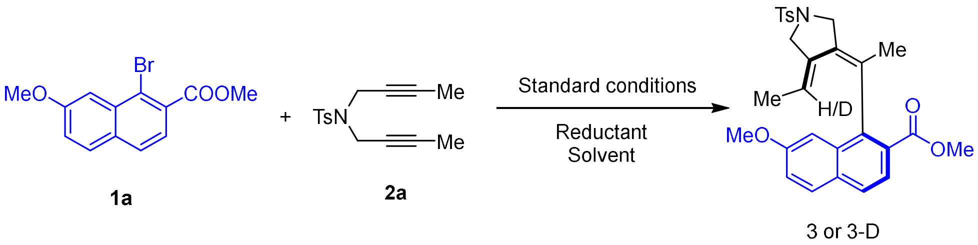

To a sealable tube (8 mL) were charged with aryl bromide (0.12 mmol), 1,6-diyne (0.10 mmol), $Pd(OAc)_2$ (8 mol%), **L10** (10 mol%), reductant (0.3 mmol), 15-crown-5 (0.1 mmol), and an anhydrous methanol (2.0 mL) under N_2. The resulting mixture was stirred for 24 h at 50 °C. After that, the reaction mixture was concentrated under vacuum.

The residue was purified by flash chromatography on silica gel (petroleum ether/EtOA = 5/1) to afford the desired product. The deuteration level was determined by ^{1}H NMR analysis.

Reductant	Solvent	Deuterated ratio / %
HCOOK	CD_3OD	0
HCOOK	CH_3OD	0
DCOONa	CH_3OH	>99
DCOONa	CD_3OD	>99
DCOONa	CH_3OD	>99

^{1}H NMR (600 MHz, Chloroform-d) spectrum of 3-D

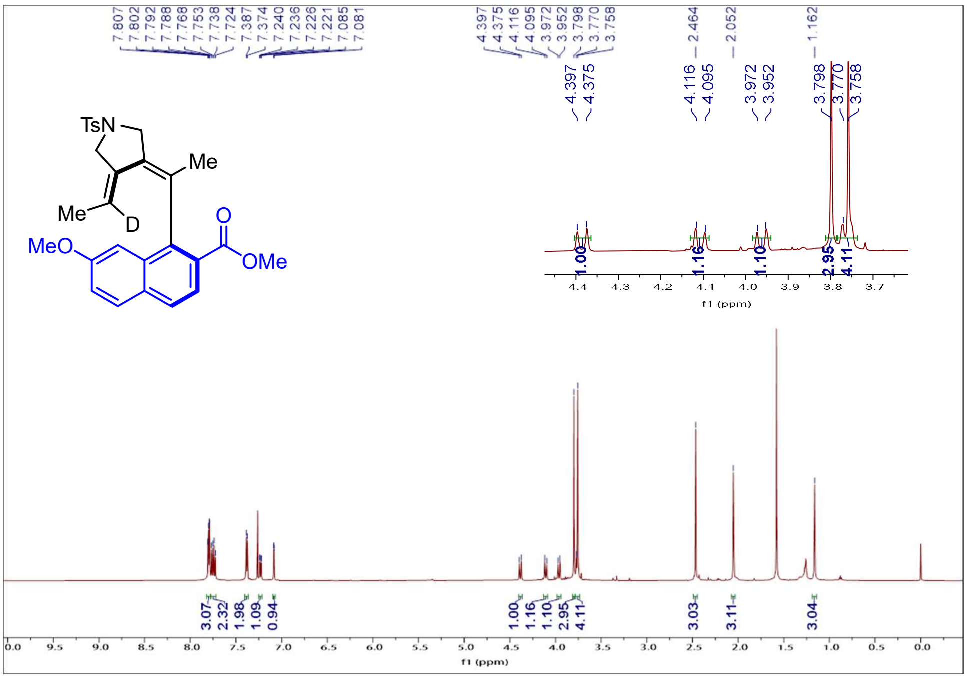

5.2 KIE Experiment

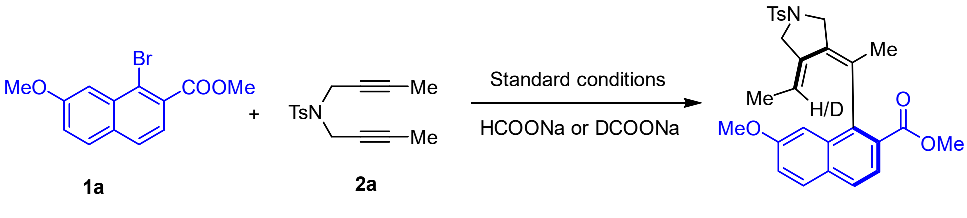

To a sealable tube (8 mL) were charged with aryl bromides (0.12 mmol), 1,6-diyne (0.10 mmol), $Pd(OAc)_2$ (8 mol%), **L10** (10 mol%), HCOONa or DCOONa (0.3 mmol), 15-Crown-5 (0.10 mmol), and anhydrous MeOH (2.0 mL) under N_2 at 50 °C, Aliquots of the reaction mixture (200 µL) were taken out via syringe at specific time. The mixture was transferred into an NMR tube, and analyzed by quantitative ^1H NMR using the signal of mesitylene (δ 7.46 ppm) as an internal standard ($CDCl_3$ as a deuterated solvent).

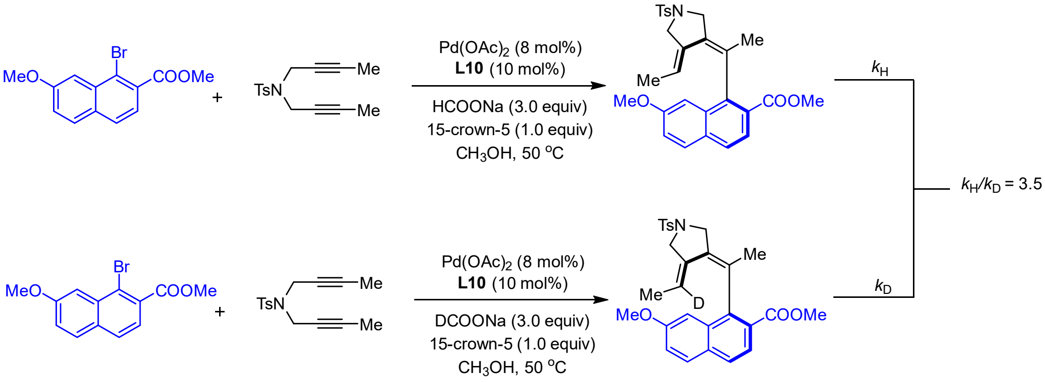

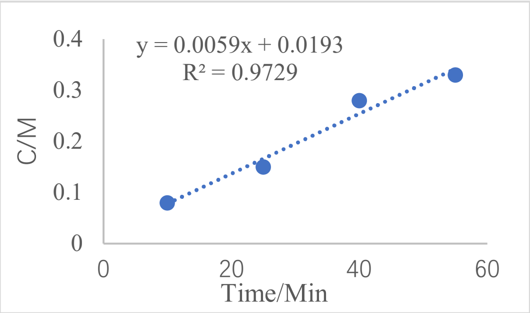

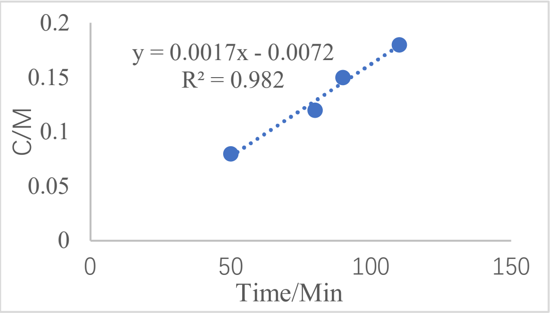

3 **3-D**

5.3 Non-Linear Effect of Chiral Ligand L10

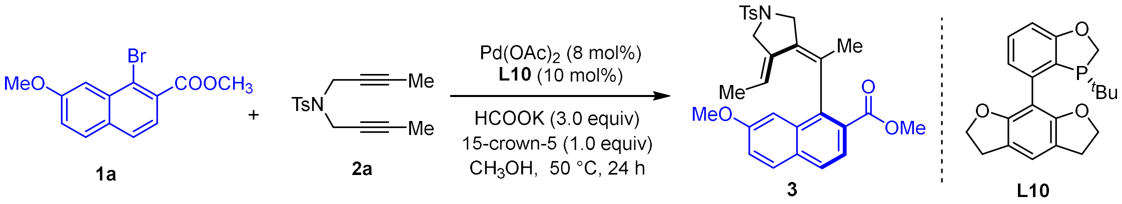

A screw-cap vial (8 mL) was charged with $Pd(OAc)_2$ (8.0 mol%), chiral ligand **L10** (10 mol%, 20% ee, 40% ee, 60% ee, 80% ee, 99% ee) and aryl bromides (0.12 mmol), 1,6-diyne (0.10 mmol), 15-crown-5 (0.10 mmol) and HCOOK (0.30 mmol) in MeOH (2 mL). The reaction mixtures were stirred at 50 °C for 24 h. The residue was purified by preparative TLC to afford the product's ee. The ee was determined by HPLC using a chiral stationary phase.

**L10** of ee (%)	**3** of ee (%)
0	0
20	13.9
40	32.9
60	54.0
80	74.3
99	95.1

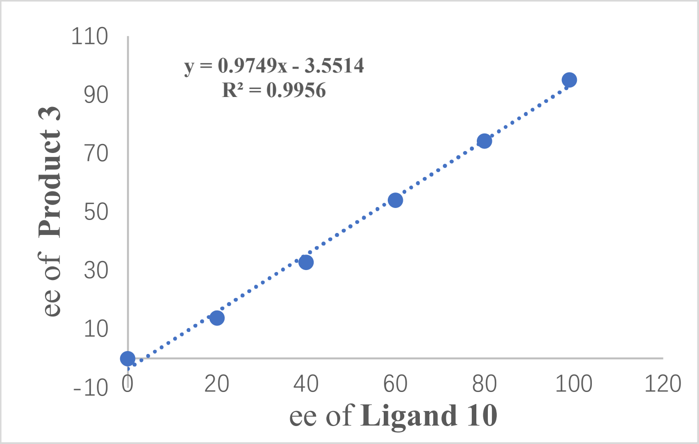

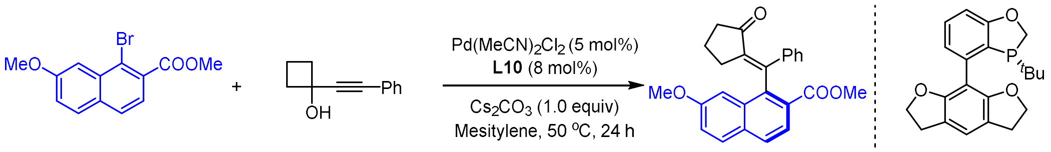

To a screw-cap vial (8 mL) were charged $Pd(MeCN)_2Cl_2$ (5.0 mol%), chiral ligand **L10** (8 mol%, 20% ee, 40% ee, 60% ee, 80% ee, 99% ee), aryl bromide (0.15 mmol), 1-alkynylcyclobutanol (0.10 mmol) and Cs_2CO_3 (0.10 mmol) in mesitylene (2.0 mL). The reaction mixtures were stirred at 50 °C for 24 h. The residue was purified by preparative TLC to afford the product's ee. The ee was determined by HPLC using a chiral stationary phase.

**L10** of ee (%)	**44** of ee (%).
0	0
20	15.3
40	35.1
60	47.8
80	73.7
99	92.0

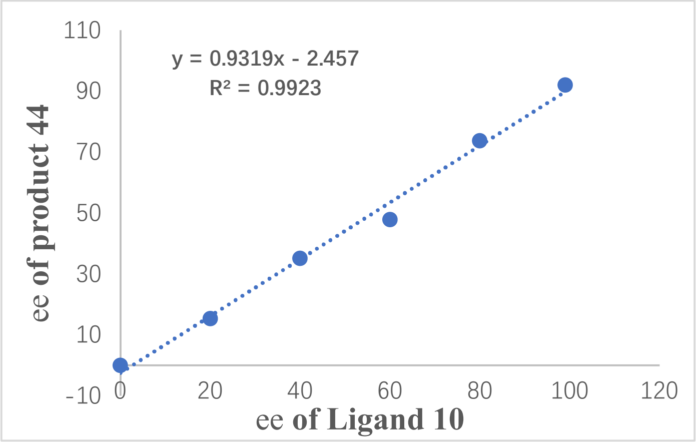

5.4.1 Effect of concentration on the enantioselectivity of product 3

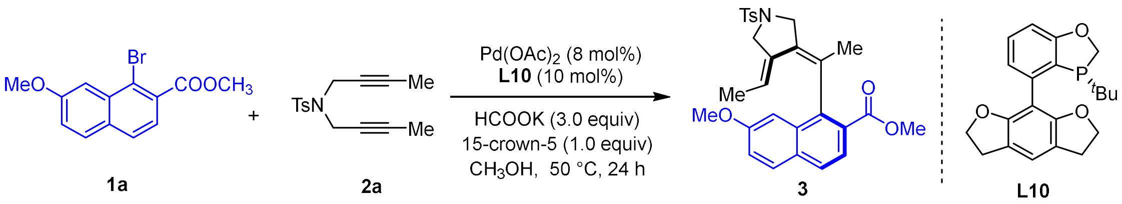

A screw-cap vial (8 mL) was charged with $Pd(OAc)_2$ (8.0 mol%), chiral ligand **L10** (10 mol%) and aryl bromide (0.12 mmol), 1,6-diyne (0.10 mmol), 15-crown-5 (0.10 mmol) and HCOOK (0.30 mmol) in MeOH (0.050M, 0.033M, 0.025M, 0.020M, 0.017M). The reaction mixtures were stirred at 50 °C for 24 h. The ee was determined by HPLC using a chiral stationary phase.

concentration (M)	**3** of ee (%).
0.05	95.1
0.033	95.2
0.025	95.2
0.02	94.9
0.017	94.8

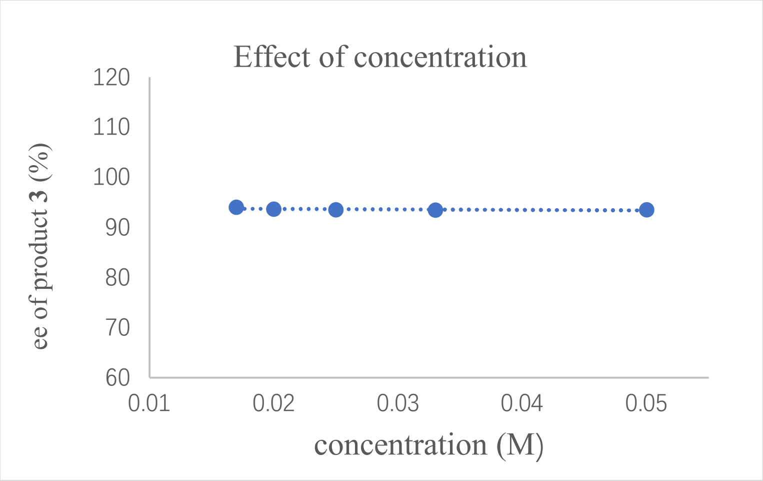

5.4.2 Effect of concentration for the enantioselectivity of product 44

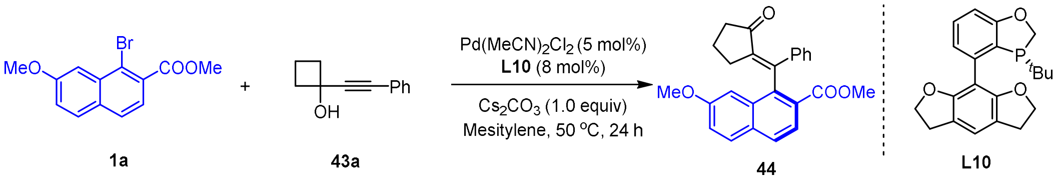

A screw-cap vial (8 mL) was charged with $Pd(MeCN)_2Cl_2$ (5.0 mol%), chiral ligand **L10** (8.0 mol%), aryl bromide (0.15 mmol), 1-alkynylcyclobutanol (0.10 mmol) and Cs_2CO_3 (0.10 mmol) in mesitylene (0.050M, 0.033M, 0.025M, 0.020M, 0.017M). The reaction mixtures were stirred at 50 °C for 24 h. The ee was determined by HPLC using a chiral

stationary phase.

concentration (M)	**44** of ee (%).
0.05	93.5
0.033	93.4
0.025	93.5
0.02	93.6
0.017	94.0

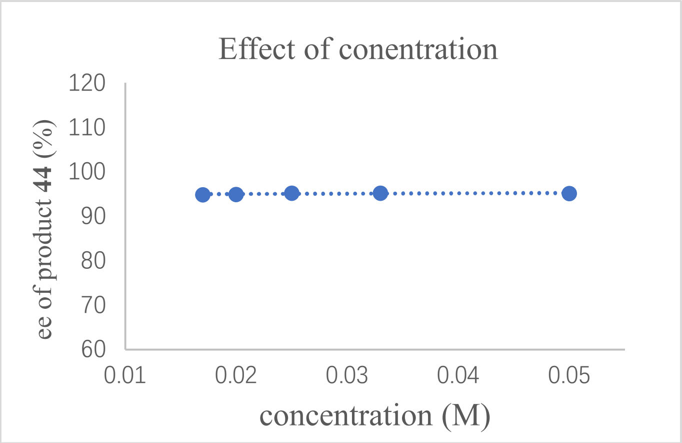

5.5 The Hammett Plot of alkynylcyclobutanols

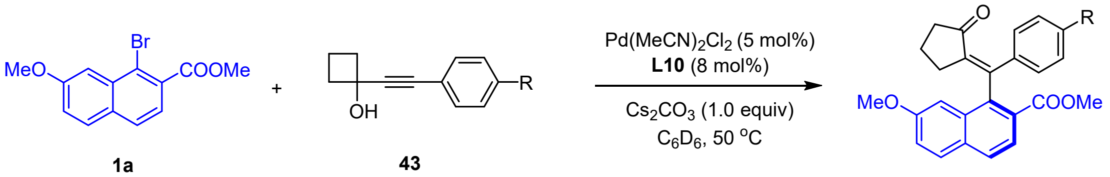

1a **43**

R = H, F, Me, CF_3

To a sealable tube (8 mL) were charged with aryl bromides (0.15 mmol), 1-alkynylcyclobutanols (0.10 mmol), $Pd(MeCN)_2Cl_2$ (5 mol%), **L10** (8 mol%), Cs_2CO_3 (1.0 mmol), and C_6D_6 (2.0 mL) under N_2 at 50 °C, Aliquots of the reaction mixture (200 µL) were taken out via syringe at a specific time. The mixture was transferred into an NMR tube and analyzed by quantitative ^1H NMR using the signal of trimethoxybenzene (δ 6.11 ppm) as an internal standard (C_6D_6 as a deuterated solvent).

Table S12. Hammett plots of 1-alkynylcyclobutanols

R	σ_p	Initial Reaction Rate (M/min)
Me	-0.17	9.69E-02
H	0	0
F	0.06	-3.78E-02
CF_3	0.43	-4.77E-01

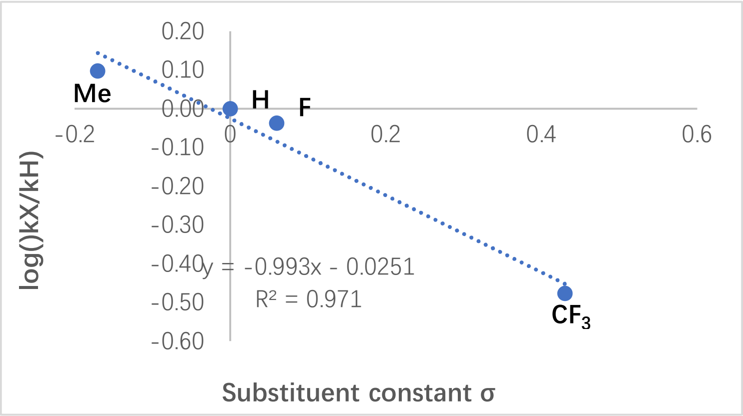

5.6 Deuterium-labeling experiment of 44

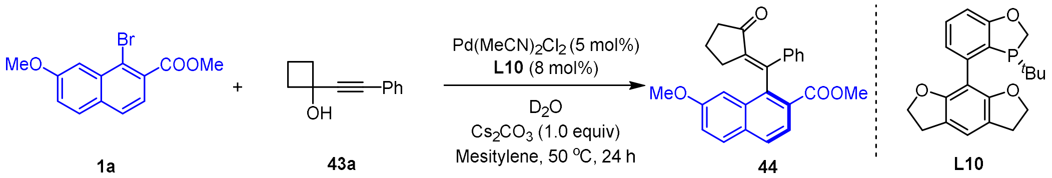

A screw-cap vial (8 mL) was charged with $Pd(MeCN)_2Cl_2$ (5.0 mol%), chiral ligand **L10** (8.0 mol%) and aryl bromides (0.15 mmol), 1-alkynylcyclobutanols (0.10 mmol) and Cs_2CO_3 (0.10 mmol) in mesitylene (2.0 mL), separately add varying variable amount of D_2O (1.0 eq, 3.0 eq, 5.0 eq, 7.0 eq), The reaction mixtures were stirred at 50 °C for 12 h. After that, the reaction mixture was concentrated under vacuum. The residue was purified by flash chromatography on silica gel (petroleum ether/EtOAc = 10/1) to afford the desired product. The deuteration level was determined by ^1H NMR analysis (600 MHz, $CDCl_3$), no deuteration was observed.

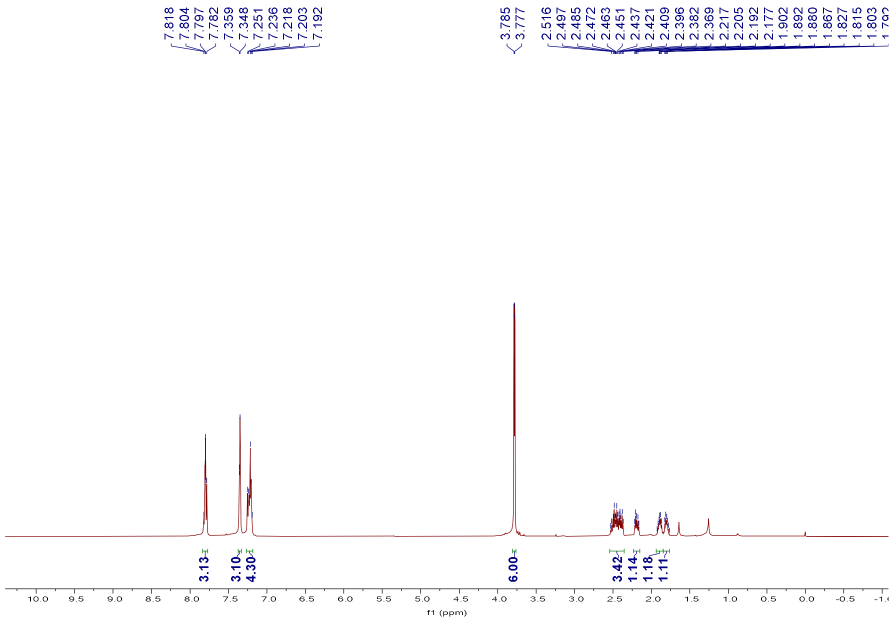

5.7 The inhibition of TBAB for 3

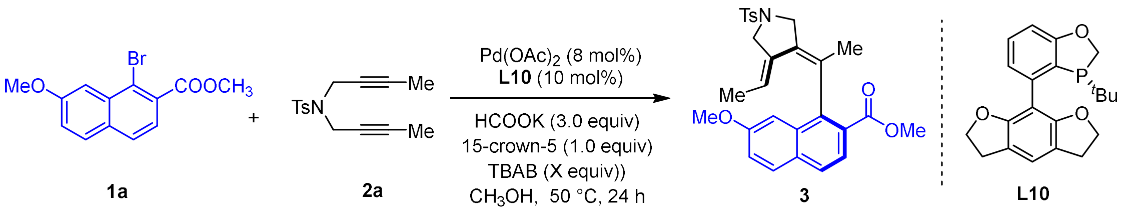

A screw-cap vial (8 mL) was charged with Pd(OAc)$_2$ (8.0 mol%), chiral ligand **L10** (10 mol%) and aryl bromides (0.12 mmol), 1,6-diyne (0.10 mmol), 15-crown-5 (0.10 mmol) and HCOOK (0.30 mmol) in MeOH (2.0 mL), separately add varying variable amount of TBAB (0.5 equiv, 1.0 equiv, 2.0 equiv), The reaction mixtures were stirred at 50 °C for 24 h. After that, the reaction mixture was concentrated under vacuum. The residue was purified by flash chromatography on silica gel (petroleum ether/EtOAc = 8/1) to afford the desired product.

Equivalent of TBAB	**3** of Yield (%).
0	72
0.5	55
1.0	41
2.0	33

6. X-Ray Crystallographic Data

X-ray crystal structure of (*R*)-5 (CCDC 2416759)

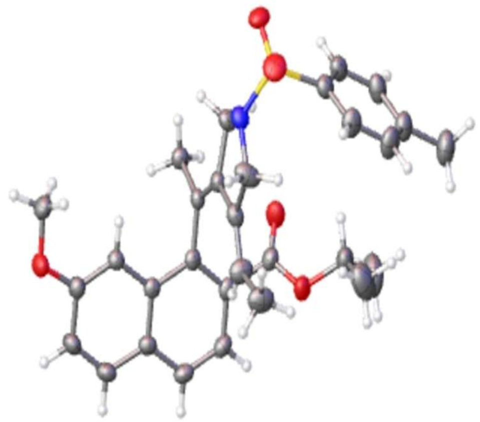

Table S13. Crystal data and structure refinement for 5.

Bond precision:	C-C = 0.0026 A		Wavelength = 1.54178 A
Cell:	a = 10.0206(9)	b = 15.6245(14)	c = 17.2804(16)
	Alpha = 90	Beta = 90	Gamma = 90
Temperature: 183 K			

	Calculated	Reported
Volume	2705.5(4))	2705.5(4)
Space group	P 21 21 21	P 21 21 21
Hall group	P 2ac 2ab	P 2ac 2ab
Moiety formula	C30 H33 N O5 S	C30 H33 N O5 S
Sum formula	C30 H33 N O5 S	C30 H33 N O5 S
Mr	519.63	519.67
Dx,g cm-3	1.460	1.460
Z	4	4
Mu (mm-1)	1.387	1.387
F000	1104.0	1480.0
F000'	1485.68	1108.9
h,k,lmax	12,18,20	12,18,20
Nref	4954[2805]	4848
Tmin,Tmax	0.847,0.870	0.678,0.753
Tmin'	0.847	

Correction method= # Reported T Limits: Tmin= 0.678 Tmax= 0.753 AbsCorr = MULTI-SCAN

Data completeness = 1.73/0.98 Theta(max)= 68.260

R(reflections) = 0.0318(4733) wR2(reflections)= 0.0874(4848)

S = 1.048 Npar = 340

Flack parameter: 0.038(6)

X-ray crystal structure of (*R*)-26 (CCDC 2416760)

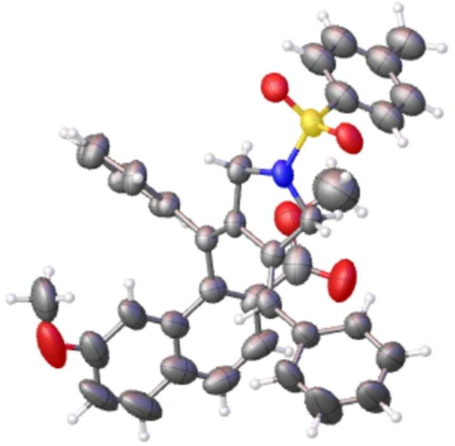

Table S14. Crystal data and structure refinement for 26.

Bond precision:	C-C = 0.0098A		Wavelength = 1.54178 A
Cell:	a = 9.8872(12)	b = 31.082(4)	c = 11.0103(14)
	Alpha = 90	Beta = 108.905(4)	Gamma = 90
Temperature: 243 K			

	Calculated	Reported
Volume	3201.1(7)	3201.1(7)
Space group	P 21	P 1 21 1
Hall group	P 2yb	P 2yb
Moiety formula	C38 H33 N O5 S [+ solvent]	4(C38 H33 N O5 S)
Sum formula	C38 H33 N O5 S [+ solvent]	C152 H132 N4 O20 S4
Mr	615.71	2462.85
Dx,g cm-3	1.278	1.278
Z	4	1
Mu (mm-1)	1.262	1.262
F000	1296.0	1296.0
F000'	1301.04	
h,k,lmax	11,37,13	12,18,20
Nref	11801[6018]	11558
Tmin,Tmax	0.886,0.904	0.644,0.753
Tmin'	0.859	

Correction method= # Reported T Limits: Tmin= 0.644 Tmax= 0.753 AbsCorr = MULTI-SCAN

Data completeness = 1.92/0.98 Theta(max)= 68.511

R(reflections) = 0.0895(11261) wR2(reflections)= 0.2551(11558)

S = 0.995 Npar =744

Flack parameter: 0.064(4)

X-ray crystal structure of (*R*)-59 (CCDC 2416758)

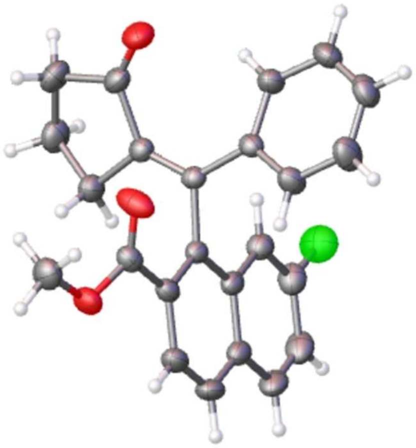

Table S15. Crystal data and structure refinement for 59.

Bond precision:	C-C = 0.0034 A		Wavelength = 1.54178 A
Cell:	a = 9.2269(2)	b = 13.0774(3)	c = 15.7550(4)
	Alpha = 90	Beta = 100.223(1)	Gamma = 90
Temperature:	200 K		

	Calculated	Reported
Volume	1870.88(8)	1870.88(8)
Space group	P 21	P 1 21 1
Hall group	P 2yb	P 2yb
Moiety formula	C24 H19 F O3 [+ solvent]	C24 H19 F O3
Sum formula	C24 H19 F O3	C24 H19 F O3
Mr	374.39	374.39
Dx,g cm-3	1.329	1.329
Z	4	4
Mu (mm-1)	0.765	0.765
F000	784.0	784.0
F000'	786.53	
h,k,lmax	11,15,18	11,15,18
Nref	6871[3599]	6719
Tmin,Tmax	0.848,0.871	0.658,0.753
Tmin'	0.826	

Correction method= # Reported T Limits: Tmin= 0.658 Tmax= 0.753 AbsCorr = MULTI-SCAN

Data completeness = 1.87/0.98 Theta(max)= 68.357

R(reflections) = 0.0336(6627) wR2(reflections)= 0.0957(6719)

S = 1.034 Npar =507

Flack parameter: -0.09(5)

7. Rotational Barriers

7.1 Rotational Barrier of (*R*)-3

The enantiomerisation barrier, corresponding to the barrier to rotation for the following atropisomers, was obtained by kinetic of racemization of an enantiomer. The slope of the first order kinetic line gives the racemization constant ($k_{racemisation}$ = 2 × $k_{enantiomerisation}$). Eyring equation gives the enantiomerisation barrier ($\Delta G^{\ddagger}$ enaniomerization) from enantiomerisation constant ($k_{enantiomerisation}$), R = 8.31451 $J{\cdot}K^{-1}mol^{-1}$, h = 6.62608 × 10^{-34} Js and k_B = 1.38066 × 10^{-23} J/K. Reactions were conducted at 1 mg/mL concentration in a pressure tube. Enantiomeric excess data were determined by HPLC.

Estimated Measurement of Racemization of **3** in Toluene at 135 °C.

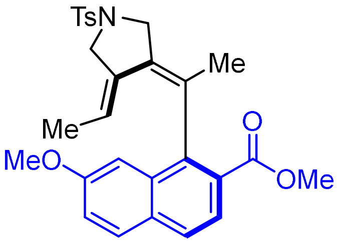

$\Delta G^{\ddagger}$ = 34.76 kcal/mol (135 °C, Toluene)

T/s	ee	ln (ee_0/ee_t)
0	95.0	0.000000
3600	94.1	0.009519
7200	93.1	0.020203
10800	92.0	0.032088
14400	90.2	0.051847
21600	87.6	0.081096
25200	86.4	0.094889

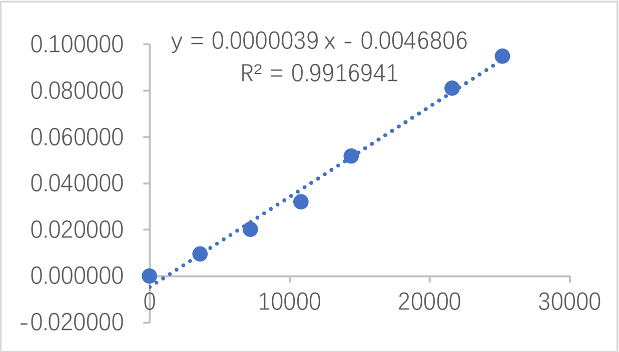

$k_{racemization}$ (135 °C) = 3.9×10^{-6} s^{-1}

$k_{enantiomerization}$ (135 °C) = 1.95×10^{-6} s^{-1}

Employing the Eyring equation: $\Delta G^{\ddagger}_{enantiomerization} = RT\times \ln\frac{k_B\times T}{hk_{enantiomerisation}}$

$$\Delta G^{\neq} = 8.314\ J \bullet K^{-1} \bullet mol^{-1} \times 408.15\ K \times \ln\frac{1.381\times10^{-23} J\bullet K^{-1} \times 408.15\ K}{1.95\times10^{-5}\ S^{-1}\times 6.626\times10^{-34}\ J\bullet s}$$

$$\Delta G^{\neq} = 145.5\ kJ \bullet mol^{-1} = 34.8\ kcal \bullet mol^{-1}$$

7.2 Rotational Barriers of (*R*)-44

The enantiomerisation barrier, corresponding to the barrier to rotation for the

following atropisomers, was obtained by kinetic of racemisation of an enantiomer. The slope of the first order kinetic line gives the racemisation constant ($k_{racemisation}$ =2 × $k_{enantiomerisation}$). Eyring equation gives the enantiomerisation barrier ($\Delta G^{\ddagger}$ enaniomerization) from enantiomerisation constant ($k_{enantiomerisation}$), R = 8.31451 $J \cdot K^{-1} mol^{-1}$, h = 6.62608 × 10^{-34} Js and k_B = 1.38066 × 10^{-23} J/K. Reactions were conducted at 1 mg/mL concentration in a pressure tube. Enantiomeric excess data were determined by HPLC.

Measurement of Racemization of **44** in Toluene at 115 °C.

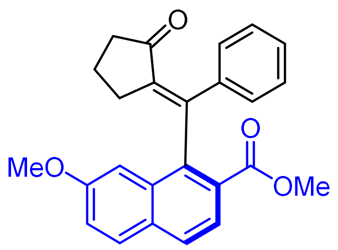

$\Delta G^{\ddagger}$ = 31.8 kcal/mol (115 °C, toluene)

t/s	ee	ln(ee_0/ee_t)
0	92.1	0.000000
3600	86.9	0.058117
7200	80.6	0.133376
10800	76.0	0.192142
14400	71.3	0.255979
18000	68.1	0.301898
21600	63.3	0.374990

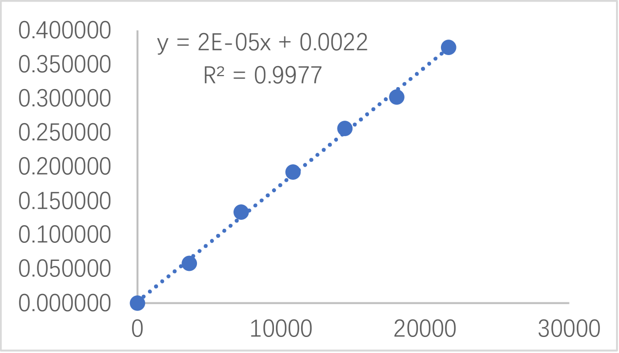

$k_{racemization}$ (115 °C) = 1.7 ×10^{-5} s^{-1}

$k_{enantiomerization}$ (115 °C) = 8.5 ×10^{-6} s^{-1}

Employing the Eyring equation: $\Delta G^{\ddagger}_{enantiomerization}$ = RT× ln$\frac{k_B \times T}{h k_{enantiomerisation}}$

$$\Delta G^{\neq} = 8.314\ J \bullet K^{-1} \bullet mol^{-1} \times 388.15\ K \times \ln\frac{1.381\times10^{-23} J \bullet K^{-1} \times 388.15\ K}{8.5\times10^{-6}\ S^{-1} \times 6.626\times10^{-34}\ J \bullet s}$$

$$\Delta G^{\neq} = 133.4\ kJ \bullet mol^{-1} = 31.9\ kcal \bullet mol^{-1}$$

8. DFT Calculation

8.1 Computational details

All the calculations were performed using Gaussian 09 package.[11] The geometry optimizations were carried out using B3LYP-D3(BJ)[12] functional with a mixed basis set of SDD for Pd and 6-31G(d) for all other atoms. Frequencies were computed analytically at the same level of theory to confirm whether the structures are minima (no imaginary frequencies) or transition states (only one imaginary frequency). Selected transition-state structures were confirmed to connect the correct reactants and products by intrinsic reaction coordinate (IRC) calculations.[13] To obtain better accuracy, solution-phase single-point energies for the optimized geometries were recalculated using B3LYP-D3(BJ) functional with a larger mix basis set of SDD for Pd and 6-311+G(d,p) for all other atoms. Solvation effects (solvent = mesitylene) were considered by performing single-point calculations with the SMD model.[14] The final free energies reported in the article are the large basis set single-point energies corrected by gas-phase Gibbs free energy correction (at 298.15 K). All 3D structures of the optimized geometries were generated using CYLview.[15]

8.2 Migration insertion into Pd-C(alkyl) bond

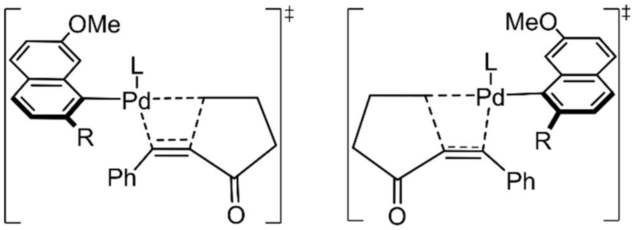

TS2 $\Delta\Delta G^{\ddagger} = 3.0$ **TS2'** $\Delta\Delta G^{\ddagger} = 4.7$

Figure S1. Migration insertion into Pd-C(alkyl) bond. Energies are given in kcal/mol.

Table S16. Calculated energies and energy corrections

Stationary point	Single-point energy- B3LYP-D3(BJ)-SMD/SDD&6-311+G(d,p) (a.u.)	Thermal correction to Gibbs free energy at 298.15 K (a.u.)
**TS1 (R)**	-944.819901	0.362408
**TS1' (S)**	-311.861902	0.149973
**TS2**	-944.855006	0.362512
**TS2'**	-944.855006	0.362512

8.3. Cartesian coordinates

TS1

C	-0.77459000	-4.54943600	-0.84858100
C	-1.64199100	-4.07268000	-1.79417500
C	-2.05457200	-2.71350000	-1.78658400
C	-1.50695500	-1.82614700	-0.80288600
C	-0.59046400	-2.33785200	0.15198400
C	-0.25526900	-3.67920400	0.14605200
H	-3.43741300	-2.92398800	-3.43309200
H	-0.46696300	-5.58981100	-0.82580800
H	-2.04645200	-4.73813800	-2.55268700
C	-3.02523900	-2.23601600	-2.69921200
C	-1.92972800	-0.45486400	-0.78082800
H	-0.16302200	-1.65098200	0.87002300
C	-2.91210000	-0.04561200	-1.68786000
C	-3.46650800	-0.93459300	-2.64152200
H	-4.23528800	-0.57550300	-3.31788600
O	0.56379500	-4.29049800	1.04245700
C	-3.39627500	1.36647500	-1.68259400
O	-2.67345800	2.34803600	-1.70354300
O	-4.73750700	1.43039200	-1.66240200
C	-5.29387600	2.75647200	-1.55632000
H	-6.37197100	2.62153800	-1.64497800
H	-5.03553700	3.17799000	-0.58263000
H	-4.91487700	3.39395900	-2.35902100
C	1.00232000	-3.52959900	2.15927600
H	1.65681200	-2.71133200	1.85052800

H	0.14587100	-3.11522100	2.70739200
H	1.54790800	-4.22695300	2.79829400
Pd	-0.69848100	1.19254900	0.07829000
C	0.16258000	2.84238500	1.07037800
H	1.19305400	2.56316800	1.30630300
H	0.18955600	3.67522300	0.35529900
C	-0.55876000	3.29565800	2.33758100
H	0.01507300	4.09365300	2.83694100
H	-0.61917800	2.47385200	3.06615700
C	-1.97231800	3.81655500	2.08011200
H	-2.39320700	4.31698100	2.96052600
H	-1.95073500	4.56790300	1.27743200
C	-2.96036800	2.74697900	1.65660600
O	-4.16997400	2.96899100	1.59690900
C	-2.41867500	1.47490800	1.23984900
C	-2.53630500	0.20869000	1.07153700
C	-2.98328400	-1.00013900	1.75467300
C	-3.86326000	-1.91127300	1.15283600
C	-2.51380000	-1.25308300	3.05264000
C	-4.25671900	-3.05709800	1.83706100
H	-4.22616500	-1.71709700	0.14993700
C	-2.90516000	-2.40570900	3.73141100
H	-1.83674300	-0.54183200	3.51323700
C	-3.77316600	-3.31361400	3.12266000
H	-4.93782700	-3.75718300	1.36224800
H	-2.53073500	-2.59495600	4.73363300
H	-4.07317300	-4.21609000	3.64741700
C	3.25756300	0.92424400	2.63066100
C	4.54789200	1.42340300	2.46757000
C	5.26274000	1.03304200	1.33652800
C	4.67969300	0.17218600	0.40152000
C	3.38180900	-0.34050800	0.51352300
C	2.71344000	0.05889700	1.67602500
H	4.98192200	2.10065000	3.19835700
O	5.48946100	-0.08753500	-0.66918300
C	6.60611600	0.83750700	-0.59480600
C	6.66264300	1.35230200	0.86198600
H	7.49909800	0.29790000	-0.91494800
H	6.40614100	1.65397600	-1.29877300

H	7.41940700	0.81601900	1.45013400
H	6.90765800	2.41845900	0.90366500
O	1.45385100	-0.35720200	1.98759500
C	1.13138300	0.12574100	3.31734500
C	2.21481700	1.16229500	3.69956700
H	1.13208400	-0.74030000	3.98898600
H	0.12702600	0.54402800	3.26586100
H	2.59089900	0.99706000	4.71479300
H	1.81808700	2.18331900	3.65437400
C	2.79208600	-1.21294200	-0.53454800
C	1.85437900	-0.71810800	-1.45466700
C	3.21360600	-2.54246200	-0.64728000
C	1.36564400	-1.56309600	-2.46369600
C	2.69907800	-3.37148400	-1.64754900
H	3.94789800	-2.92348300	0.05477000
C	1.77632600	-2.89021300	-2.57163100
H	3.02251000	-4.40641000	-1.70498400
H	1.35989300	-3.51954200	-3.34967000
C	0.20217500	0.34225400	-3.14268900
O	0.44708100	-1.06106800	-3.33124300
H	0.63898800	0.88033500	-3.98825100
H	-0.87439300	0.49558700	-3.13466000
P	0.98048200	0.88274500	-1.50837200
C	2.18614600	2.22471800	-2.10101800
C	1.32654900	3.29476700	-2.80252600
H	0.89386000	2.94061200	-3.74316300
H	0.50580300	3.63114700	-2.15915700
H	1.95340200	4.16399500	-3.03741900
C	2.91077700	2.88937000	-0.91879200
H	2.22819200	3.47566100	-0.30504700
H	3.41206100	2.16687600	-0.27611200
H	3.67399400	3.57144500	-1.31673500
C	3.23312300	1.62758100	-3.05438500
H	3.88916500	0.92638600	-2.52975000
H	2.78636400	1.09732900	-3.90177200
H	3.85090100	2.43826500	-3.46220000

TS1'

C	0.80818000	-4.24839300	-1.78420400

C	1.55134900	-3.53123100	-2.69536100
C	1.94382500	-2.19543800	-2.44039200
C	1.50289800	-1.56615700	-1.22992600
C	0.72685000	-2.31409900	-0.31982500
C	0.41134600	-3.63742500	-0.56807300
H	3.13684800	-1.97217200	-4.23373200
H	0.54350700	-5.27651100	-1.99977700
H	1.86594300	-4.00186300	-3.62347400
C	2.79649500	-1.48282400	-3.32473000
C	1.88988600	-0.21329400	-0.94781400
H	0.38526800	-1.84634700	0.59368500
C	2.74962000	0.42578200	-1.83822300
C	3.21289100	-0.20916100	-3.02110400
H	3.89120300	0.32570300	-3.67803400
O	-0.27772400	-4.29468500	0.41073200
C	3.20555600	1.82090100	-1.55545200
O	2.46522300	2.75930200	-1.32025300
O	4.54346600	1.91541000	-1.59244800
C	5.08736300	3.19402100	-1.20146100
H	6.15881000	3.11663600	-1.38639500
H	4.64591500	3.99427800	-1.80025400
H	4.88760500	3.35734000	-0.14011000
C	-0.66316900	-5.63781800	0.18266500
H	0.20712800	-6.29008500	0.03450000
H	-1.19589700	-5.95241500	1.08185400
H	-1.33269800	-5.72481900	-0.68319500
Pd	0.69503200	1.13467700	0.36323700
C	-0.09840200	2.51985800	1.75374200
H	-0.03585100	3.51881000	1.30096000
H	-1.14678700	2.25963200	1.89364700
C	0.62751600	2.51948000	3.09563100
H	0.69924200	1.49698200	3.49260200
H	0.04455700	3.08575900	3.83912600
C	2.02639400	3.13007700	3.01956200
H	1.96833500	4.14778000	2.60758600
H	2.48293400	3.22989900	4.01284800
C	3.00837900	2.36503500	2.15187900
O	4.17777500	2.72837300	2.02103800
C	2.50090200	1.22967700	1.41974700

C	2.65600100	0.05297900	0.93333800
C	3.22942800	-1.24469800	1.27961100
C	4.06991000	-1.94710800	0.40373400
C	2.93010600	-1.79631800	2.53502600
C	4.59233600	-3.18145900	0.77734400
H	4.30465100	-1.52106900	-0.56515000
C	3.44868000	-3.03672100	2.90129400
H	2.28588200	-1.24352300	3.21110700
C	4.27677800	-3.73491500	2.02112700
H	5.24480200	-3.71648200	0.09331700
H	3.20428200	-3.45731800	3.87266500
H	4.67846700	-4.70387800	2.30376700
C	-4.04753800	-2.54334300	0.19473500
C	-3.10672200	-3.04427200	1.09217500
C	-2.36733000	-2.13397600	1.84044000
C	-2.57206100	-0.75755500	1.68092000
C	-3.47962200	-0.20849400	0.76798200
C	-4.21852600	-1.16354000	0.06084100
H	-2.95278900	-4.11281200	1.20354600
O	-1.81909500	-0.00694500	2.53093600
C	-0.81659200	-0.88438600	3.10751800
C	-1.33569500	-2.32902400	2.92785200
H	0.11099100	-0.71436500	2.55109800
H	-0.67688000	-0.57991900	4.14595100
H	-0.53655100	-3.01571100	2.63410500
H	-1.79000100	-2.71295000	3.85136100
O	-5.12653400	-0.82524800	-0.90725400
C	-5.80084500	-2.04422600	-1.30988300
C	-4.91576900	-3.22235300	-0.83988200
H	-5.94302100	-1.99619900	-2.39107600
H	-6.78160000	-2.06094000	-0.81960900
H	-4.31074600	-3.62403800	-1.66441800
H	-5.51907700	-4.04596600	-0.44326000
C	-3.68247900	1.24733300	0.54943200
C	-2.76375200	2.02936500	-0.17061900
C	-4.85510200	1.84902700	1.02305600
C	-3.03907200	3.38939400	-0.39158500
C	-5.10688300	3.20359400	0.79566100
H	-5.56978500	1.24362700	1.57052100

C	-4.20349700	3.99007000	0.08548800
H	-6.01987300	3.65182100	1.17721400
H	-4.38195600	5.04286300	-0.10473500
C	-0.95425600	3.38690700	-1.44543100
O	-2.14628000	4.12112300	-1.11021100
H	-0.78033000	3.49724200	-2.51728700
H	-0.10928900	3.81910000	-0.90841400
P	-1.16983800	1.58408800	-0.94270200
C	-1.59428500	0.73906300	-2.59107000
C	-1.86477500	-0.75491000	-2.35469600
H	-1.90519700	-1.26811700	-3.32454200
H	-1.08241400	-1.22683300	-1.75771800
H	-2.82095600	-0.90745600	-1.85754800
C	-2.83358000	1.39004100	-3.22567600
H	-3.72115200	1.24250200	-2.60418900
H	-2.70446700	2.46514300	-3.39063500
H	-3.02206500	0.92489600	-4.20201800
C	-0.37621700	0.88301800	-3.51927600
H	-0.61091300	0.41529100	-4.48363300
H	-0.11174100	1.92667500	-3.71625300
H	0.50151800	0.38572800	-3.10829100

TS2

C	-0.91176100	4.94334500	0.37047400
C	-1.88507200	4.50506800	1.22963700
C	-2.18671400	3.12266000	1.35733700
C	-1.41518300	2.17199900	0.60899100
C	-0.42322300	2.65602600	-0.29376700
C	-0.18819200	4.01148100	-0.41835200
H	-3.83809900	3.40879600	2.72562000
H	-0.68400500	5.99817700	0.25389100
H	-2.45797900	5.22010300	1.81526200
C	-3.25064700	2.67535900	2.17897300
C	-1.66974000	0.77367500	0.76374000
H	0.14308500	1.92930800	-0.86115100
C	-2.73869900	0.39294700	1.57322100
C	-3.54828900	1.33919600	2.25773800
H	-4.38491800	0.98906400	2.85412100
O	0.71451400	4.58538000	-1.26752100

C	-3.06599800	-1.05107900	1.73810300
O	-2.26486500	-1.94294200	1.97909700
O	-4.38560000	-1.28551400	1.58756400
C	-4.78372100	-2.66566200	1.63408000
H	-5.87326000	-2.65289000	1.59545200
H	-4.37965500	-3.19733100	0.76899800
H	-4.43426900	-3.13860200	2.55527500
C	1.34525200	3.74677800	-2.21936500
H	1.99184400	3.00981600	-1.73781500
H	0.60188300	3.21772200	-2.83109900
H	1.94056700	4.40742600	-2.85363900
Pd	-0.66095500	-0.76650400	-0.19312400
C	0.22473700	-2.53581200	-1.35106000
H	1.18450600	-2.09046100	-1.08709400
C	0.07691900	-3.96153400	-0.84217700
H	0.01045500	-3.96139100	0.25070300
H	0.95056800	-4.57371400	-1.10994800
C	-1.19575700	-4.57772000	-1.42607300
H	-1.02078400	-4.88766300	-2.46751200
H	-1.54794700	-5.46269800	-0.88804800
C	-2.29806900	-3.54373700	-1.46526400
O	-3.48879100	-3.83352700	-1.56268700
C	-1.88412300	-2.14878300	-1.38872300
C	-2.30559400	-0.92976100	-1.44433900
C	3.35444600	-0.73537600	-2.61861000
C	4.63105700	-1.25949500	-2.42636000
C	5.32656300	-0.88389200	-1.27868300
C	4.74420000	-0.00203300	-0.36249200
C	3.45948200	0.53667100	-0.50224500
C	2.80407300	0.13725600	-1.67406000
H	5.07038700	-1.94164300	-3.14943300
O	5.54280500	0.25657100	0.71627300
C	6.64331500	-0.68943500	0.67125700
C	6.71237000	-1.22374500	-0.77793200
H	7.54087700	-0.16199100	0.99843900
H	6.41750900	-1.49275100	1.38256400
H	7.48788800	-0.70936900	-1.36101300
H	6.93795100	-2.29473600	-0.80261500
O	1.55007900	0.55699400	-2.00379500

C	1.24771200	0.09067500	-3.34669500
C	2.34169700	-0.93969300	-3.72328400
H	1.26419200	0.96527900	-4.00562800
H	0.23734000	-0.31983400	-3.31879000
H	2.75087800	-0.74306500	-4.72001300
H	1.94715700	-1.96251700	-3.72554500
C	2.87803700	1.46065700	0.50742100
C	1.89304600	1.04153000	1.41381500
C	3.34290000	2.77953700	0.57811900
C	1.38873500	1.95158400	2.35714100
C	2.80715000	3.67655900	1.50600200
H	4.12004700	3.10132500	-0.10735900
C	1.82580500	3.27358300	2.40706800
H	3.15949600	4.70336000	1.52369000
H	1.39017800	3.95678300	3.12733100
C	0.21170700	0.09808800	3.13147500
O	0.42413100	1.51618300	3.20963000
H	0.69538400	-0.36759200	3.99479100
H	-0.85709500	-0.08740700	3.16497900
P	0.96824500	-0.52689300	1.52364400
C	2.14836900	-1.87356800	2.17326200
C	2.82499100	-2.62196800	1.01338400
H	3.58243600	-3.30147800	1.42604000
H	2.11375100	-3.23028200	0.45638600
H	3.32628900	-1.95011700	0.31682900
C	3.23527000	-1.26154700	3.07048800
H	3.83104700	-2.07121000	3.51255600
H	3.90661100	-0.61357900	2.49988900
H	2.82162600	-0.67198700	3.89475800
C	1.27773500	-2.87441200	2.95914800
H	0.90026700	-2.45524300	3.89644900
H	0.41367300	-3.20678700	2.37425200
H	1.88010700	-3.75576700	3.21299100
H	0.14079400	-2.48175900	-2.43838900
C	-3.46734200	-0.14956600	-1.79909500
C	-3.39370700	1.24808700	-1.92932700
C	-4.69939900	-0.80127700	-2.01141700
C	-4.52990000	1.98186000	-2.25476200
H	-2.44680200	1.74482500	-1.75941800

C	-5.83149700	-0.05873200	-2.33259100
H	-4.74194300	-1.88058600	-1.90698800
C	-5.75091700	1.33174800	-2.45215900
H	-4.46480200	3.06236300	-2.34477900
H	-6.78119800	-0.56381900	-2.48591100
H	-6.63902800	1.90782700	-2.69780200

TS2'

C	-1.98460000	-4.15361000	1.48904000
C	-2.54578400	-3.36583800	2.47068800
C	-2.39905400	-1.95916900	2.46020500
C	-1.60588100	-1.34962200	1.43309100
C	-0.97654200	-2.19116800	0.48454600
C	-1.20213400	-3.55317900	0.47399200
H	-3.65008300	-1.59853800	4.19445800
H	-2.14904700	-5.22524800	1.49787800
H	-3.14851800	-3.82386800	3.25123000
C	-3.07085200	-1.13206200	3.40142200
C	-1.55914700	0.08134200	1.29778900
H	-0.36841200	-1.74971900	-0.29466600
C	-2.28960600	0.84100300	2.21514400
C	-3.01949500	0.22871100	3.28447300
H	-3.54899600	0.85978600	3.98760300
O	-0.64793700	-4.26829100	-0.55949500
C	-2.33340500	2.31726600	2.09280700
O	-1.82341300	2.99586500	1.21056800
O	-3.00876100	2.90562100	3.11414300
C	-3.07310600	4.33391400	3.06554500
H	-2.06992000	4.76693900	3.11915100
H	-3.55336500	4.66985000	2.14273600
H	-3.66181500	4.62933300	3.93473800
C	-1.25477100	-5.50374600	-0.89872300
H	-2.33946700	-5.38963300	-1.01951300
H	-0.81169600	-5.80439200	-1.85054400
H	-1.05184300	-6.28320900	-0.15185400
Pd	-0.64317200	0.96025700	-0.31980800
C	0.13658600	2.33765500	-1.97997800
H	1.19415800	2.07946800	-1.95773400
C	-0.26495600	2.76456300	-3.38486400

H	0.31772200	3.65462300	-3.66191500
H	-1.32359200	3.05280000	-3.41779400
C	-0.00265200	1.62154400	-4.35718300
H	-0.30639000	1.82029900	-5.38947700
H	1.06720900	1.37028400	-4.35937000
C	-0.76044200	0.43361800	-3.83127600
O	-1.09646900	-0.53077000	-4.51634300
C	-1.12186400	0.49096400	-2.41994800
C	-1.98510200	0.02885000	-1.58484000
C	1.99498800	-2.51972000	-1.60700400
C	2.27352700	-3.29895600	-0.48922000
C	2.98073700	-2.70888200	0.55492700
C	3.35798600	-1.36741900	0.47236800
C	3.03132400	-0.52266600	-0.59560600
C	2.38045300	-1.17369900	-1.65103900
H	1.93901900	-4.32867100	-0.43423400
O	4.09247200	-0.94779600	1.54806300
C	4.36120300	-2.10479400	2.38195900
C	3.42806900	-3.24027000	1.89720900
H	5.41836000	-2.36387100	2.25454400
H	4.18918400	-1.80496600	3.41755700
H	3.95608000	-4.19770500	1.83652400
H	2.57437300	-3.37578700	2.57399900
O	2.07604700	-0.57826400	-2.84738500
C	1.77677300	-1.66726100	-3.77417300
C	1.27357700	-2.82527900	-2.89838600
H	2.71527600	-1.93202200	-4.27823700
H	1.05240000	-1.30136200	-4.49714500
H	1.52203400	-3.80167500	-3.32557200
H	0.18587700	-2.78107300	-2.76028200
C	3.44032000	0.90584100	-0.62309900
C	2.73356300	1.89623700	0.08213600
C	4.57493500	1.27819300	-1.35474300
C	3.17636000	3.22862600	0.02270200
C	5.00821900	2.60560100	-1.38113000
H	5.11938800	0.51349600	-1.89867000
C	4.31494200	3.59757500	-0.69111200
H	5.89204100	2.87159000	-1.95393200
H	4.62511800	4.63677200	-0.71066600

C	1.15669500	3.68165800	1.07817400
O	2.45242200	4.17119800	0.68888700
H	0.93859300	4.06425800	2.07358300
H	0.40640400	4.07626100	0.38971400
P	1.15210500	1.80199500	1.00515200
C	1.57332500	1.29938700	2.80220800
C	1.33928300	-0.21246100	2.97411100
H	1.72262100	-0.79795400	2.14076800
H	1.83961100	-0.54956200	3.89113700
H	0.27564800	-0.43378800	3.06587200
C	3.03248500	1.68119000	3.10280600
H	3.73302700	1.11753000	2.48665600
H	3.20660000	2.75128800	2.94590700
H	3.24545000	1.45993300	4.15704300
C	0.65846600	2.03113900	3.80272400
H	0.82512300	3.11200500	3.82094400
H	-0.39949700	1.83673800	3.62451200
H	0.89107400	1.65582700	4.80735700
H	-0.09921900	3.14766300	-1.28872400
C	-3.07136100	-0.92229800	-1.49910200
C	-4.03455800	-0.85912900	-0.47836200
C	-3.11410600	-1.98440300	-2.42425400
C	-5.00497600	-1.84976600	-0.36876400
H	-3.99403800	-0.04628900	0.23645300
C	-4.08055700	-2.97867000	-2.29943400
H	-2.38120900	-2.00637700	-3.22353400
C	-5.02219000	-2.91898700	-1.26832300
H	-5.73675400	-1.79835100	0.43209300
H	-4.10201400	-3.80026800	-3.01058900
H	-5.77188900	-3.69955600	-1.16989400

9. References

(1) Q. Li, Y. Wang, B. Li and B. Wang, *Org. Lett.,* 2018, **20**, 7884–7887.

(2) P. Hu, L. Hu, X. Li, M. Pan, G. Lu and X. Li, *Angew. Chem. Int. Ed.,* 2024, **63**, e202312923.

(3) K. R. Strom, A. C. Impastato, K. J. Moy, A. J. Landreth and J. K. Snyder, *Org. Lett.,* 2015, **17**, 2126–2129.

(4) T. Xu, Q. Yang, W. Ye, Q. Jiang, Z. Xu, J. Chen and Z. Yu, Substituent-Dependent, *Chem. Eur. J.,* 2011, **17**, 10547–10551.

(5) W. Li, S. Chen, J. Xie, Z. Fan, K. Yang and Q. Song, *Nat. Synth.,* 2023, **2**, 140–151.

(6) S. Xu, H. Qiu, P. Xie, Z. Wang, X. Wang, C. Zheng, S. You and T, Mei, *CCS Chem.,* 2025, **7**, 245–255.

(7) D. Guo, J. Zhang, B. Zhang and J. Wang, *Org. Lett.,* 2018, **20**, 6284–6288.

(8) S. Gupta, V. R. Sabbasani, S. Su, D. J. Wink and D. Lee, *ACS Catal.,* 2021, **11**, 1977–1987.

(9) Y. Gang and L. Dong, *J. Org. Chem.,* 2024, **89**, 12912–12923.

(10) H. Chen, Y. Chen, J. Gao, L. Ye and B. Zhou, *Angew. Chem. Int. Ed.,* 2024, **63**, e202411709.

(11) M. J. Frisch, et al. *Gaussian 09, Revision E.01;* Gaussian, inc.: Wallingford, ct. **2013**.

(12) (a) C. Lee, W. Yang and R. G. Parr, *Phys. Rev. B,* 1988, **37**, 785–789; (b) A. D. Becke, *J. Chem. Phys.,* 1993, **98**, 5648–5652; c) S. Grimme, J. Antony, S. Ehrlich and H. Krieg, *J. Chem. Phys.,* 2010, **132**, 154104.

(13) (a) K. Fukui, *J. Phys. Chem.,* 1970, **74**, 4161–4163; (b) K. Fukui, *Acc. Chem. Res.*, 1981, **14**, 363–368.

(14) A. V. Marenich, C. J. Cramer and D. J. Truhlar, *J. Phys. Chem. B,* 2009, **113**, 6378–6396.

(15) C. Y. Legault, CYLview 1.0b, Université de Sherbrooke: http://www.cylview.org **2009.**

10. NMR Spectra

^{1}H NMR (600 MHz, Chloroform-d) spectrum of 3

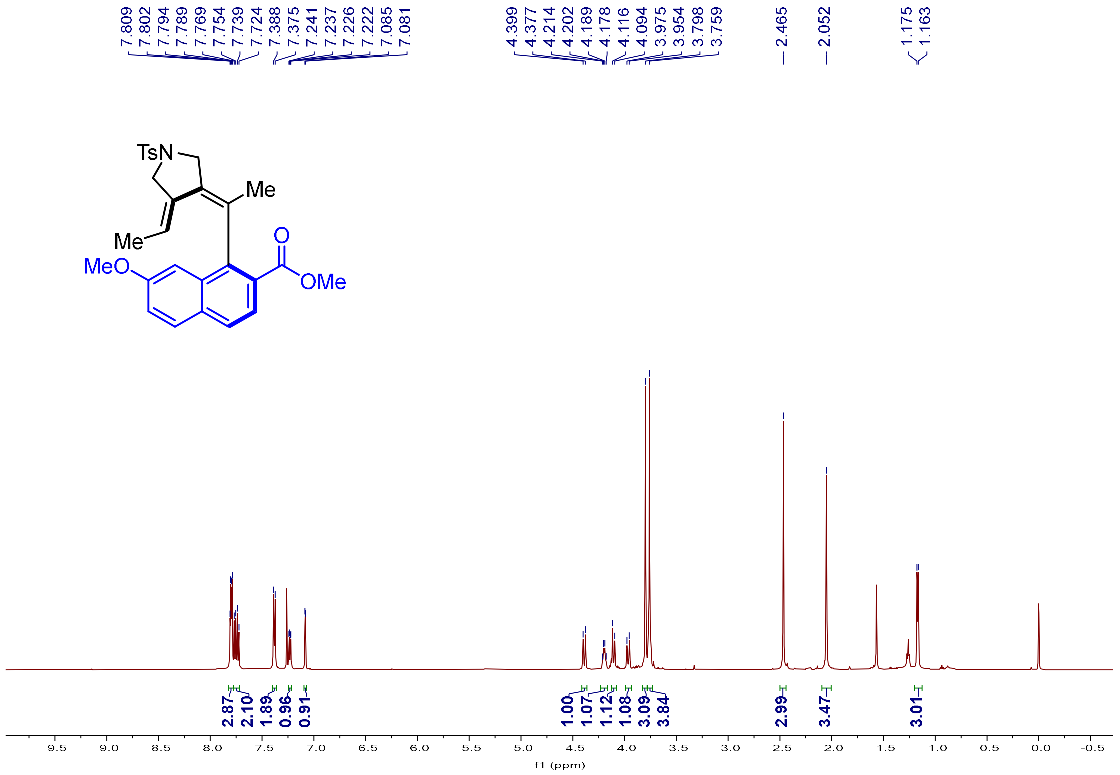

^{13}C NMR (150 MHz, Chloroform-d) spectrum of 3

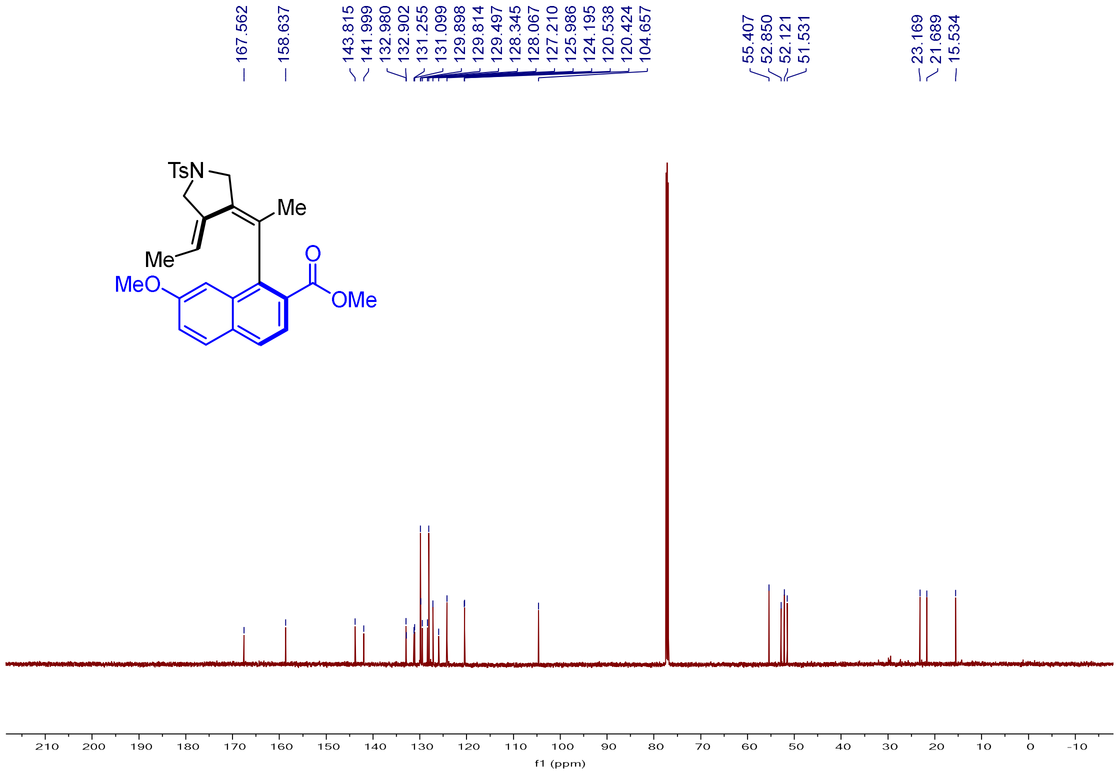

^{1}H NMR (600 MHz, Chloroform-d) spectrum of 4

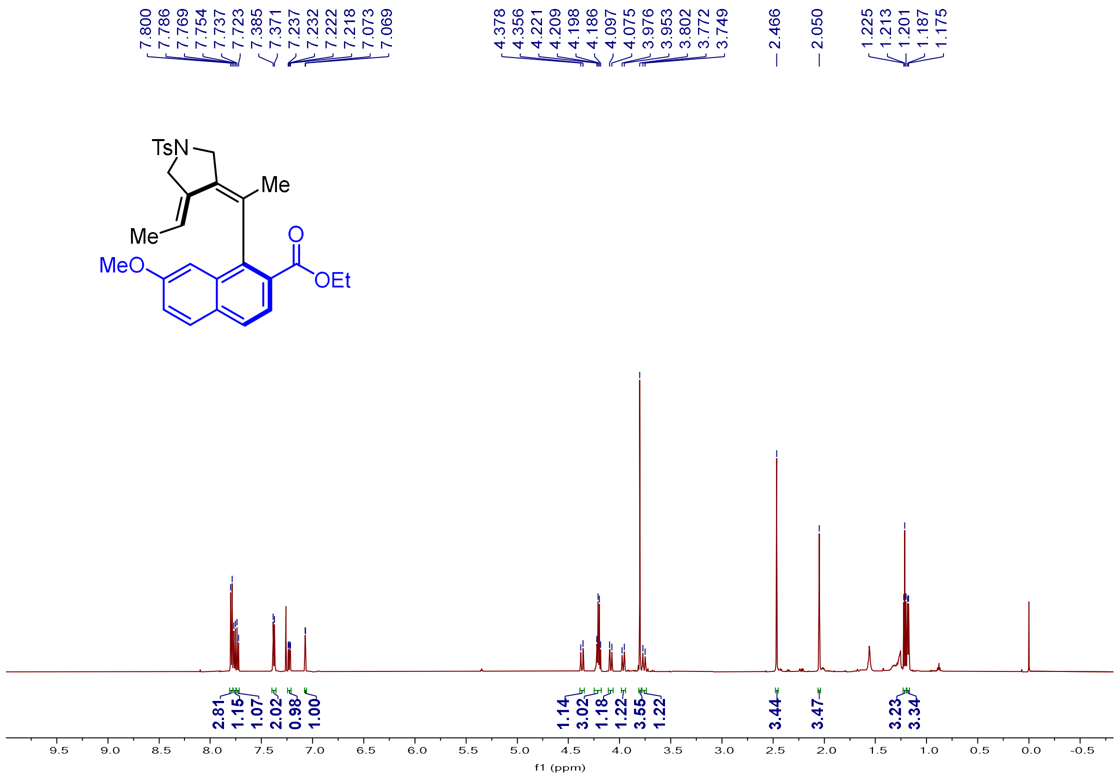

^{13}C NMR (150 MHz, Chloroform-d) spectrum of 4

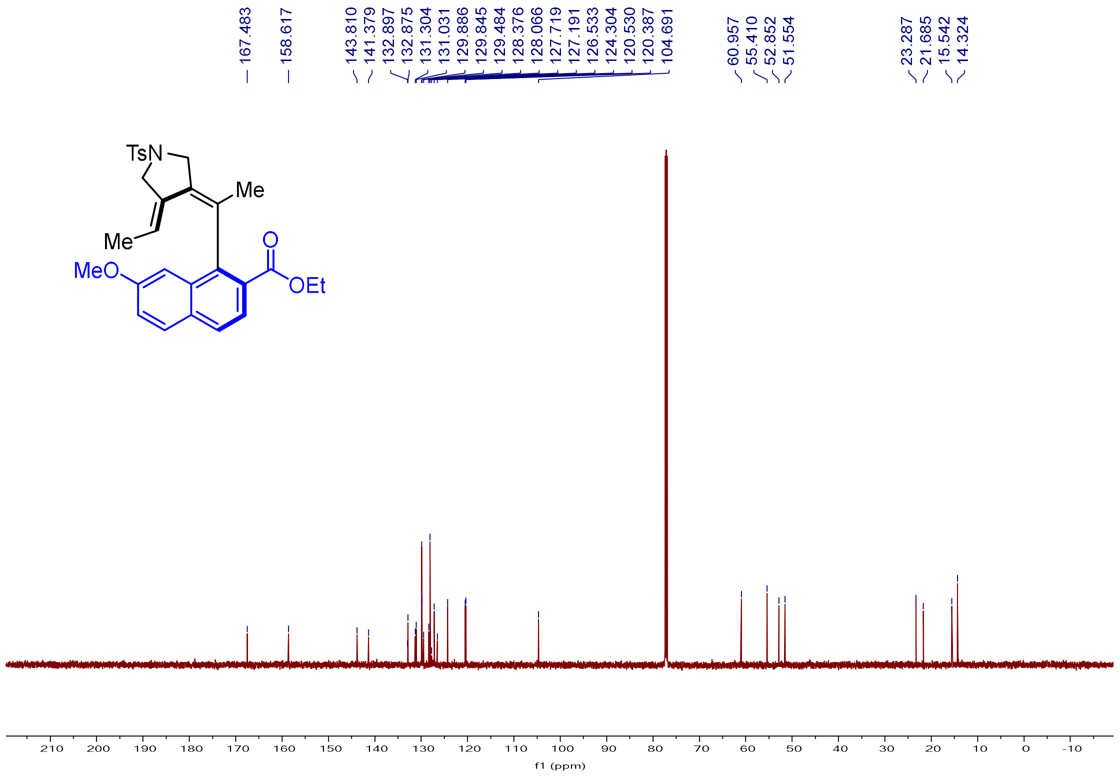

^{1}H NMR (600 MHz, Chloroform-d) spectrum of 5

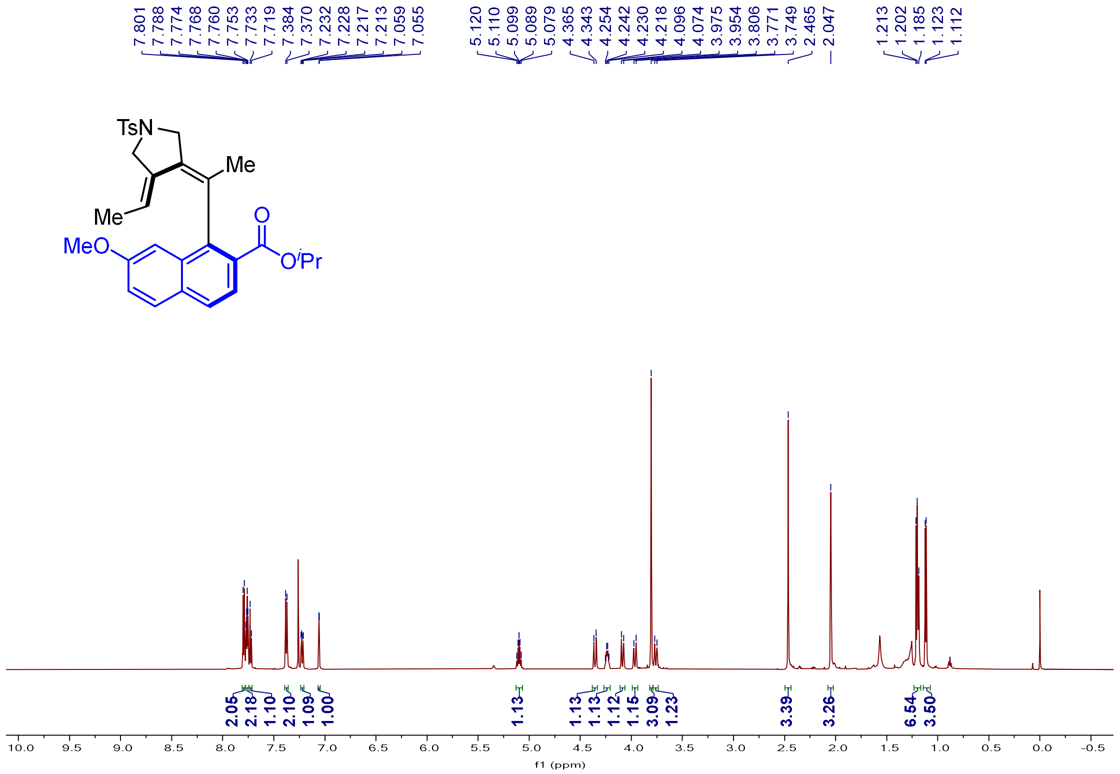

^{13}C NMR (150 MHz, Chloroform-d) spectrum of 5

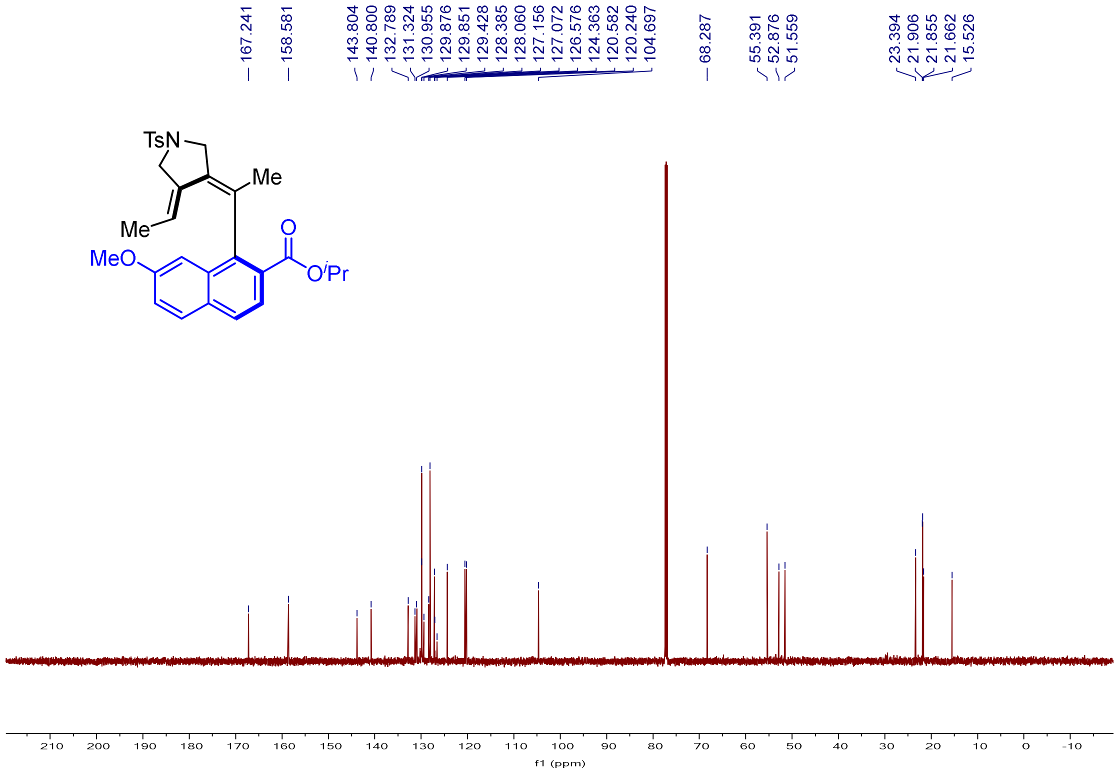

^{1}H NMR (600 MHz, Chloroform-d) spectrum of 6

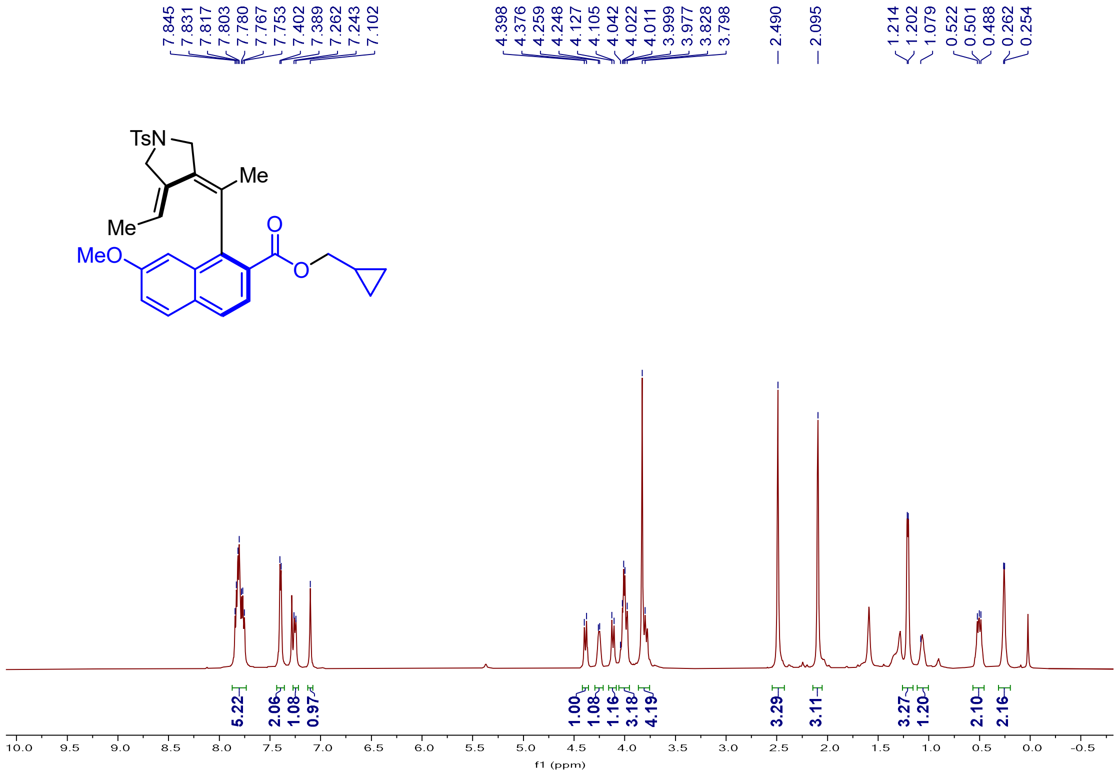

^{13}C NMR (150 MHz, Chloroform-d) spectrum of 6

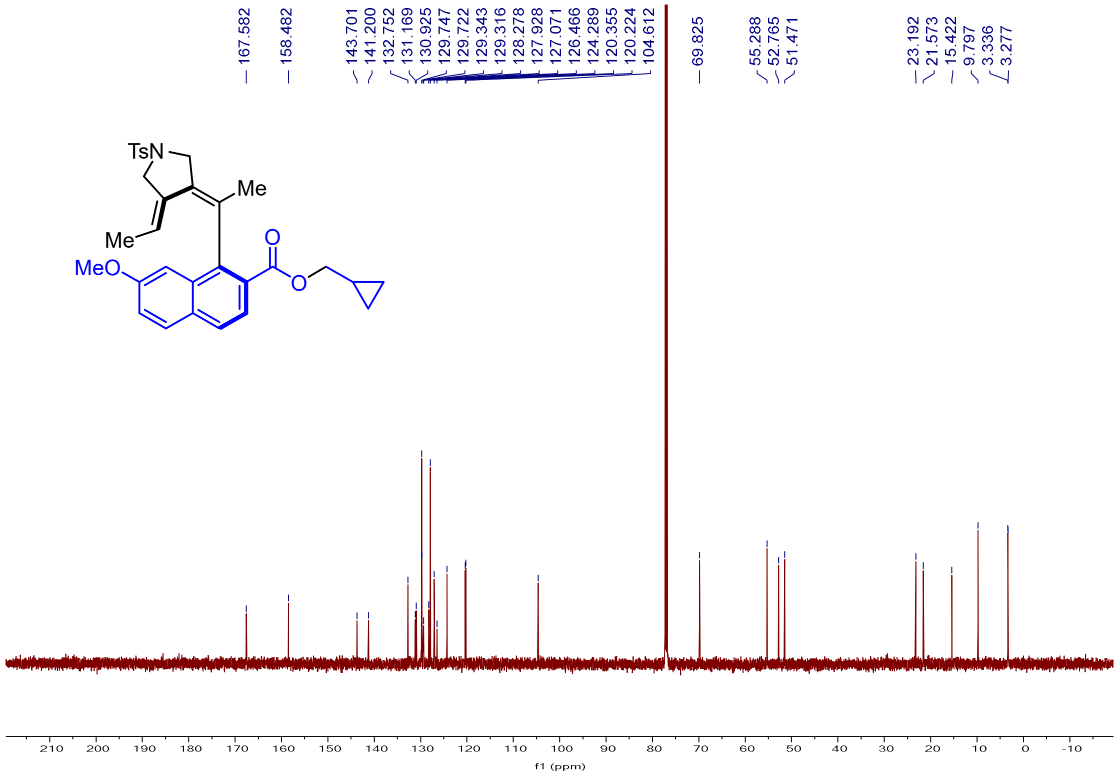

^{1}H NMR (600 MHz, Chloroform-d) spectrum of 7

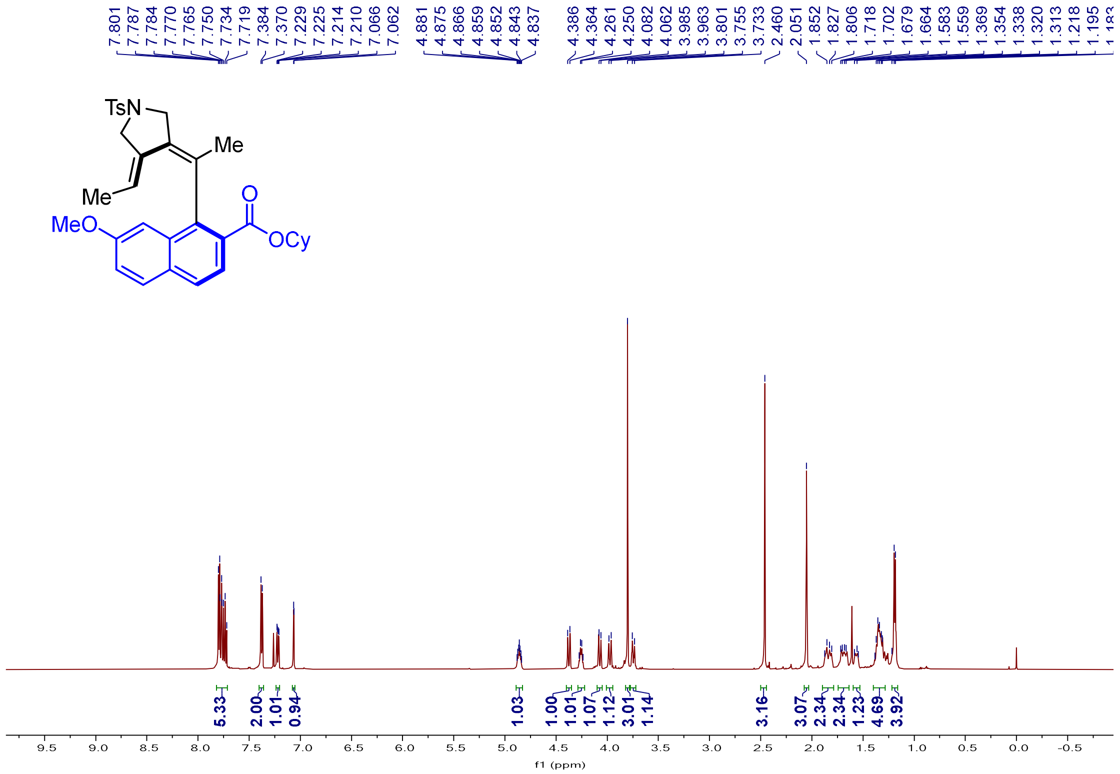

^{13}C NMR (150 MHz, Chloroform-d) spectrum of 7

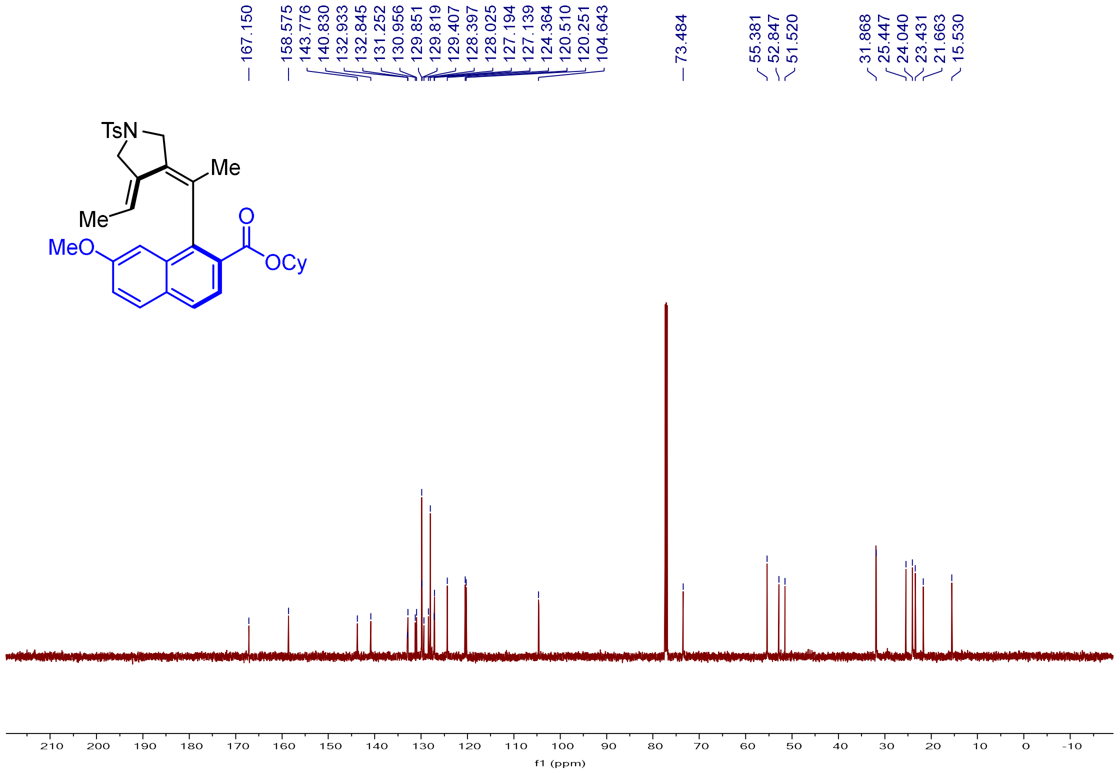

^{1}H NMR (600 MHz, Chloroform-d) spectrum of 8

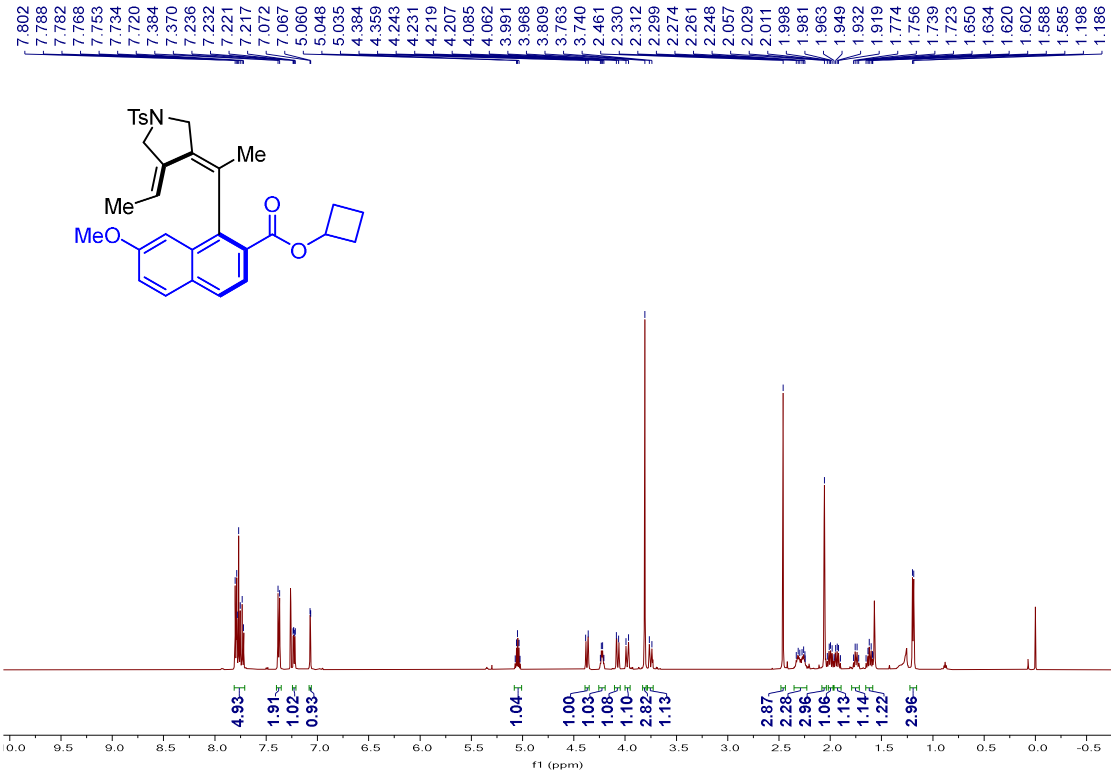

^{13}C NMR (150 MHz, Chloroform-d) spectrum of 8

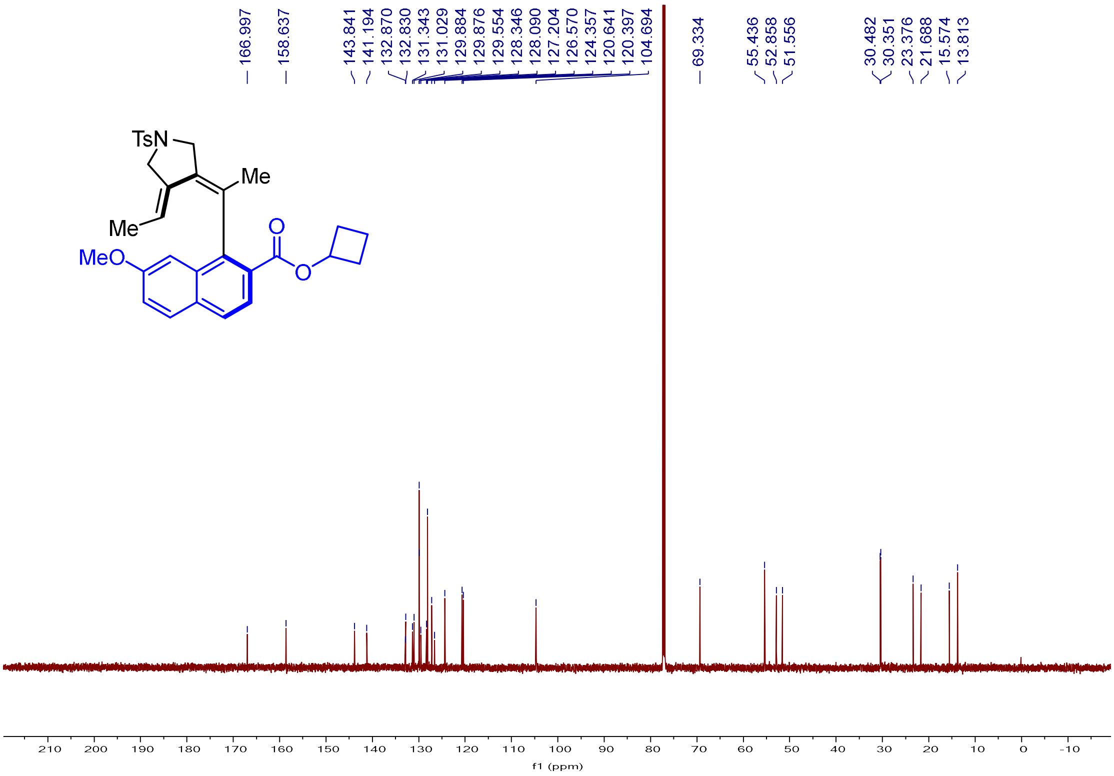

^{1}H NMR (600 MHz, Chloroform-d) spectrum of 9

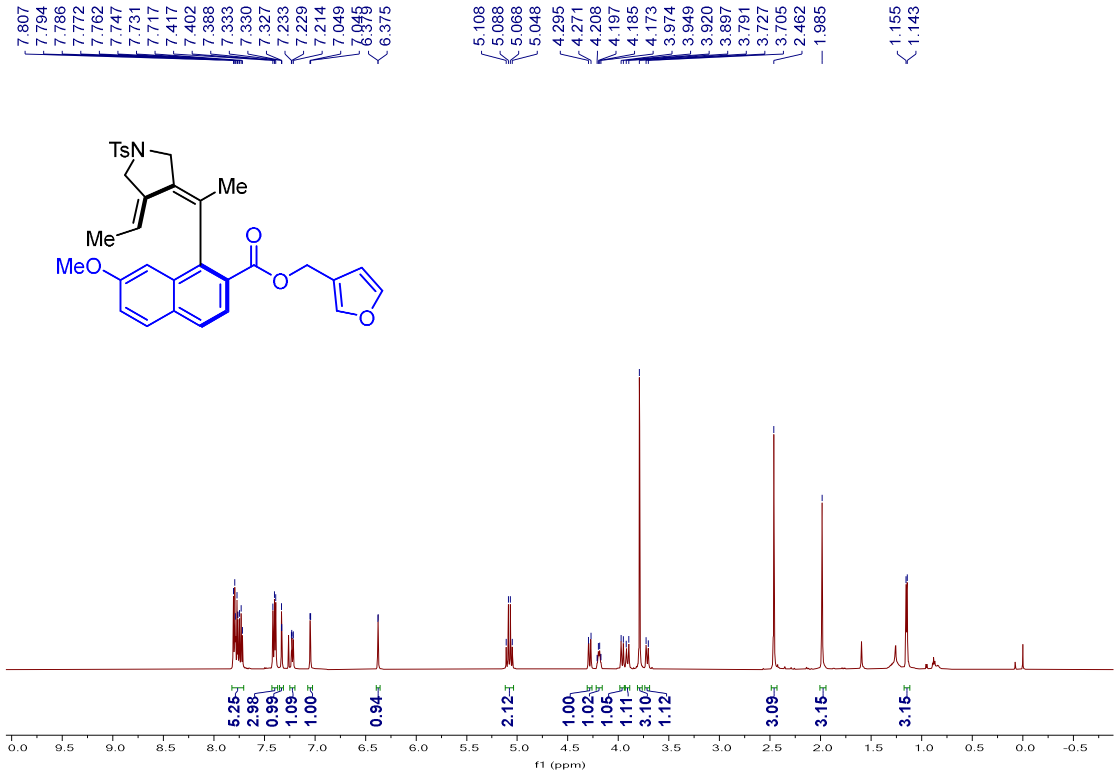

^{13}C NMR (150 MHz, Chloroform-d) spectrum of 9

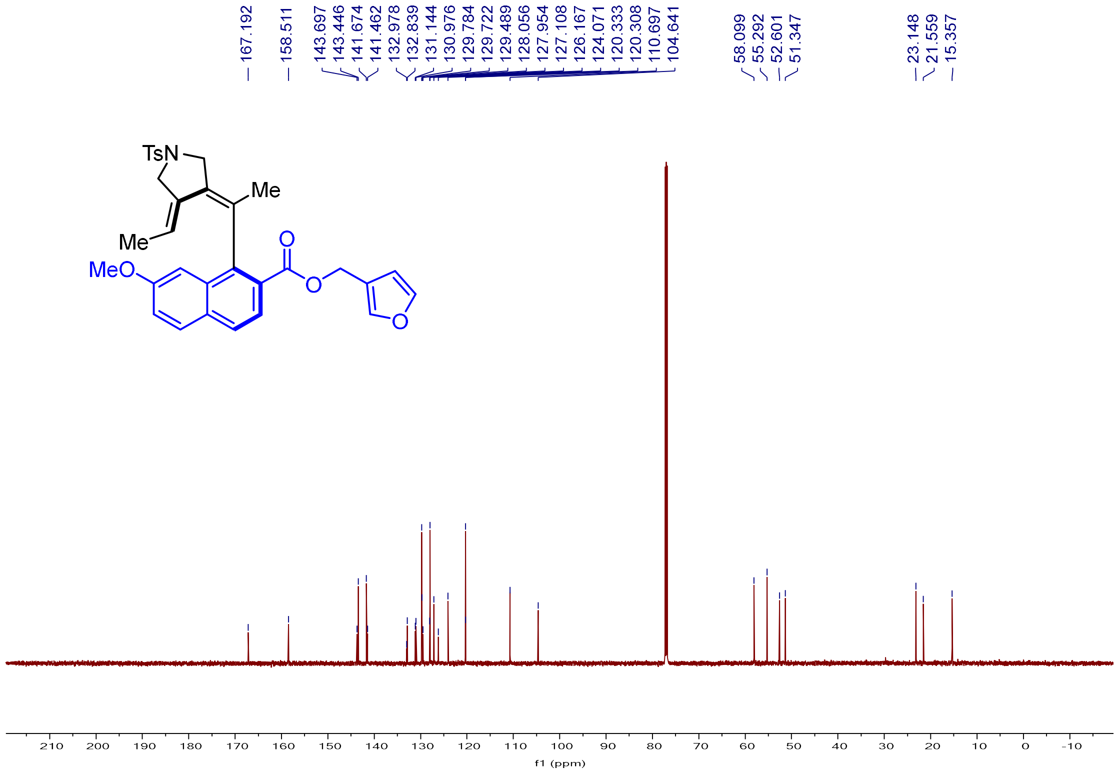

^{1}H NMR (600 MHz, Chloroform-d) spectrum of 10

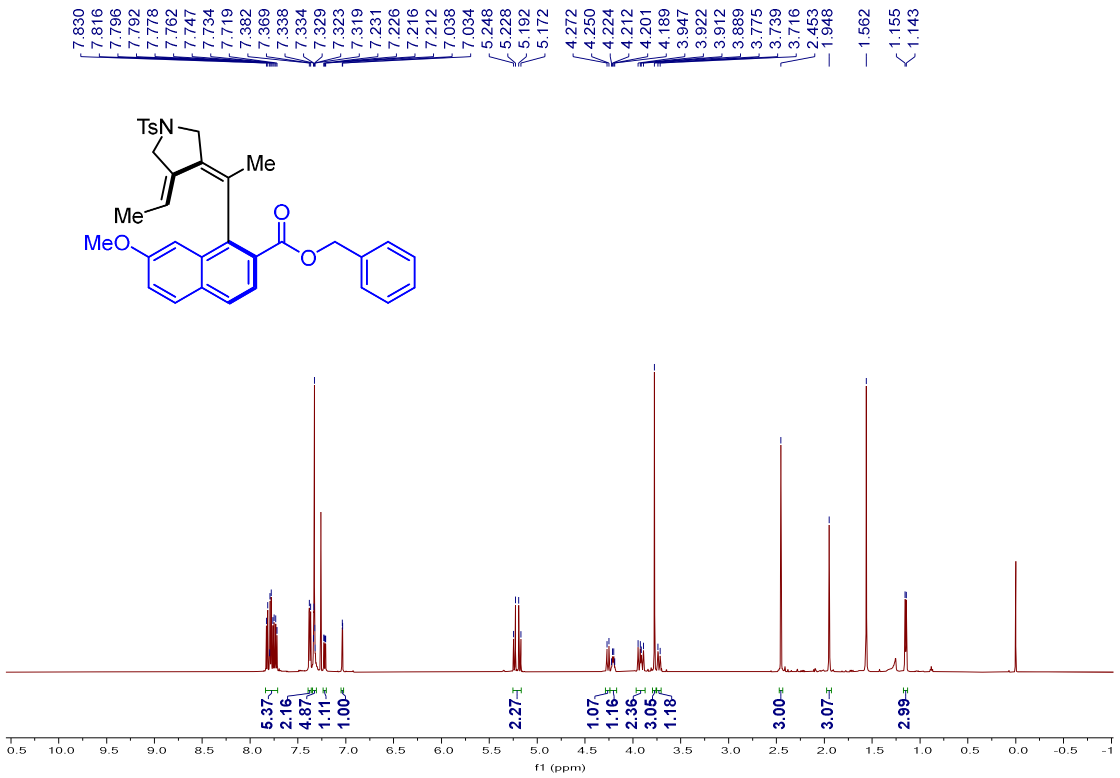

^{13}C NMR (150 MHz, Chloroform-d) spectrum of 10

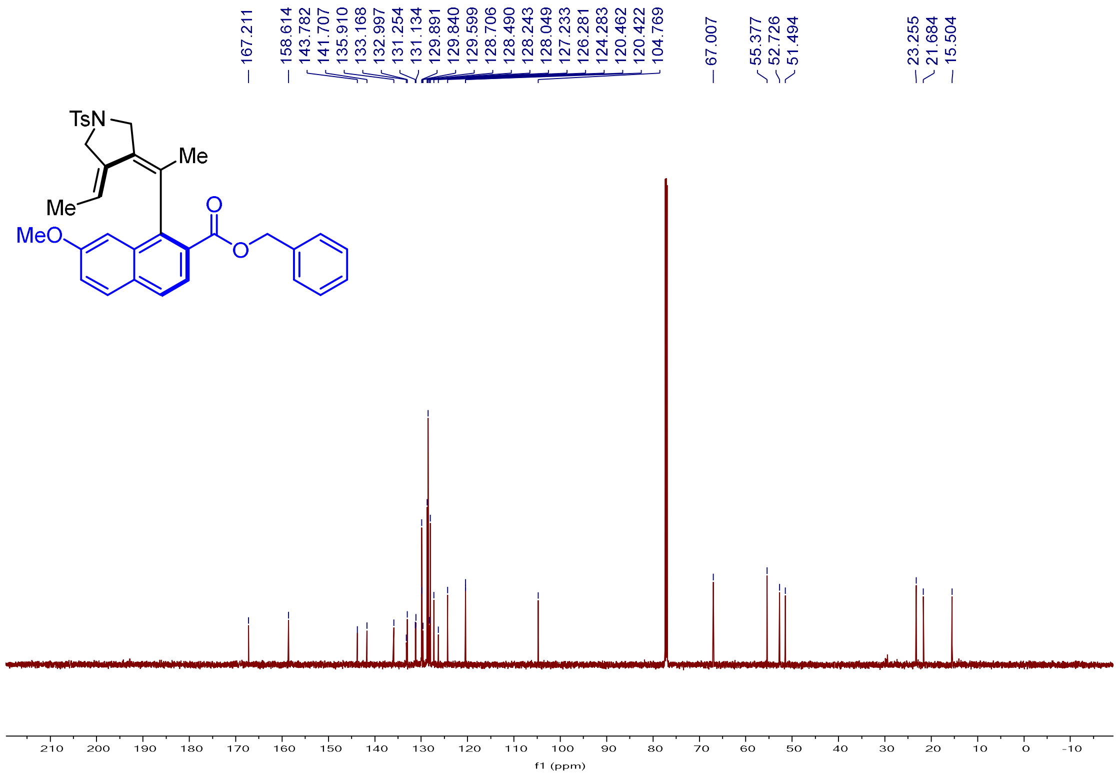

^{1}H NMR (600 MHz, Chloroform-d) spectrum of 11

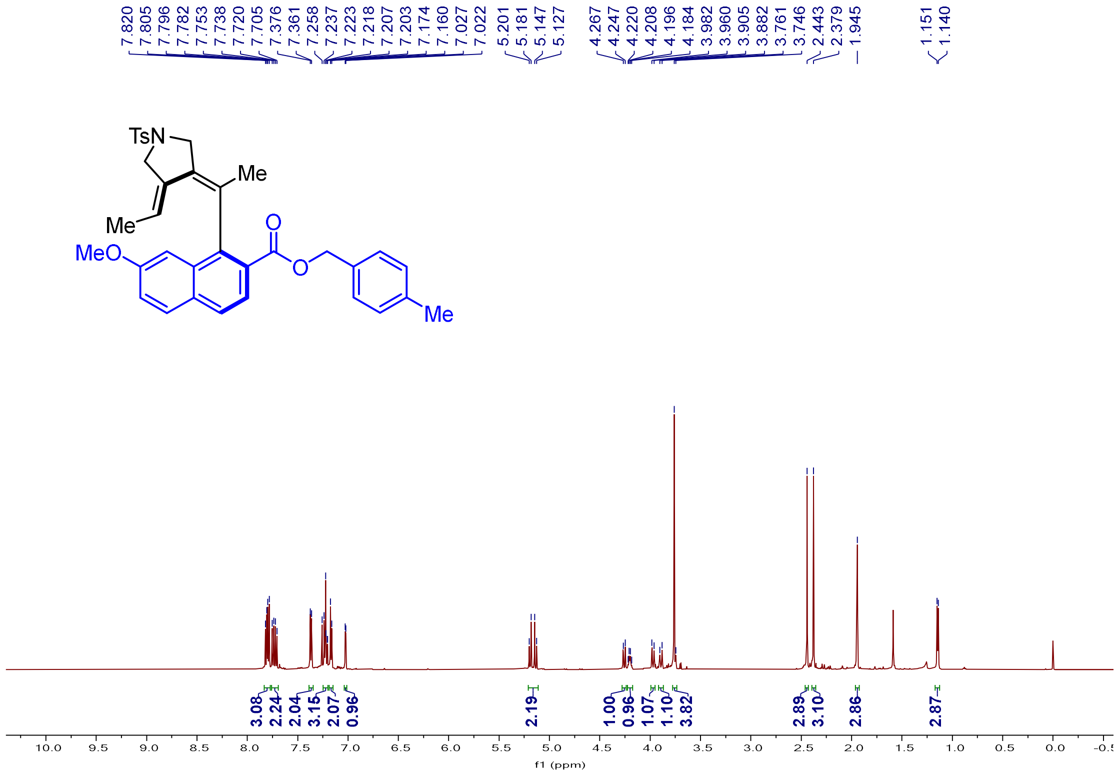

^{13}C NMR (150 MHz, Chloroform-d) spectrum of 11

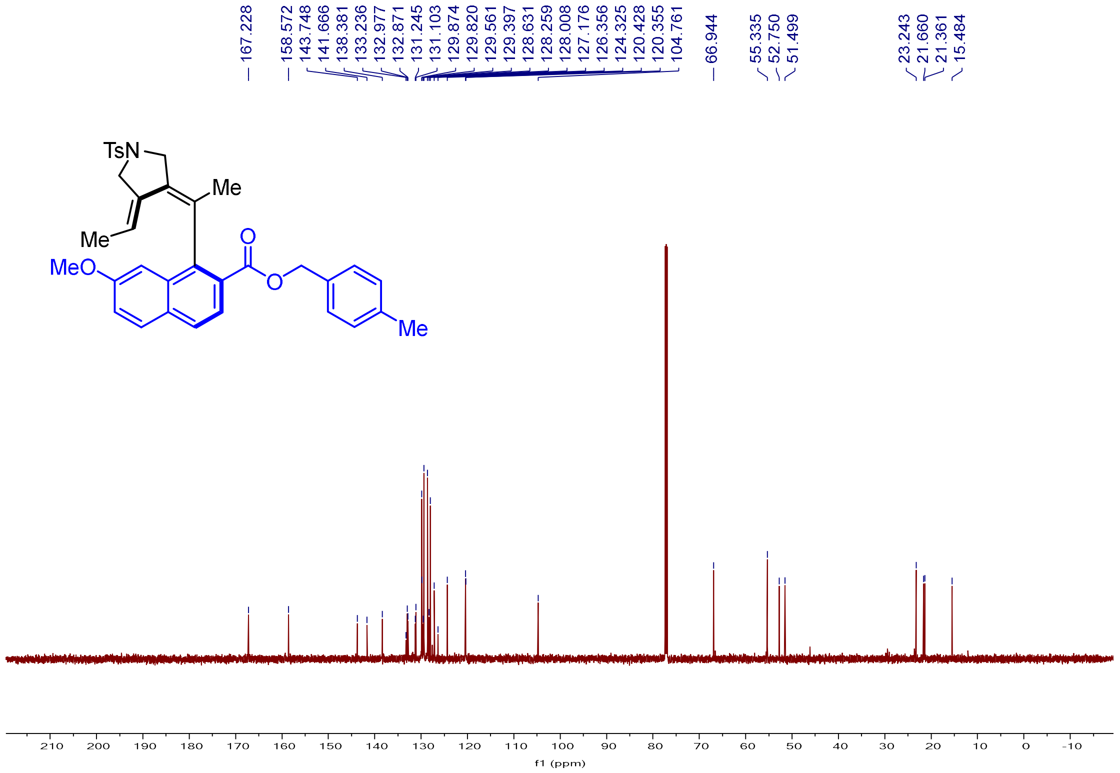

^{1}H NMR (600 MHz, Chloroform-d) spectrum of 12

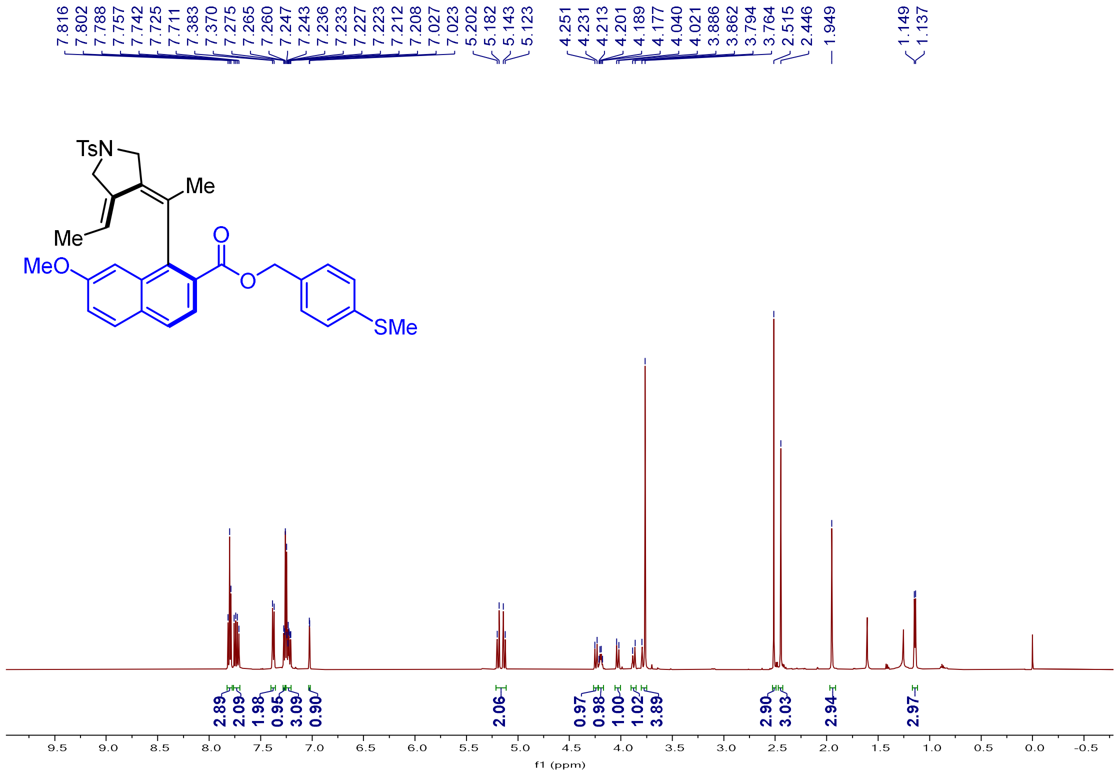

^{13}C NMR (150 MHz, Chloroform-d) spectrum of 12

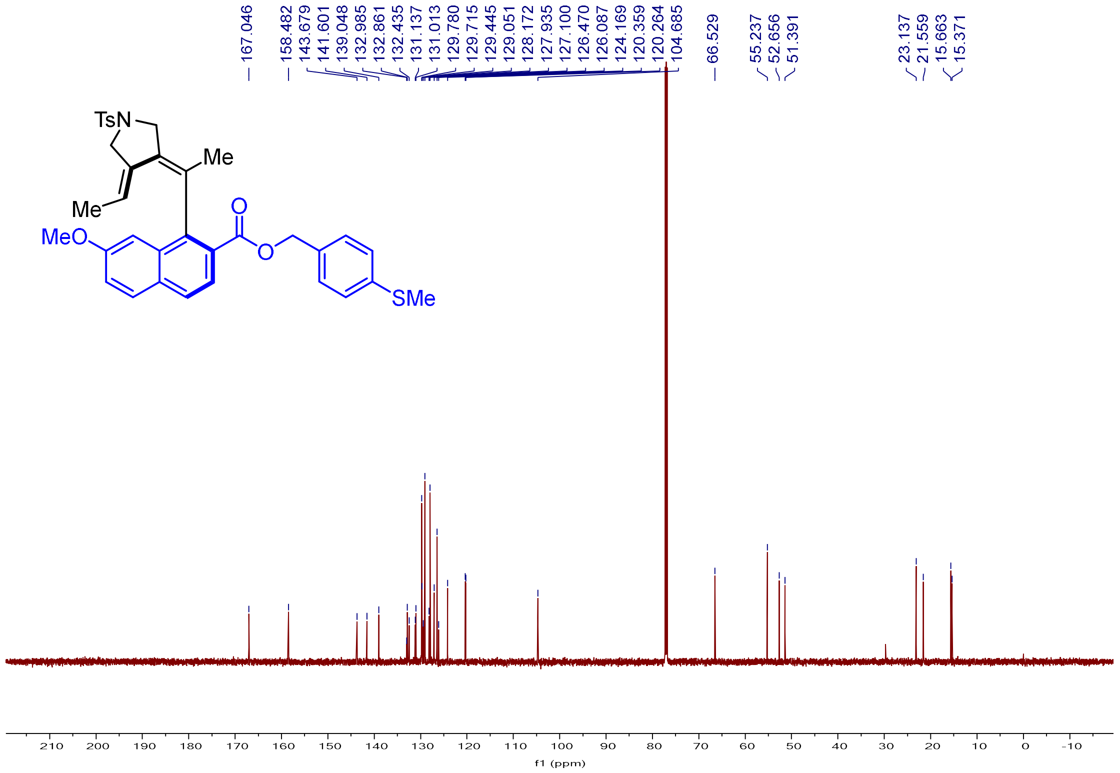

^{1}H NMR (600 MHz, Chloroform-d) spectrum of 13

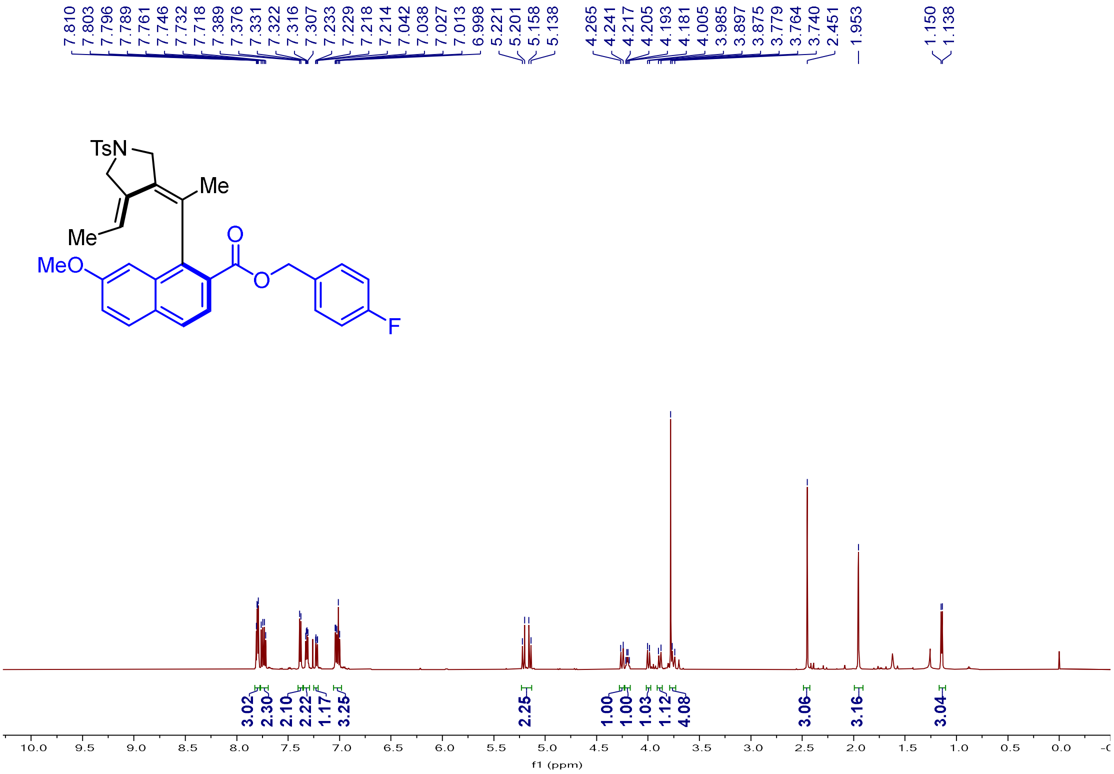

^{13}C NMR (150 MHz, Chloroform-d) spectrum of 13

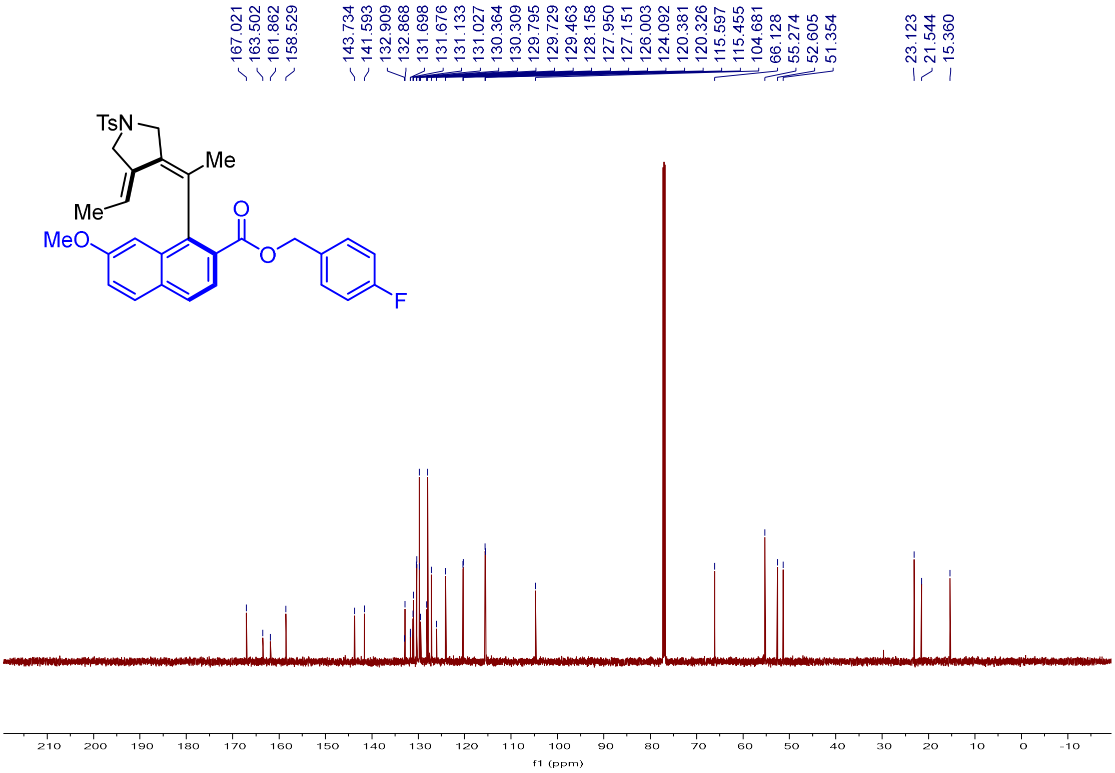

^{19}F NMR (376 MHz, Chloroform-d) spectrum of 13

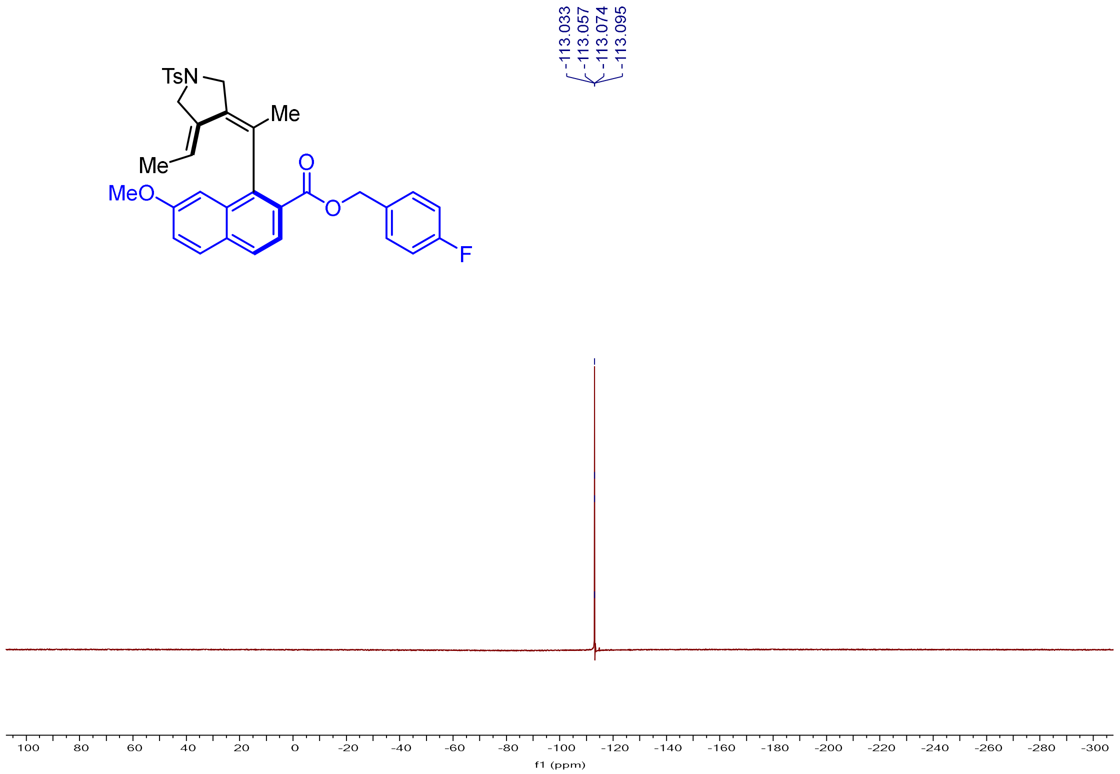

^{1}H NMR (600 MHz, Chloroform-d) spectrum of 14

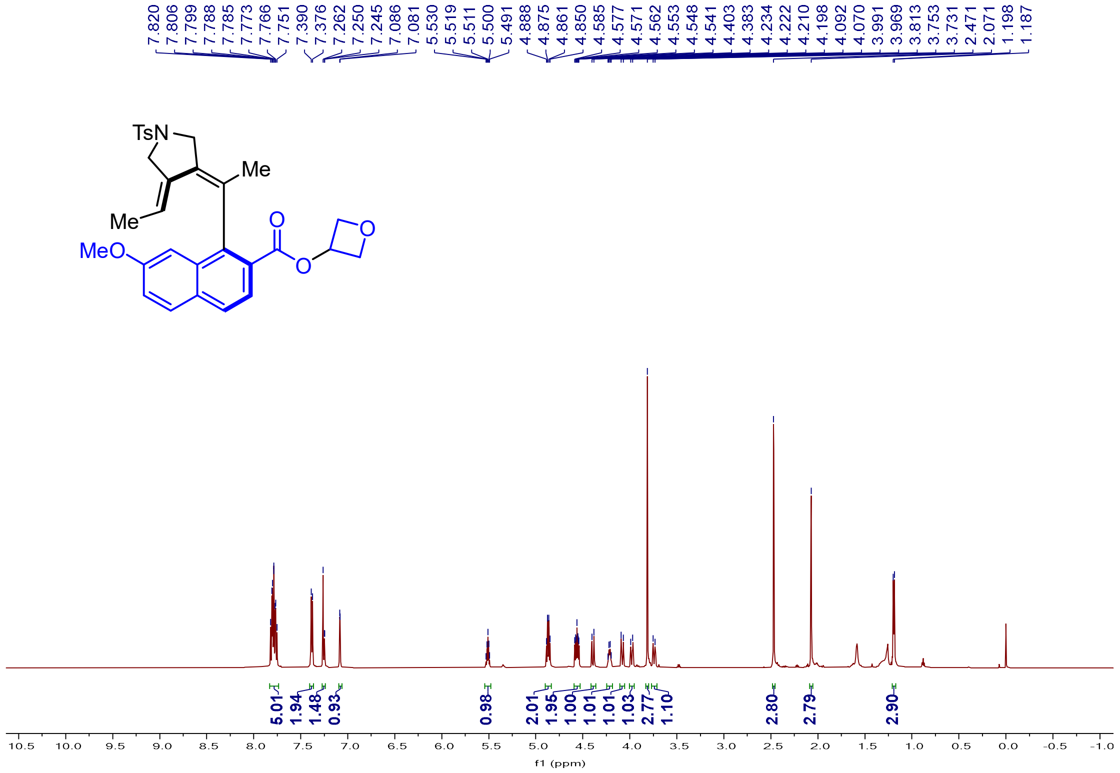

^{13}C NMR (150 MHz, Chloroform-d) spectrum of 14

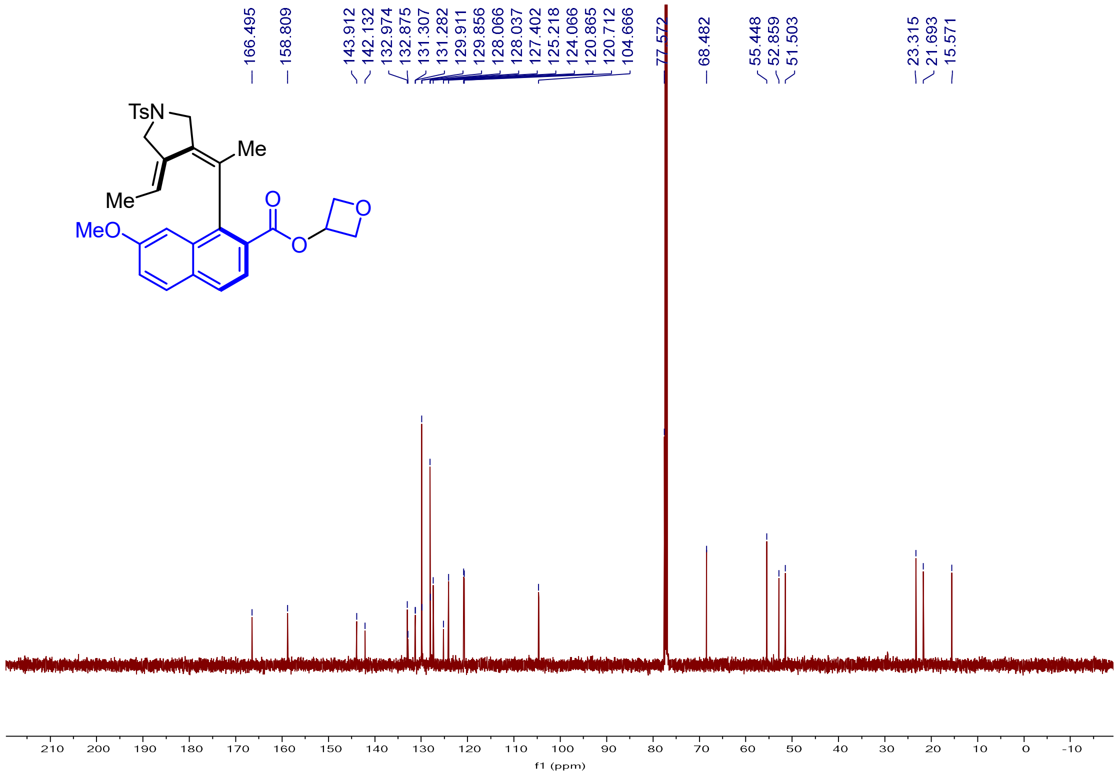

^{1}H NMR (600 MHz, Chloroform-d) spectrum of 15

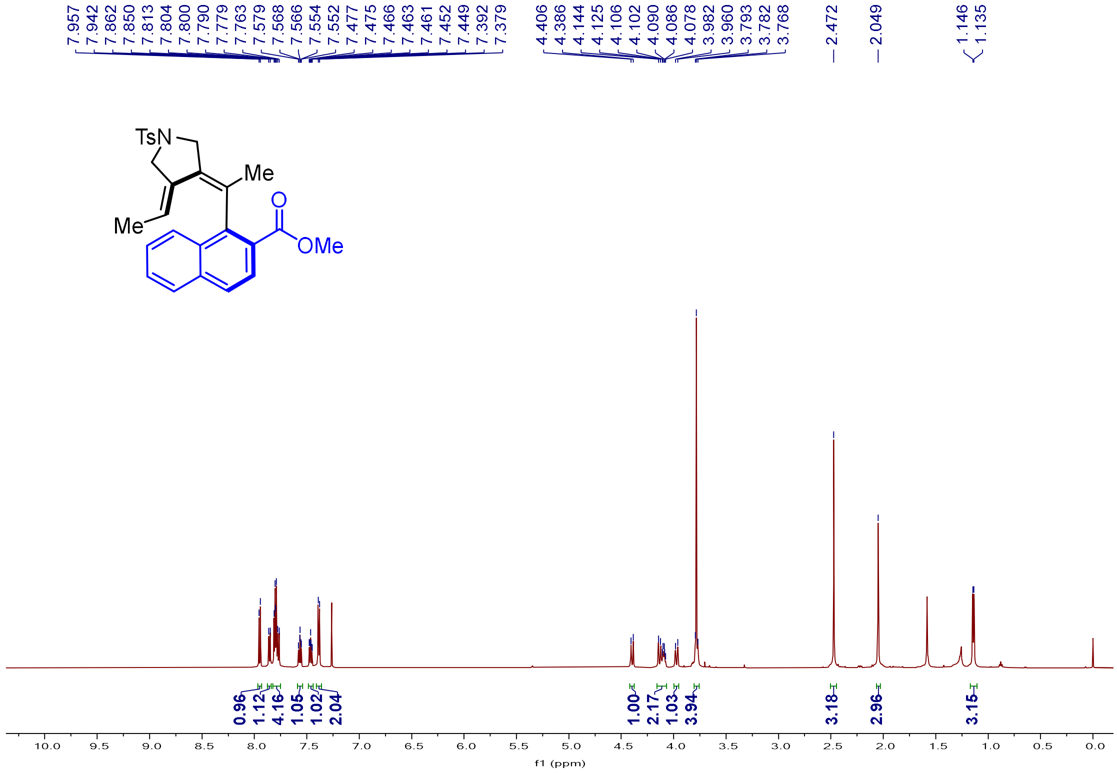

^{13}C NMR (150 MHz, Chloroform-d) spectrum of 15

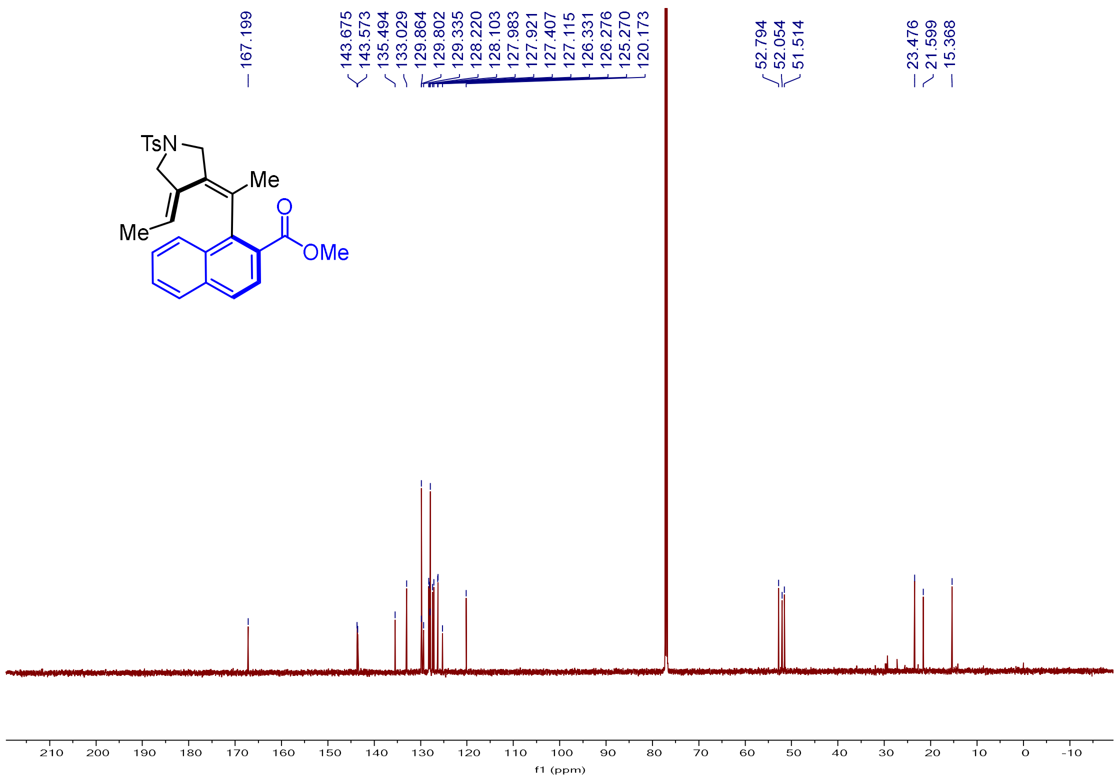

^{1}H NMR (600 MHz, Chloroform-d) spectrum of 16

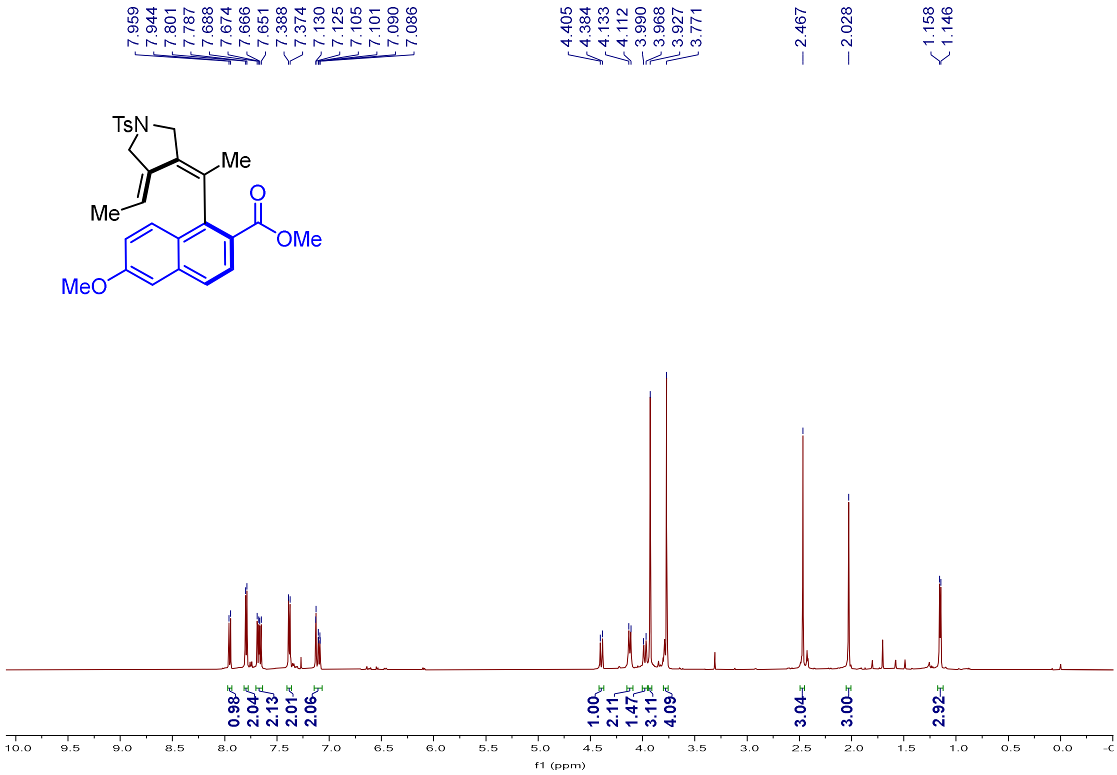

^{13}C NMR (150 MHz, Chloroform-d) spectrum of 16

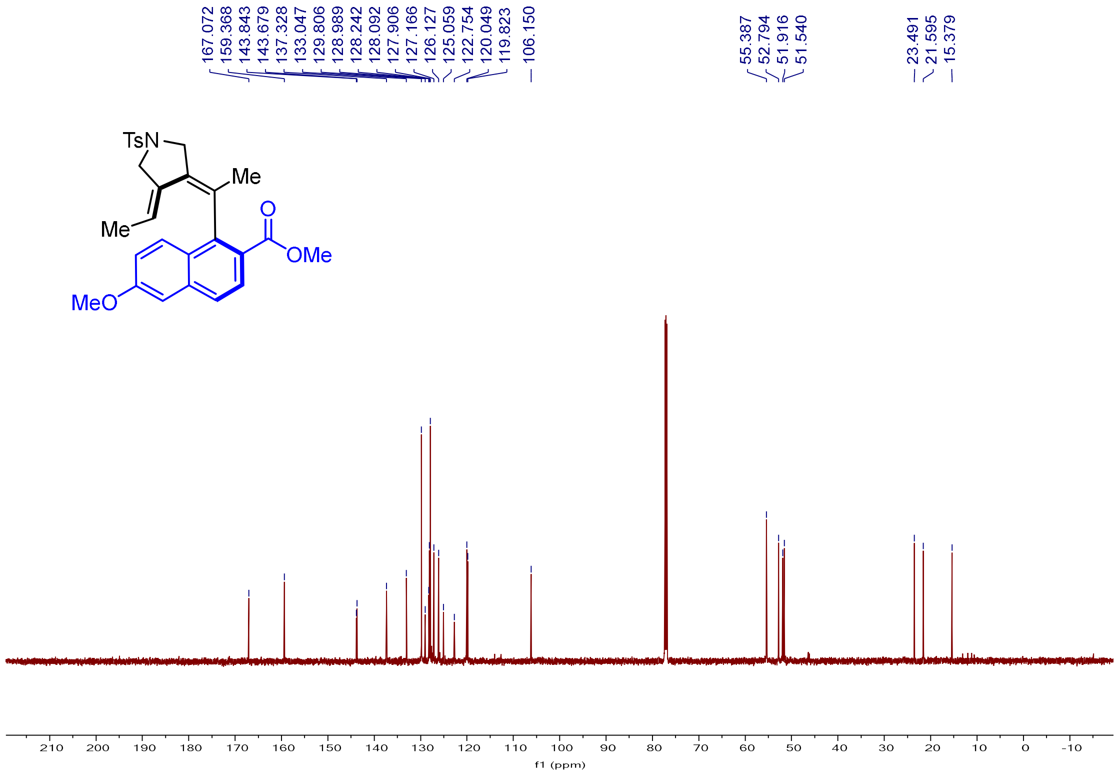

^{1}H NMR (600 MHz, Chloroform-d) spectrum of 17

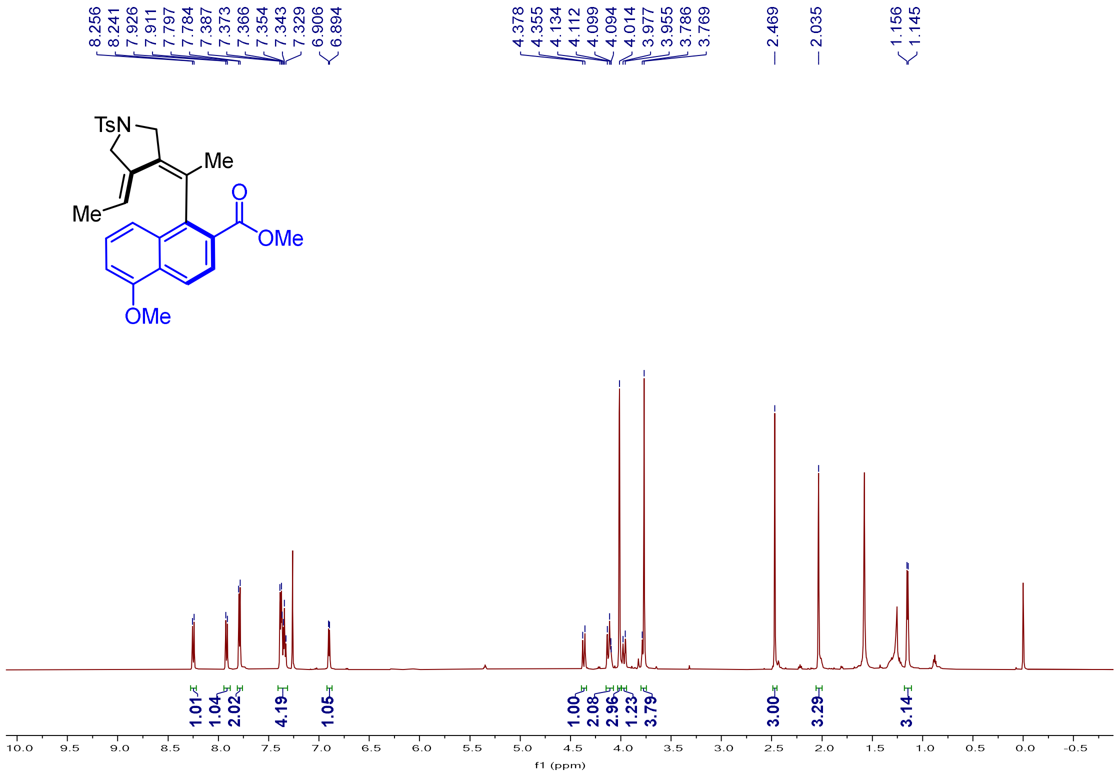

^{13}C NMR (150 MHz, Chloroform-d) spectrum of 17

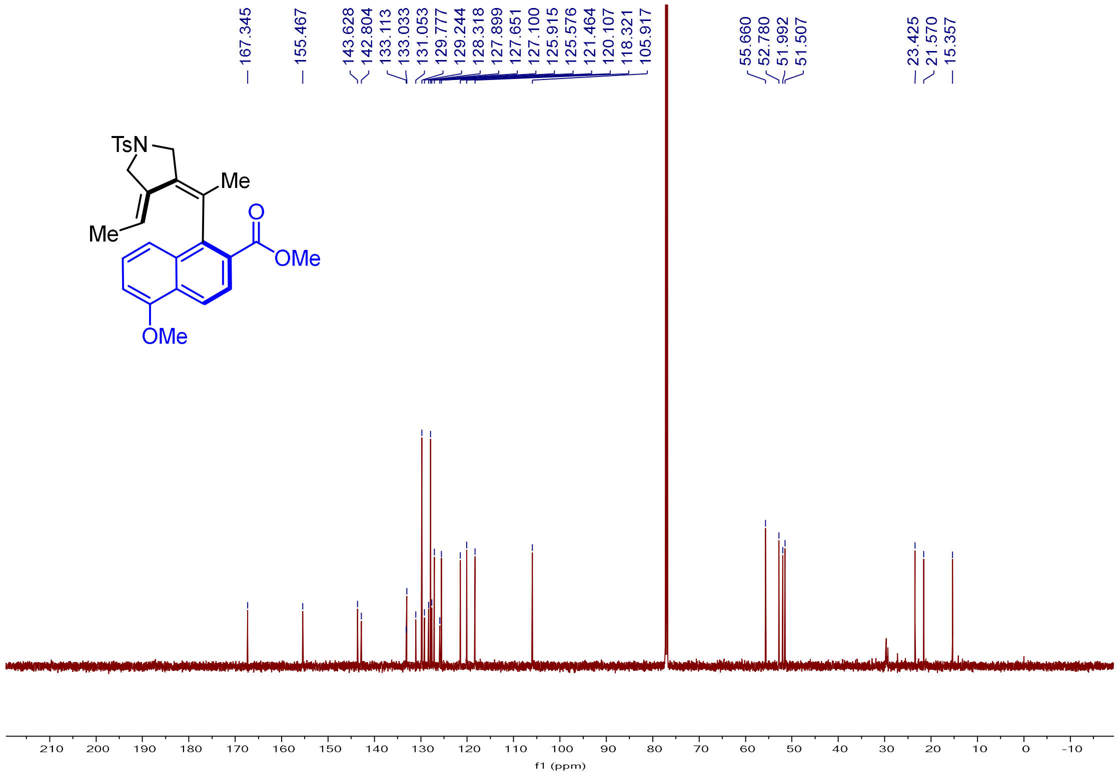

^{1}H NMR (600 MHz, Chloroform-d) spectrum of 18

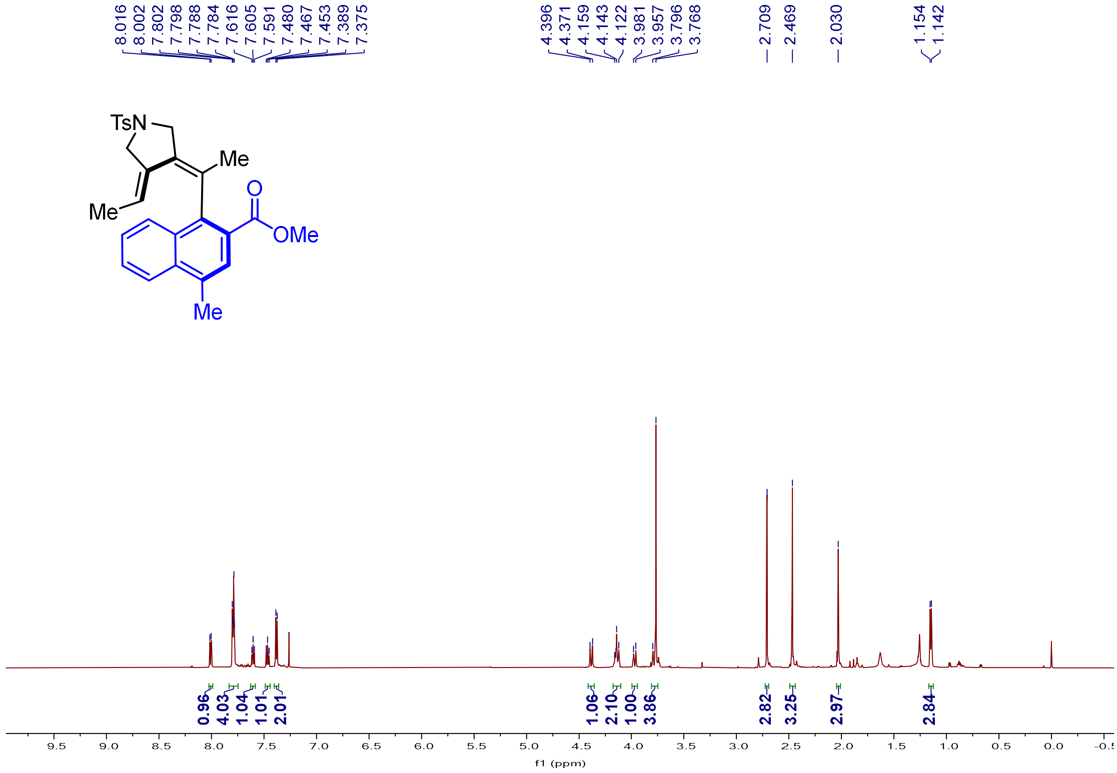

^{13}C NMR (150 MHz, Chloroform-d) spectrum of 18

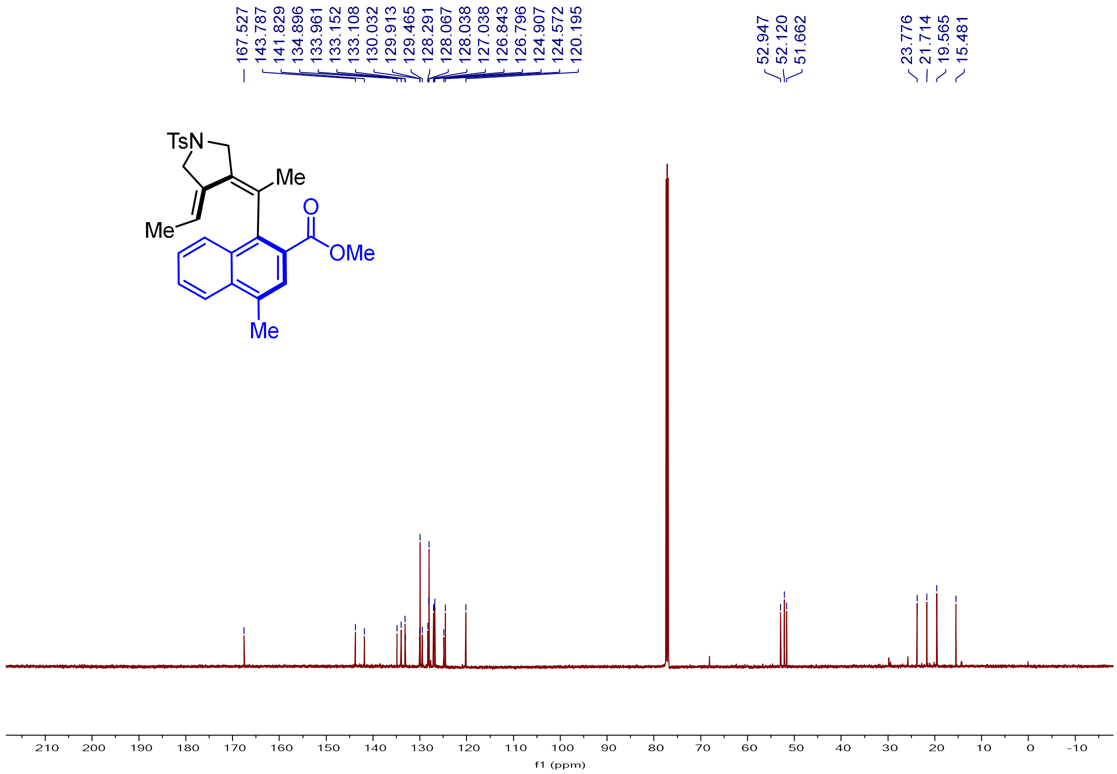

^{1}H NMR (600 MHz, Chloroform-d) spectrum of 19

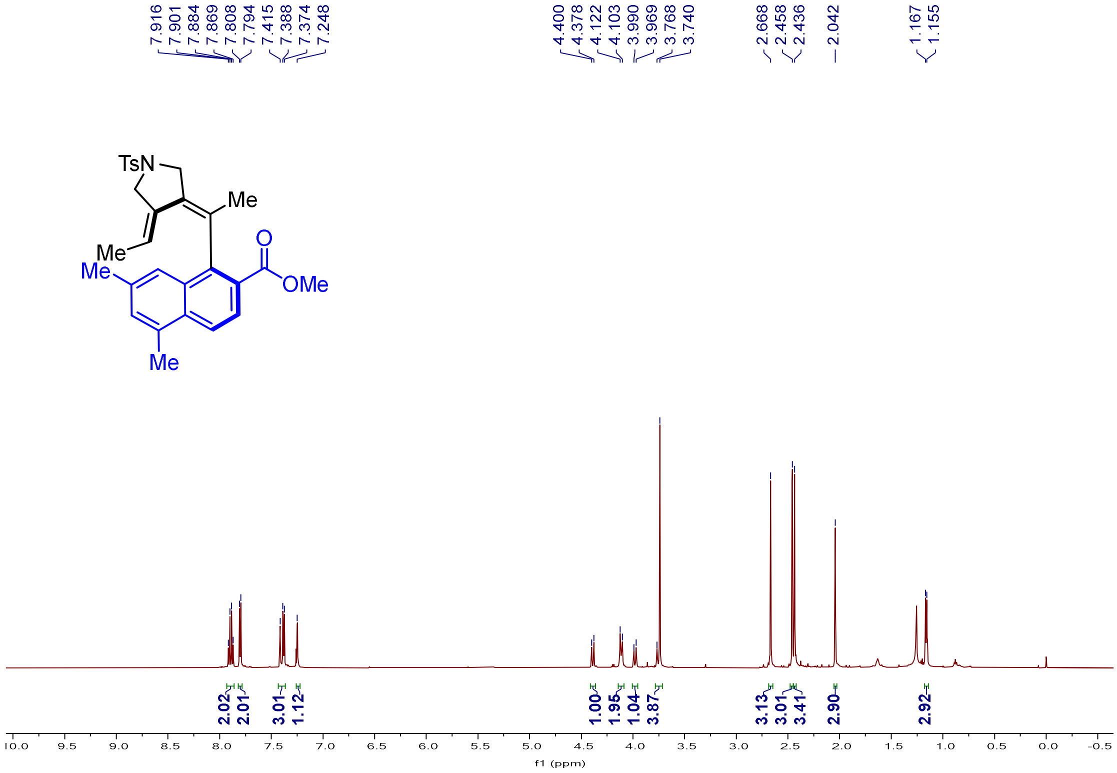

^{13}C NMR (150 MHz, Chloroform-d) spectrum of 19

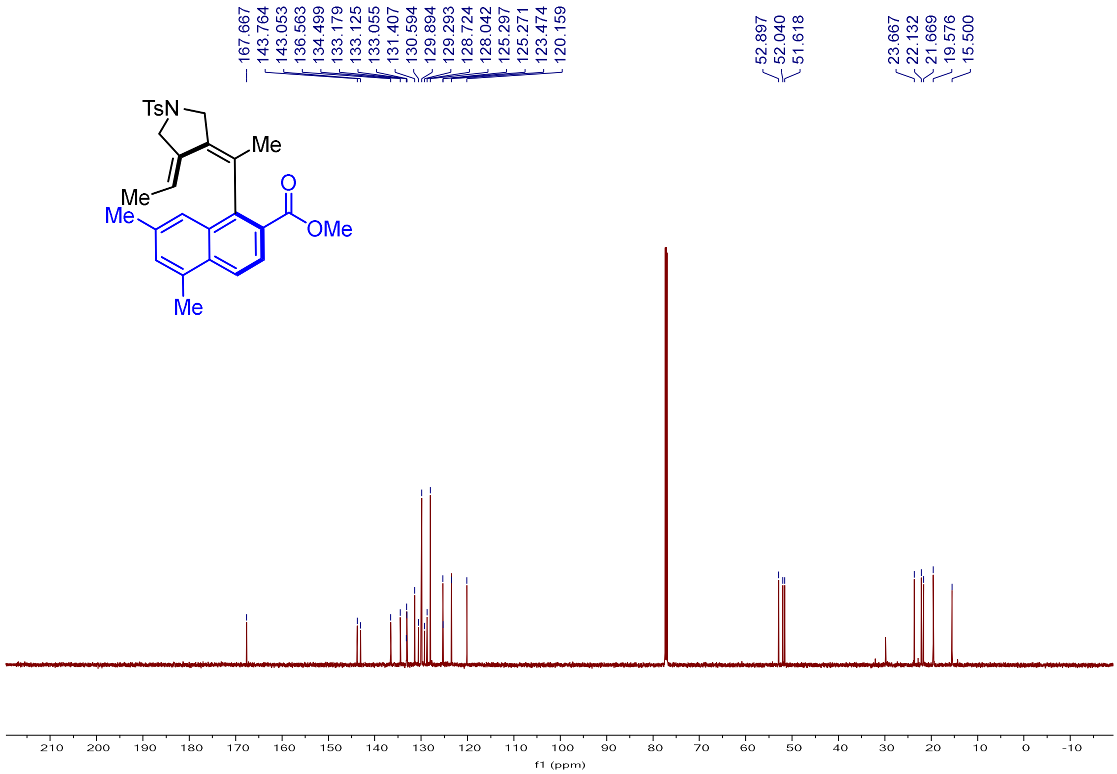

^{1}H NMR (600 MHz, Chloroform-d) spectrum of 20

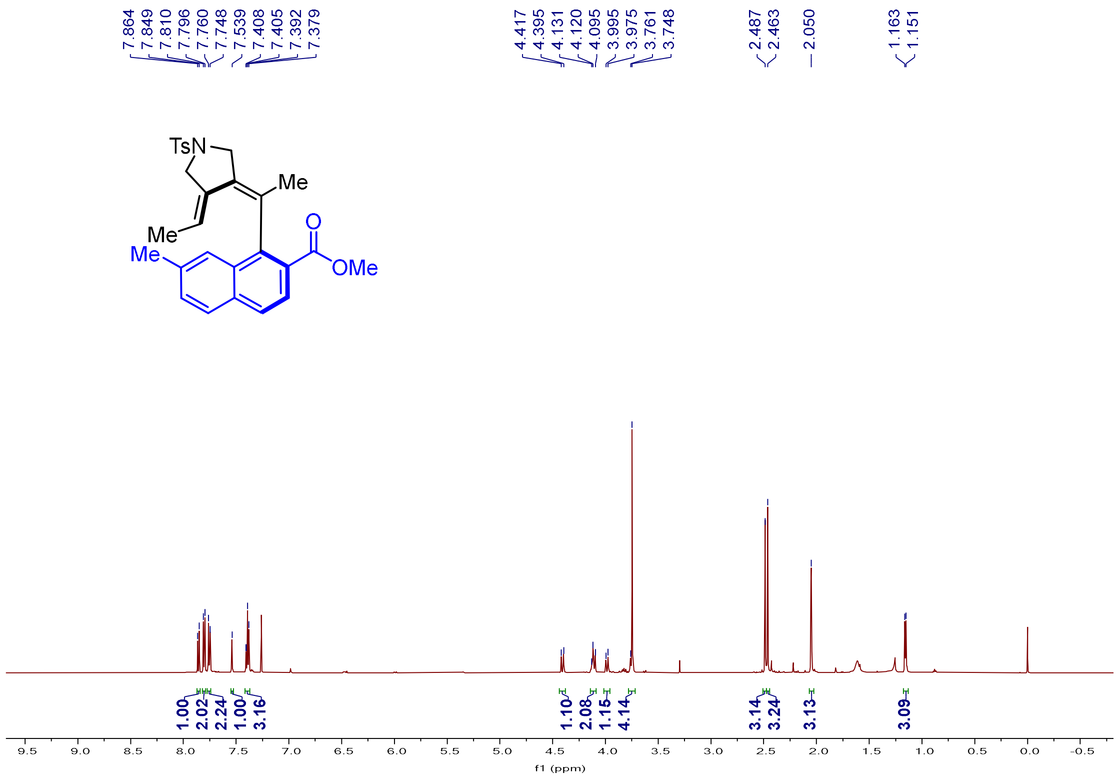

^{13}C NMR (150 MHz, Chloroform-d) spectrum of 20

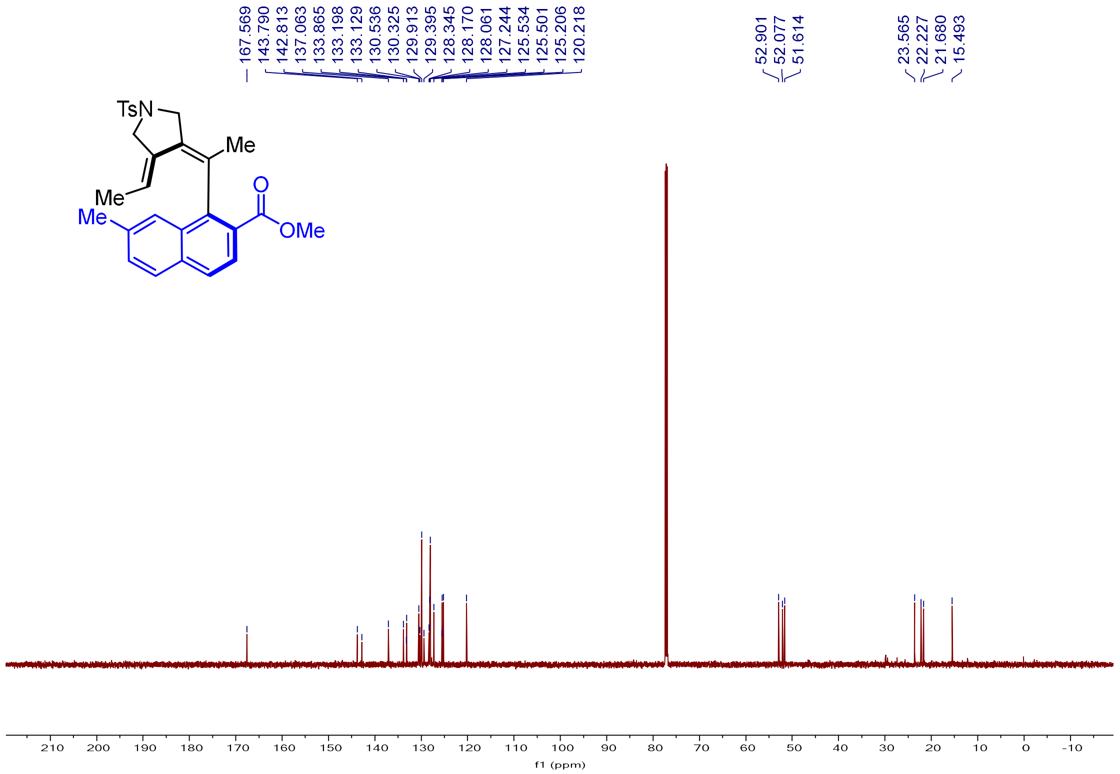

^{1}H NMR (600 MHz, Chloroform-d) spectrum of 21

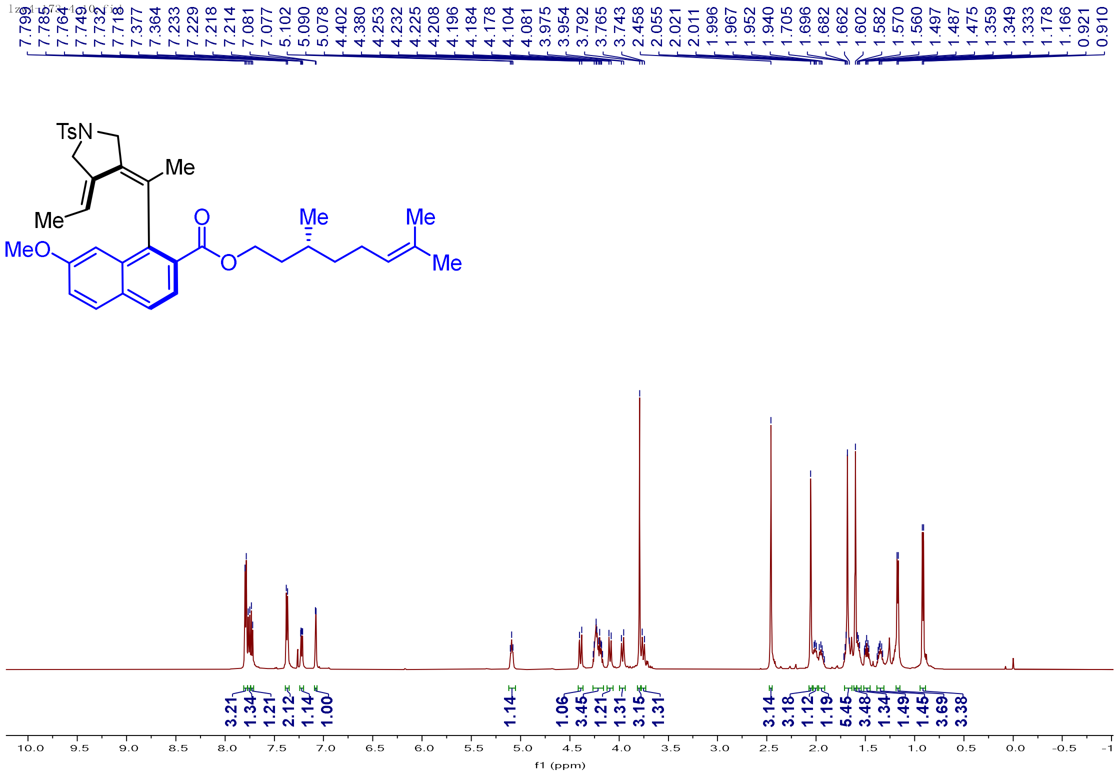

^{13}C NMR (150 MHz, Chloroform-d) spectrum of 21

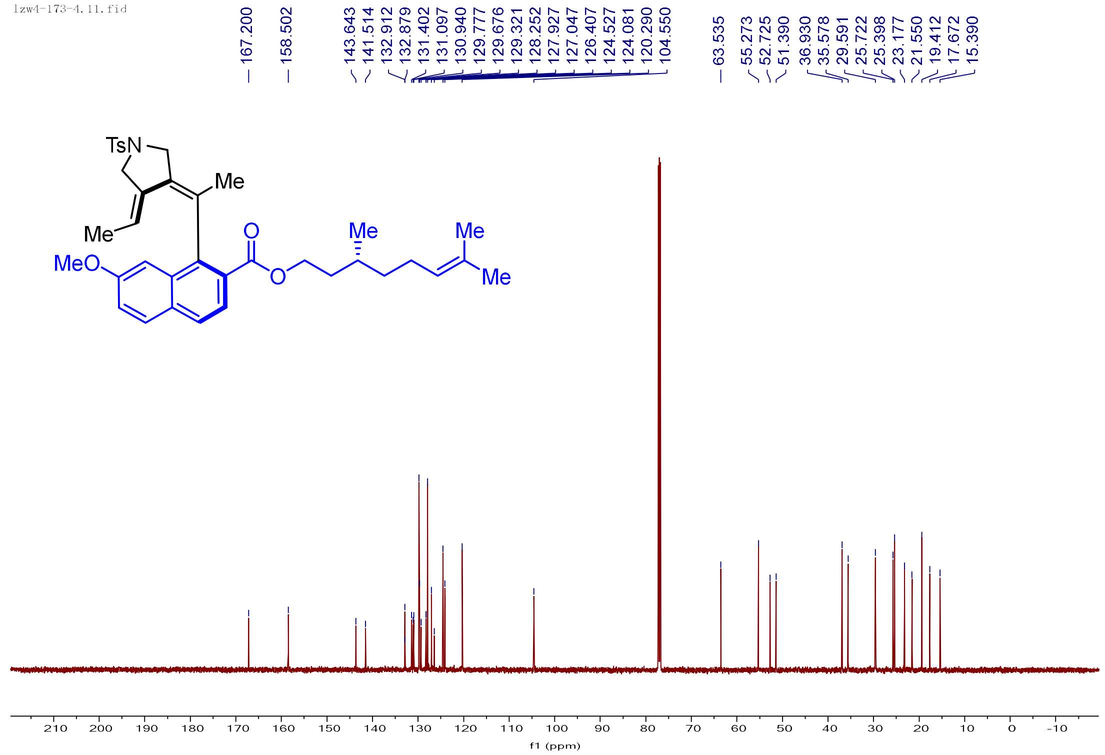

^{1}H NMR (600 MHz, Chloroform-d) spectrum of 22

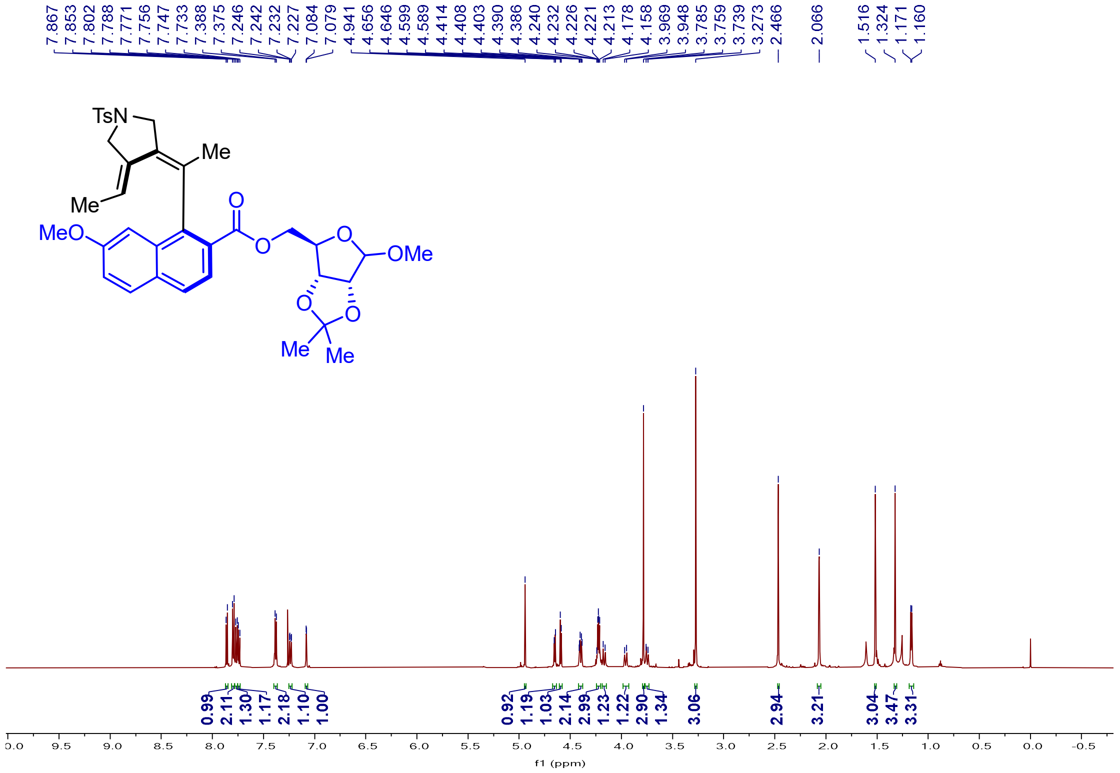

^{13}C NMR (150 MHz, Chloroform-d) spectrum of 22

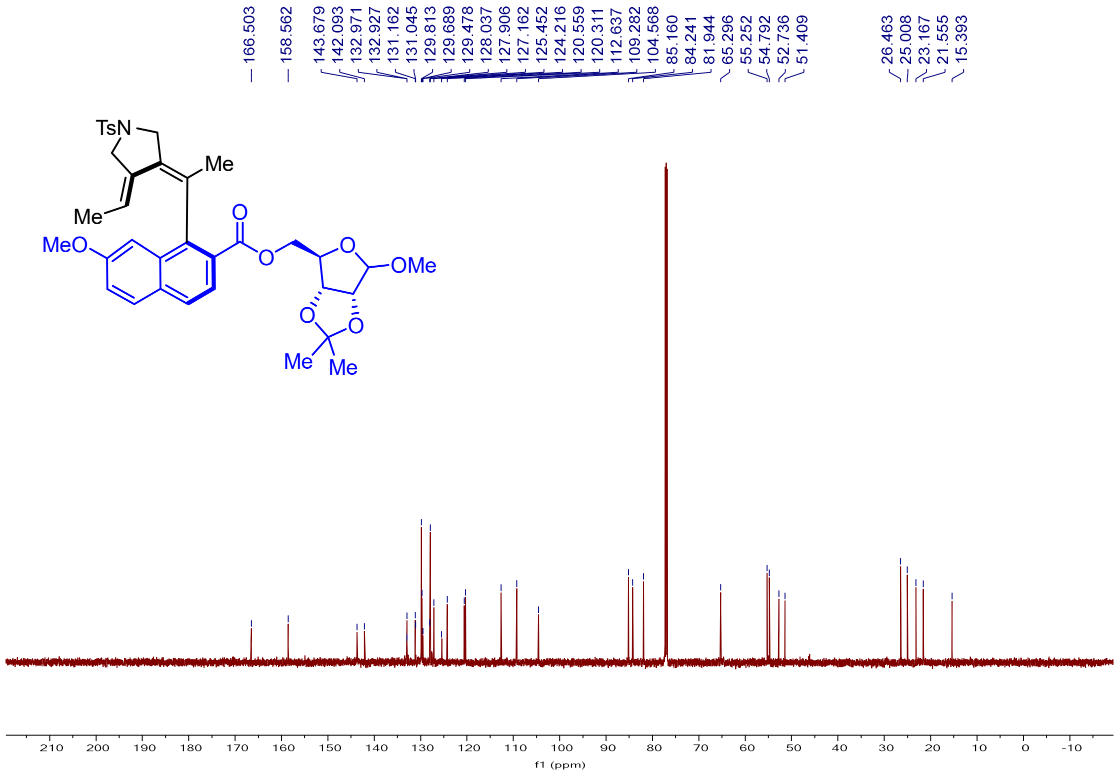

^{1}H NMR (600 MHz, Chloroform-d) spectrum of 23

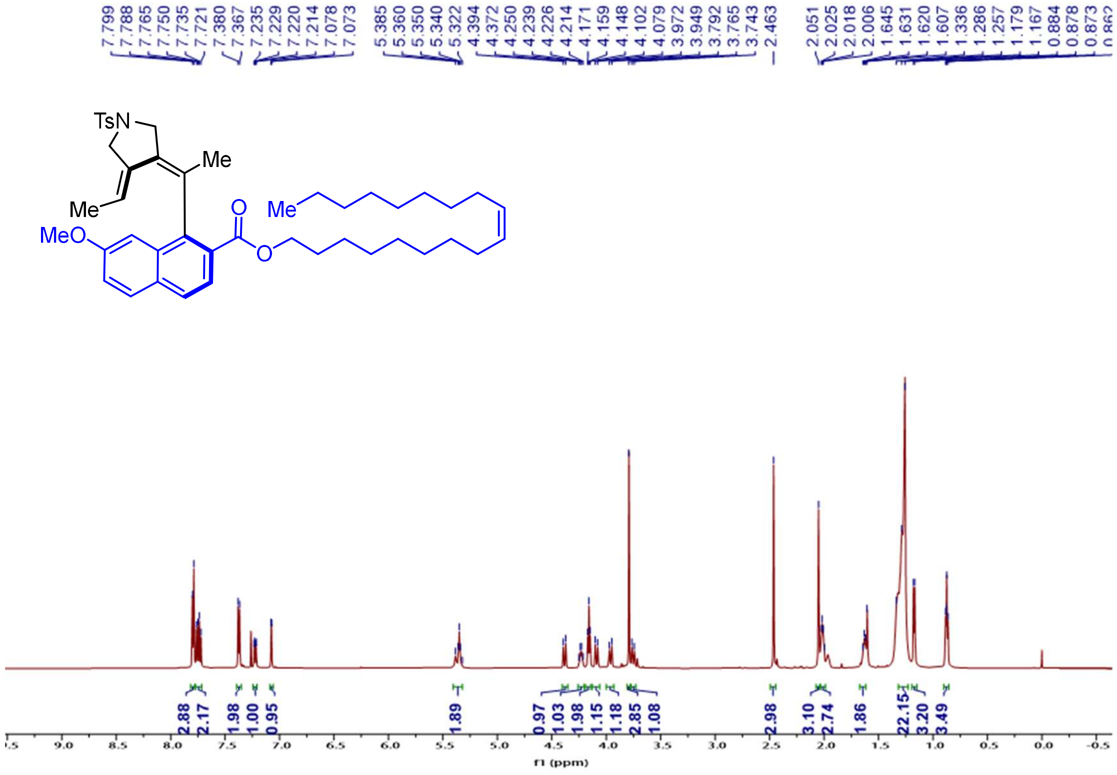

^{13}C NMR (150 MHz, Chloroform-d) spectrum of 23

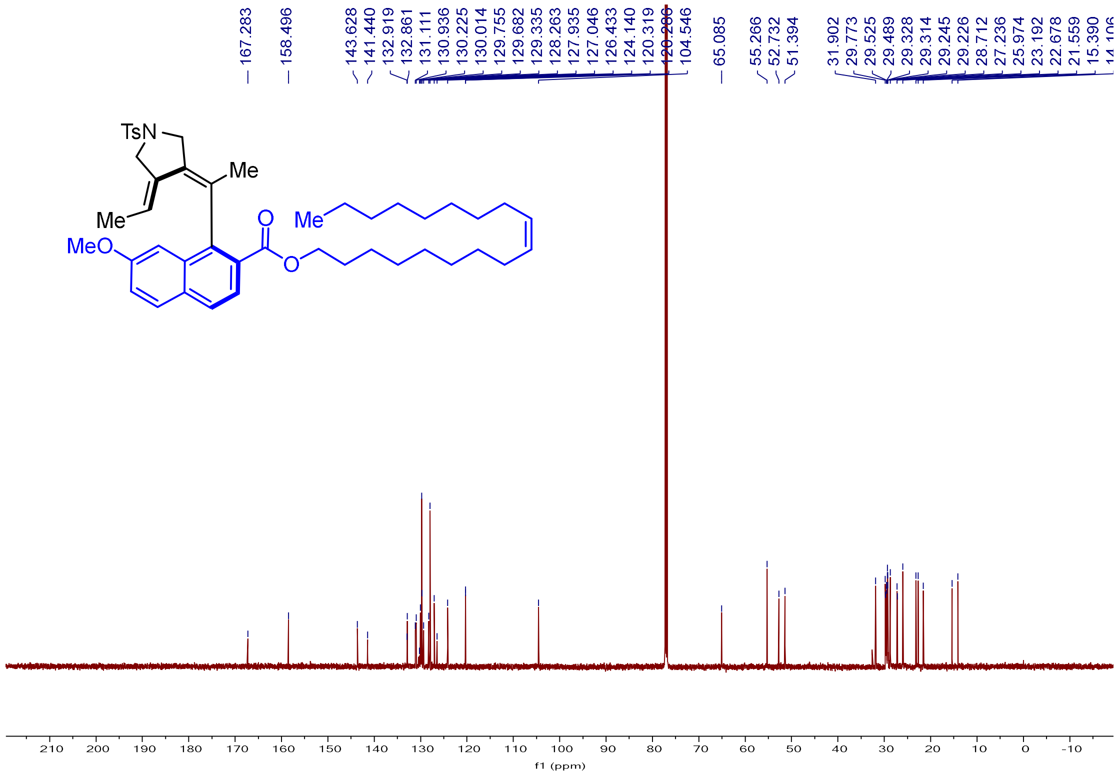

^{1}H NMR (600 MHz, Chloroform-d) spectrum of 24

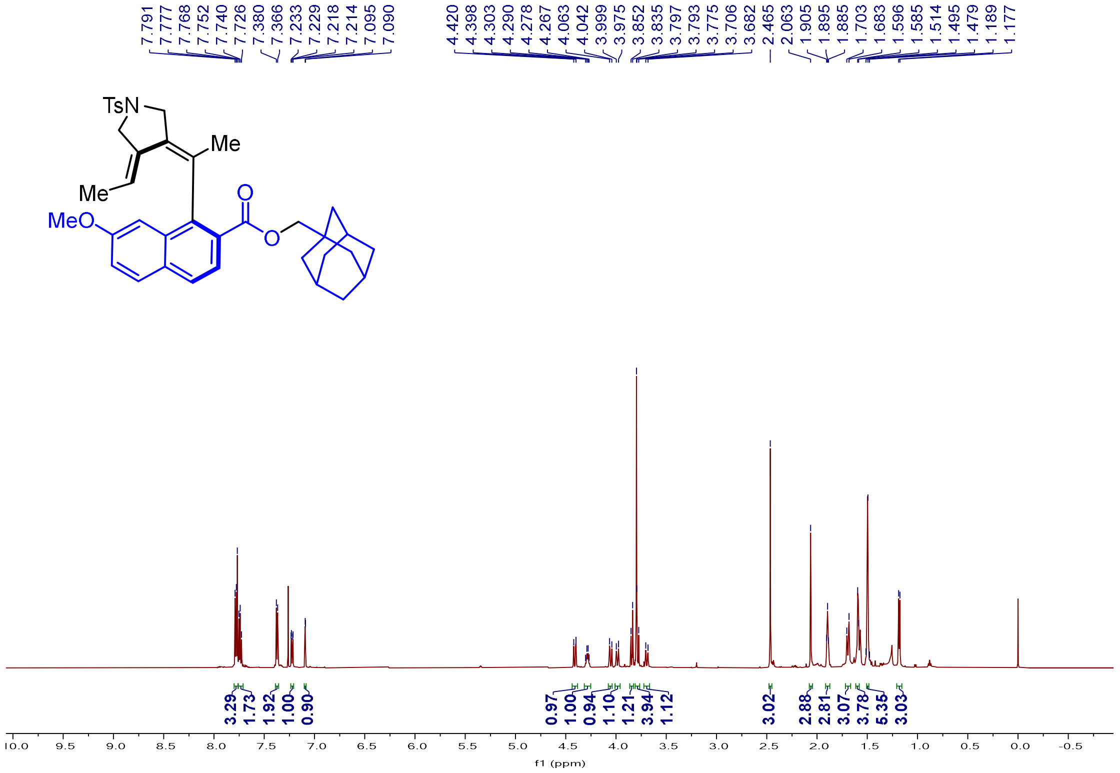

^{13}C NMR (150 MHz, Chloroform-d) spectrum of 24

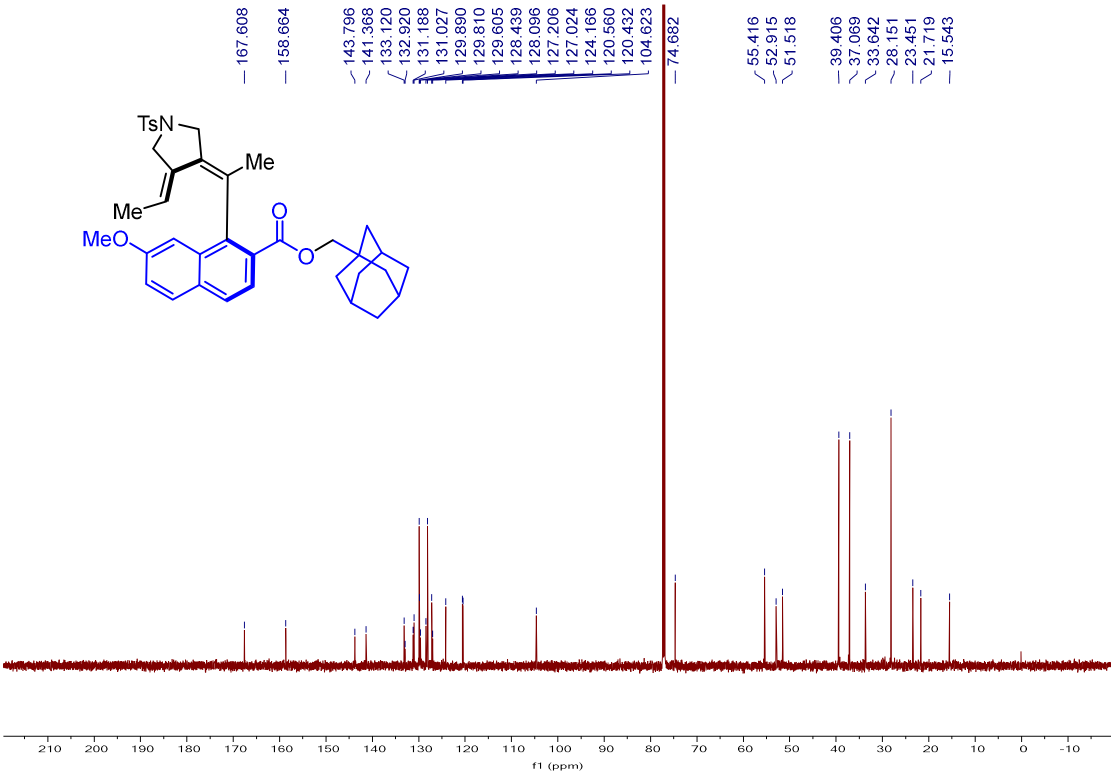

^{1}H NMR (600 MHz, Chloroform-d) spectrum of 25

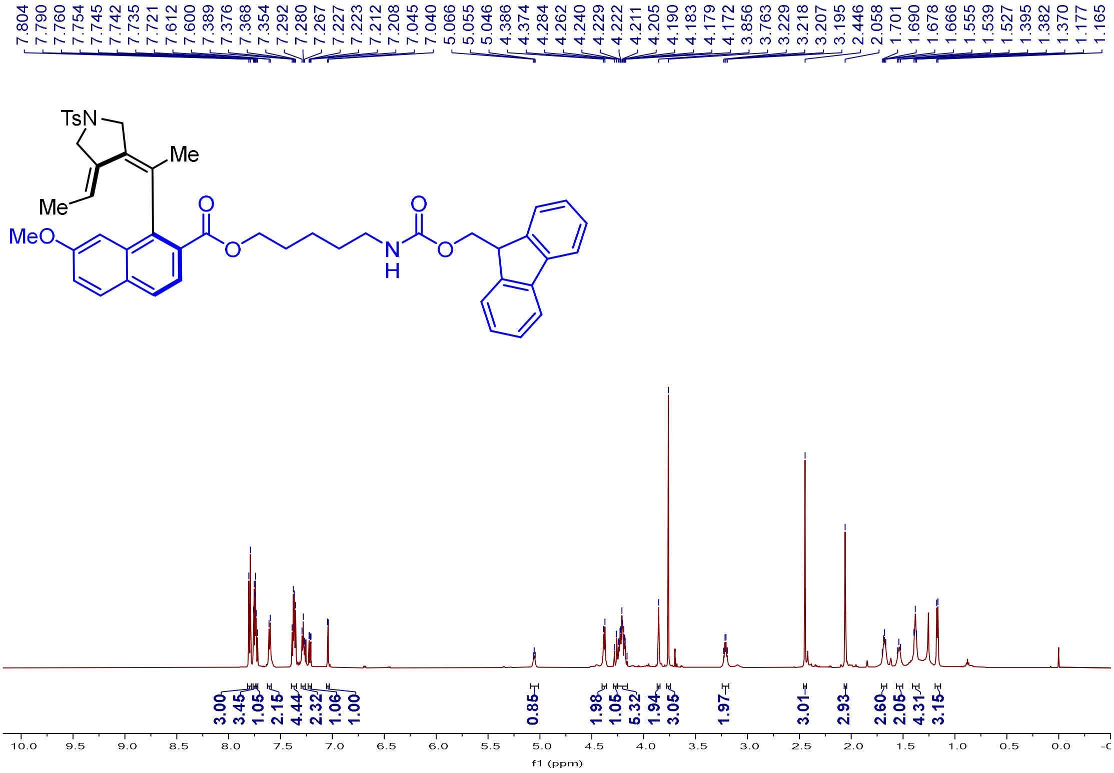

^{13}C NMR (150 MHz, Chloroform-d) spectrum of 25

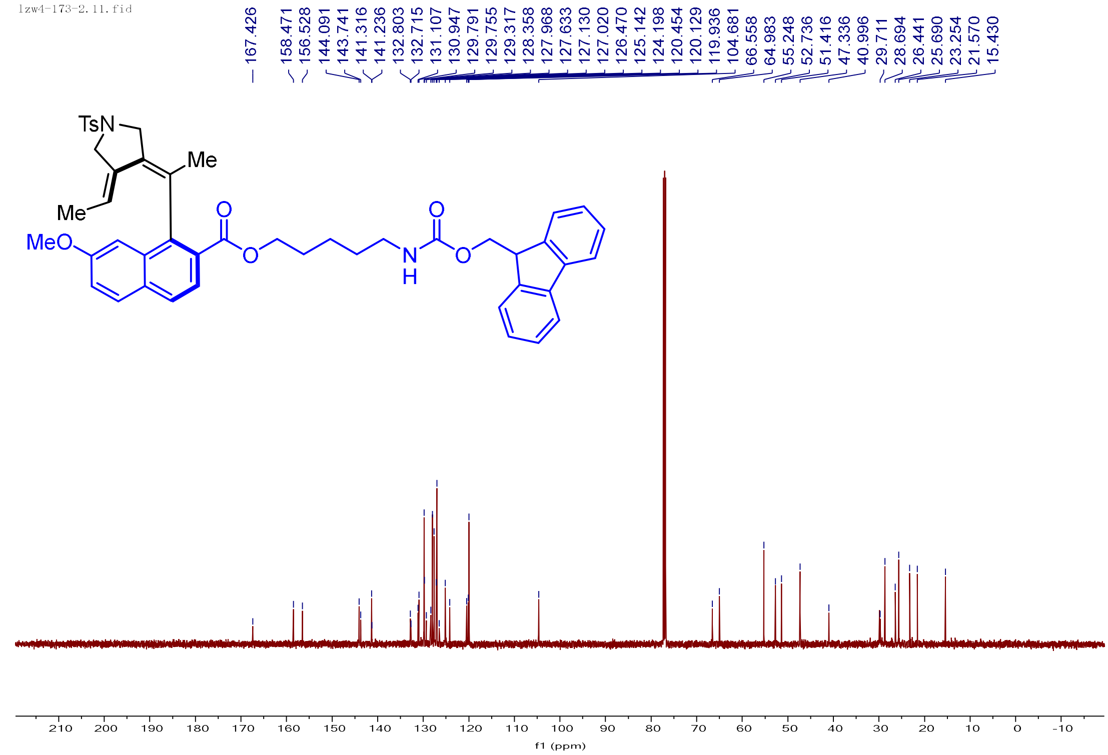

^{1}H NMR (600 MHz, Chloroform-d) spectrum of 10-*d*

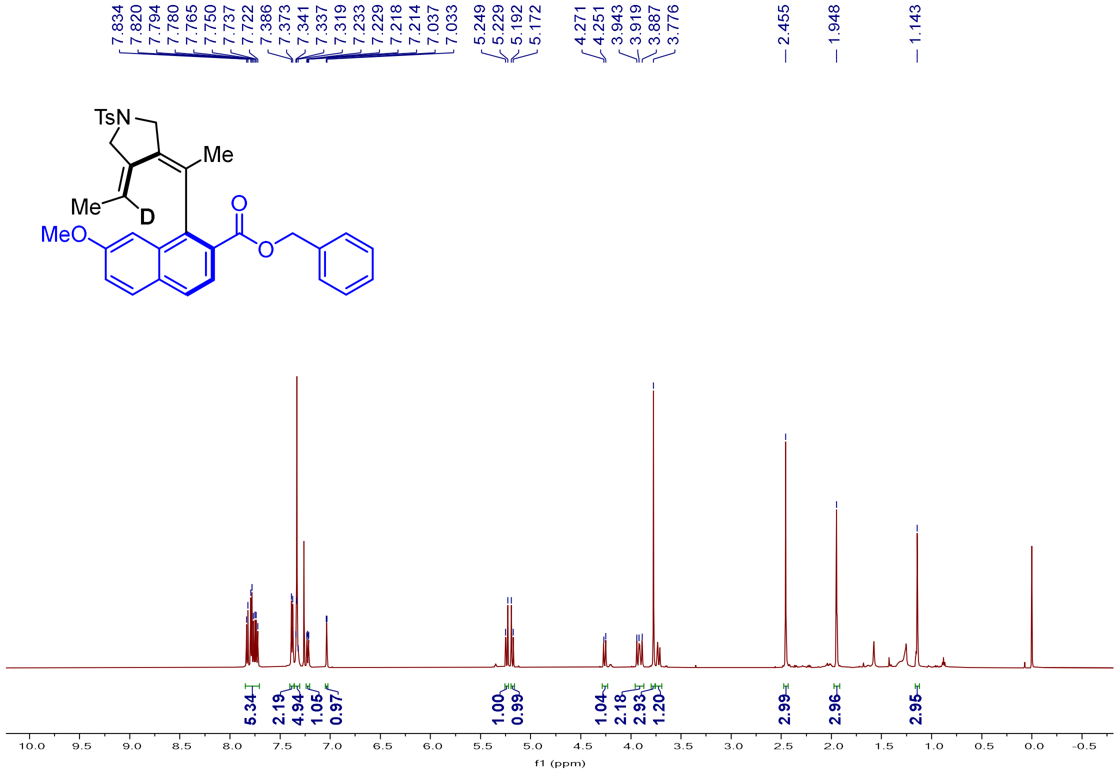

^{13}C NMR (150 MHz, Chloroform-d) spectrum of 10-*d*

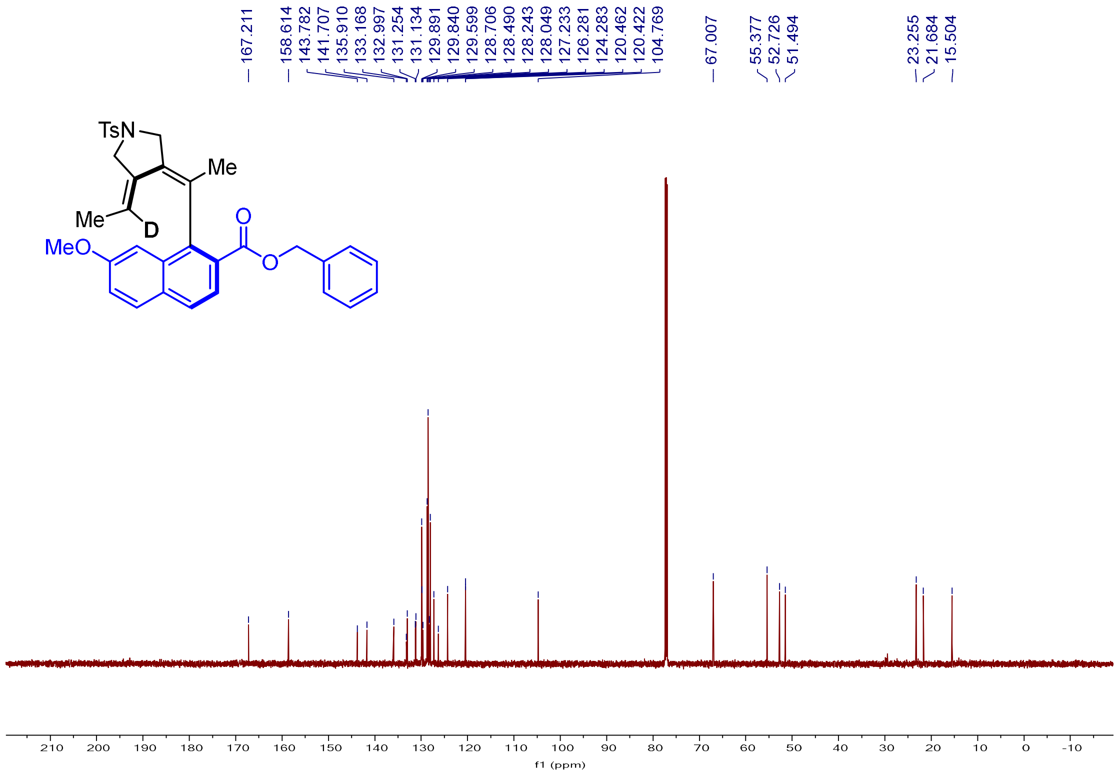

^{1}H NMR (600 MHz, Chloroform-d) spectrum of 20-*d*

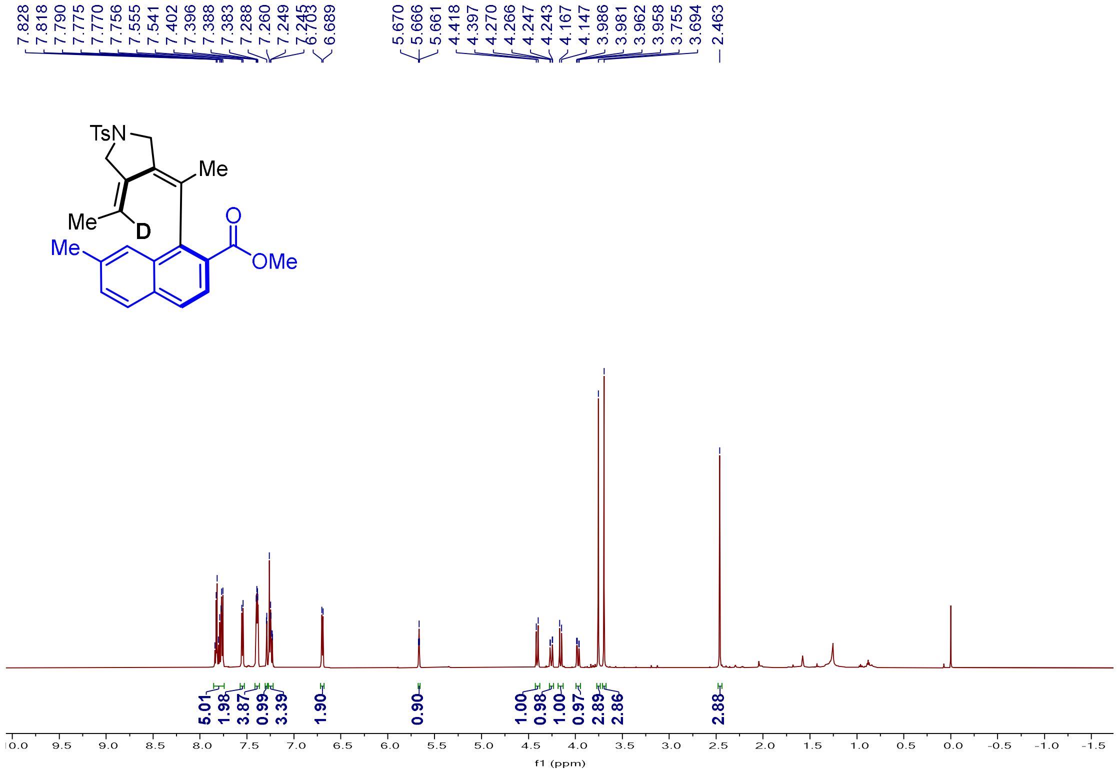

^{13}C NMR (150 MHz, Chloroform-d) spectrum of 20-*d*

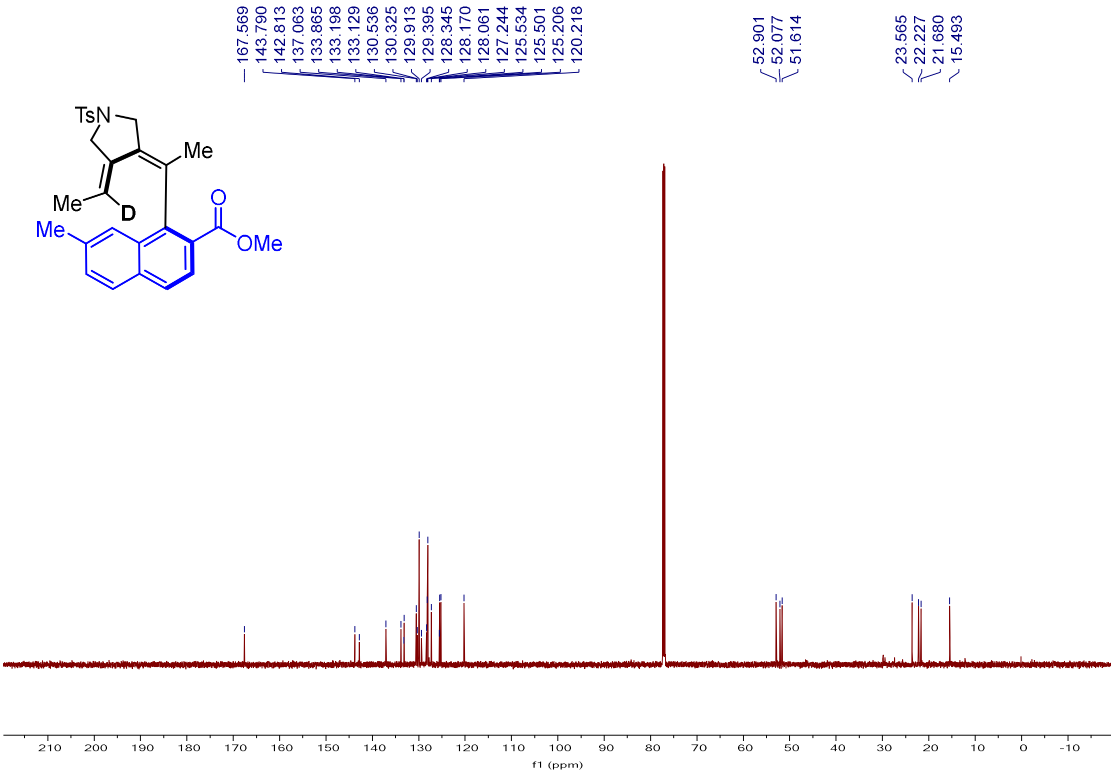

^{1}H NMR (600 MHz, Chloroform-d) spectrum of 21-*d*

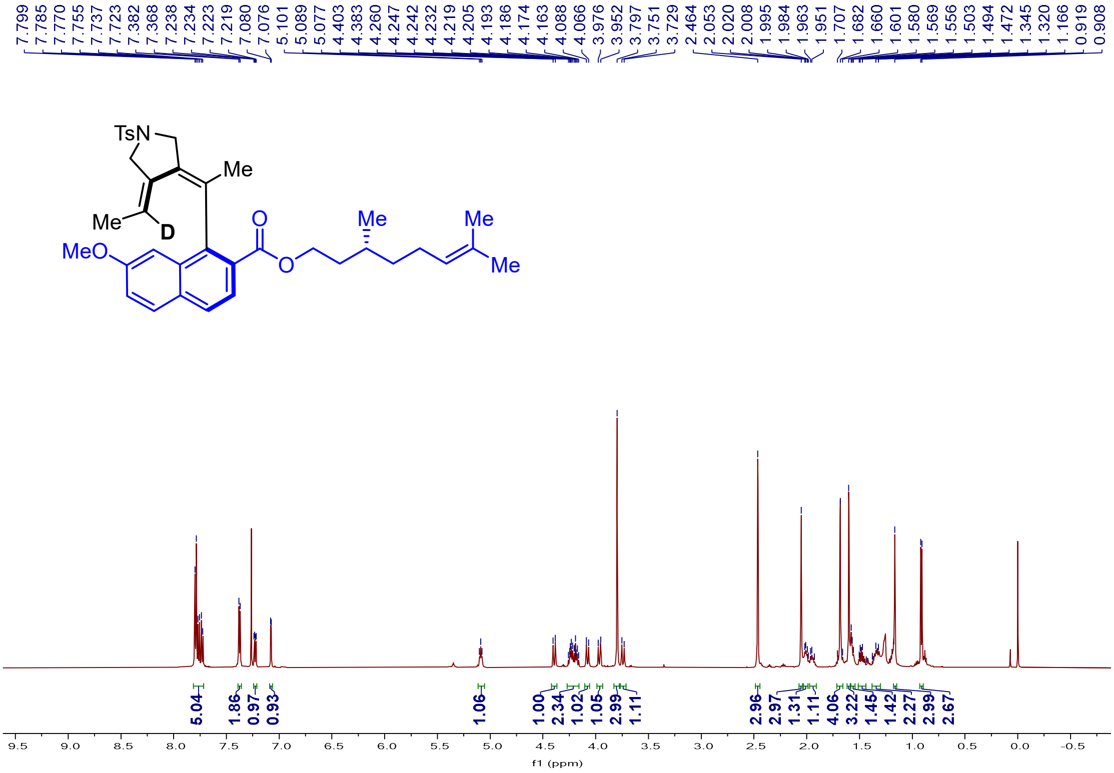

^{13}C NMR (150 MHz, Chloroform-d) spectrum of 21-*d*

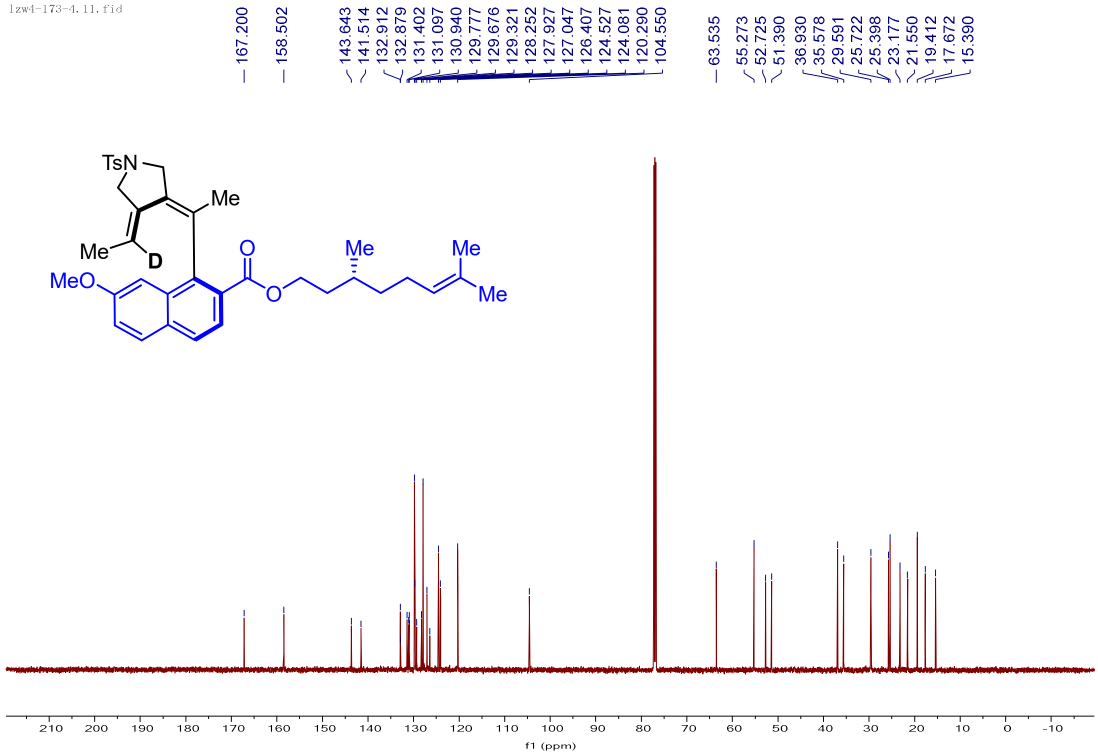

^{1}H NMR (600 MHz, Chloroform-d) spectrum of 26

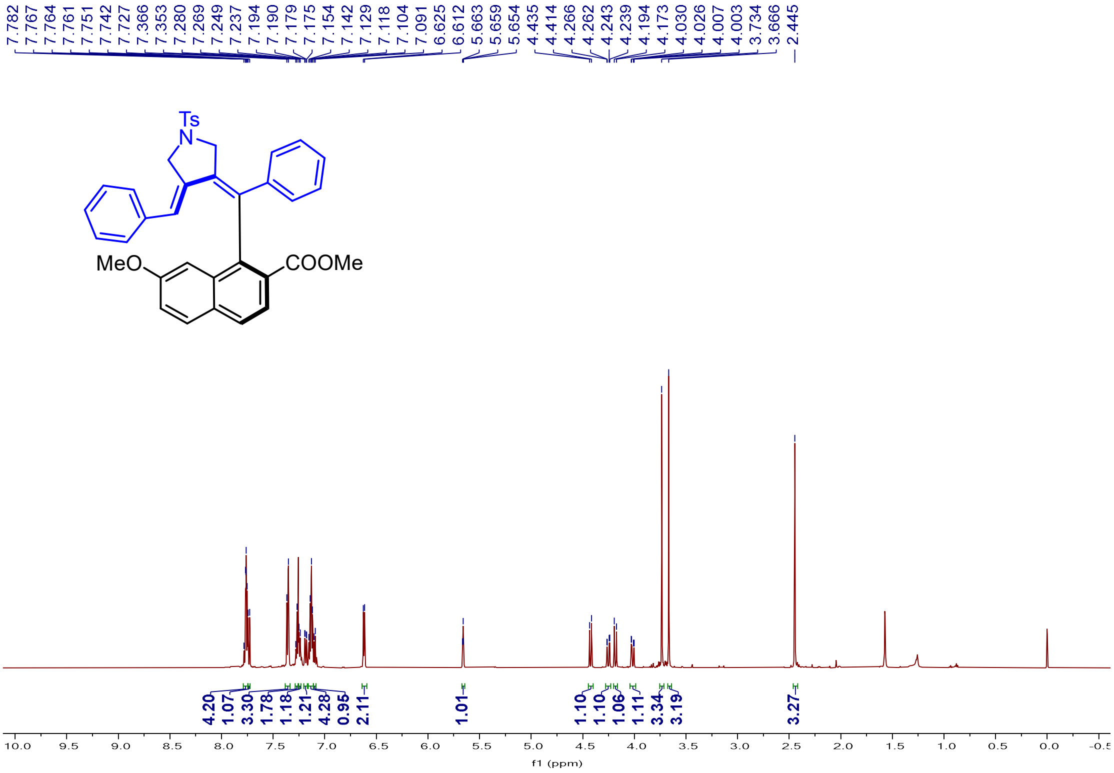

^{13}C NMR (150 MHz, Chloroform-d) spectrum of 26

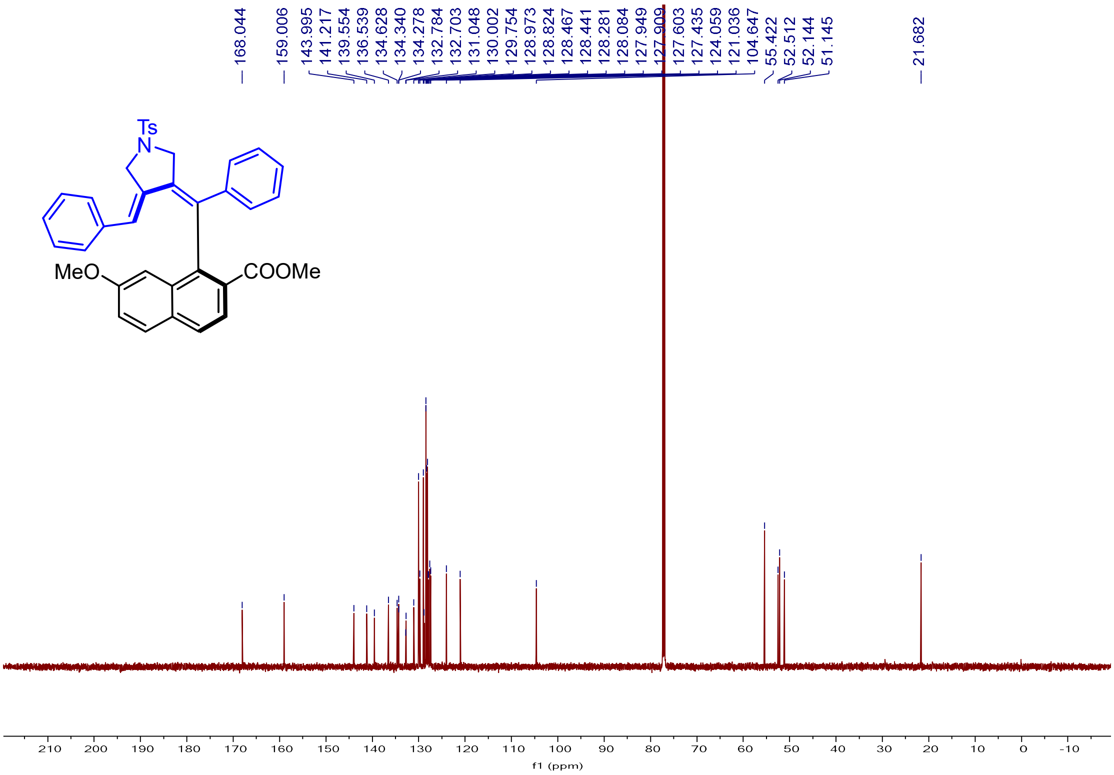

^{1}H NMR (600 MHz, Chloroform-d) spectrum of 27

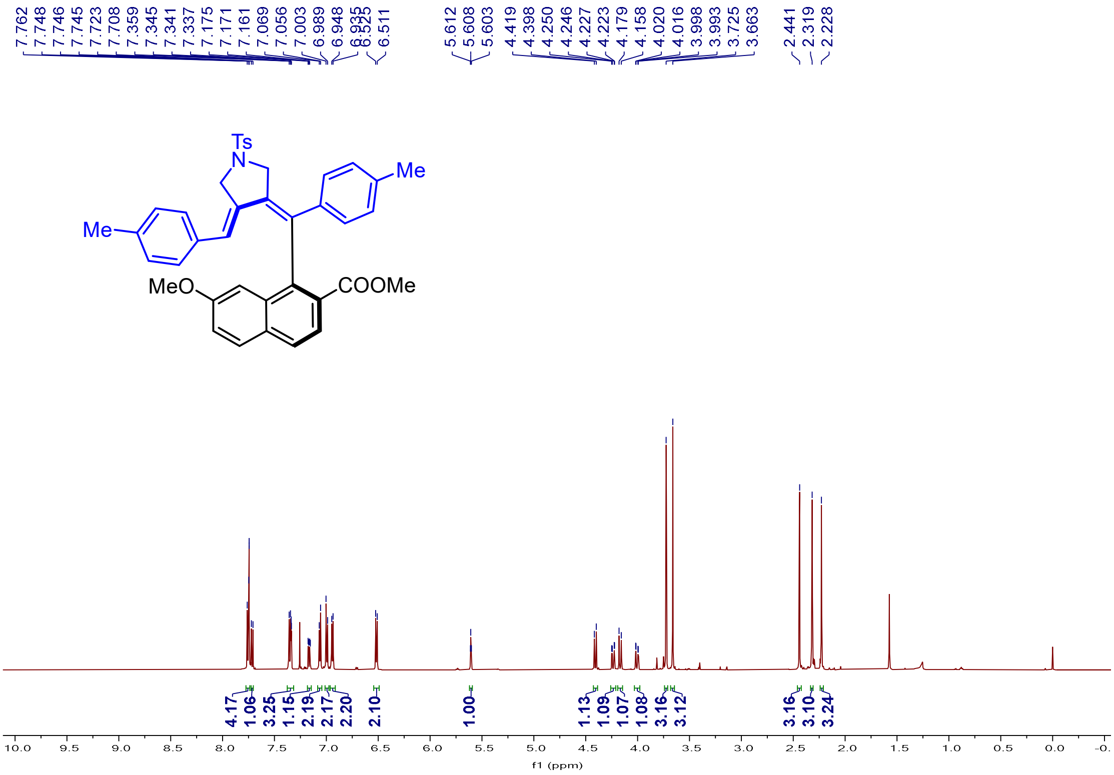

^{13}C NMR (150 MHz, Chloroform-d) spectrum of 27

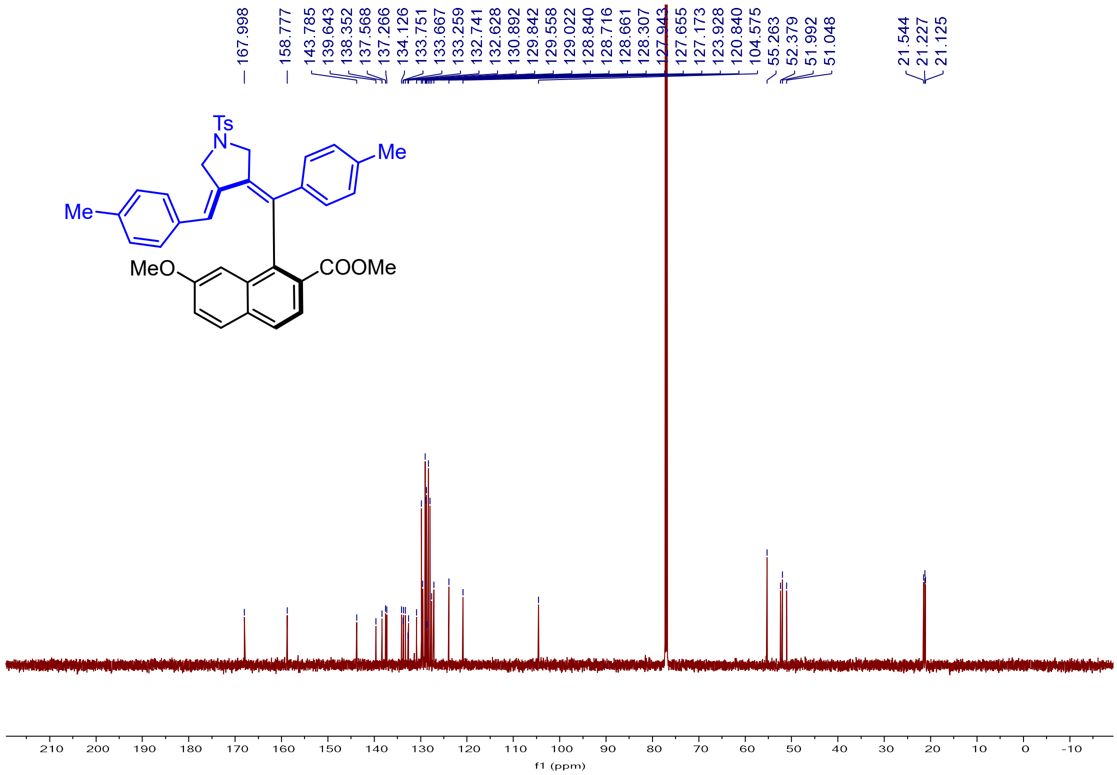

^{1}H NMR (600 MHz, Chloroform-d) spectrum of 28

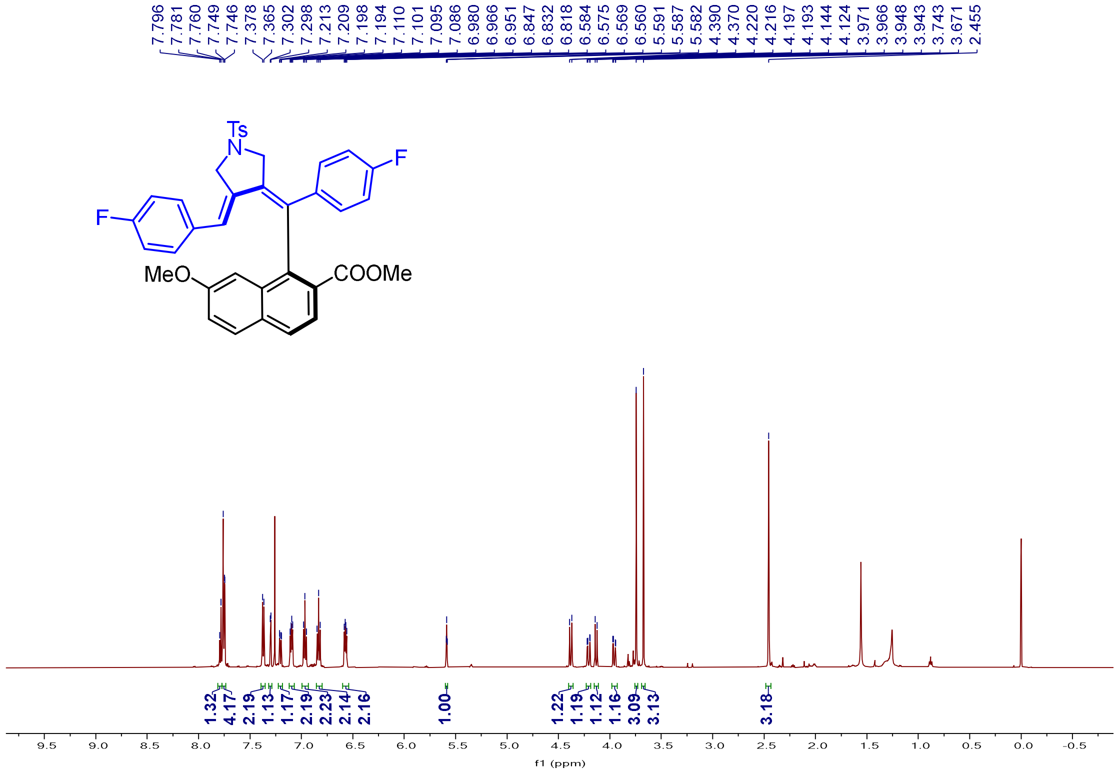

^{13}C NMR (150 MHz, Chloroform-d) spectrum of 28

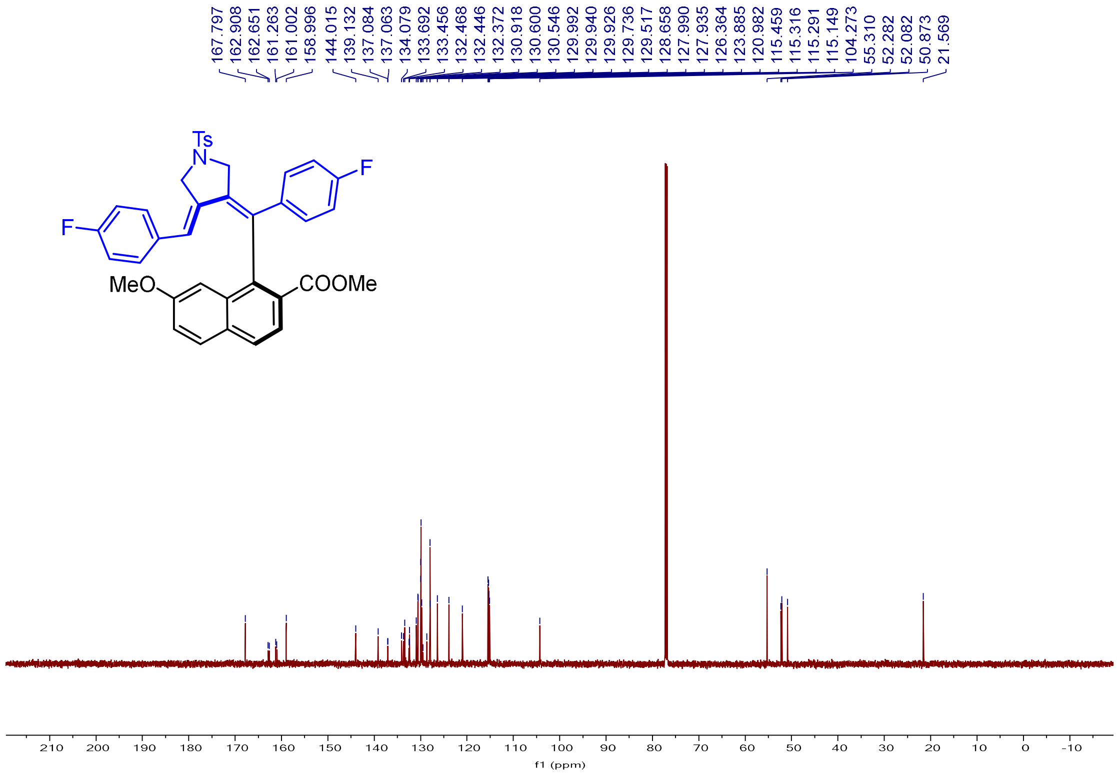

^{19}F NMR (376 MHz, Chloroform-d) spectrum of 28

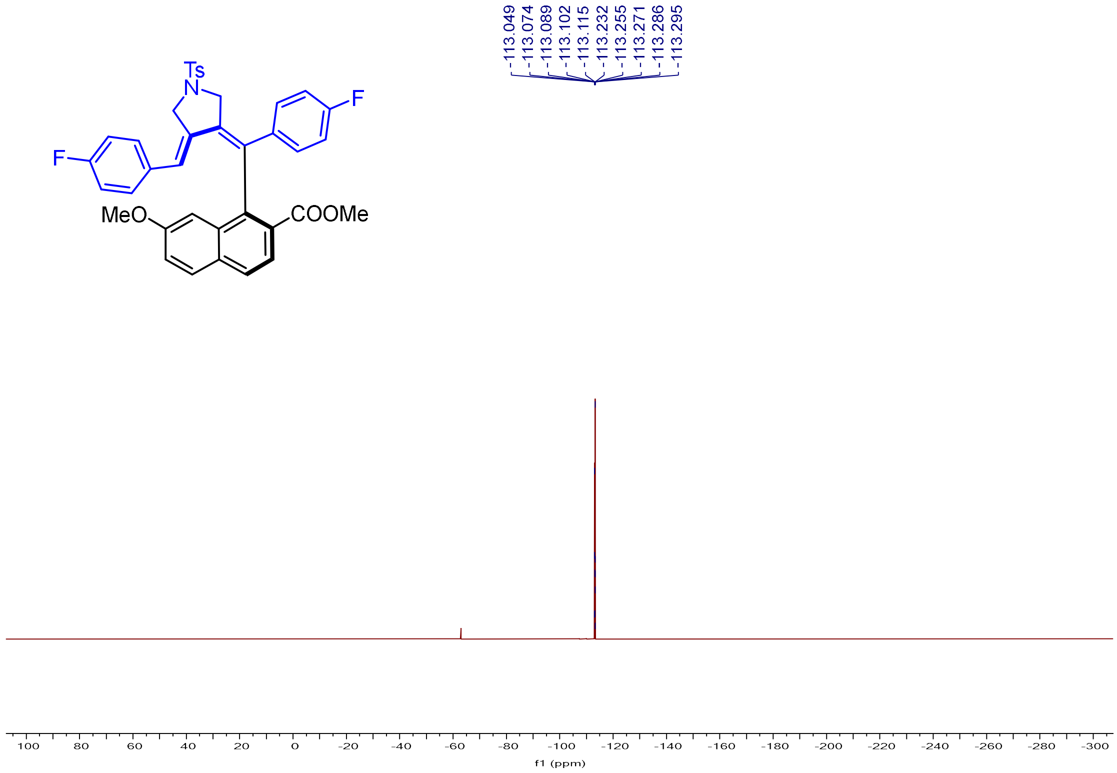

^{1}H NMR (600 MHz, Chloroform-d) spectrum of 29

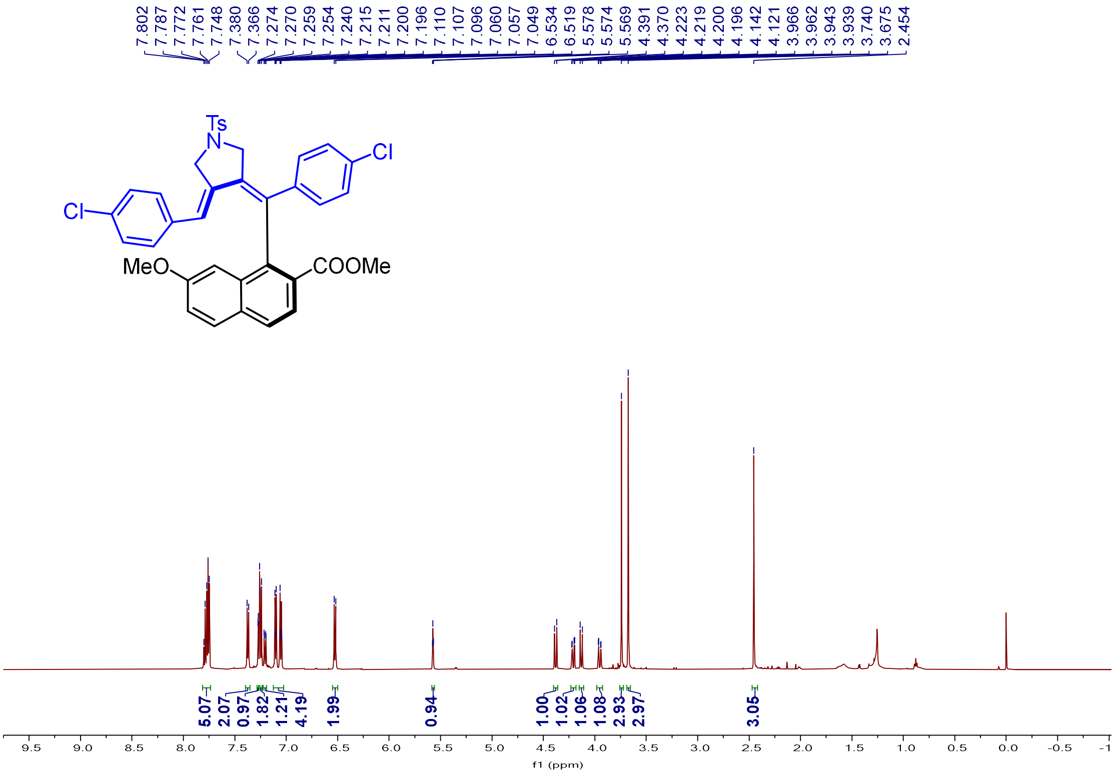

^{13}C NMR (150 MHz, Chloroform-d) spectrum of 29

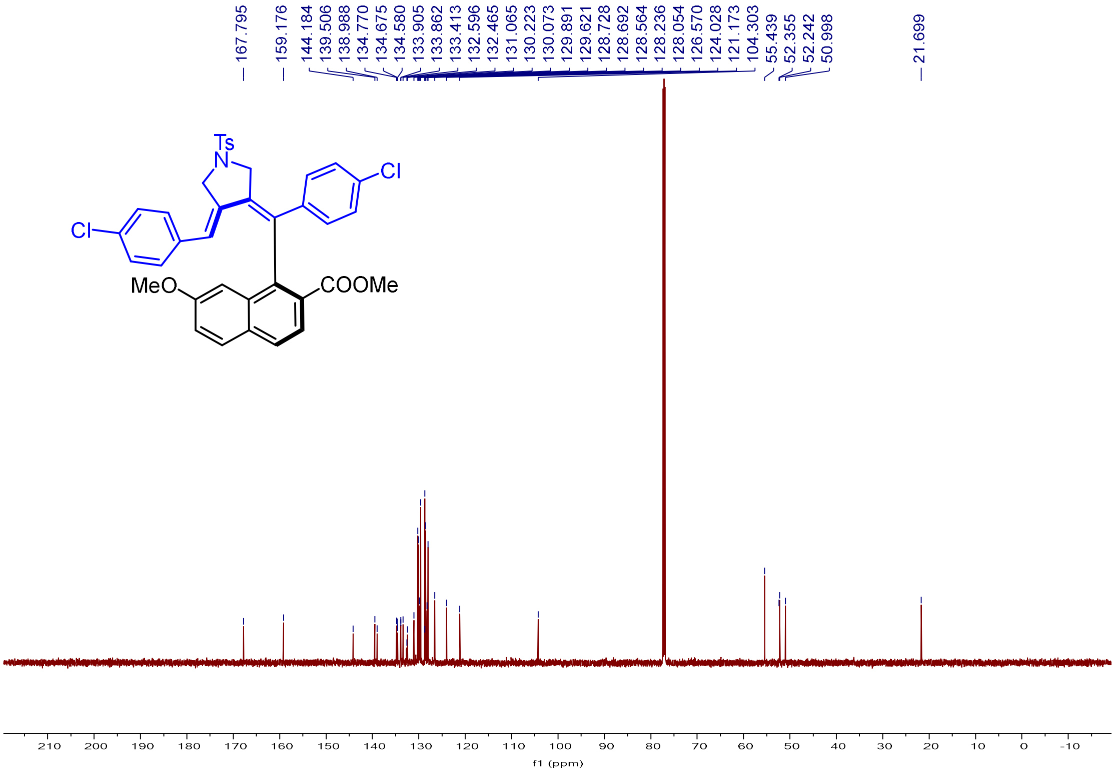

^{1}H NMR (600 MHz, Chloroform-d) spectrum of 30

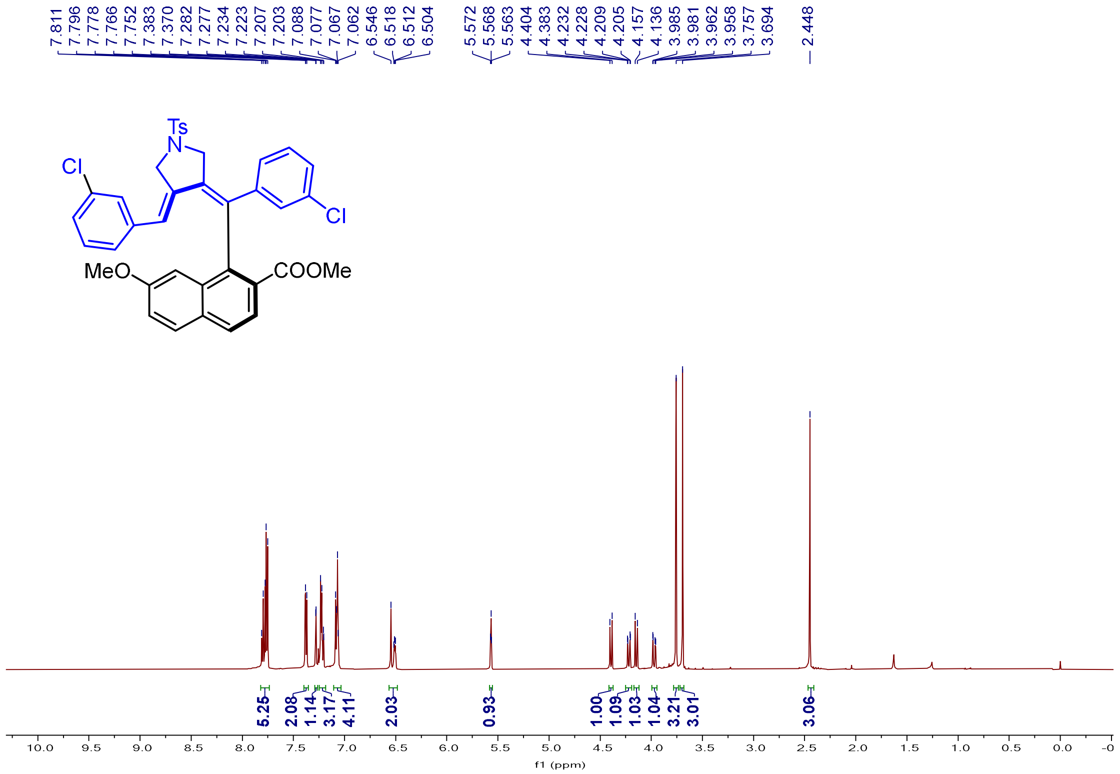

^{13}C NMR (150 MHz, Chloroform-d) spectrum of 30

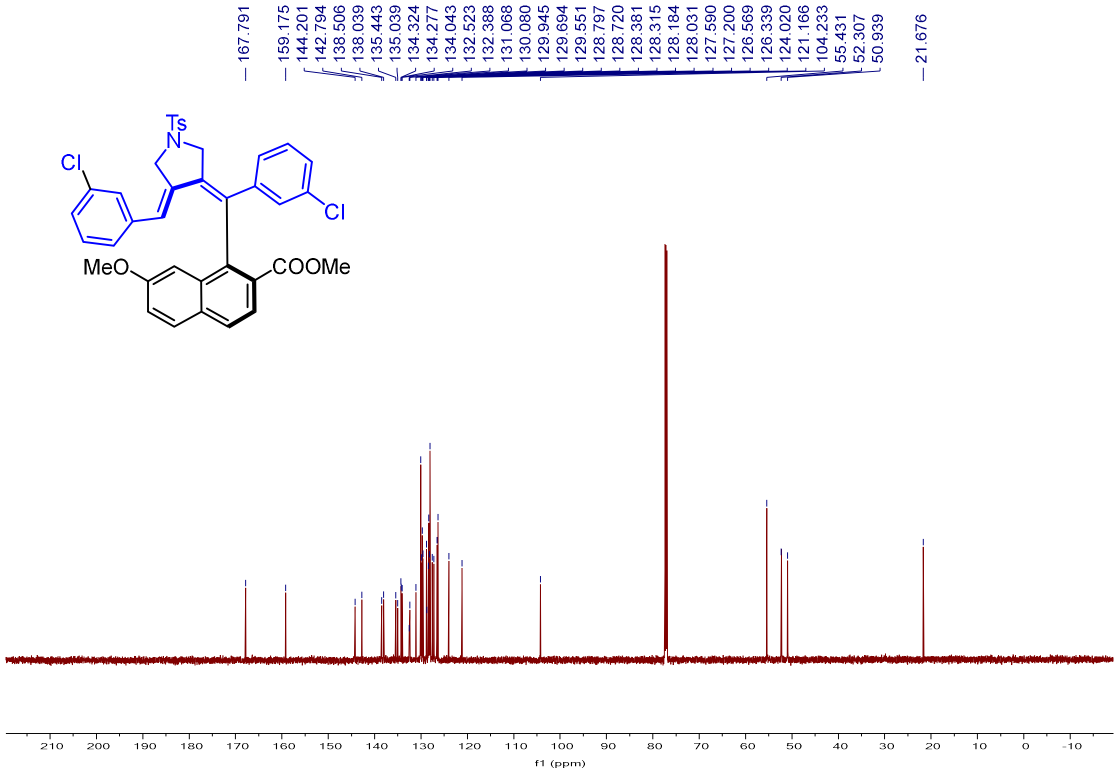

^{1}H NMR (600 MHz, Chloroform-d) spectrum of 31

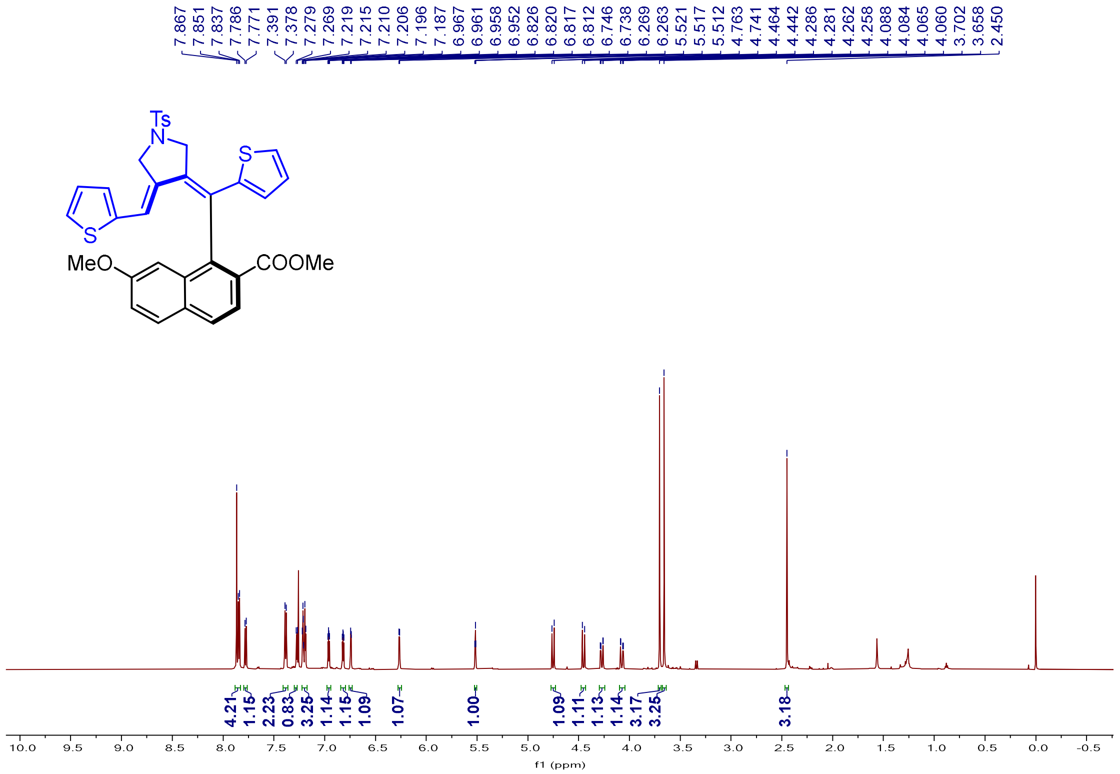

^{13}C NMR (150 MHz, Chloroform-d) spectrum of 31

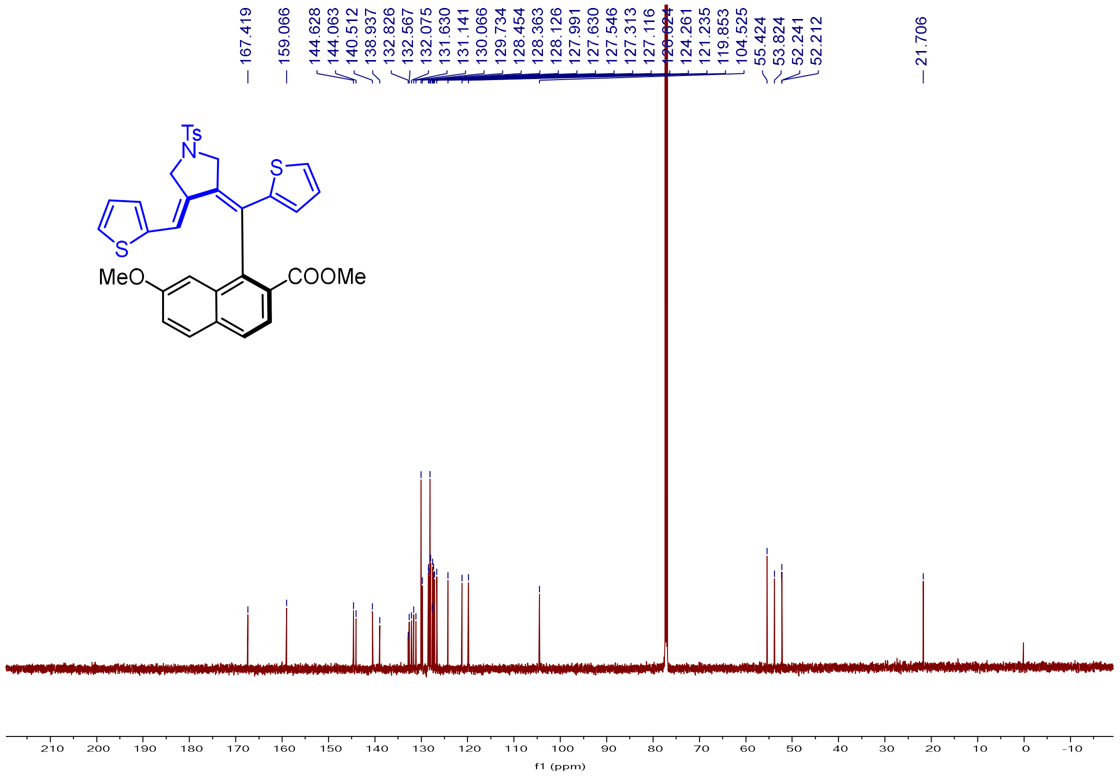

^{1}H NMR (600 MHz, Chloroform-d) spectrum of 32

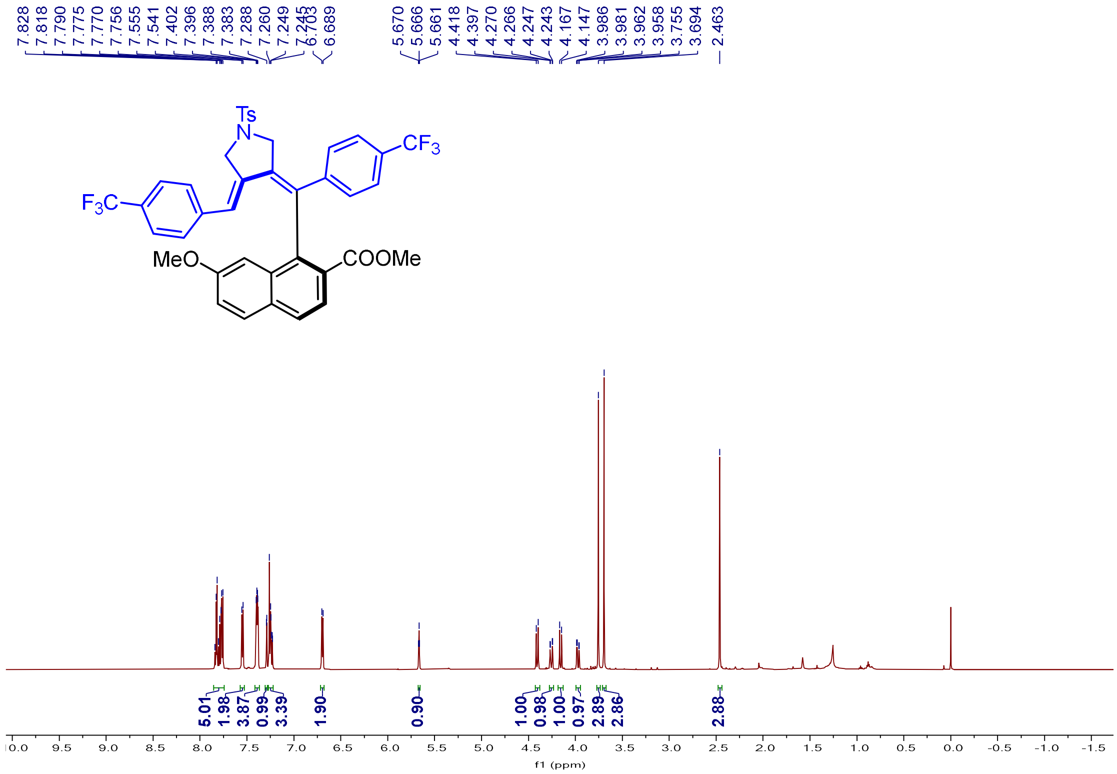

^{13}C NMR (150 MHz, Chloroform-d) spectrum of 32

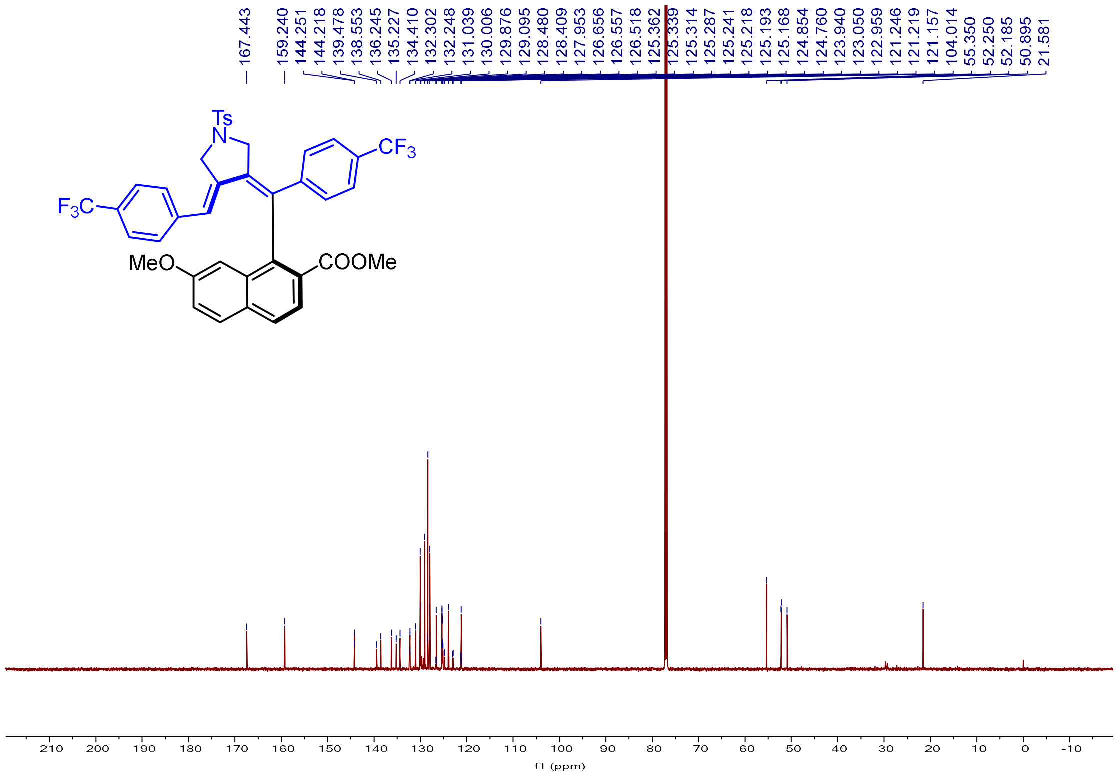

^{19}F NMR (376 MHz, Chloroform-d) spectrum of 32

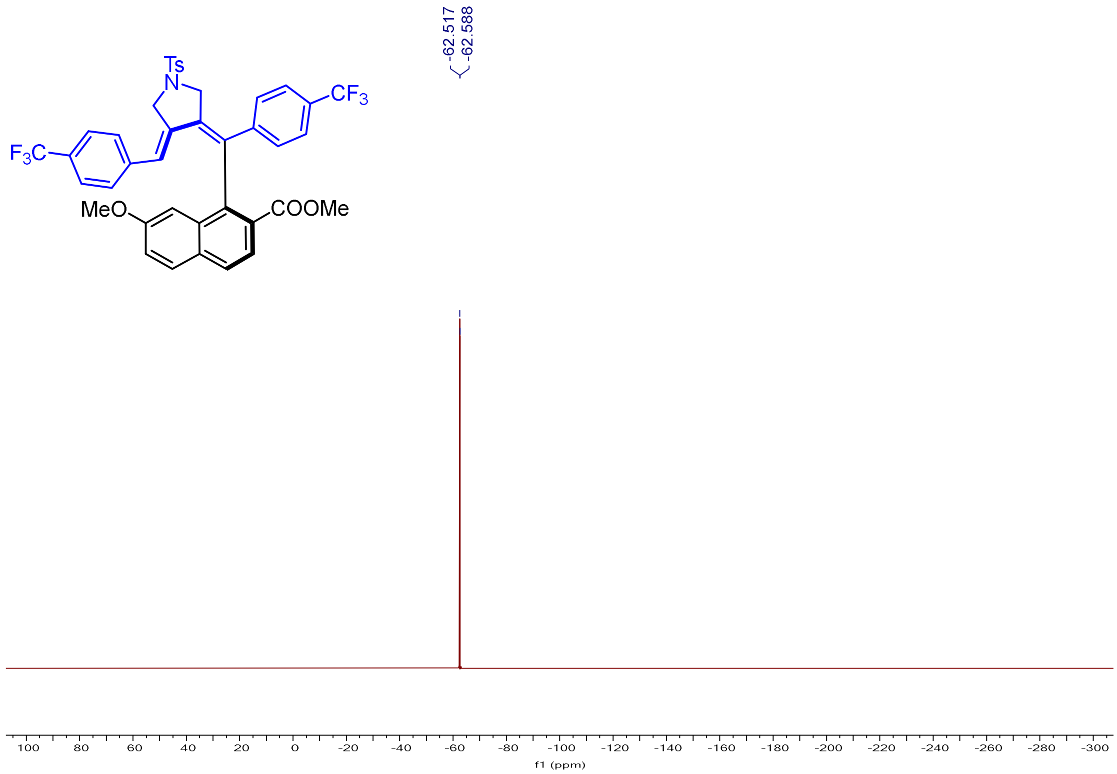

^{1}H NMR (600 MHz, Chloroform-d) spectrum of 33

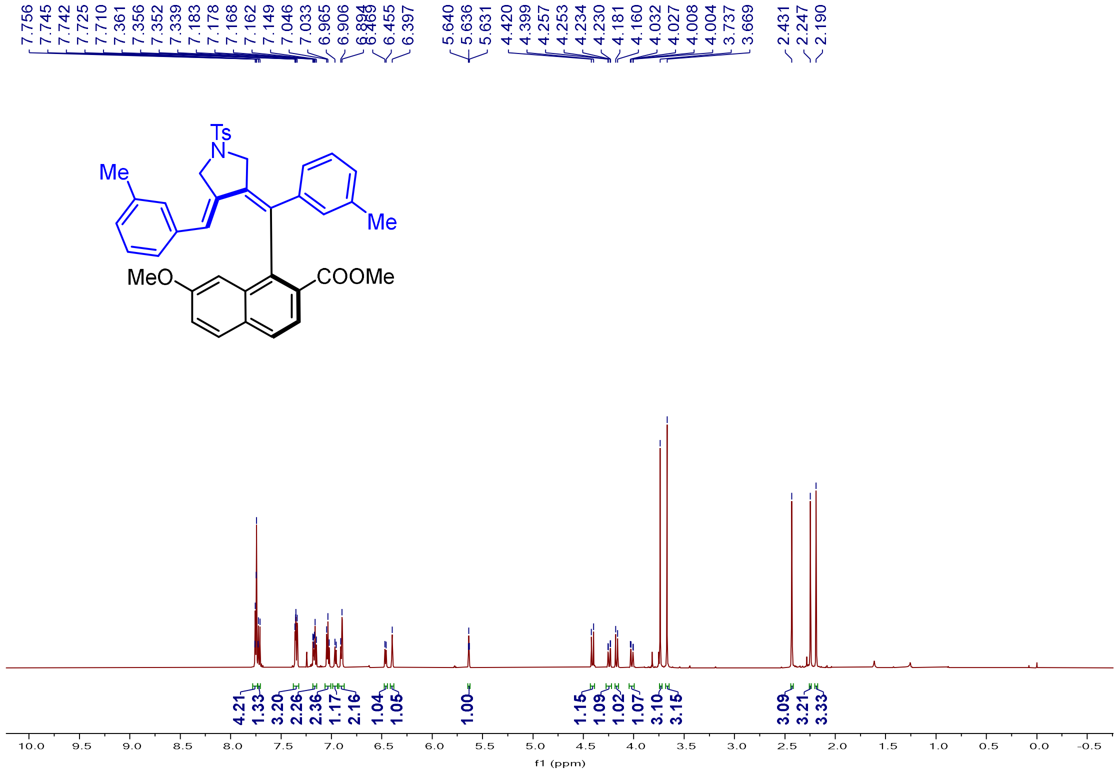

^{13}C NMR (150 MHz, Chloroform-d) spectrum of 33

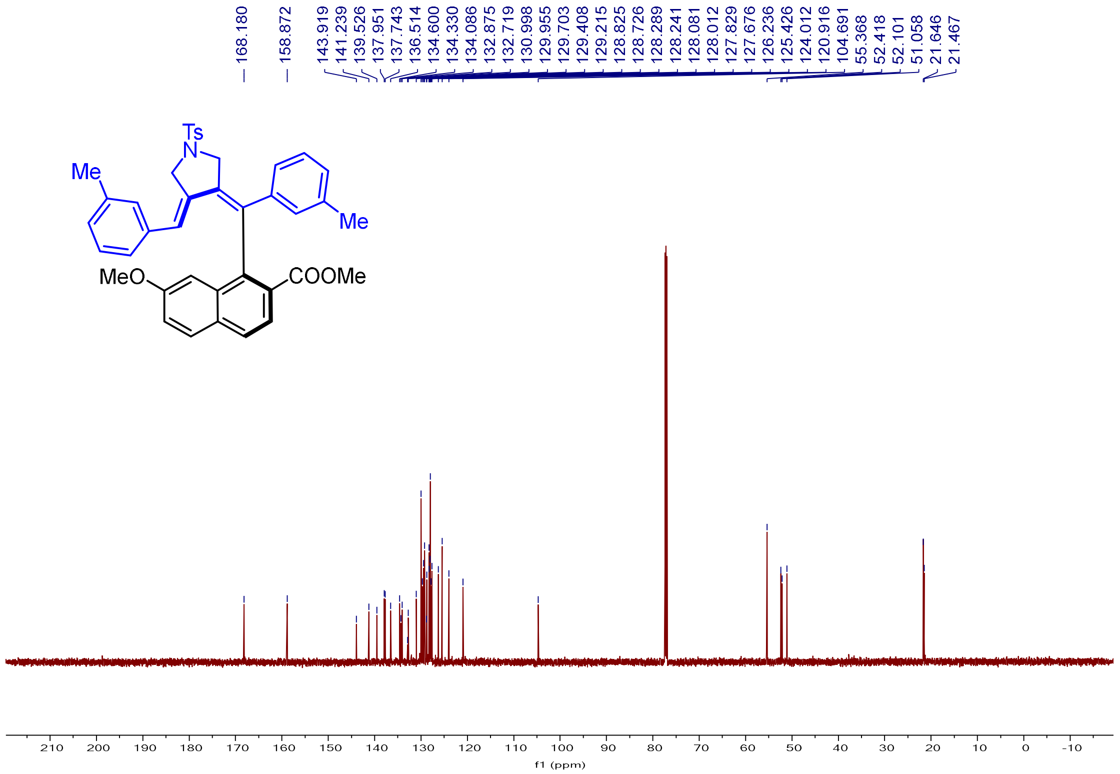

^{1}H NMR (600 MHz, Chloroform-d) spectrum of 34

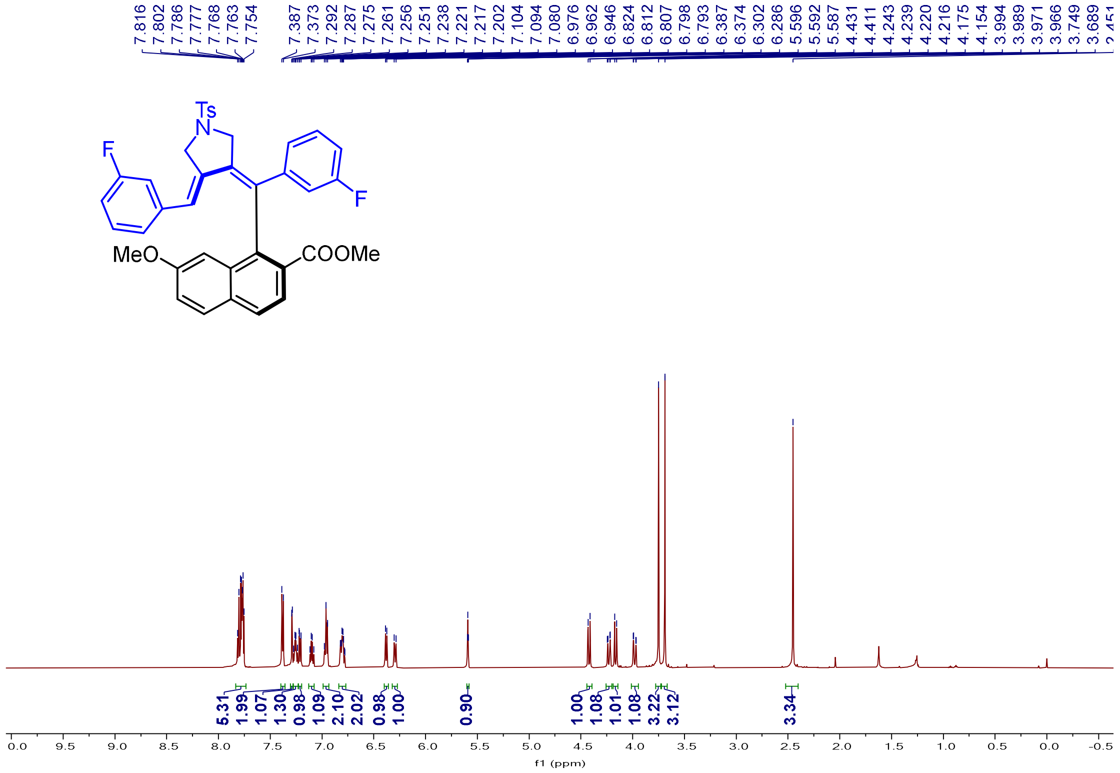

^{13}C NMR (150 MHz, Chloroform-d) spectrum of 34

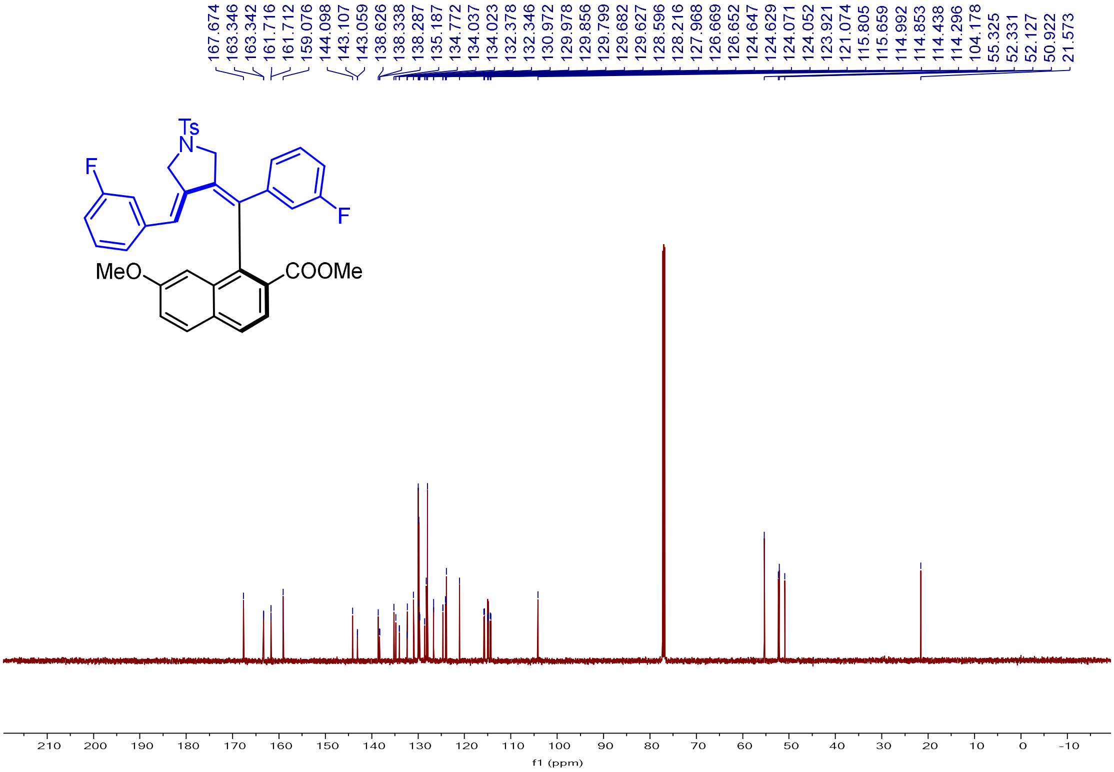

^{19}F NMR (376 MHz, Chloroform-d) spectrum of 34

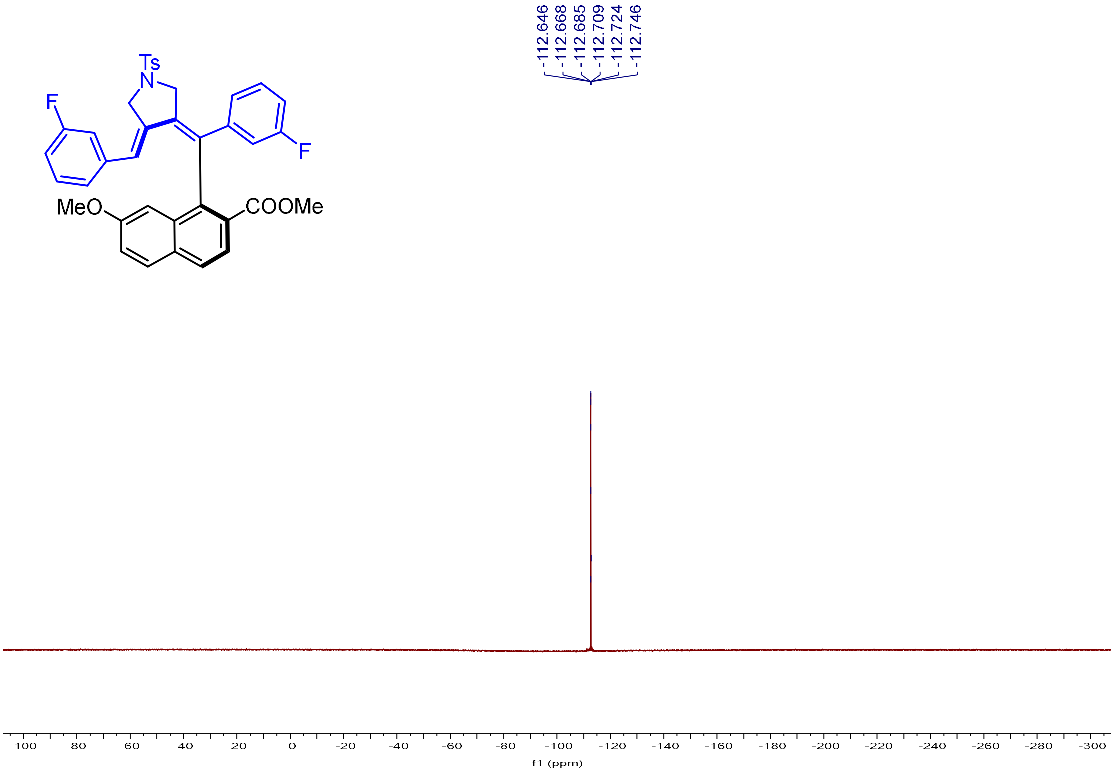

^{1}H NMR (600 MHz, Chloroform-d) spectrum of 35

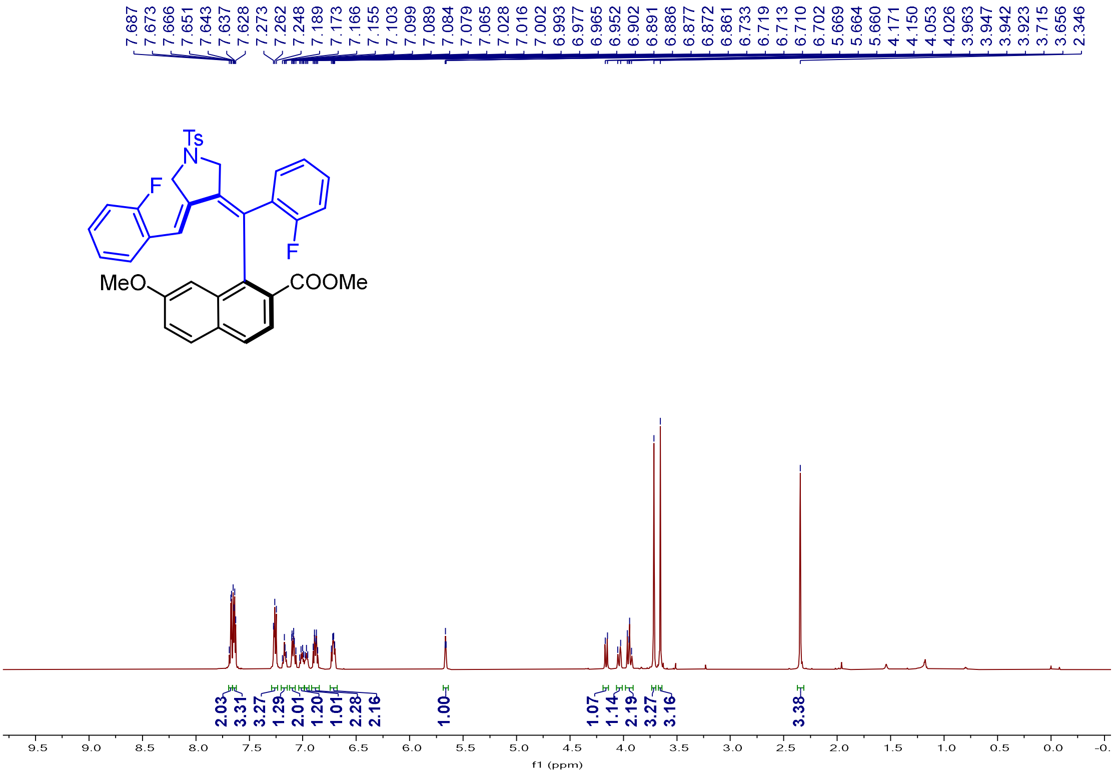

^{13}C NMR (150 MHz, Chloroform-d) spectrum of 35

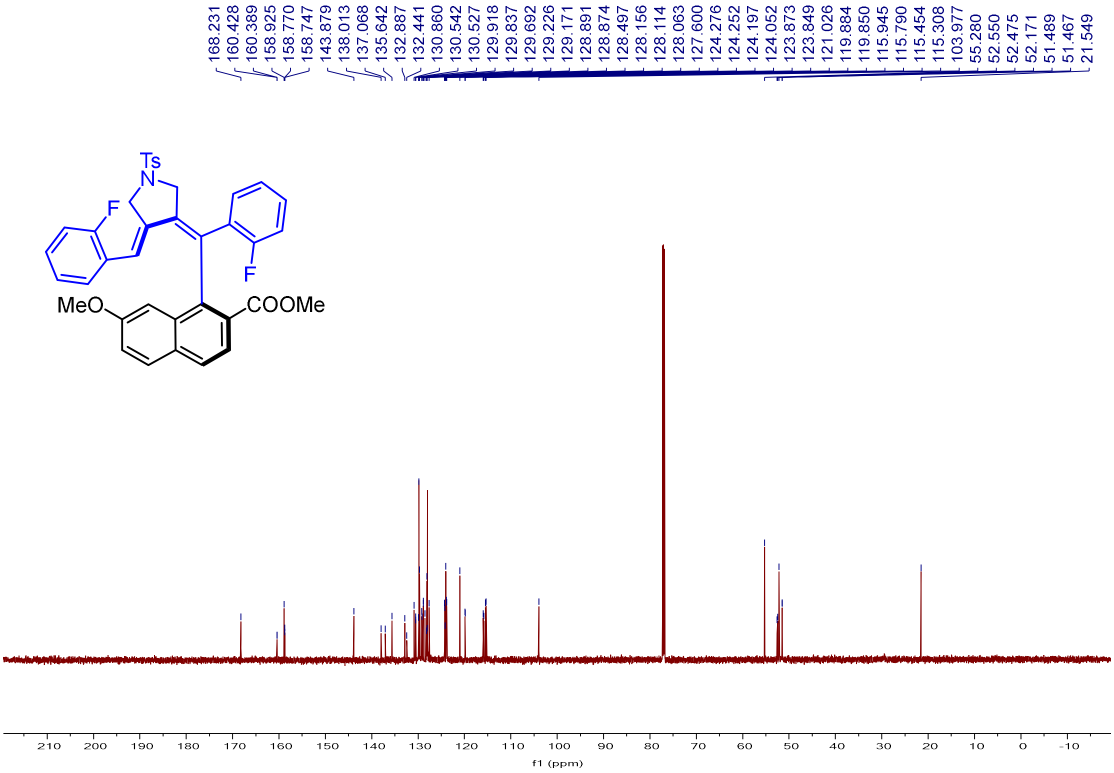

^{19}F NMR (376 MHz, Chloroform-d) spectrum of 35

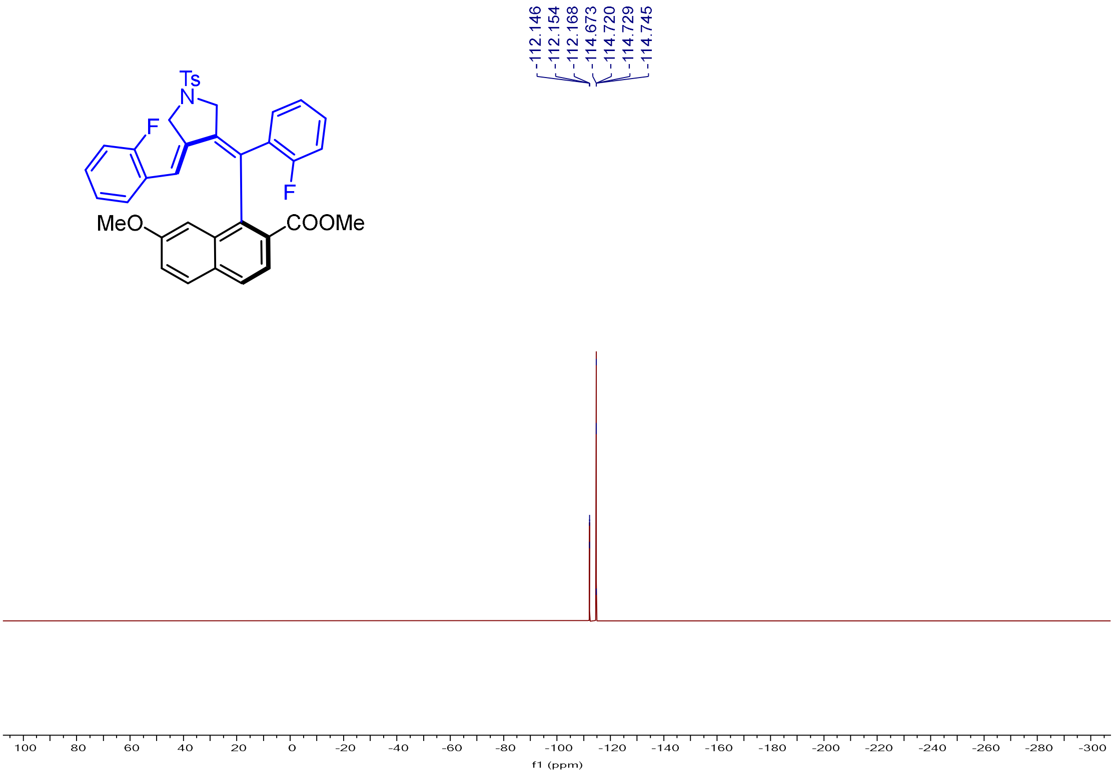

^{1}H NMR (600 MHz, Chloroform-d) spectrum of 36

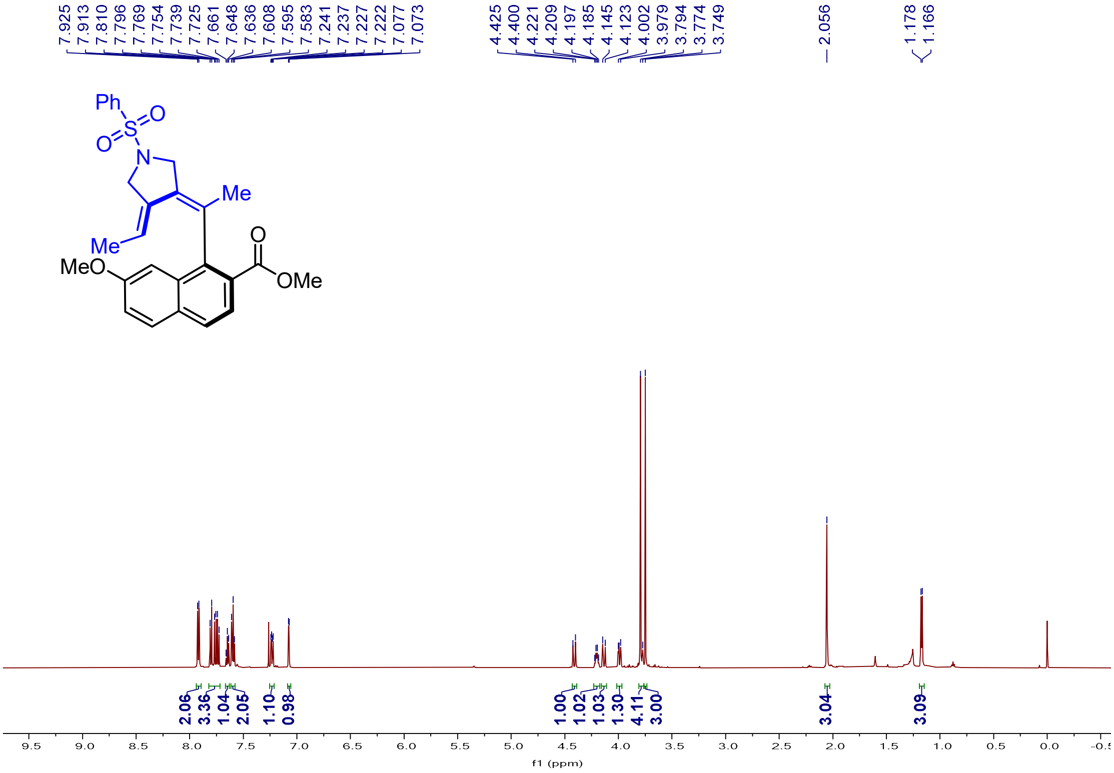

^{13}C NMR (150 MHz, Chloroform-d) spectrum of 36

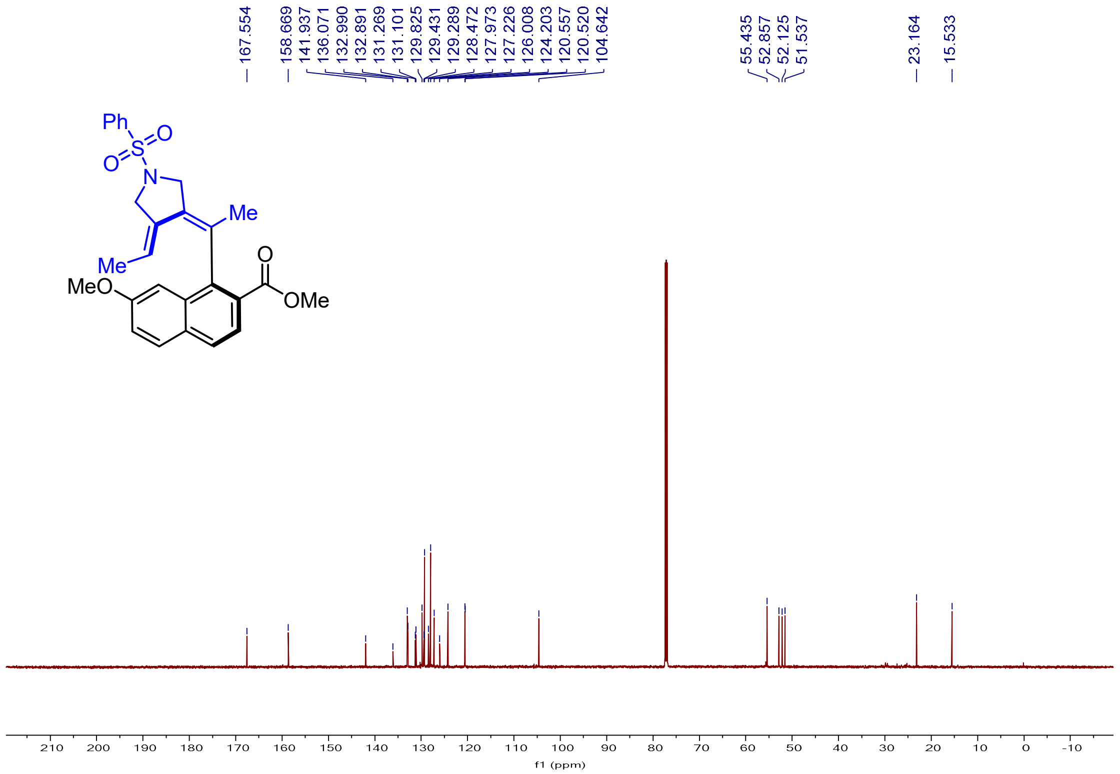

^{1}H NMR (600 MHz, Chloroform-d) spectrum of 37

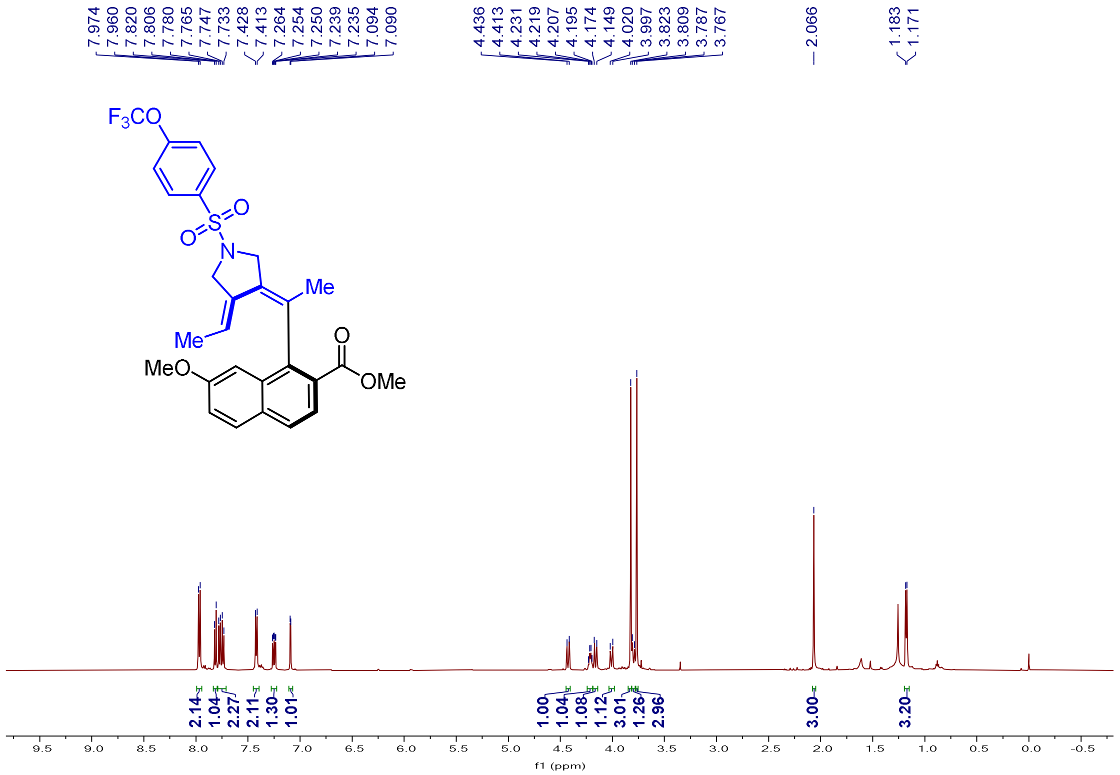

^{13}C NMR (150 MHz, Chloroform-d) spectrum of 37

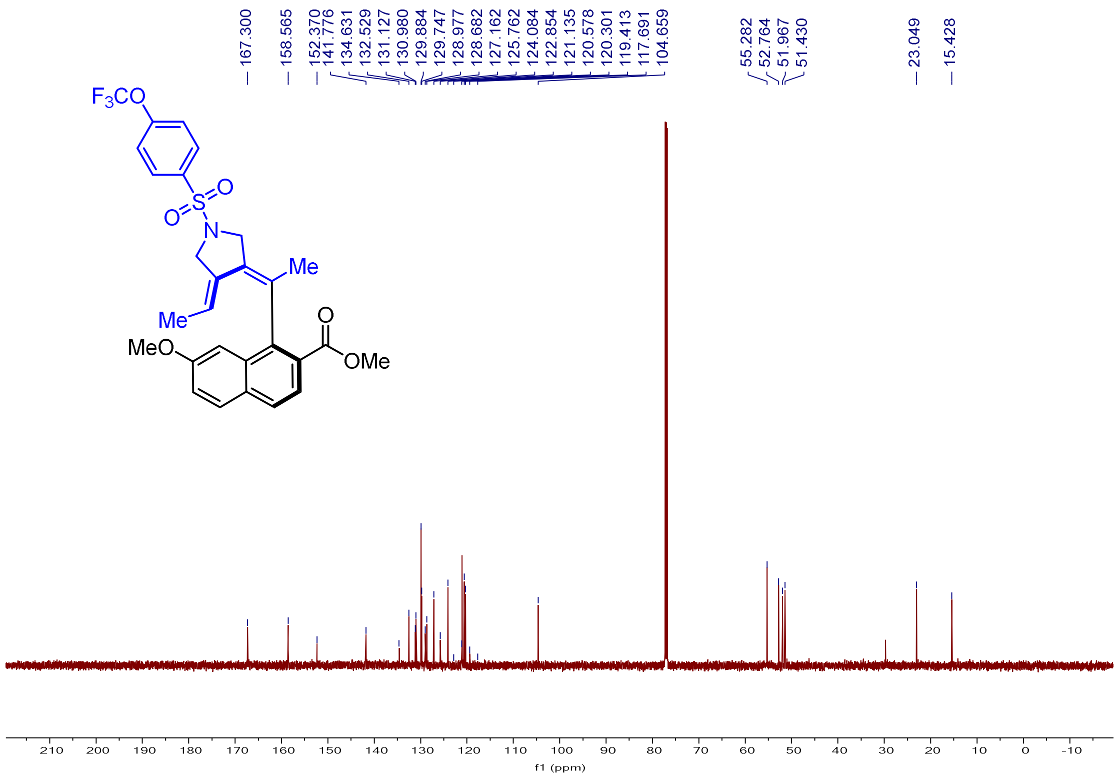

^{19}F NMR (376 MHz, Chloroform-d) spectrum of 37

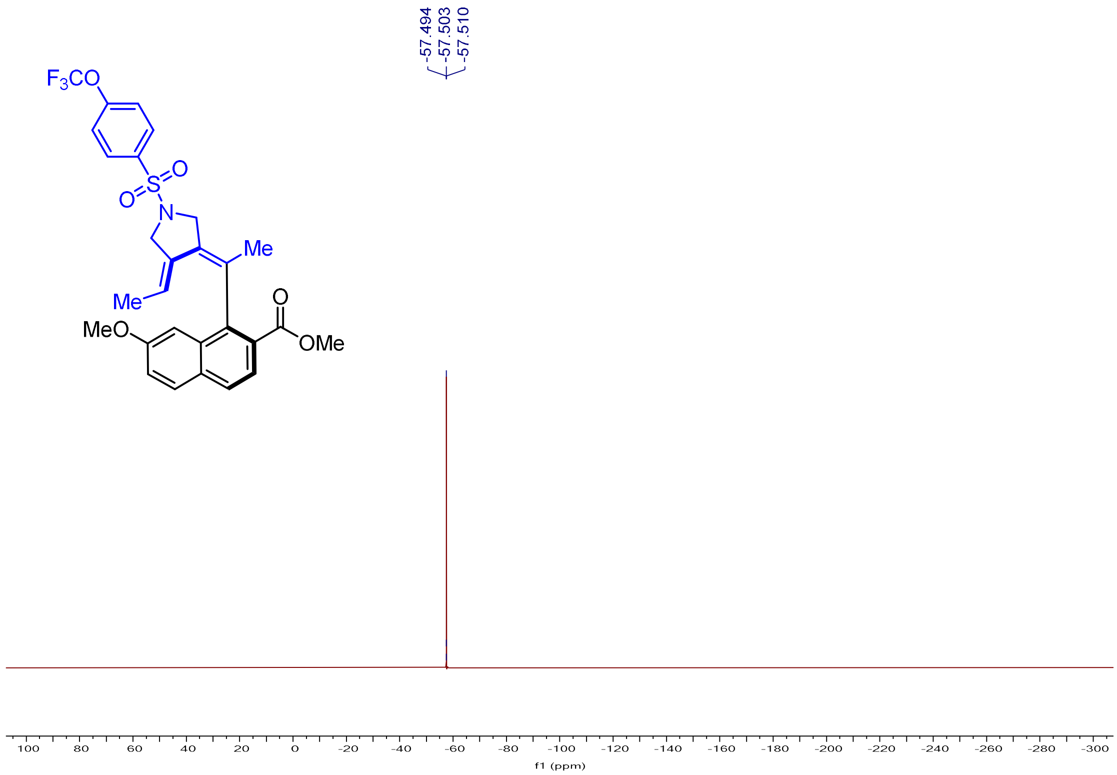

^{1}H NMR (600 MHz, Chloroform-d) spectrum of 38

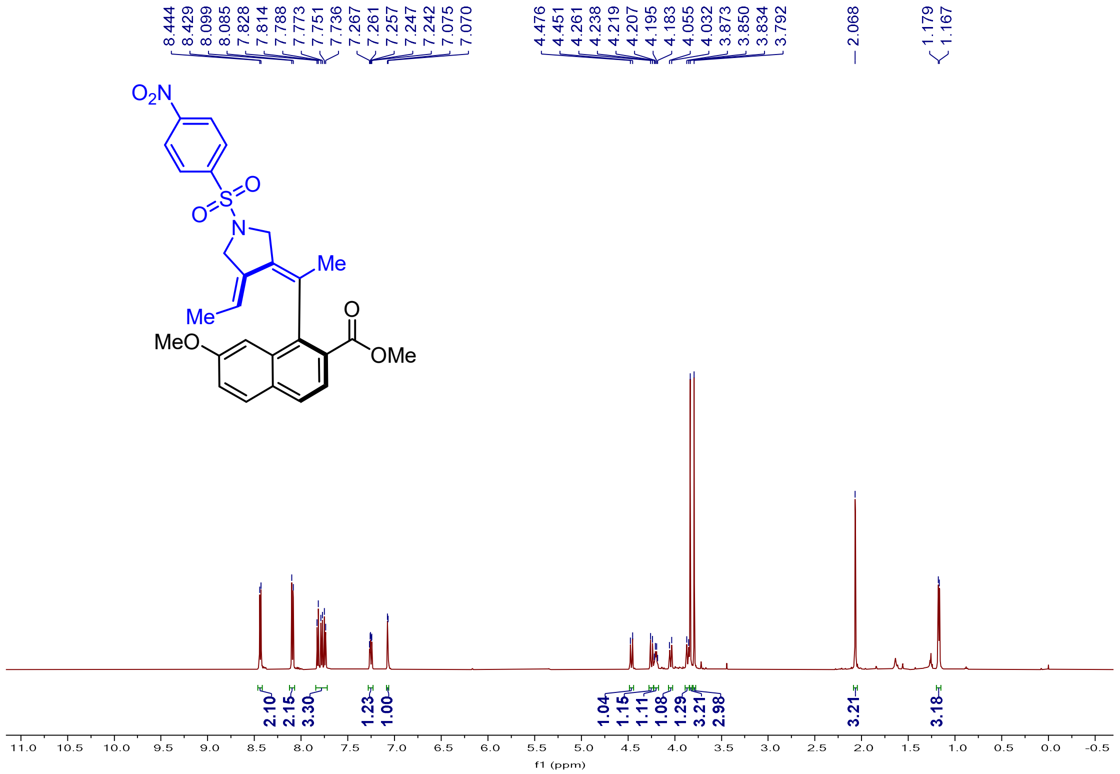

^{13}C NMR (150 MHz, Chloroform-d) spectrum of 38

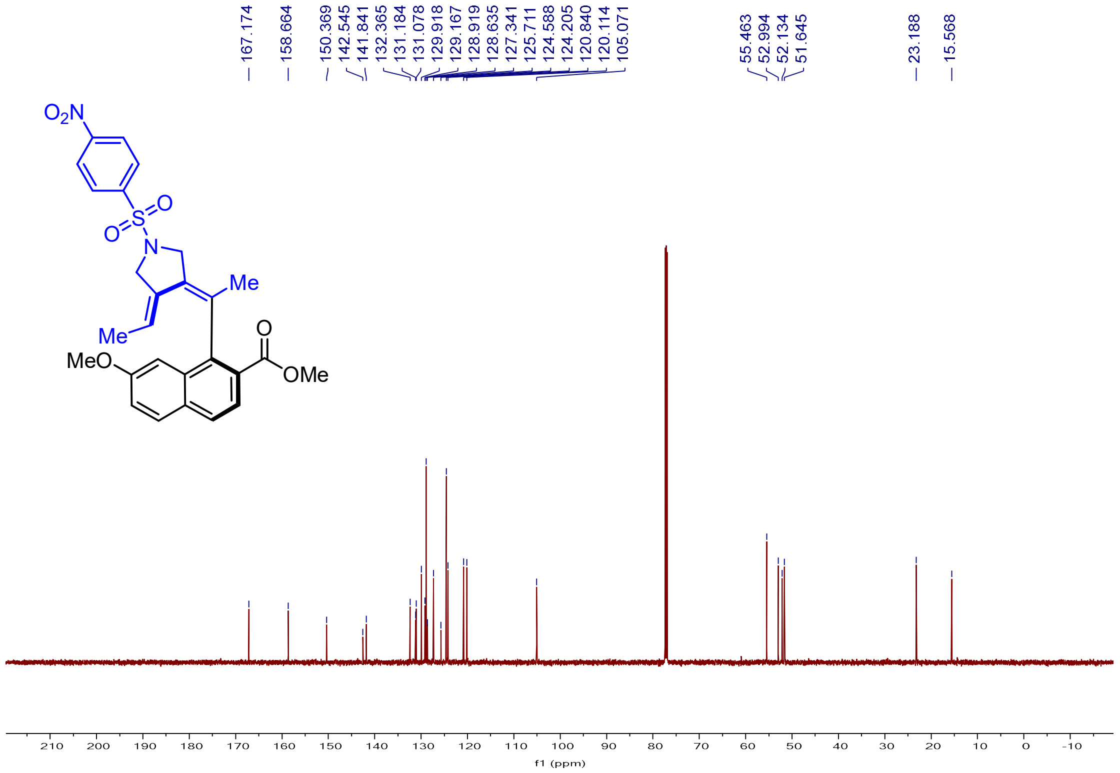

1H NMR (600 MHz, Chloroform-d) spectrum of 39

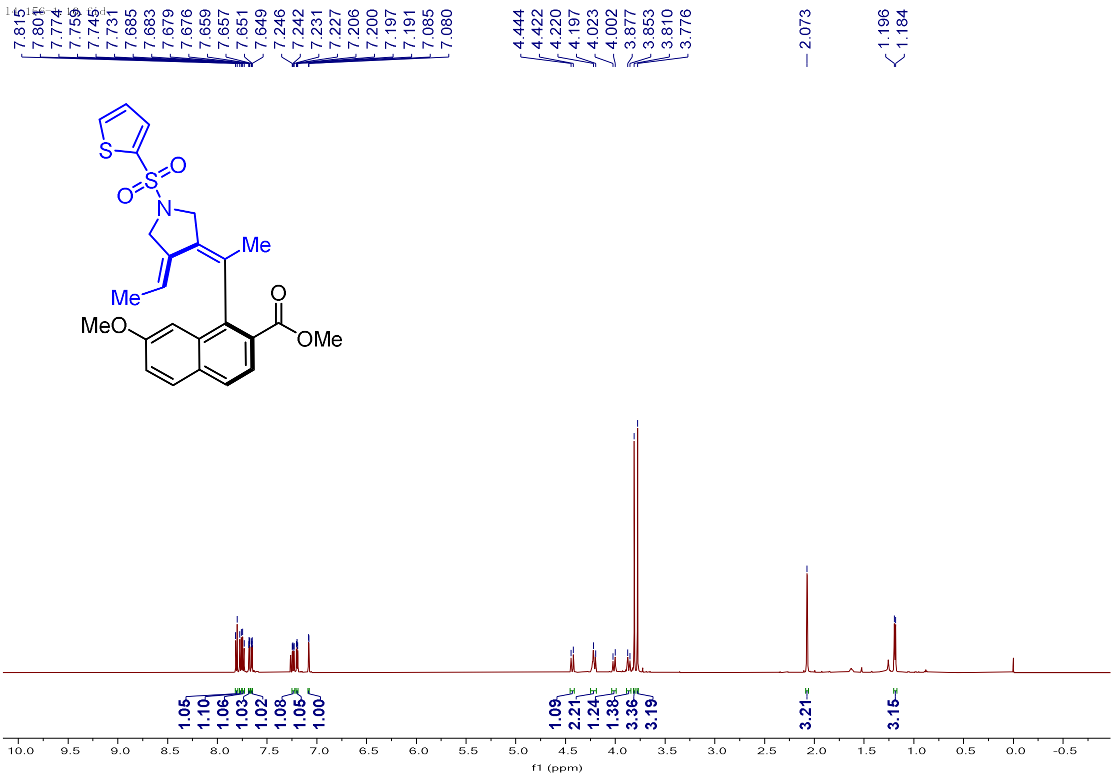

13C NMR (150 MHz, Chloroform-d) spectrum of 39

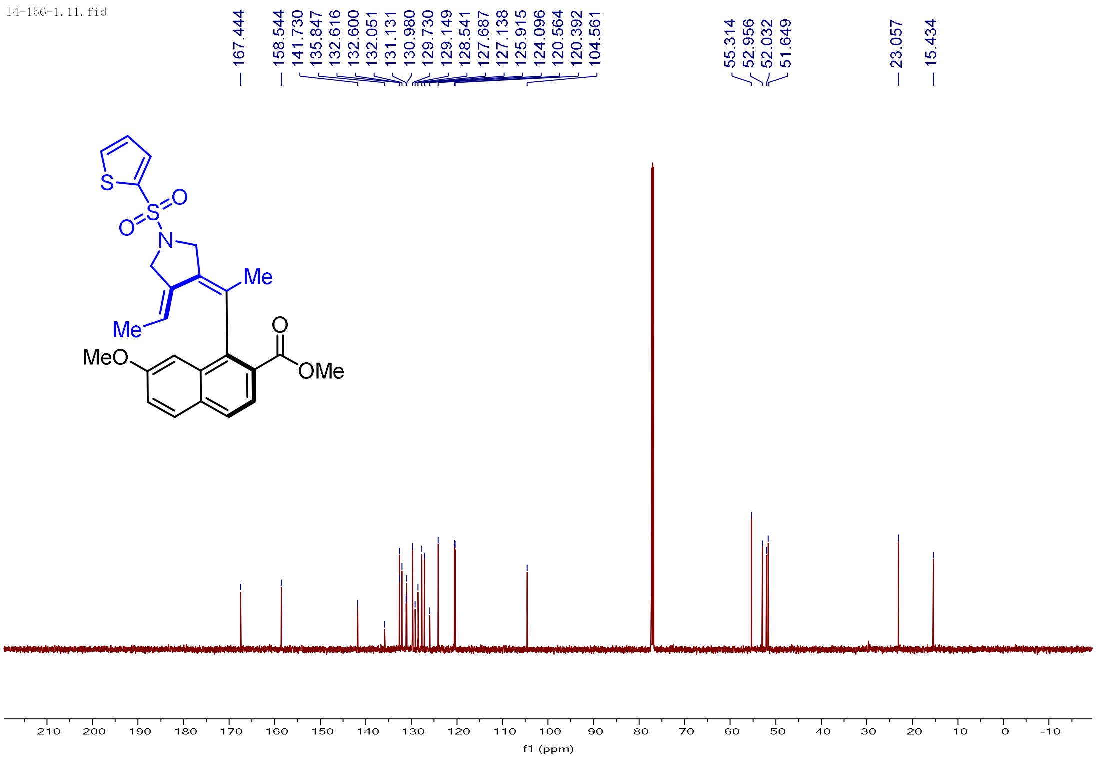

^{1}H NMR (600 MHz, Chloroform-d) spectrum of 40

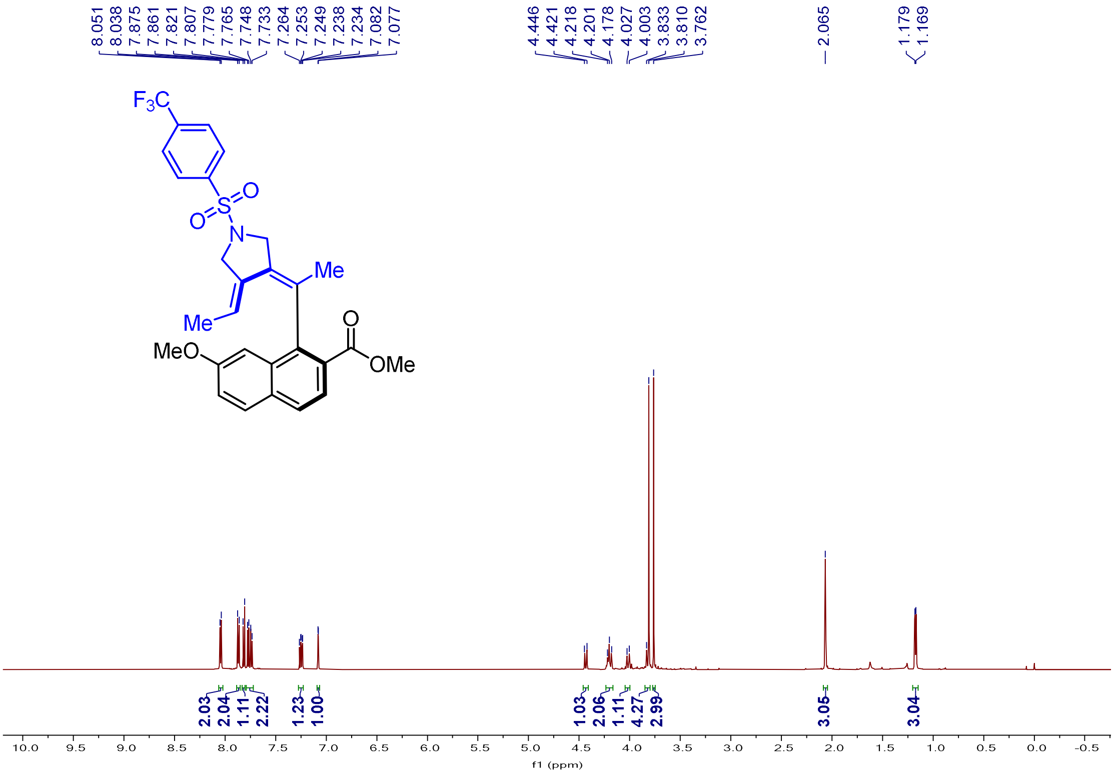

^{13}C NMR (150 MHz, Chloroform-d) spectrum of 40

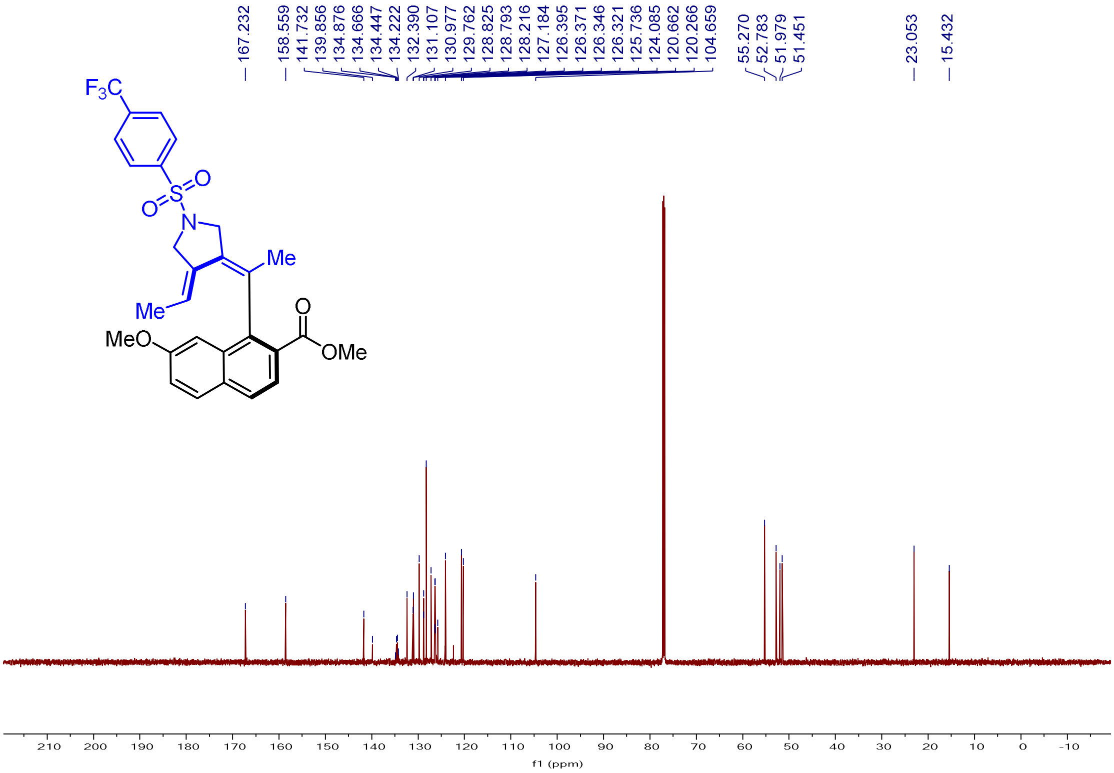

^{19}F NMR (376 MHz, Chloroform-d) spectrum of 40

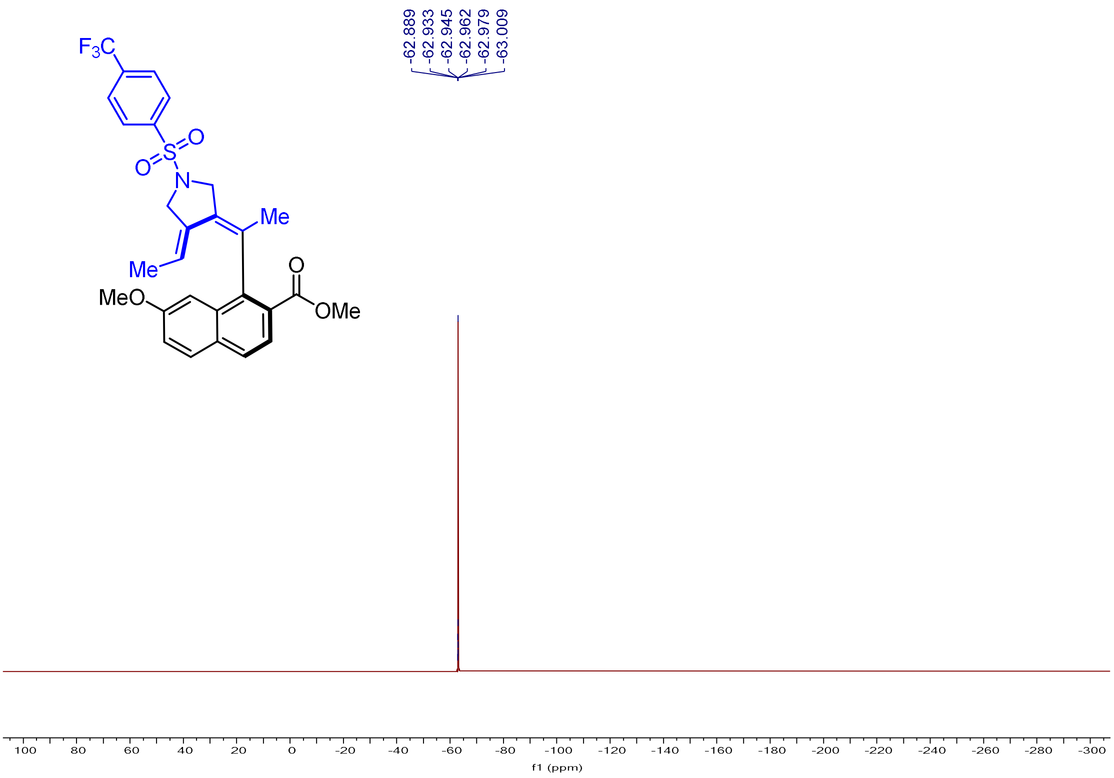

^{1}H NMR (600 MHz, Chloroform-d) spectrum of 41

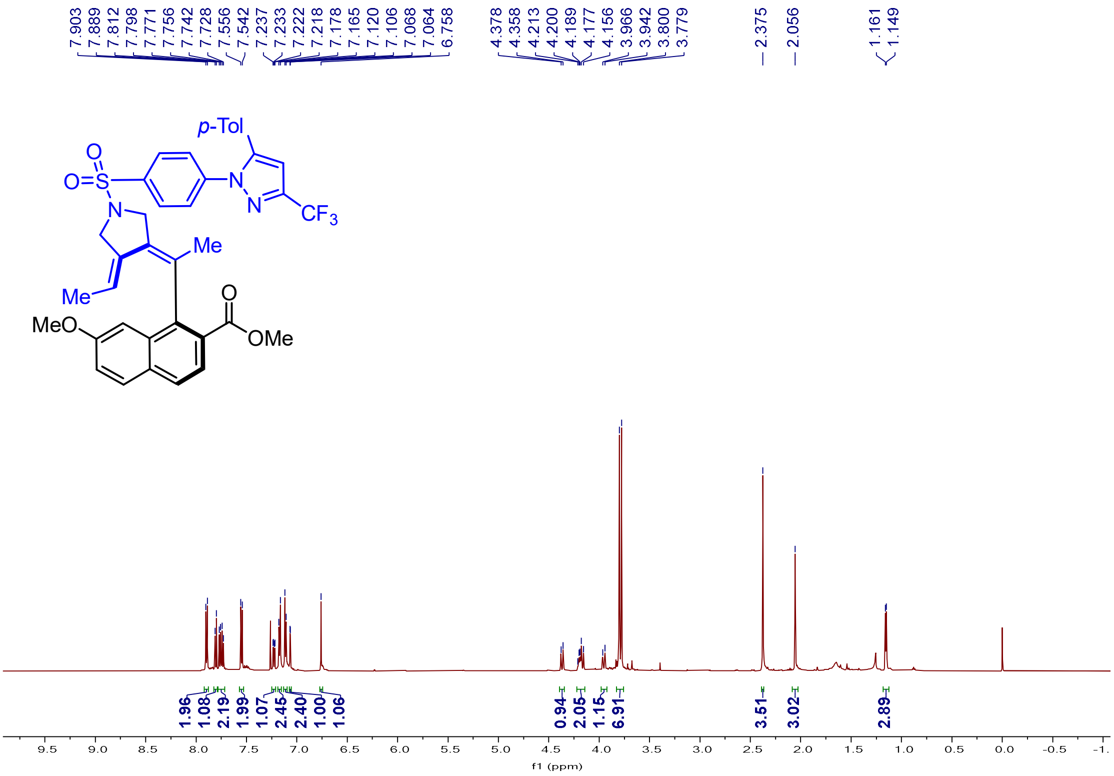

^{13}C NMR (150 MHz, Chloroform-d) spectrum of 41

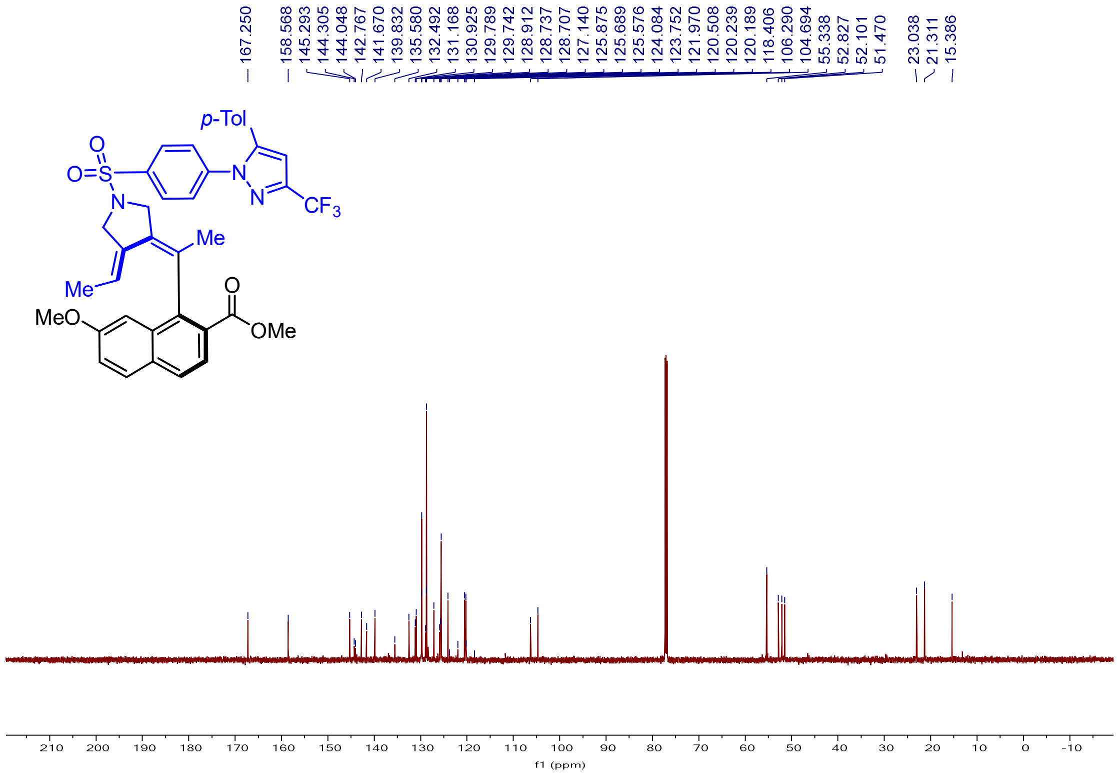

^{19}F NMR (376 MHz, Chloroform-d) spectrum of 41

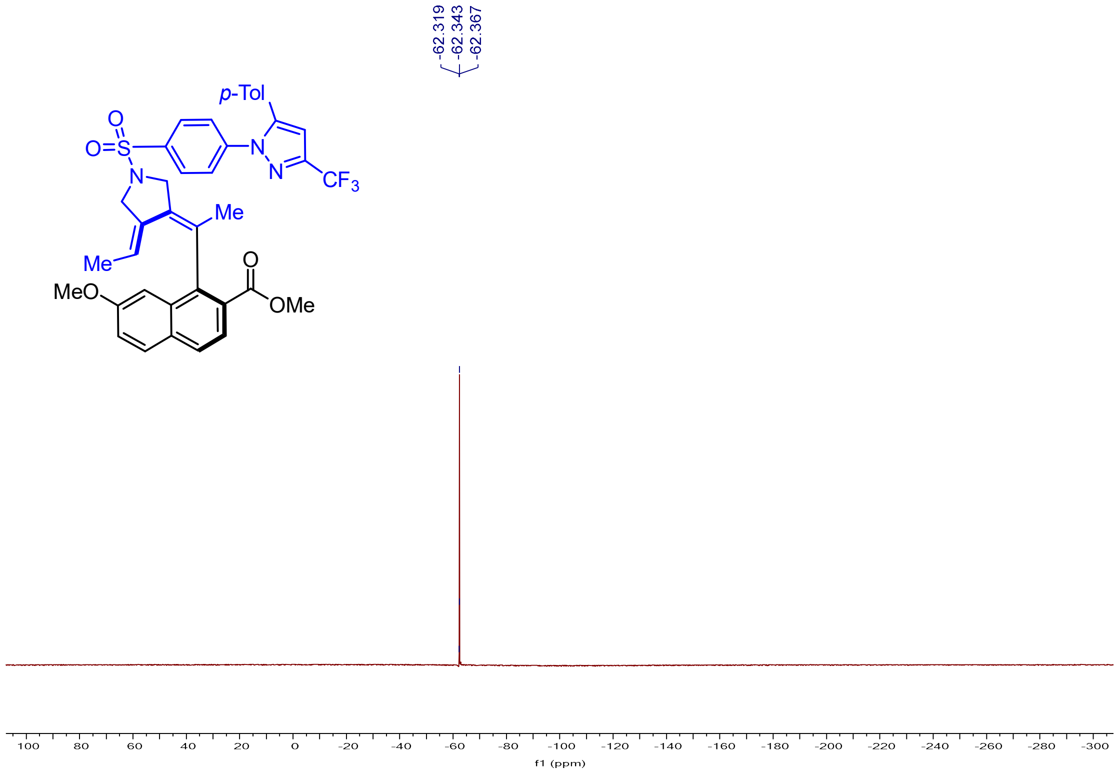

^{1}H NMR (600 MHz, Chloroform-d) spectrum of 42

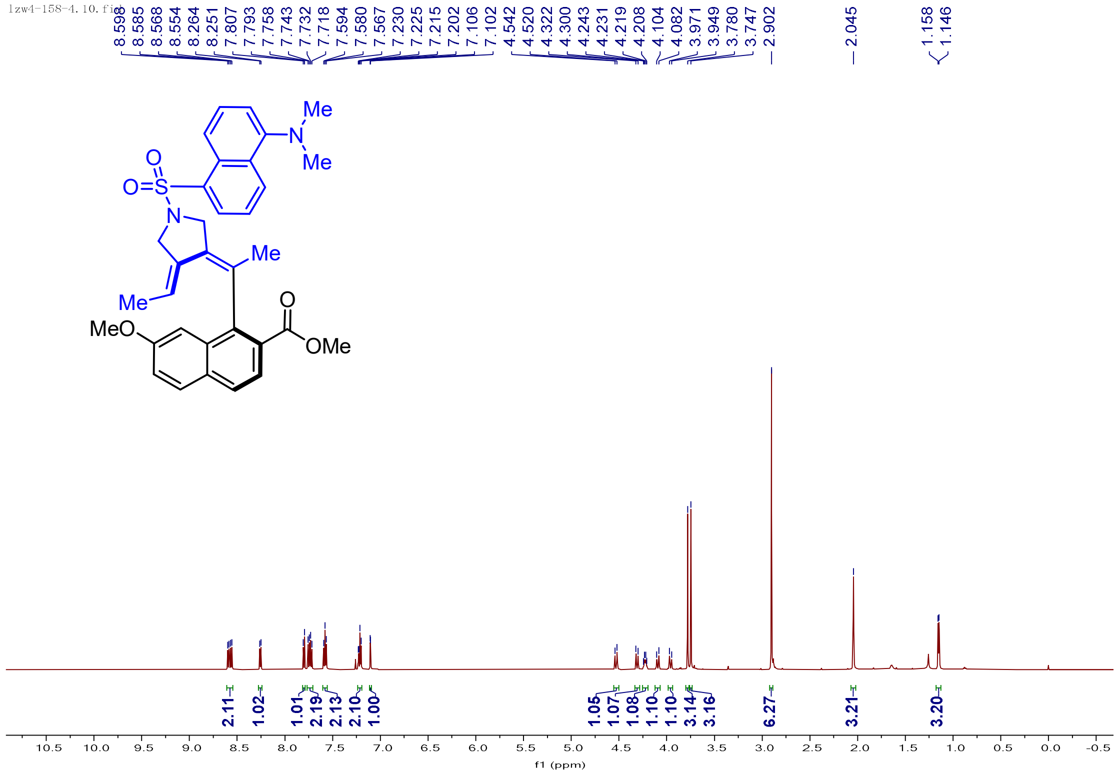

^{13}C NMR (150 MHz, Chloroform-d) spectrum of 42

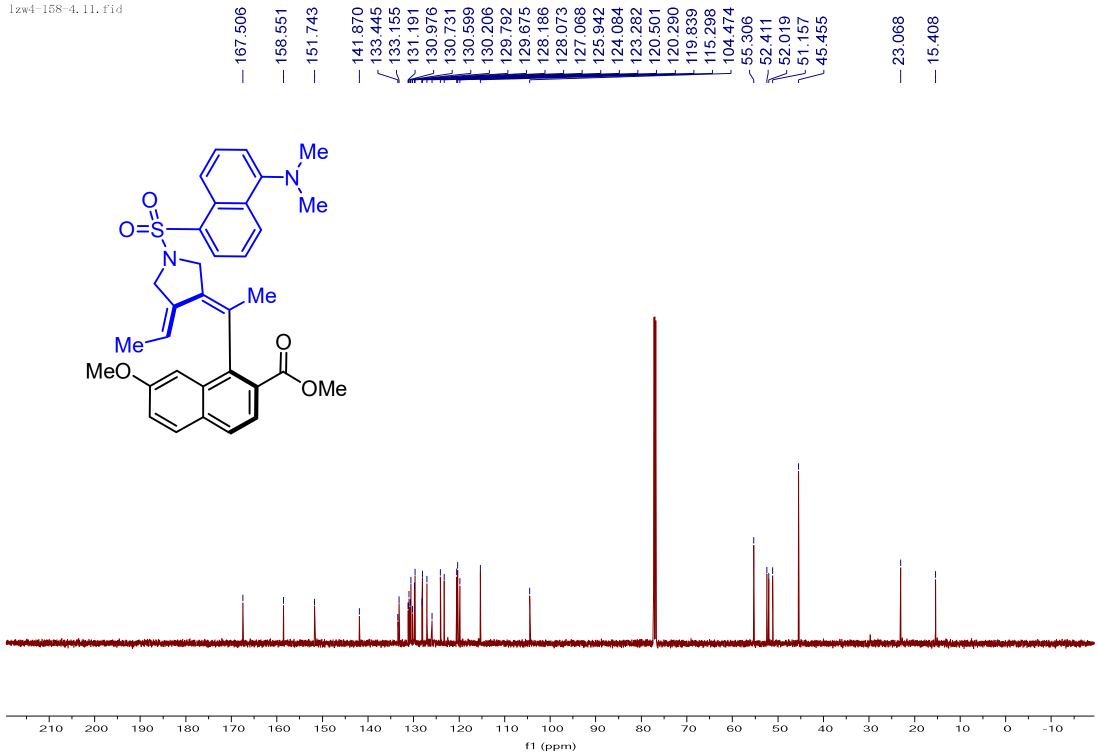

^{1}H NMR (600 MHz, DMSO-d_6) spectrum of 3'

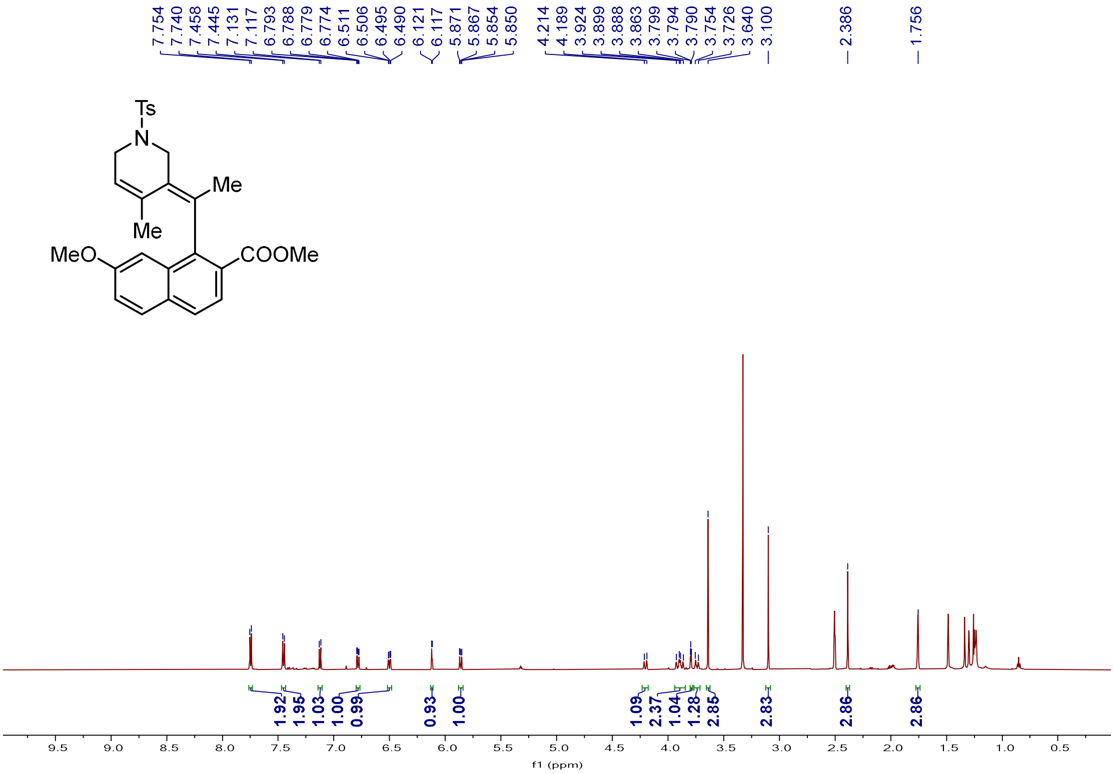

^{13}C NMR (150 MHz, Chloroform-d) spectrum of 3'

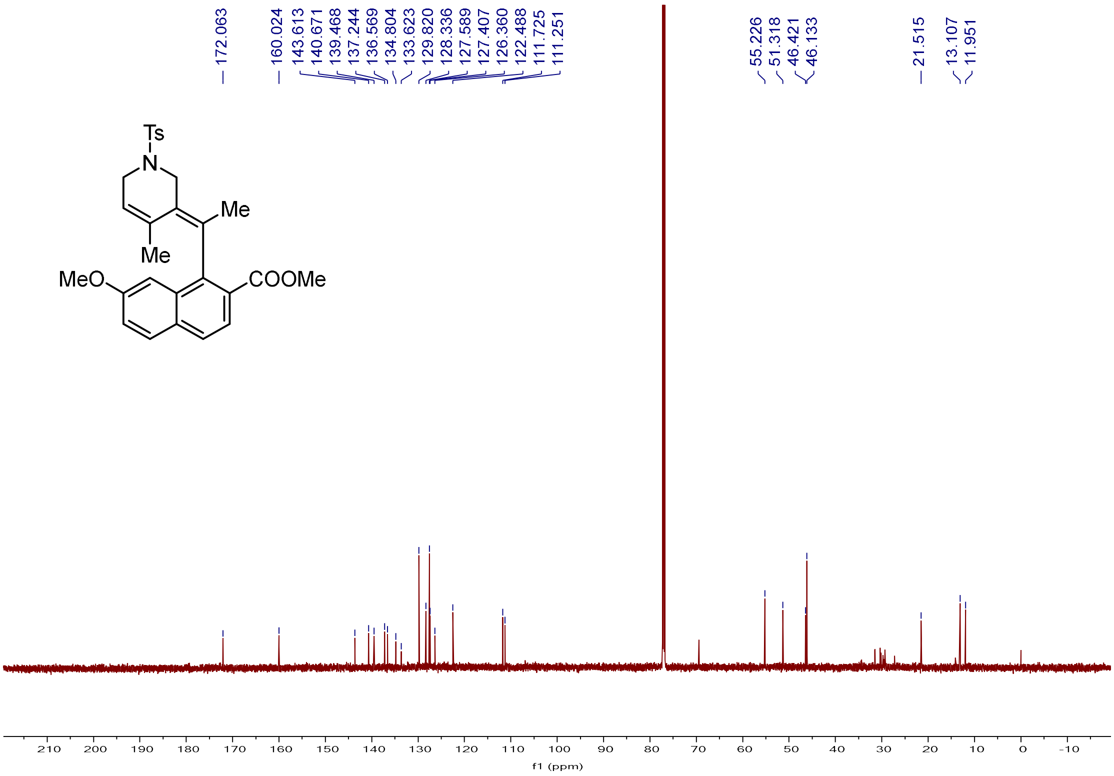

^{1}H NMR (600 MHz, Chloroform-d) spectrum of 44

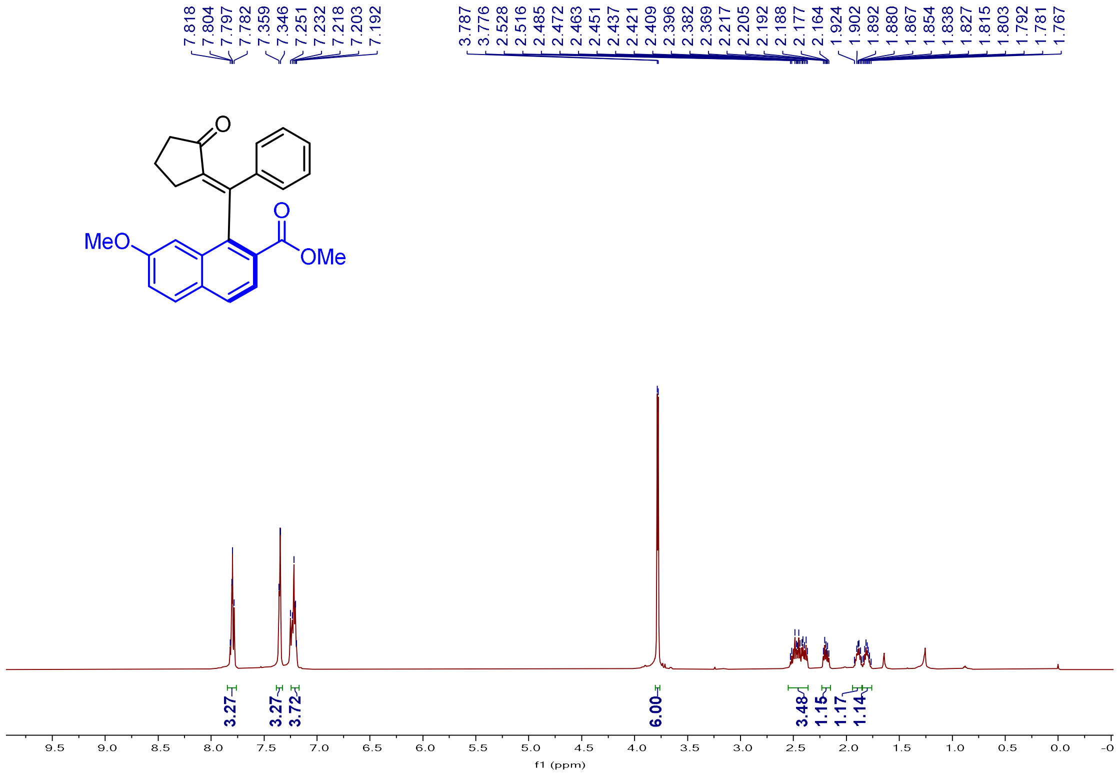

^{13}C NMR (150 MHz, Chloroform-d) spectrum of 44

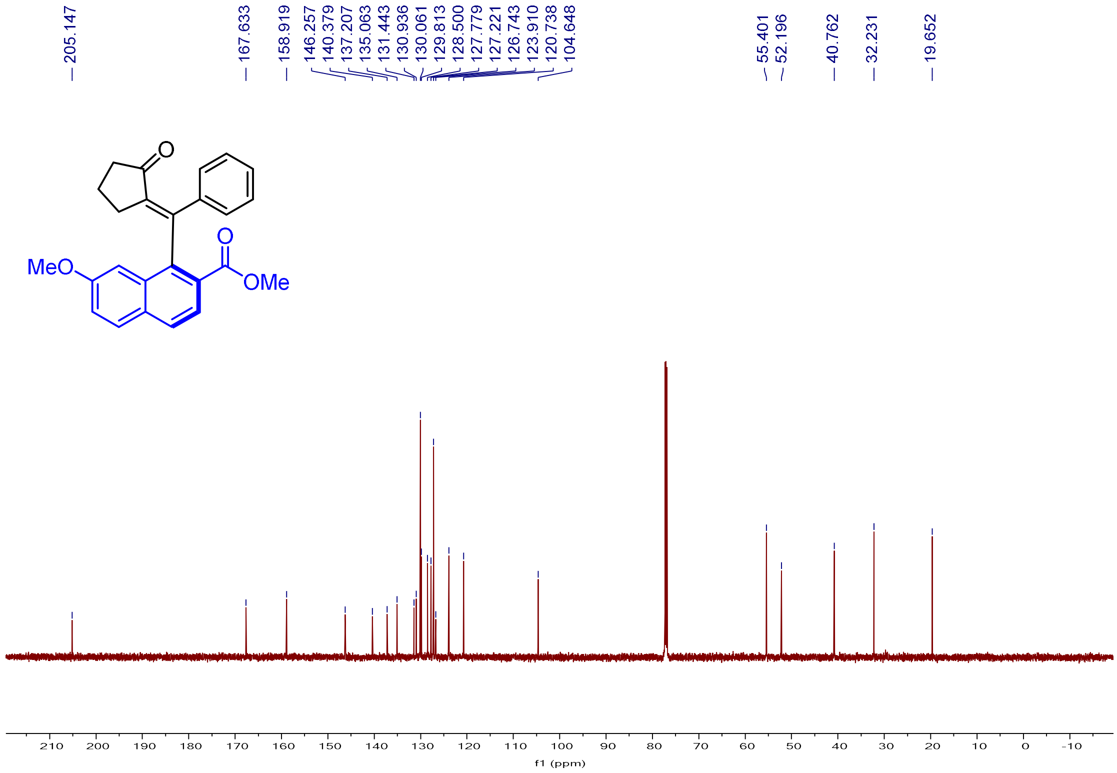

^{1}H NMR (600 MHz, Chloroform-d) spectrum of 45

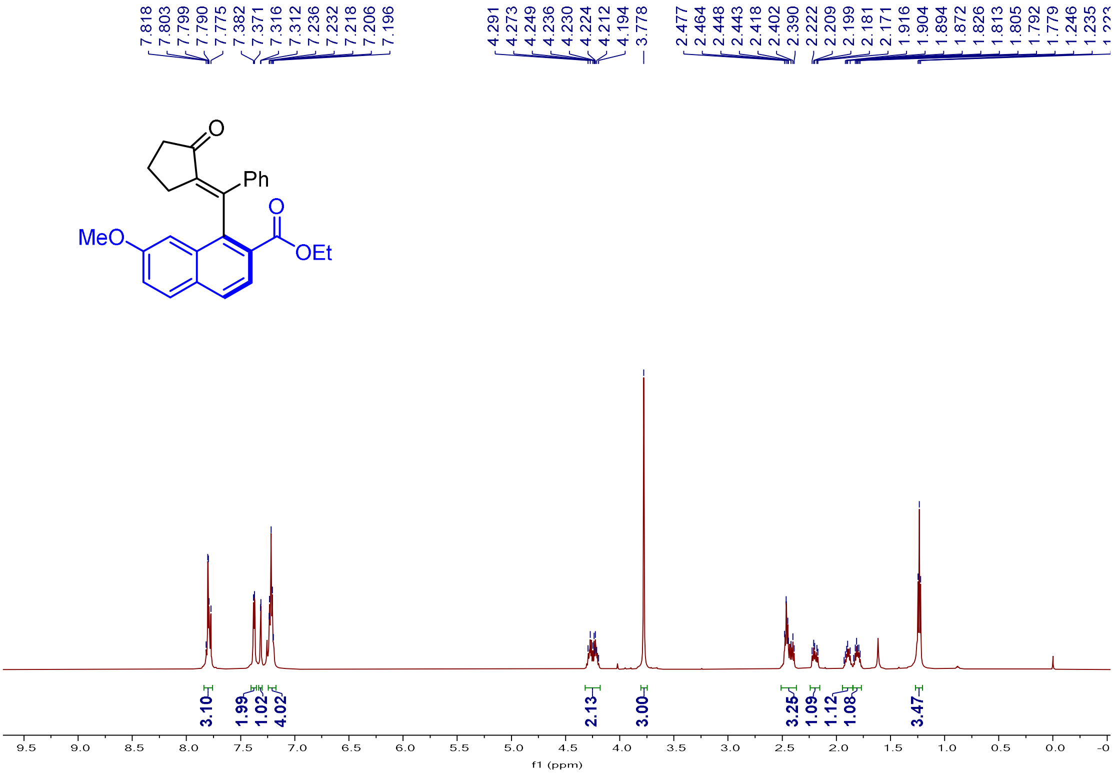

^{13}C NMR (150 MHz, Chloroform-d) spectrum of 45

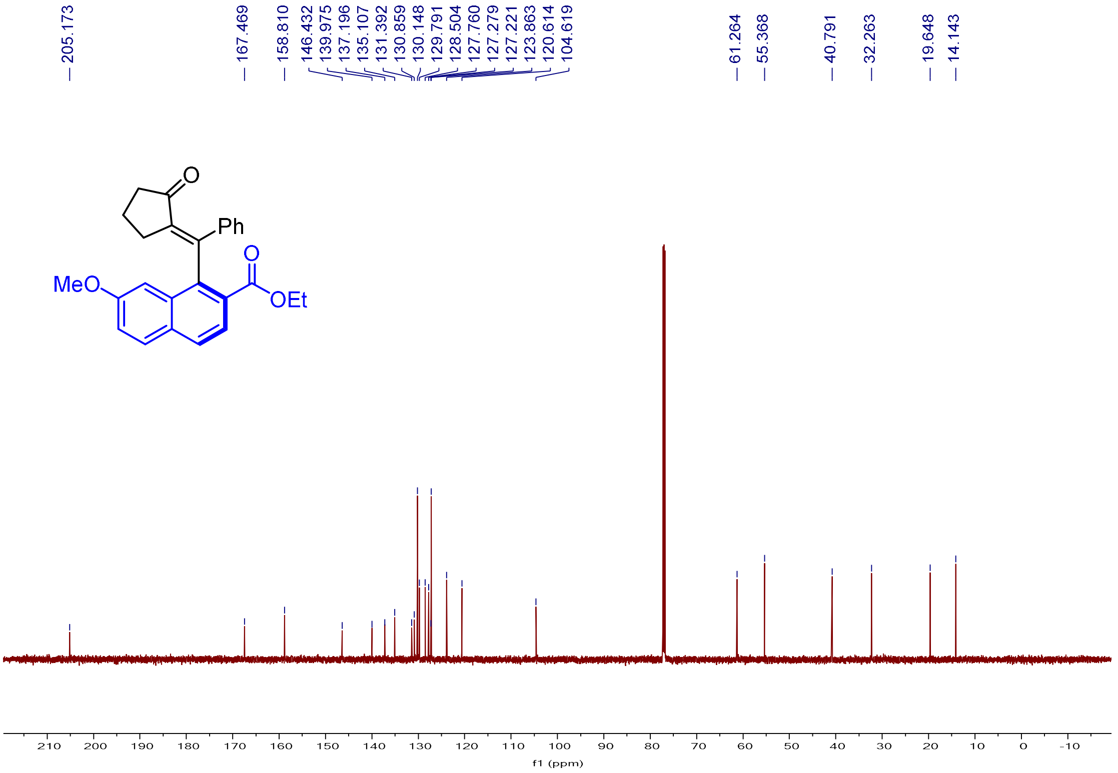

^1H NMR (600 MHz, Chloroform-d) spectrum of 46

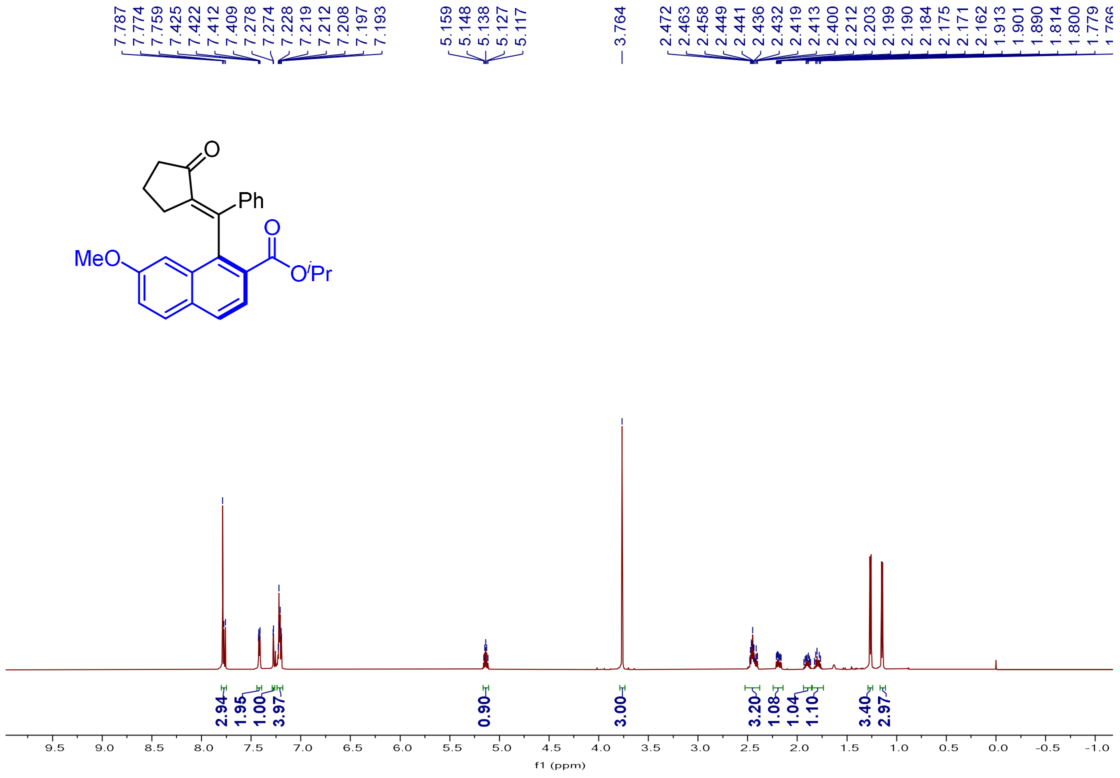

^{13}C NMR (150 MHz, Chloroform-d) spectrum of 46

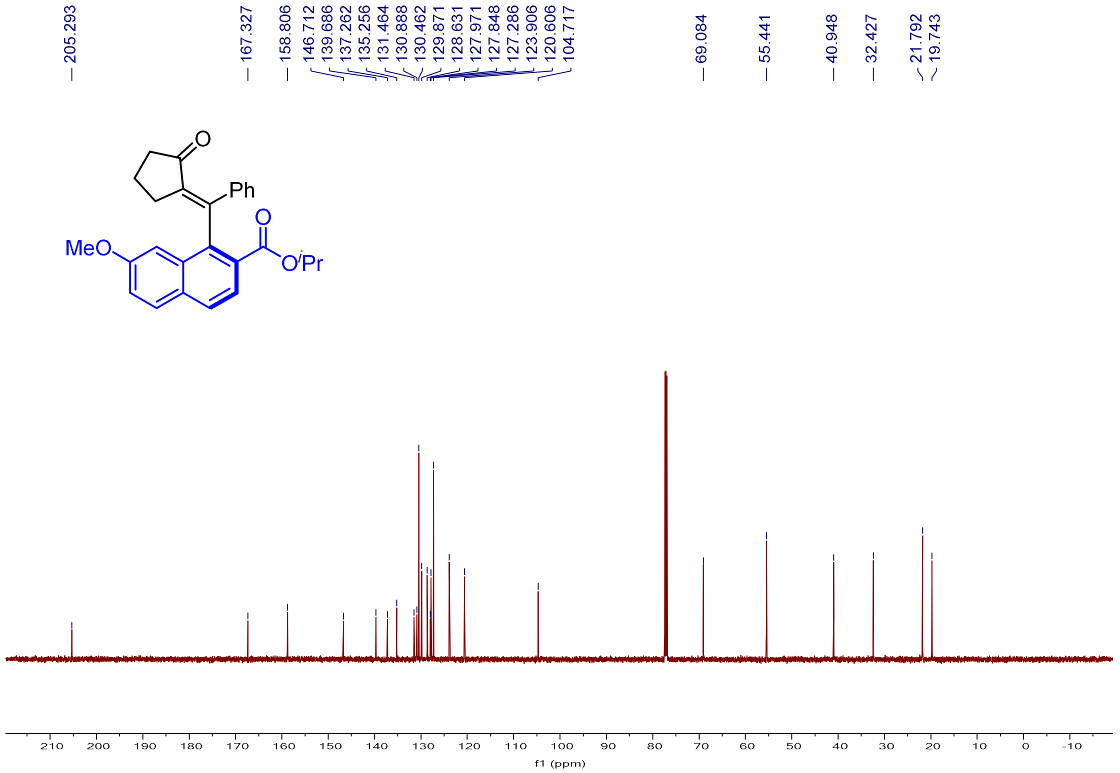

^{1}H NMR (600 MHz, Chloroform-d) spectrum of 47

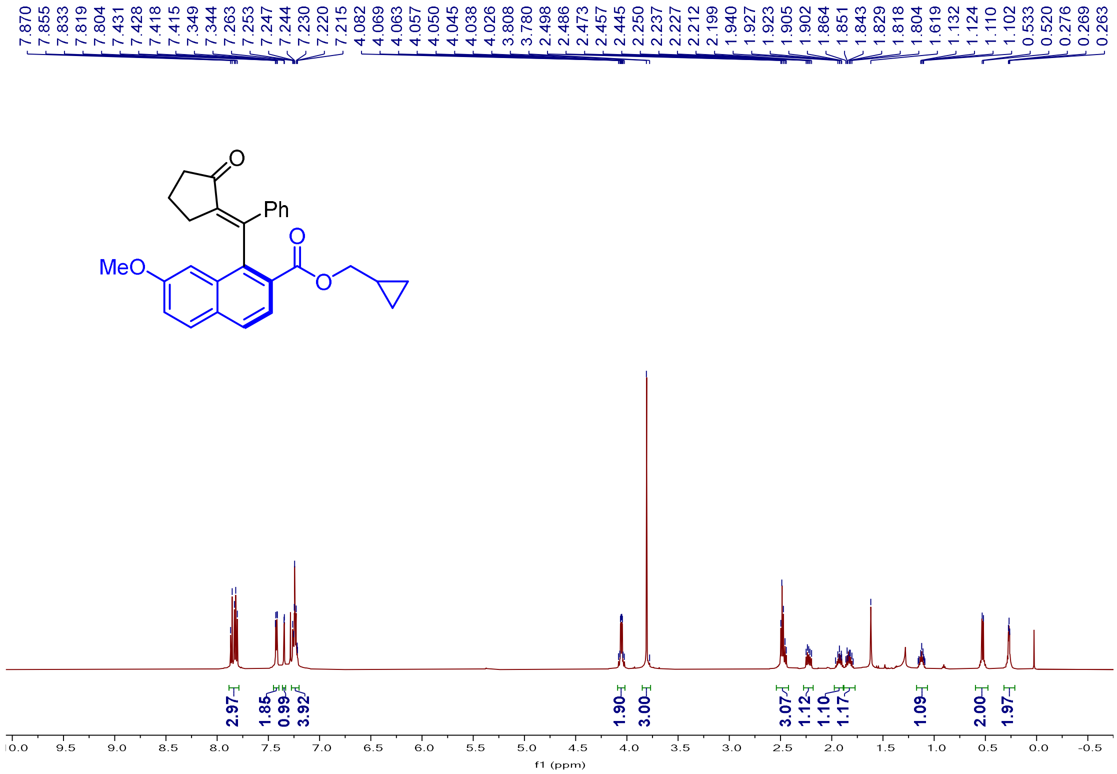

^{13}C NMR (150 MHz, Chloroform-d) spectrum of 47

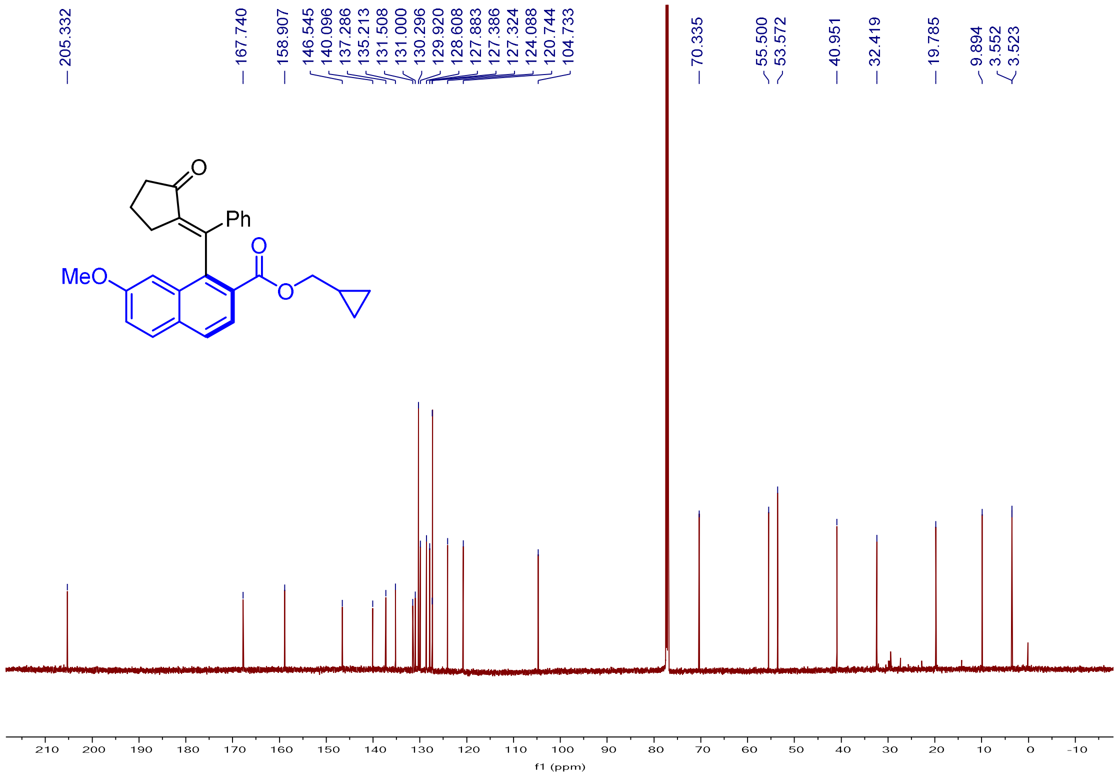

^{1}H NMR (600 MHz, Chloroform-d) spectrum of 48

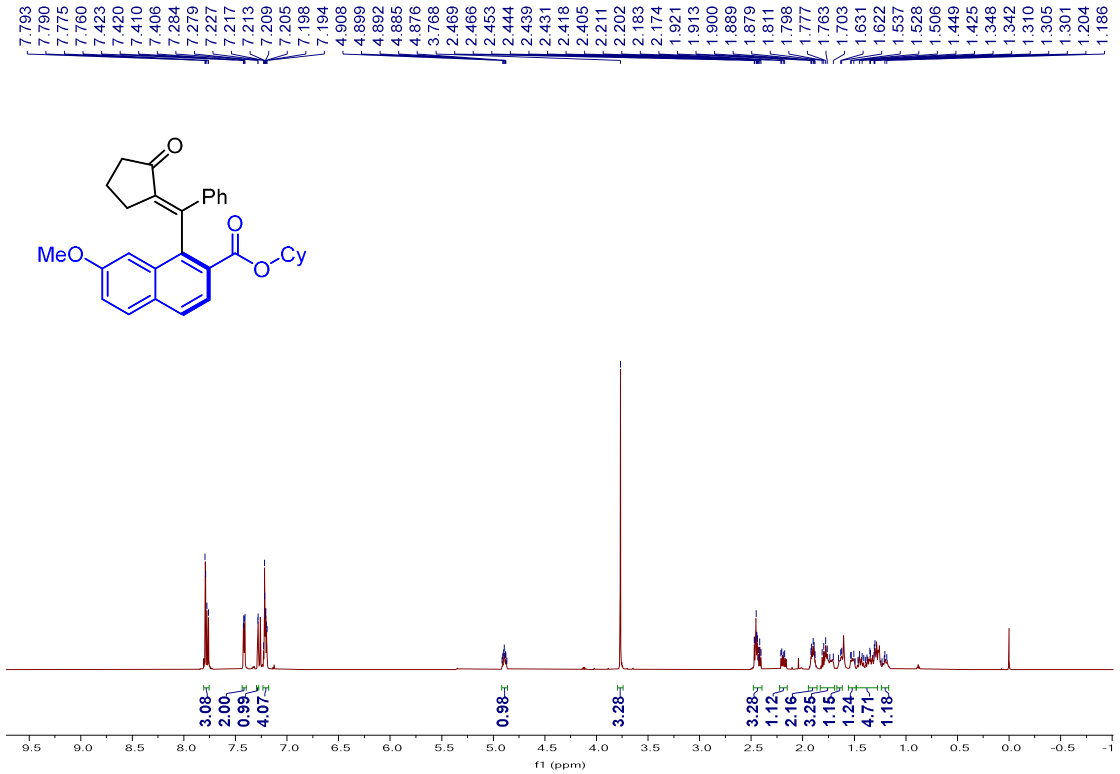

^{13}C NMR (150 MHz, Chloroform-d) spectrum of 48

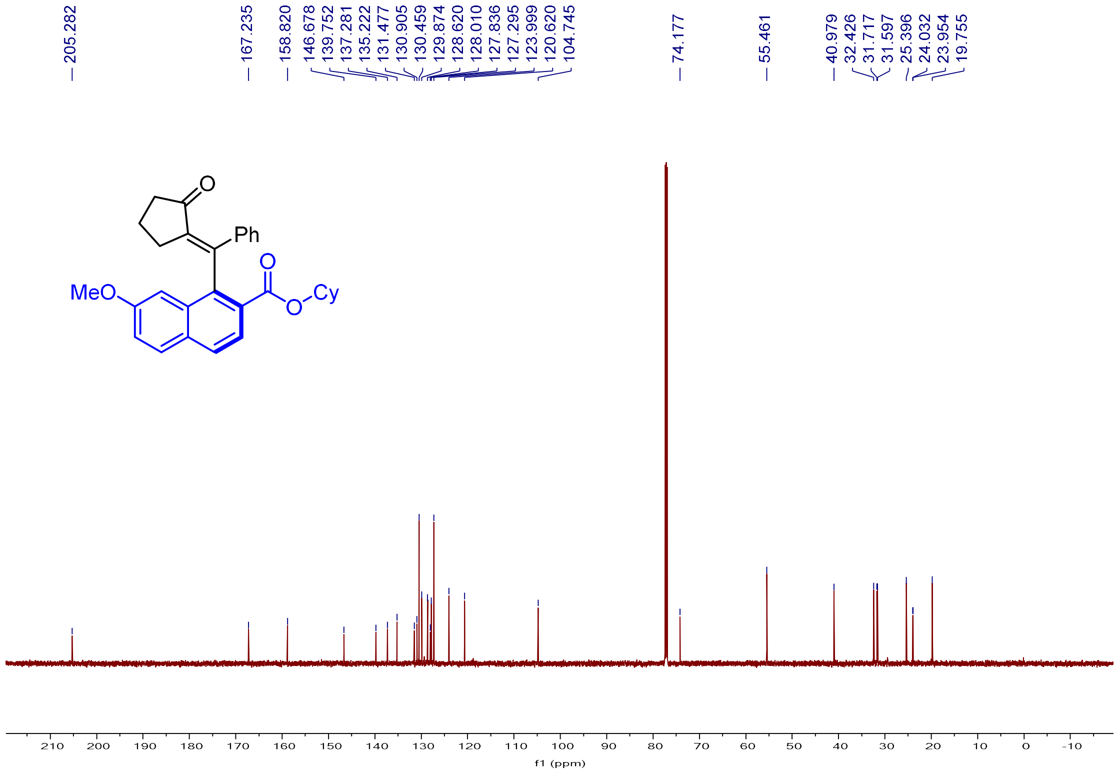

^{1}H NMR (600 MHz, Chloroform-d) spectrum of 49

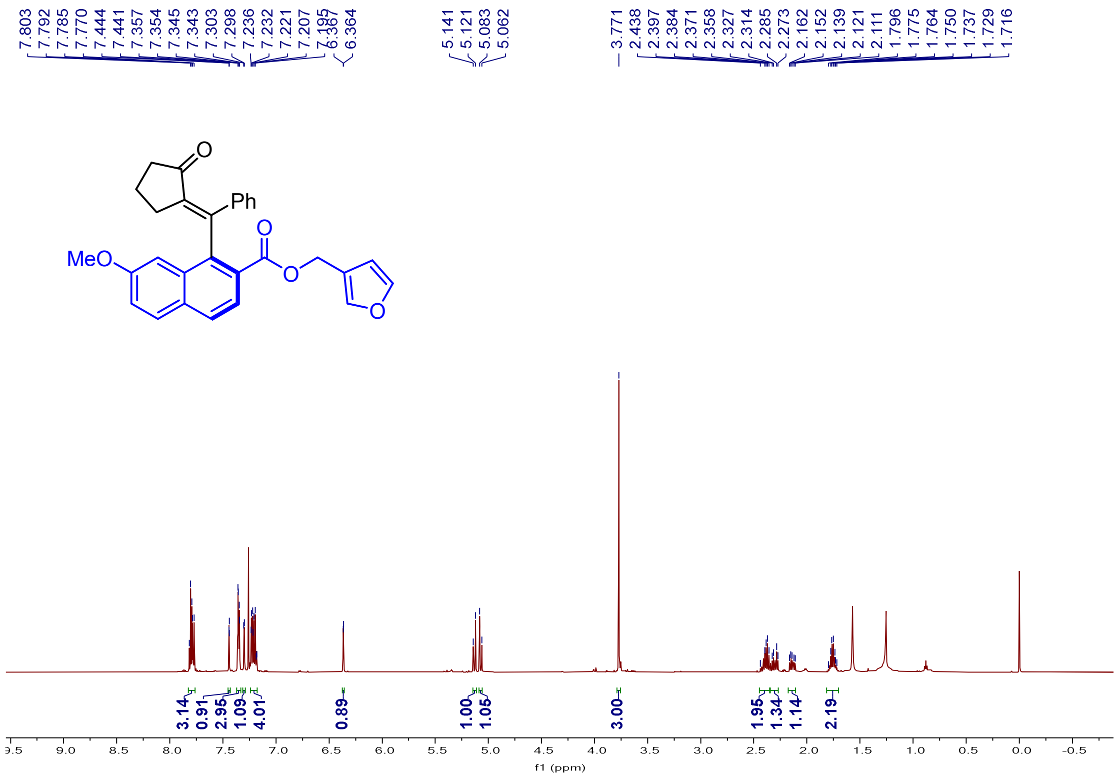

^{13}C NMR (150 MHz, Chloroform-d) spectrum of 49

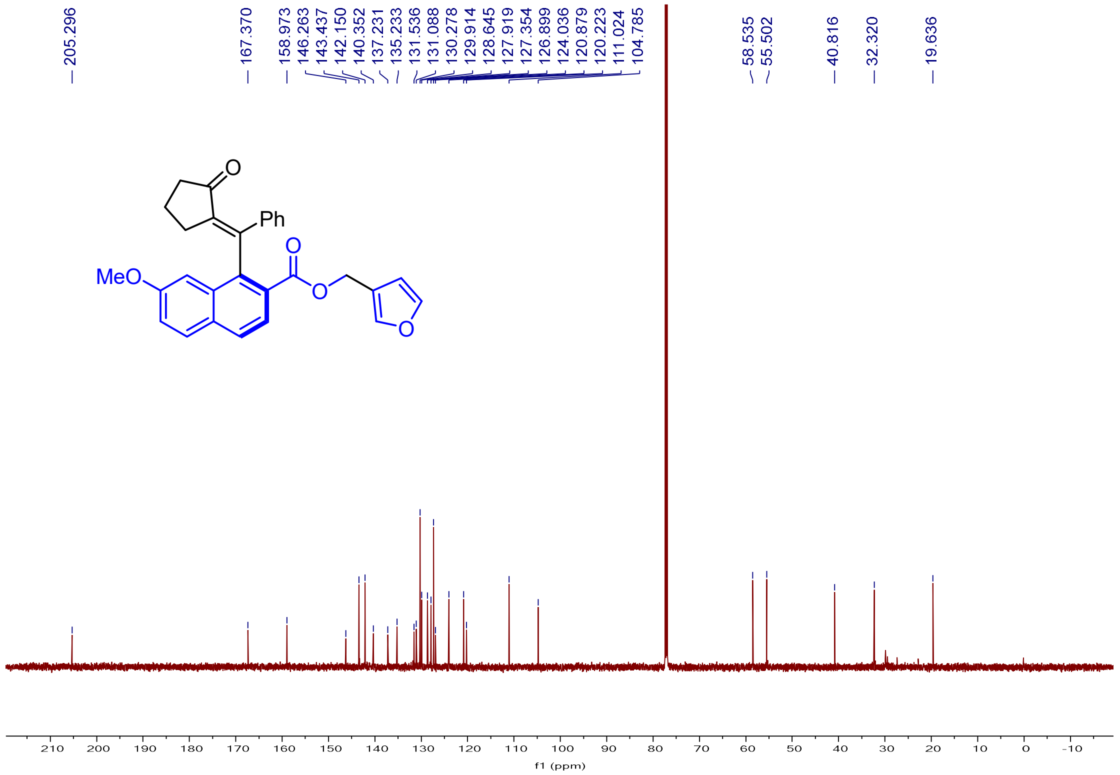

^{1}H NMR (600 MHz, Chloroform-d) spectrum of 50

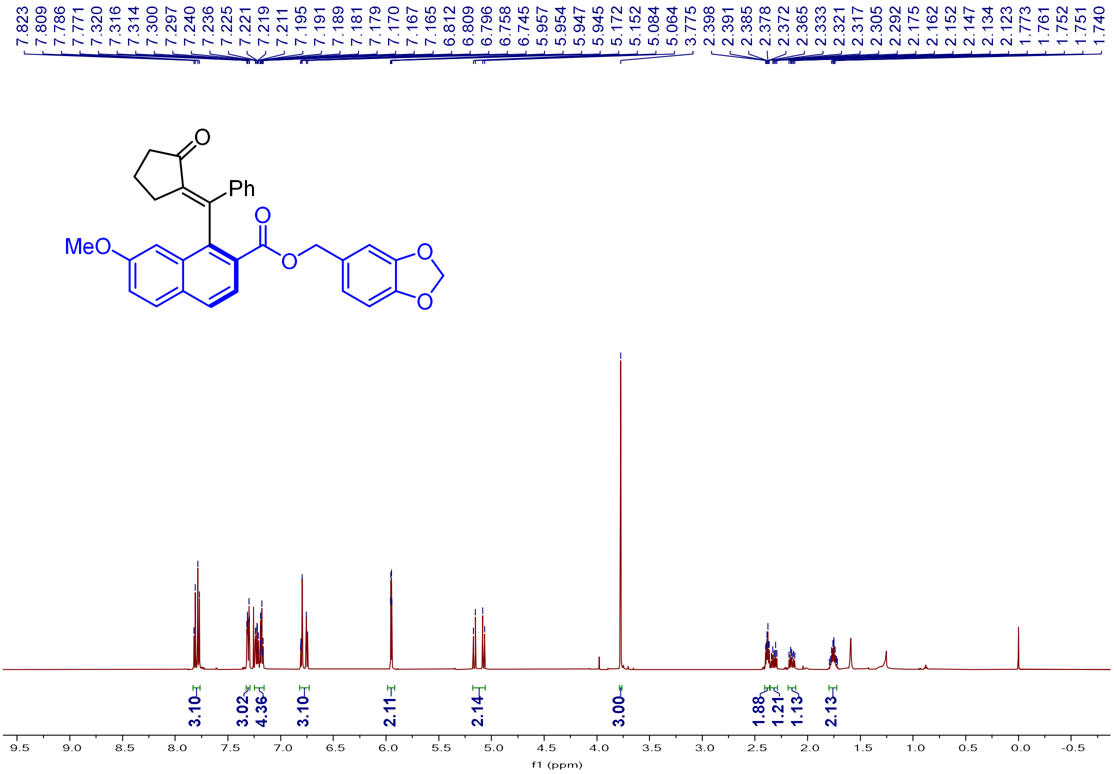

^{13}C NMR (150 MHz, Chloroform-d) spectrum of 50

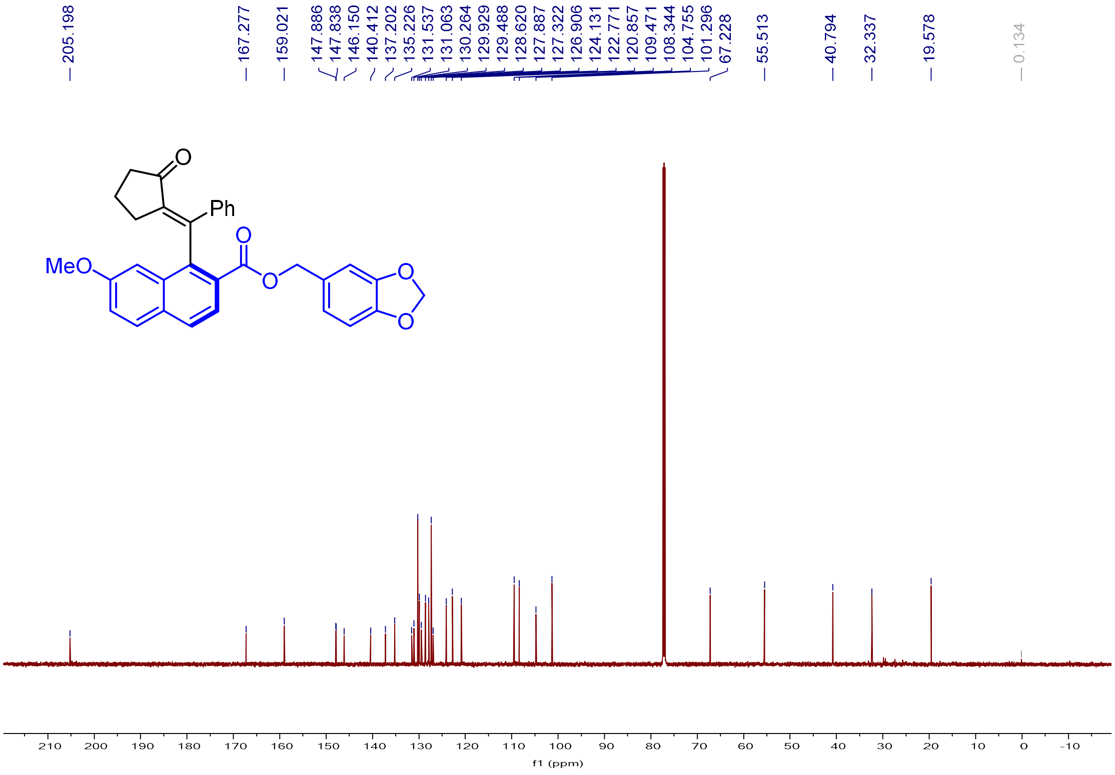

^{1}H NMR (600 MHz, Chloroform-d) spectrum of 51

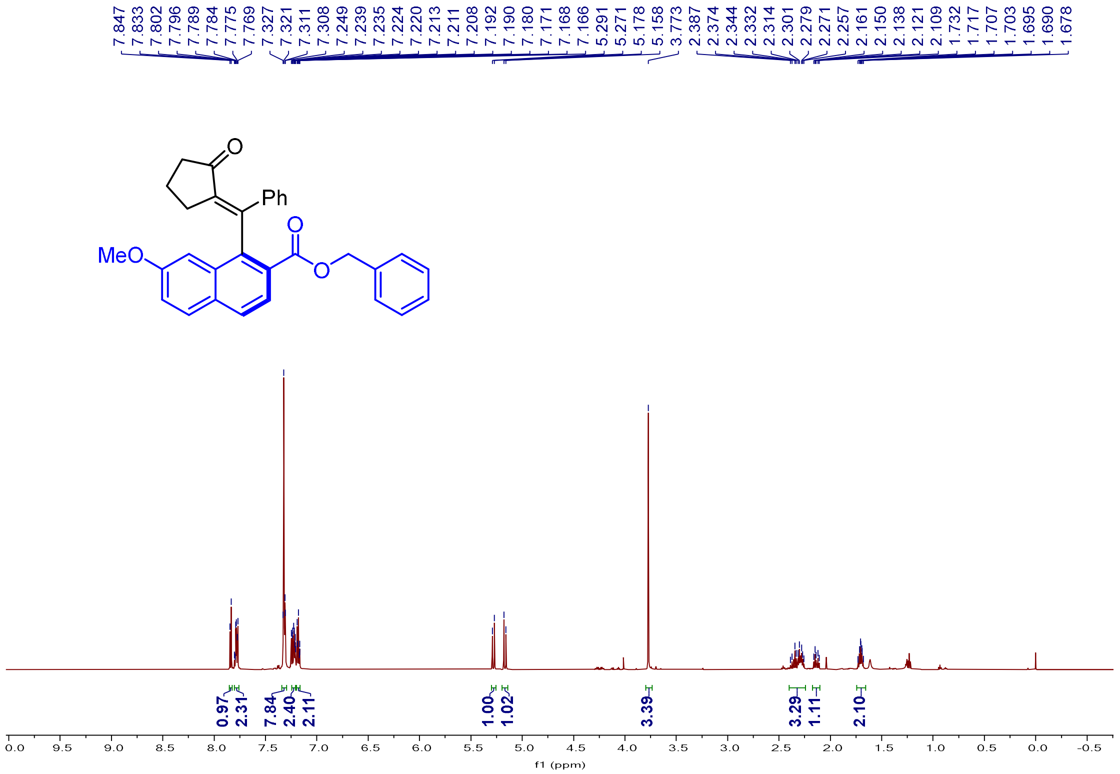

^{13}C NMR (150 MHz, Chloroform-d) spectrum of 51

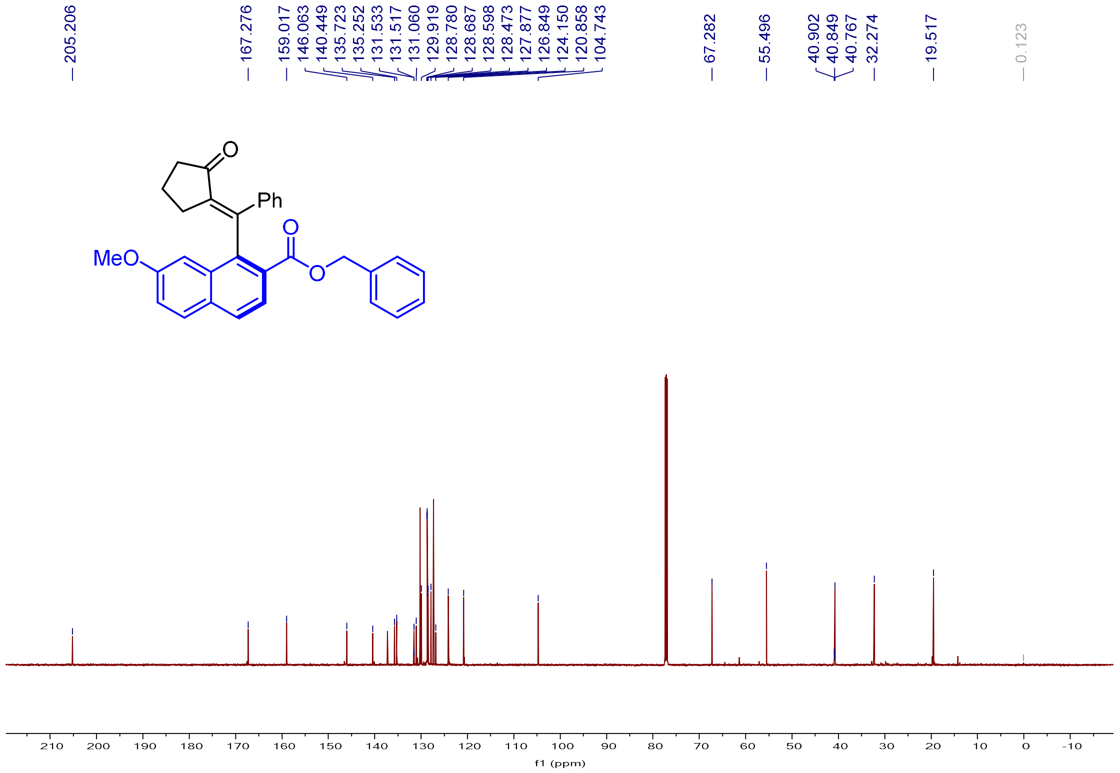

^{1}H NMR (600 MHz, Chloroform-d) spectrum of 52

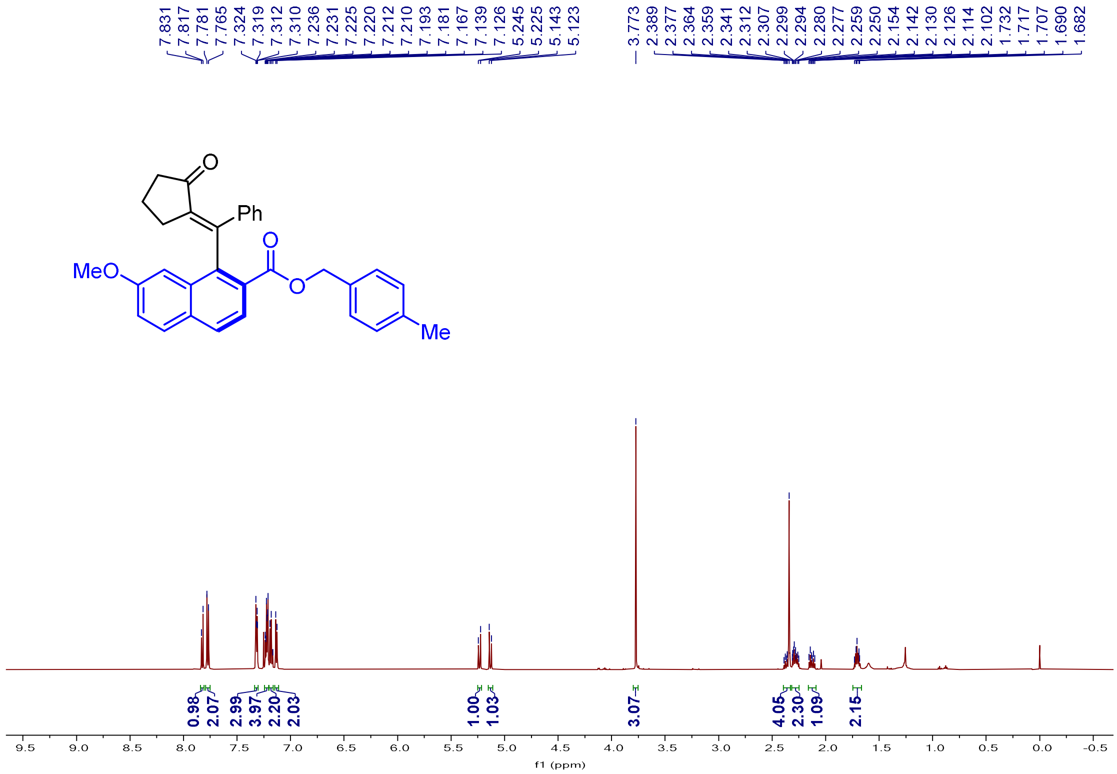

^{13}C NMR (150 MHz, Chloroform-d) spectrum of 52

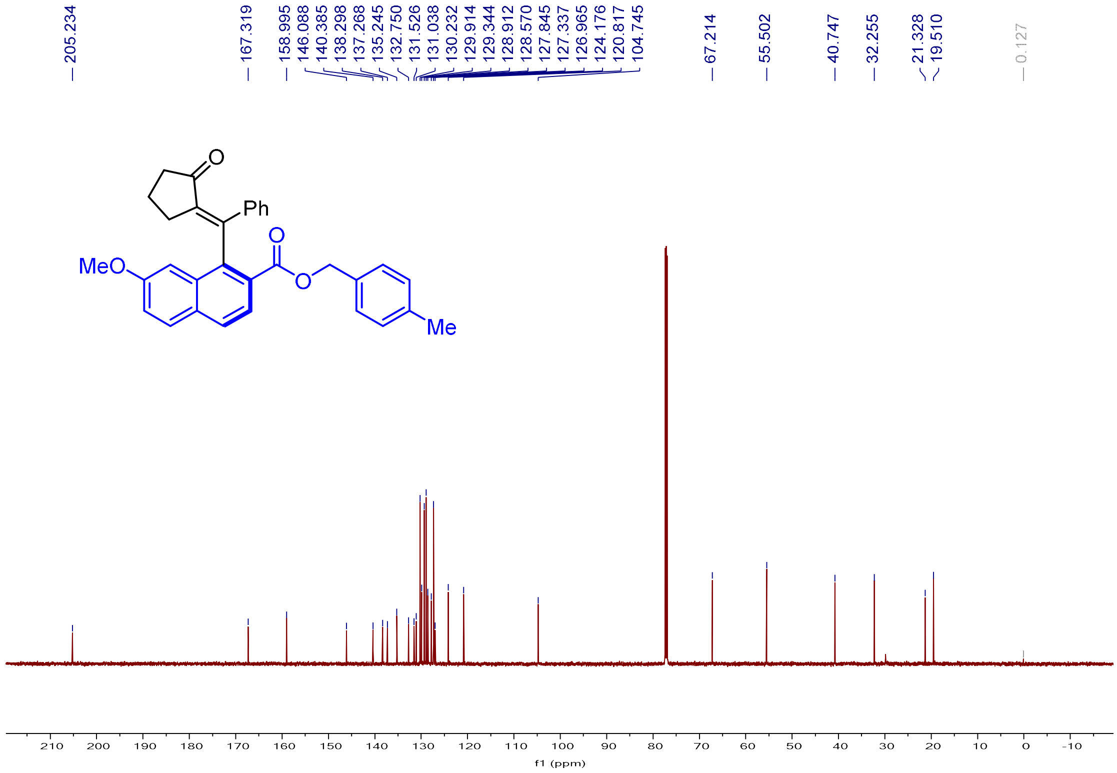

^{1}H NMR (600 MHz, Chloroform-d) spectrum of 53

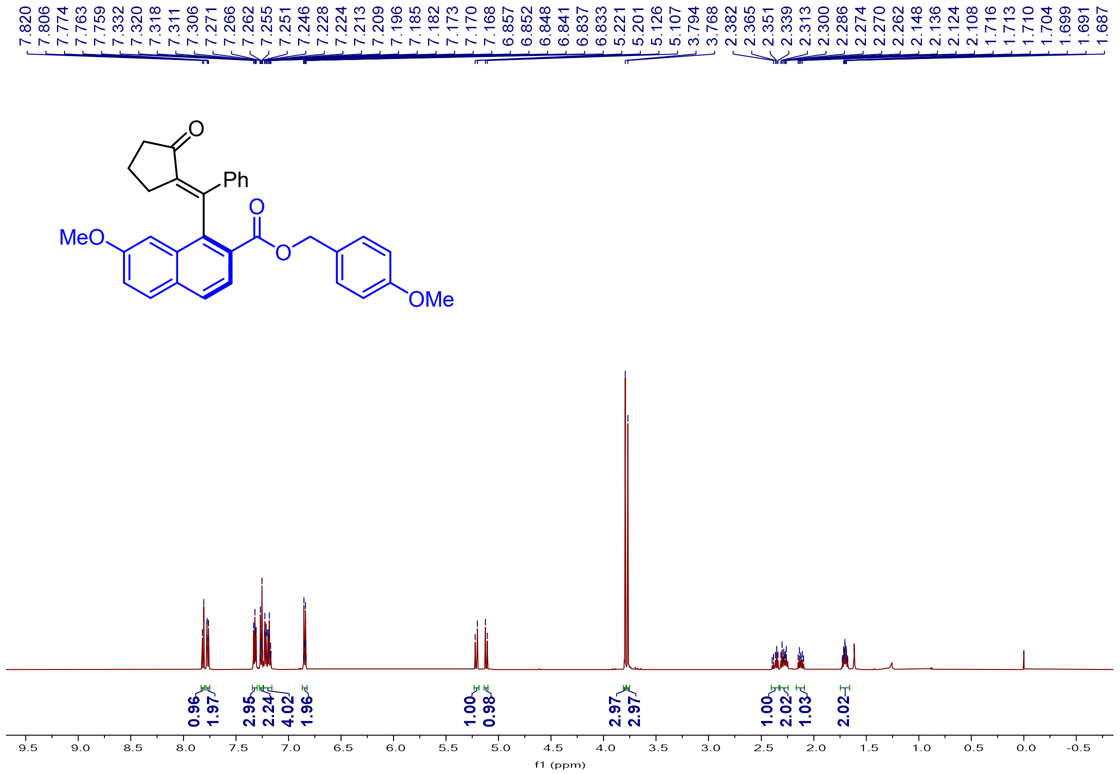

^{13}C NMR (150 MHz, Chloroform-d) spectrum of 53

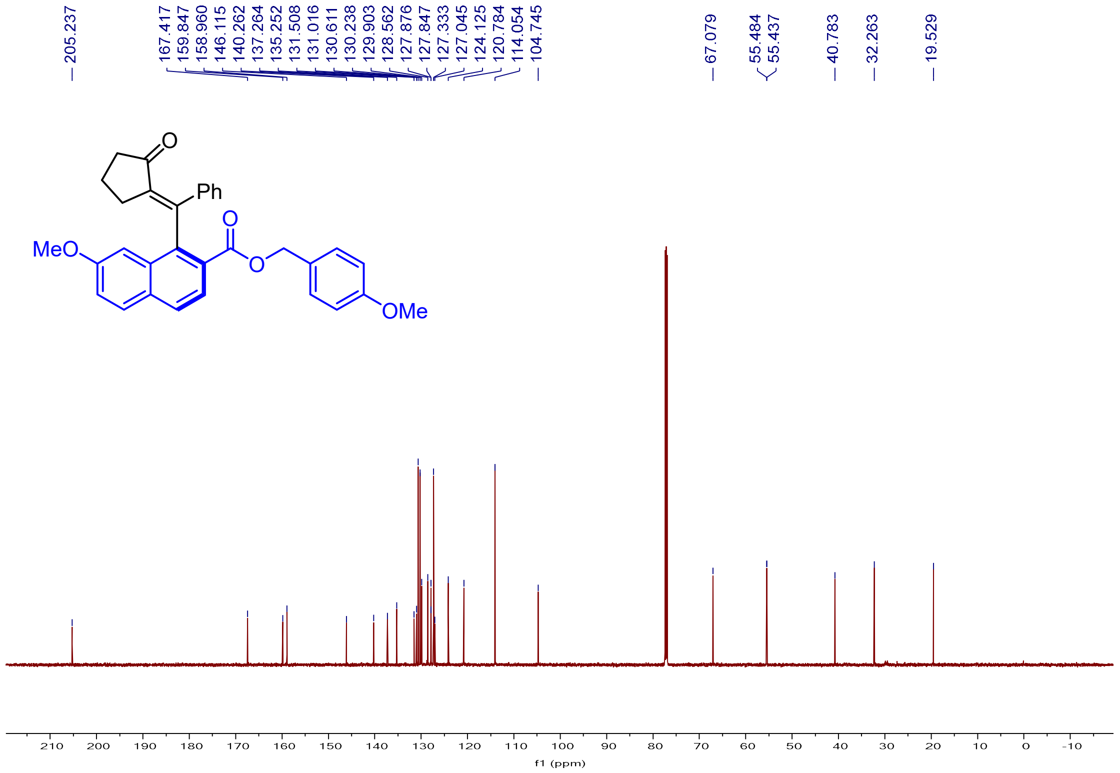

^{1}H NMR (600 MHz, Chloroform-d) spectrum of 54

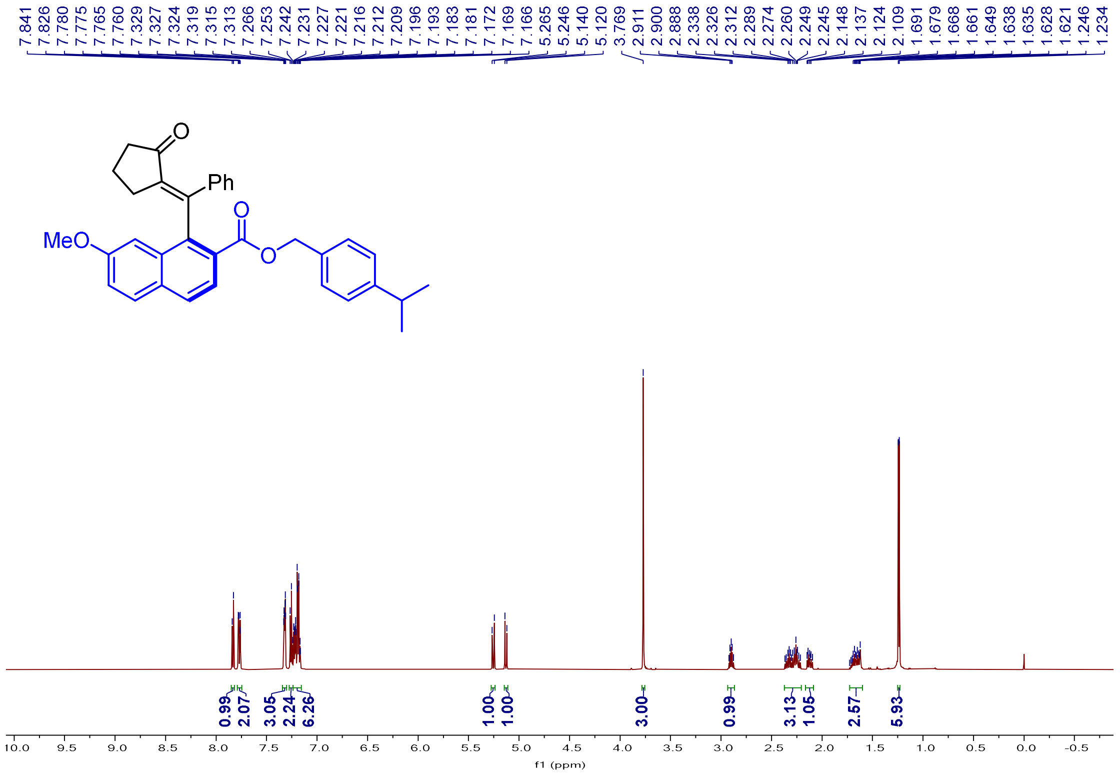

^{13}C NMR (150 MHz, Chloroform-d) spectrum of 54

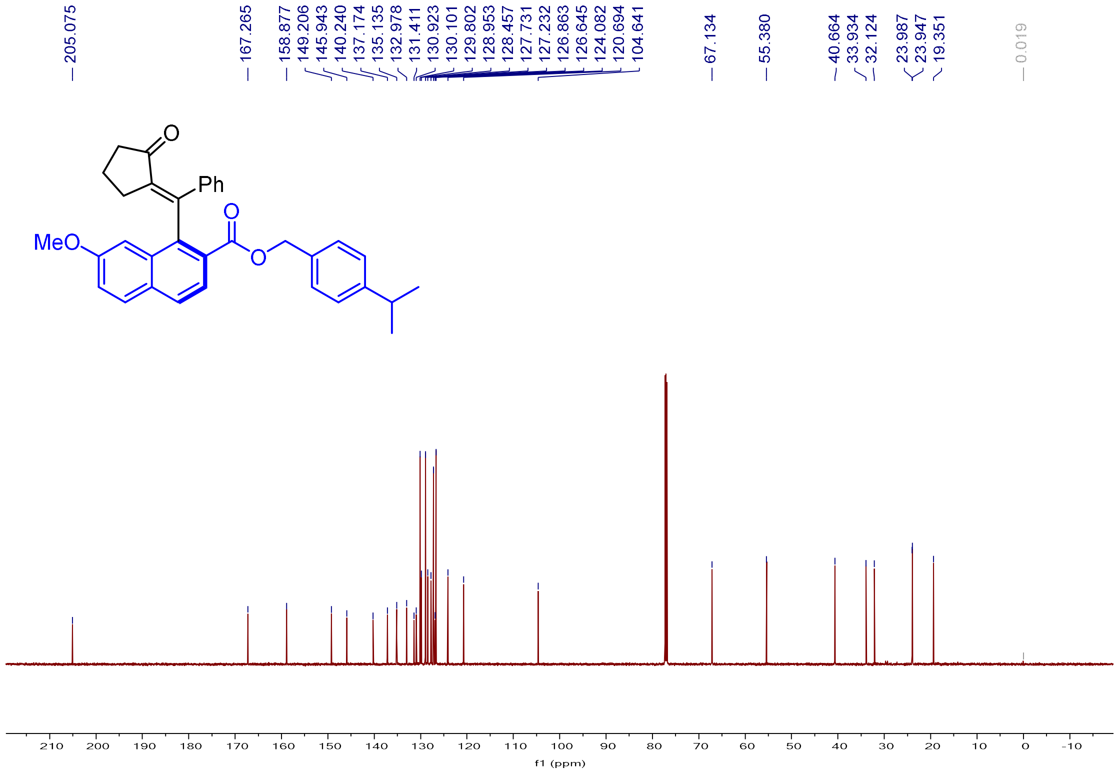

^{1}H NMR (600 MHz, Chloroform-d) spectrum of 55

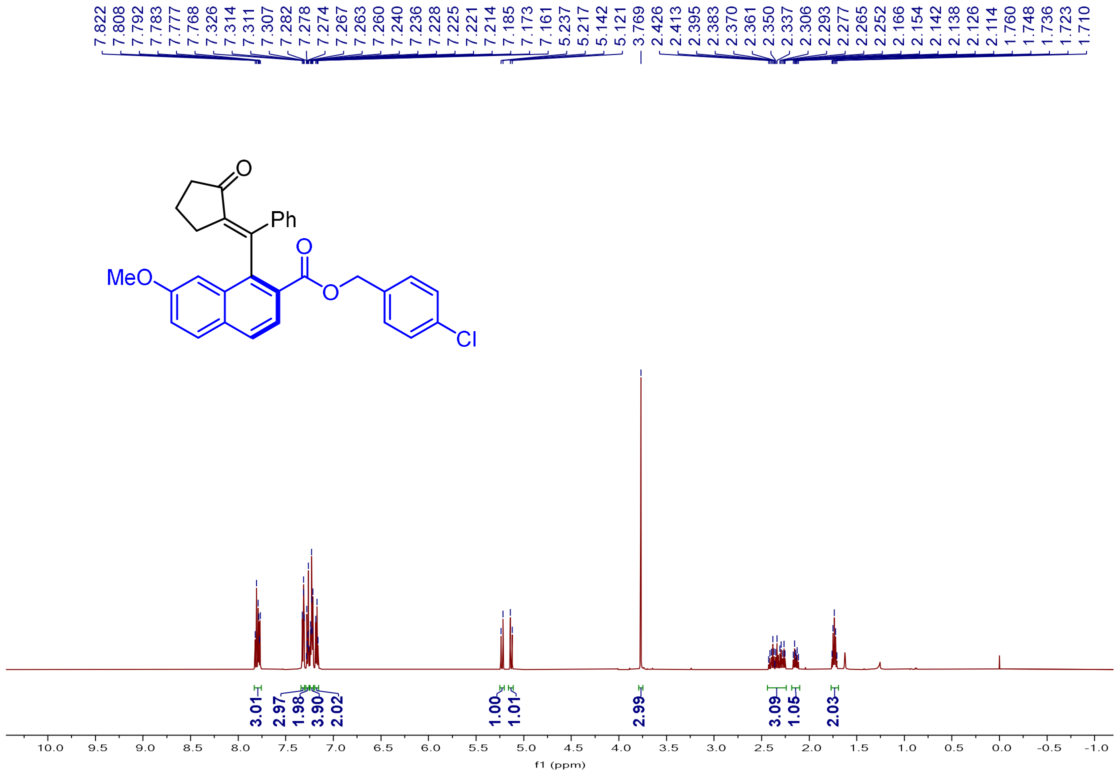

^{13}C NMR (150 MHz, Chloroform-d) spectrum of 55

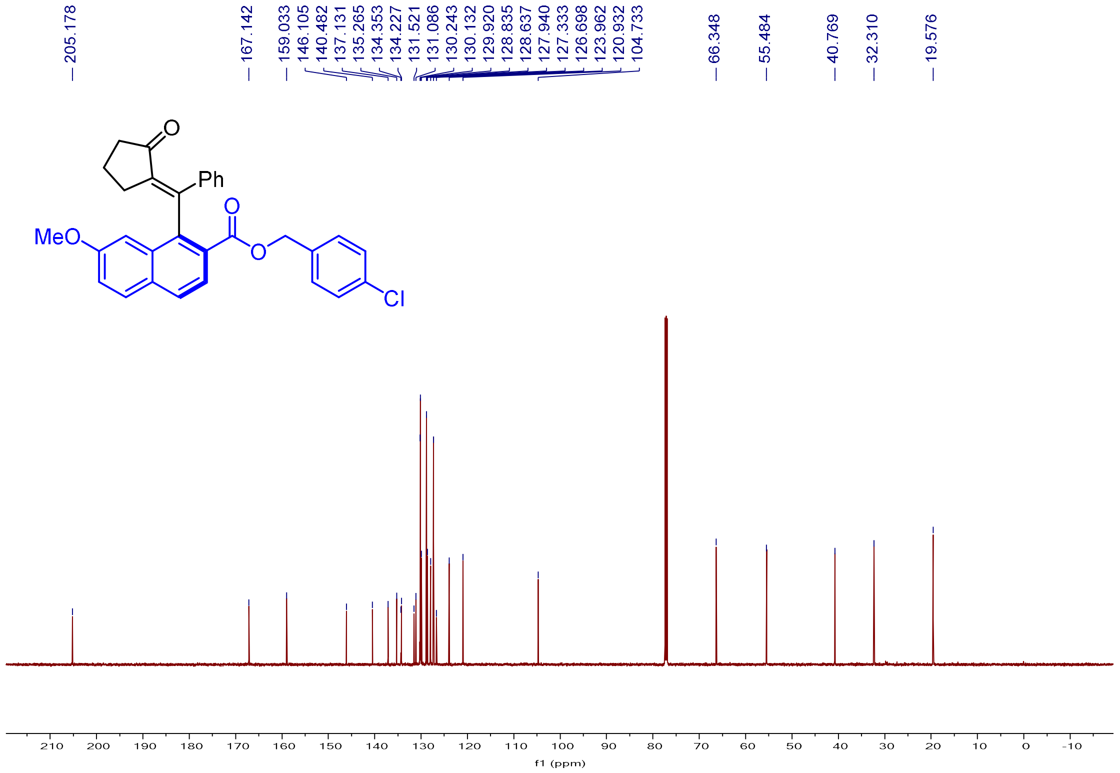

^{1}H NMR (600 MHz, Chloroform-d) spectrum of 56

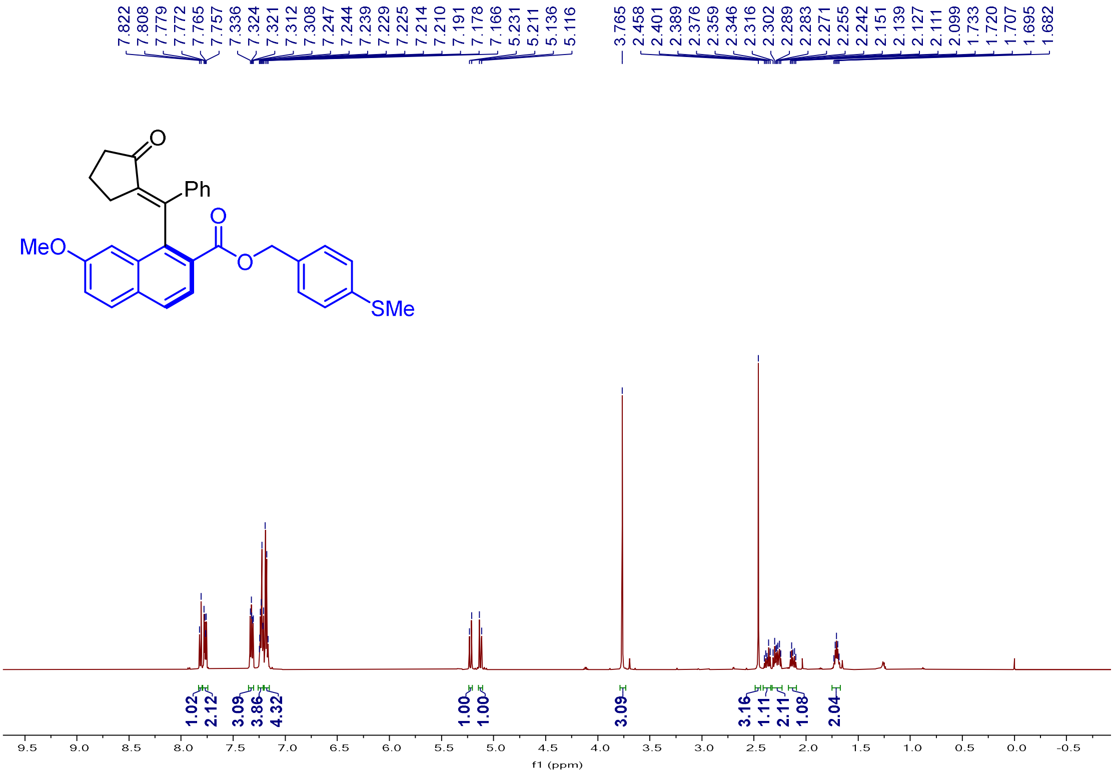

^{13}C NMR (150 MHz, Chloroform-d) spectrum of 56

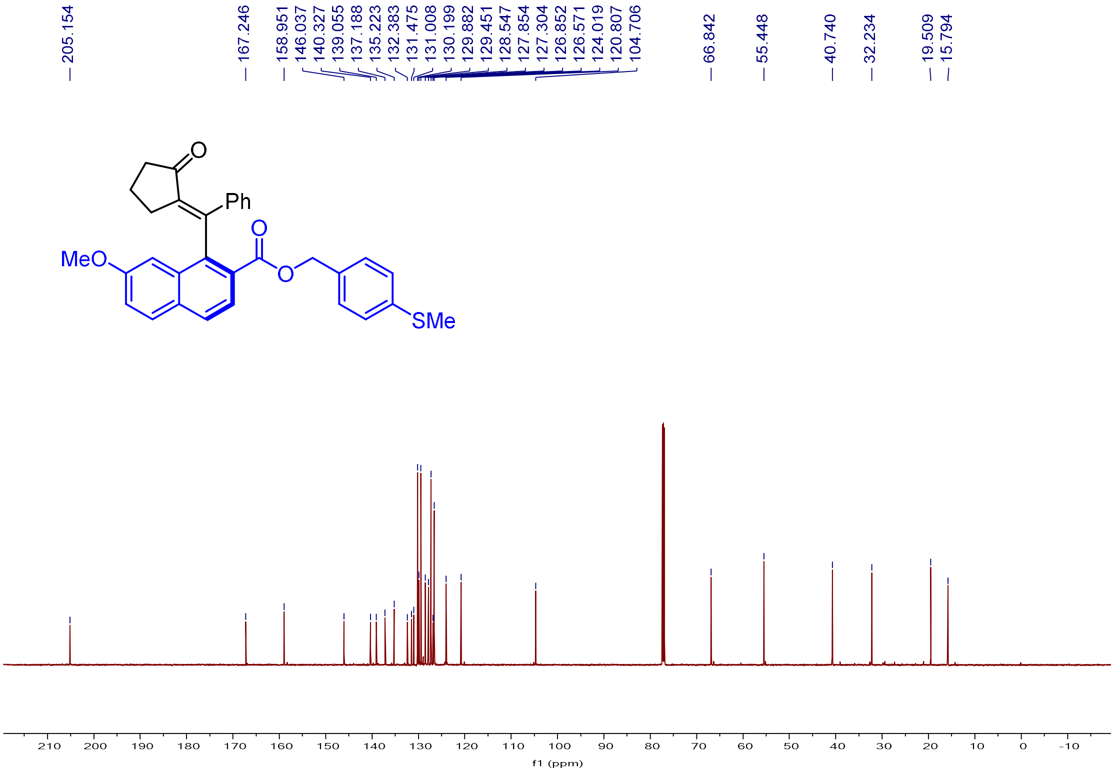

^{1}H NMR (600 MHz, Chloroform-d) spectrum of 57

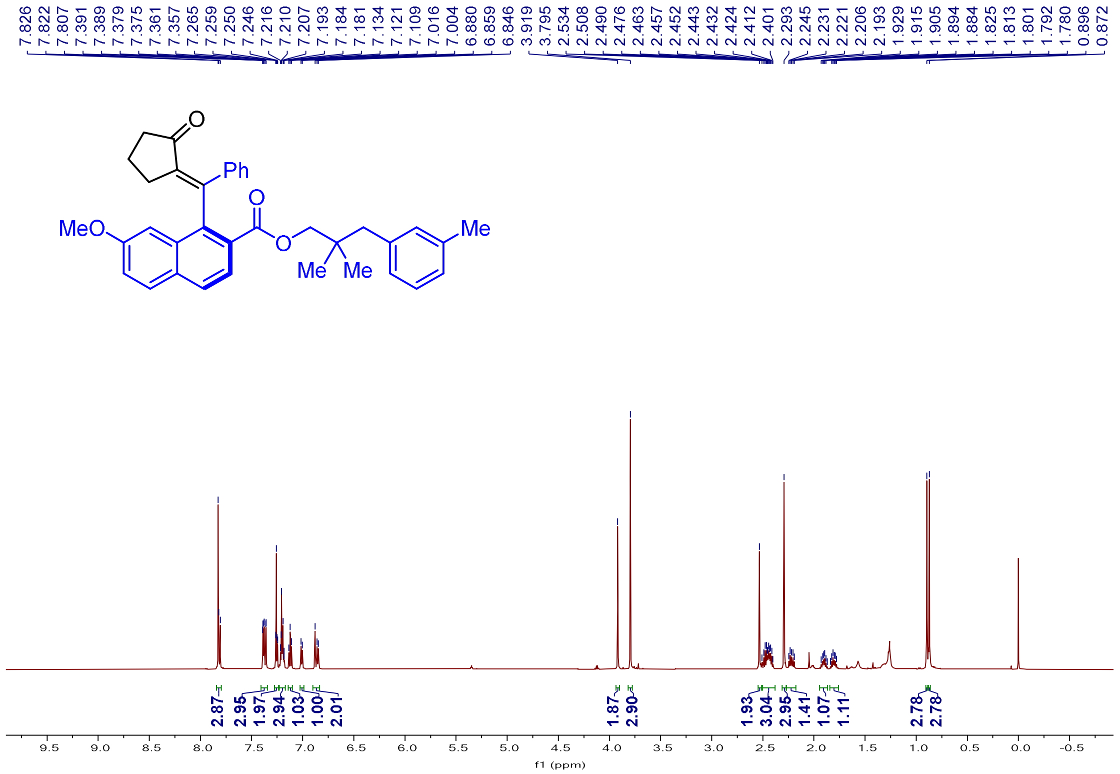

^{13}C NMR (150 MHz, Chloroform-d) spectrum of 57

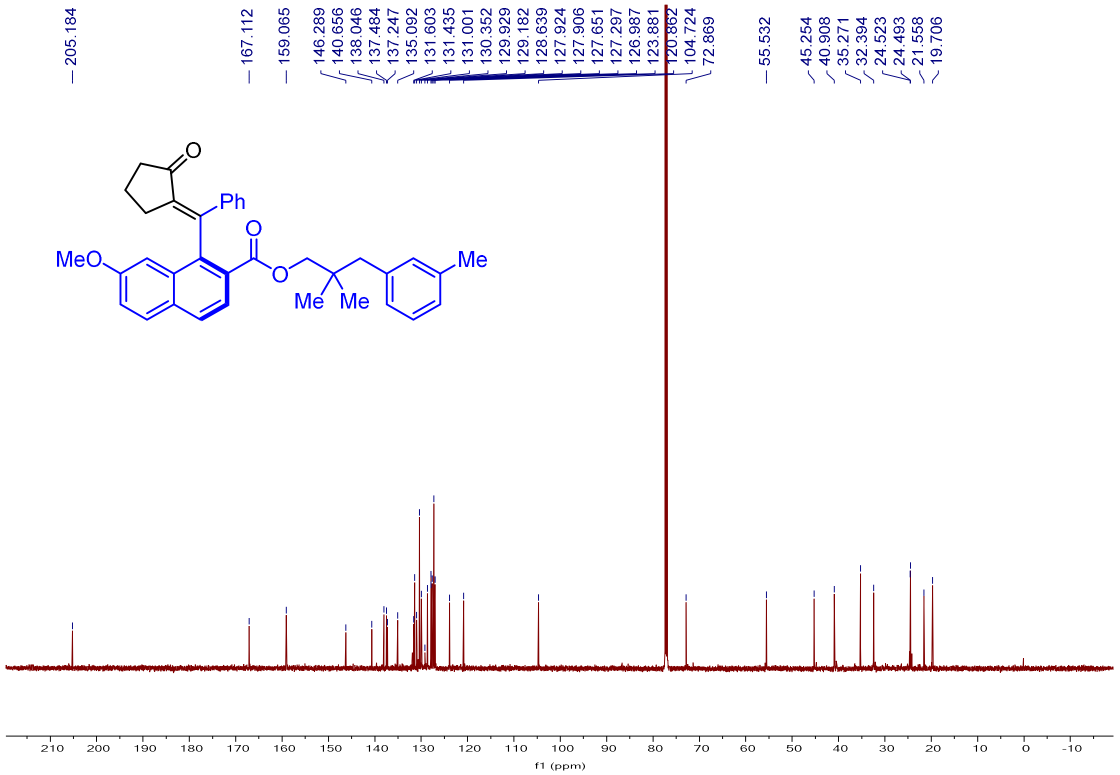

^{1}H NMR (600 MHz, Chloroform-d) spectrum of 58

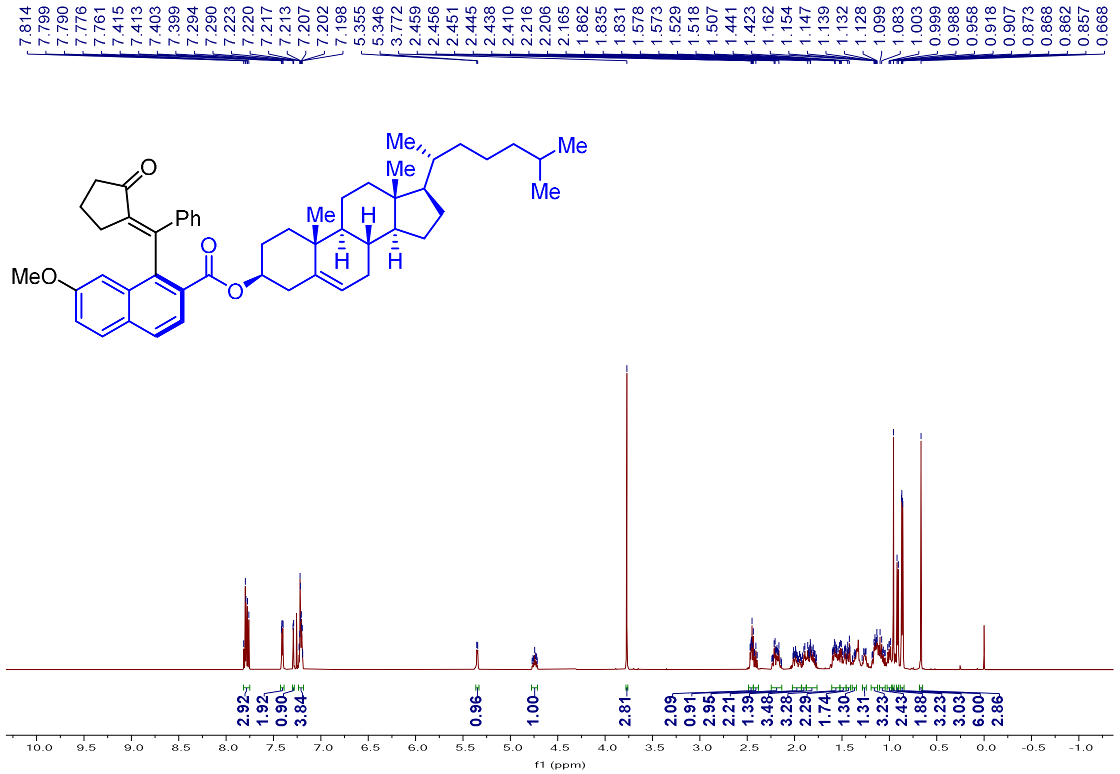

^{13}C NMR (150 MHz, Chloroform-d) spectrum of 58

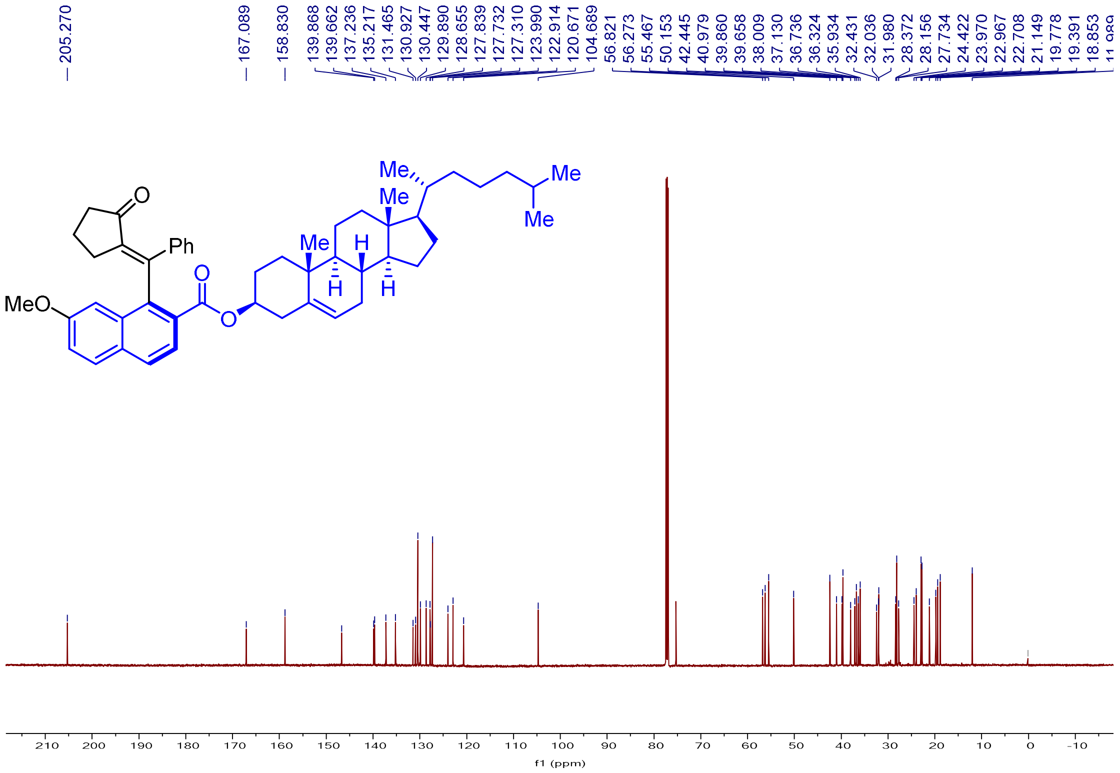

¹H NMR (600 MHz, Chloroform-d) spectrum of 59

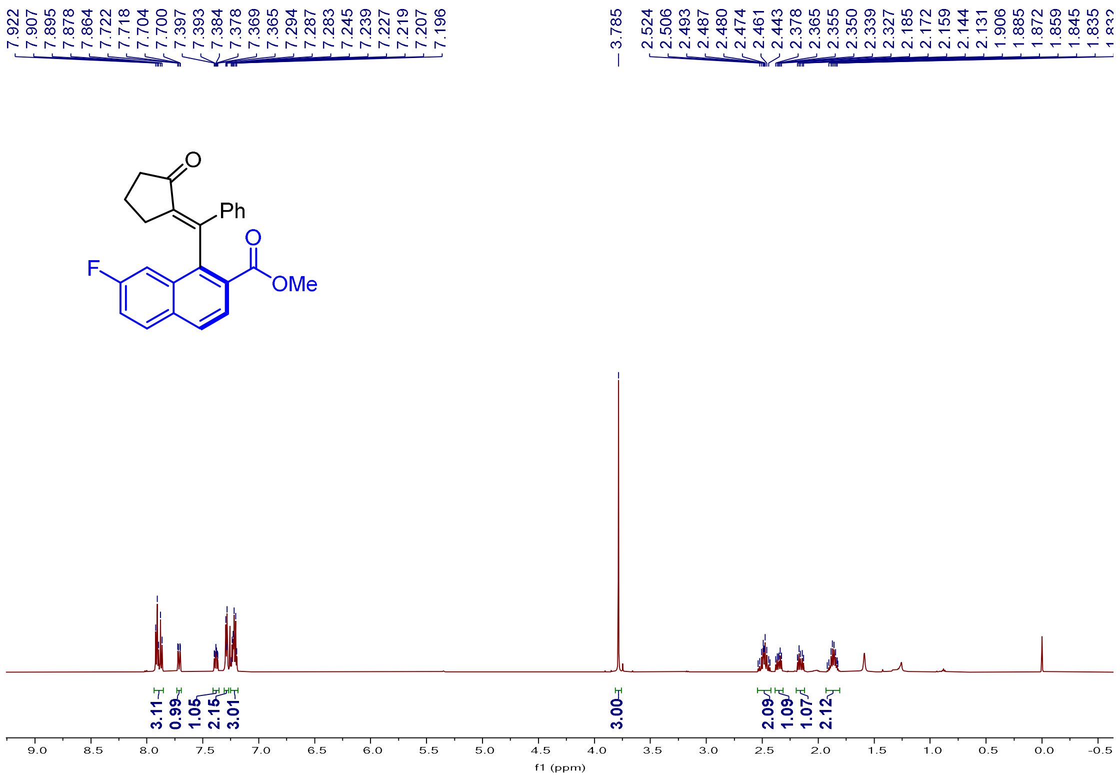

¹³C NMR (150 MHz, Chloroform-d) spectrum of 59

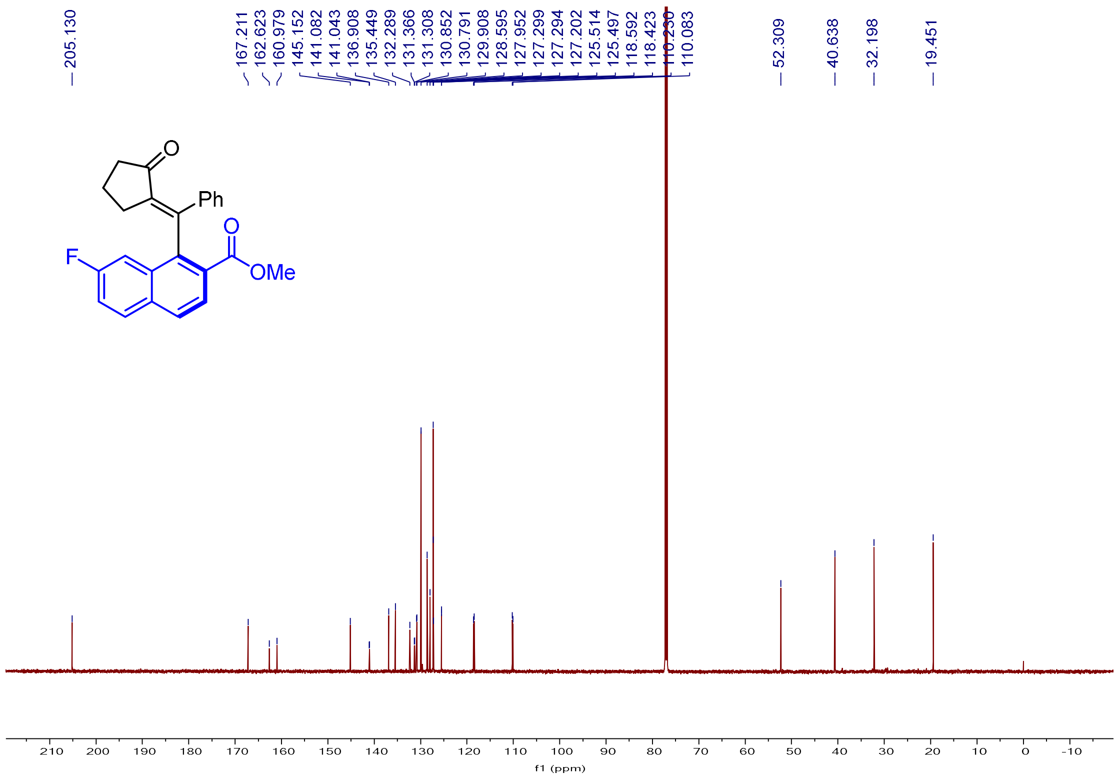

^{19}F NMR (376 MHz, Chloroform-d) spectrum of 59

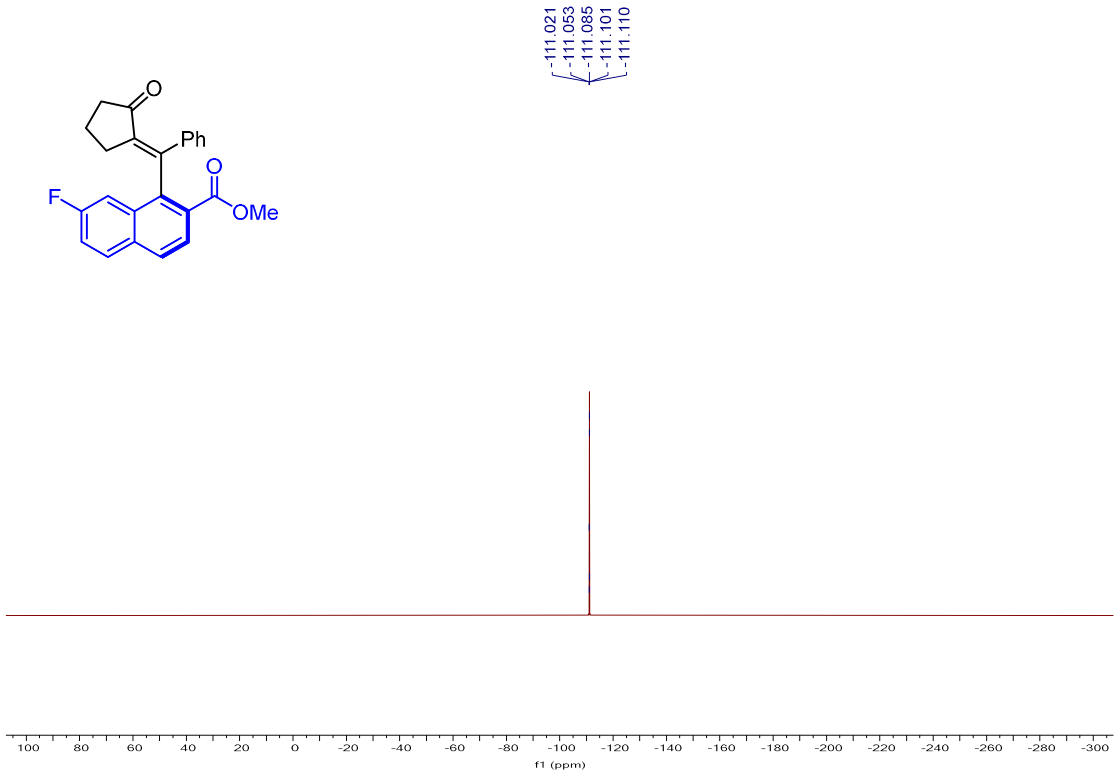

^{1}H NMR (600 MHz, Chloroform-d) spectrum of 60

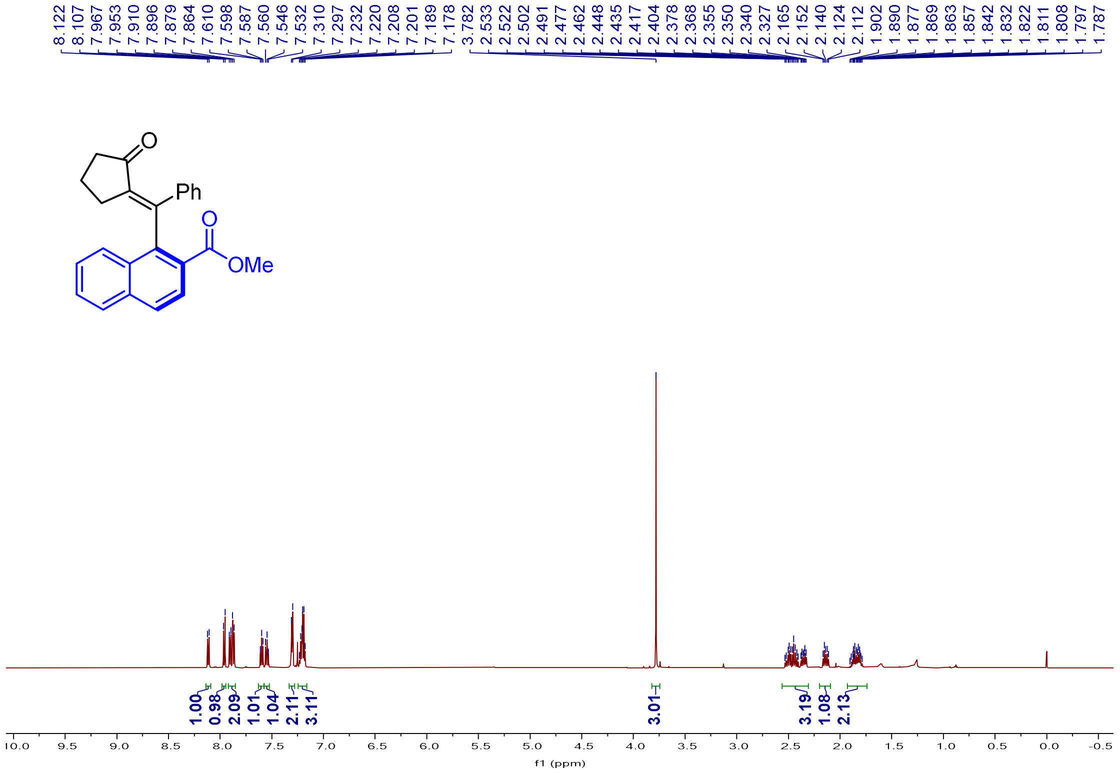

^{13}C NMR (150 MHz, Chloroform-d) spectrum of 60

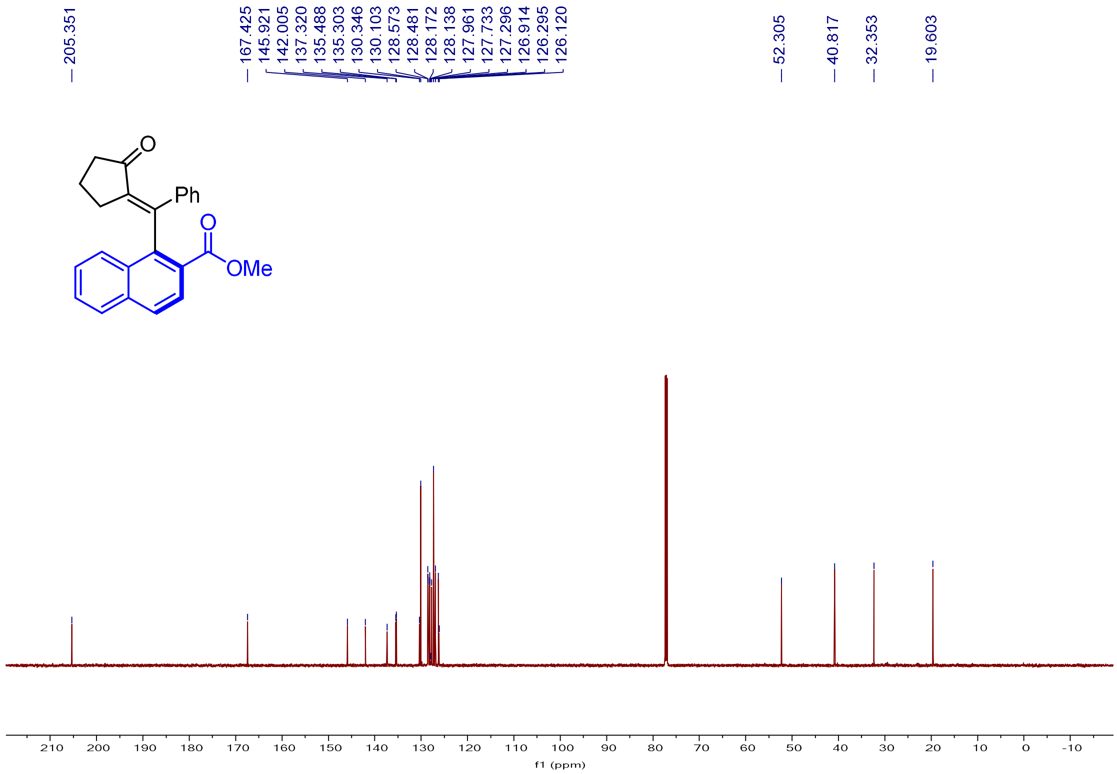

^{1}H NMR (600 MHz, Chloroform-d) spectrum of 61

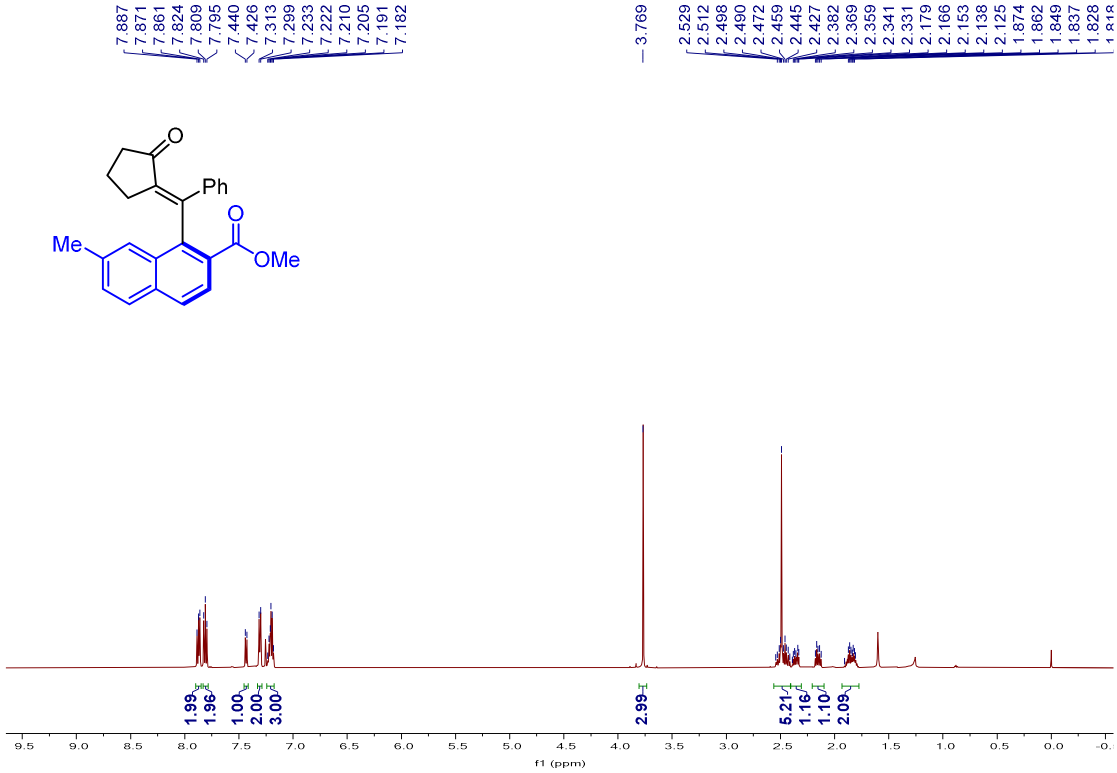

^{13}C NMR (150 MHz, Chloroform-d) spectrum of 61

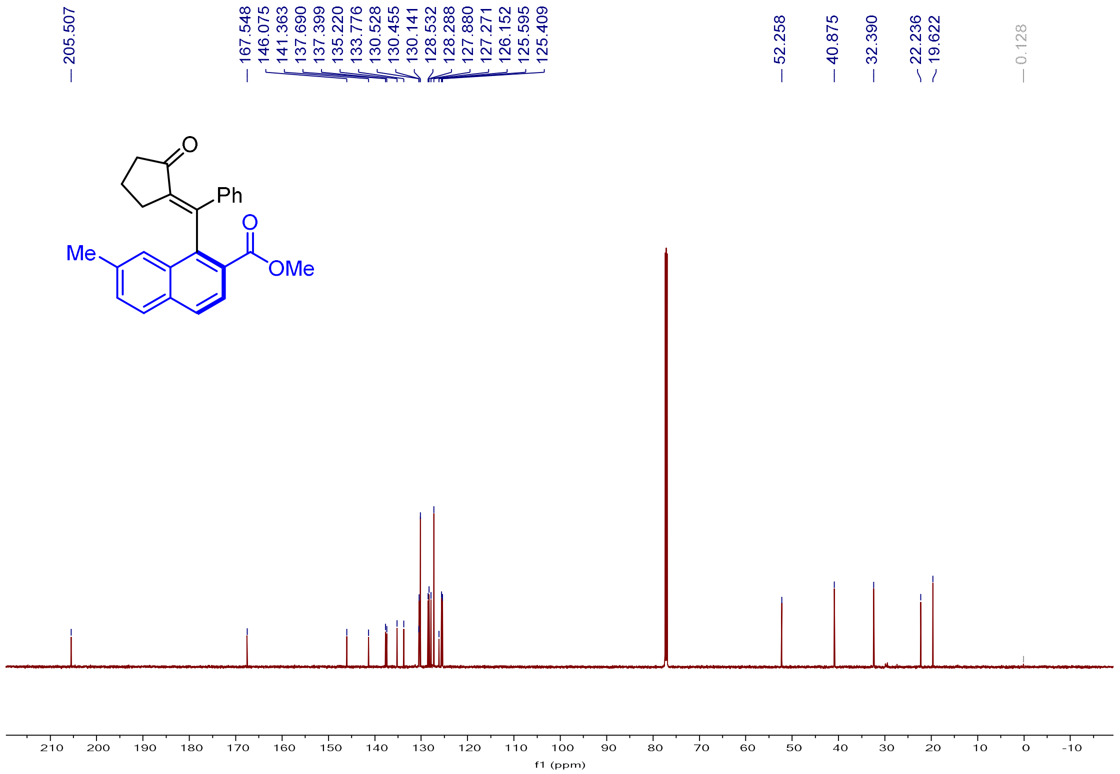

^{1}H NMR (600 MHz, Chloroform-d) spectrum of 62

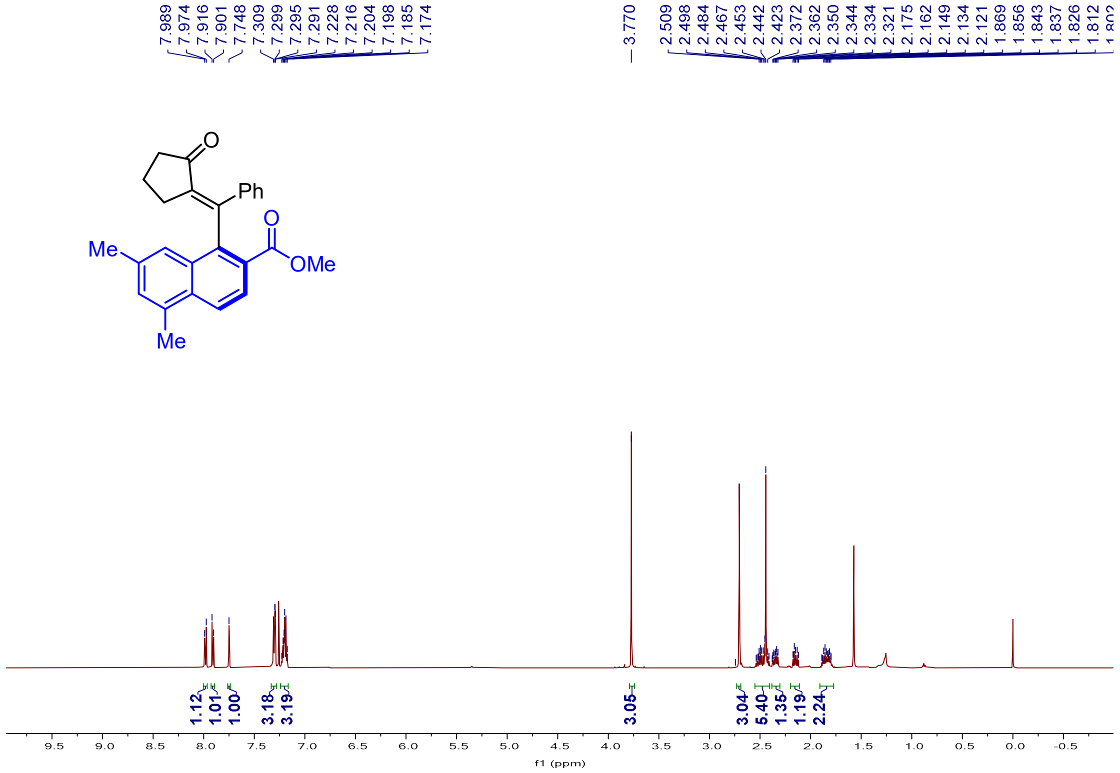

^{13}C NMR (150 MHz, Chloroform-d) spectrum of 62

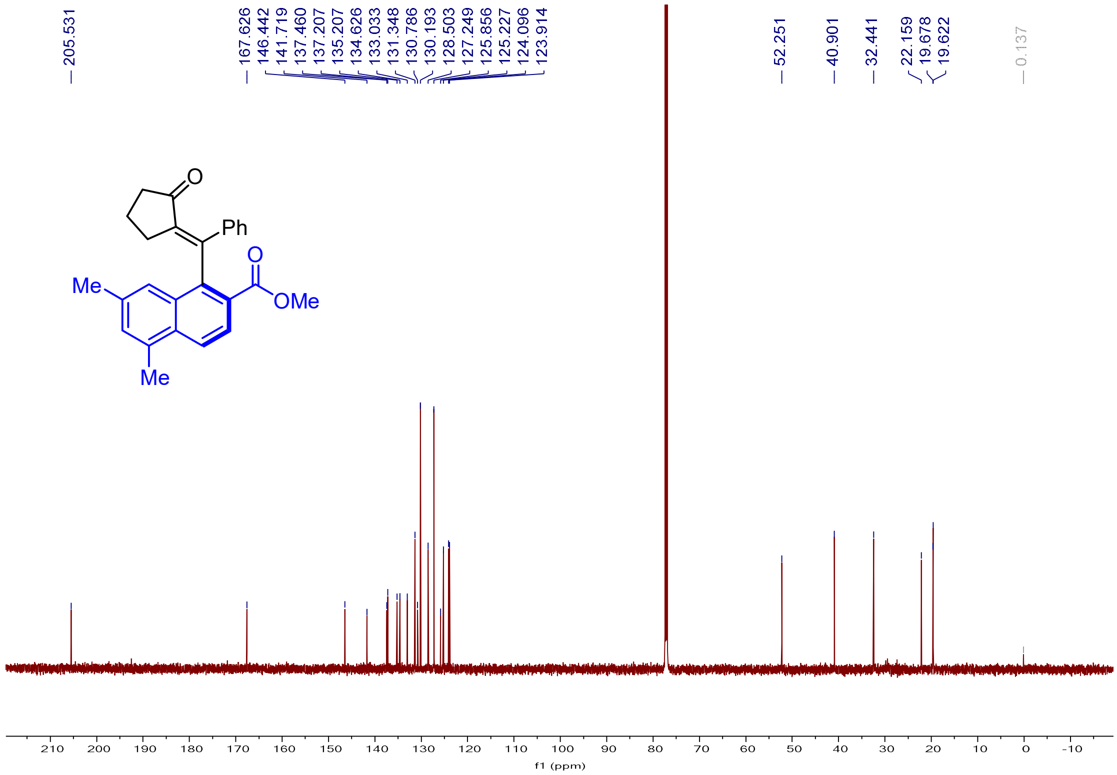

^{1}H NMR (600 MHz, Chloroform-d) spectrum of 63

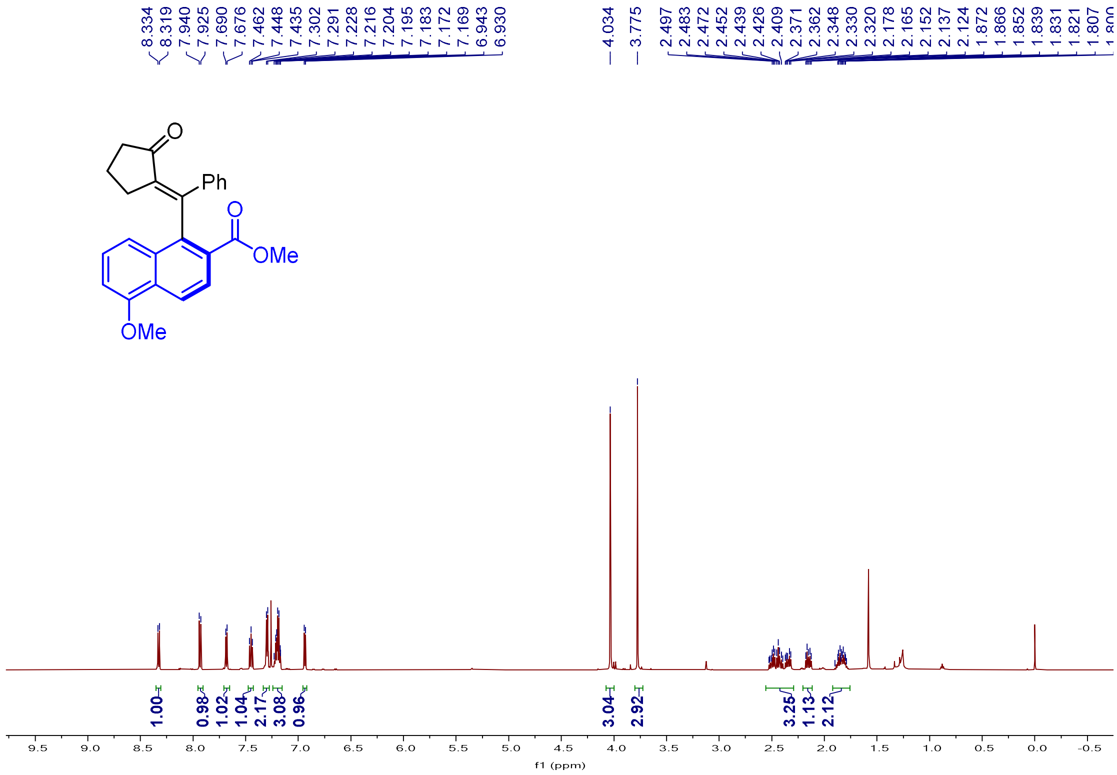

^{13}C NMR (150 MHz, Chloroform-d) spectrum of 63

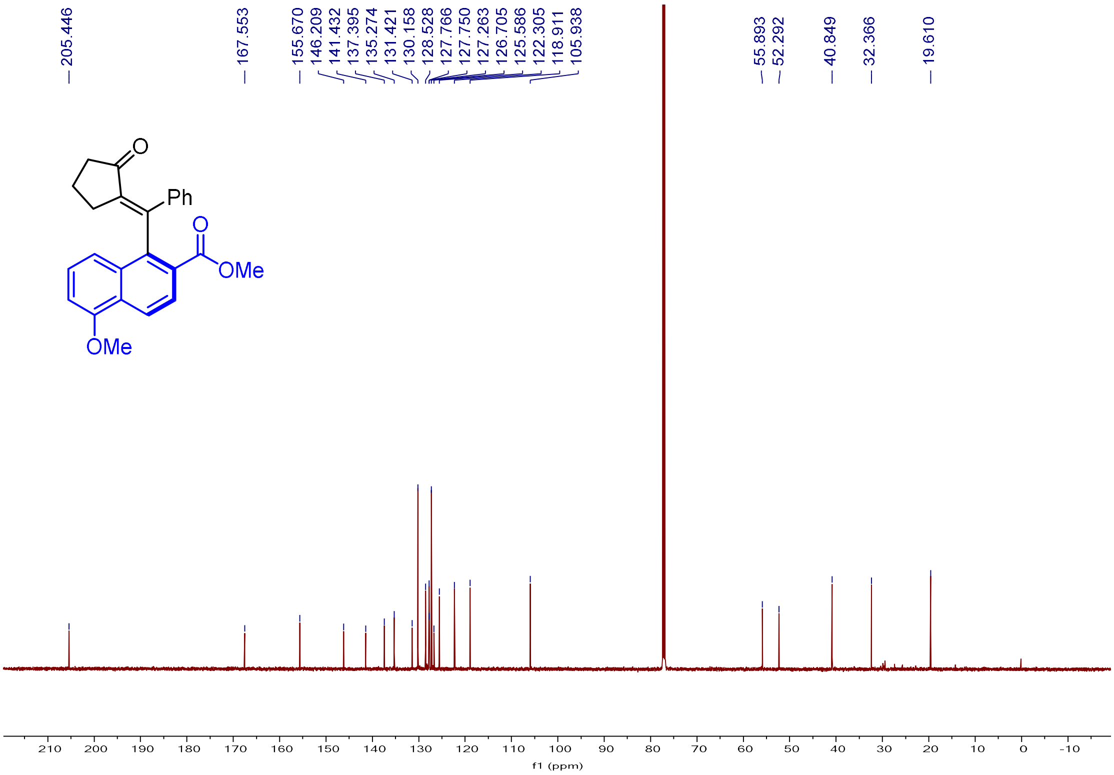

^{1}H NMR (600 MHz, Chloroform-d) spectrum of 64

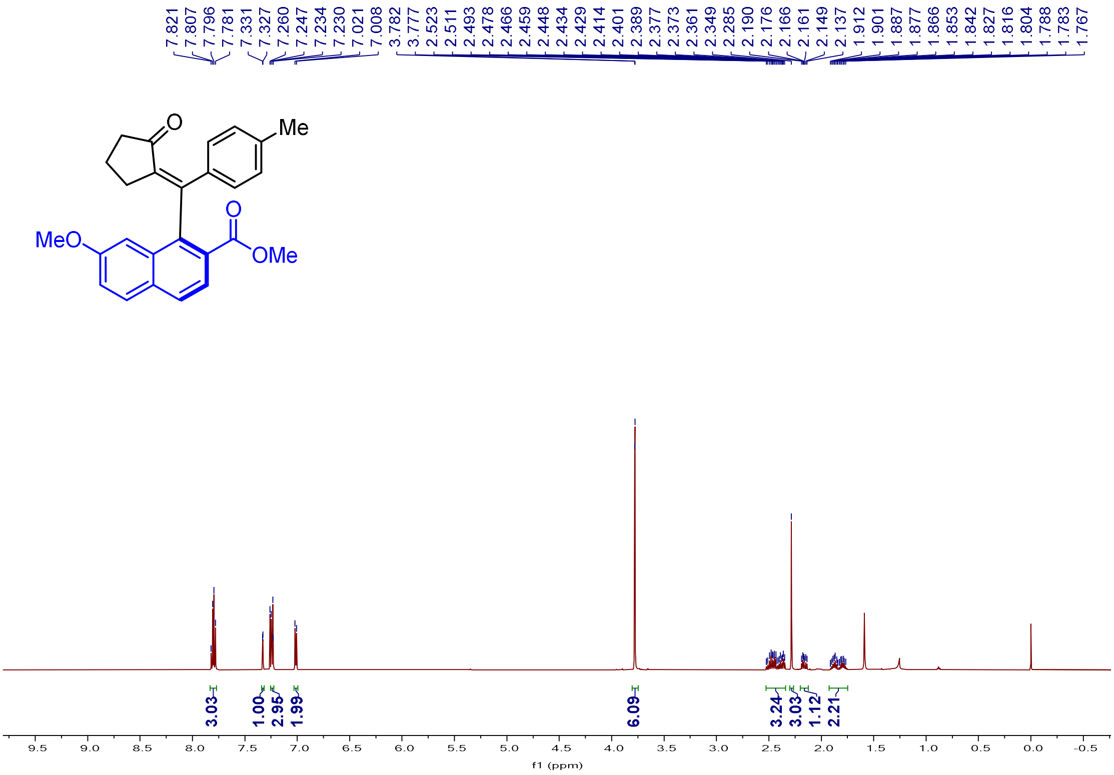

^{13}C NMR (150 MHz, Chloroform-d) spectrum of 64

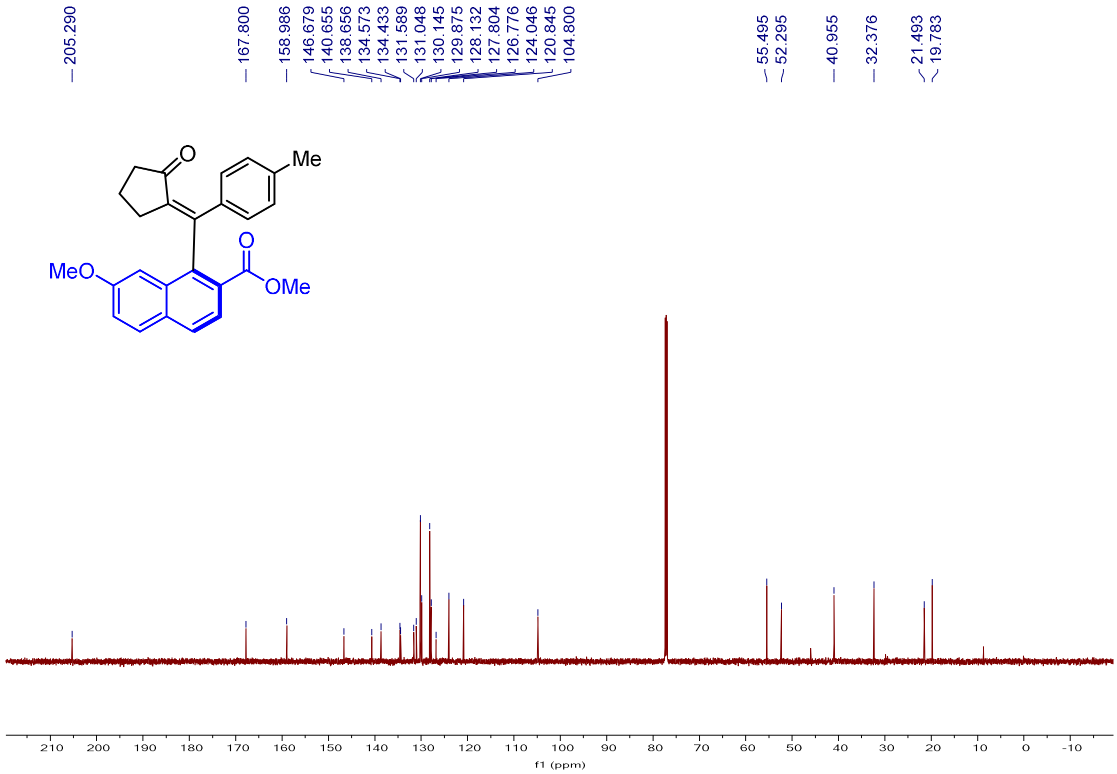

^{1}H NMR (600 MHz, Chloroform-d) spectrum of 65

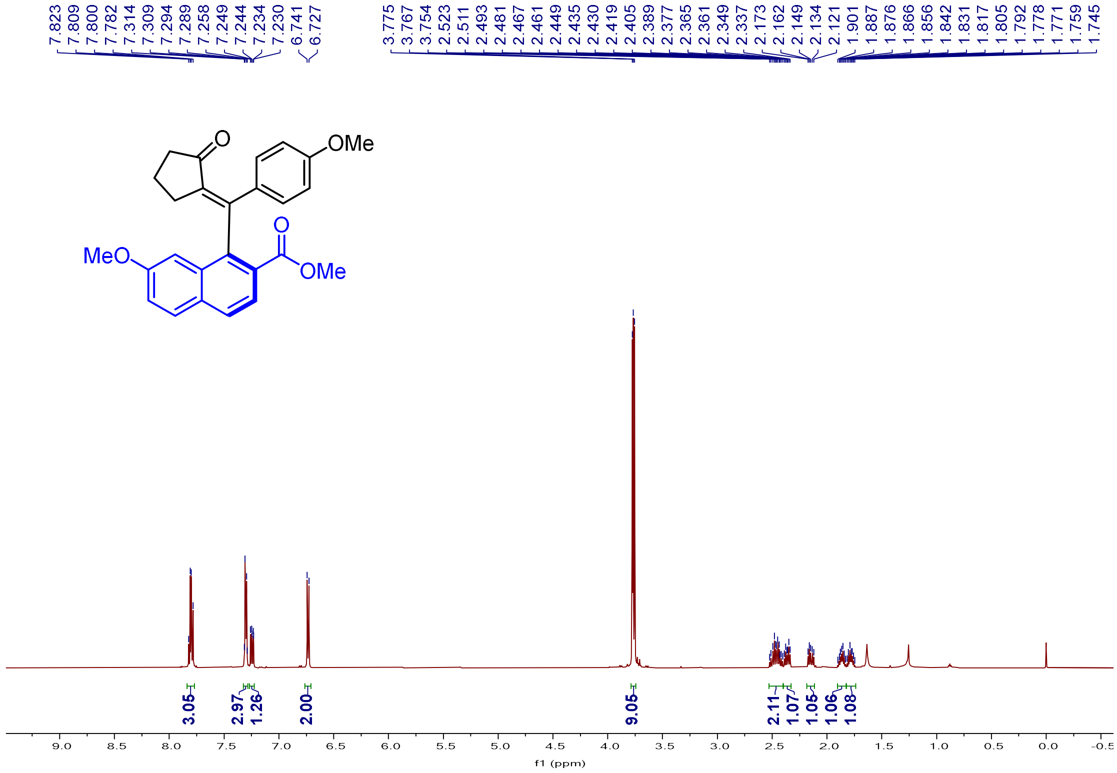

^{13}C NMR (150 MHz, Chloroform-d) spectrum of 65

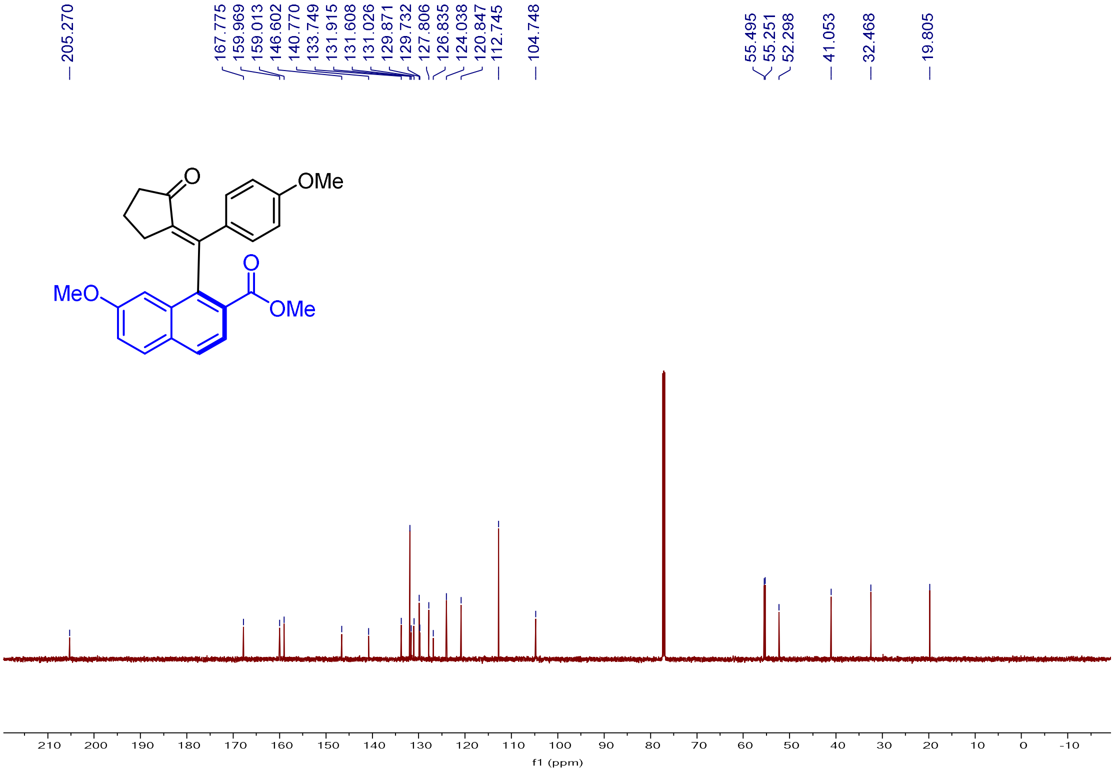

^{1}H NMR (600 MHz, Chloroform-d) spectrum of 66

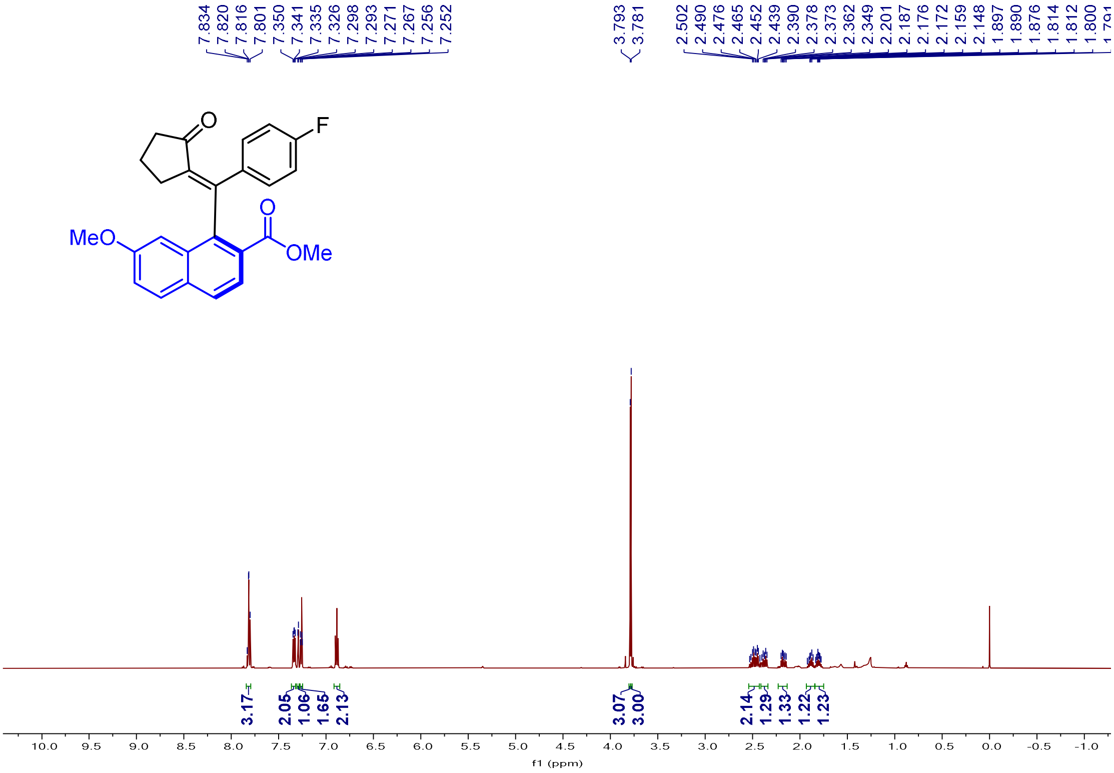

^{13}C NMR (150 MHz, Chloroform-d) spectrum of 66

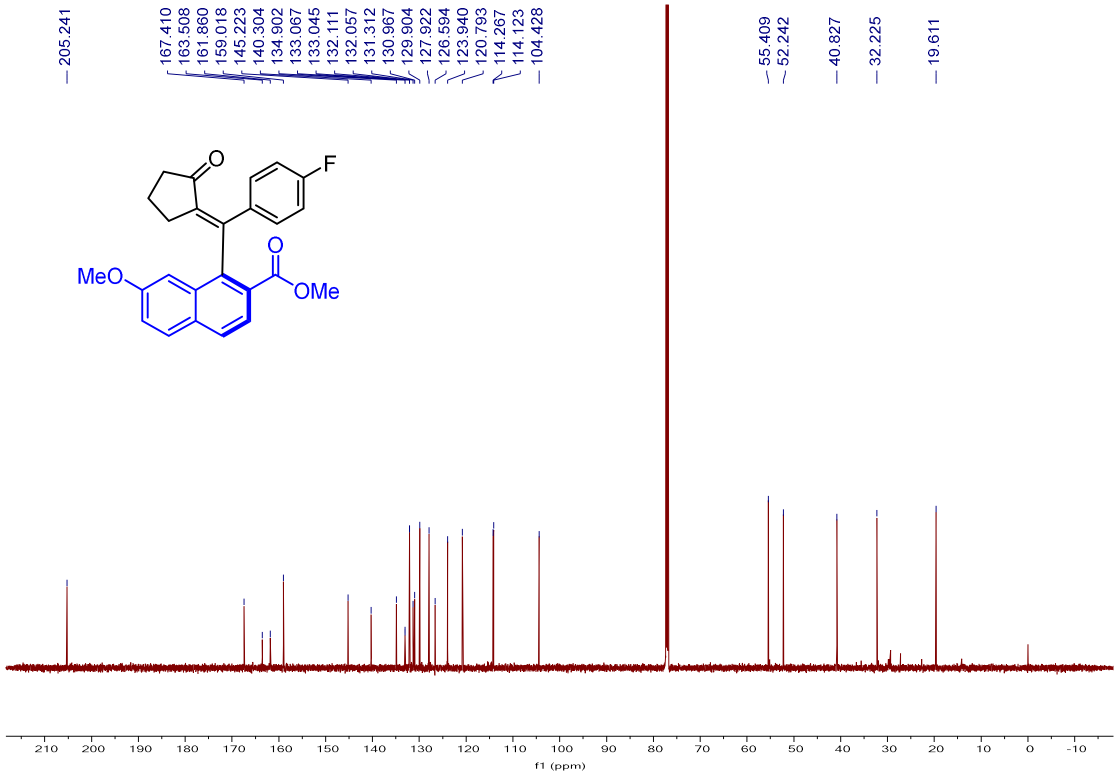

^{19}F NMR (376 MHz, Chloroform-d) spectrum of 66

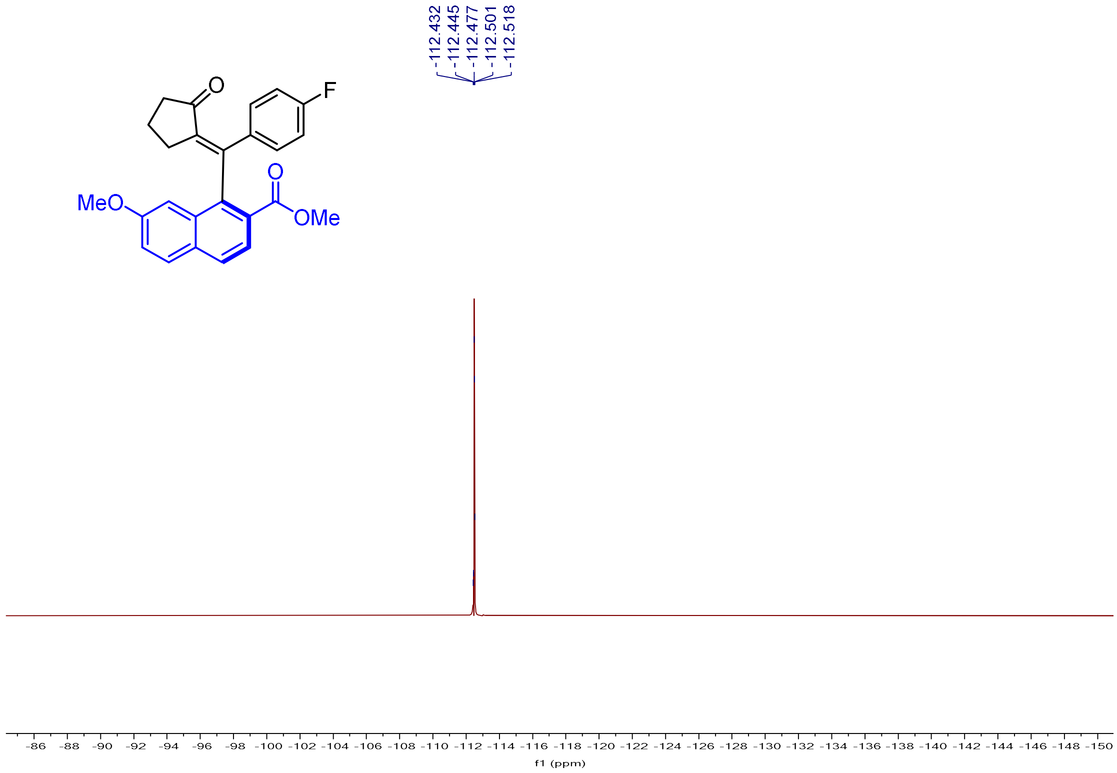

^{1}H NMR (600 MHz, Chloroform-d) spectrum of 67

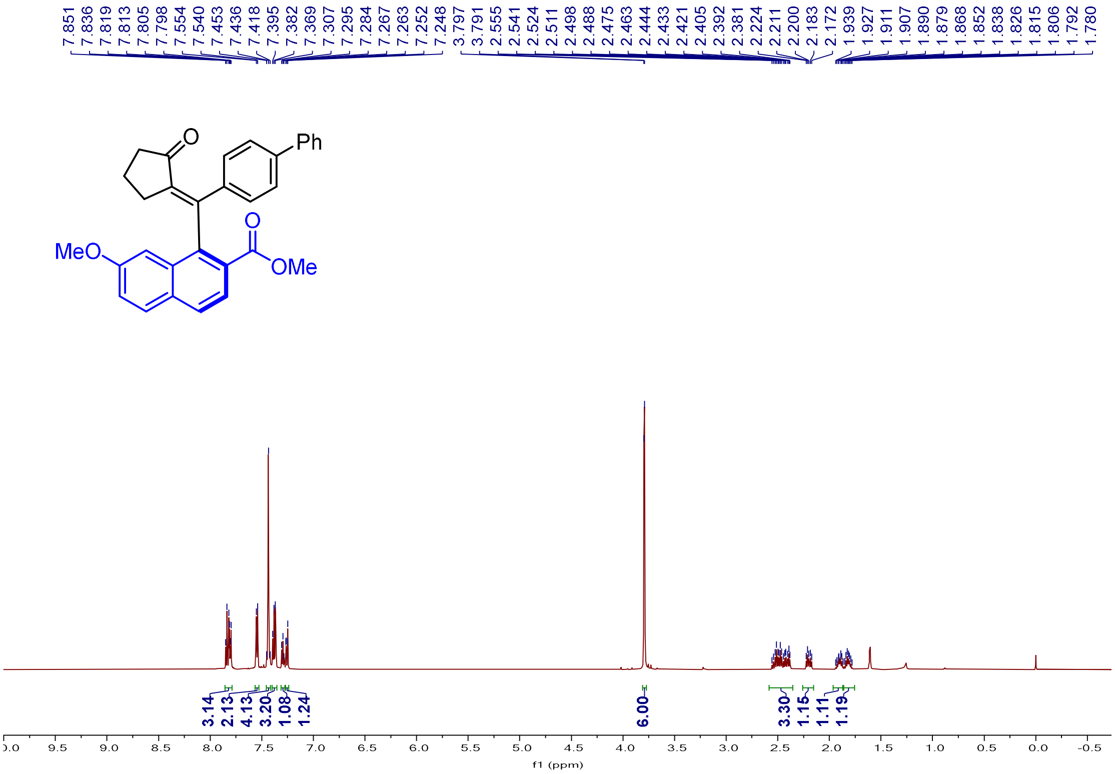

^{13}C NMR (150 MHz, Chloroform-d) spectrum of 67

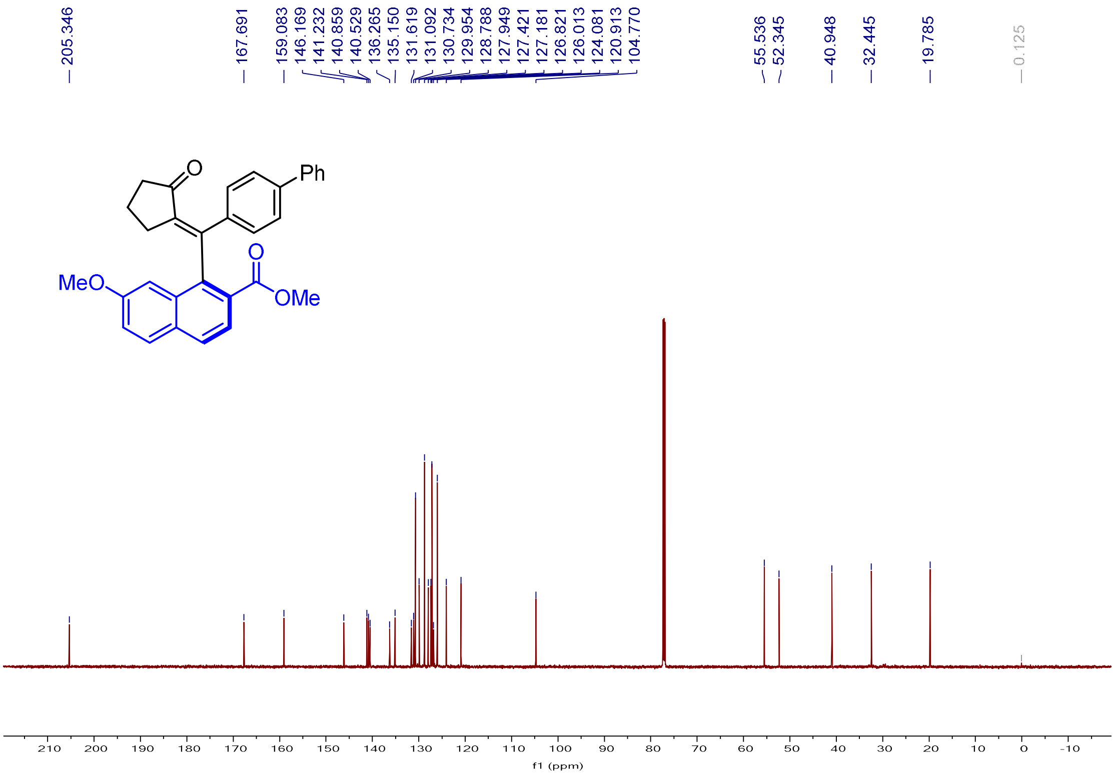

^{1}H NMR (600 MHz, Chloroform-d) spectrum of 68

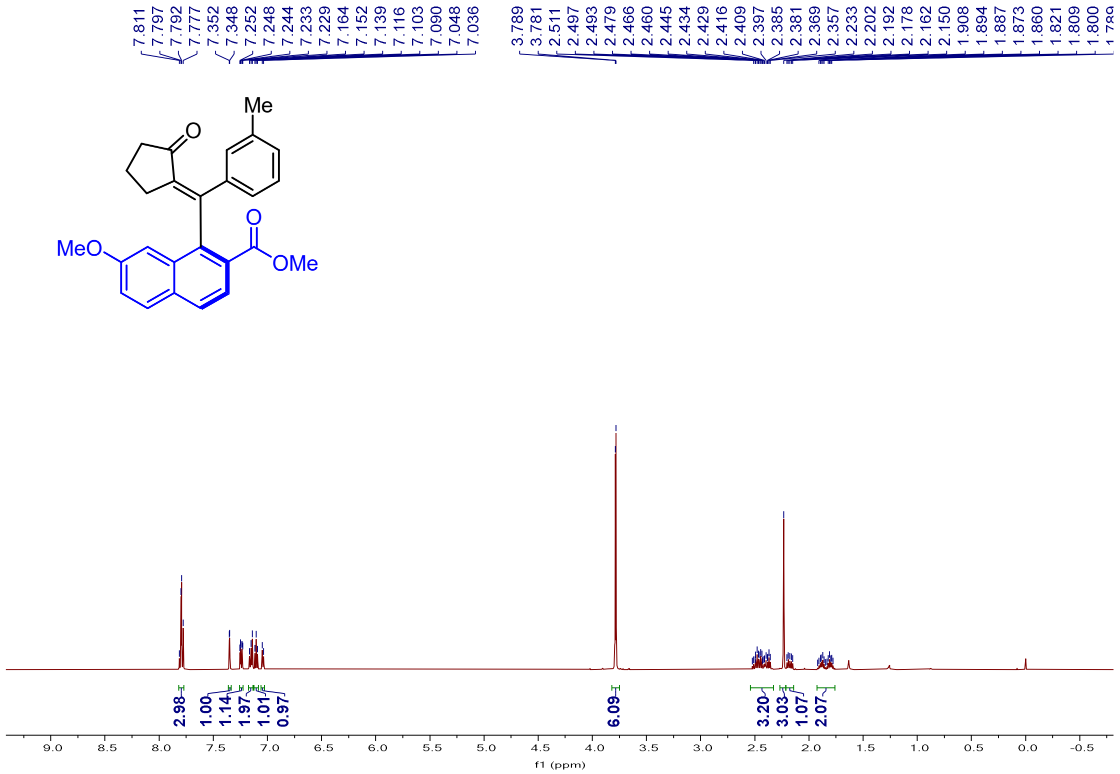

^{13}C NMR (150 MHz, Chloroform-d) spectrum of 68

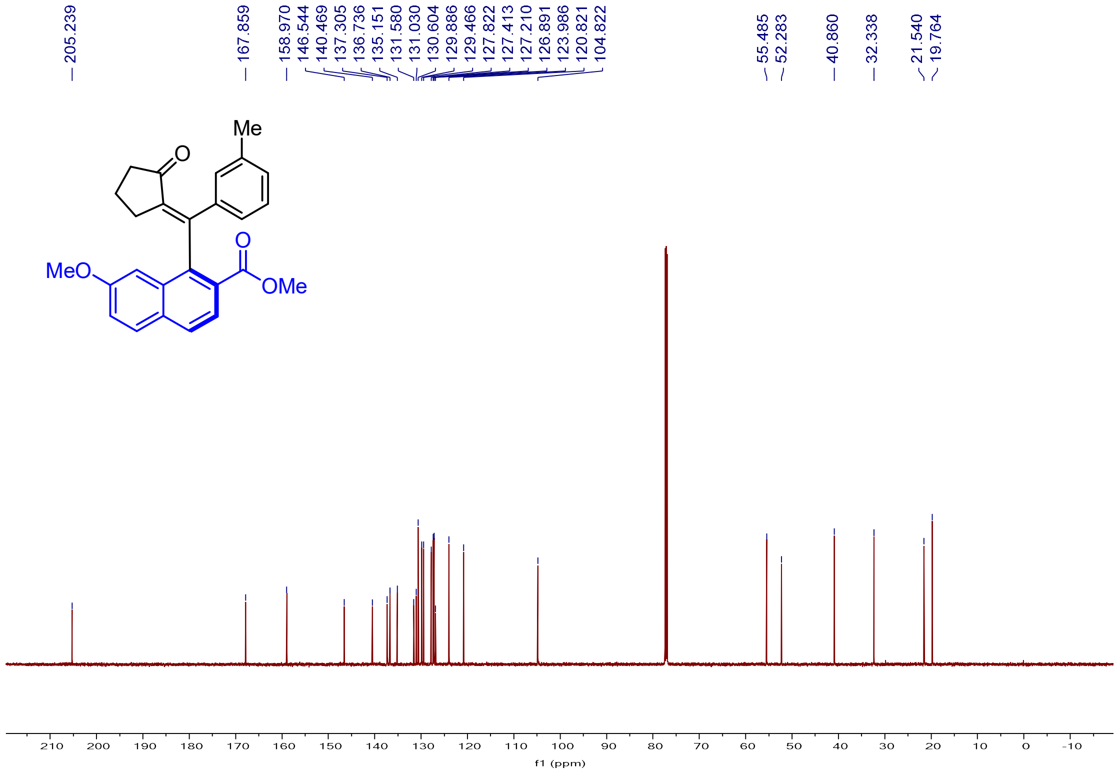

^{1}H NMR (600 MHz, Chloroform-d) spectrum of 69

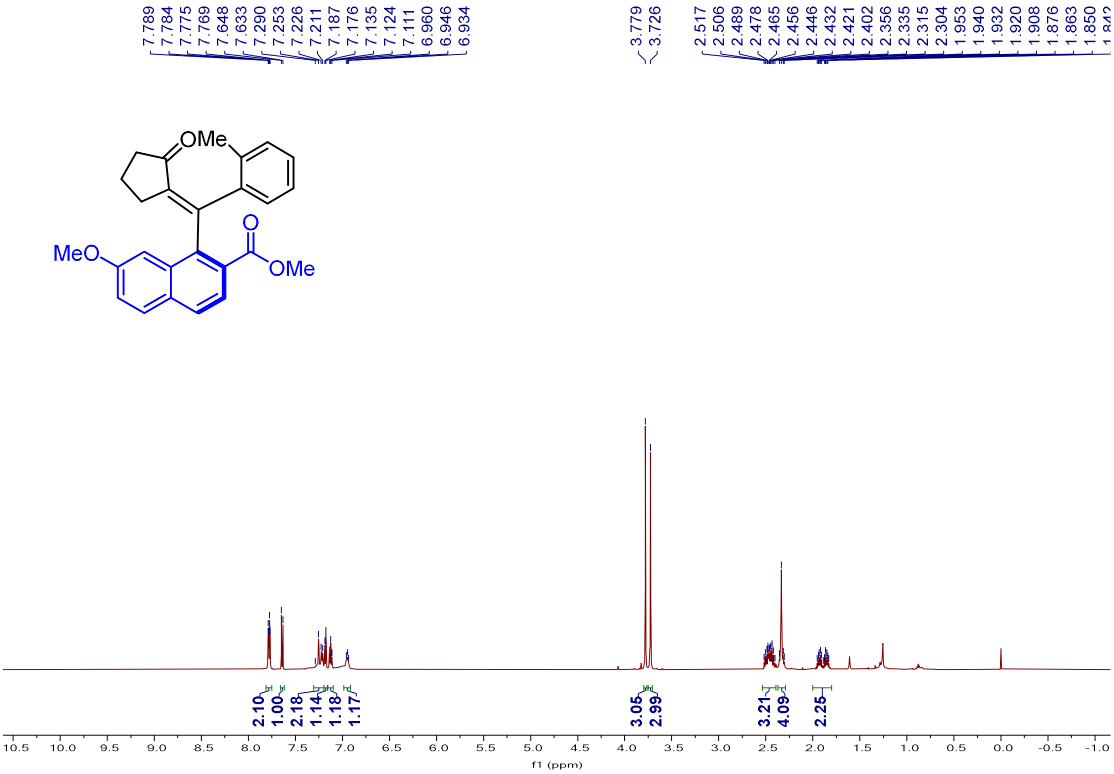

^{13}C NMR (150 MHz, Chloroform-d) spectrum of 69

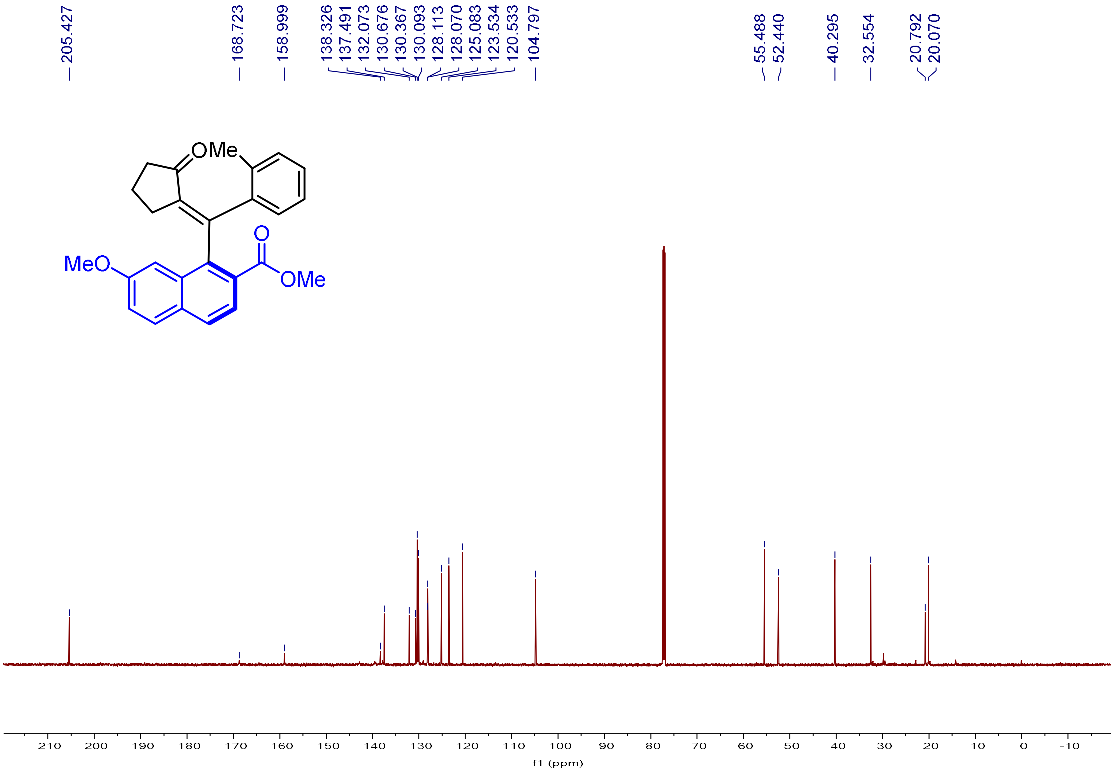

^{1}H NMR (600 MHz, Chloroform-d) spectrum of 70

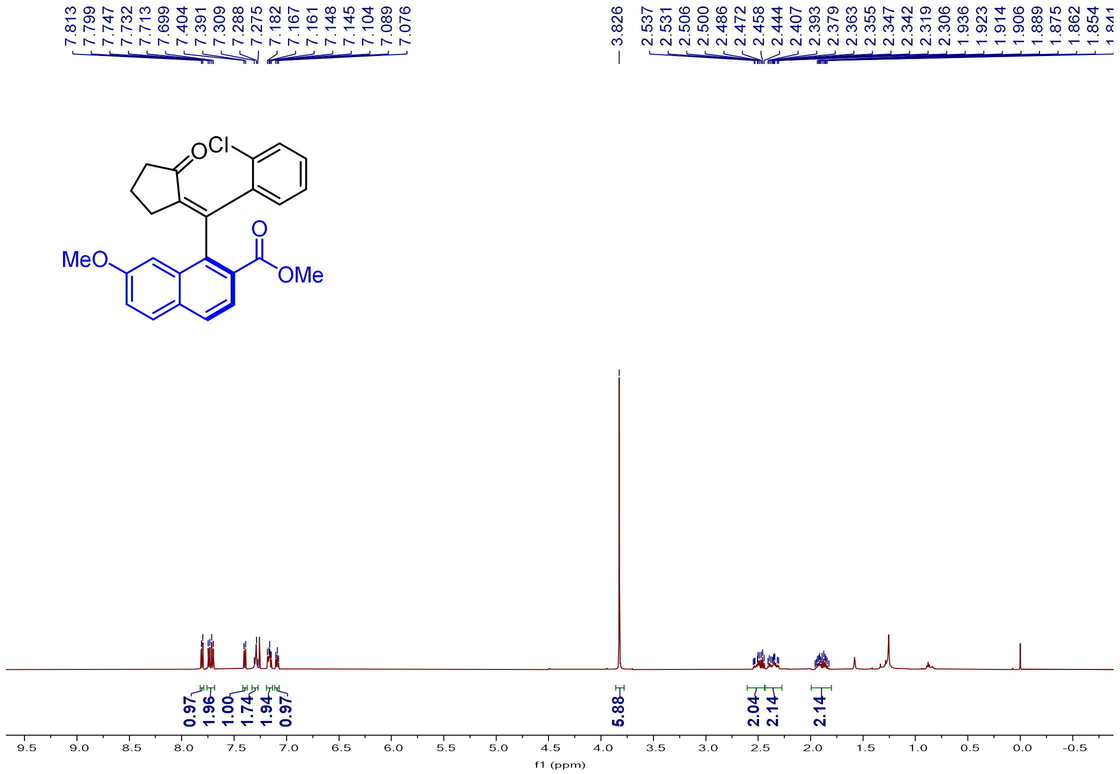

^{13}C NMR (150 MHz, Chloroform-d) spectrum of 70

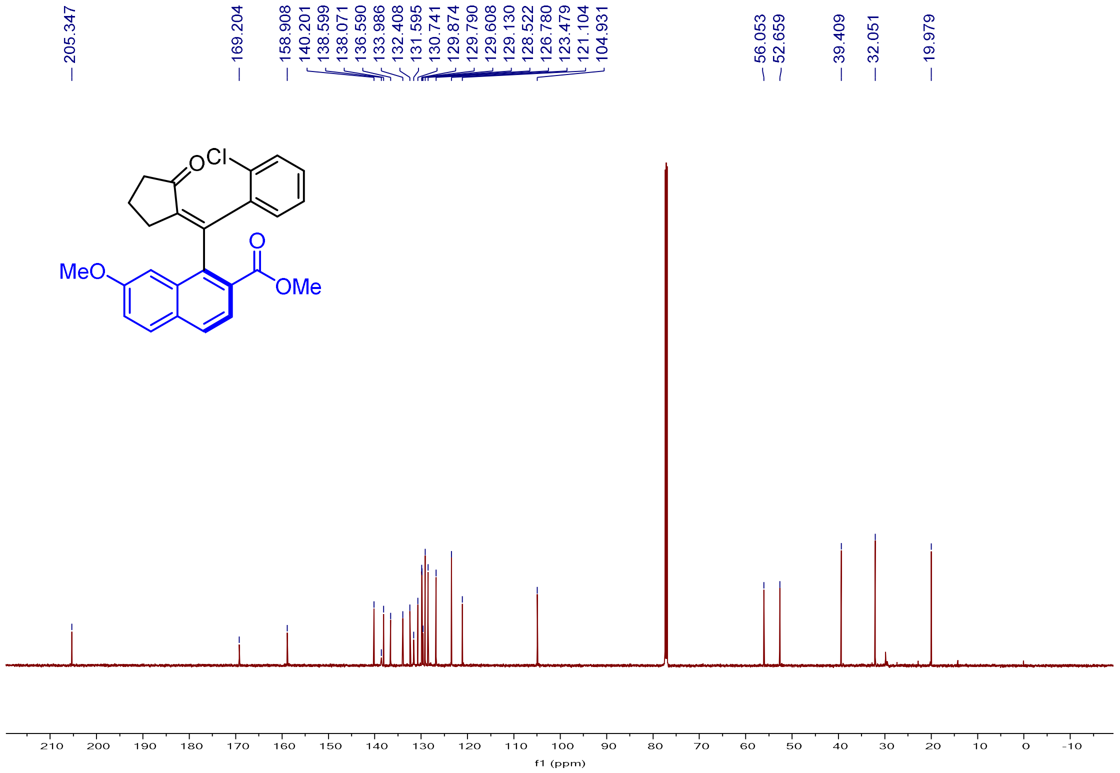

^{1}H NMR (600 MHz, Chloroform-d) spectrum of 71

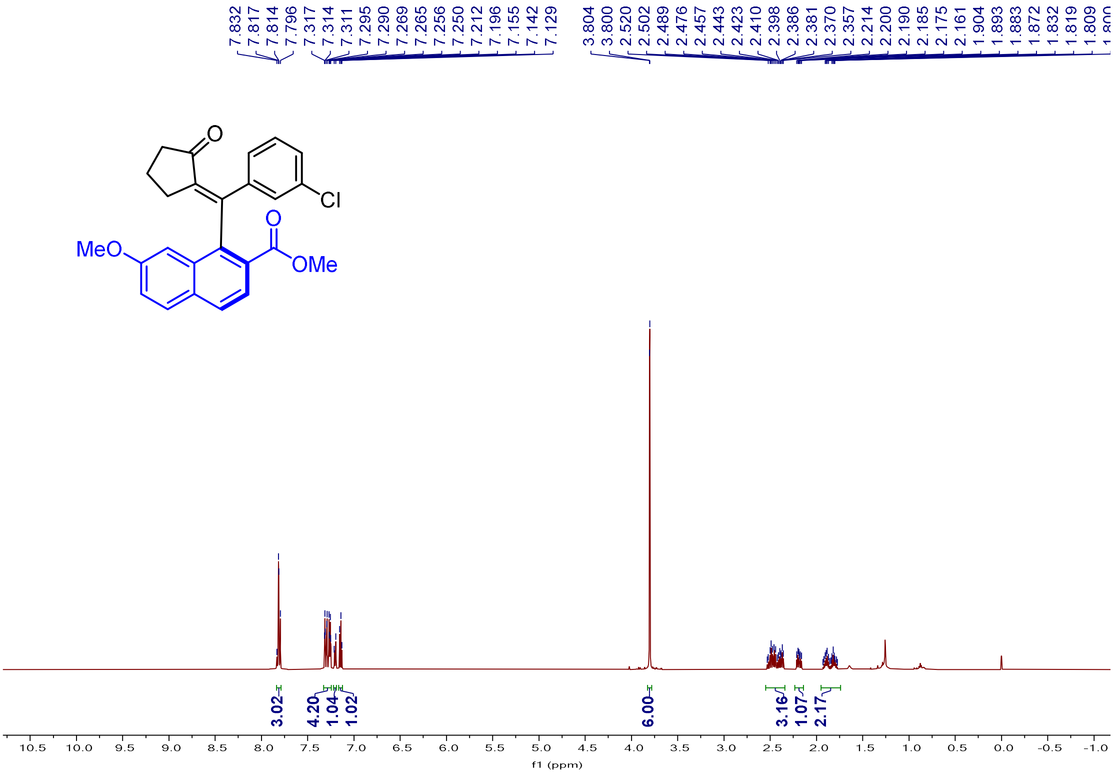

^{13}C NMR (150 MHz, Chloroform-d) spectrum of 71

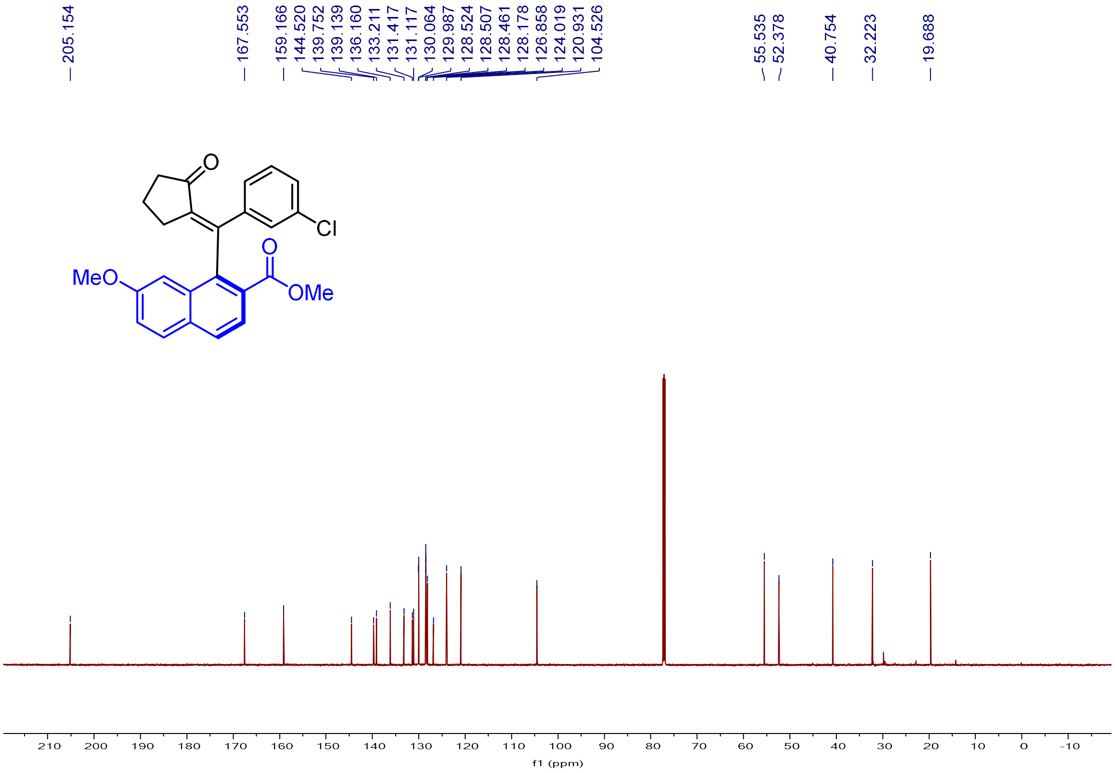

^{1}H NMR (600 MHz, Chloroform-d) spectrum of 72

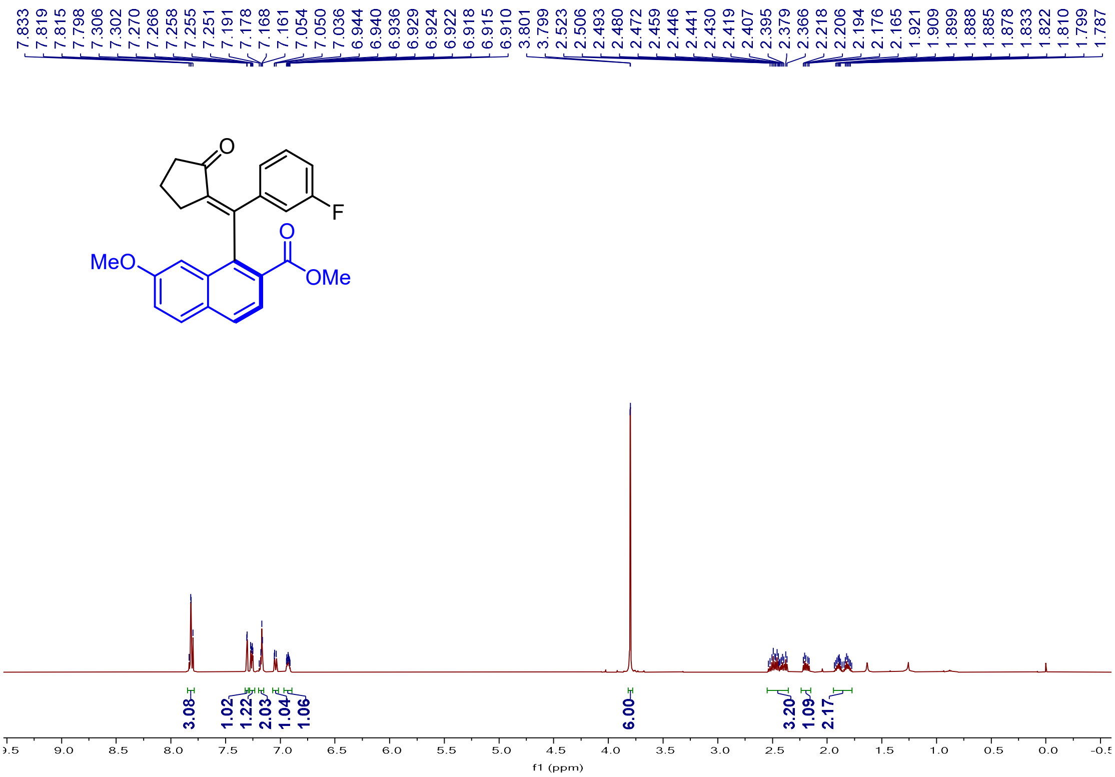

^{13}C NMR (150 MHz, Chloroform-d) spectrum of 72

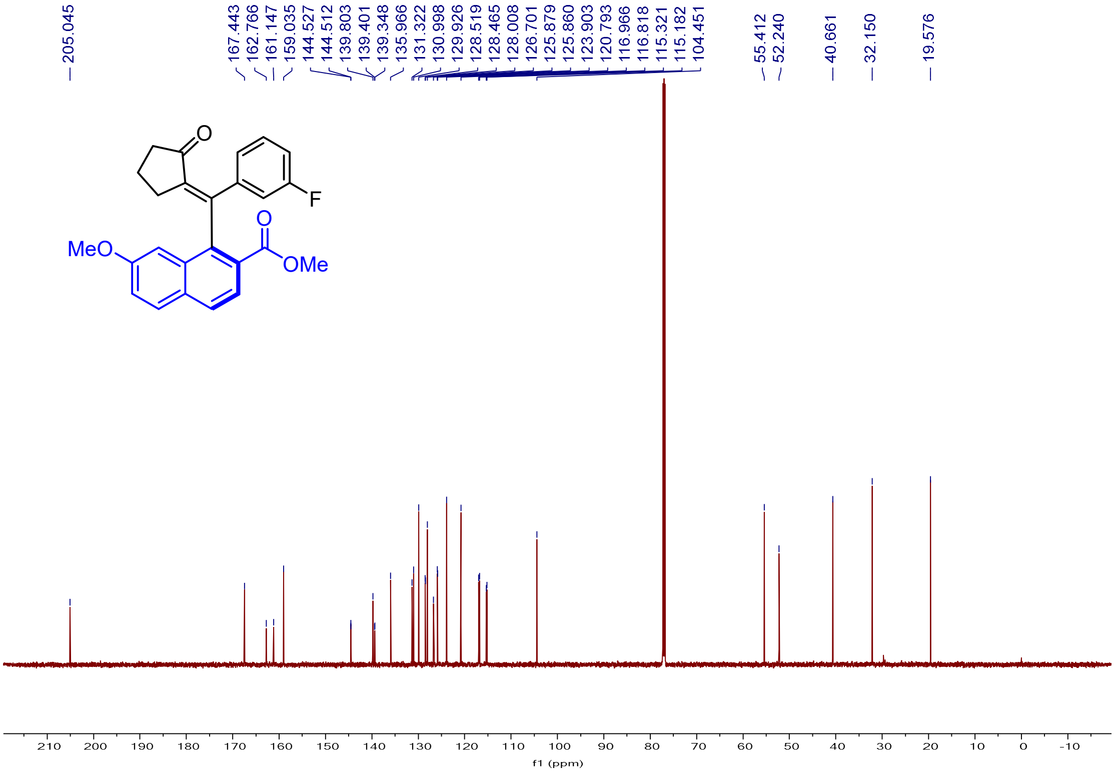

^{19}F NMR (376 MHz, Chloroform-d) spectrum of 72

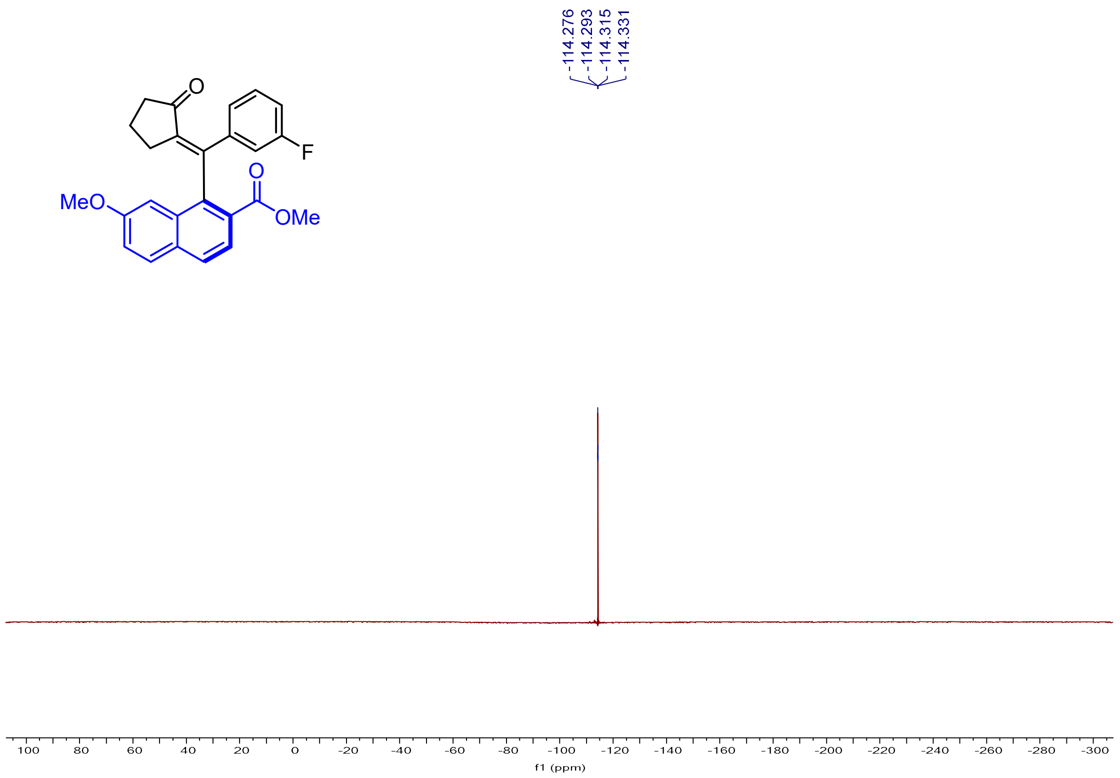

^{1}H NMR (600 MHz, Chloroform-d) spectrum of 73

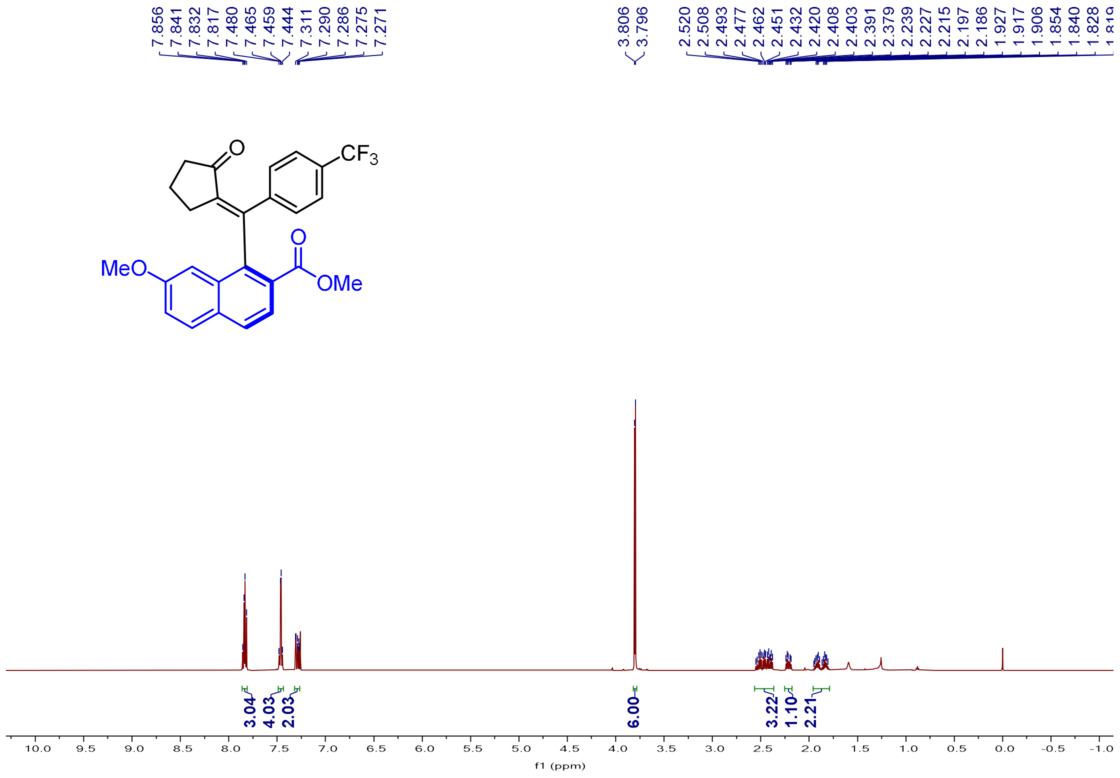

^{13}C NMR (150 MHz, Chloroform-d) spectrum of 73

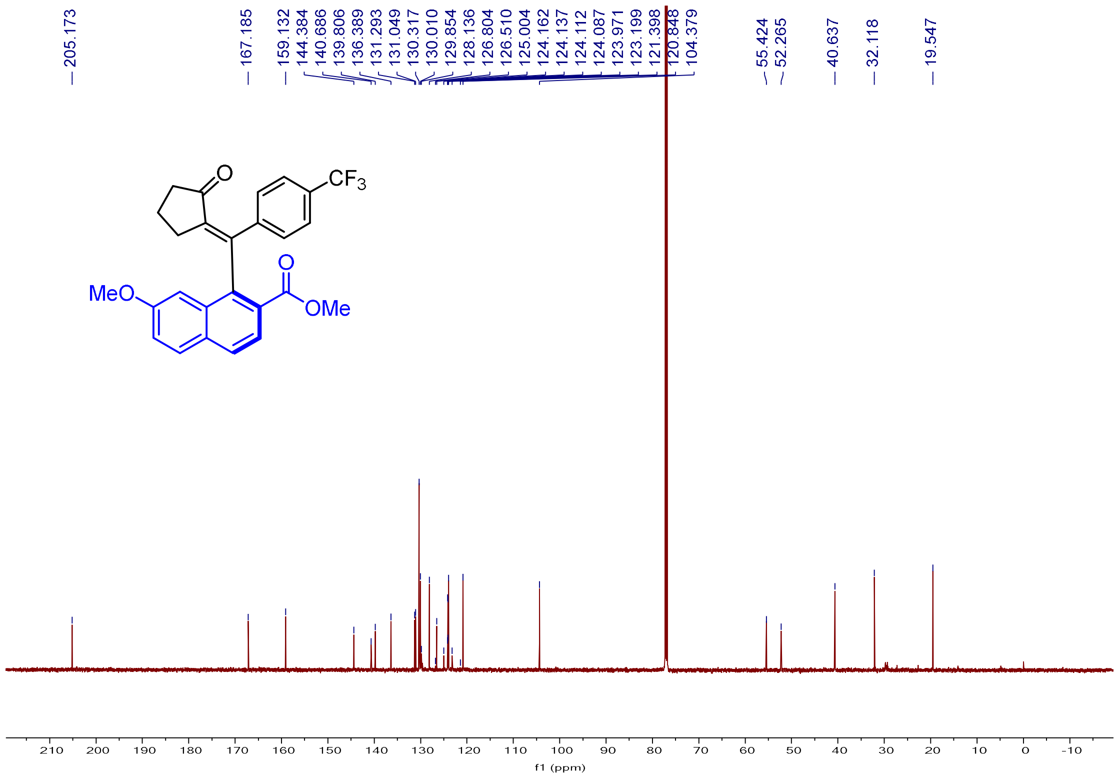

^{19}F NMR (376 MHz, Chloroform-d) spectrum of 73

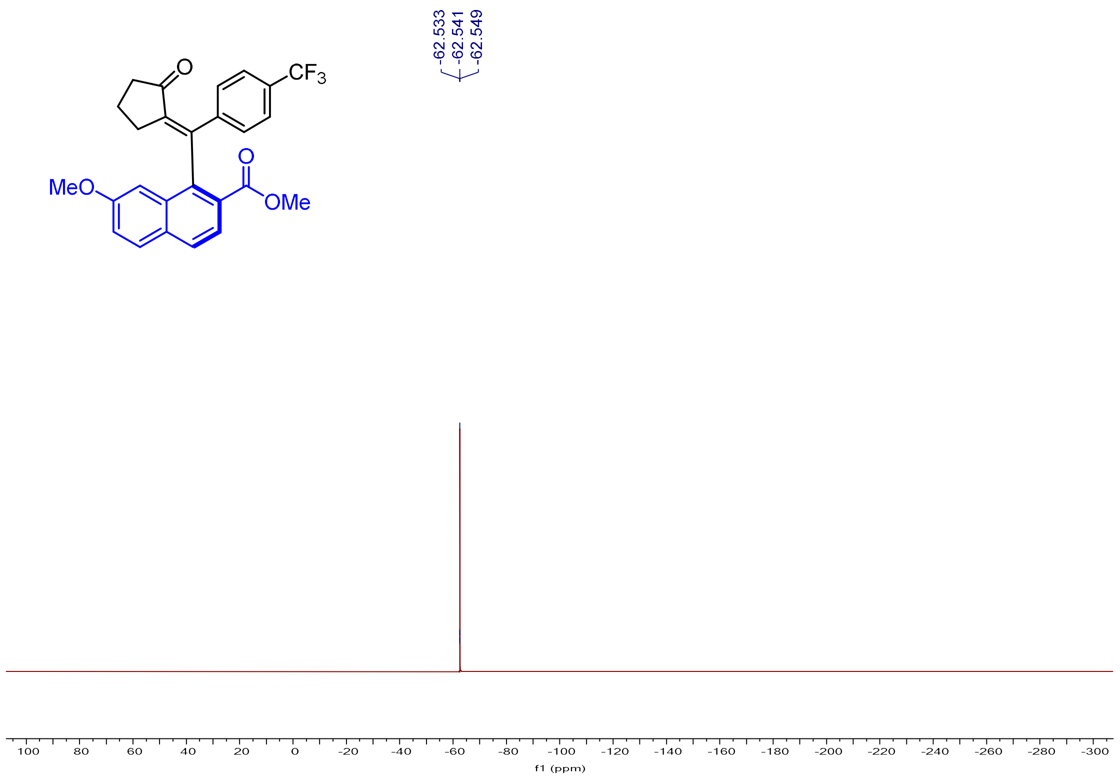

^{1}H NMR (600 MHz, Chloroform-d) spectrum of 74

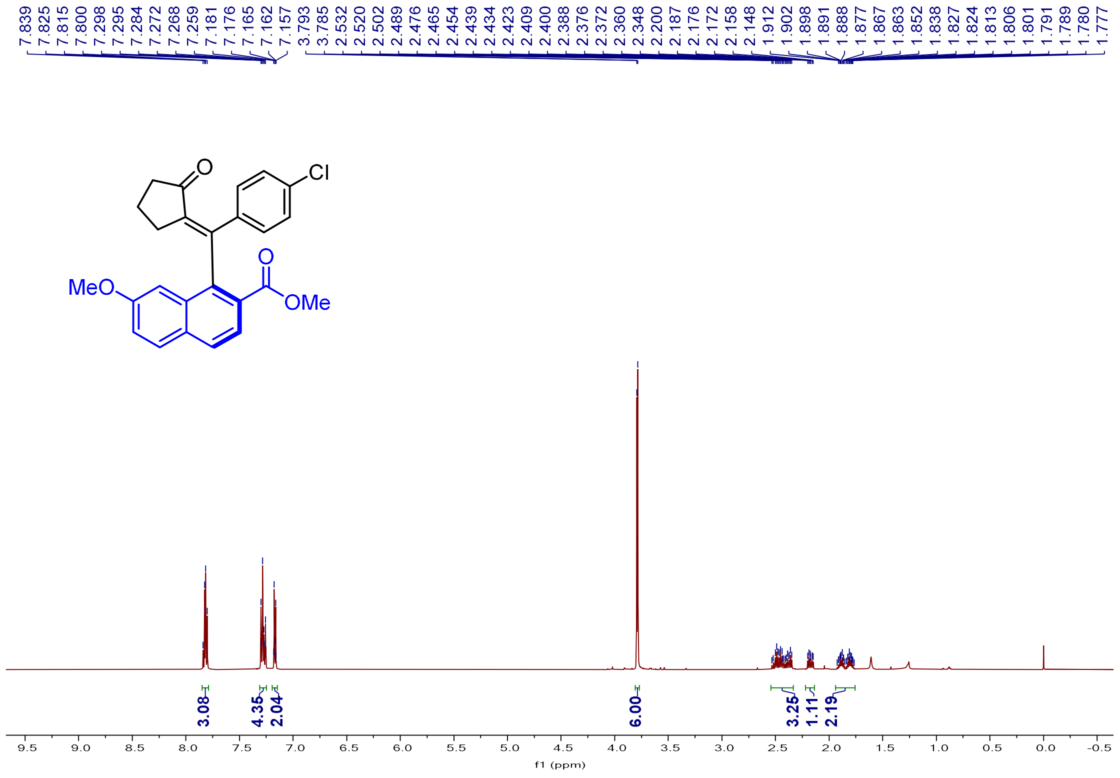

^{13}C NMR (150 MHz, Chloroform-d) spectrum of 74

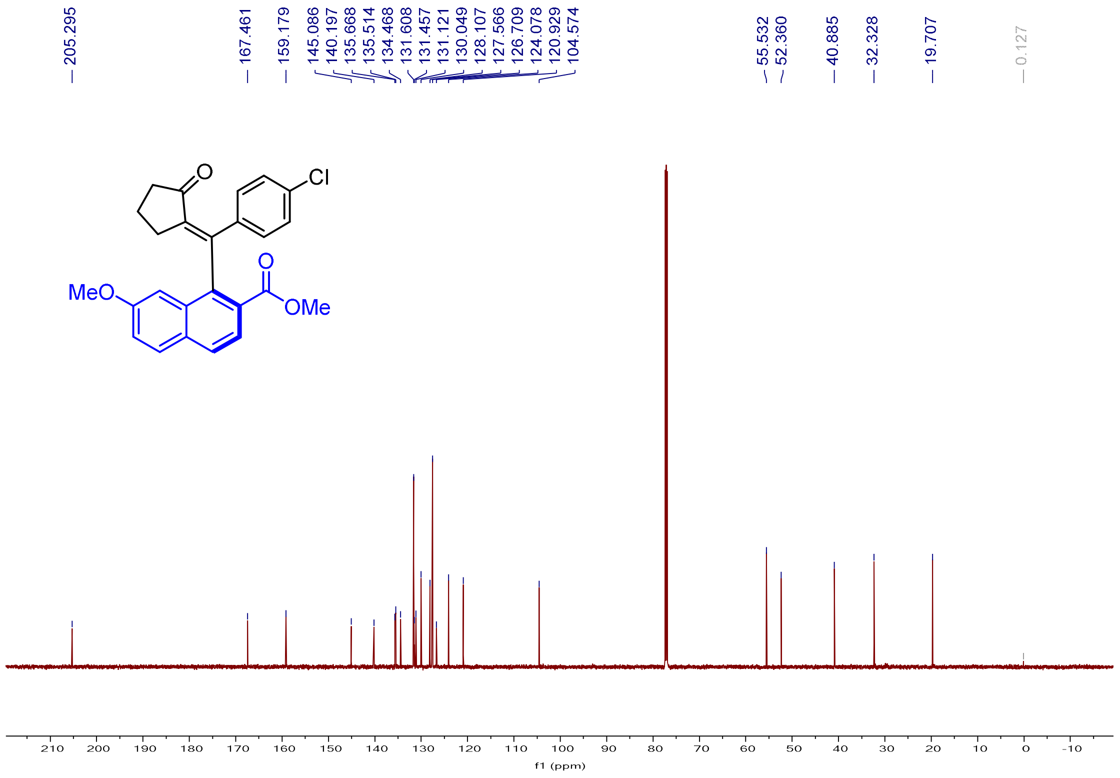

^{1}H NMR (600 MHz, Chloroform-d) spectrum of 75

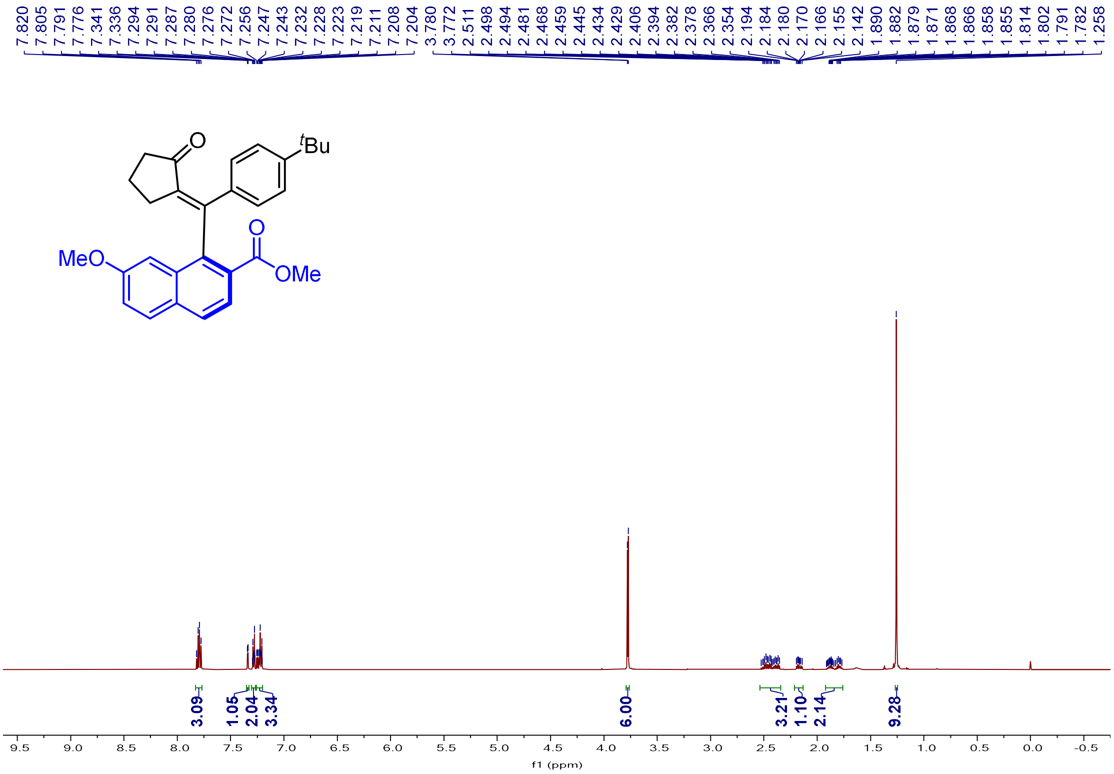

^{13}C NMR (150 MHz, Chloroform-d) spectrum of 75

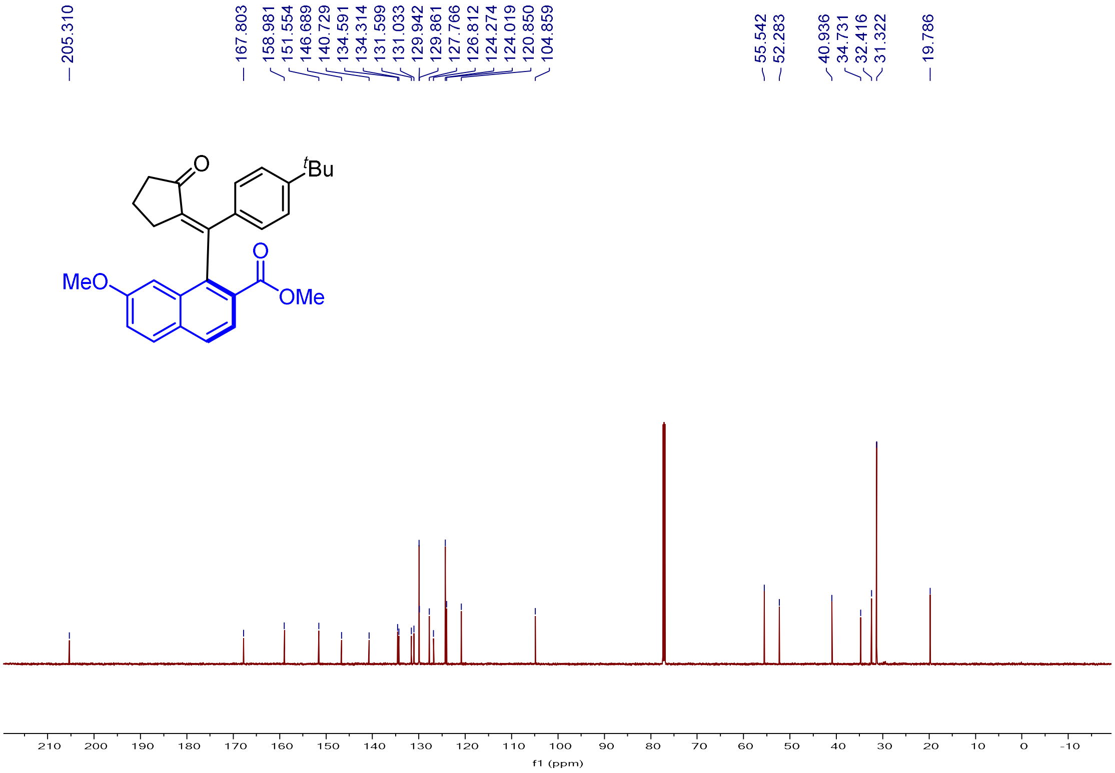

^{1}H NMR (600 MHz, Chloroform-d) spectrum of 76

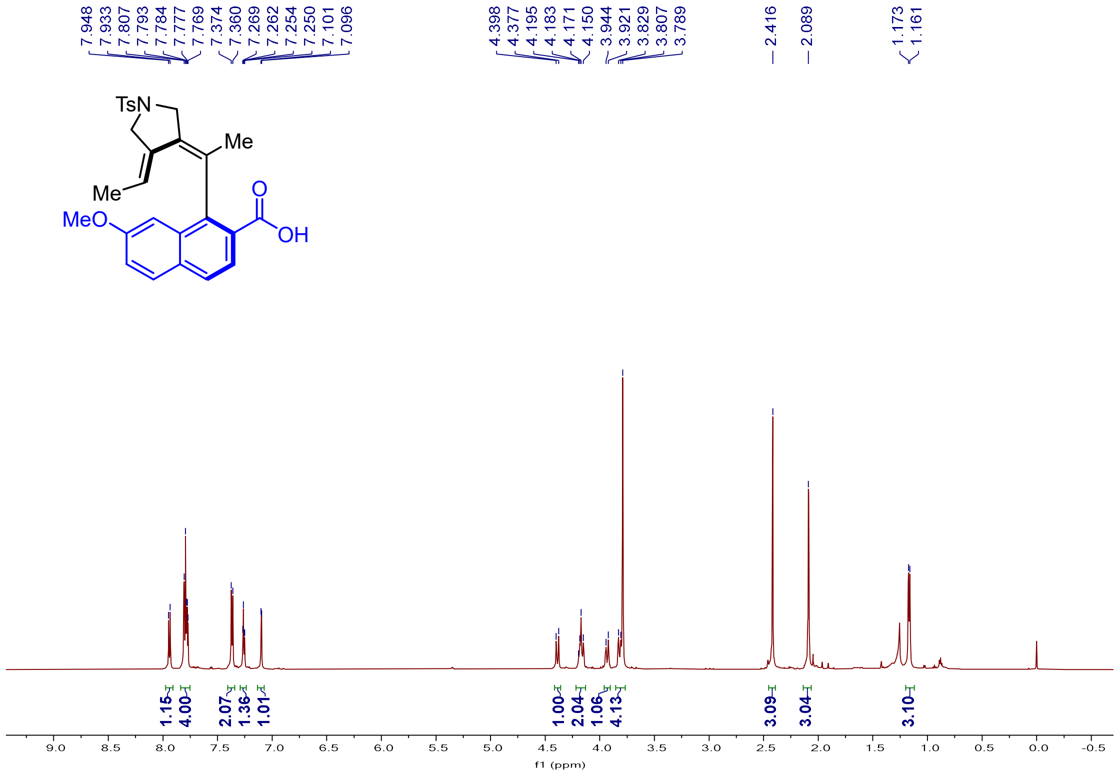

^{13}C NMR (150 MHz, Chloroform-d) spectrum of 76

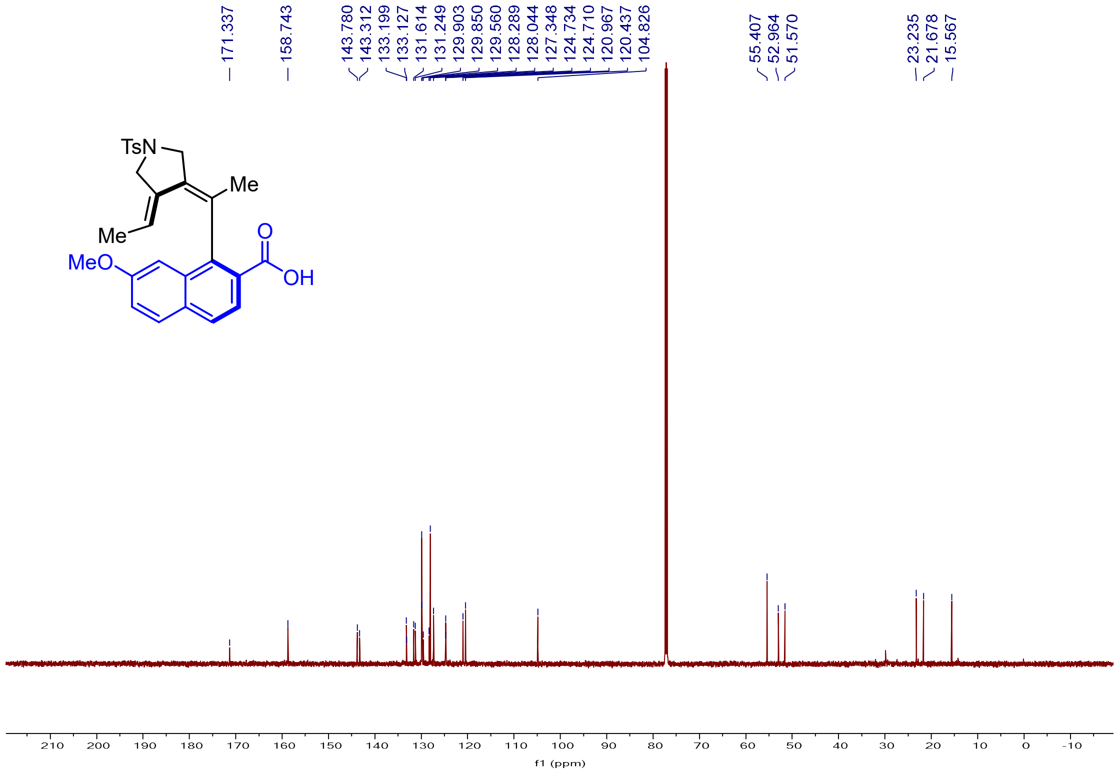

^{1}H NMR (600 MHz, Chloroform-d) spectrum of 77

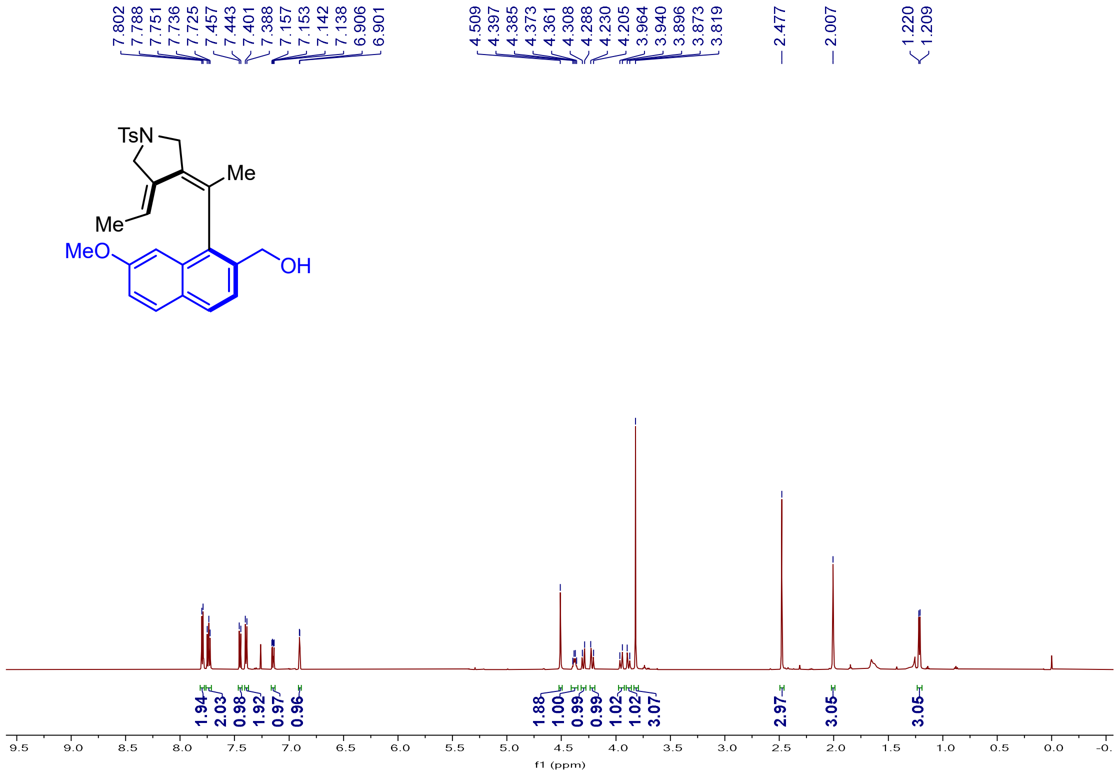

^{13}C NMR (150 MHz, Chloroform-d) spectrum of 77

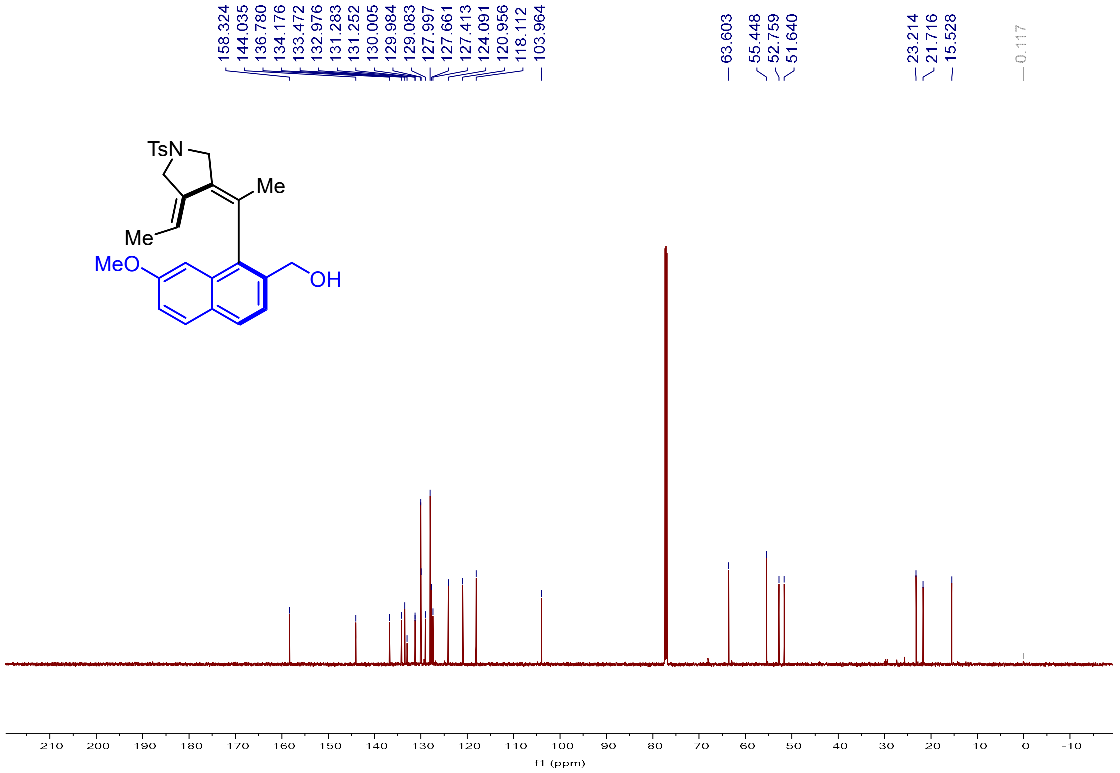

¹H NMR (600 MHz, Chloroform-d) spectrum of 78

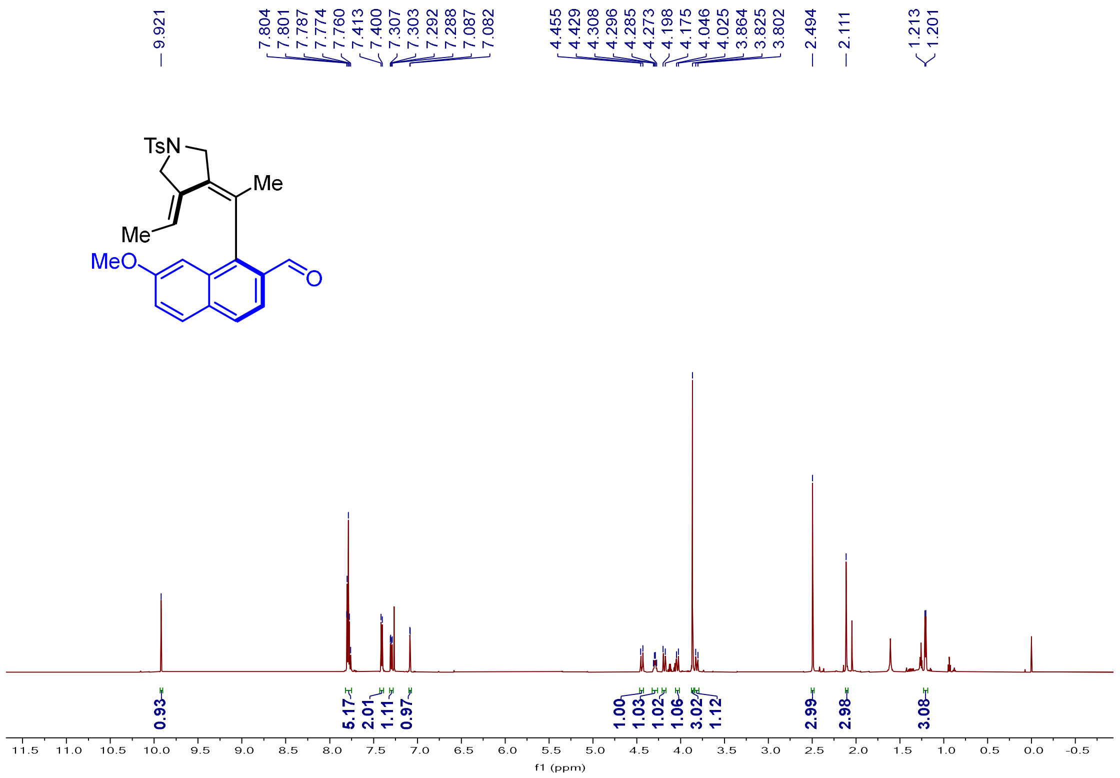

¹³C NMR (150 MHz, Chloroform-d) spectrum of 78

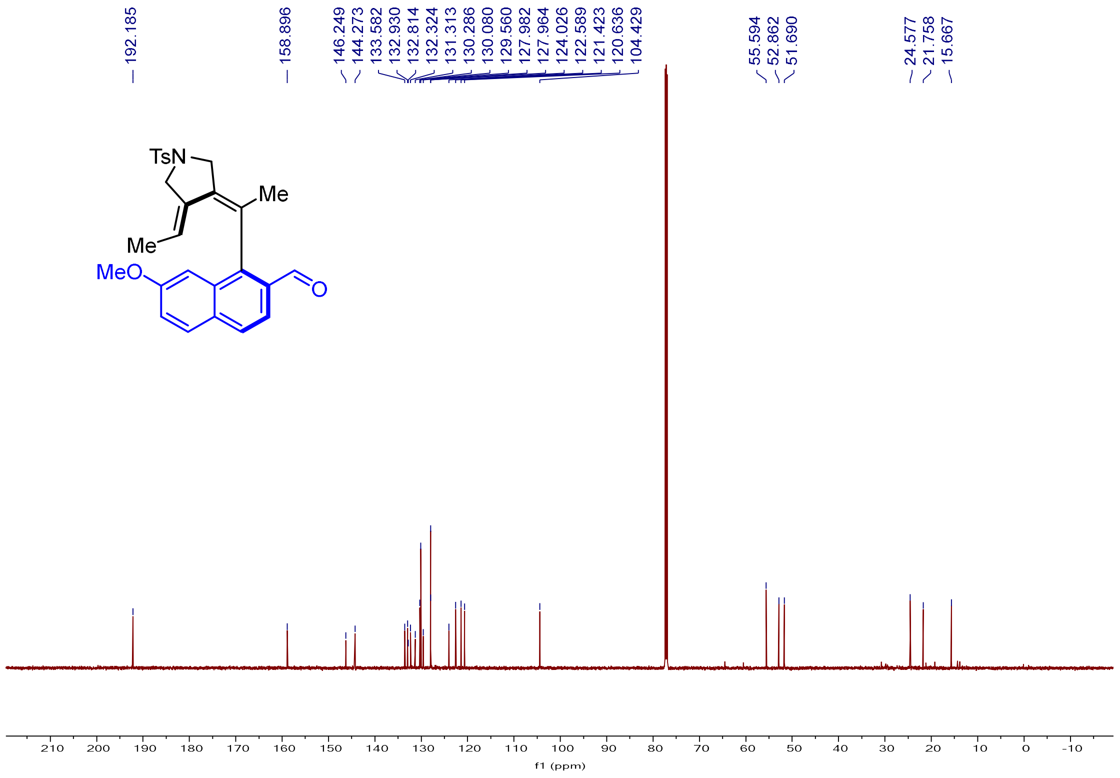

^{1}H NMR (600 MHz, Chloroform-d) spectrum of 79

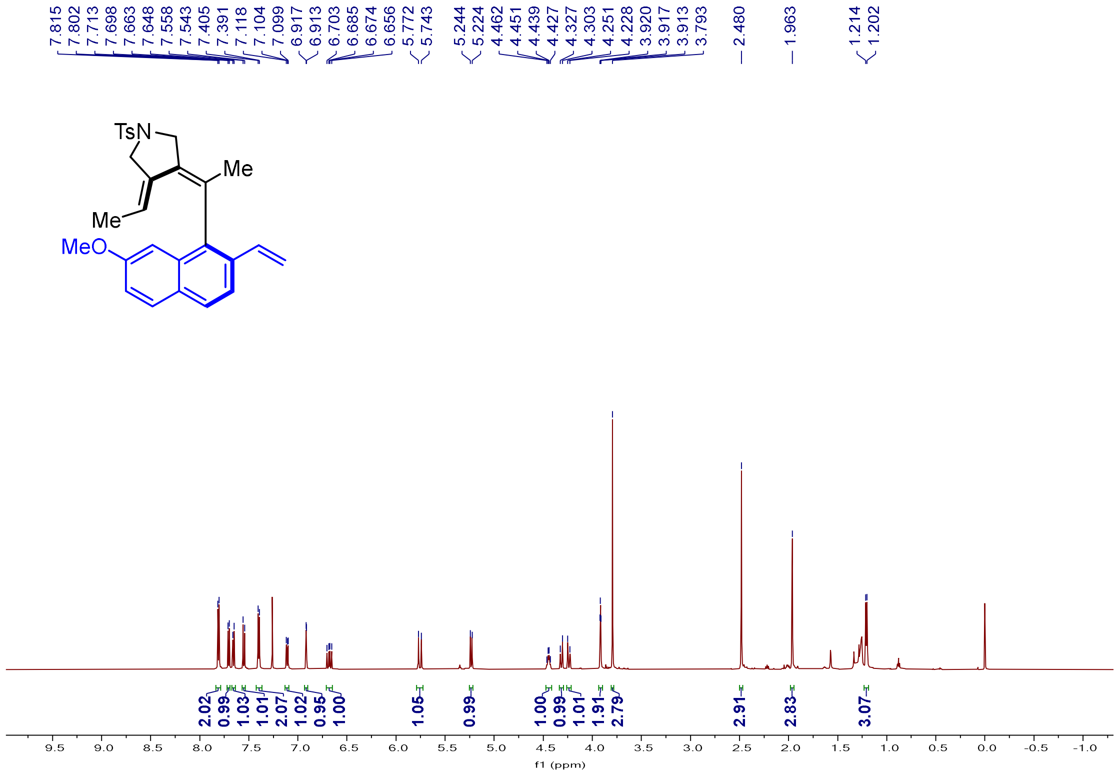

^{13}C NMR (150 MHz, Chloroform-d) spectrum of 79

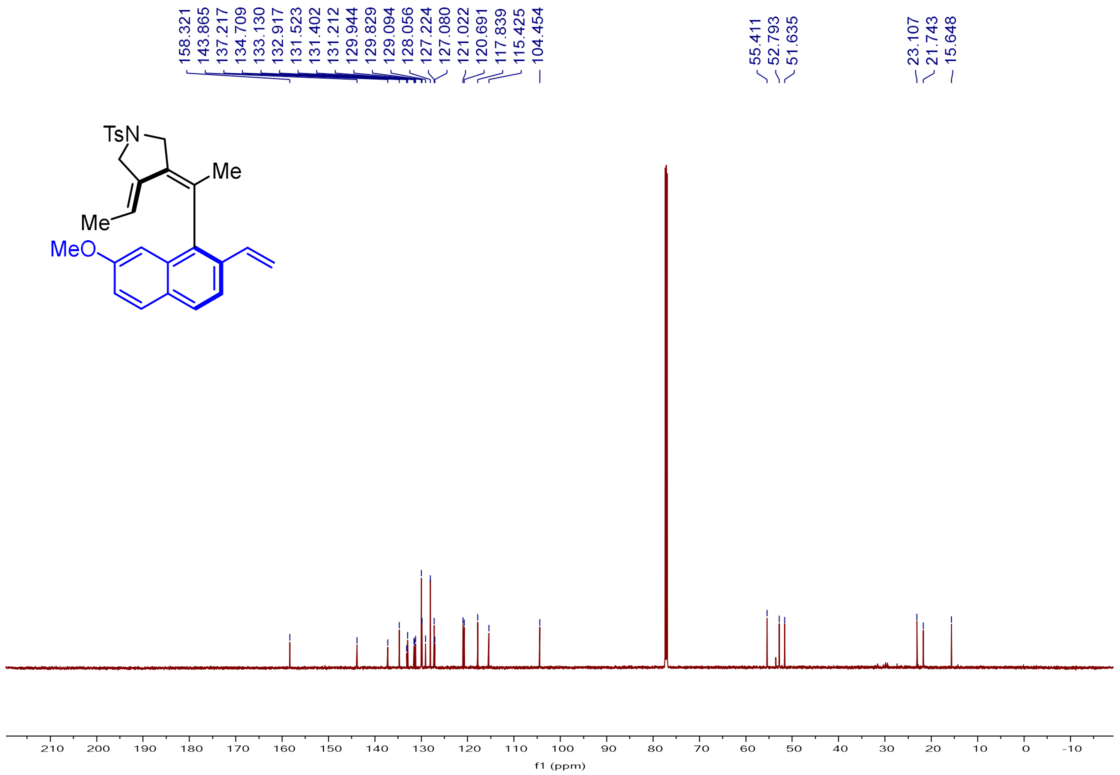

^{1}H NMR (600 MHz, Chloroform-d) spectrum of 80

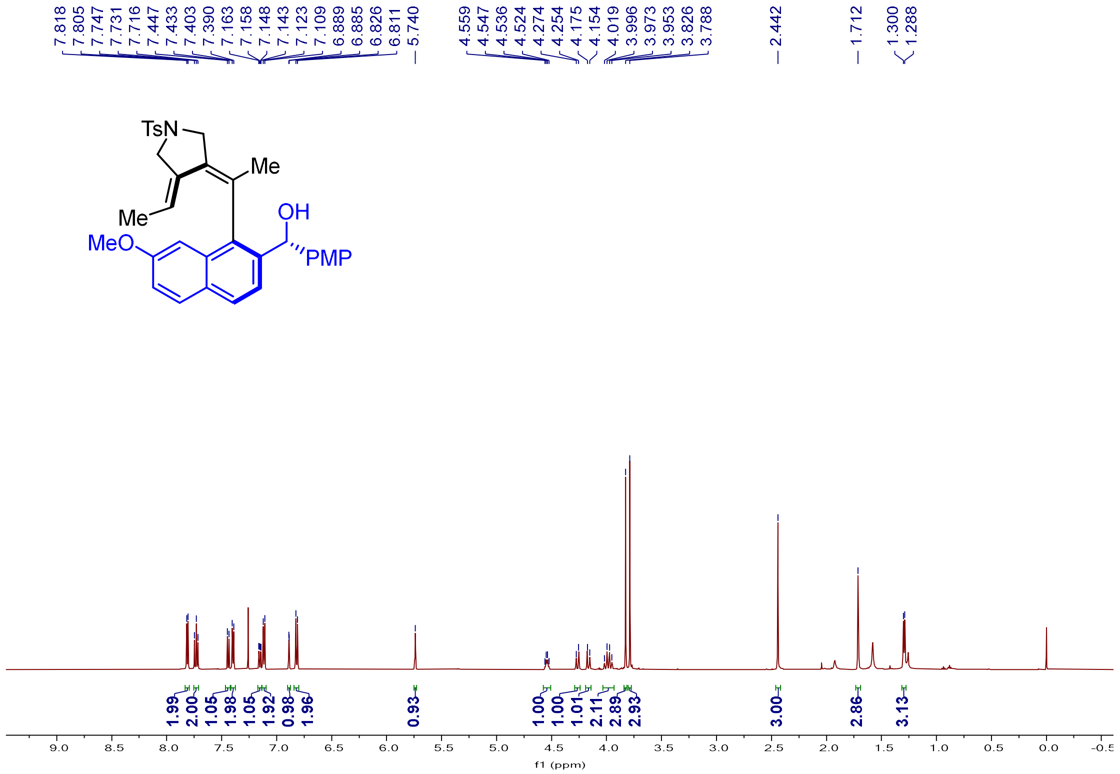

^{13}C NMR (150 MHz, Chloroform-d) spectrum of 80

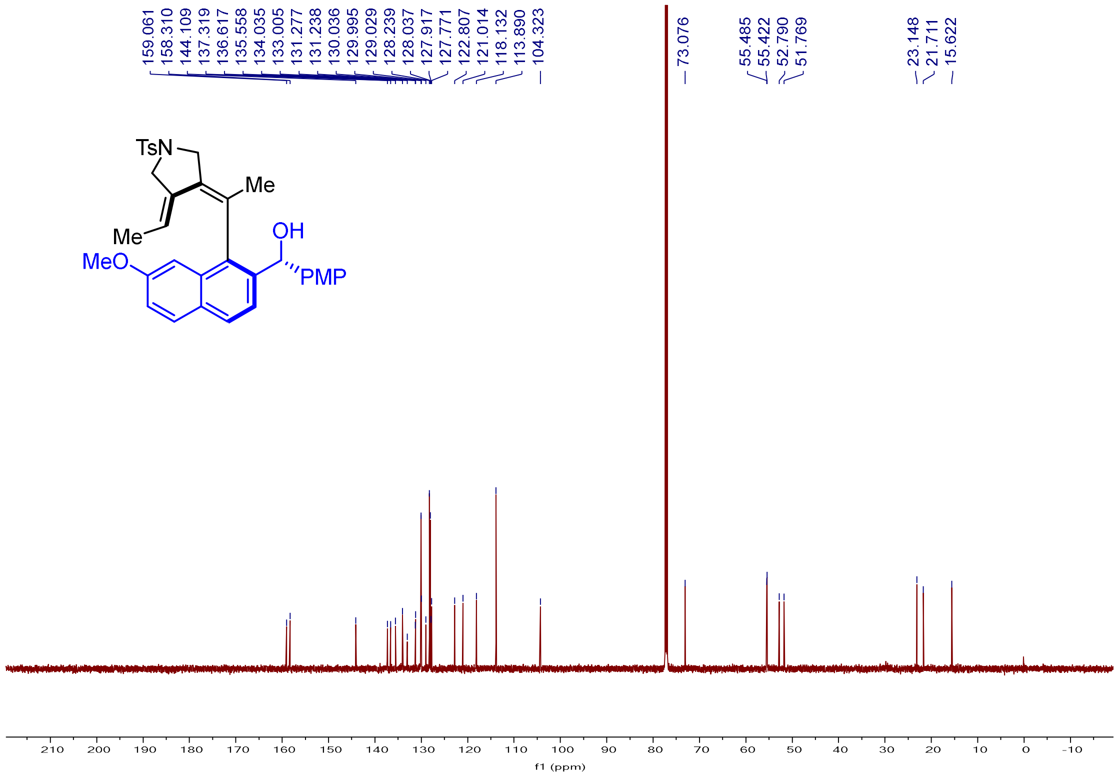

^{1}H NMR (600 MHz, Chloroform-d) spectrum of 81

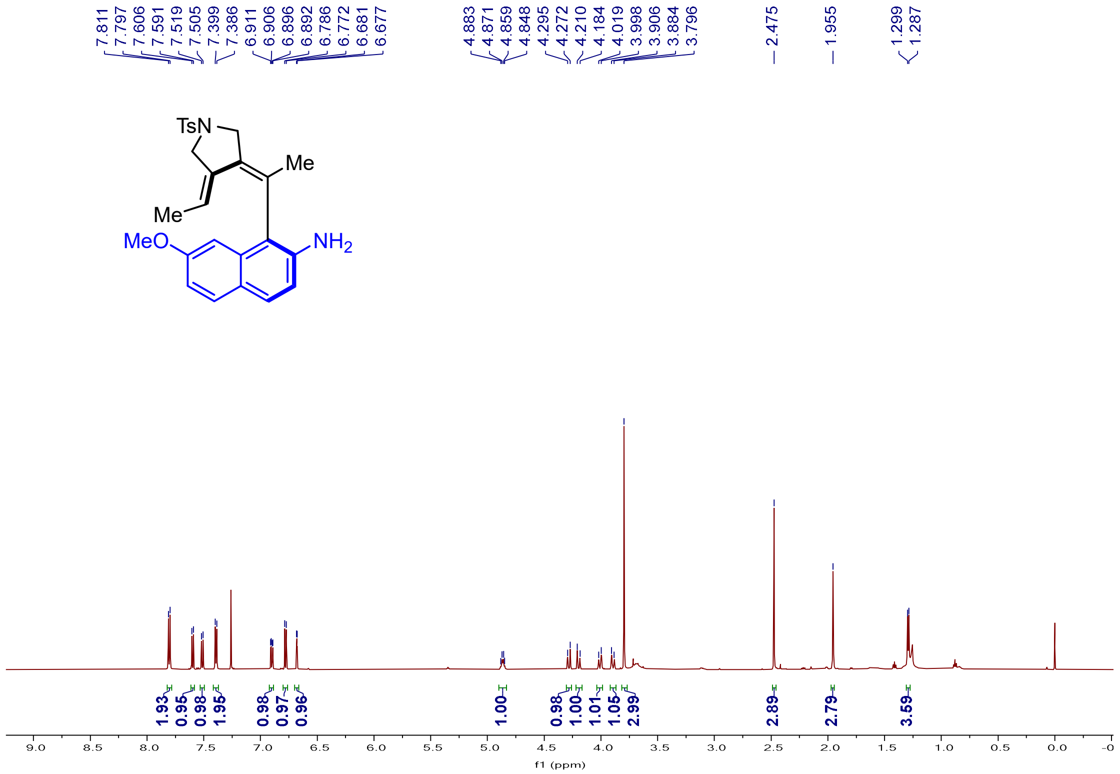

^{13}C NMR (150 MHz, Chloroform-d) spectrum of 81

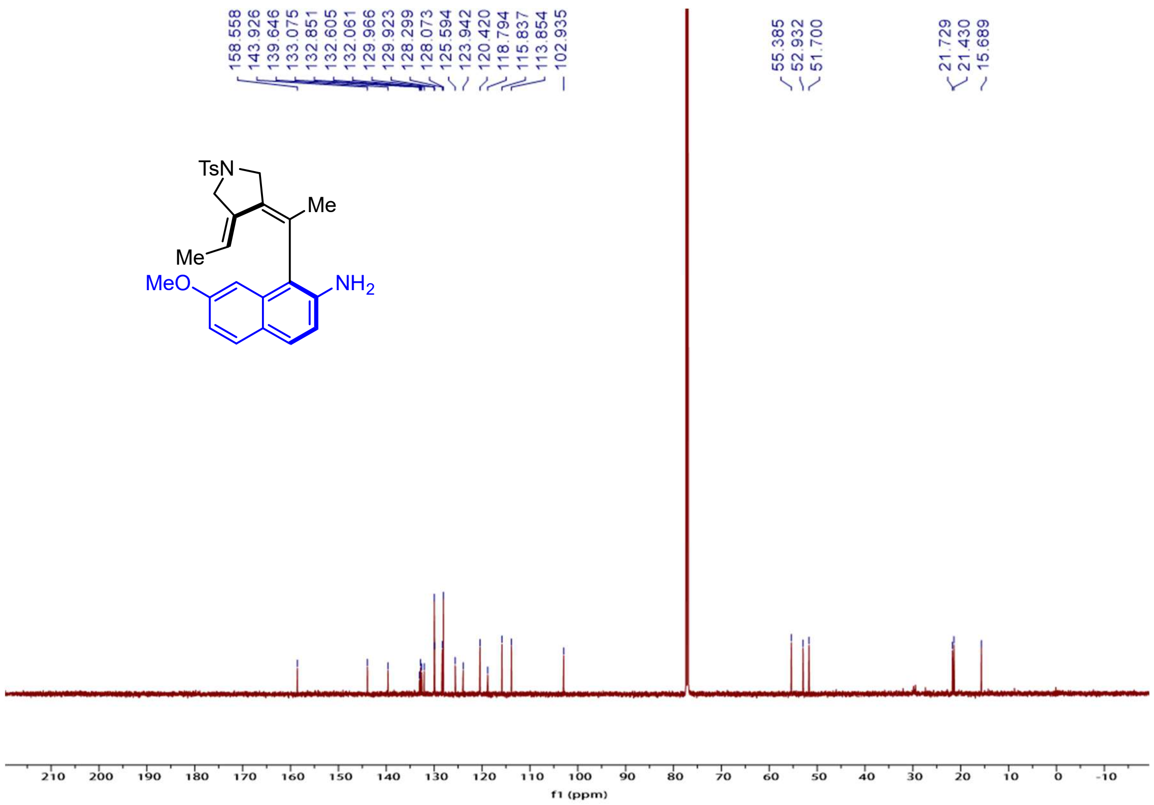

^{1}H NMR (600 MHz, Chloroform-d) spectrum of 82

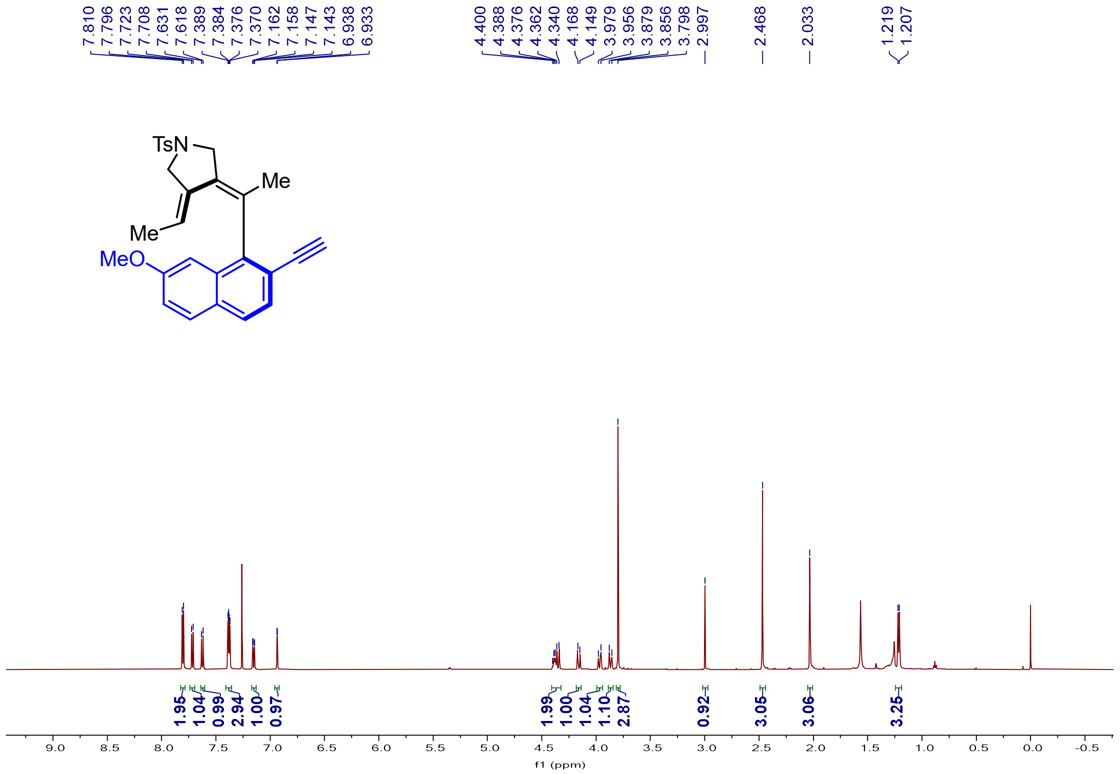

^{13}C NMR (150 MHz, Chloroform-d) spectrum of 82

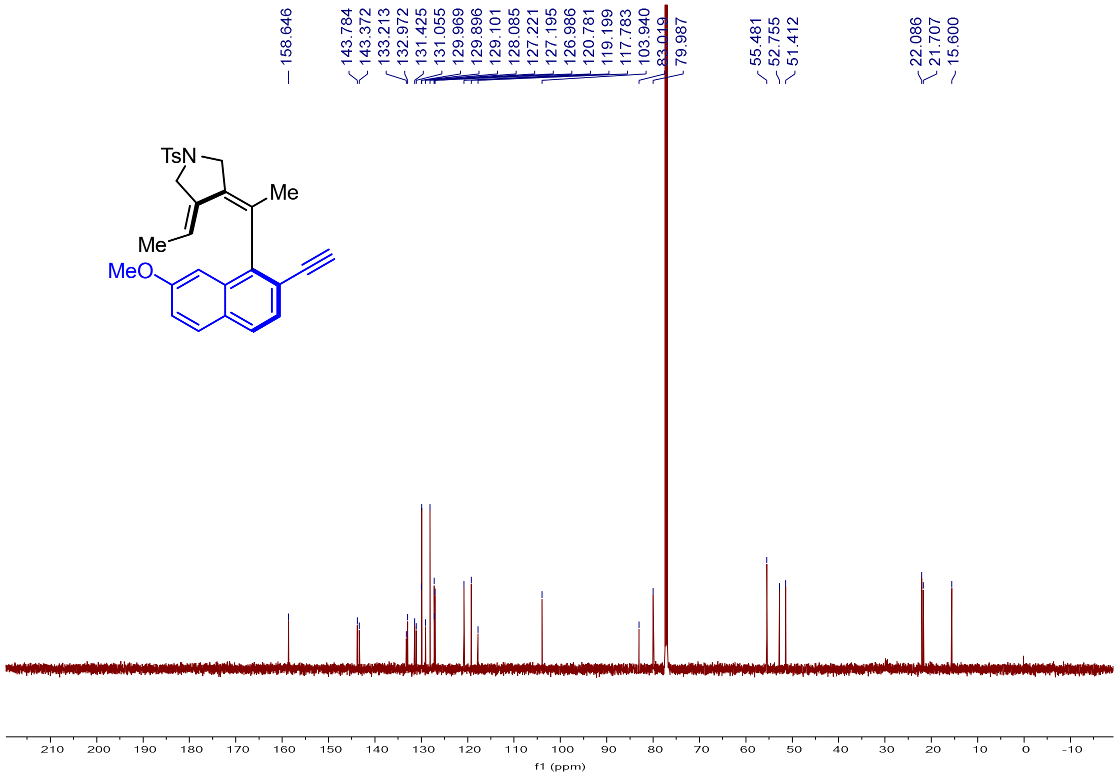

^{1}H NMR (600 MHz, Chloroform-d) spectrum of 83

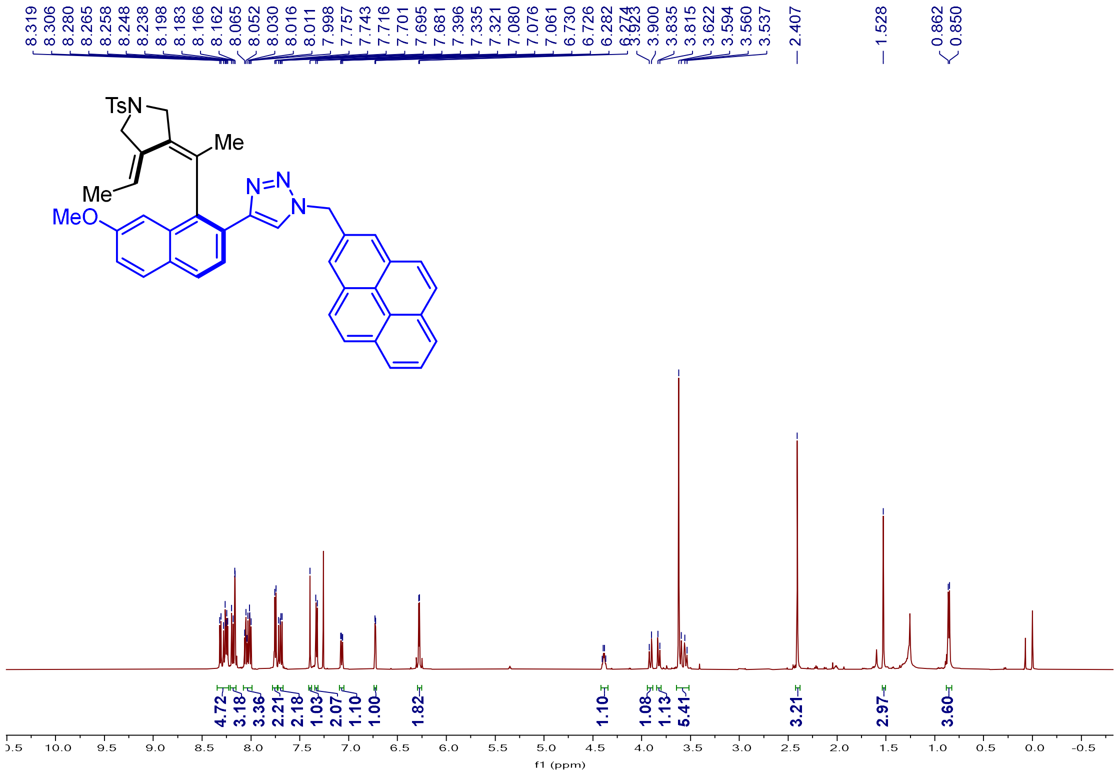

^{13}C NMR (150 MHz, Chloroform-d) spectrum of 83

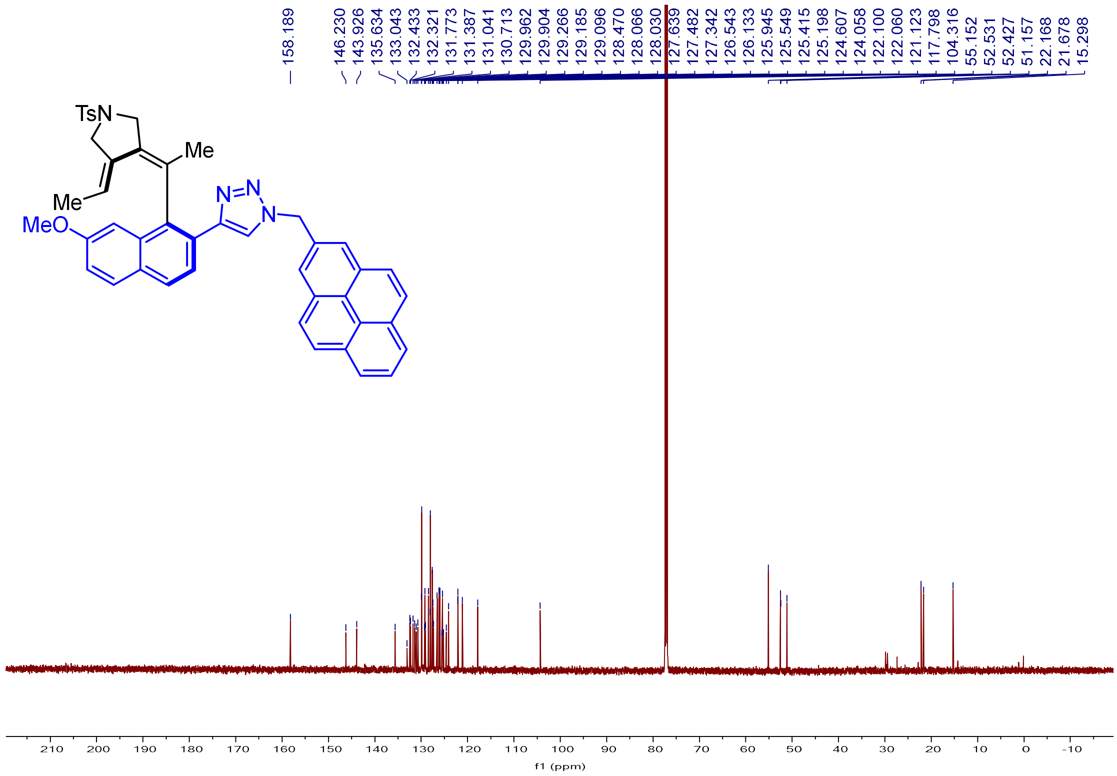

^{1}H NMR (600 MHz, Chloroform-d) spectrum of 84

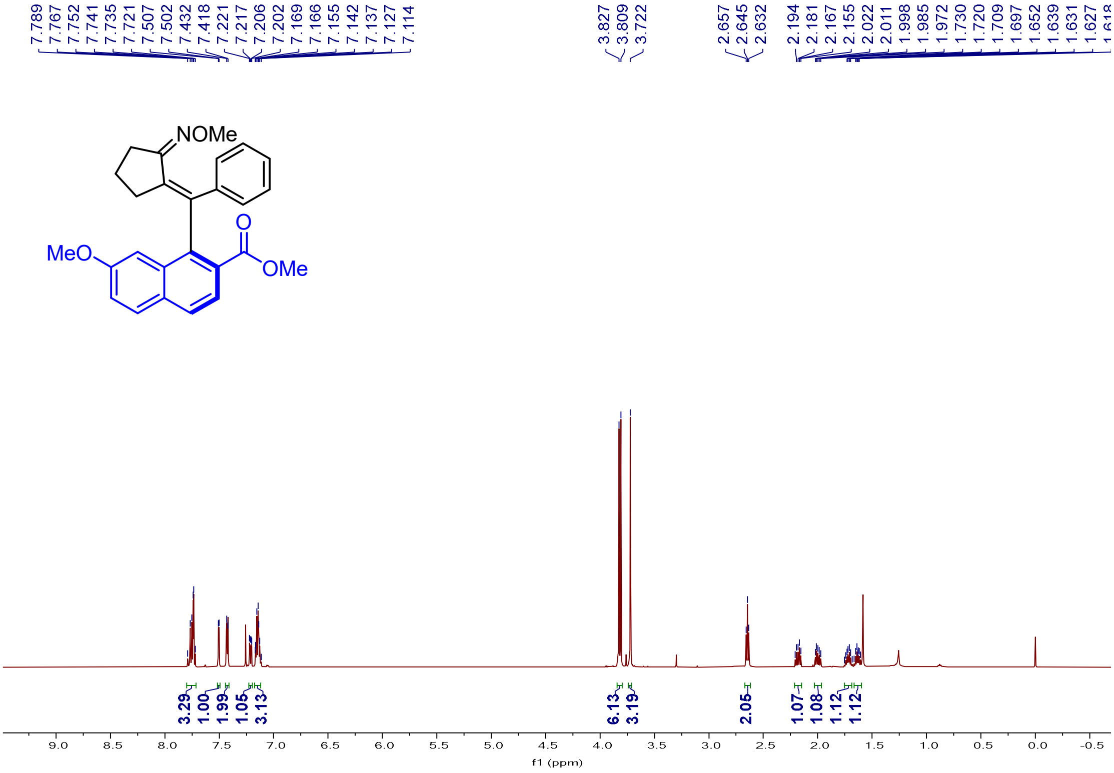

^{13}C NMR (150 MHz, Chloroform-d) spectrum of 84

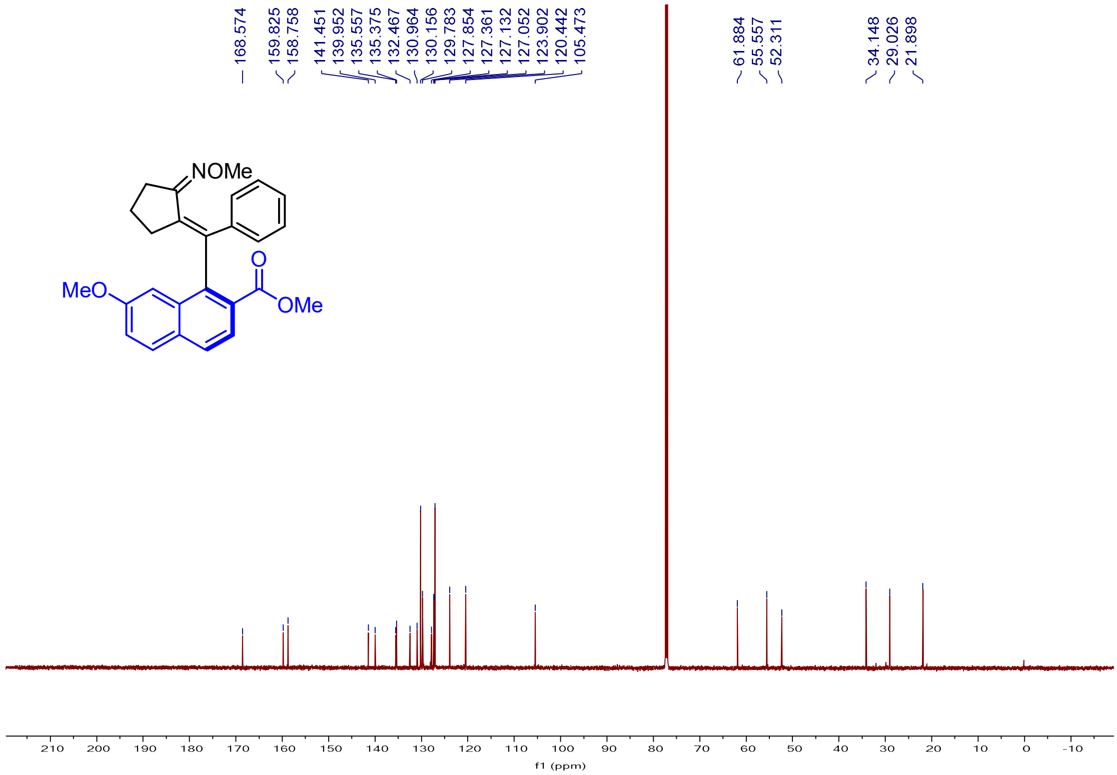

^{1}H NMR (600 MHz, Chloroform-d) spectrum of 85

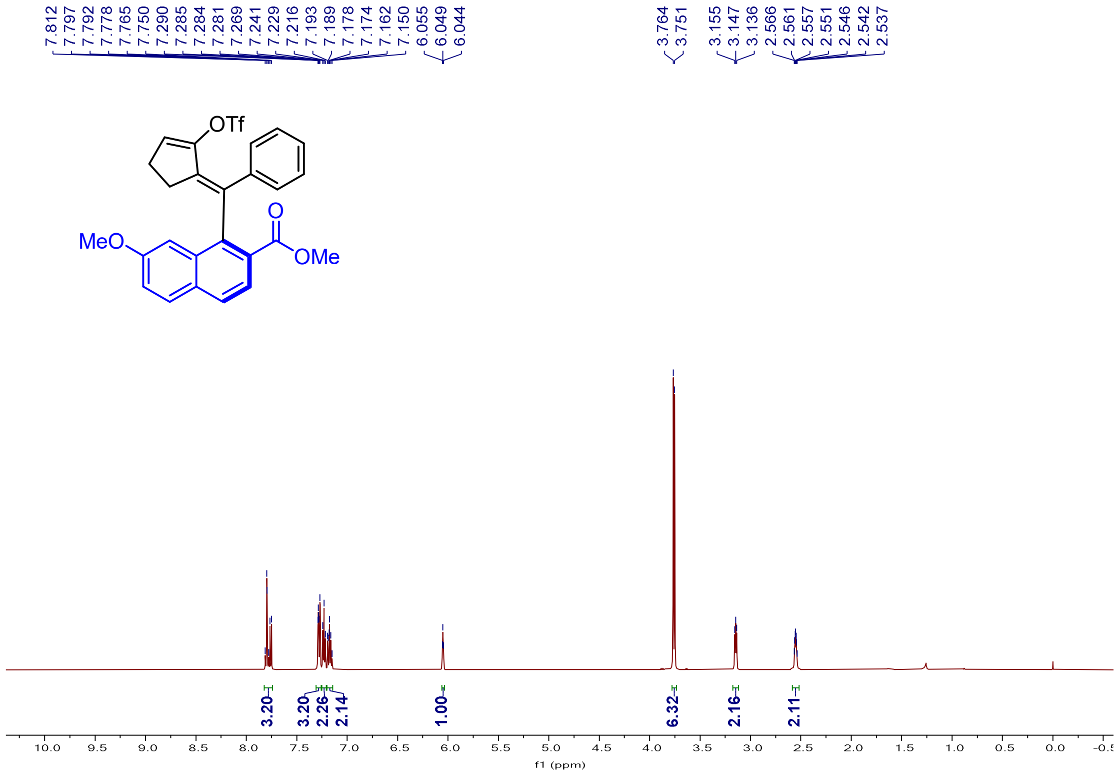

^{13}C NMR (150 MHz, Chloroform-d) spectrum of 85

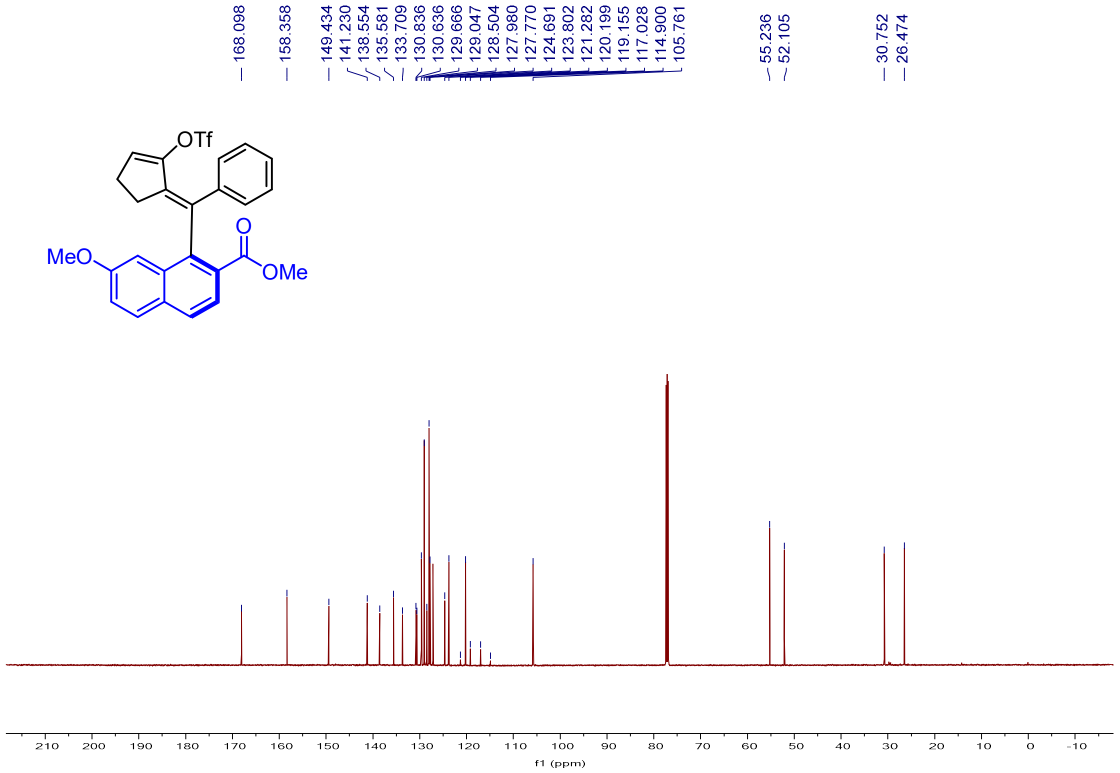

^{19}F NMR (376 MHz, Chloroform-d) spectrum of 85

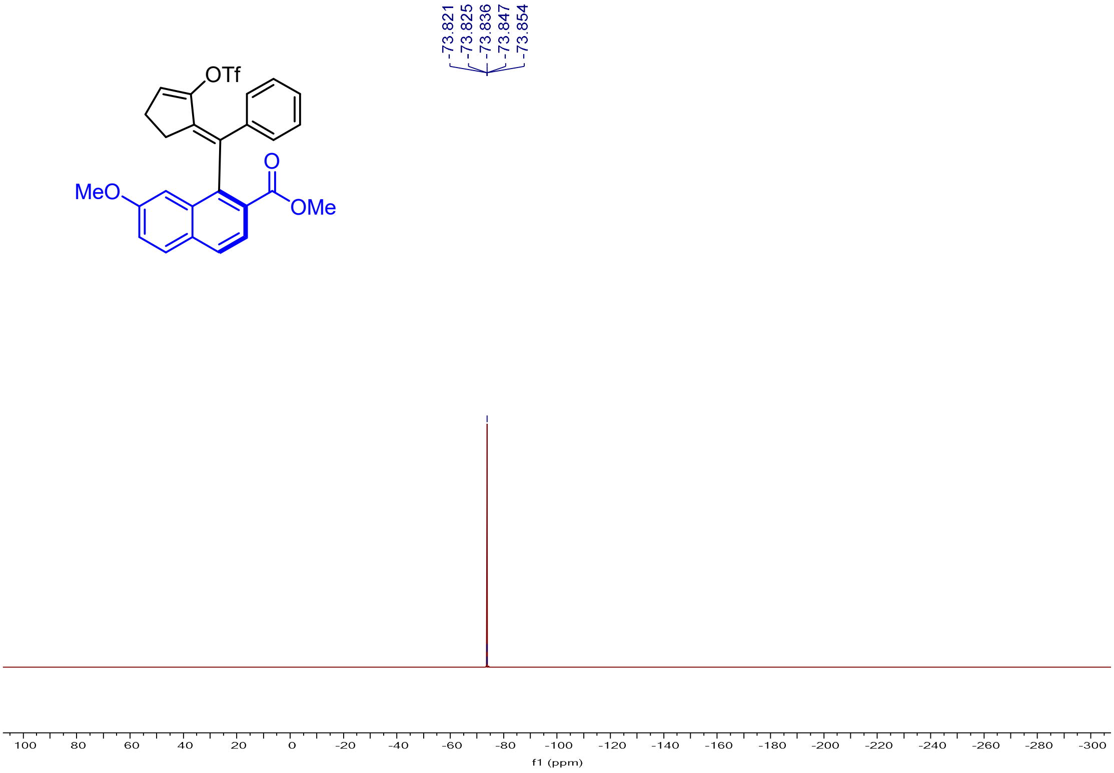

^{1}H NMR (600 MHz, Chloroform-d) spectrum of 86

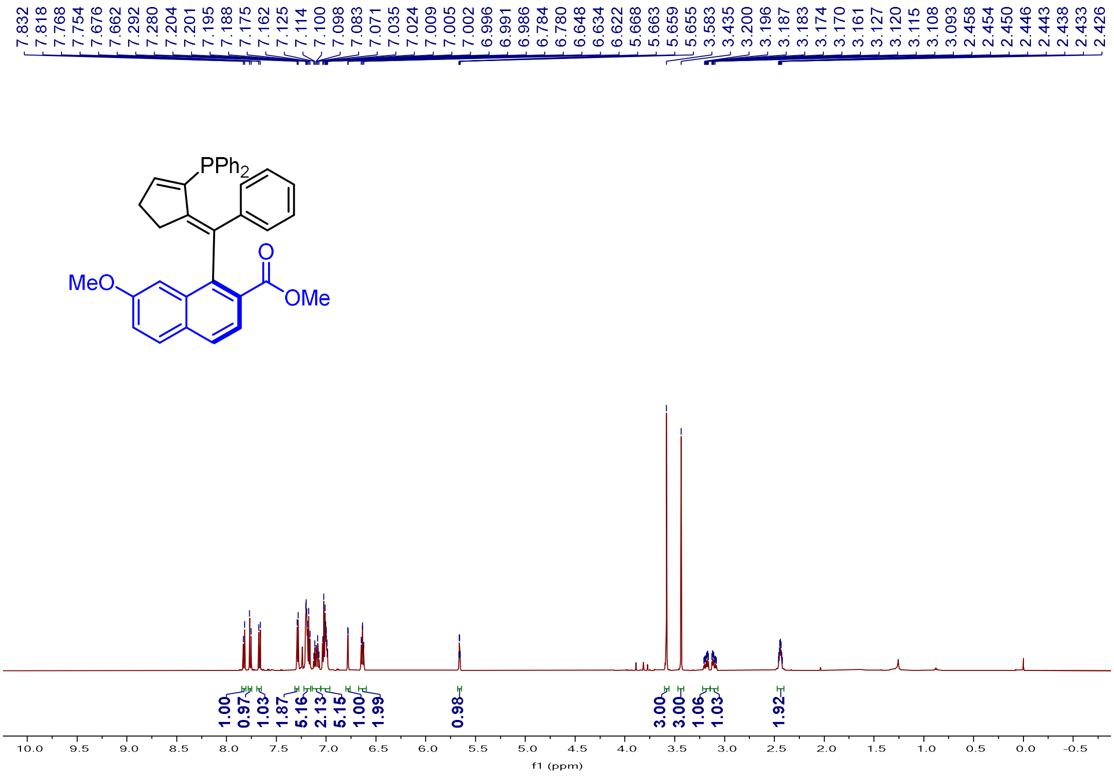

^{13}C NMR (150 MHz, Chloroform-d) spectrum of 86

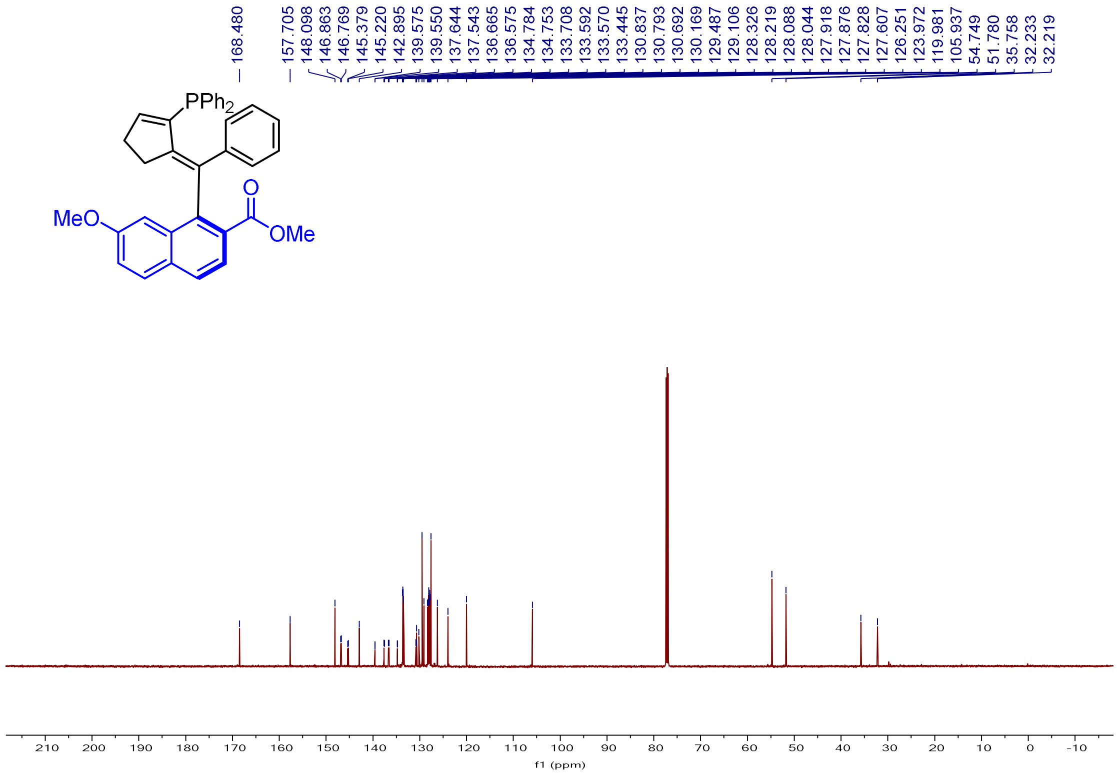

^{31}P NMR (162 MHz, Chloroform-d) spectrum of 86

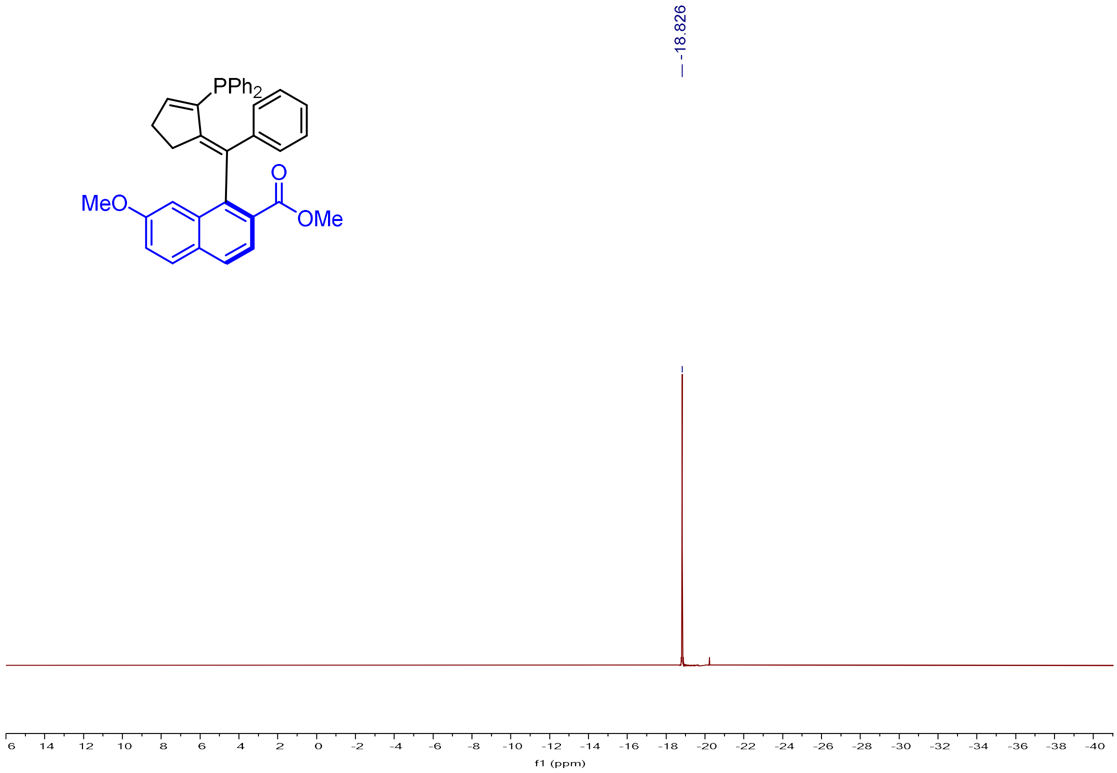

^{1}H NMR (600 MHz, Chloroform-d) spectrum of 87

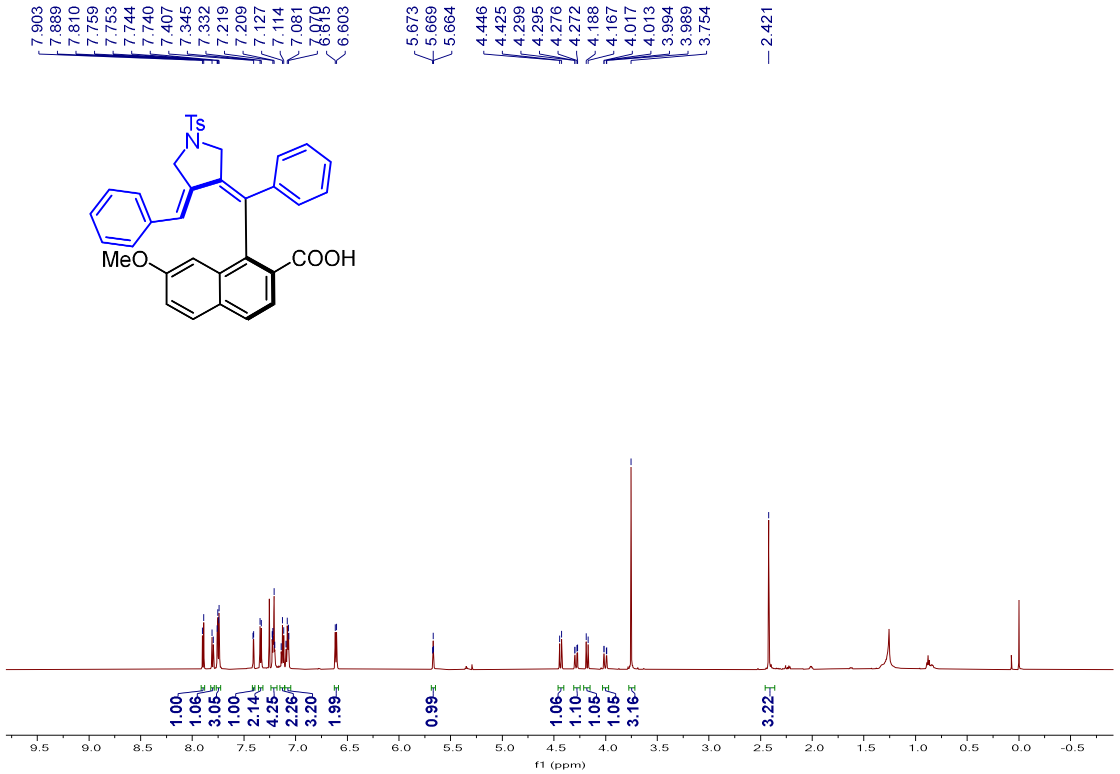

^{13}C NMR (150 MHz, Chloroform-d) spectrum of 87

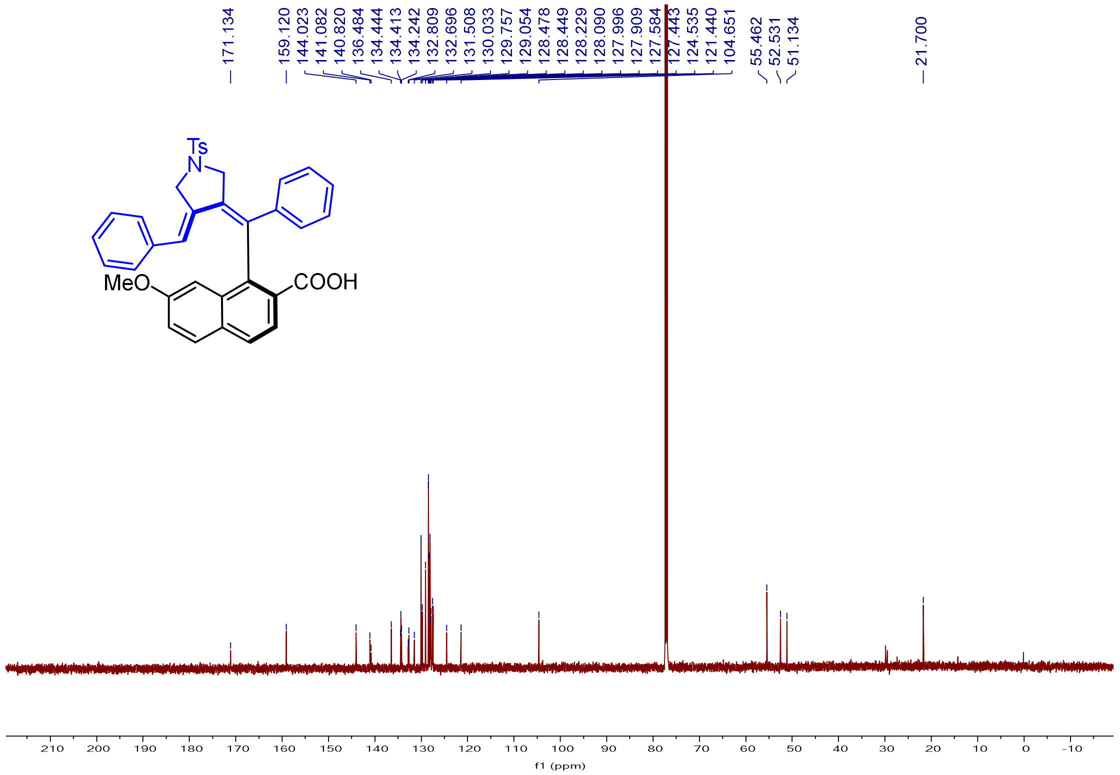

^{1}H NMR (600 MHz, Chloroform-d) spectrum of 88

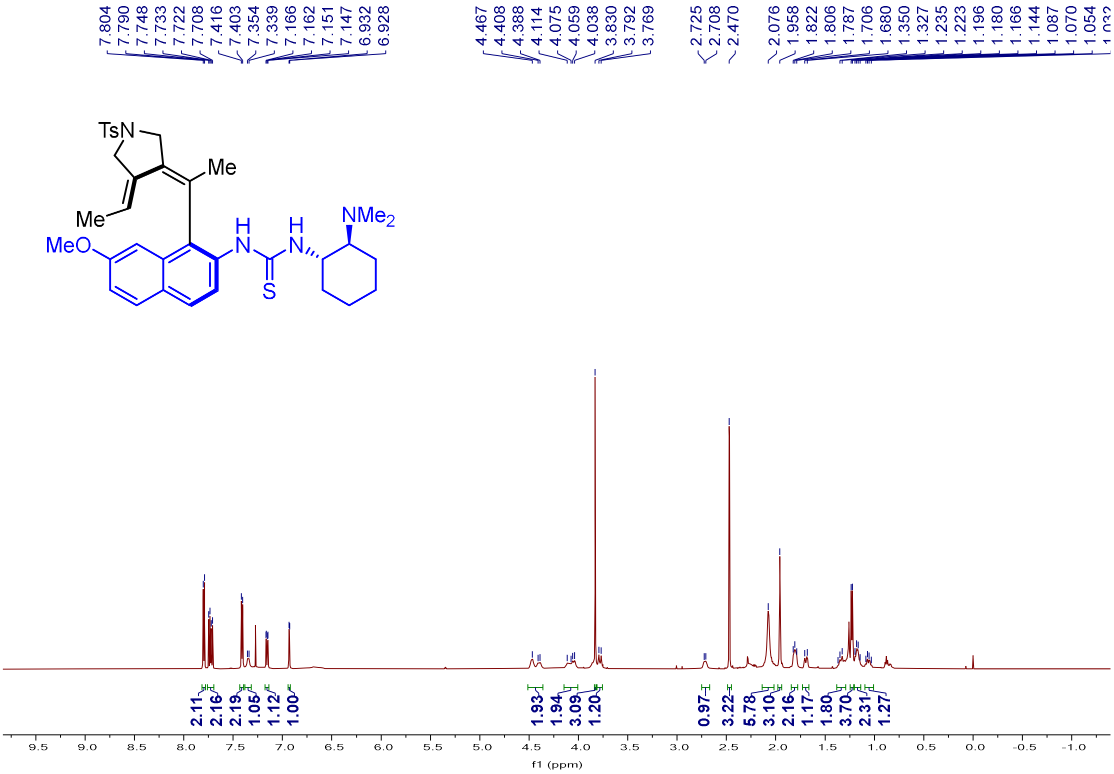

^{13}C NMR (150 MHz, Chloroform-d) spectrum of 88

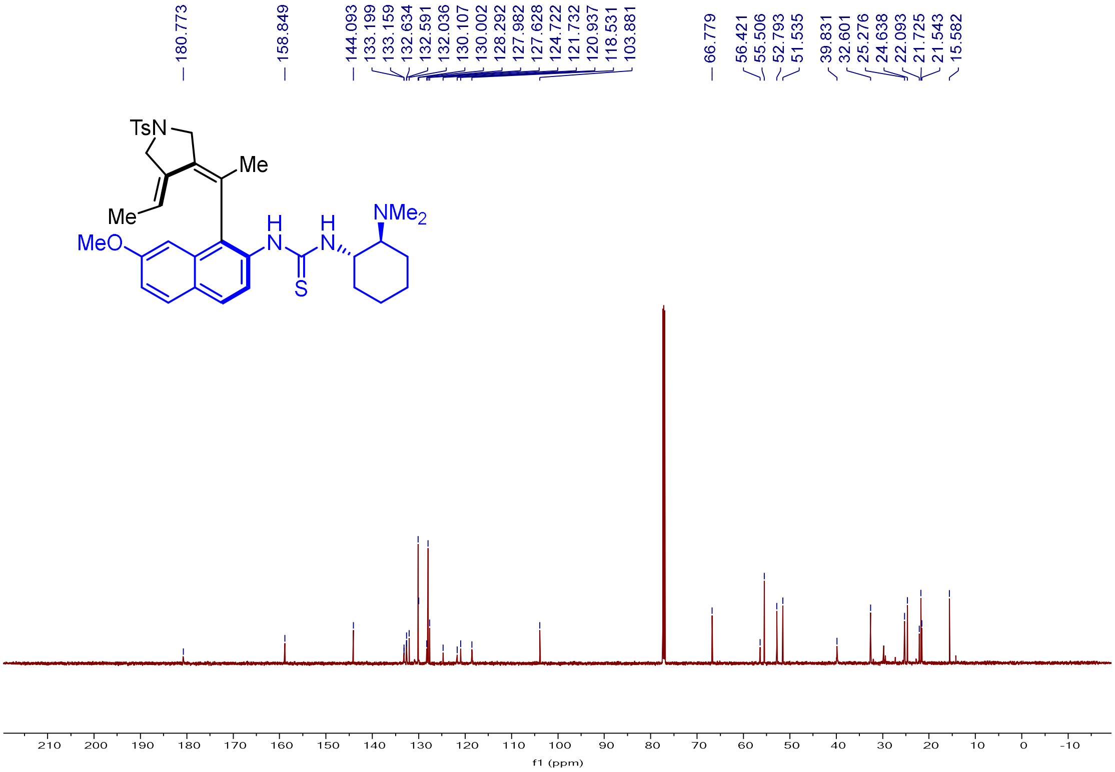

^{1}H NMR (600 MHz, Chloroform-d) spectrum of 91

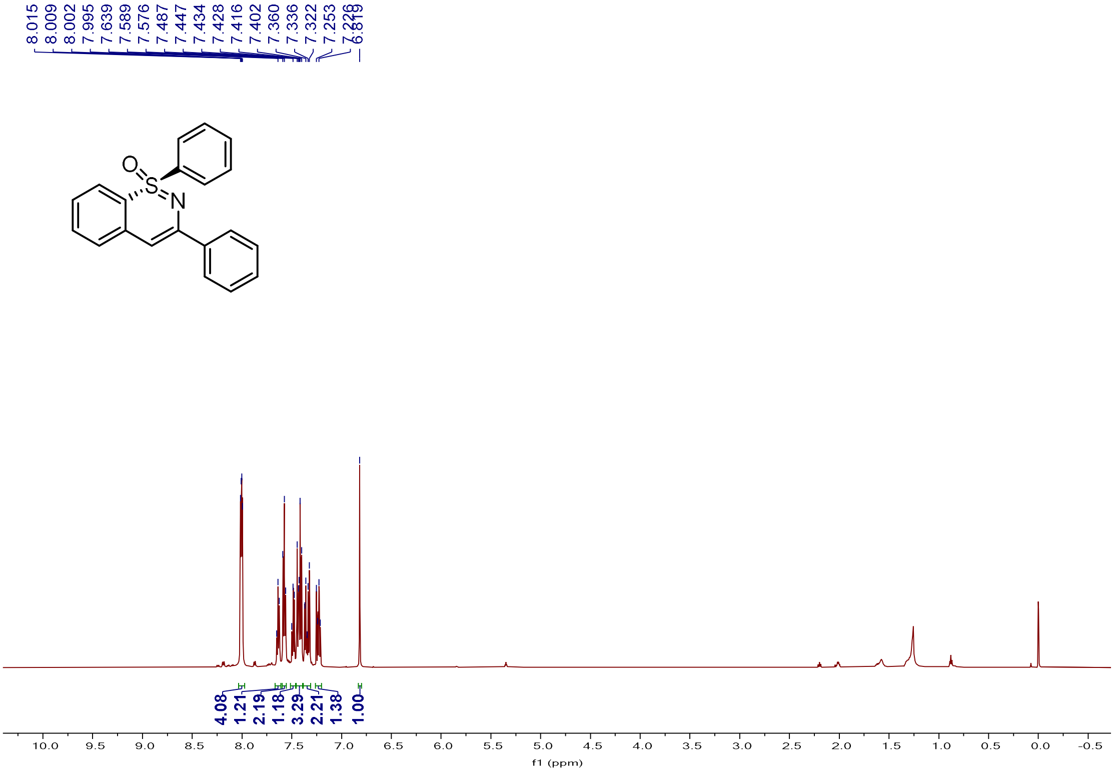

^{13}C NMR (150 MHz, Chloroform-d) spectrum of 91

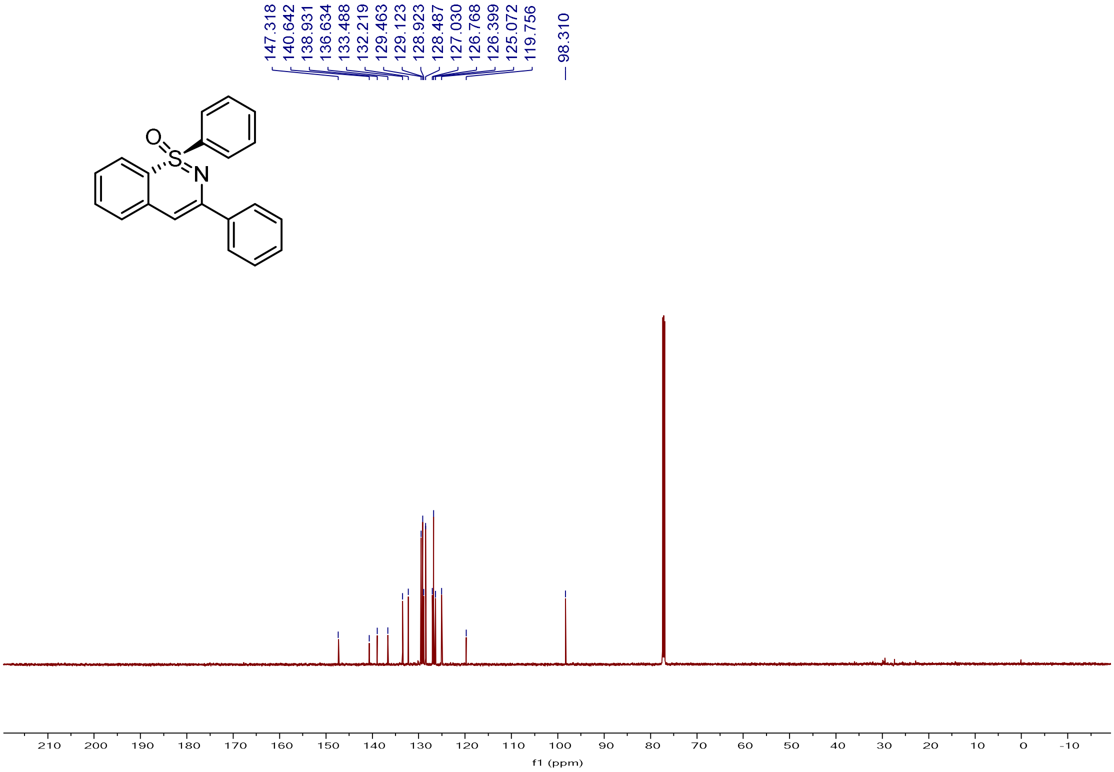

^{1}H NMR (600 MHz, Chloroform-d) spectrum of 92

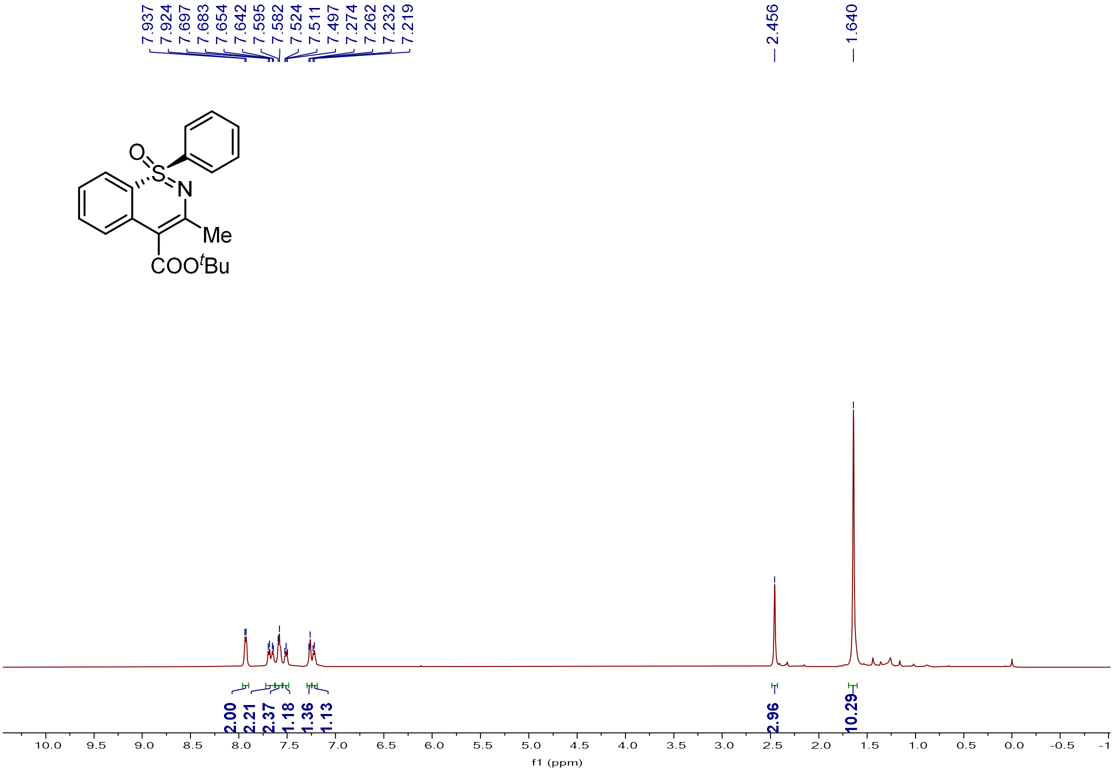

^{13}C NMR (150 MHz, Chloroform-d) spectrum of 92

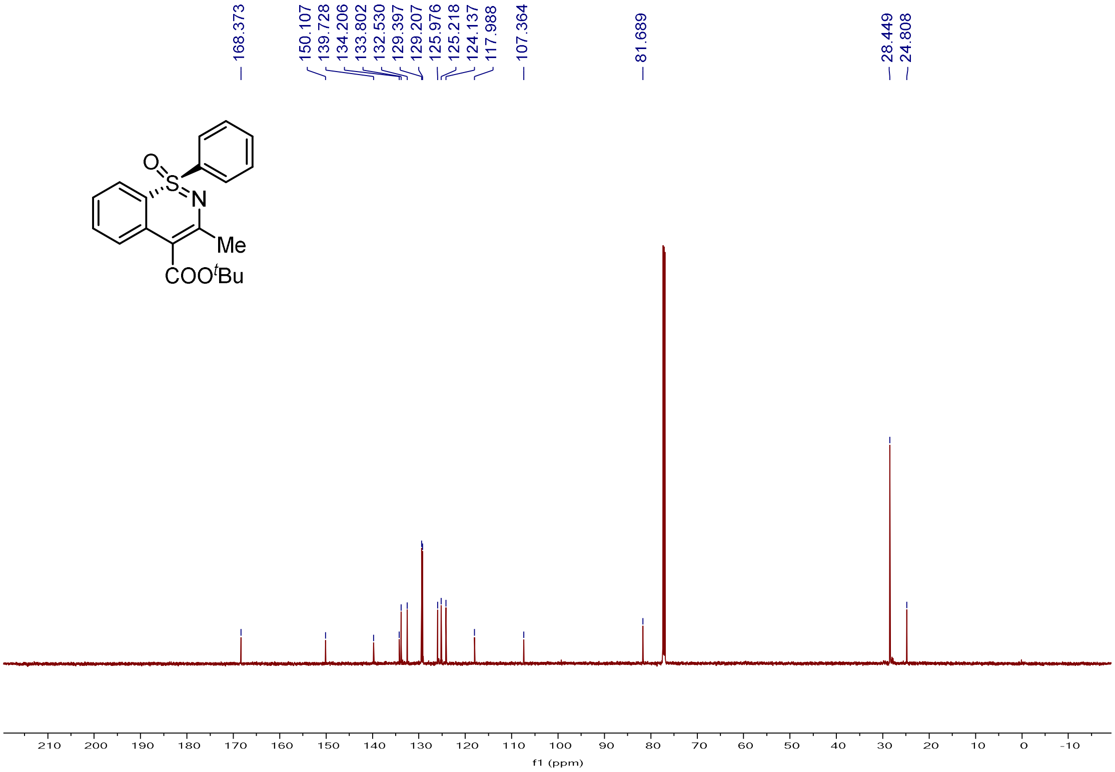

^{1}H NMR (600 MHz, Chloroform-d) spectrum of 96

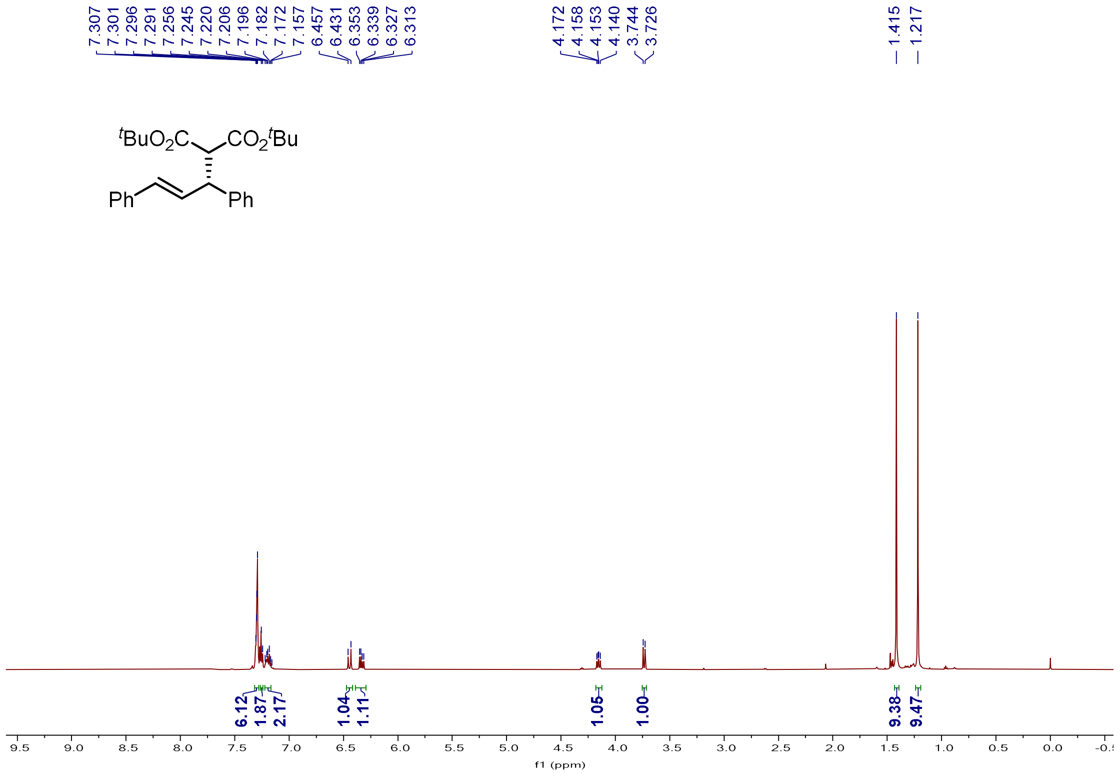

^{13}C NMR (150 MHz, Chloroform-d) spectrum of 96

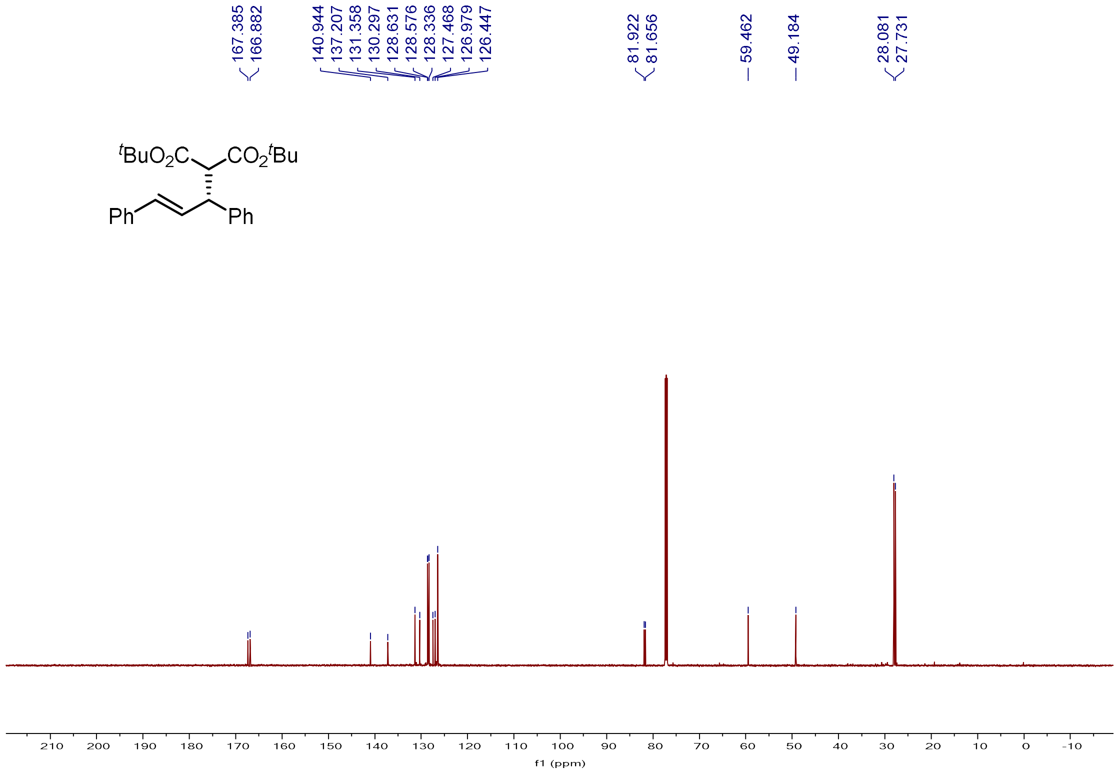

^1H NMR (600 MHz, Chloroform-d) spectrum of 99

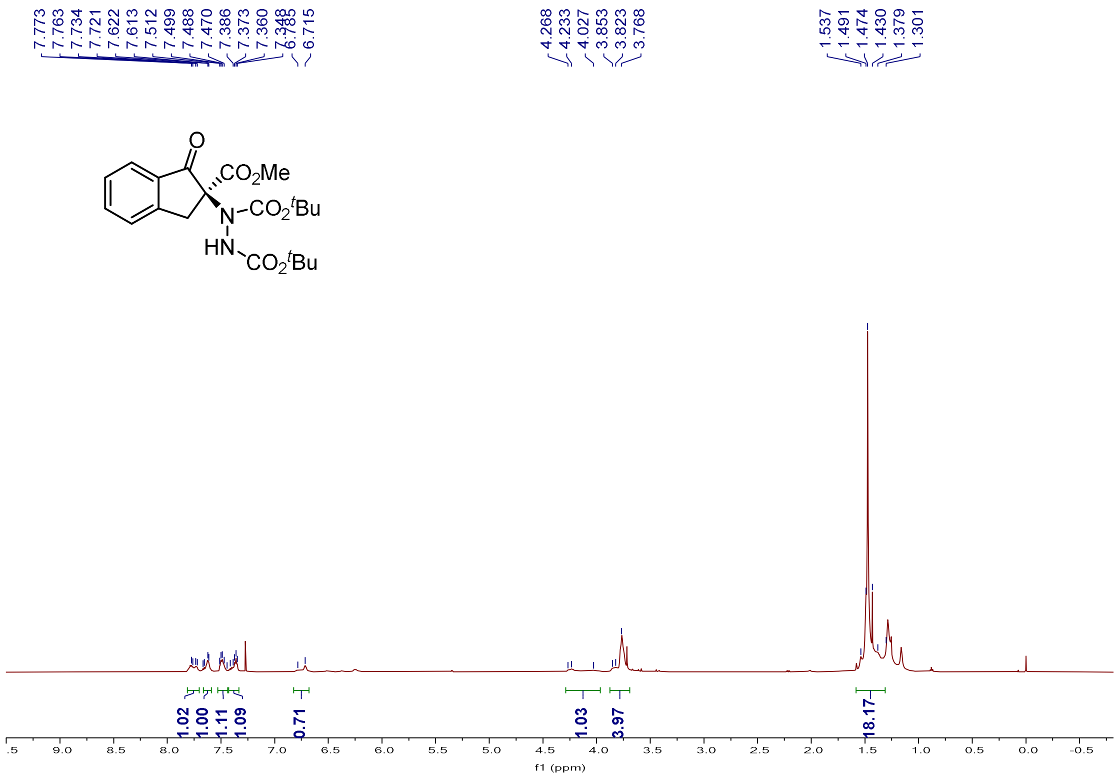

Supplement: SC-016-D5SC04080A-s001 [file SC-016-D5SC04080A-s001.pdf]
